# Author Correction: Regulatory genomic circuitry of human disease loci by integrative epigenomics

**DOI:** 10.1038/s41586-025-09134-4

**Published:** 2025-06-20

**Authors:** Carles A. Boix, Benjamin T. James, Yongjin P. Park, Wouter Meuleman, Manolis Kellis

**Affiliations:** 1 Computer Science and Artificial Intelligence Laboratory, Massachusetts Institute of Technology, Cambridge, MA USA; 2 Broad Institute of MIT and Harvard, Cambridge, MA USA; 3 Computational and Systems Biology Program, Massachusetts Institute of Technology, Cambridge, MA USA; 4 Department of Pathology and Laboratory Medicine, University of British Columbia, Vancouver, British Columbia Canada; 5 Altius Institute for Biomedical Sciences, Seattle, WA USA

**Keywords:** Development, Gene regulation, Genome-wide association studies, Epigenomics

Correction to: *Nature* 10.1038/s41586-020-03145-z Published online 3 February 2021

In the version of the article initially published, arising from an implementation mistake, the false discovery rate (FDR) in the analysis of epigenomic enrichments for GWAS loci was not correctly controlled, leading to inflated enrichment statistics due to double-counting of enhancer-SNP overlaps in the case of multiple nearby enhancers from the same tissue.

Specifically, our enrichment analysis between tissue/biosample enhancers and GWAS trait SNPs sought to match the Roadmap Epigenomics analysis, but we made two implementation mistakes. The first mistake was that we calculated the enrichment in the wrong direction: instead of calculating the enrichment of SNPs in biosample enhancers as was previously done in Roadmap, we mistakenly calculated the enrichment of enhancers in GWAS lead SNPs. This led to double-counting SNPs and inflated enrichment statistics in the case of multiple nearby enhancers within 2.5 kb of the same lead SNP, which were all counted as independently overlapping the same SNP, when in fact the multiple overlaps came from their genomic clustering in the same locus. The second mistake was that our FDR procedure only controlled for inflation due to multiple enhancers proximal to the same SNP, but not for the multiple hypothesis testing across many tissue-trait combinations, and thus our reported statistics correspond to nominal *P* values rather than FDR-corrected *P* values. This issue affected the section “Interpreting GWAS loci,” and the figures and sections that depended on it.

To correct this issue, we have re-calculated all GWAS enrichments in the paper to assess the enrichment of GWAS SNPs in enhancers, which calculates the enrichment in the opposite direction, and doesn’t lead to double-counting (as a given SNP counts as ‘overlapping’ a biosample if it hits any enhancer from that biosample, and thus multiple overlapping enhancers do not pose an issue). This analysis has been properly FDR controlled and results in 226 GWA studies at FDR of 1% for the flat epigenomes analysis (instead of the previously reported “245 at 1% FDR”).

Following the revised analysis, the GWAS-relevant Results sections have been rewritten, the GWAS Methods sections have been updated, and GWAS numbers have been updated in the Abstract and Discussion; and Figs. 3 and 4, Extended Data Figs. 9–12, Supplementary Figs. 18–24, 26 and the Supplementary Data have been updated. Reference 36 in the paper has been updated to Nagel, M. et al. Meta-analysis of genome-wide association studies for neuroticism in 449,484 individuals identifies novel genetic loci and pathways. *Nat. Genet.* **50**, 920–927 (2018). To allow transparent comparison, the original article and Supplementary Figures and Data are available as Supplementary Information accompanying this amendment.

**Supplementary information** is available in the online version of this amendment.

## Supplementary information

Original, uncorrected article

Original, uncorrected Supplementary Figures and Supplementary Data

