# Supplementary material for: Regulatory genomic circuitry of human disease loci by integrative epigenomics
Source: Nature. 2021 Feb 3;590(7845):300–7. doi: 10.1038/s41586-020-03145-z (PMC7875769; doi:10.1038/s41586-020-03145-z)
Supplement: Supplementary file 1 — Supplementary Figures S1-S27. [file 41586_2020_3145_MOESM1_ESM.pdf]

nature portfolio

https://doi.org/10.1038/s41586-020-03145-z

Supplementary information

Regulatory genomic circuitry of human disease loci by integrative epigenomics

In the format provided by the authors and unedited

#	Group	BSSID	Extended Info
1	Adipose	BSS00038	ADIPOCYTE
2	Adipose	BSS00043	ADIPOSE TISSUE
3	Adipose	BSS01671	ADIPOSE TISSUE
4	Adipose	BSS01665	ADIPOSE TISSUE
5	Adipose	BSS01666	ADIPOSE TISSUE
6	Adipose	BSS01668	ADIPOSE TISSUE
7	Adipose	BSS01669	ADIPOSE TISSUE
8	Adipose	BSS01667	ADIPOSE TISSUE
9	Adipose	BSS01394	OMENTAL FAT PAD
10	Adipose	BSS01393	OMENTAL FAT PAD
11	Blood & T-cell	BSS00188	CD4 T CELL
12	Blood & T-cell	BSS00185	CD4 T CELL
13	Blood & T-cell	BSS00189	CD4 T CELL
14	Blood & T-cell	BSS00190	CD4 T CELL
15	Blood & T-cell	BSS00183	CD4 T CELL
16	Blood & T-cell	BSS00186	CD4 T CELL
17	Blood & T-cell	BSS00191	CD4 T CELL
18	Blood & T-cell	BSS00192	CD4 T CELL
19	Blood & T-cell	BSS00274	CD4 T CELL
20	Blood & T-cell	BSS00195	CD8 T CELL
21	Blood & T-cell	BSS00196	CD8 T CELL
22	Blood & T-cell	BSS00198	CD8 T CELL
23	Blood & T-cell	BSS00200	CD8 T CELL
24	Blood & T-cell	BSS00193	CD8 T CELL
25	Blood & T-cell	BSS00194	CD8 T CELL
26	Blood & T-cell	BSS00197	CD8 T CELL
27	Blood & T-cell	BSS01420	MONONUCLEAR CELL
28	Blood & T-cell	BSS01421	MONONUCLEAR CELL
29	Blood & T-cell	BSS01279	MONONUCLEAR CELL
30	Blood & T-cell	BSS01419	MONONUCLEAR CELL
31	Blood & T-cell	BSS01423	MONONUCLEAR CELL
32	Blood & T-cell	BSS01424	MONONUCLEAR CELL
33	Blood & T-cell	BSS01347	NAIVE T CELL
34	Blood & T-cell	BSS01348	NAIVE T CELL
35	Blood & T-cell	BSS01346	NAIVE T CELL
36	Blood & T-cell	BSS01688	T CELL
37	Blood & T-cell	BSS01687	T CELL
38	Blood & T-cell	BSS01689	T CELL
39	Blood & T-cell	BSS01684	T CELL
40	Blood & T-cell	BSS01691	T1 CELL
41	Blood & T-cell	BSS01692	T1 CELL
42	Blood & T-cell	BSS01690	T1 CELL
43	Blood & T-cell	BSS01693	T17 CELL
44	Blood & T-cell	BSS01694	T17 CELL
45	Blood & T-cell	BSS01695	T17 CELL
46	Blood & T-cell	BSS01697	T2 CELL
47	Blood & T-cell	BSS01698	T2 CELL
48	Blood & T-cell	BSS01696	T2 CELL
49	Blood & T-cell	BSS01478	TREG CELL
50	Blood & T-cell	BSS01479	TREG CELL
51	Blood & T-cell	BSS01480	TREG CELL
52	Bone	BSS00084	BONE ARM
53	Bone	BSS00330	BONE FEMUR
54	Bone	BSS01154	BONE LEG
55	Bone	BSS00705	BONE MARROW STROMA
56	Bone	BSS01397	OSTEOBLAST
57	Brain	BSS00071	AMMONS HORN
58	Brain	BSS00077	ANGULAR GYRUS
59	Brain	BSS00078	ANGULAR GYRUS
60	Brain	BSS00089	ASTROCYTE
61	Brain	BSS00090	ASTROCYTE CEREBELLUM
62	Brain	BSS00091	ASTROCYTE HIPPOCAMPUS
63	Brain	BSS00092	ASTROCYTE SPINAL CORD
64	Brain	BSS00135	BRAIN
65	Brain	BSS00136	BRAIN
66	Brain	BSS00129	BRAIN
67	Brain	BSS00130	BRAIN
68	Brain	BSS00131	BRAIN
69	Brain	BSS00133	BRAIN
70	Brain	BSS00138	BRAIN
71	Brain	BSS00139	BRAIN
72	Brain	BSS00140	BRAIN
73	Brain	BSS00142	BRAIN
74	Brain	BSS00126	BRAIN
75	Brain	BSS00127	BRAIN
76	Brain	BSS00125	BRAIN
77	Brain	BSS00132	BRAIN
78	Brain	BSS00134	BRAIN
79	Brain	BSS00141	BRAIN
80	Brain	BSS00174	CAUDATE NUCLEUS
81	Brain	BSS00175	CAUDATE NUCLEUS
82	Brain	BSS00173	CAUDATE NUCLEUS
83	Brain	BSS00201	CEREBELLAR CORTEX
84	Brain	BSS00205	CEREBELLUM
85	Brain	BSS00207	CEREBELLUM

#	Group	BSSID	Extended Info
288	Digestive	BSS01654	STOMACH
289	Digestive	BSS01651	STOMACH
290	Digestive	BSS01639	STOMACH
291	Digestive	BSS01284	STOMACH MUCOSA
292	Digestive	BSS01848	TRANSVERSE COLON
293	Digestive	BSS01851	TRANSVERSE COLON
294	Digestive	BSS01849	TRANSVERSE COLON
295	Digestive	BSS01850	TRANSVERSE COLON
296	Endocrine	BSS00282	ENDOCRINE PANCREAS
297	Endocrine	BSS00283	ENDOCRINE PANCREAS
298	Endocrine	BSS00284	ENDOCRINE PANCREAS
299	Endocrine	BSS00281	ENDOCRINE PANCREAS
300	Endocrine	BSS01403	OVARY
301	Endocrine	BSS01401	OVARY
302	Endocrine	BSS01402	OVARY
303	Endocrine	BSS01399	OVARY
304	Endocrine	BSS01719	TESTIS
305	Endocrine	BSS01718	TESTIS
306	Endocrine	BSS01715	TESTIS
307	Endocrine	BSS00052	ADRENAL GLAND
308	Endocrine	BSS00050	ADRENAL GLAND
309	Endocrine	BSS00051	ADRENAL GLAND
310	Endocrine	BSS00059	ADRENAL GLAND
311	Endocrine	BSS00057	ADRENAL GLAND
312	Endocrine	BSS00058	ADRENAL GLAND
313	Endocrine	BSS00045	ADRENAL GLAND
314	Endocrine	BSS00046	ADRENAL GLAND
315	Endocrine	BSS00047	ADRENAL GLAND
316	Endocrine	BSS00048	ADRENAL GLAND
317	Endocrine	BSS00054	ADRENAL GLAND
318	Endocrine	BSS00060	ADRENAL GLAND
319	Endocrine	BSS00055	ADRENAL GLAND
320	Endocrine	BSS00056	ADRENAL GLAND
321	Endocrine	BSS01831	THYROID GLAND
322	Endocrine	BSS01834	THYROID GLAND
323	Endocrine	BSS01832	THYROID GLAND
324	Endocrine	BSS01835	THYROID GLAND
325	Endothelial	BSS00143	BRAIN MICROVASCULAR ENDOTHELIAL CELL
326	Endothelial	BSS00387	GLOMERULUS ENDOTHELIAL CELL
327	Endothelial	BSS01077	KIDNEY CAPILLARY ENDOTHELIAL CELL
328	Endothelial	BSS01206	LUNG MICROVASCULAR ENDOTHELIAL CELL
329	Endothelial	BSS01465	PULMONARY ARTERY ENDOTHELIAL CELL
330	Endothelial	BSS00298	UMBILICAL VEIN ENDOTHELIAL CELL
331	Endothelial	BSS00296	UMBILICAL VEIN ENDOTHELIAL CELL
332	Endothelial	BSS00258	DERMIS BLOOD VESSEL ENDOTHELIAL CELL
333	Endothelial	BSS00260	DERMIS BLOOD VESSEL ENDOTHELIAL CELL
334	Endothelial	BSS00262	DERMIS LYMPHATIC VESSEL ENDOTHELIAL CELL
335	Endothelial	BSS00264	DERMIS LYMPHATIC VESSEL ENDOTHELIAL CELL
336	Epithelial	BSS00704	BONE MARROW EPITHELIAL CELL
337	Epithelial	BSS00218	CHOROID PLEXUS EPITHELIAL CELL
338	Epithelial	BSS00223	COLON EPITHELIAL CELL
339	Epithelial	BSS00307	ESOPHAGUS EPITHELIAL CELL
340	Epithelial	BSS00743	IRIS PIGMENT EPITHELIAL CELL
341	Epithelial	BSS01385	NON-PIGMENTED CILIARY EPITHELIAL CELL
342	Epithelial	BSS01092	GLOMERULUS EPITHELIAL CELL
343	Epithelial	BSS00389	GLOMERULUS VISCERAL EPITHELIAL CELL
344	Epithelial	BSS01080	KIDNEY EPITHELIAL CELL
345	Epithelial	BSS00310	PROXIMAL TUBULE EPITHELIAL CELL
346	Epithelial	BSS00701	PROXIMAL TUBULE EPITHELIAL CELL
347	Epithelial	BSS01491	RENAL CORTICAL EPITHELIAL CELL
348	Epithelial	BSS01505	RETINAL EPITHELIAL CELL
349	Epithelial	BSS01103	TUBULE CELL
350	Epithelial	BSS01102	TUBULE CELL
351	Epithelial	BSS00153	BRONCHIAL EPITHELIAL CELL
352	Epithelial	BSS00150	BRONCHIAL EPITHELIAL CELL
353	Epithelial	BSS00703	PANCREATIC DUCT EPITHELIAL CELL
354	Epithelial	BSS00075	AMNION EPITHELIAL CELL
355	Epithelial	BSS00308	PROSTATE EPITHELIAL CELL
356	Epithelial	BSS00309	PROSTATE EPITHELIAL CELL
357	Epithelial	BSS01539	PROSTATE EPITHELIAL CELL
358	Epithelial	BSS01538	PROSTATE EPITHELIAL CELL
359	Epithelial	BSS01217	BREAST EPITHELIAL CELL
360	Epithelial	BSS01224	BREAST EPITHELIAL CELL
361	Epithelial	BSS01225	BREAST EPITHELIAL CELL
362	Epithelial	BSS00356	FORESKIN KERATINOCYTE
363	Epithelial	BSS00357	FORESKIN KERATINOCYTE
364	Epithelial	BSS00358	FORESKIN KERATINOCYTE
365	Epithelial	BSS00359	FORESKIN KERATINOCYTE
366	Epithelial	BSS00360	FORESKIN KERATINOCYTE
367	Epithelial	BSS00362	FORESKIN KERATINOCYTE
368	Epithelial	BSS00363	FORESKIN KERATINOCYTE
369	Epithelial	BSS00364	FORESKIN KERATINOCYTE
370	Epithelial	BSS00365	FORESKIN KERATINOCYTE
371	Epithelial	BSS00366	FORESKIN KERATINOCYTE
372	Epithelial	BSS00355	FORESKIN KERATINOCYTE

#	Group	BSSID	Extended Info
575	Kidney	BSS01533	RENAL PELVIS
576	Kidney	BSS01497	RENAL PELVIS
577	Kidney	BSS01498	RENAL PELVIS
578	Kidney	BSS01153	RENAL PELVIS
579	Kidney	BSS01534	RENAL PELVIS
580	Kidney	BSS01499	RENAL PELVIS
581	Liver	BSS00553	HEPATIC STELLATE CELL
582	Liver	BSS00554	HEPATOCYTE
583	Liver	BSS00511	LIVER
584	Liver	BSS01164	LIVER
585	Liver	BSS01170	LIVER
586	Liver	BSS01169	LIVER
587	Liver	BSS01159	LIVER
588	Liver	BSS01168	LIVER
589	Liver	BSS01519	LIVER
590	Liver	BSS01158	LIVER
591	Lung	BSS01195	LUNG
592	Lung	BSS01142	LUNG
593	Lung	BSS01525	LUNG
594	Lung	BSS01143	LUNG
595	Lung	BSS01526	LUNG
596	Lung	BSS01137	LUNG
597	Lung	BSS01520	LUNG
598	Lung	BSS01138	LUNG
599	Lung	BSS01521	LUNG
600	Lung	BSS01139	LUNG
601	Lung	BSS01192	LUNG
602	Lung	BSS01522	LUNG
603	Lung	BSS01140	LUNG
604	Lung	BSS01523	LUNG
605	Lung	BSS01141	LUNG
606	Lung	BSS01524	LUNG
607	Lung	BSS01205	LUNG
608	Lung	BSS01147	LUNG
609	Lung	BSS01529	LUNG
610	Lung	BSS01148	LUNG
611	Lung	BSS01149	LUNG
612	Lung	BSS01530	LUNG
613	Lung	BSS01202	LUNG
614	Lung	BSS01144	LUNG
615	Lung	BSS01527	LUNG
616	Lung	BSS01203	LUNG
617	Lung	BSS01145	LUNG
618	Lung	BSS01146	LUNG
619	Lung	BSS01528	LUNG
620	Lung	BSS01204	LUNG
621	Lung	BSS01189	LUNG
622	Lung	BSS01188	LUNG
623	Lung	BSS01187	LUNG
624	Lung	BSS01198	LUNG
625	Lung	BSS01186	LUNG
626	Lung	BSS01869	LUNG
627	Lung	BSS01196	LUNG
628	Lung	BSS01197	LUNG
629	Lung	BSS01871	LUNG
630	Lung	BSS01201	LUNG
631	Lung	BSS01190	LUNG
632	Lung	BSS01870	LUNG
633	Lung	BSS01193	LUNG
634	Lymphoblastoid	BSS00403	LYMPHOBLASTOID CELL LINE
635	Lymphoblastoid	BSS00462	LYMPHOBLASTOID CELL LINE
636	Lymphoblastoid	BSS00456	LYMPHOBLASTOID CELL LINE
637	Lymphoblastoid	BSS00457	LYMPHOBLASTOID CELL LINE
638	Lymphoblastoid	BSS00438	LYMPHOBLASTOID CELL LINE
639	Lymphoblastoid	BSS00473	LYMPHOBLASTOID CELL LINE
640	Lymphoblastoid	BSS00471	LYMPHOBLASTOID CELL LINE
641	Lymphoblastoid	BSS00474	LYMPHOBLASTOID CELL LINE
642	Lymphoblastoid	BSS00404	LYMPHOBLASTOID CELL LINE
643	Lymphoblastoid	BSS00405	LYMPHOBLASTOID CELL LINE
644	Lymphoblastoid	BSS00472	LYMPHOBLASTOID CELL LINE
645	Lymphoblastoid	BSS00428	LYMPHOBLASTOID CELL LINE
646	Lymphoblastoid	BSS00454	LYMPHOBLASTOID CELL LINE
647	Lymphoblastoid	BSS00427	LYMPHOBLASTOID CELL LINE
648	Lymphoblastoid	BSS00452	LYMPHOBLASTOID CELL LINE
649	Lymphoblastoid	BSS00395	LYMPHOBLASTOID CELL LINE
650	Lymphoblastoid	BSS00439	LYMPHOBLASTOID CELL LINE
651	Mesench	BSS00039	ADIPOCYTE FROM MSC
652	Mesench	BSS00250	AMNIOTIC FLUID FROM MSC
653	Mesench	BSS00279	EMBRYONIC FACIAL PROMINENCE
654	Mesench	BSS01260	MESENCHYMAL STEM CELL
655	Muscle	BSS01293	ARM MUSCLE
656	Muscle	BSS01294	ARM MUSCLE
657	Muscle	BSS01290	ARM MUSCLE
658	Muscle	BSS00352	ARM MUSCLE
659	Muscle	BSS01291	ARM MUSCLE

Figure S1: Sample list with tissue group, unique identifier, and short name for 859 observed/imputed samples (full metadata in Table S1, page 1 of 4).

	Group	BSSID	Extended Info
86	Brain	BSS00206	CEREBELLUM
87	Brain	BSS00219	CINGULATE GYRUS
88	Brain	BSS00220	CINGULATE GYRUS
89	Brain	BSS00369	FRONTAL CORTEX
90	Brain	BSS00371	FRONTAL CORTEX
91	Brain	BSS00385	GERMINAL MATRIX
92	Brain	BSS00386	GLOBUS PALLIDUS
93	Brain	BSS01125	HIPPOCAMPUS
94	Brain	BSS01126	HIPPOCAMPUS
95	Brain	BSS01124	HIPPOCAMPUS
96	Brain	BSS00729	INFERIOR PARIETAL CORTEX
97	Brain	BSS01250	MEDULLA OBLONGATA
98	Brain	BSS01270	MIDBRAIN
99	Brain	BSS01271	MIDDLE FRONTAL AREA
100	Brain	BSS01272	MIDDLE FRONTAL AREA
101	Brain	BSS01273	MIDDLE FRONTAL GYRUS
102	Brain	BSS01388	OCCIPITAL LOBE
103	Brain	BSS01451	PONS
104	Brain	BSS01452	POSTERIOR CINGULATE CORTEX
105	Brain	BSS01469	PUTAMEN
106	Brain	BSS01675	SUBSTANTIA NIGRA
107	Brain	BSS01676	SUBSTANTIA NIGRA
108	Brain	BSS01677	SUPERIOR TEMPORAL GYRUS
109	Brain	BSS01714	TEMPORAL LOBE
110	Brain	BSS01712	TEMPORAL LOBE
111	Cancer	BSS01105	ACUTE LYMPHOBLASTIC LEUKEMIA
112	Cancer	BSS00267	ACUTE LYMPHOBLASTIC LEUKEMIA
113	Cancer	BSS01178	ACUTE LYMPHOBLASTIC LEUKEMIA
114	Cancer	BSS01267	OSTEOSARCOMA
115	Cancer	BSS01550	OSTEOSARCOMA
116	Cancer	BSS00246	DESMOPLASTIC MEDULLOBLASTOMA
117	Cancer	BSS01208	GLIOBLASTOMA
118	Cancer	BSS00004	GLIOBLASTOMA
119	Cancer	BSS00482	GLIOBLASTOMA
120	Cancer	BSS01251	MEDULLOBLASTOMA
121	Cancer	BSS01554	NEUROBLASTOMA
122	Cancer	BSS01558	NEUROBLASTOMA
123	Cancer	BSS00102	NEUROBLASTOMA
124	Cancer	BSS01571	NEUROBLASTOMA
125	Cancer	BSS01562	NEUROBLASTOMA
126	Cancer	BSS01559	NEUROEPITHELIOMA
127	Cancer	BSS00481	NEUROGLIOMA
128	Cancer	BSS01535	COLON CARCINOMA
129	Cancer	BSS01682	COLORECTAL ADENOCARCINOMA
130	Cancer	BSS00708	COLORECTAL ADENOCARCINOMA
131	Cancer	BSS01179	COLORECTAL ADENOCARCINOMA
132	Cancer	BSS00159	COLORECTAL ADENOCARCINOMA
133	Cancer	BSS00492	COLORECTAL ADENOCARCINOMA
134	Cancer	BSS01412	PARATHYROID ADENOMA
135	Cancer	BSS01411	PARATHYROID ADENOMA
136	Cancer	BSS01386	TESTICULAR EMBRYONAL CARCINOMA
137	Cancer	BSS01536	MELANOMA
138	Cancer	BSS01551	MELANOMA
139	Cancer	BSS00222	MELANOMA
140	Cancer	BSS01365	MYELOMA
141	Cancer	BSS01890	EYE RETINOBLASTOMA
142	Cancer	BSS00702	ACUTE PROMYELOCYTIC LEUKEMIA
143	Cancer	BSS01356	ACUTE PROMYELOCYTIC LEUKEMIA
144	Cancer	BSS01391	B CELL LYMPHOMA
145	Cancer	BSS01390	B CELL LYMPHOMA
146	Cancer	BSS01065	B CELL LYMPHOMA
147	Cancer	BSS00268	B CELL LYMPHOMA
148	Cancer	BSS01664	B CELL LYMPHOMA
149	Cancer	BSS01389	B CELL LYMPHOMA
150	Cancer	BSS01350	BURKITT LYMPHOMA
151	Cancer	BSS01351	BURKITT LYMPHOMA
152	Cancer	BSS00491	HAPLOID MYELOGENOUS LEUKEMIA
153	Cancer	BSS01038	MYELOGENOUS LEUKEMIA
154	Cancer	BSS01039	MYELOGENOUS LEUKEMIA
155	Cancer	BSS01056	MYELOGENOUS LEUKEMIA
156	Cancer	BSS01057	MYELOGENOUS LEUKEMIA
157	Cancer	BSS01059	MYELOGENOUS LEUKEMIA
158	Cancer	BSS00221	MYELOGENOUS LEUKEMIA
159	Cancer	BSS01066	MYELOGENOUS LEUKEMIA
160	Cancer	BSS00762	MYELOGENOUS LEUKEMIA
161	Cancer	BSS01104	MYELOMA
162	Cancer	BSS01274	MYELOMA
163	Cancer	BSS01537	PLASMA CELL MYELOMA
164	Cancer	BSS00160	KIDNEY CLEAR CELL CARCINOMA
165	Cancer	BSS00372	KIDNEY RHABOID TUMOR
166	Cancer	BSS00037	RENAL CELL ADENOCARCINOMA
167	Cancer	BSS01474	RENAL CELL ADENOCARCINOMA
168	Cancer	BSS01481	RENAL CELL CARCINOMA
169	Cancer	BSS00718	HEPATOCELLULAR CARCINOMA

	Group	BSSID	Extended Info
373	Epithelial	BSS00361	FORESKIN KERATINOCYTE
374	Epithelial	BSS00367	FORESKIN KERATINOCYTE
375	Epithelial	BSS00354	FORESKIN KERATINOCYTE
376	Epithelial	BSS01071	KERATINOCYTE
377	Epithelial	BSS01068	KERATINOCYTE
378	Epithelial	BSS01209	MAMMARY EPITHELIAL CELL
379	Epithelial	BSS01211	MAMMARY EPITHELIAL CELL
380	Epithelial	BSS01213	MAMMARY EPITHELIAL CELL
381	Epithelial	BSS01185	MAMMARY LUMINAL EPITHELIAL CELL
382	Epithelial	BSS01340	MAMMARY MYOEPITHELIAL CELL
383	Epithelial	BSS01341	MAMMARY MYOEPITHELIAL CELL
384	Epithelial	BSS01181	SKIN LEG
385	Epithelial	BSS01182	SKIN LEG
386	Epithelial	BSS01587	SKIN OF BODY
387	ES-deriv	BSS00112	BIPOLAR NEURON DERIV
388	ES-deriv	BSS01366	NEURAL DERIV
389	ES-deriv	BSS00272	NEURAL PROGENITOR DERIV
390	ES-deriv	BSS01372	NEURAL PROGENITOR DERIV
391	ES-deriv	BSS01370	NEURAL PROGENITOR DERIV
392	ES-deriv	BSS01371	NEURAL PROGENITOR DERIV
393	ES-deriv	BSS01375	NEURON DERIV
394	ES-deriv	BSS00169	CARDIAC MESODERM DERIV
395	ES-deriv	BSS00171	CARDIAC MUSCLE DERIV
396	ES-deriv	BSS00556	HEPATOCYTE DERIV
397	ES-deriv	BSS01261	MESENCHYMAL STEM DERIV
398	ES-deriv	BSS01857	TROPHOBLAST DERIV
399	ES-deriv	BSS01612	SMOOTH MUSCLE DERIV
400	ES-deriv	BSS00273	ECTODERMAL DERIV
401	ES-deriv	BSS00285	ENDODERMAL CELL
402	ES-deriv	BSS00287	ENDODERMAL DERIV
403	ES-deriv	BSS01263	MESENDODERM DERIV
404	ES-deriv	BSS01264	MESODERMAL DERIV
405	ESC	BSS00277	ESC
406	ESC	BSS00315	ESC
407	ESC	BSS01866	ESC
408	ESC	BSS00483	ESC
409	ESC	BSS00715	ESC
410	ESC	BSS00716	ESC
411	ESC	BSS00717	ESC
412	ESC	BSS00484	ESC
413	ESC	BSS00478	ESC
414	Eye	BSS00329	EYE
415	Eye	BSS00328	EYE
416	Eye	BSS01504	EYE RETINA
417	Eye	BSS01503	EYE RETINA
418	Eye	BSS01502	EYE RETINA
419	Heart	BSS00079	AORTA
420	Heart	BSS00080	AORTA
421	Heart	BSS00088	ASCENDING AORTA
422	Heart	BSS00087	ASCENDING AORTA
423	Heart	BSS00242	CORONARY ARTERY
424	Heart	BSS00243	CORONARY ARTERY
425	Heart	BSS00505	HEART
426	Heart	BSS00498	HEART
427	Heart	BSS00499	HEART
428	Heart	BSS00500	HEART
429	Heart	BSS00502	HEART
430	Heart	BSS00503	HEART
431	Heart	BSS00501	HEART
432	Heart	BSS00516	HEART
433	Heart	BSS00522	HEART
434	Heart	BSS00518	HEART
435	Heart	BSS00519	HEART
436	Heart	BSS00514	HEART
437	Heart	BSS00520	HEART
438	Heart	BSS00495	HEART
439	Heart	BSS00496	HEART
440	Heart	BSS00494	HEART
441	Heart	BSS00521	HEART
442	Heart	BSS00517	HEART
443	Heart	BSS00493	HEART
444	Heart	BSS01127	HEART LEFT ATRIUM
445	Heart	BSS00509	HEART LEFT VENTRICLE
446	Heart	BSS00508	HEART LEFT VENTRICLE
447	Heart	BSS00506	HEART LEFT VENTRICLE
448	Heart	BSS00513	HEART LEFT VENTRICLE
449	Heart	BSS00512	HEART LEFT VENTRICLE
450	Heart	BSS00507	HEART LEFT VENTRICLE
451	Heart	BSS01506	HEART RIGHT ATRIUM
452	Heart	BSS01508	HEART RIGHT ATRIUM
453	Heart	BSS01507	HEART RIGHT ATRIUM
454	Heart	BSS00523	HEART RIGHT VENTRICLE
455	Heart	BSS00524	HEART RIGHT VENTRICLE
456	Heart	BSS00525	HEART RIGHT VENTRICLE

	Group	BSSID	Extended Info
660	Muscle	BSS01292	ARM MUSCLE
661	Muscle	BSS01303	ARM MUSCLE
662	Muscle	BSS01304	ARM MUSCLE
663	Muscle	BSS01295	ARM MUSCLE
664	Muscle	BSS01296	ARM MUSCLE
665	Muscle	BSS01297	ARM MUSCLE
666	Muscle	BSS01298	ARM MUSCLE
667	Muscle	BSS01299	ARM MUSCLE
668	Muscle	BSS01300	ARM MUSCLE
669	Muscle	BSS01301	ARM MUSCLE
670	Muscle	BSS01289	ARM MUSCLE
671	Muscle	BSS01308	BACK MUSCLE
672	Muscle	BSS01309	BACK MUSCLE
673	Muscle	BSS01305	BACK MUSCLE
674	Muscle	BSS01306	BACK MUSCLE
675	Muscle	BSS01307	BACK MUSCLE
676	Muscle	BSS01315	BACK MUSCLE
677	Muscle	BSS01316	BACK MUSCLE
678	Muscle	BSS01317	BACK MUSCLE
679	Muscle	BSS01310	BACK MUSCLE
680	Muscle	BSS01311	BACK MUSCLE
681	Muscle	BSS01312	BACK MUSCLE
682	Muscle	BSS01313	BACK MUSCLE
683	Muscle	BSS01314	BACK MUSCLE
684	Muscle	BSS00170	CARDIAC MYOCYTE
685	Muscle	BSS00376	GASTROCNEMIUS MEDIALIS
686	Muscle	BSS00378	GASTROCNEMIUS MEDIALIS
687	Muscle	BSS00377	GASTROCNEMIUS MEDIALIS
688	Muscle	BSS00379	GASTROCNEMIUS MEDIALIS
689	Muscle	BSS01322	LEG MUSCLE
690	Muscle	BSS01318	LEG MUSCLE
691	Muscle	BSS01320	LEG MUSCLE
692	Muscle	BSS01321	LEG MUSCLE
693	Muscle	BSS01329	LEG MUSCLE
694	Muscle	BSS01330	LEG MUSCLE
695	Muscle	BSS01323	LEG MUSCLE
696	Muscle	BSS01324	LEG MUSCLE
697	Muscle	BSS01325	LEG MUSCLE
698	Muscle	BSS01327	LEG MUSCLE
699	Muscle	BSS00700	LEG MUSCLE
700	Muscle	BSS01328	LEG MUSCLE
701	Muscle	BSS01319	LEG MUSCLE
702	Muscle	BSS01460	PSOAS MUSCLE
703	Muscle	BSS01461	PSOAS MUSCLE
704	Muscle	BSS01462	PSOAS MUSCLE
705	Muscle	BSS01463	PSOAS MUSCLE
706	Muscle	BSS01581	SKELETAL MUSCLE
707	Muscle	BSS01577	SKELETAL MUSCLE
708	Muscle	BSS01578	SKELETAL MUSCLE
709	Muscle	BSS01572	SKELETAL MUSCLE CELL
710	Muscle	BSS01845	TONGUE
711	Muscle	BSS01846	TONGUE
712	Muscle	BSS01331	TRUNK MUSCLE
713	Muscle	BSS01333	TRUNK MUSCLE
714	Muscle	BSS01334	TRUNK MUSCLE
715	Muscle	BSS01332	TRUNK MUSCLE
716	Myosat	BSS01338	MYOCYTE
717	Myosat	BSS01344	MYOTUBE
718	Myosat	BSS01155	SKELETAL MUSCLE MYOBLAST
719	Myosat	BSS01573	SKELETAL MUSCLE MYOBLAST
720	Myosat	BSS01574	SKELETAL MUSCLE MYOBLAST
721	Myosat	BSS01576	SKELETAL MUSCLE SATELLITE CELL
722	Neurosph	BSS01378	NEUROSPHERE
723	Neurosph	BSS01379	NEUROSPHERE
724	Neurosph	BSS01377	NEUROSPHERE
725	Neurosph	BSS01392	OLFACTORY NEUROSPHERE
726	Other	BSS00148	BREAST EPITHELIUM
727	Other	BSS00145	BREAST EPITHELIUM
728	Other	BSS00146	BREAST EPITHELIUM
729	Other	BSS00304	EPIDERMAL MELANOCYTE
730	Other	BSS00368	FORESKIN MELANOCYTE
731	Other	BSS01156	LIMB EMBRYO
732	Other	BSS01157	LIMB EMBRYO
733	Other	BSS01216	MAMMARY STEM CELL
734	Pancreas	BSS00121	BODY OF PANCREAS
735	Pancreas	BSS00122	BODY OF PANCREAS
736	Pancreas	BSS00123	BODY OF PANCREAS
737	Pancreas	BSS00124	BODY OF PANCREAS
738	Pancreas	BSS00758	ISLET PRECURSOR CELL
739	Pancreas	BSS01406	PANCREAS
740	Pancreas	BSS01407	PANCREAS
741	Placenta & EEM	BSS00074	AMNION
742	Placenta & EEM	BSS00076	AMNION STEM CELL
743	Placenta & EEM	BSS00209	CHORION

Figure S1: (continued, 2 of 4)

	Group	BSSID	Extended Info		Group	BSSID	Extended Info		Group	BSSID	Extended Info
170	Cancer	BSS00719	HEPATOCELLULAR CARCINOMA	457	Heart	BSS01815	THORACIC AORTA	744	Placenta & EEM	BSS00211	CHORION
171	Cancer	BSS00558	HEPATOCELLULAR CARCINOMA	458	Heart	BSS01814	THORACIC AORTA	745	Placenta & EEM	BSS00212	CHORION
172	Cancer	BSS01360	LARGE CELL LUNG CANCER	459	Heart	BSS01839	TIBIAL ARTERY	746	Placenta & EEM	BSS00215	CHORIONIC VILLUS
173	Cancer	BSS01415	LUNG ADENOCARCINOMA	460	Heart	BSS01838	TIBIAL ARTERY	747	Placenta & EEM	BSS00216	CHORIONIC VILLUS
174	Cancer	BSS00017	LUNG EPITHELIAL CARCINOMA	461	Heart	BSS01837	TIBIAL ARTERY	748	Placenta & EEM	BSS00217	CHORIONIC VILLUS
175	Cancer	BSS00019	LUNG EPITHELIAL CARCINOMA	462	HSC & B-cell	BSS00097	B CELL	749	Placenta & EEM	BSS00214	CHORIONIC VILLUS
176	Cancer	BSS00021	LUNG EPITHELIAL CARCINOMA	463	HSC & B-cell	BSS01345	B CELL	750	Placenta & EEM	BSS01440	PLACENTA
177	Cancer	BSS00022	LUNG EPITHELIAL CARCINOMA	464	HSC & B-cell	BSS00098	B CELL	751	Placenta & EEM	BSS01436	PLACENTA
178	Cancer	BSS00027	LUNG EPITHELIAL CARCINOMA	465	HSC & B-cell	BSS00093	B CELL	752	Placenta & EEM	BSS01437	PLACENTA
179	Cancer	BSS00016	LUNG EPITHELIAL CARCINOMA	466	HSC & B-cell	BSS00096	B CELL	753	Placenta & EEM	BSS01435	PLACENTA
180	Cancer	BSS00020	LUNG EPITHELIAL CARCINOMA	467	HSC & B-cell	BSS00100	B CELL	754	Placenta & EEM	BSS01443	PLACENTA
181	Cancer	BSS00023	LUNG EPITHELIAL CARCINOMA	468	HSC & B-cell	BSS00101	B CELL	755	Placenta & EEM	BSS01444	PLACENTA
182	Cancer	BSS00024	LUNG EPITHELIAL CARCINOMA	469	HSC & B-cell	BSS00095	B CELL	756	Placenta & EEM	BSS01433	PLACENTA
183	Cancer	BSS00026	LUNG EPITHELIAL CARCINOMA	470	HSC & B-cell	BSS00179	CD14 MONOCYTE	757	Placenta & EEM	BSS01432	PLACENTA
184	Cancer	BSS00028	LUNG EPITHELIAL CARCINOMA	471	HSC & B-cell	BSS00181	CD14 MONOCYTE	758	Placenta & EEM	BSS01430	PLACENTA
185	Cancer	BSS00029	LUNG EPITHELIAL CARCINOMA	472	HSC & B-cell	BSS00180	CD14 MONOCYTE	759	Placenta & EEM	BSS01448	PLACENTA
186	Cancer	BSS00018	LUNG EPITHELIAL CARCINOMA	473	HSC & B-cell	BSS00178	CD14 MONOCYTE	760	Placenta & EEM	BSS01446	PLACENTA
187	Cancer	BSS00025	LUNG EPITHELIAL CARCINOMA	474	HSC & B-cell	BSS00182	CD1C MYELOID DENDRITIC CELL	761	Placenta & EEM	BSS01441	PLACENTA
188	Cancer	BSS00030	LUNG EPITHELIAL CARCINOMA	475	HSC & B-cell	BSS00233	CD34 CMP	762	Placenta & EEM	BSS01431	PLACENTA
189	Cancer	BSS00007	LUNG EPITHELIAL CARCINOMA	476	HSC & B-cell	BSS00230	CD34 CMP	763	Placenta & EEM	BSS01438	PLACENTA
190	Cancer	BSS00013	LUNG EPITHELIAL CARCINOMA	477	HSC & B-cell	BSS00236	CD34 CMP	764	Placenta & EEM	BSS00714	TROPHOBLAST
191	Cancer	BSS00015	LUNG EPITHELIAL CARCINOMA	478	HSC & B-cell	BSS00238	CD34 CMP	765	Placenta & EEM	BSS01856	TROPHOBLAST
192	Cancer	BSS01359	SQUAMOUS CELL CARCINOMA	479	HSC & B-cell	BSS00240	CD34 CMP	766	Placenta & EEM	BSS01853	TROPHOBLAST
193	Cancer	BSS00035	MUSCLE EWING SARCOMA	480	HSC & B-cell	BSS00241	CD34 CMP	767	Placenta & EEM	BSS01855	TROPHOBLAST
194	Cancer	BSS01549	RHABDOMYOSARCOMA	481	HSC & B-cell	BSS00234	CD34 CMP	768	Placenta & EEM	BSS01852	TROPHOBLAST
195	Cancer	BSS00036	ADENOID CYSTIC CARCINOMA	482	HSC & B-cell	BSS00235	CD34 CMP	769	Placenta & EEM	BSS01859	TROPHOBLAST
196	Cancer	BSS01240	MAMMARY GLAND ADENOCARCINOMA	483	HSC & B-cell	BSS00229	CD34 CMP	770	Placenta & EEM	BSS01860	TROPHOBLAST
197	Cancer	BSS01243	MAMMARY GLAND ADENOCARCINOMA	484	HSC & B-cell	BSS00237	CD34 CMP	771	Placenta & EEM	BSS01867	UMBILICAL CORD
198	Cancer	BSS01244	MAMMARY GLAND ADENOCARCINOMA	485	HSC & B-cell	BSS00239	CD34 CMP	772	PNS	BSS01618	SPINAL CORD
199	Cancer	BSS01235	MAMMARY GLAND ADENOCARCINOMA	486	HSC & B-cell	BSS00231	CD34 CMP	773	PNS	BSS01619	SPINAL CORD
200	Cancer	BSS01226	MAMMARY GLAND ADENOCARCINOMA	487	HSC & B-cell	BSS00232	CD34 CMP	774	PNS	BSS01617	SPINAL CORD
201	Cancer	BSS01699	MAMMARY GLAND DUCTAL CARCINOMA	488	HSC & B-cell	BSS00384	GERMINAL CENTER	775	PNS	BSS01621	SPINAL CORD
202	Cancer	BSS01705	MAMMARY GLAND DUCTAL CARCINOMA	489	HSC & B-cell	BSS00760	LYMPHOCYTE	776	PNS	BSS01620	SPINAL CORD
203	Cancer	BSS00003	PANCREAS ADENOCARCINOMA	490	HSC & B-cell	BSS00544	MPP	777	PNS	BSS01614	SPINAL CORD
204	Cancer	BSS01405	PANCREAS DUCT EPITHELIAL CARCINOMA	491	HSC & B-cell	BSS00545	MPP	778	PNS	BSS01613	SPINAL CORD
205	Cancer	BSS00541	CERVIX ADENOCARCINOMA	492	HSC & B-cell	BSS00546	MPP	779	PNS	BSS01842	TIBIAL NERVE
206	Cancer	BSS00531	CERVIX ADENOCARCINOMA	493	HSC & B-cell	BSS00547	MPP	780	PNS	BSS01840	TIBIAL NERVE
207	Cancer	BSS00529	CERVIX ADENOCARCINOMA	494	HSC & B-cell	BSS00548	MPP	781	PNS	BSS01841	TIBIAL NERVE
208	Cancer	BSS00748	ENDOMETRIAL ADENOCARCINOMA	495	HSC & B-cell	BSS00549	MPP	782	Reproductive	BSS01456	PROSTATE GLAND
209	Cancer	BSS00756	ENDOMETRIAL ADENOCARCINOMA	496	HSC & B-cell	BSS00550	MPP	783	Reproductive	BSS01457	PROSTATE GLAND
210	Cancer	BSS00745	ENDOMETRIAL ADENOCARCINOMA	497	HSC & B-cell	BSS00551	MPP	784	Reproductive	BSS01459	PROSTATE GLAND
211	Cancer	BSS01174	PROSTATE ADENOCARCINOMA	498	HSC & B-cell	BSS00552	MPP	785	Reproductive	BSS01884	UTERUS
212	Cancer	BSS01173	PROSTATE ADENOCARCINOMA	499	HSC & B-cell	BSS00543	MPP	786	Reproductive	BSS01886	VAGINA
213	Cancer	BSS01414	PROSTATE ADENOCARCINOMA	500	HSC & B-cell	BSS01381	NEUTROPHIL	787	Reproductive	BSS01887	VAGINA
214	Cancer	BSS00157	PROSTATE CANCER	501	HSC & B-cell	BSS01380	NEUTROPHIL	788	Sm. Muscle	BSS01606	BRAIN VASCULATURE SMOOTH MUSCLE CELL
215	Cancer	BSS01888	PROSTATE EPITHELIAL CARCINOMA	502	HSC & B-cell	BSS01353	NK CELL	789	Sm. Muscle	BSS01285	COLON MUSCLE
216	Cancer	BSS00001	PROSTATE EPITHELIAL CARCINOMA	503	HSC & B-cell	BSS01355	NK CELL	790	Sm. Muscle	BSS01286	COLON MUSCLE
217	Cancer	BSS00002	PROSTATE EPITHELIAL CARCINOMA	504	HSC & B-cell	BSS01354	NK CELL	791	Sm. Muscle	BSS01288	DUODENUM MUSCLE
218	Cancer	BSS00709	FIBROSARCOMA	505	iPSC	BSS00742	iPSC	792	Sm. Muscle	BSS01287	DUODENUM MUSCLE
219	Digestive	BSS00227	COLON MUCOSA	506	iPSC	BSS00735	iPSC	793	Sm. Muscle	BSS01475	RECTUM MUSCLE
220	Digestive	BSS00228	COLON MUCOSA	507	iPSC	BSS00741	iPSC	794	Sm. Muscle	BSS01660	STOMACH MUSCLE
221	Digestive	BSS00271	DUODENUM MUCOSA	508	iPSC	BSS00732	iPSC	795	Sm. Muscle	BSS01659	STOMACH MUSCLE
222	Digestive	BSS00270	DUODENUM MUCOSA	509	iPSC	BSS00733	iPSC	796	Spleen	BSS01625	SPLEEN
223	Digestive	BSS00316	ESOPHAGUS	510	iPSC	BSS00244	iPSC	797	Spleen	BSS01628	SPLEEN
224	Digestive	BSS00318	ESOPHAGUS	511	iPSC	BSS01107	iPSC	798	Spleen	BSS01629	SPLEEN
225	Digestive	BSS00323	ESOPHAGUS MUSCULARIS MUCOSA	512	iPSC	BSS01108	iPSC	799	Spleen	BSS01633	SPLEEN
226	Digestive	BSS00322	ESOPHAGUS MUSCULARIS MUCOSA	513	iPSC	BSS00738	iPSC	800	Spleen	BSS01634	SPLEEN
227	Digestive	BSS00321	ESOPHAGUS MUSCULARIS MUCOSA	514	iPSC	BSS00736	iPSC	801	Spleen	BSS01631	SPLEEN
228	Digestive	BSS00324	ESOPHAGUS SQUAMOUS EPITHELIUM	515	iPSC	BSS00737	iPSC	802	Spleen	BSS01630	SPLEEN
229	Digestive	BSS00326	ESOPHAGUS SQUAMOUS EPITHELIUM	516	iPSC	BSS00739	iPSC	803	Stromal	BSS01661	BONE MARROW STROMAL CELL
230	Digestive	BSS00325	ESOPHAGUS SQUAMOUS EPITHELIUM	517	iPSC	BSS00731	iPSC	804	Stromal	BSS00144	PERICYTE
231	Digestive	BSS00380	GASTROESOPHAGEAL SPHINCTER	518	iPSC	BSS00734	iPSC	805	Stromal	BSS00349	CONJUNCTIVA FIBROBLAST
232	Digestive	BSS00381	GASTROESOPHAGEAL SPHINCTER	519	iPSC	BSS00477	iPSC	806	Stromal	BSS00347	AORTA FIBROBLAST
233	Digestive	BSS01116	LARGE INTESTINE	520	Kidney	BSS01091	KIDNEY	807	Stromal	BSS00168	CARDIAC FIBROBLAST
234	Digestive	BSS01117	LARGE INTESTINE	521	Kidney	BSS01132	KIDNEY	808	Stromal	BSS00166	CARDIAC FIBROBLAST
235	Digestive	BSS01109	LARGE INTESTINE	522	Kidney	BSS01512	KIDNEY	809	Stromal	BSS00167	CARDIAC FIBROBLAST
236	Digestive	BSS01110	LARGE INTESTINE	523	Kidney	BSS01133	KIDNEY	810	Stromal	BSS00064	LUNG FIBROBLAST
237	Digestive	BSS01111	LARGE INTESTINE	524	Kidney	BSS01513	KIDNEY	811	Stromal	BSS01891	LUNG FIBROBLAST
238	Digestive	BSS01112	LARGE INTESTINE	525	Kidney	BSS01084	KIDNEY	812	Stromal	BSS00342	LUNG FIBROBLAST
239	Digestive	BSS01113	LARGE INTESTINE	526	Kidney	BSS01128	KIDNEY	813	Stromal	BSS00339	LUNG FIBROBLAST
240	Digestive	BSS01114	LARGE INTESTINE	527	Kidney	BSS01509	KIDNEY	814	Stromal	BSS00062	LUNG FIBROBLAST
241	Digestive	BSS01122	LARGE INTESTINE	528	Kidney	BSS01085	KIDNEY	815	Stromal	BSS00341	LUNG FIBROBLAST
242	Digestive	BSS01118	LARGE INTESTINE	529	Kidney	BSS01129	KIDNEY	816	Stromal	BSS00720	LUNG FIBROBLAST
243	Digestive	BSS01120	LARGE INTESTINE	530	Kidney	BSS01086	KIDNEY	817	Stromal	BSS00345	PULMONARY ARTERY FIBROBLAST
244	Digestive	BSS01121	LARGE INTESTINE	531	Kidney	BSS01510	KIDNEY	818	Stromal	BSS00338	GINGIVAL FIBROBLAST
245	Digestive	BSS01119	LARGE INTESTINE	532	Kidney	BSS01089	KIDNEY	819	Stromal	BSS00067	GINGIVAL FIBROBLAST
246	Digestive	BSS01427	PEYERS PATCH	533	Kidney	BSS01130	KIDNEY	820	Stromal	BSS00344	PERIDONTAL LIGAMENT FIBROBLAST
247	Digestive	BSS01428	PEYERS PATCH	534	Kidney	BSS01511	KIDNEY	821	Stromal	BSS00350	VILLOUS MESENCHYME FIBROBLAST
248	Digestive	BSS01426	PEYERS PATCH	535	Kidney	BSS01090	KIDNEY	822	Stromal	BSS00332	BREAST FIBROBLAST
249	Digestive	BSS01282	RECTUM MUCOSA	536	Kidney	BSS01078	KIDNEY	823	Stromal	BSS00333	BREAST FIBROBLAST
250	Digestive	BSS01283	RECTUM MUCOSA	537	Kidney	BSS01100	KIDNEY	824	Stromal	BSS00335	DERMIS FIBROBLAST
251	Digestive	BSS01542	SIGMOID COLON	538	Kidney	BSS01101	KIDNEY	825	Stromal	BSS00334	DERMIS FIBROBLAST
252	Digestive	BSS01546	SIGMOID COLON	539	Kidney	BSS01135	KIDNEY	826	Stromal	BSS00337	DERMIS FIBROBLAST
253	Digestive	BSS01547	SIGMOID COLON	540	Kidney	BSS01516	KIDNEY	827	Stromal	BSS00697	FORESKIN FIBROBLAST

Figure S1: (continued, 3 of 4)

	Group	BSSID	Extended Info
254	Digestive	BSS01548	SIGMOID COLON
255	Digestive	BSS01545	SIGMOID COLON
256	Digestive	BSS01543	SIGMOID COLON
257	Digestive	BSS01595	SMALL INTESTINE
258	Digestive	BSS01596	SMALL INTESTINE
259	Digestive	BSS01590	SMALL INTESTINE
260	Digestive	BSS01591	SMALL INTESTINE
261	Digestive	BSS01592	SMALL INTESTINE
262	Digestive	BSS01593	SMALL INTESTINE
263	Digestive	BSS01594	SMALL INTESTINE
264	Digestive	BSS01603	SMALL INTESTINE
265	Digestive	BSS01604	SMALL INTESTINE
266	Digestive	BSS01600	SMALL INTESTINE
267	Digestive	BSS01602	SMALL INTESTINE
268	Digestive	BSS01597	SMALL INTESTINE
269	Digestive	BSS01599	SMALL INTESTINE
270	Digestive	BSS01588	SMALL INTESTINE
271	Digestive	BSS01601	SMALL INTESTINE
272	Digestive	BSS01637	STOMACH
273	Digestive	BSS01642	STOMACH
274	Digestive	BSS01643	STOMACH
275	Digestive	BSS01644	STOMACH
276	Digestive	BSS01646	STOMACH
277	Digestive	BSS01647	STOMACH
278	Digestive	BSS01641	STOMACH
279	Digestive	BSS01658	STOMACH
280	Digestive	BSS01655	STOMACH
281	Digestive	BSS01656	STOMACH
282	Digestive	BSS01657	STOMACH
283	Digestive	BSS01636	STOMACH
284	Digestive	BSS01638	STOMACH
285	Digestive	BSS01650	STOMACH
286	Digestive	BSS01653	STOMACH
287	Digestive	BSS01649	STOMACH

	Group	BSSID	Extended Info
541	Kidney	BSS01517	KIDNEY
542	Kidney	BSS01136	KIDNEY
543	Kidney	BSS01518	KIDNEY
544	Kidney	BSS01099	KIDNEY
545	Kidney	BSS01514	KIDNEY
546	Kidney	BSS01134	KIDNEY
547	Kidney	BSS01515	KIDNEY
548	Kidney	BSS01131	KIDNEY
549	Kidney	BSS01079	KIDNEY
550	Kidney	BSS01096	KIDNEY
551	Kidney	BSS01097	KIDNEY
552	Kidney	BSS01088	KIDNEY
553	Kidney	BSS00528	KIDNEY CELL
554	Kidney	BSS00526	KIDNEY CELL
555	Kidney	BSS01484	RENAL CORTEX INTERSTITIUM
556	Kidney	BSS01485	RENAL CORTEX INTERSTITIUM
557	Kidney	BSS01482	RENAL CORTEX INTERSTITIUM
558	Kidney	BSS01483	RENAL CORTEX INTERSTITIUM
559	Kidney	BSS01489	RENAL CORTEX INTERSTITIUM
560	Kidney	BSS01490	RENAL CORTEX INTERSTITIUM
561	Kidney	BSS01150	RENAL CORTEX INTERSTITIUM
562	Kidney	BSS01531	RENAL CORTEX INTERSTITIUM
563	Kidney	BSS01486	RENAL CORTEX INTERSTITIUM
564	Kidney	BSS01487	RENAL CORTEX INTERSTITIUM
565	Kidney	BSS01151	RENAL CORTEX INTERSTITIUM
566	Kidney	BSS01532	RENAL CORTEX INTERSTITIUM
567	Kidney	BSS01488	RENAL CORTEX INTERSTITIUM
568	Kidney	BSS01495	RENAL PELVIS
569	Kidney	BSS01496	RENAL PELVIS
570	Kidney	BSS01493	RENAL PELVIS
571	Kidney	BSS01494	RENAL PELVIS
572	Kidney	BSS01500	RENAL PELVIS
573	Kidney	BSS01501	RENAL PELVIS
574	Kidney	BSS01152	RENAL PELVIS

	Group	BSSID	Extended Info
828	Stromal	BSS00353	FORESKIN FIBROBLAST
829	Stromal	BSS00343	MAMMARY FIBROBLAST
830	Stromal	BSS00275	SKIN FIBROBLAST
831	Stromal	BSS00276	SKIN FIBROBLAST
832	Stromal	BSS00393	SKIN FIBROBLAST
833	Stromal	BSS00394	SKIN FIBROBLAST
834	Stromal	BSS00063	SKIN FIBROBLAST
835	Stromal	BSS00069	SKIN FIBROBLAST
836	Stromal	BSS00346	SKIN FIBROBLAST
837	Stromal	BSS01583	SKIN FIBROBLAST
838	Stromal	BSS00278	SKIN FIBROBLAST
839	Stromal	BSS00390	SKIN FIBROBLAST
840	Stromal	BSS00066	SKIN FIBROBLAST
841	Stromal	BSS00061	SKIN FIBROBLAST
842	Stromal	BSS00068	SKIN FIBROBLAST
843	Stromal	BSS00476	SKIN FIBROBLAST
844	Stromal	BSS00113	SKIN FIBROBLAST
845	Thymus	BSS01824	THYMUS
846	Thymus	BSS01819	THYMUS
847	Thymus	BSS01821	THYMUS
848	Thymus	BSS01823	THYMUS
849	Thymus	BSS01818	THYMUS
850	Thymus	BSS01826	THYMUS
851	Thymus	BSS01827	THYMUS
852	Thymus	BSS01828	THYMUS
853	Thymus	BSS01829	THYMUS
854	Thymus	BSS01820	THYMUS
855	Thymus	BSS01825	THYMUS
856	Urinary	BSS01878	URINARY BLADDER
857	Urinary	BSS01876	URINARY BLADDER
858	Urinary	BSS01879	UROTHELIUM CELL
859	Urinary	BSS01880	UROTHELIUM CELL

Figure S1: (continued, 4 of 4)

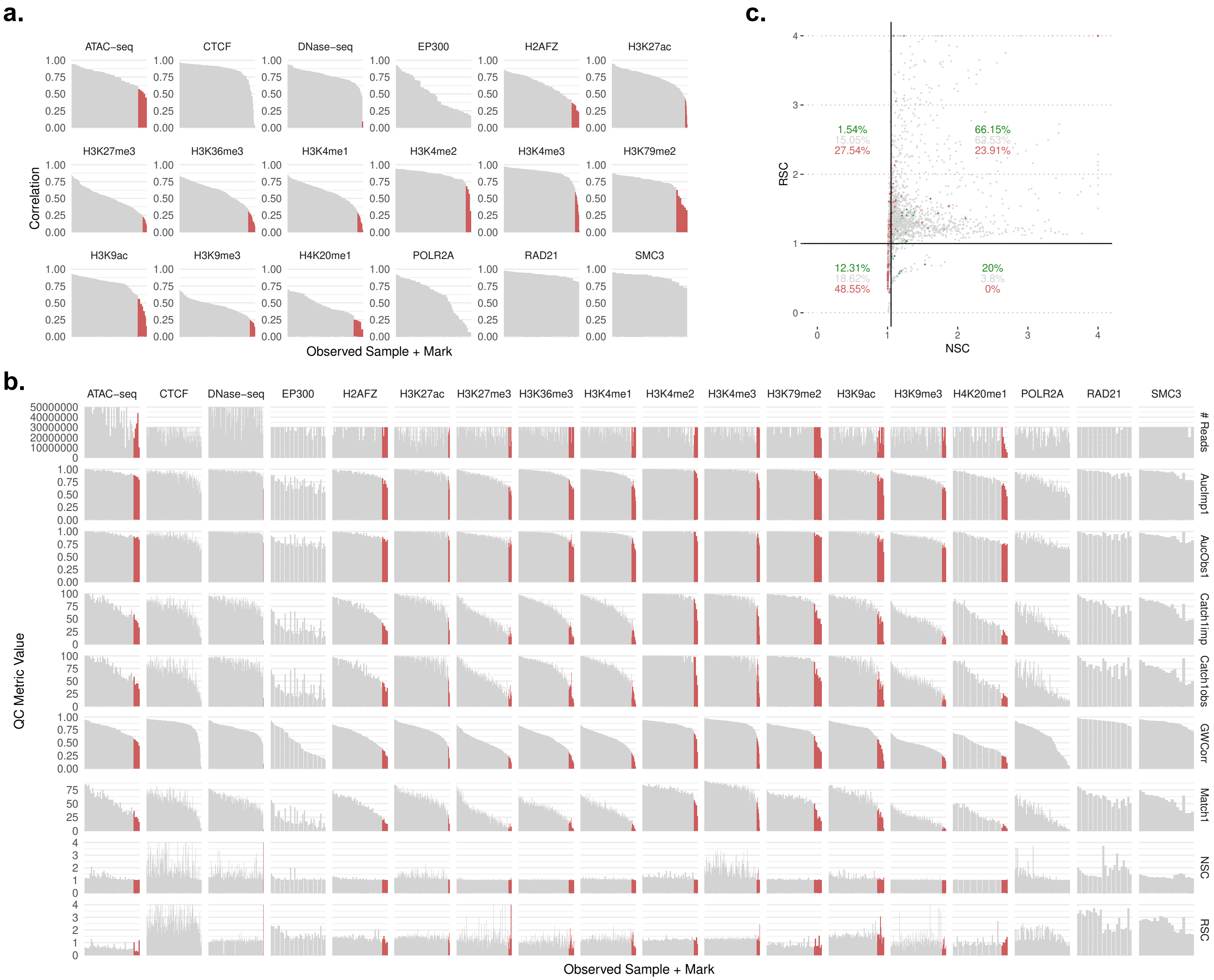

Figure S2: **a.** Track agreement between observed and imputed in the Tier 1 marks, where both are available, by genome wide correlation. Top (green) and bottom (red) tracks are labeled according to automated elbow discovery in the ranked list. **b.** All imputation metrics (from original ChromImpute paper: correlation, AUC, and peak recovery) and QC metrics (reads, NSC, RSC) across all 18 imputed Tier 1,2, and 3 assays, ordered according to correlation between imputed and observed within each assay. Bottom tracks for Tier 1 and 2 assays are labeled in red, as above. **c.** Imputation QC metrics reflect external ChIP-seq quality metrics (NSC and RSC). Normalized Strand Cross-correlation coefficient (NSC) against Relative Strand Cross-correlation coefficient (RSC) for observed tracks. Top (green) and bottom (red) tracks are labeled according to automated elbow discovery in imputed vs. observed correlation ranked list. Poor agreement tracks are strongly clustered in lower left quadrant of tracks failing QC. Critical values subdivide the plot for NSC (1.05) and RSC (1).

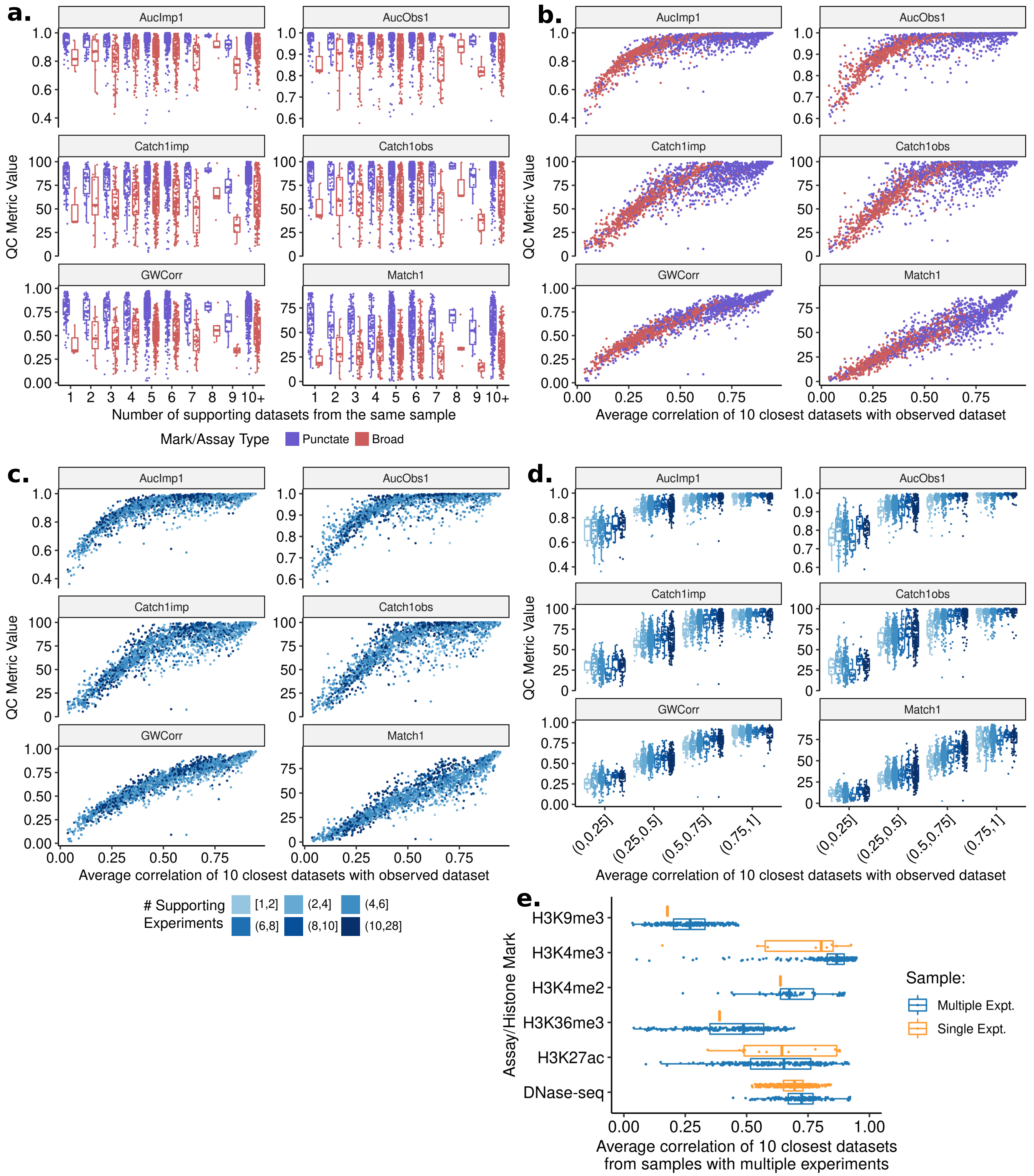

Figure S3: Imputation quality by level of sample-internal and sample-external support. a. Imputation QC metrics in punctate (blue) and broad (red) marks by number of other supporting datasets from the same sample (sample-internal support). **b.** Imputation QC metrics for punctate and broad marks against the average correlation of the 10 closest datasets with the observed dataset. (sample-external support). **c-d.** Metrics against continuous (**c**) and quartiles of (**d**) sample-external support, colored by level of sample-internal support, showing that imputation metrics are dominated by the level of sample-external support. **e.** Level of sample-external support from samples with multiple experiments for samples with only one experiment (orange) or samples with multiple experiments (blue), by available mark or assay. Almost all samples with only DNase-seq have strong cross-sample support from other samples with multiple experiments (average correlation of nearest samples above 50%).

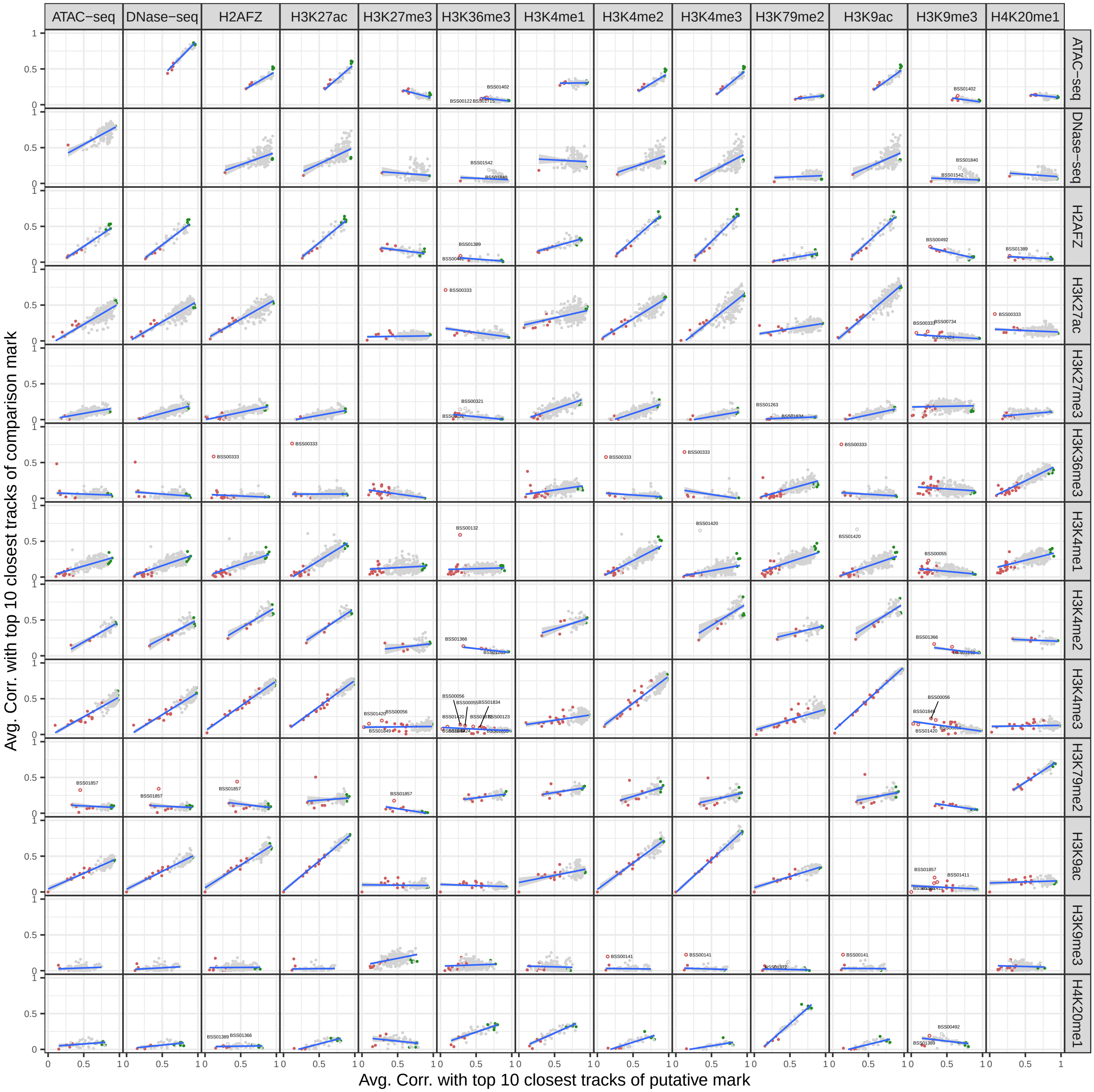

Figure S4: Imputed-observed agreement can be used to systematically flag antibody swaps. Each panel shows the average correlation with closest 10 tracks within the putative mark (each row) for each observed dataset against all other Tier 1 and 2 marks and assays. We used the overall mark-mark trend to flag outliers for visual inspection.

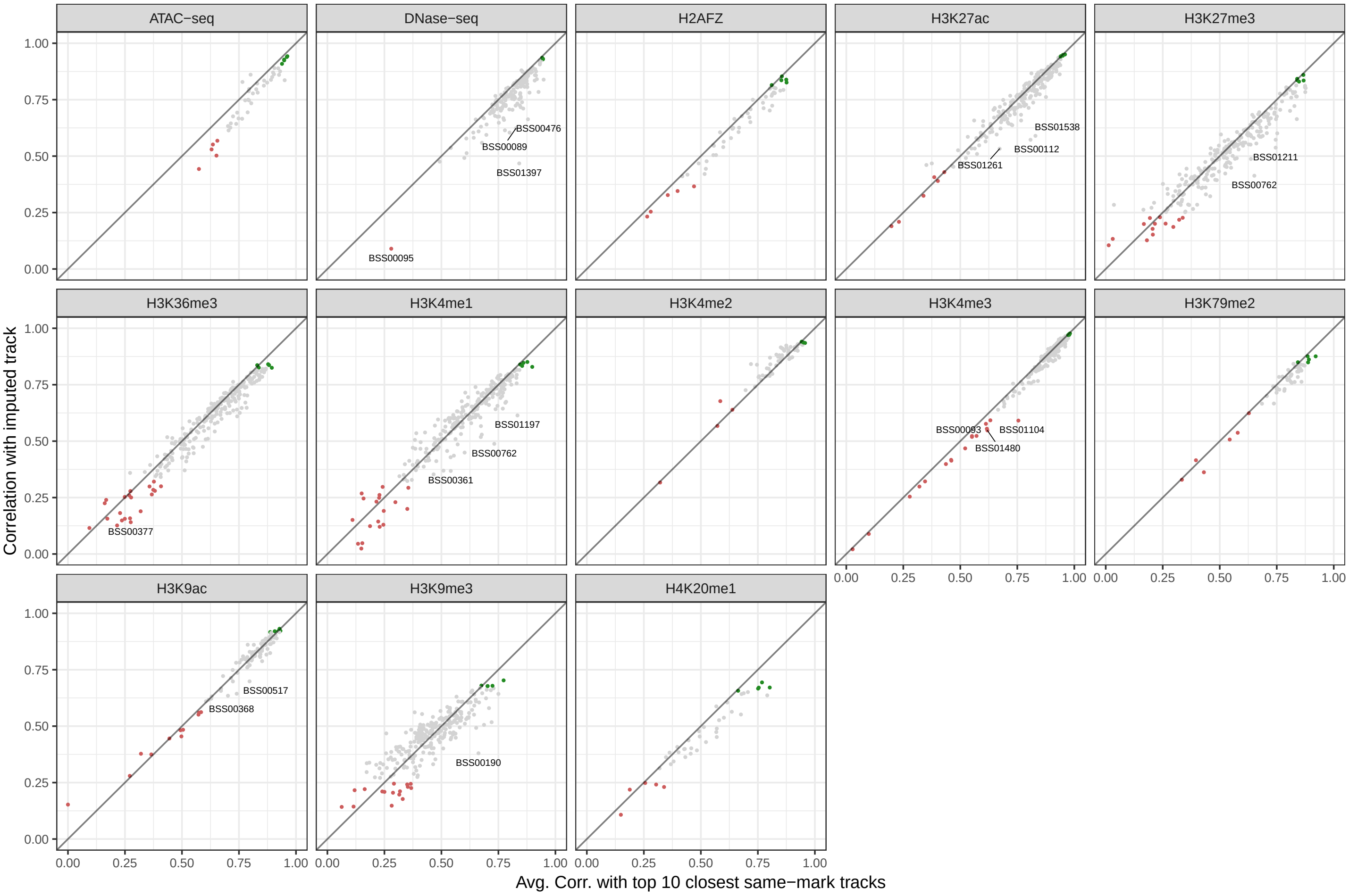

Figure S5: Imputed-observed agreement can be used to flag sample swaps. We compare the imputed-observed correlation to the average imputed-observed correlation within the top 10 closest samples for the putative mark and flagged outliers for visual inspection (after removing antibody swaps).

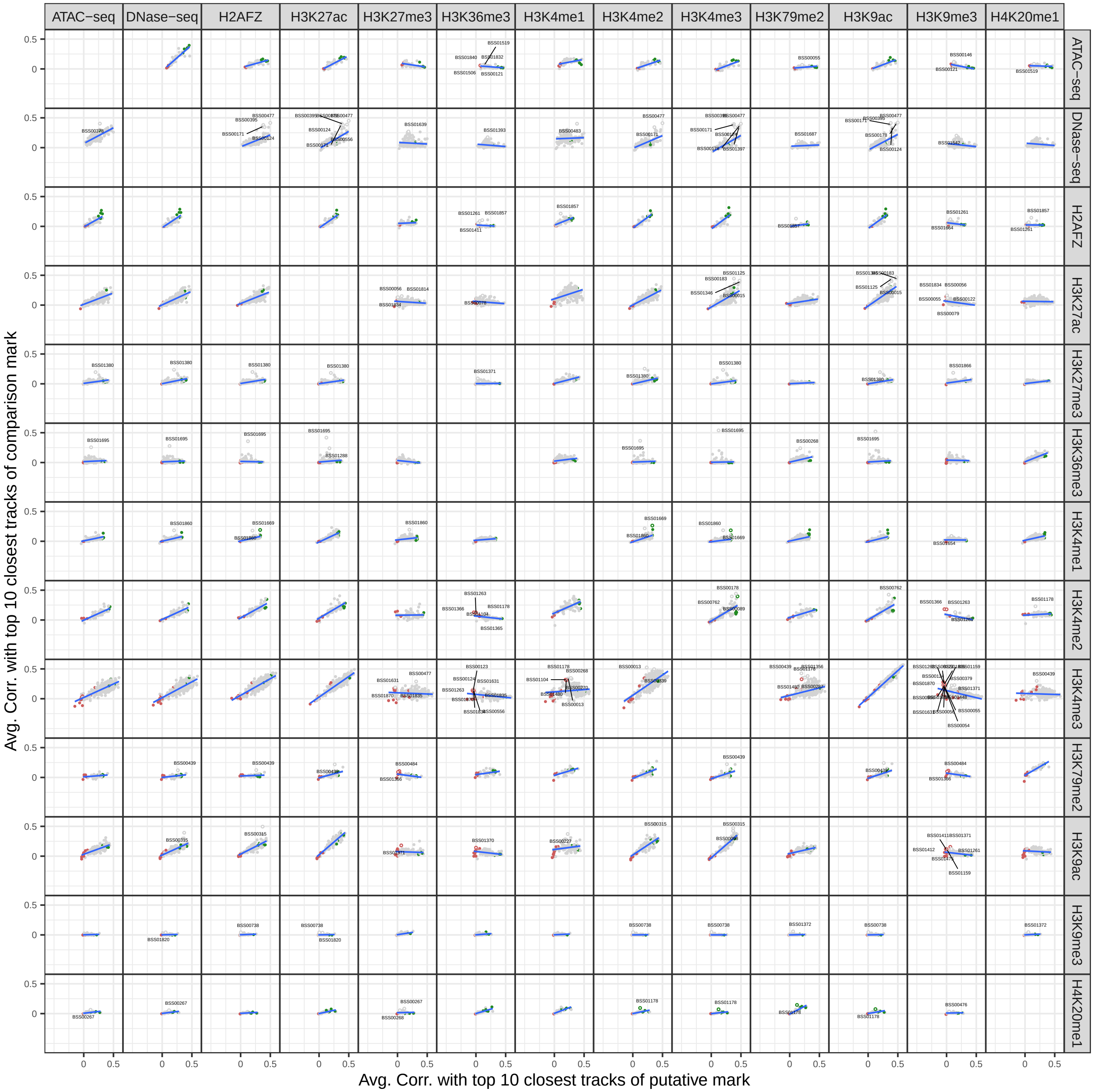

Figure S6: Imputed-observed agreement can be used to systematically flag secondary reactivities of antibodies. Each panel shows the average correlation of the difference between a pair of observed and imputed datasets with closest 10 tracks within the putative mark (each row) for each dataset against all other Tier 1 and 2 marks and assays. As with antibody swaps, we used the overall mark-mark trend to flag outliers for visual inspection.

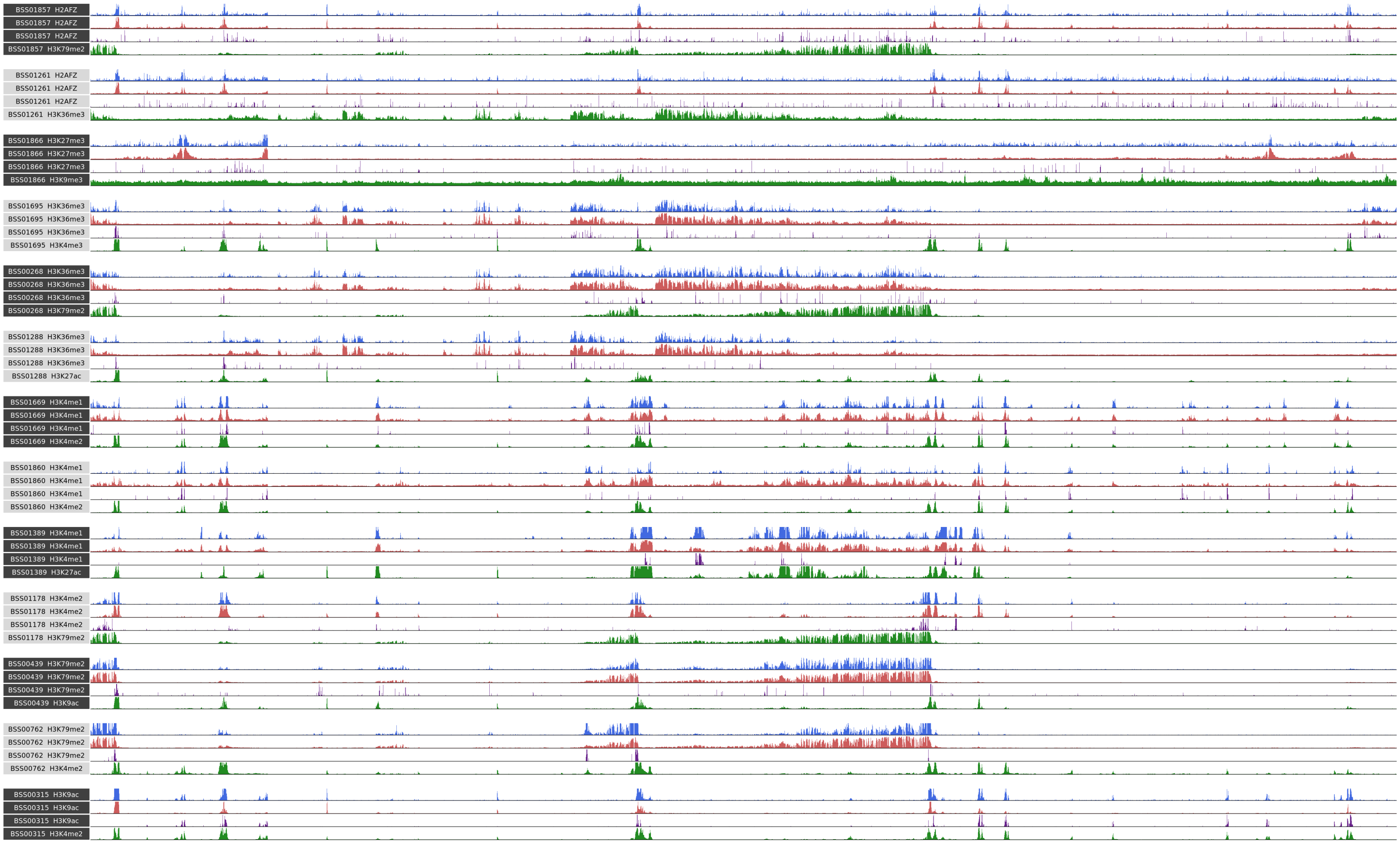

Figure S7: We can use imputation to identify antibodies with secondary reactivities. Track sets for 13 flagged samples highlight a disagreement from secondary reactivity (or sample swapping of one experiment out of multiple replicates), where the difference between observed and imputed best correlate with an external histone mark or assay. Each track set shows the Difference (purple) from Observed (blue) to Imputed (red) and the best Match (green) to the difference (a mark in the same sample) in chromosome 1 from 1.5Mb to 2Mb (chosen to show a range of diverse elements and marks).

	id	mark	(low quality tracks)
1	BSS01365	H3K4me2	
2	BSS01104	H3K4me2	
3	BSS01365	H3K79me2	
4	BSS00556	H3K4me3	
5	BSS01104	H3K4me3	
6	BSS01263	H3K4me3	
7	BSS01815	ATAC-seq	
8	BSS01263	H3K4me2	
9	BSS01475	H3K9ac	
10	BSS01196	H3K9ac	
11	BSS00093	H3K4me3	
12	BSS01506	ATAC-seq	
13	BSS01667	H3K9ac	
14	BSS01480	H3K4me3	
15	BSS00484	H3K79me2	
16	BSS01715	ATAC-seq	
17	BSS00123	H3K4me3	
18	BSS01834	H3K4me3	
19	BSS01835	H3K4me3	
20	BSS01366	H3K79me2	
21	BSS01402	ATAC-seq	
22	BSS01371	H3K9ac	
23	BSS01370	H3K9ac	
24	BSS00284	H3K4me3	
25	BSS00197	H3K9ac	
26	BSS01341	H3K9ac	
27	BSS00122	ATAC-seq	
28	BSS00055	H3K27ac	
29	BSS01870	H3K4me3	
30	BSS01412	H3K79me2	
31	BSS00141	H3K4me3	
32	BSS00074	H3K27ac	
33	BSS00521	H3K4me3	
34	BSS01857	H3K79me2	
35	BSS01866	H3K27ac	
36	BSS01412	H3K9ac	
37	BSS01411	H3K9ac	
38	BSS00478	H2AFZ	
39	BSS00556	H3K79me2	
40	BSS00558	H2AFZ	
41	BSS01411	H3K79me2	
42	BSS00477	H2AFZ	
43	BSS00281	H3K27ac	
44	BSS00055	H3K4me3	
45	BSS00378	H3K36me3	
46	BSS01366	H3K4me2	
47	BSS01857	H3K9ac	
48	BSS01832	H3K36me3	
49	BSS01841	H3K36me3	
50	BSS01424	H3K4me3	
51	BSS00055	H3K4me1	
52	BSS00087	H3K4me1	
53	BSS00080	H3K4me1	
54	BSS00325	H3K36me3	
55	BSS00556	H3K36me3	
56	BSS01340	H3K9ac	
57	BSS00284	H3K36me3	
58	BSS00055	H3K36me3	
59	BSS00281	H3K4me1	
60	BSS01543	H3K36me3	
61	BSS01835	H3K4me1	
62	BSS01080	H3K36me3	
63	BSS00056	H3K4me3	
64	BSS01389	H2AFZ	
65	BSS00493	H3K36me3	
66	BSS01835	H3K36me3	
67	BSS00284	H3K4me1	
68	BSS01389	H4K20me1	
69	BSS00054	H3K4me1	
70	BSS00122	H3K9me3	
71	BSS00123	H3K9me3	
72	BSS01850	H3K9me3	
73	BSS01213	H4K20me1	
74	BSS00093	H3K36me3	
75	BSS00132	H3K4me1	
76	BSS00492	H2AFZ	
77	BSS01876	H3K4me1	
78	BSS01370	H3K9me3	
79	BSS00439	H4K20me1	
80	BSS00325	H3K4me1	
81	BSS01178	H3K27me3	
82	BSS00141	H3K9me3	
83	BSS01849	H3K27me3	
84	BSS01543	H3K9me3	
85	BSS00284	H3K27me3	
86	BSS00054	H3K36me3	
87	BSS00493	H3K9me3	
88	BSS00341	H4K20me1	
89	BSS00325	H3K27me3	
90	BSS00074	H3K9me3	
91	BSS01426	H3K9me3	
92	BSS00483	H3K9me3	
93	BSS00734	H3K27ac	
94	BSS01715	H3K9me3	
95	BSS00325	H3K9me3	
96	BSS01850	H3K27me3	
97	BSS01407	H3K27me3	
98	BSS00381	H3K4me1	
99	BSS00395	H3K27me3	
100	BSS00381	H3K9me3	
101	BSS00321	H3K4me1	
102	BSS01424	H3K27ac	
103	BSS00124	H3K36me3	
104	BSS01507	H3K27me3	
105	BSS01715	H3K36me3	
106	BSS01209	H3K27me3	
107	BSS01832	H3K9me3	
108	BSS01850	H3K36me3	
109	BSS01411	H3K36me3	
110	BSS01370	H3K36me3	
111	BSS01366	H3K9ac	
112	BSS01870	H3K27me3	
113	BSS01834	H3K4me1	
114	BSS01887	H3K36me3	
115	BSS01831	H3K9me3	
116	BSS01630	H3K4me1	
117	BSS01459	H3K9me3	
118	BSS01424	H3K9me3	
119	BSS00377	H3K36me3	
120	BSS00159	H3K27me3	
121	BSS01507	H3K4me1	
122	BSS01837	H3K27me3	
123	BSS01426	H3K36me3	
124	BSS01837	H3K4me1	
125	BSS01426	H3K4me1	
126	BSS01459	H3K36me3	
127	BSS00484	H4K20me1	
128	BSS00531	H3K27me3	
129	BSS00333	H3K36me3	
130	BSS00093	H3K27me3	
131	BSS01562	DNase-seq	
132	BSS00095	DNase-seq	
133	BSS01420	H3K4me3	
134	BSS00333	H3K27ac	
135	BSS01849	H3K4me1	
136	BSS01519	H3K4me1	
137	BSS01543	H3K4me1	
138	BSS01849	H3K4me3	

	id	mark	potential.abswap
1	BSS00333	H3K36me3	H3K27ac
2	BSS00333	H3K27ac	H3K36me3
3	BSS01857	H3K9ac	H3K79me2
4	BSS01857	H3K79me2	H3K9ac
5	BSS00141	H3K9me3	H3K9ac
6	BSS00132	H3K4me1	H3K36me3
7	BSS01420	H3K4me1	H3K4me3

	id	mark	potential.sampswap
1	BSS00089	DNase-seq	BSS00339
2	BSS00095	DNase-seq	BSS01452
3	BSS00476	DNase-seq	BSS01397
4	BSS01397	DNase-seq	BSS00334
5	BSS00112	H3K27ac	BSS01366
6	BSS01261	H3K27ac	BSS00387
7	BSS01538	H3K27ac	BSS00703
8	BSS00762	H3K27me3	BSS00547
9	BSS01211	H3K27me3	BSS01224
10	BSS00377	H3K36me3	BSS01715
11	BSS00361	H3K4me1	BSS00365
12	BSS00762	H3K4me1	BSS01038
13	BSS01197	H3K4me1	BSS01144
14	BSS00093	H3K4me3	BSS00702
15	BSS01104	H3K4me3	BSS01365
16	BSS01480	H3K4me3	BSS00702
17	BSS00368	H3K9ac	BSS00207
18	BSS00517	H3K9ac	BSS00502
19	BSS00190	H3K9me3	BSS00196

	id	mark	potential.secondary
1	BSS01857	H2AFZ	H3K79me2
2	BSS01261	H2AFZ	H3K36me3
3	BSS01866	H3K27me3	H3K9me3
4	BSS01695	H3K36me3	H3K4me3
5	BSS00268	H3K36me3	H3K79me2
6	BSS01288	H3K36me3	H3K27ac
7	BSS01669	H3K4me1	H3K4me2
8	BSS01860	H3K4me1	H3K4me2
9	BSS01389	H3K4me1	H3K27ac
10	BSS01178	H3K4me2	H3K79me2
11	BSS00439	H3K79me2	H3K9ac
12	BSS00762	H3K79me2	H3K4me2
13	BSS00315	H3K9ac	H3K4me2

Figure S8: Table of flagged samples. **(left)** low agreement tracks, **(right, top)** potential antibody swaps, **(right, middle)** potential sample swaps, **(right, bottom)** potential secondary antibody reactivities or single replicate or experiment swaps.

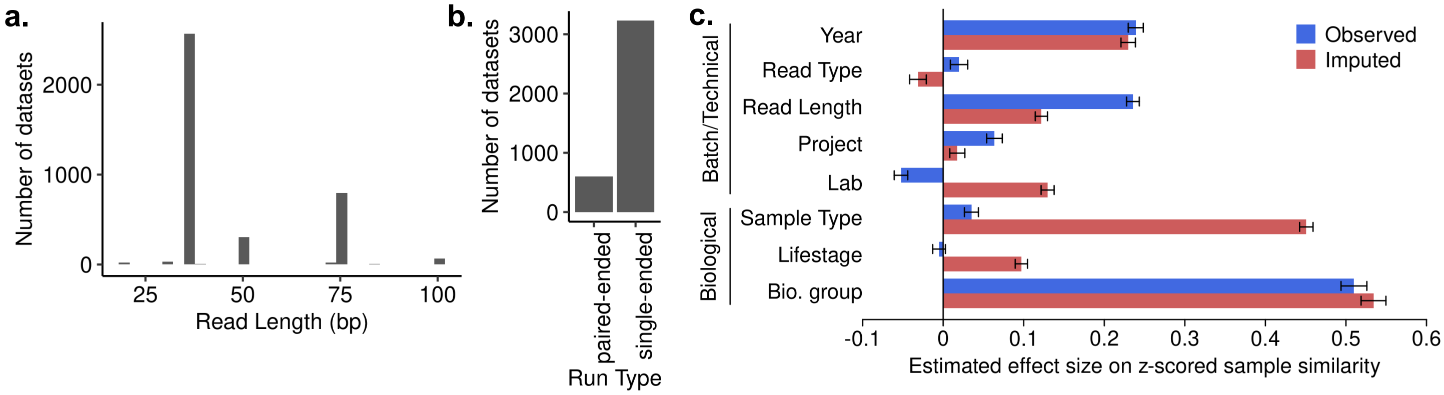

Figure S9: a. Read length distribution in observed datasets before processing. Over two-thirds of observed datasets have read-length of 36bp or lower. **b.** Distribution of sequencing run type in observed datasets. **c.** Estimated effect sizes of technical and biological covariates on the genome-wide correlation between two imputed or two observed datasets in the same mark. We regressed indicator variables for each covariate against per-mark z-scored correlations of all pair-wise combinations of either observed or imputed datasets in each mark. Imputed datasets show lower effect sizes from all technical covariates except for lab or origin and higher effect sizes for all biological covariates than observed datasets. Error bars represent two standard errors.

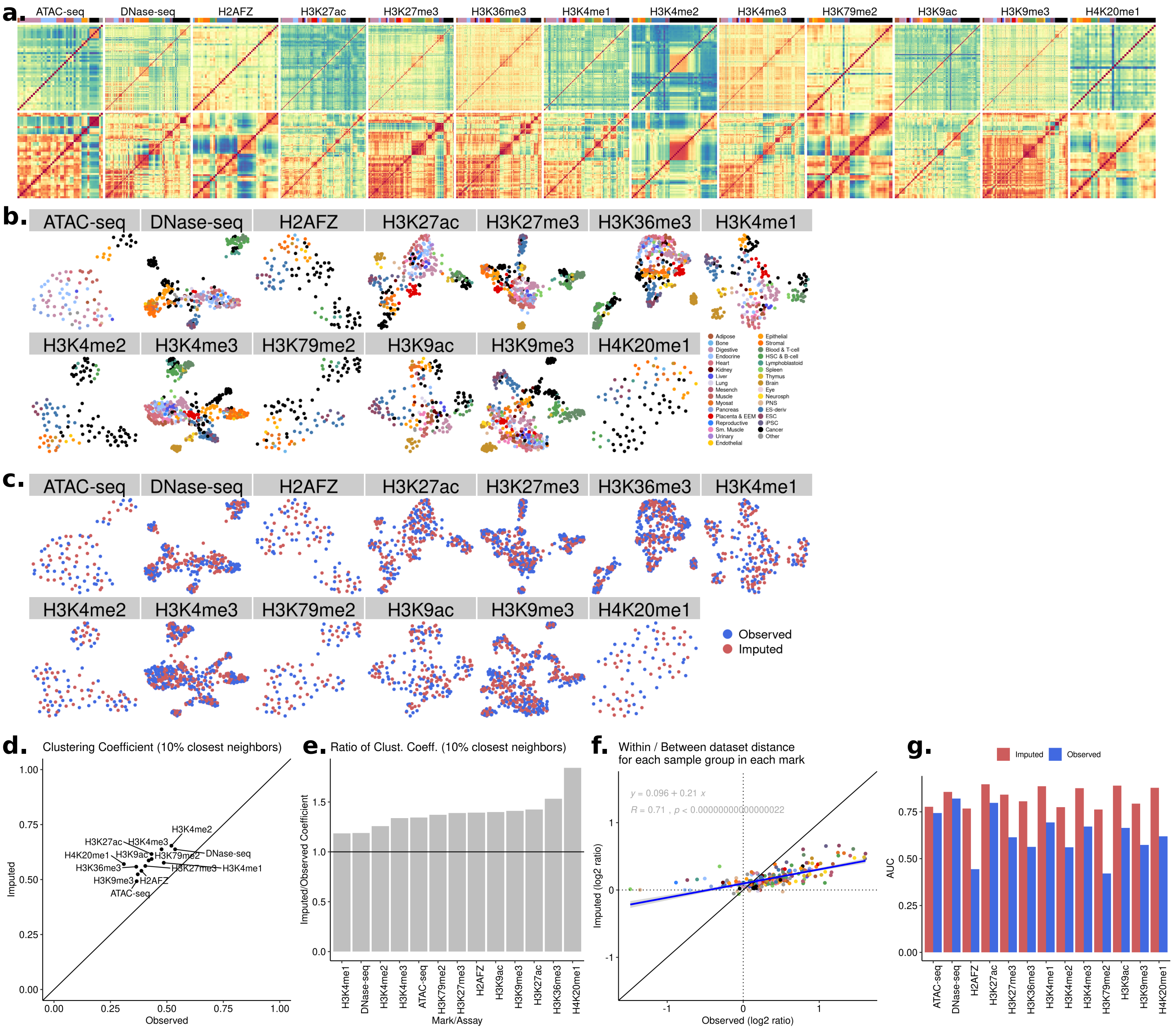

Figure S10: Data clustering and homogeneity in samples with both observed and imputed data. **a.** Per-mark heatmap of Spearman correlation for observed (top) and imputed (bottom) data in samples with both observed and imputed data in the mark or assay. **b,c.** Joint imputed and observed per-mark dimensionality reduction (UMAP) from Spearman correlation between all imputed and observed tracks in the subset of samples with both datatypes, colored by **b.** sample group and **c.** datatype. **d.** Per-mark observed versus imputed clustering coefficients. Clustering coefficients were computed on per-mark networks for either observed or imputed datasets constructed by connecting each dataset with its top decile of closest neighbors. **e.** Ratio of imputed to observed clustering coefficients. **f.** Ratio of within-group to between-group distance in observed versus imputed data for each sample group (colors) and each mark. Imputed and observed data show concordant ratios (Pearson $R = 0.72$, Spearman $\rho = 0.71$) **g.** AUC for predicting whether a pair of marks is in the same sample group from their pairwise distance. Imputed data outperforms observed data across all marks and assays and achieves $AUC > 0.75$ for most marks.

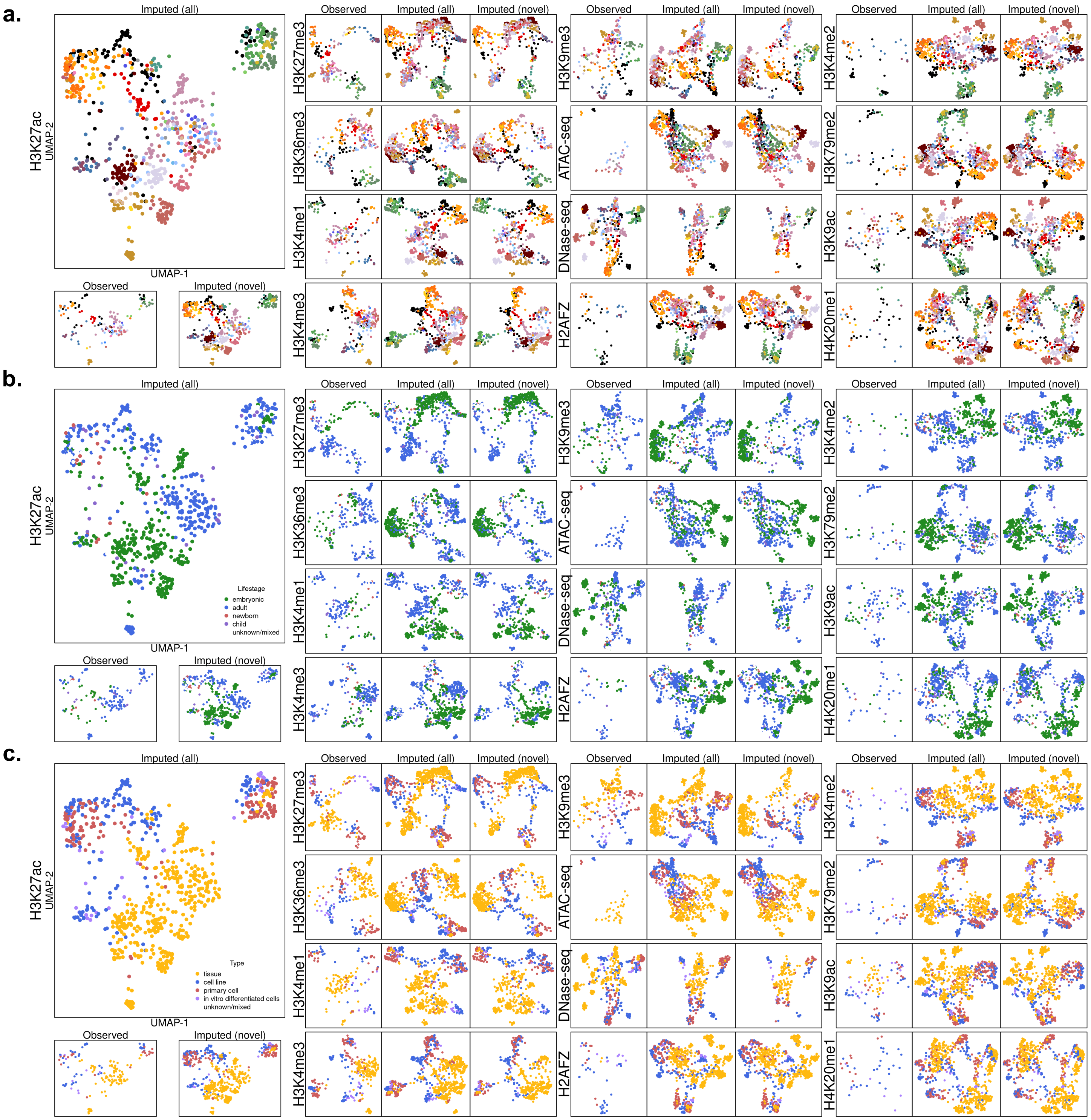

Figure S11: a-c. Joint UMAP embeddings of observed and imputed data within each Tier 1 and 2 mark/assay. Separately, observed, imputed samples, and all novel imputed tracks are plotted for each mark/assay and colored according to **a.** tissue group, **b.** biological lifestage, or **d.** sample type. Imputed UMAP highlights differences in cell types: H3K27ac clusters hematopoietic cells and tissues (green in panel a) closely, reflecting lineage, whereas H3K27me3 clusters iPSC, ESCs, and derived cells (purple/blue in panel a), reflecting differentiation stage. UMAP embedding was calculated from spearman correlation of tracks within regions marked by mark or assay relevant states within the Roadmap compendium.

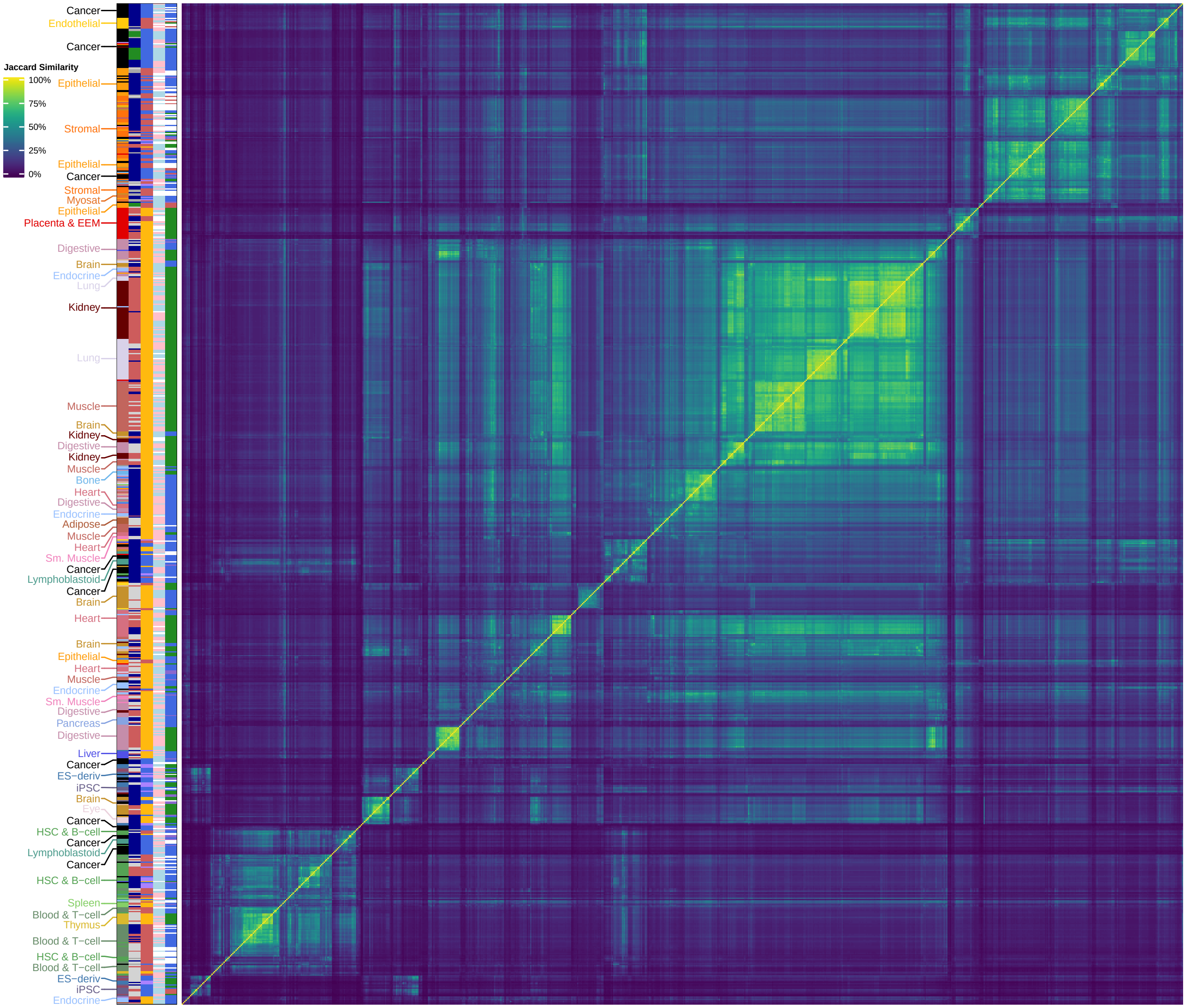

Figure S12: Jaccard similarity matrix (heatmap) across 833 epigenomes (metadata on left) from binarized enhancer activity matrix (2.1M enhancers by 833 epigenomes). Similarity matrix clustered by complete-linkage clustering. Consecutive blocks of at least six samples from the same group are labeled on the left.

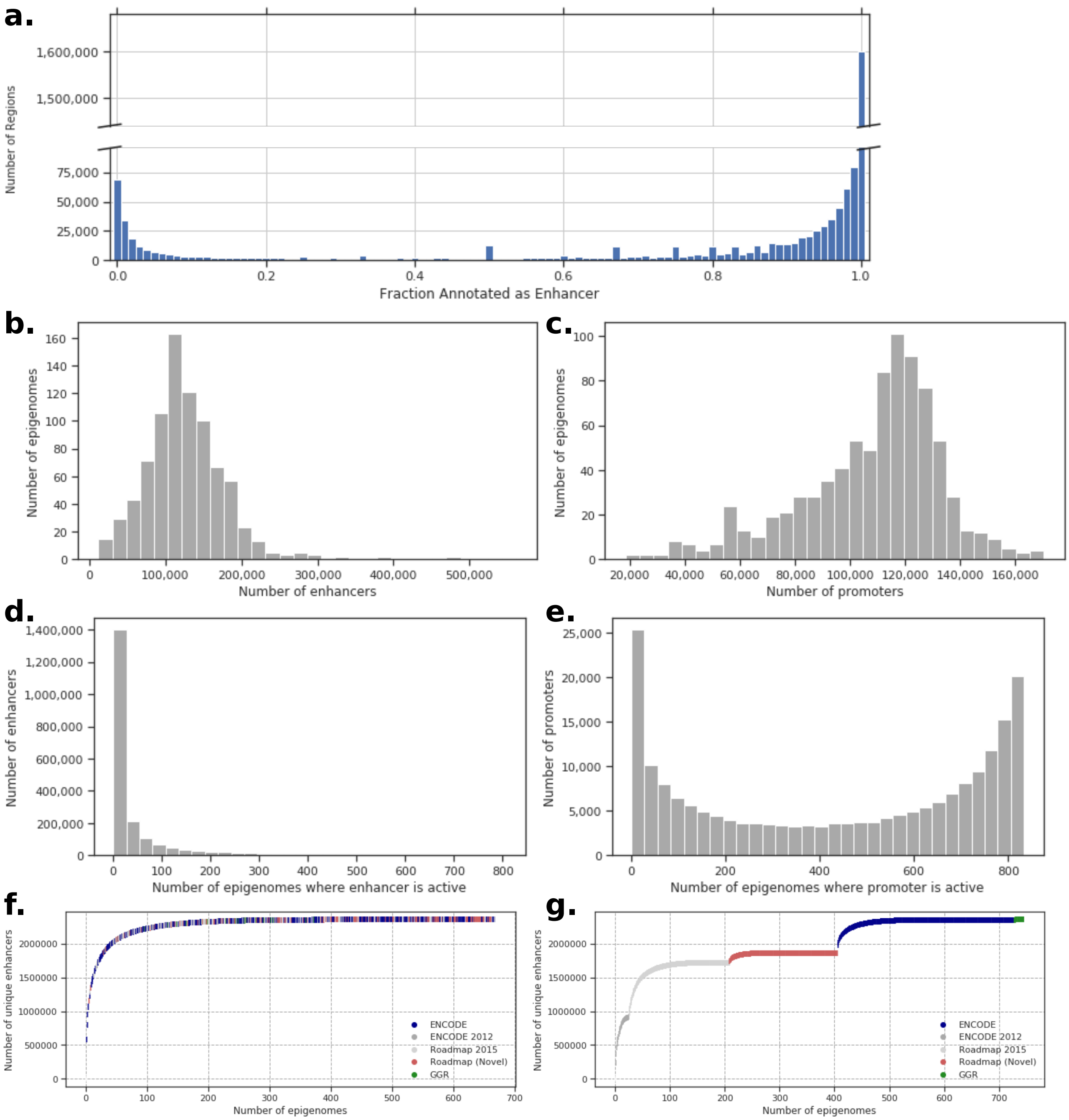

Figure S13: a. Number of DHS sites annotated as enhancers instead of promoters across 833 epigenomes. Most regions are either labeled enhancer (at least 75% of occurrences are enhancers) or promoter (at least 75% of occurrences are promoters) across all of their active occurrences. Using these cutoffs, we labeled 2,069,090 enhancers, 204,104 promoters, and 122,358 dyadic elements (neither specifically promoter or enhancer). **b,c.** Histogram of number of active enhancers (**b**) and promoters (**c**) per sample (row-margins of binary activity matrix). **d,e.** Histogram of the number of samples for which each enhancer (**d**) or promoter (**e**) is an active enhancer (column-margins of binary activity matrix) **f.** Rarefaction curve for enhancer recovery for all enhancer and dyadic elements (2.3M total) across 833 samples (points, colored by project of origin). Curve was created by iteratively adding the sample contributing the most novel active elements until all 2.3M elements were accounted for. **g.** Rarefaction curve for enhancer recovery, shown in order of project completion, for all enhancer and dyadic elements (2.3M total) across 833 samples (points, colored by project of origin). Curve was created by iteratively adding the sample contributing the most novel active elements until all 2.3M elements were accounted for. Samples were considered in order of project publication/completion, only taking samples from the next project when the current project did not contribute any more enhancers.

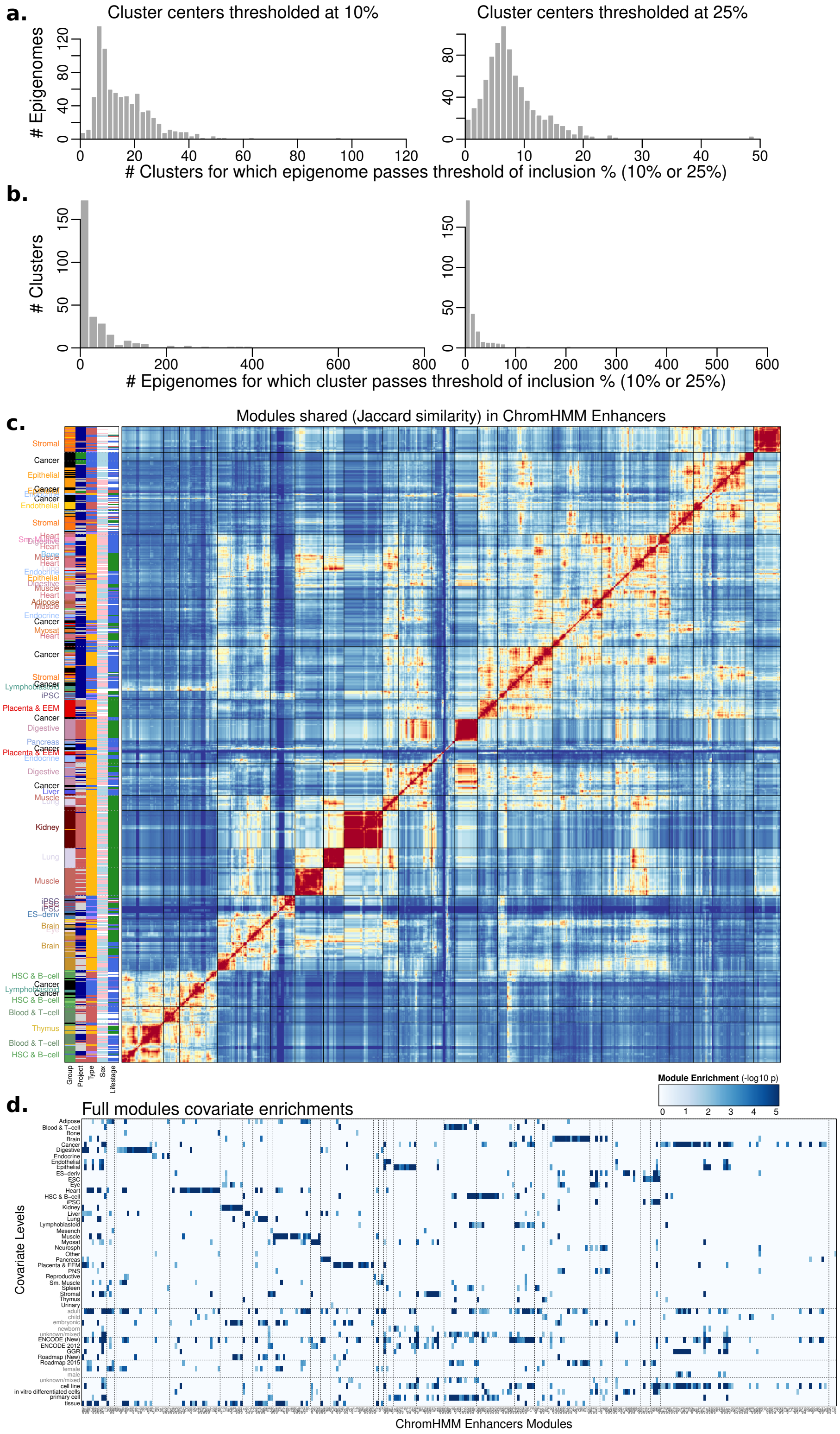

Figure S14: a,b. Module inclusion distributions according to two different module inclusion cutoffs of 10% and 25% (columns). **a.** Number of modules (N=300) for each of 833 epigenomes with module inclusion of at least 10% or 25% (top). **b.** Number of epigenomes (N=833) for each of 300 modules at the same inclusion cutoffs. **c.** Sample similarity (heatmap) across 833 epigenomes by their number of shared modules.(jaccard similarity, intersection over union of modules of each pair of epigenomes). Similarity matrix clustered by Ward's method. Consecutive blocks of at least six samples from the same group are labeled on the left. **d.** Full module enrichments across main metadata facets of group, project, type, sex, and lifestage, for which the reduced version is shown as a panel in Figure 2a. Significance assessed according to hypergeometric test on the module centers matrix with module inclusion of at least 25%.

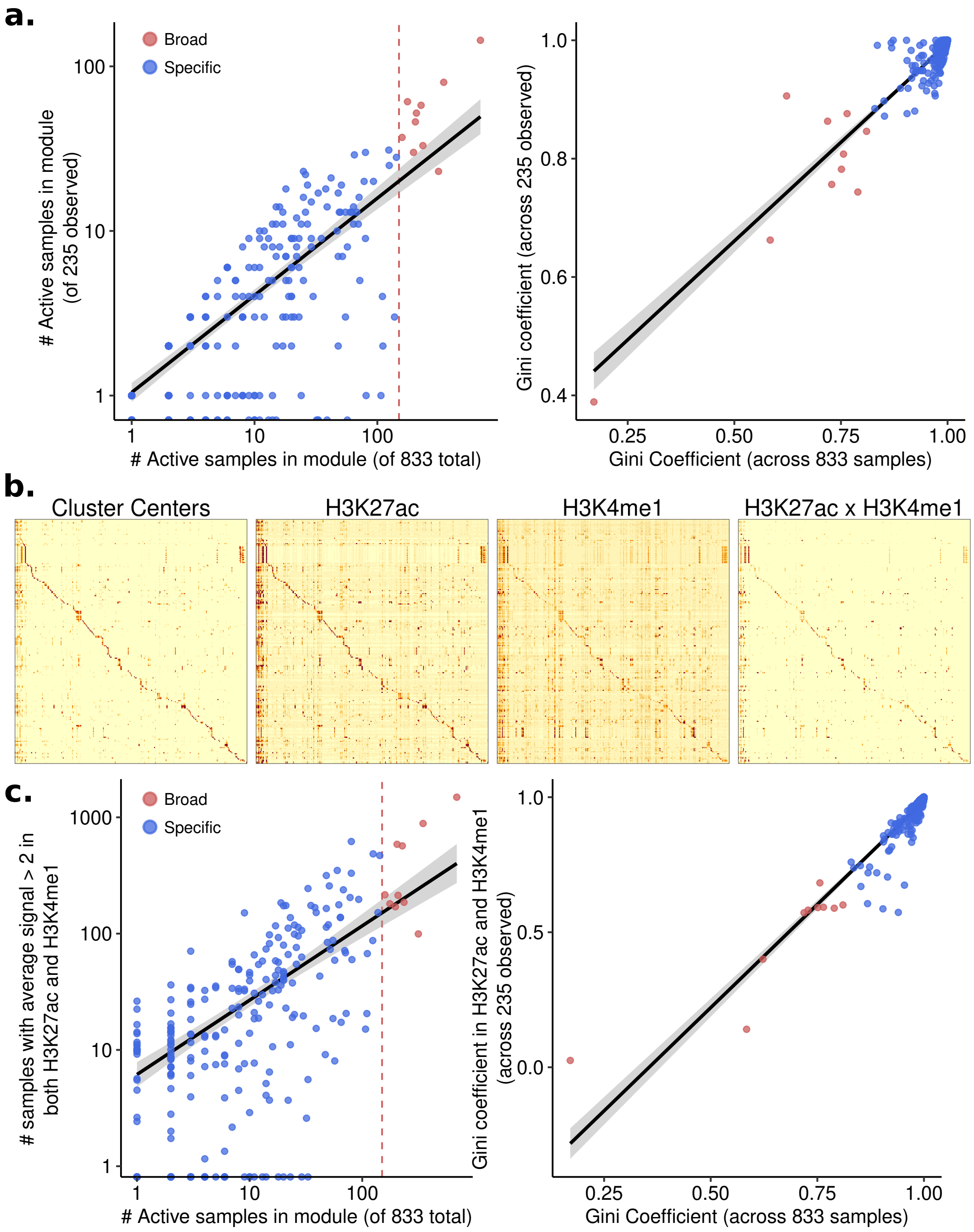

Figure S15: Observed data validation of broad and specific modules. a. Validation with 235 observed H3K27ac datasets. Number of samples (left) and Gini coefficient (right) per module in the full data versus the observed-only H3K27ac data. Broad modules are defined as modules with over 150 actie samples in the full data (dashed line) and labeled in red. Despite the sample composition differences in observed vs. full data, the fraction of active samples is quite consistent between the datasets (left, R^2=0.802, right R^2=0.82). For the 10 broad modules (red) from the full data, all were active in at least 9.7% of observed samples and 24% on average, seven were the most broadly active in terms of active samples in observed, and nine were in the bottom ten modules by Gini coefficient (least unequal, or most broadly expressed). **b.** Side-by-side heatmaps of module centers, average observed H3K27ac, average observed H3K4me1, and the product of H3K27ac and H3K4me1 in 205 datasets with observed data for both marks. **c.** Number of samples (left) and Gini coefficient (right) as in (**a**), for the product of observed H3K27ac and H3K4me1 in 205 datasets with observed data for both marks.

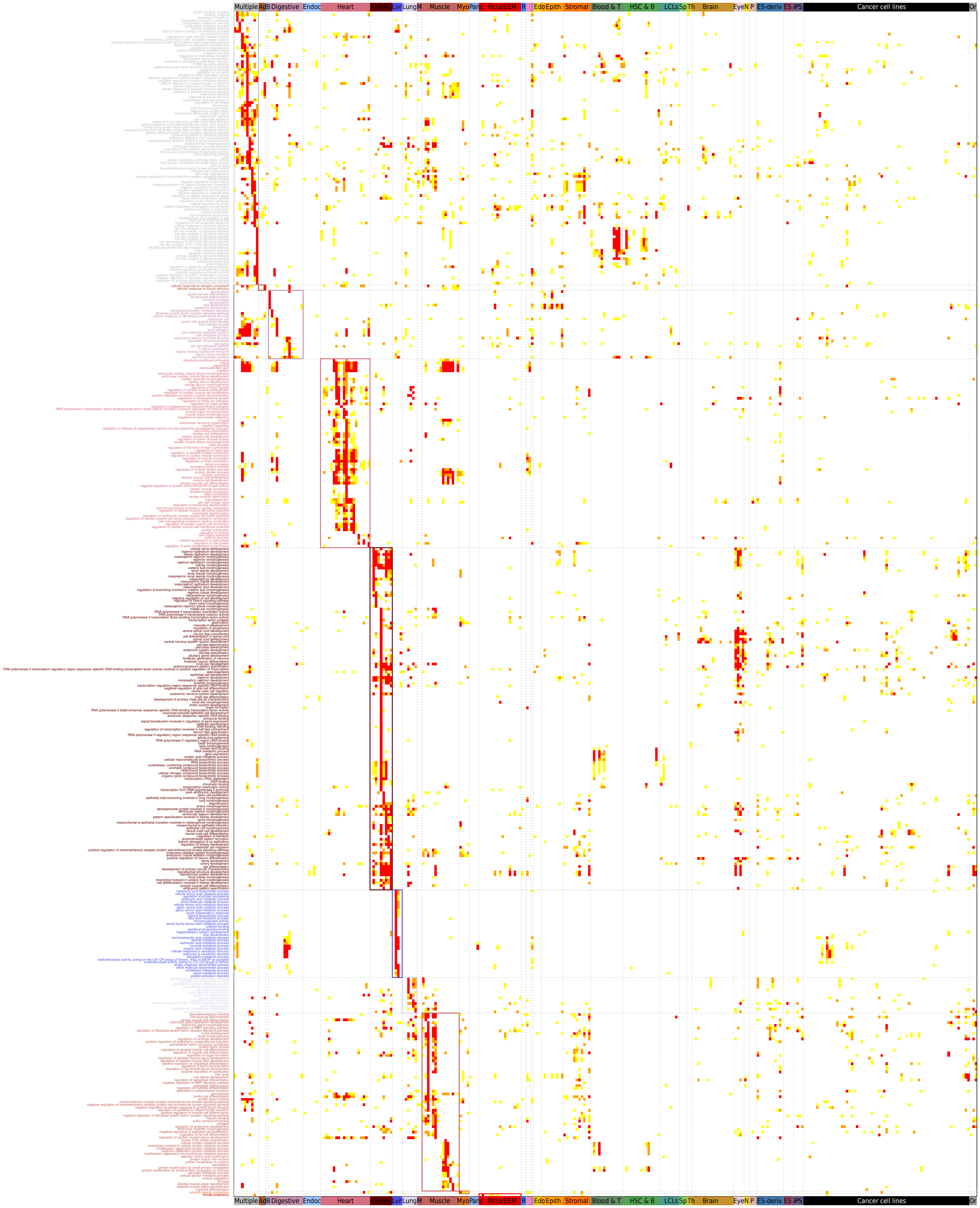

Figure S15: All 865 module-specific GO terms (BP, MF, CC), including all terms enriched in less than 10% of modules and with a maximum enrichment of at least $-\log_{10} p > 4$. Full version of Figure 2b.

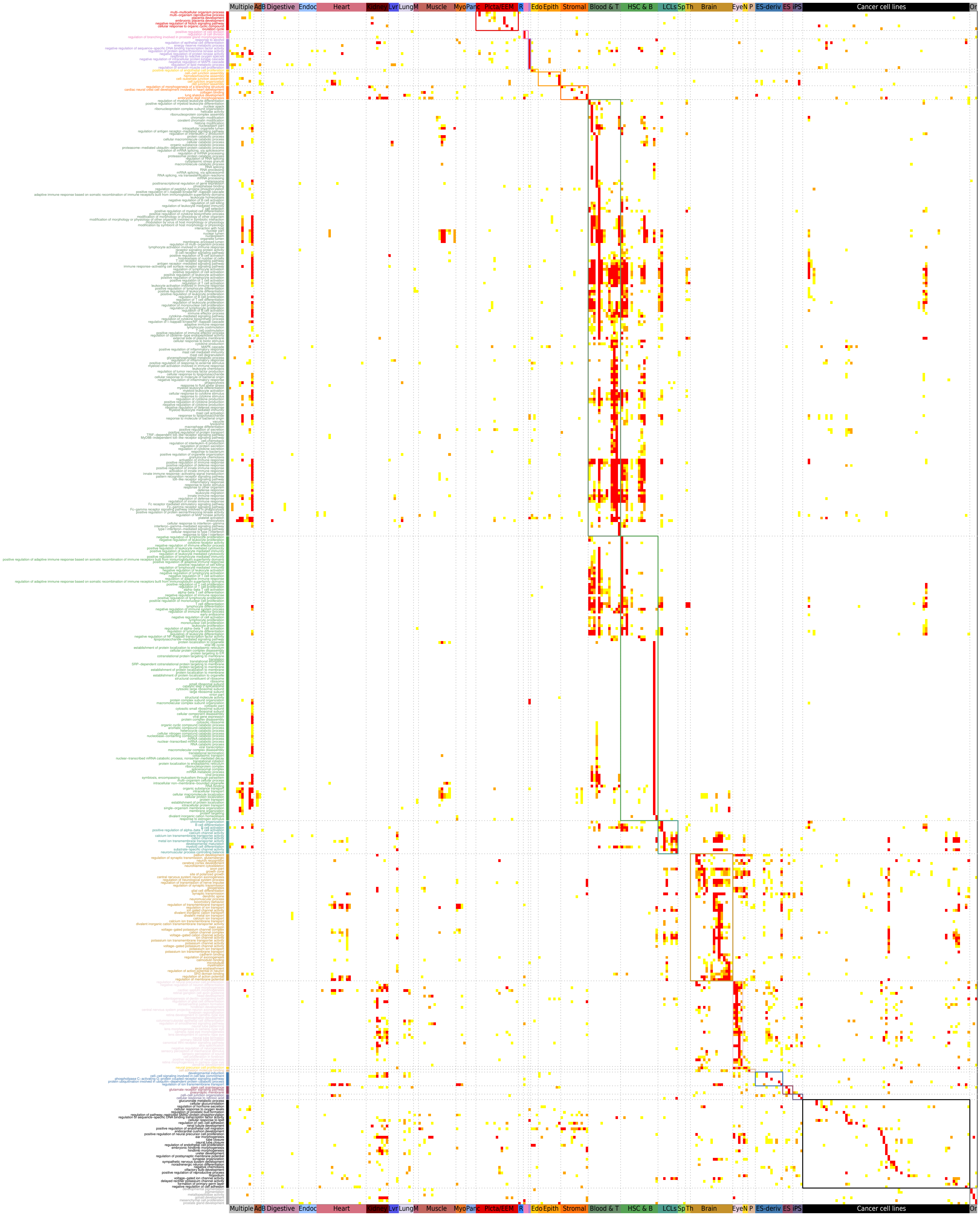

Figure S16: (continued)

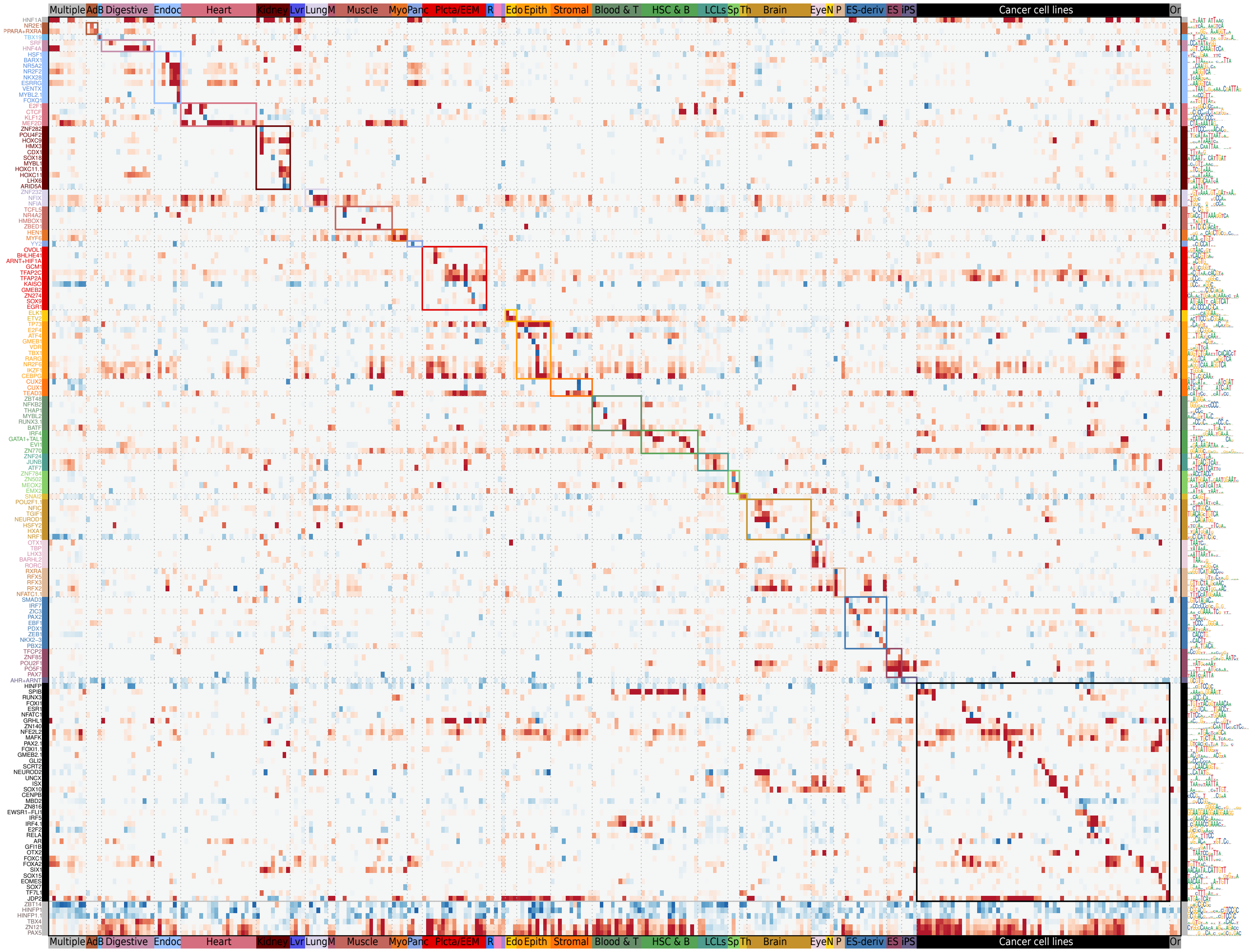

Figure S17: Full motif enrichments for 160 motif families with enrichments with $\log_2$FC ≥ 1 on modules (Figure 2c).

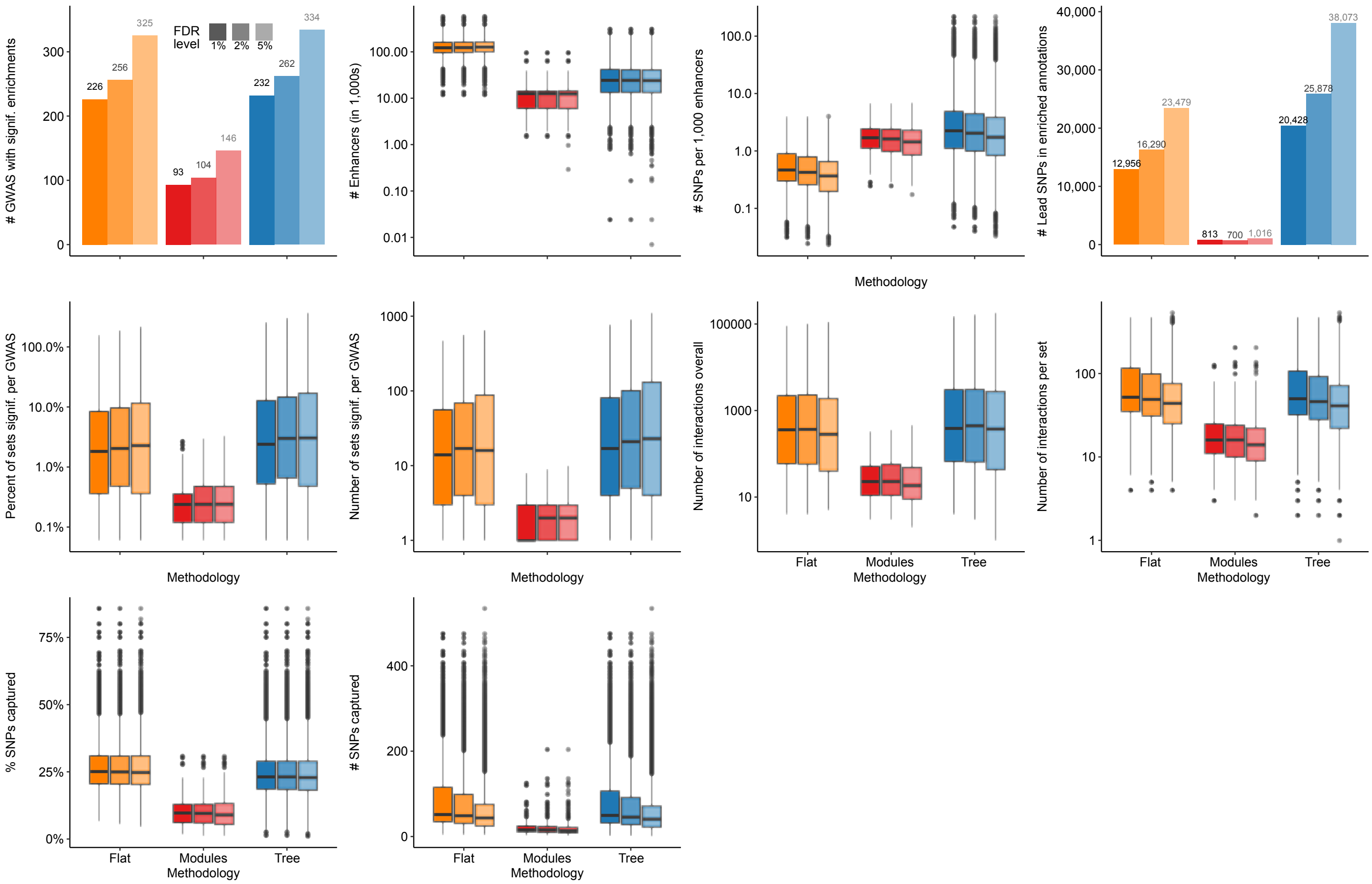

Figure S18: Extended method validation figures comparing epigenomic GWAS enrichments using different methodologies (x-axis) for three FDR cutoffs (shades). First and last figures from top row are shown as Extended Data Fig. 7e.

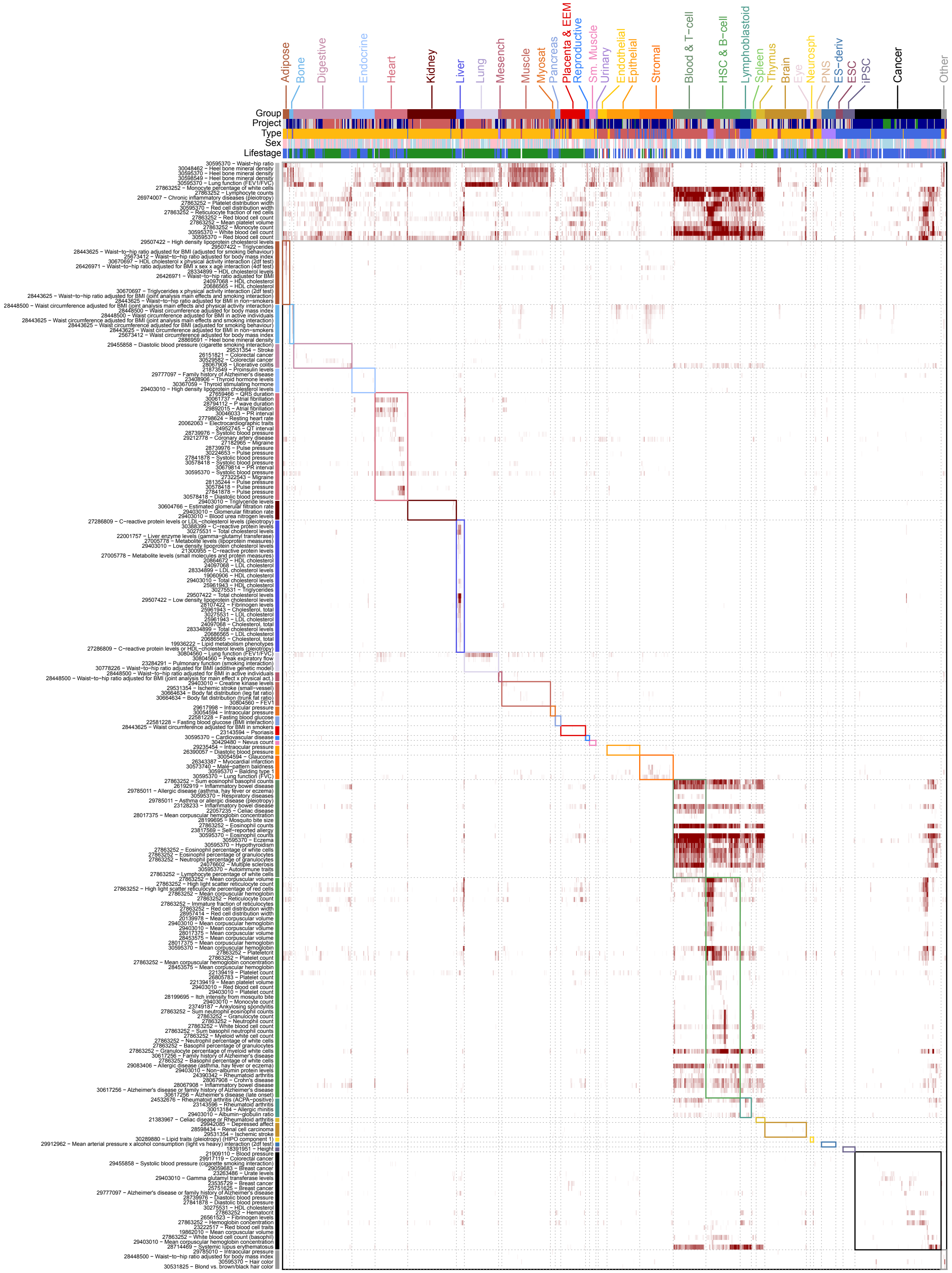

Figure S19: All epigenome trait enrichments at FDR < 1%, for 226 traits (rows) and 833 epigenomes (columns)

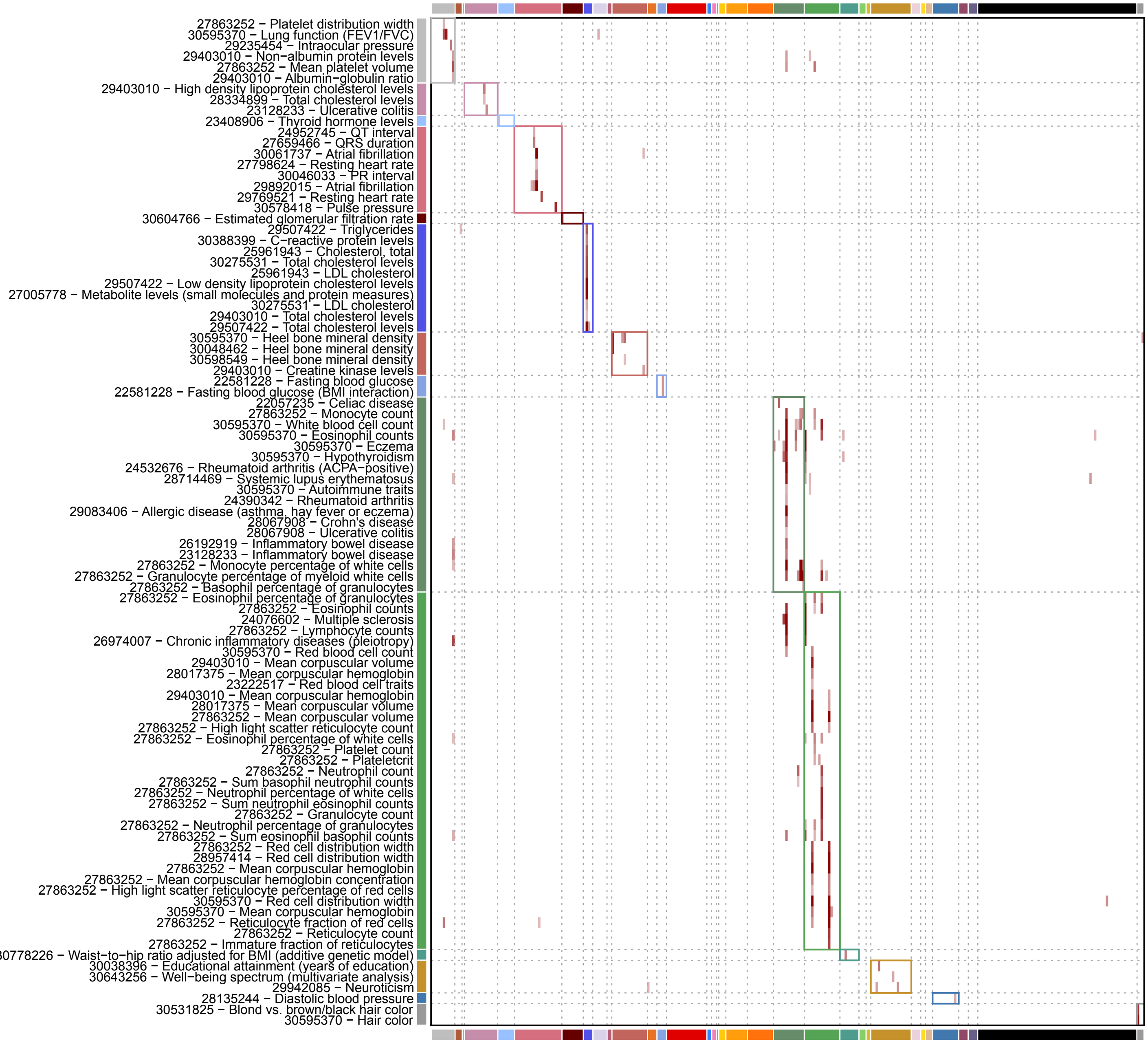

Figure S20: All module trait enrichments at FDR $< 1\%$, for 93 traits (rows) and 300 modules (columns). Modules are ordered as in Figure 2a.

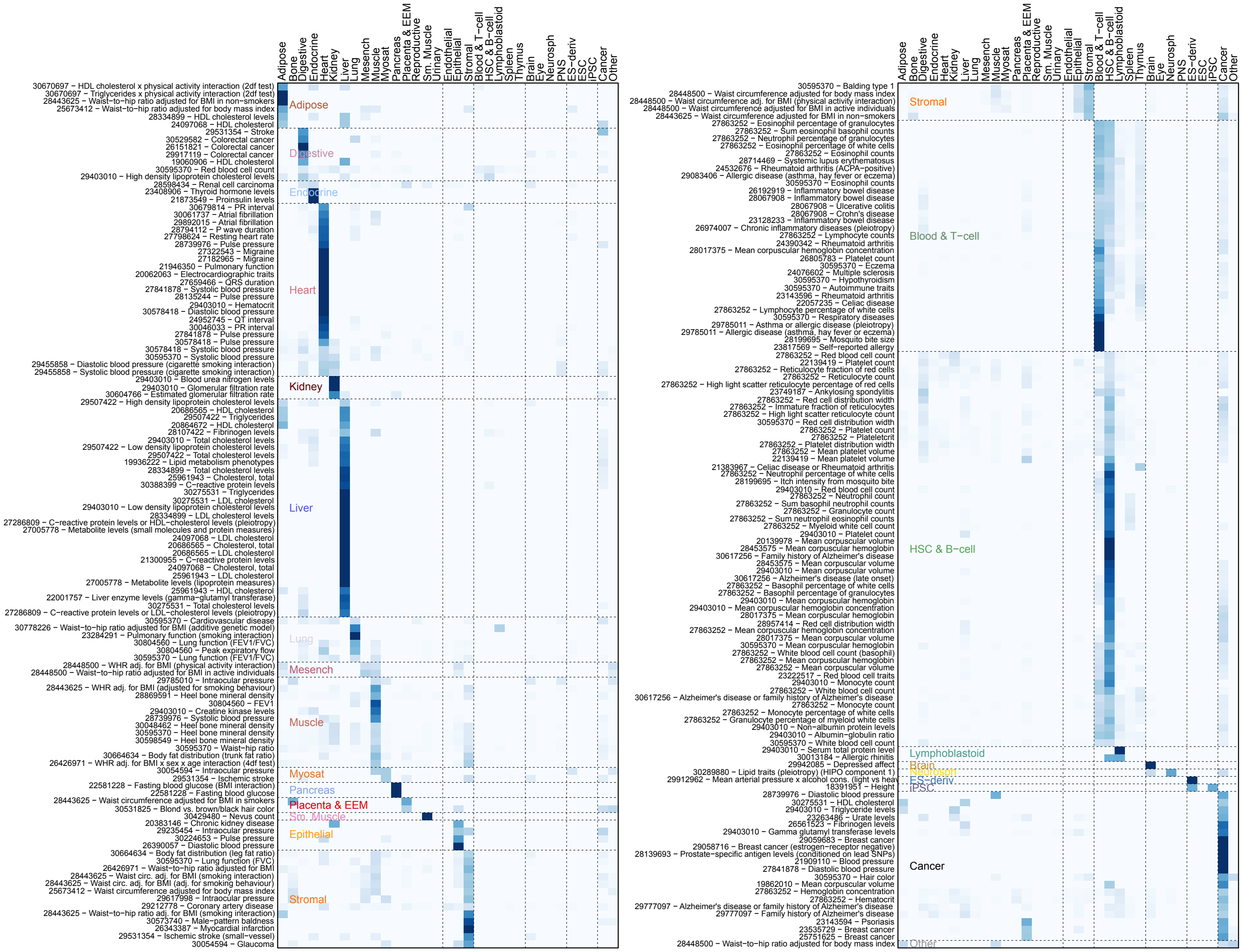

Figure S21: Overall tissue-level prioritization of 230 traits at FDR $< 1\%$. Heatmap represents trait (rows) vs. tissue matrix and is split for visibility. Traits are diagonalized according to their top tissue enrichment, and values are the trait-normalized and tissue aggregated $-\log_{10}$p-values.

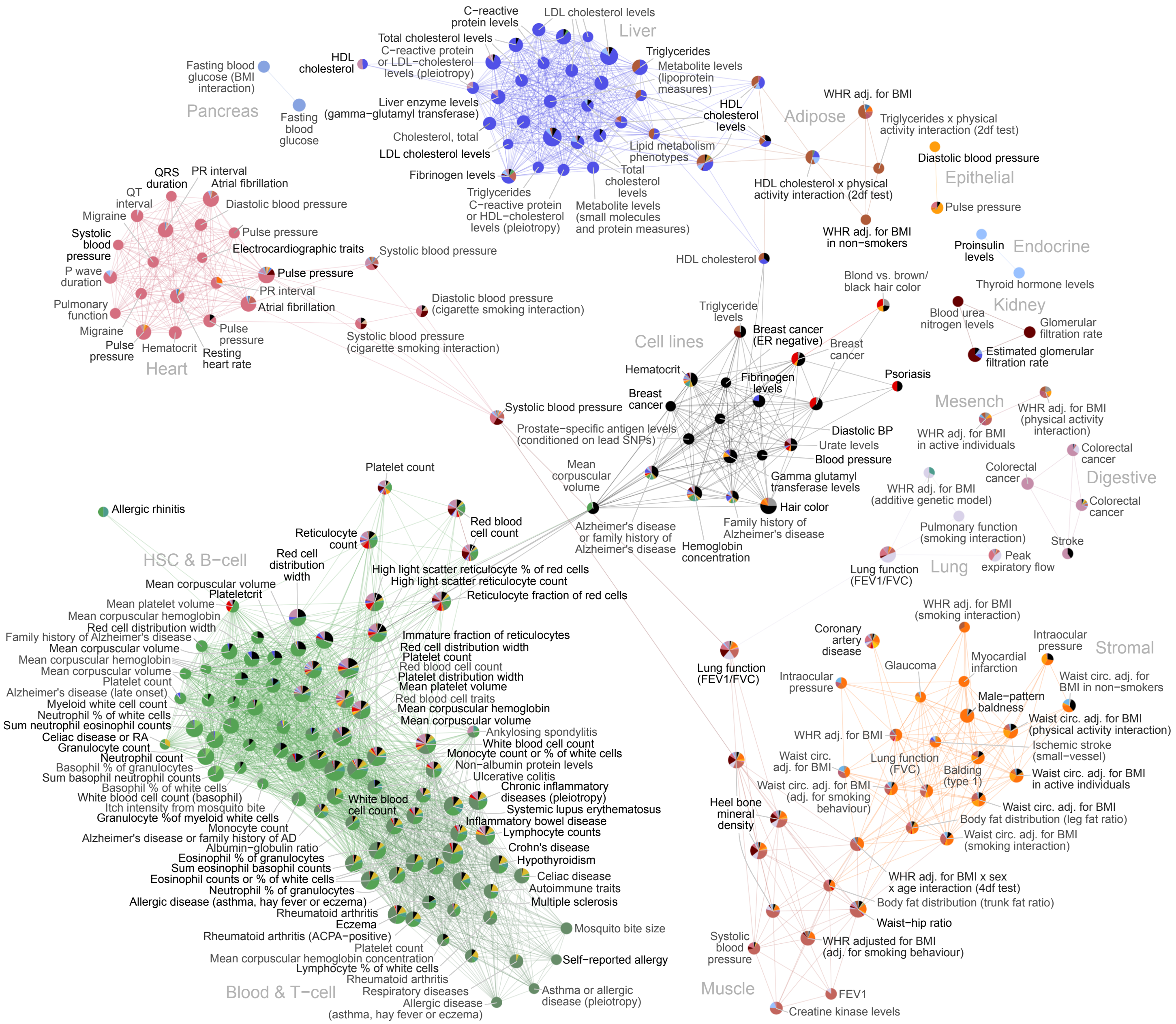

Figure S22: Trait-trait network (as Figure 5) across 215 traits by similarity of epigenetic enrichments (cosine sim. ≥ 0.75), laid out using the Fruchterman-Reingold algorithm. Traits (nodes) are colored by contributing groups (pie chart by fraction of $-\log_{10}$ adjusted p-value, size by maximal $-\log_{10}$ adjusted p-value) and interactions (edges) by the group with maximal dot product of enrichments between two traits. All 215 traits labeled.

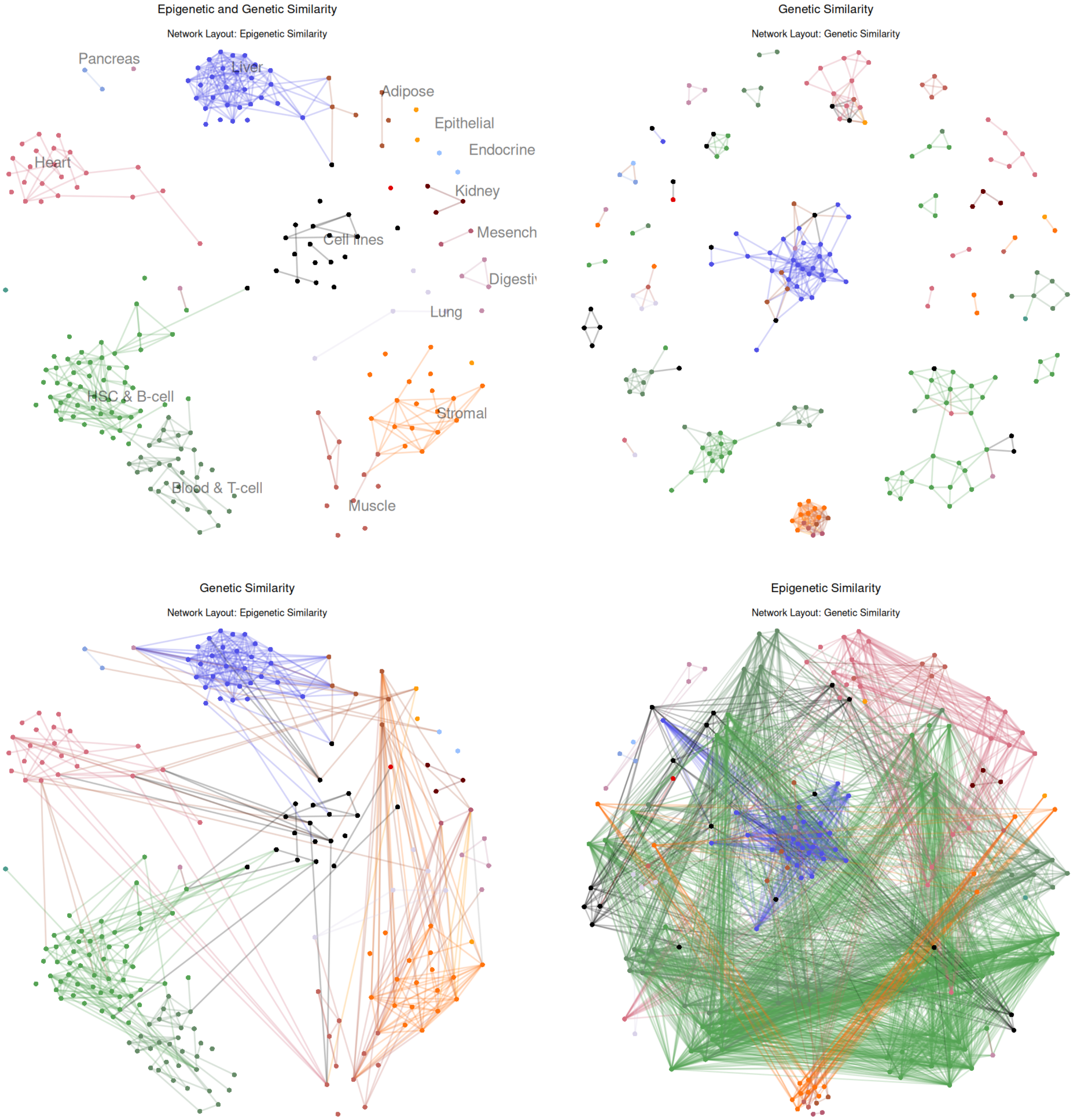

Figure S23: Comparison networks with genetics and epigenetics. (top left) Edges with both high epigenetic similarity and any genetic overlap on epigenetic similarity layout. (bottom left) All trait pairs with genetic overlap (> 5% jaccard similarity of lead SNPs overlapping when binned into 10k bp bins starting from the start of each chromosome, network using epigenetic layout. (top right) Network of trait pairs with any genetic overlap laid out by genetics. (bottom right) All trait pairs with high epigenetic similarity on the genetic layout network

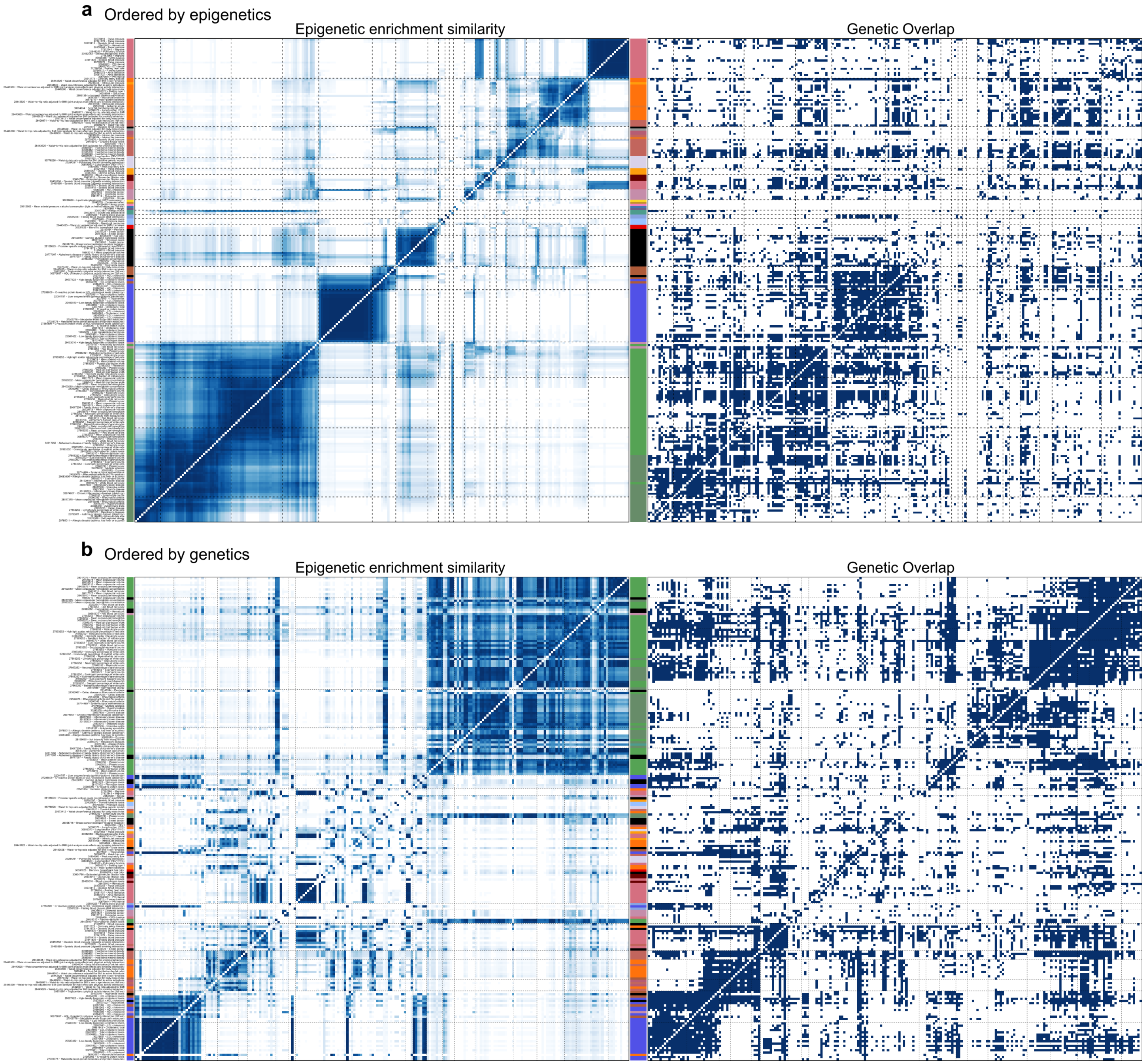

Figure S24: a. Trait-trait similarity matrix by cosine similarity of epigenetic enrichments (left) and by 0/1 genetic similarity (defined as > 5% jaccard similarity of lead SNPs overlapping when binned into 10k bp bins starting from the start of each chromosome). Matrices are ordered by hierarchical clustering according to Ward's method on the epigenetic matrix. **b.** Epigenetic similarity (left) and genetic similarity (right) matrices, as above. Matrices ordered by hierarchical clustering according to Ward's method on the genetic similarity matrix.

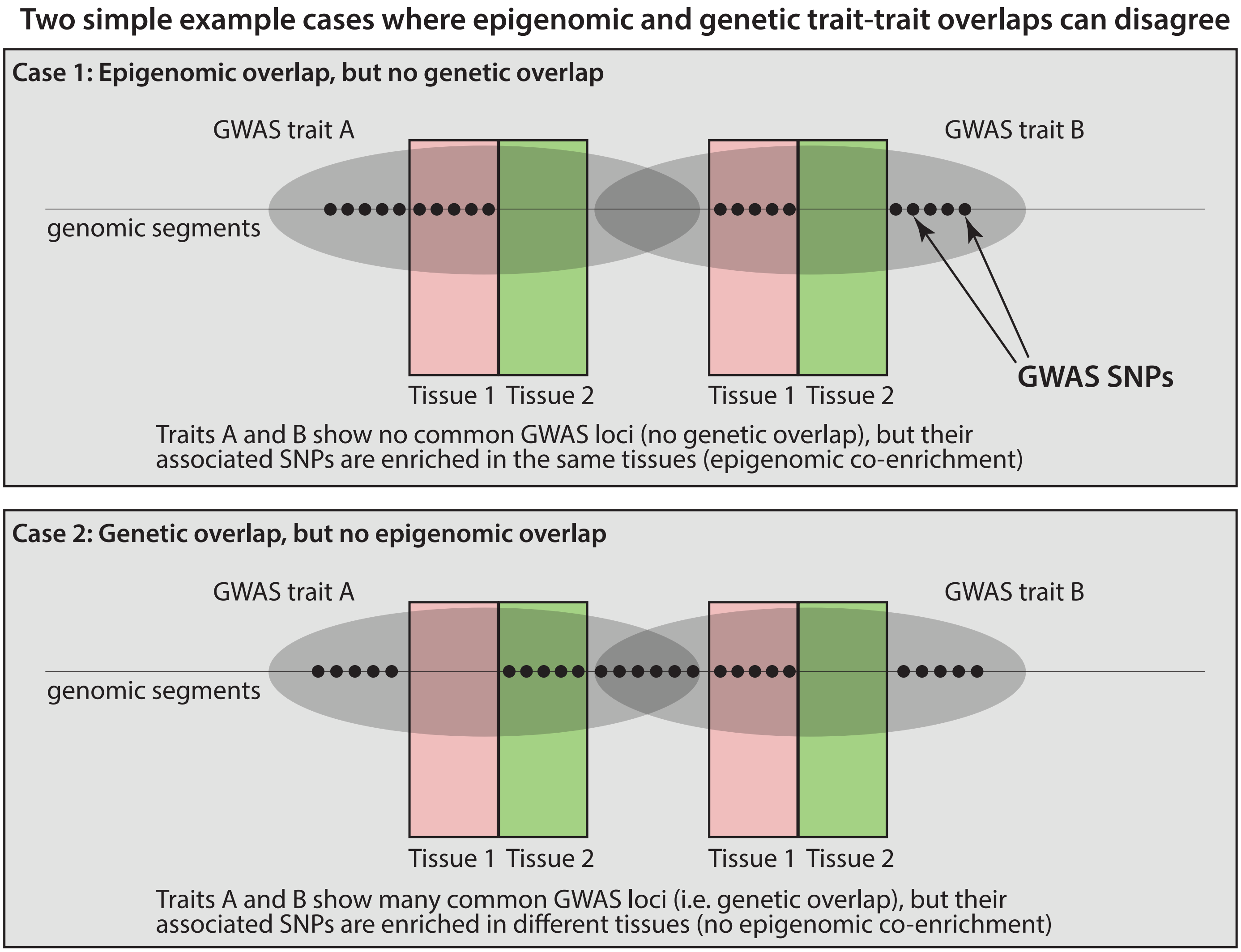

Figure S25: Two example cases where the epigenetic and the genetic trait-trait links may not agree.

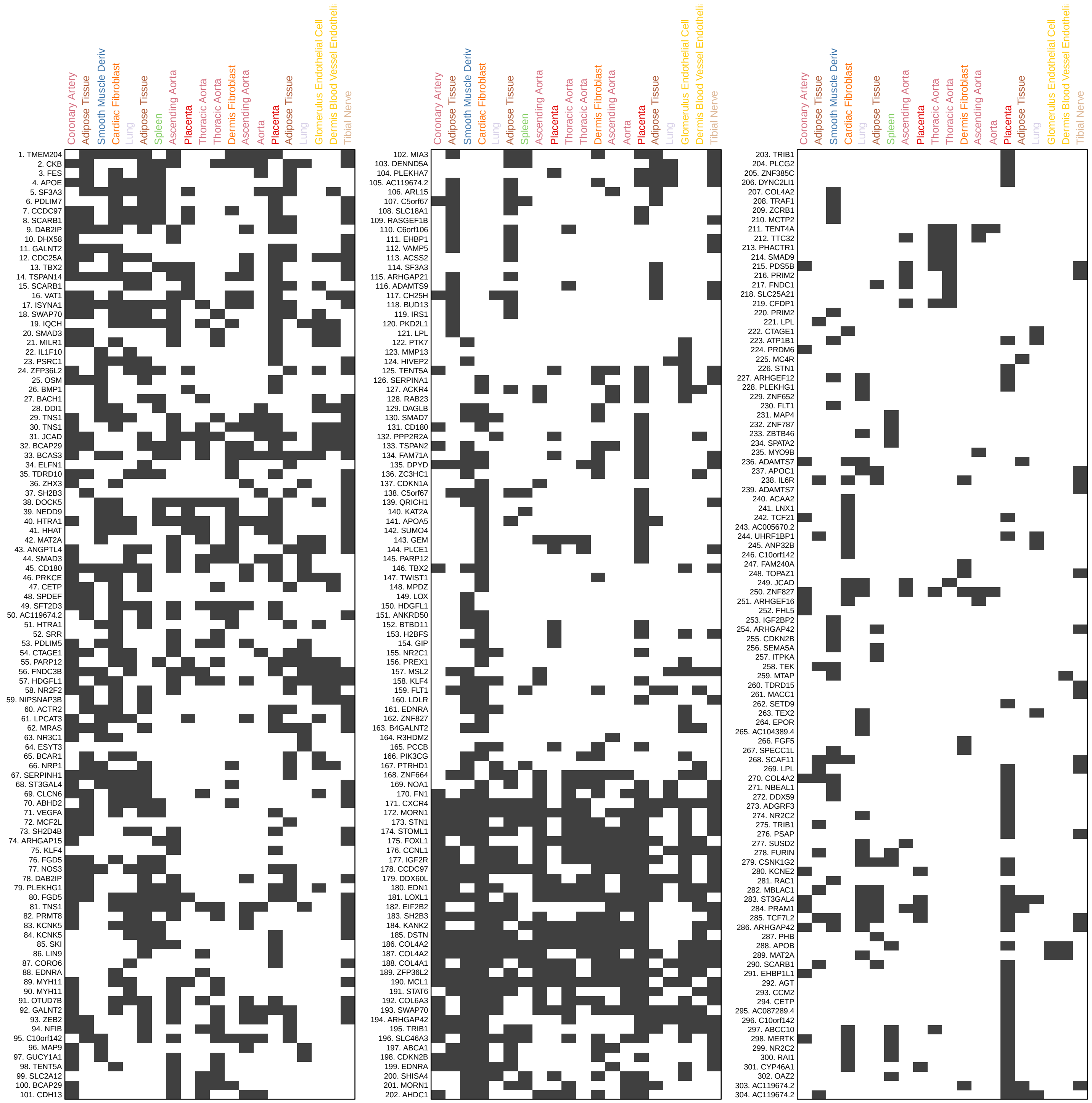

Figure S26: Loci of lead SNPs for CAD in top 20 significantly enriched nodes. Matrix is split in three and shows 304 SNPs against 20 nodes, by presence (black) or absence (white) in the node's enhancers. Loci are ordered by clustering their jaccard similarity across nodes using the Ward method. Each SNP is annotated with its nearest protein-coding gene.

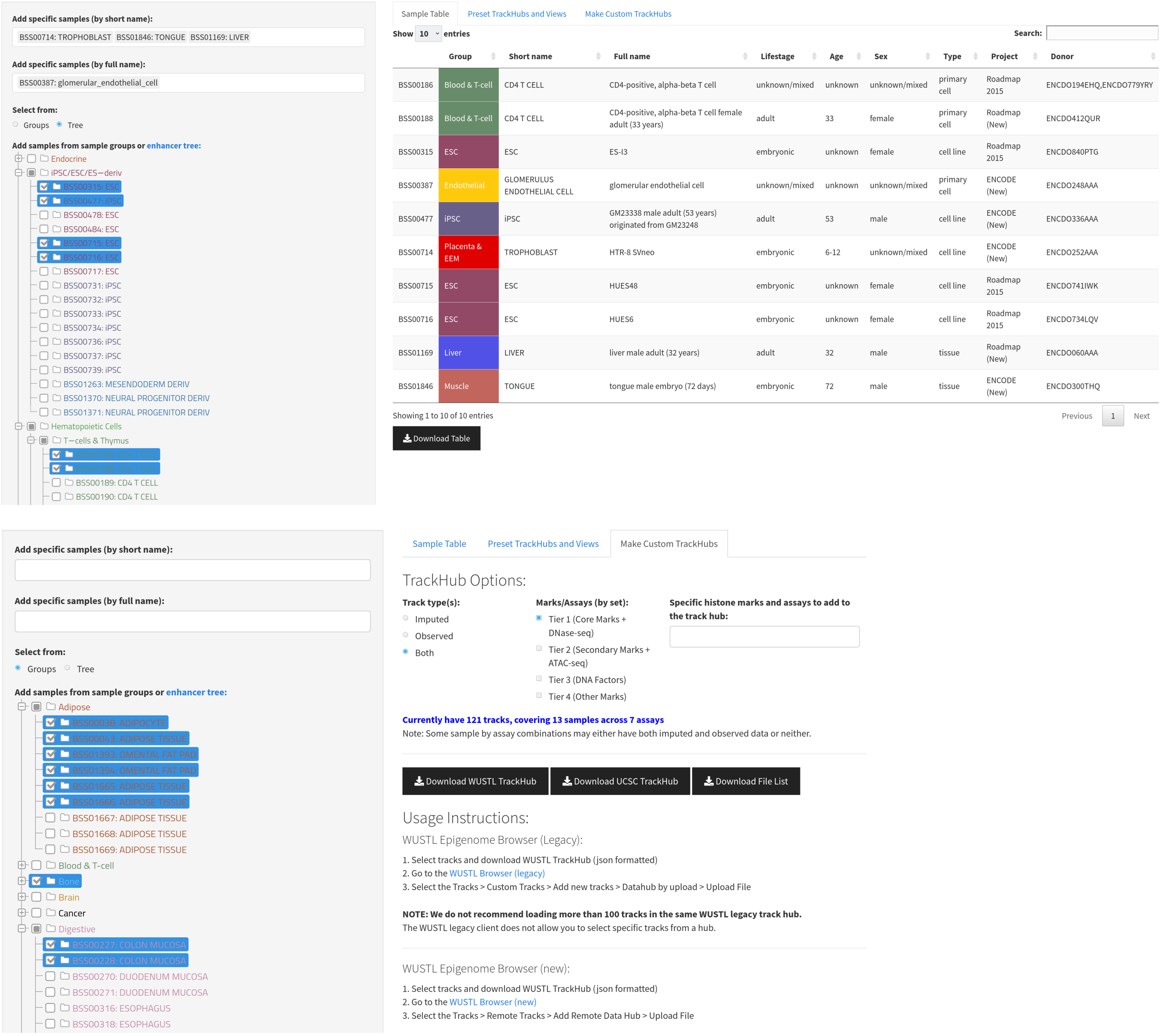

Figure S27: a. Example screenshot from interactive browser showing dataset selection by groups and by enhancer tree, metadata, and custom trackhub creation.

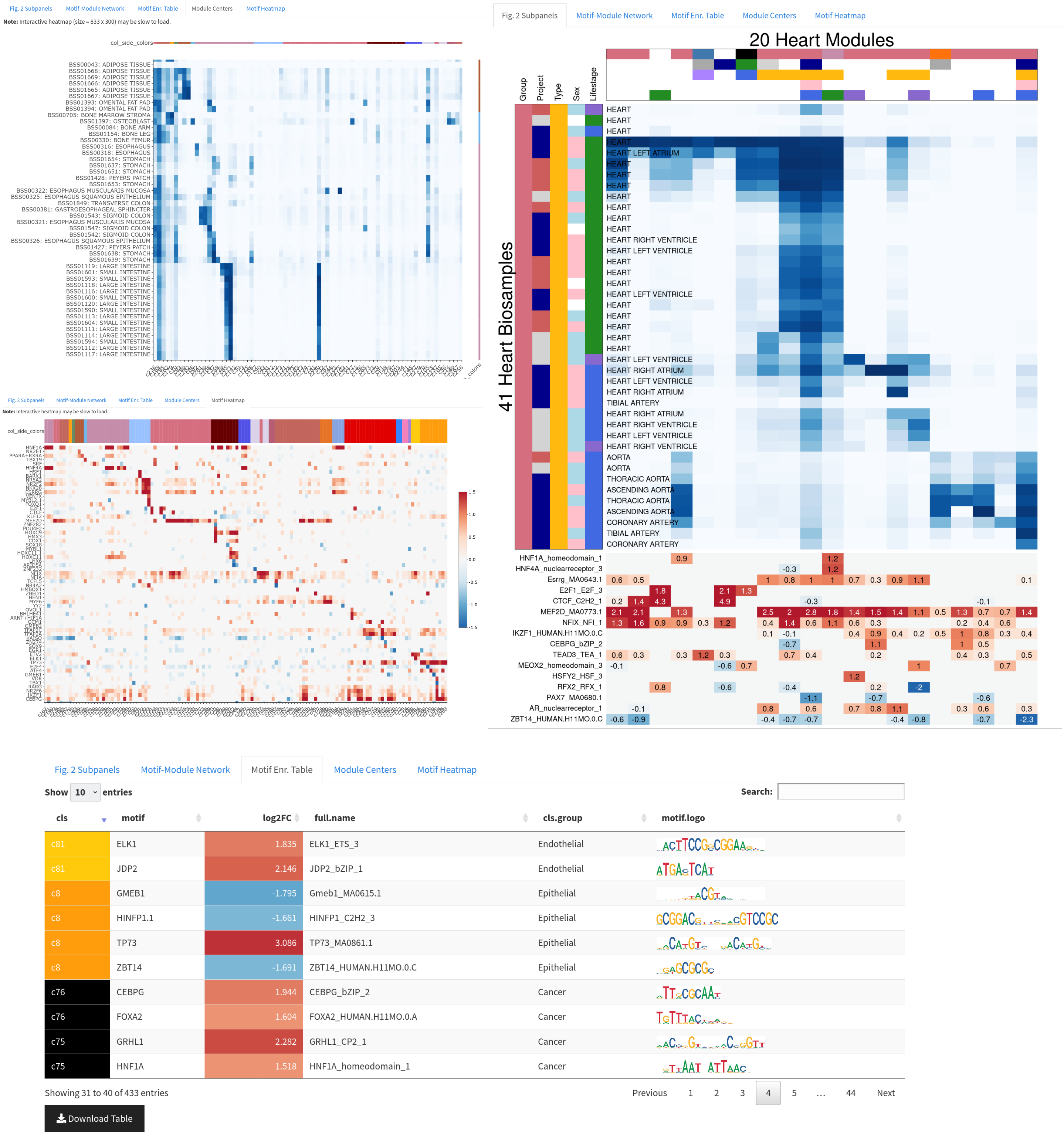

cls	motif	log2FC	full.name	cls.group	motif.logo
c81	ELK1	1.835	ELK1_ETS_3	Endothelial	
c81	JDP2	2.146	JDP2_bZIP_1	Endothelial	
c8	GMEB1	-1.795	Gmeb1_MA0615.1	Epithelial	
c8	HINFP1.1	-1.661	HINFP1_C2H2_3	Epithelial	
c8	TP73	3.086	TP73_MA0861.1	Epithelial	
c8	ZBT14	-1.691	ZBT14_HUMAN.H11MO.0.C	Epithelial	
c76	CEBPG	1.944	CEBPG_bZIP_2	Cancer	
c76	FOXA2	1.604	FOXA2_HUMAN.H11MO.0.A	Cancer	
c75	GRHL1	2.282	GRHL1_CP2_1	Cancer	
c75	HNF1A	1.518	HNF1A_homeodomain_1	Cancer	

Figure S27: b. Example screenshots from interactive browser showing exploration of modules and motifs, subpanels of Figure 2 for heart, and motif enrichments as a table.

Fig. 2 Subpanels | Motif-Module Network | Motif Enr. Table | Module Centers | Motif Heatmap

Note: Select nodes to see edge and node descriptions below the network.

Select by id

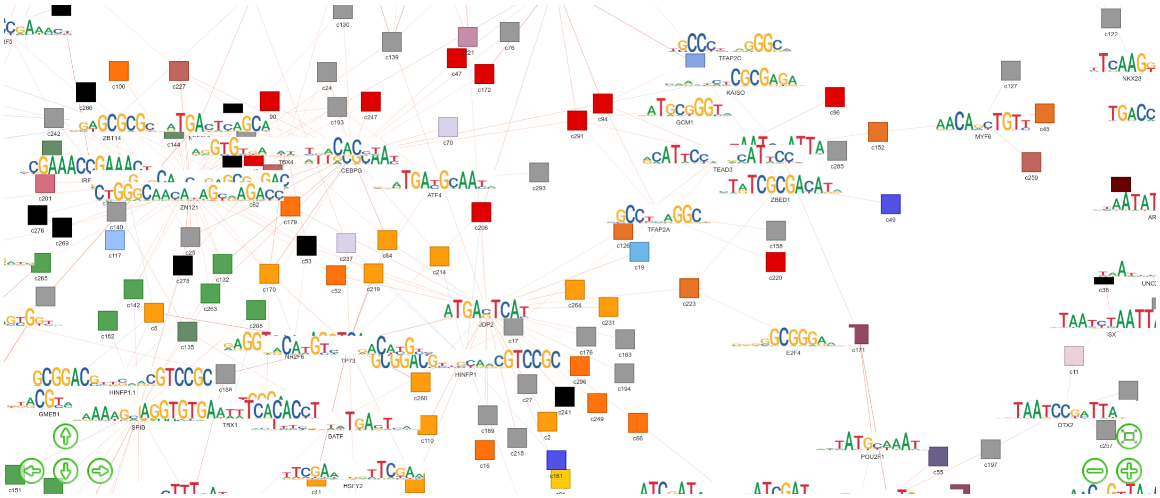

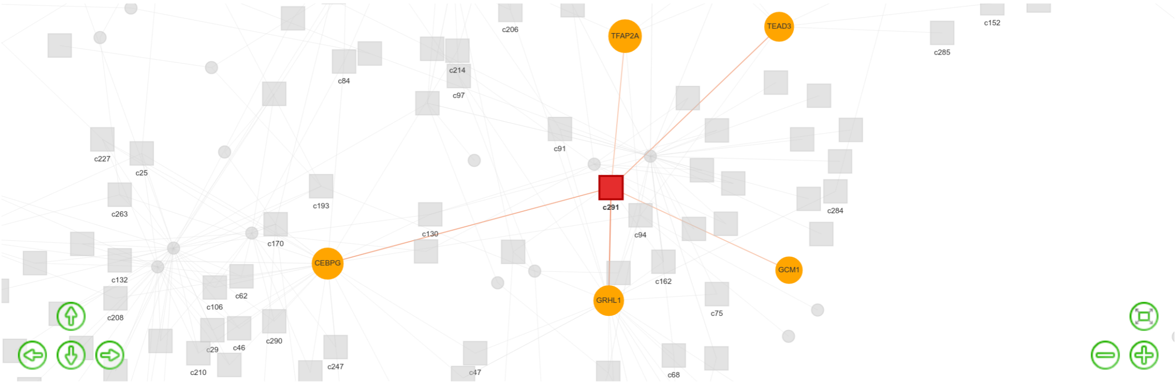

Network edges (for selected node):

Show 10 entries | Search:

cls	motif	log2FC	full.name	cls.group	motif.logo
c291	GRHL1	2.414	GRHL1_CP2_1	Placenta/EEM	
c291	CEBPG	1.913	CEBPG_bZIP_2	Placenta/EEM	
c291	TEAD3	1.814	TEAD3_TEA_1	Placenta/EEM	
c291	TFAP2A	1.552	TFAP2A_AP2_4	Placenta/EEM	
c291	GCM1	1.547	GCM1_GCM_1	Placenta/EEM	

Showing 1 to 5 of 5 entries — Previous 1 Next

Node summary (samples in cluster/motif logo):

Show 10 entries | Search:

cls	id	inclusion	name	group	type	lifestage
c291	BSS01852	0.91	TROPHOBLAST	Placenta/EEM	primary cell	embryonic
c291	BSS01853	0.8	TROPHOBLAST	Placenta/EEM	primary cell	embryonic
c291	BSS01435	0.79	PLACENTA	Placenta/EEM	tissue	embryonic
c291	BSS01859	0.76	TROPHOBLAST	Placenta/EEM	tissue	embryonic
c291	BSS01436	0.55	PLACENTA	Placenta/EEM	tissue	embryonic
c291	BSS01860	0.4	TROPHOBLAST	Placenta/EEM	tissue	embryonic
c291	BSS01440	0.37	PLACENTA	Placenta/EEM	tissue	embryonic

Figure S27: c. Example screenshots from interactive browser showing motif-module network and selection of specific module nodes to show enriched motifs and all included samples in the module.

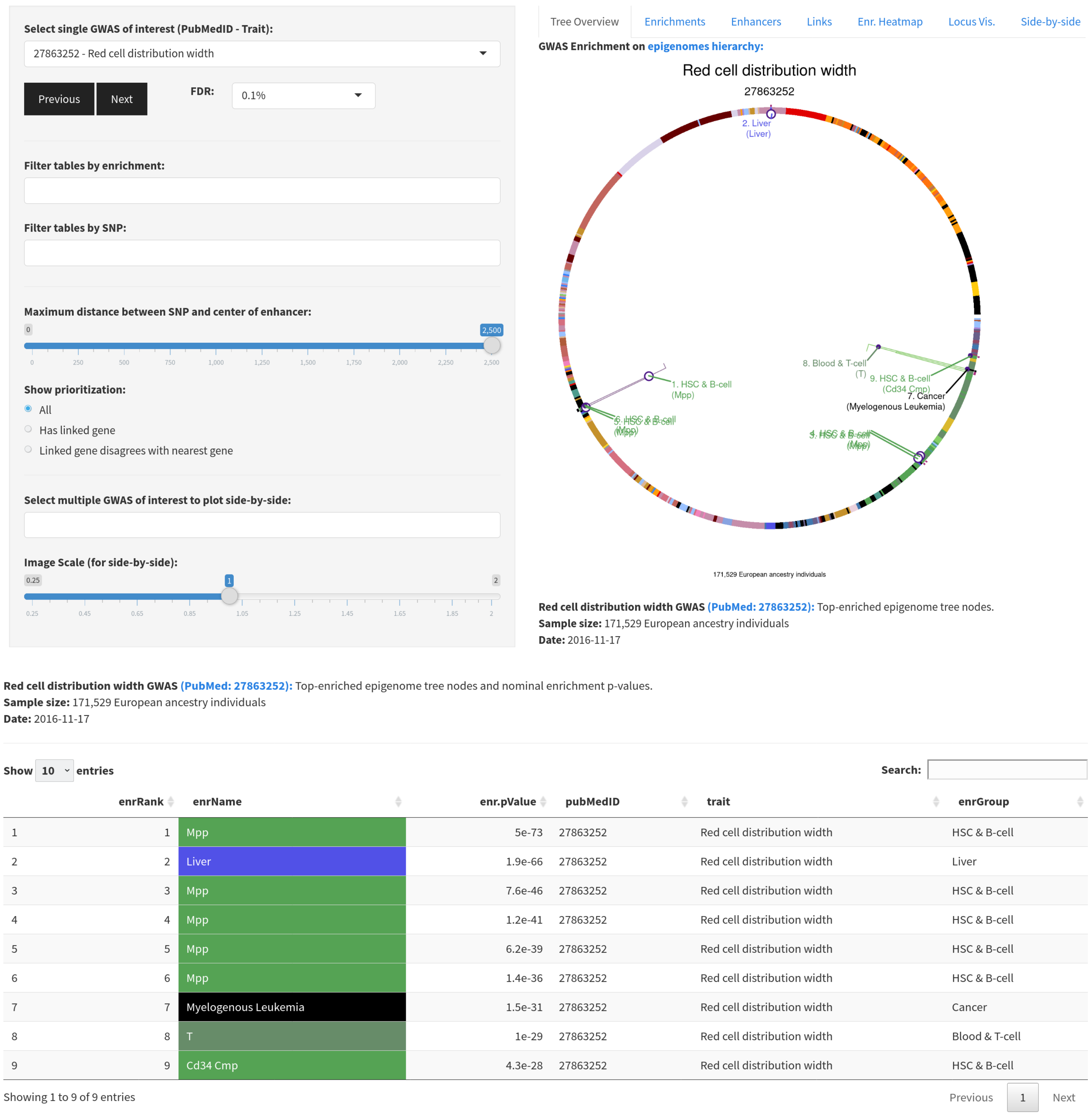

Figure S27: d. Example screenshots from interactive browser showing GWAS browsing and enrichments in figure and table view.

Tissue-specific enhancers near GWAS lead SNPs: All enhancers within 2.5kb of a GWAS lead SNP that are also active in one of the top-enriched tree nodes in the GWAS.

Show 10 entries   Search:

	chr	snpPos	snp.pValue	enhStart	enhEnd	distToCenter	nearestGene	enrRank	enrName	enr.pValue	enrGroup
1	chr1	43428757	1e-16	43429771	43430151	1204.5	SLC2A1	9	Cd34 Cmp	4.3e-28	HSC & B-cell
2	chr2	60725451	8e-21	60727115	60727295	1754.5	BCL11A	9	Cd34 Cmp	4.3e-28	HSC & B-cell
3	chr8	26243136	9e-19	26243356	26243576	330.5	BNIP3L	9	Cd34 Cmp	4.3e-28	HSC & B-cell
4	chr1	43428757	1e-16	43429051	43429407	472.5	SLC2A1	9	Cd34 Cmp	4.3e-28	HSC & B-cell
5	chr11	205198	4e-24	202620	202880	2447.5	BET1L	9	Cd34 Cmp	4.3e-28	HSC & B-cell
6	chr1	3691998	1e-17	3691784	3692024	93.5	SMIM1	9	Cd34 Cmp	4.3e-28	HSC & B-cell
7	chr11	44594611	1e-21	44595064	44595290	566.5	CD82	9	Cd34 Cmp	4.3e-28	HSC & B-cell
8	chr8	26243136	9e-19	26244376	26244596	1350.5	BNIP3L	9	Cd34 Cmp	4.3e-28	HSC & B-cell
9	chr19	1014538	1e-12	1016279	1016819	2011.5	TMEM259	9	Cd34 Cmp	4.3e-28	HSC & B-cell
10	chr1	203281175	6e-14	203279688	203279808	1426.5	BTG2	9	Cd34 Cmp	4.3e-28	HSC & B-cell

Showing 1 to 10 of 9,039 entries   Previous 1 2 3 4 5 … 904 Next

Download Enhancers

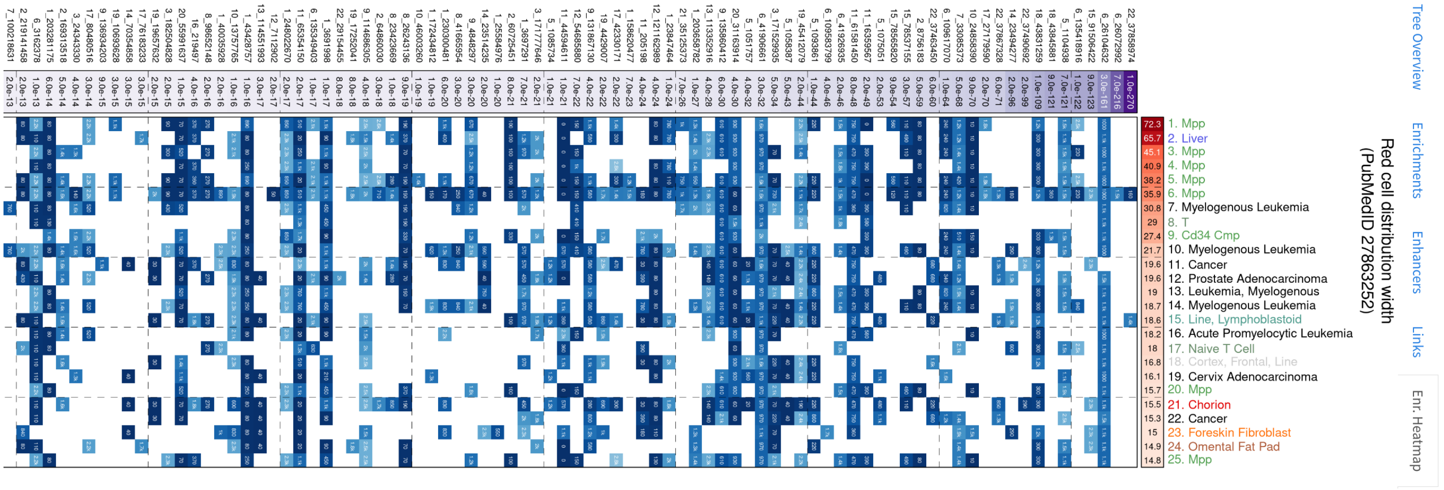

Figure S27: e. Example screenshots from interactive browser showing enhancer predictions nearby GWAS SNPs and enhancer versus subtree intersection, showing the closest enhancer to the SNP in each tissue.

Tree Overview | Enrichments | Enhancers | Links | Enr. Heatmap | Locus Vis. | Side-by-side

Table of gene-enhancer links: Gene-enhancer links in the GWAS loci (SNPs +/- 1Mb), reported for the top-enriched sample groups in the GWAS.

Show 10 entries

Search:

	chr	snpPos	snp.pValue	distToCenter	nearestGene	linkedGene	linkScore	linkDist	enrRank	enrName	enr.pValue	enrGroup
6774	chr6	26104632	3e-161	1464	HIST1H4C	HFE	0.89	15658.5	1	Mpp	5e-73	HSC & B-cell
6769	chr6	26104632	3e-161	1621	HIST1H4C	HFE	0.87	15501.5	1	Mpp	5e-73	HSC & B-cell
6764	chr6	26104632	3e-161	996.5	HIST1H4C	HIST1H1T	0.36	-2736	1	Mpp	5e-73	HSC & B-cell
2563	chr6	26104632	3e-161	1199	HIST1H4C	HFE	0.84	15923.5	2	Liver	1.9e-66	Liver
2575	chr6	26104632	3e-161	1060.5	HIST1H4C	HFE	0.84	16062	2	Liver	1.9e-66	Liver
2759	chr6	26104632	3e-161	1621	HIST1H4C	HFE	0.9	15501.5	3	Mpp	7.6e-46	HSC & B-cell
2954	chr6	26104632	3e-161	1199	HIST1H4C	HFE	0.9	15923.5	3	Mpp	7.6e-46	HSC & B-cell
3149	chr6	26104632	3e-161	1464	HIST1H4C	HFE	0.89	15658.5	3	Mpp	7.6e-46	HSC & B-cell
2804	chr6	26104632	3e-161	1106.5	HIST1H4C	HIST1H1T	0.81	-2626	3	Mpp	7.6e-46	HSC & B-cell
3144	chr6	26104632	3e-161	996.5	HIST1H4C	SLC17A2	0.8	174674	3	Mpp	7.6e-46	HSC & B-cell

Showing 1 to 10 of 1,459 entries

Previous 1 2 3 4 5 … 146 Next

Download Links

Select locus:

chr6:135,418,916 (p=1.0e-122, HBS1L)

Previous | Next

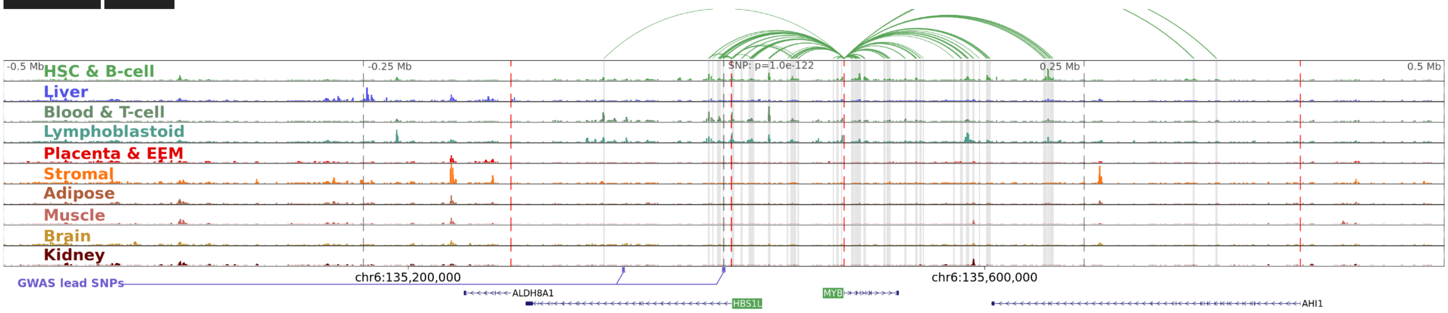

Locus overview for 1Mb around selected lead SNP: Tracks show average H3K27ac signal of enhancers in locus, and red dashed lines indicate the TSSes of nearby genes. Two types of correlation-based links are plotted: **(1)** Links from one of the enhancers near a lead SNP in the enriched epigenomes. **(2)** Any links in the locus present in at least half of the samples in one of the top sample groups (**HSC & B-cell**). Genes linked to an enhancer within 2.5kb of a GWAS lead SNP are highlighted and colored according to the sample group with the highest link score. Link data and images for this GWAS are also available from our **data repository** .
Click to enable/disable zoom on locus, scroll to change zoom size.

Figure S27: f. Example screenshots from interactive browser showing linking predictions on GWAS SNPs and locus visualization with links, enhancers, and prioritized genes.
