# Supplementary material for: Regulatory genomic circuitry of human disease loci by integrative epigenomics
Source: Nature. 2021 Feb 3;590(7845):300–7. doi: 10.1038/s41586-020-03145-z (PMC7875769; doi:10.1038/s41586-020-03145-z)
Supplement: Supplementary file 3 — This file contains Supplementary Notes which include additional text on imputation validation and comparison between observed and imputed data. [file 41586_2020_3145_MOESM3_ESM.pdf]

Regulatory genomic circuitry of human disease loci by integrative epigenomics

Carles A. Boix[1-3], Benjamin T. James[1,2], Yongjin P. Park[1,2,4], Wouter Meuleman[5], Manolis Kellis[1,2]*

1. Computer Science and Artificial Intelligence Laboratory, Massachusetts Institute of Technology, Cambridge, MA, USA. 2. Broad Institute of MIT and Harvard, Cambridge, MA, USA. 3. Computational and Systems Biology Program, Massachusetts Institute of Technology, Cambridge, MA, USA. 4. Department of Pathology and Laboratory Medicine, Statistics, University of British Columbia, Vancouver, BC, Canada. 5. Altius Institute for Biomedical Sciences, United States. Correspondence to: e-mail: manoli@mit.edu

Supplementary Notes **1**
- Imputation quality by imputation support 1
- Imputation validation across external tracks 1
- Imputation quality in rare events and DHSs 2
- Comparison of statistical properties of imputed and observed data 3
- Recovery of modules in observed-only data 4
- Comparison with SCREEN elements 5

Supplementary Notes

Imputation quality by imputation support

First, we evaluated how imputation quality differs in samples with varying numbers of informant same-sample datasets (**Supp. Fig. S3a-e**), from only 1 to 10+, for all our global metrics, AucImp1 (AUROC for predicting the top 1% of imputed 25bp bins from observed), AucObs1 (AUROC for predicting the top 1% of observed 25bp bins from imputed), Catch1imp (% of top 1% imputed in top 5% observed bins), Catch1obs (% of top 1% observed in top 5% imputed bins), GWCorr (genome-wide Pearson Correlation), and Match1 (% of top 1% observed peaks matching top 1% imputed peaks). We find that overall imputation quality does not seem to suffer greatly from having few supporting datasets in the sample. Importantly, the Catch1imp and Catch1obs metrics, which measure peak recovery, are quite stable (**Supp. Fig. S3a**).

Second, we find that these metrics track much better with the average similarity of the 10 closest samples and whether the imputed assay is broad or punctate than with the number of same-sample datasets (**Supp. Fig. S3b,c**). We aggregated metric scores by dataset support across four bins of average similarity to closest samples and find that sample support more strongly affects overall imputation quality when the average similarity of the closest samples is low (**Supp. Fig. S3d**). In this regime, it is important to have many good within-sample features, but when the average similarity to close-samples is high, the number of within-sample supporting datasets has a much smaller impact, in particular on metrics of peak recovery (**Supp. Fig. S3d**).

Third, we plotted the average similarity to closest samples both for all datasets and for only datasets that are based on only one supporting experiment (orange, primarily DNase-seq) (**Supp. Fig. S3e**). In particular for DNase-seq, we find that even in the worst case, the average similarity of these tracks to experiments within the dense area of the experiment matrix is quite high (between 0.5 and 0.8), suggesting that there are enough datasets to back up imputation of samples with only DNase-seq.

Imputation validation across external tracks

In addition, we used 51 novel experimental tracks across 8 marks and assays from the ENCODE Imputation Challenge project to corroborate imputation performance for 14 of these biosamples. External datasets were processed by subsampling histone marks to 30M reads and DNase-seq and ATAC-seq to 50M reads and predicting a -log10p-value signal track relative to appropriate input control data, as was done for our observed data. However, external tracks were aligned to hg38 rather than hg19. We re-mapped the tracks to hg19 by averaging tracks both at the 200bp level, using liftOver, removing all non-fully-mapping regions and cross-chromosome maps, and binning the resulting tracks at the 200bp level for evaluation.

We evaluated genome-wide imputation performance metrics (AUPRC, AUROC predicting top 1% of observed data and peak recovery of top 1% Imputed or Observed with top 5% Observed or Imputed, respectively) on fully mapped 200bp bins (90.1%) of chr1 of the external observed tracks against either the appropriate imputed track, the best-matching of the other observed tracks, or the observed signal average (**Extended Data Fig. 1b**).

For punctate assays, imputed data heavily outperformed the nearest samples, with 41% higher average

precision (AP) on average, as well as the assay means (89% higher AP) in the prediction of external validation data. Imputed data showed much more modest improvement over other predictors for broad marks (10% better AP than nearest, 6% better than mean).

It is important to note that we cannot know the best “nearest” sample to the experimental data until we do the experiment. In practice, the nearest sample can never be better than the nearest sample we show here, knowing what the experimental data looks like, and it may often be worse. Therefore, our imputations will outperform the nearest sample by more in practice, except in cases of very close replicates.

Overall, imputed predictions best predicted the external validation data in almost all cases in punctate marks, with a higher average precision (AP) than the nearest observed track 96% of the time and higher than the mean 100% of the time (**Extended Data Fig. 1c**). Imputed performance in broad marks (H3K27me3, H3K36me3, H3K9me3) also beat the nearest sample (76.9%) and the mean (73.1%) the large majority of the time.

Imputation quality in rare events and DHSs

We performed additional validation to investigate the performance of imputed data with respect to rare events and the quantification of false positives. We calculated the average precision for recovering the top 1% of locations in the observed data in chromosome 19 (**Extended Data Fig. 1d**). For each observed track, we compared the average precision of the imputed track against that of the nearest (most predictive) observed track or of the mean of observed datasets. In almost all cases, the imputed track outperformed both of the other datasets. The only exception was for DNase-seq, where we have 643 observed datasets, and 77% of the nearest matches outperforming imputed data were near-replicate experiments. For comparison, the AUROC of the exact same task also shows that imputed data out-performs the other two metrics, except in the case of H4K20me1 (36 observed datasets), where the mean is comparable to the imputed track (**Extended Data Fig. 1d**).

Overall, we find that the imputed track has higher average precision than the nearest observed dataset in 83.8% of cases (21.2% AP higher on average) and beats the mean of the observed data in 96.4% of cases (30.8% higher AP on average, **Extended Data Fig. 1e**). To understand where imputation does not surpass nearby samples, we colored points by their sample group and highlighted cases where the nearest sample or the mean heavily outperformed the imputation, labeling points with over 25% (nearest subpanel) or 10% (mean subpanel) greater average precision than the imputed track. In the large majority of cases, the nearest sample was a near-replicate of the exact same tissue type (where labels on the left subpanel match each other). In cases where the nearest sample was not a near-replicate, the mean observed data also outperformed the imputed track, suggesting that there might have been an issue with the observed track label rather than with the imputation itself.

To specifically estimate the false discovery rate in H3K27ac in enhancer DHSs, which we use to filter active enhancer states, we asked what percentage of the 2M DHSs with imputed H3K27ac above a certain cutoff are also in the top 10%, 5%, 2.5%, 1%, and 0.1% of 3.6M DHSs by the matched observed datasets (**Extended Data Fig. 1f**). The majority of imputed DHSs crossing all cutoffs are in the top 10% of observed DHSs (~200k) for their matched sample (for cutoffs of 2, 4, and 10: 69.3%, 85.0%, and 94.5% on average) and in the top 5% observed DHSs (~100k, with 51.3%, 74.2, and 91.2% on average).

To analyze the effect of DHSs on false positives, we carried out the same false positive-geared analysis from before for H3K27ac in all enhancer states but in a DHS-agnostic manner. In particular, we calculated and compared the quantile-recovery analysis of 294k possible enhancer 200bp bins in chr19 as compared to the same analysis in 3.5M DHSs across the genome (**Extended Data Fig. 1f, blue boxplots**).

We find that the imputed H3K27ac data outside of the defined DHS set fall into top observed quantiles at about the same rate as imputed data in the DHS list (**Extended Data Fig. 1f, red boxplots**). Due to different data distribution in the DHSs and outside DHSs, the 99.9% quantile cutoffs for the observed data in the DHS-agnostic analysis were higher than in the DHS analysis, leading to lower recovery percentages, while the 95% and 97.5% quantiles were lower and showed higher recovery.

Overall, our results indicate that while including DHSs as part of our active enhancer definition improves our biological specificity, it does not necessarily cut down on false positives generated from imputation, as we

obtain comparable results in both DHS-centric and DHS-agnostic analyses of imputed data specificity.

We expect that rarer, highly specific peaks are less likely to replicate properly in the imputed data than constitutively active locations. To see the extent of this effect, we partitioned the DHSs by the number of samples in which each DHS is called as an active enhancer in our later analyses. We then calculated and plot these capture statistics along the continuum of rare to constitutive enhancer calls (**Extended Data Fig. 1g**). We find that the above estimates are quite stable in DHSs which are supported across at least a few samples (~5+, or greater than 0.5% of samples), but is about 20-30% lower in DHSs with support in only a couple of samples. However, evaluating recovery of rare events can be complicated by the fact that many of these events in observed data are not due to biological signal but rather reflect noise in the observed dataset.

We matched each observed dataset to its “nearest” observed dataset, noting that such a predictor is unattainable in a real-world situation, because we can’t know what the nearest sample of dataset X will be, without actually observing dataset X, i.e. doing the experiments to generate dataset X in the first place. We computed this “nearest” observed based on AUPRC for predicting the top 1% of observed bins genome-wide (37% are near-replicates, with the same short sample name).

We then repeated the same quantile recovery analysis to compare performance of imputed tracks compared to the performance of nearest tracks (in red). We found that the imputed track outperformed the nearest track in the majority of cases, but most importantly, imputation outperformed the nearest track in the case of rare or sample-specific enhancers, indicating that even if the “nearest” track was knowable, imputed tracks actually performed better. Specifically, showing fewer false positive peak calls for peaks that were active in fewer than 200 samples, across all signal cutoffs and for all quantiles (**Extended Data Fig. 1g**).

However, the nearest observed dataset did a better job of recovering constitutive enhancer events. This may be a function of the different dynamic ranges of observed and imputed data. Finally, when we look at very high confidence peaks (signal > 10), the imputed data outperforms the nearest possible (and often near-replicate) observed data across all regimes.

Comparison of statistical properties of imputed and observed data

To quantify the effect of various covariates on dataset similarity, we computed the pairwise genome-wide correlations between all datasets with both observed and imputed tracks. We z-scored the correlations per-mark separately within observed and imputed data to account for differences in the distribution of similarity scores across marks and datatypes. We then created indicator variables to account for whether each pair of samples came from the same year, project, or lab, had the same read length or read type (paired vs. single-ended), or had the same biological attributes (lifestage, sample type, or sample group). We estimated the effect of sharing specific covariates on the z-scored similarity of a pair of datasets in either imputed or observed data, using linear regressions to calculate effect sizes (**Supp. Fig. S9c**).

We find that most unwanted batch and technical covariates - read length, project, read type, and year of experiment - show a decreased effect on pairwise similarity in imputed data relative to observed data. On the other hand, biological covariates - sample group, lifestage, and type - increase the pairwise similarity of two samples in imputed data over observed data. Lab of origin was correlated with increased pairwise similarity in imputed datasets but its effect may be confounded by strong correlation with the types of biosamples studied in each lab. Overall, imputed data shows much stronger concordance with biological attributes than observed data (10.2% vs. 2% variance explained by biological covariates), while not showing a differential technical effect (4.2% vs. 4.0% explained from technical covariates, or 3.3% vs. 4.0% without the lab covariate).

We next investigated how imputation affects dataset homogeneity. In exploratory analyses, we calculated a UMAP for combined observed + imputed data and showed that the imputed data captured the same variability as observed data, and that the imputed data clustered with the observed data (**Extended Data Fig. 2b**).

However, the ratio of observed to imputed data is not balanced in each mark, which might affect subsequent analysis. To specifically analyze homogeneity in imputed and observed data, we analyzed the subset of samples present in both imputed and observed datasets (**Supp. Fig. S10**). We calculated the Spearman correlation of all pairs of datasets in each mark, which we plot below, ordered by their sample group. Qualitatively, we see that observed data fails to capture group patterns that imputed data makes explicit (**Supp. Fig. S10a**).

We also computed UMAP dimensionality reductions for each of the marks using all imputed and observed data together, colored by either group or data type (**Supp. Fig. S10b,c**). We find that cluster groupings hold up in all marks, and that there is no clear separation between imputed and observed data in the large majority of cases.

To more quantitatively assess homogeneity, we also built an observed and an imputed sample-sample graph for each mark, linking each sample to the top tenth of nearest neighbors by highest genome-wide Pearson correlation (for consistency in different marks with more or less samples). We calculated the clustering coefficients, defined as the number of connected triplets over the number of possible triplets, for each mark for both the imputed and observed graphs (**Supp. Fig. S10d,e**). We find that imputed data is more homogeneous for all marks, but that this homogeneity is only pronounced in H3K36me3 and H4K20me1. These are broad, independent marks that can be hard to predict from other marks and assays, so the majority of the information used to generate the predictions comes from the same mark across samples, increasing homogeneity.

We also computed dataset homogeneity in terms of the dataset sample groups in two ways. First, we calculated the ratio of average genome-wide correlation between datasets within the same group versus between groups. Plotting the within/between ratios for each mark and sample group combination in observed and imputed datasets, we see that there is a very close correspondence between imputed and observed ratios ($R=0.71$, $p << 0.01$, **Supp. Fig. S10f**), but that the observed data shows much higher variability, showing very high within-group homogeneity for some cases, and very low concordance for others.

In particular, multiple sample groups show low within-group concordance in observed data for the H3K27me3, H3K36me3, and H4K20me1 marks, including ostensibly homogeneous groups such as Spleen, ESC, PNS, and Endocrine (as opposed to larger groups such as Blood & T-cell).

We also used the genome-wide correlation between each pair of datasets of the same mark to predict whether they were in the same sample group (**Supp. Fig. S10g**). Across all marks, imputed data shows higher classification AUC scores, demonstrating that cross-sample comparisons are more consistent with biological groups in imputed than observed data.

Recovery of modules in observed-only data

We originally defined the broad vs. specific modules by defining active samples in a module as samples where at least 25% of the module's enhancers were active. In order to confirm module properties, we re-analyzed the cluster modules in the context of only observed data. In particular, we selected 235 samples out of the 833 where we have non-flagged H3K27ac observed data. We then compared the number of active samples in each module in the overall dataset and in the observed-only data.

Using this observed data, we plotted the number of observed vs. all samples in each of the 300 clusters (left), and the Gini inequality coefficients for the sample inclusion fractions for each module (right), where broad modules (red points) were defined as having at least 150 active samples in the full data (dashed red line) (**Supp. Fig. S14a).**

In the 290 specific clusters (blue), for 46 modules (15.8%), no samples have over 25% of enhancers active, because the modules' active samples did not have observed data available for H3K27ac. However, despite the different sample composition of observed vs. full data, the fraction of active samples is quite consistent between the datasets (left, $r^2=0.802$, right $r^2=0.82$).

For the 10 broad modules (red) from the full data, all were active in at least 9.7% of observed samples and 24% on average, seven were the most broadly active in terms of active samples in observed, and nine were in the bottom ten modules by Gini coefficient (least unequal, or most broadly expressed).

As a qualitative comparison, we also calculated the average activities of observed H3K27ac and H3K4me1 (as a proxy for active enhancer annotations in ChromHMM) for each enhancer module in the observed datasets in each of 205 datasets for which both marks are available as observed datasets. Comparison of these average activity measurements with cluster centers shows that the centers are very similar to the combined H3K27ac and H3K4me1 activities (**Supp. Fig. S14b**).

Finally, we computed the peak signal and Gini statistics for the joint H3K27ac and H3K4me1 raw signal in observed data, as opposed to our previous analysis using H3K27ac only in the enhancer states, and found that

the raw signals also showed high concordance in module definition with the full compendium (**Supp. Fig. S14c**).

Comparison with SCREEN elements

Overall, major differences between the EpiMap and SCREEN elements include:

1. SCREEN does not start from the same DHS dataset (2.2M clustered DHS), curates DNase-seq from 706 samples (rather than 733 as in the Index resource), and uses the DNase-seq signal in each biosample directly, whereas we use 3.6M sets of DHS coordinates from Index to define accessible regions, but not the DNase-seq signal for each biosample. DNase-seq signal data for these regions is available as a 3.6M x 833 biosample matrix on our website.
2. SCREEN only calls enhancer-like signatures (pELS and dELS) for those samples with H3K27ac experiments, whereas we uniformly incorporate H3K27ac signal in our enhancer calls.
3. SCREEN elements do not use enhancer annotations (which would typically incorporate H3K4me1), and we use annotations from ChromHMM, computed from observed and imputed data in six histone marks.
4. SCREEN specifically incorporates locations with H3K4me3 into their enhancer sets. While H3K4me3 plays a role in defining our ChromHMM annotations, our enhancers do not contain significant H3K4me3 signal, as this histone mark is typically considered promoter-associated.

Specifically, we mapped 98% of all ENCODE3 SCREEN elements to a DHS in the set of 3.6M DHSs (requiring an overlap of at least 100bp). We mapped each DHS uniquely to one SCREEN annotation, and calculated the overlap between our element annotations (into enhancers, promoters, and dyadic elements) and the SCREEN annotations.

Despite the differing DHS references, almost all SCREEN elements are contained within our annotations, with 84.5% of dELS (distal elements) corresponding to active enhancers, 93.5% of dELS mapped to one of our elements, and 98.5% of PLS and 97.6% of pELS (proximal) elements mapped to an EpiMap element (**Extended Data Fig. 10a**). DNase-H3K4me3 and CTCF-only elements were recovered much less frequently (51.8% and 36.6% of the time), but constituted less than 100k elements in total and were not explicitly defined in our analysis of genomic elements.

On the other hand, only 71.7% of promoters and 38.2% of EpiMap enhancers were recovered by the SCREEN dataset (**Extended Data Fig. 10b**).

At the level of epigenomes and modules, we find that 40-60% of enhancers in each of our epigenomes correspond to dELS, whereas modules see much more variable recovery (10-60%) (**Extended Data Fig. 10c**), both due to differences in captured biological space and in the bias towards recovery of more constitutively active elements (**Extended Data Fig. 10d**).

On a functional level, we find that subsetting modules to only the dELS + EpiMap enhancer intersection (672k locations, keeping the EpiMap activity matrices) gives slightly higher motif enrichments than when using all 2.1M EpiMap enhancers (+17.5% larger fold-changes for significant enrichments, **Extended Data Fig. 10e**). We suspect that the dELS + EpiMap intersection represents a more conservative and higher quality set than either set individually, but sacrifices a substantial number of elements (1.2M+ enhancers).

This tradeoff is evident on the GWAS side, where using our full set of enhancers captures 47% more GWAS lead SNPs within 2.5kb (81% vs. 55% of SNPs) and almost three times more SNPs within 100bp of an enhancer center (22% vs. 8%) (**Extended Data Fig. 10f-i**).
