# Supplementary material for: Regulatory genomic circuitry of human disease loci by integrative epigenomics
Source: Nature. 2021 Feb 3;590(7845):300–7. doi: 10.1038/s41586-020-03145-z (PMC7875769; doi:10.1038/s41586-020-03145-z)
Supplement: Supplementary file 4 — Summary GWAS enrichments for each trait on the hierarchical biosample tree. [file 41586_2020_3145_MOESM4_ESM.pdf]

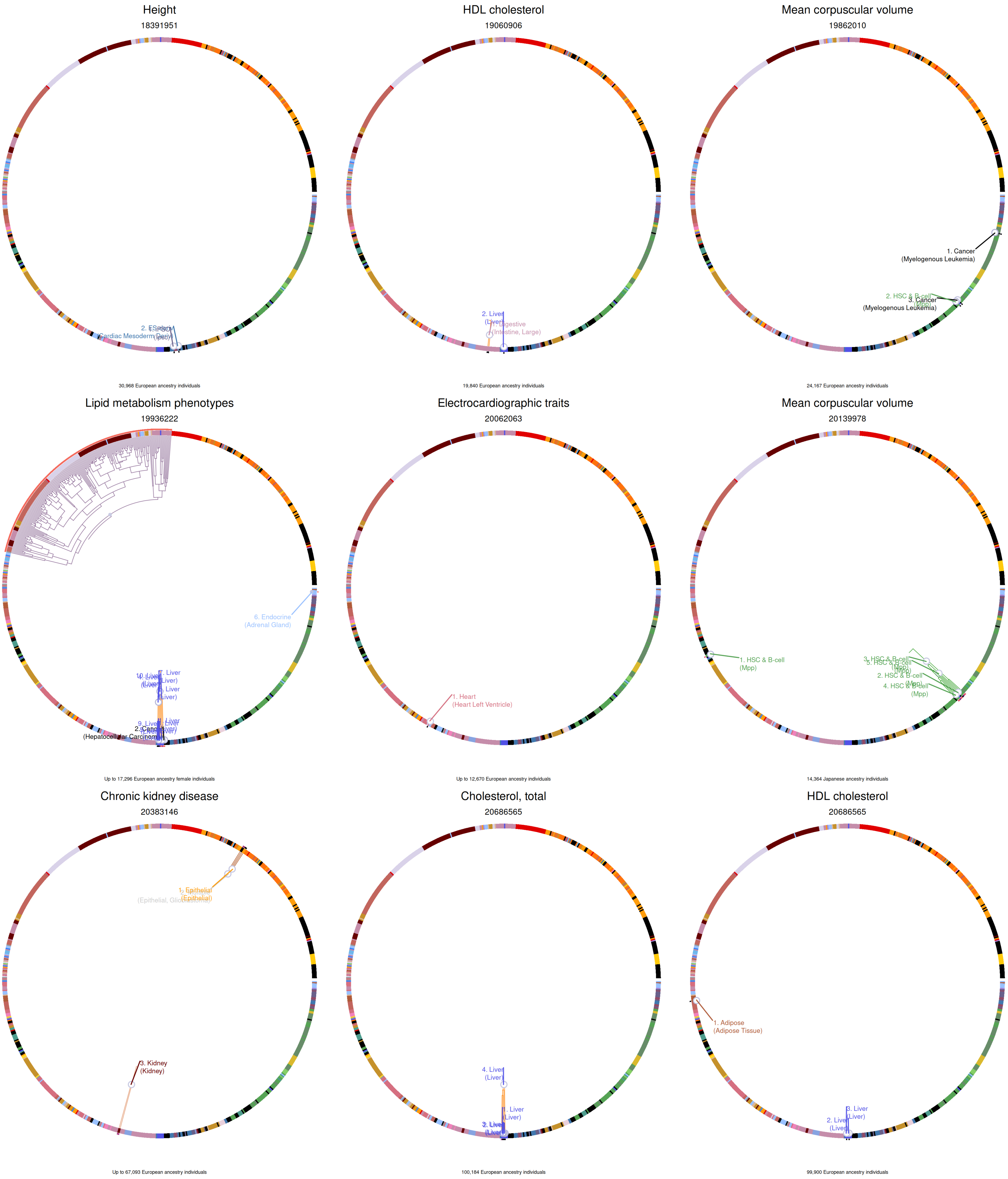

Height
18391951
Cardiac Mesoderm
30,968 European ancestry individuals
HDL cholesterol
19060906
2. Liver
(Liver)
(Intestine, Large)
19,840 European ancestry individuals
Mean corpuscular volume
19862010
1. Cancer
(Myelogenous Leukemia)
3. Cancer
(Myelogenous Leukemia)
24,167 European ancestry individuals
Lipid metabolism phenotypes
19936222
6. Endocrine
(Adrenal Gland)
(Hepatocellular Carcinoma)
Up to 17,296 European ancestry female individuals
Electrocardiographic traits
20062063
1. Heart
(Heart Left Ventricle)
Up to 12,670 European ancestry individuals
Mean corpuscular volume
20139978
1. HSC & B-cell
(Mpp)
2. HSC & B-cell
4. HSC & B-cell
(Mpp)
14,364 Japanese ancestry individuals
Chronic kidney disease
20383146
3. Kidney
(Kidney)
Up to 67,093 European ancestry individuals
Cholesterol, total
20686565
4. Liver
(Liver)
1. Liver
(Liver)
100,184 European ancestry individuals
HDL cholesterol
20686565
1. Adipose
(Adipose Tissue)
3. Liver
(Liver)
2. Liver
(Liver)
99,900 European ancestry individuals

LDL cholesterol

20686565

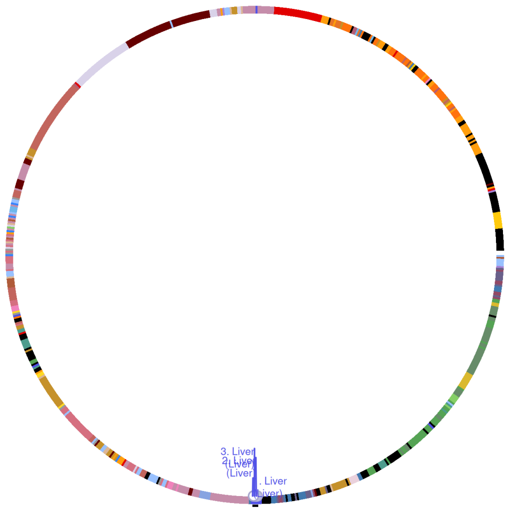

95,454 European ancestry individuals

HDL cholesterol

20864672

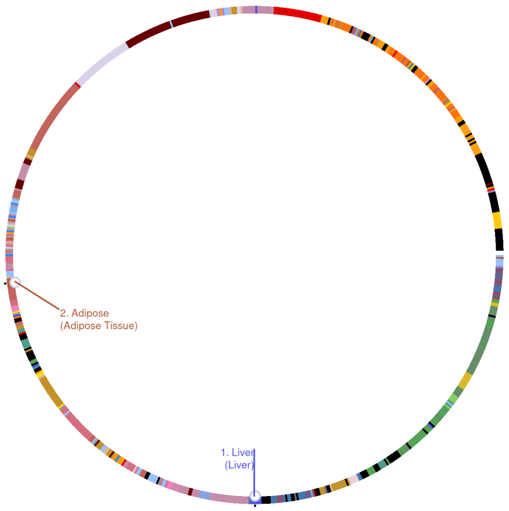

up to 17,723 European ancestry individuals

C-reactive protein levels

21300955

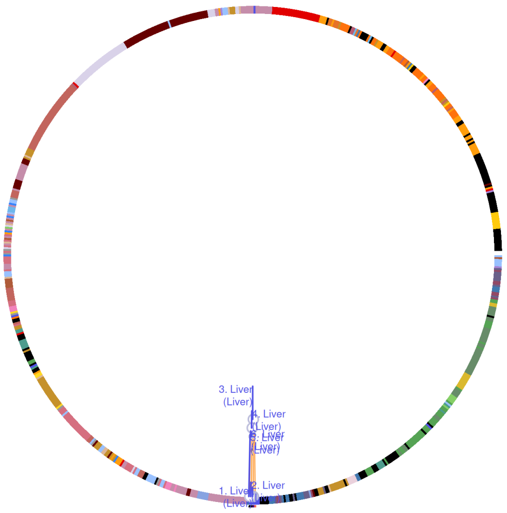

63,678 European ancestry individuals 1,792 Erasmus Rucphen individuals, 715 Orcadian individuals

Celiac disease or Rheumatoid arthritis

21383967

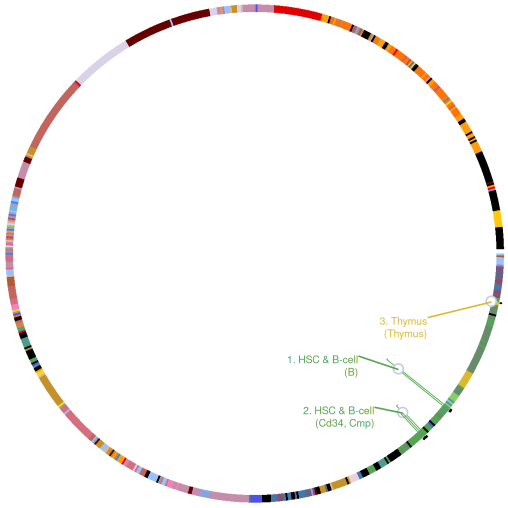

4,533 European ancestry celiac disease cases, 5,539 European ancestry rheumatoid arthritis cases, 27,981 European ancestry controls

Proinsulin levels

21873549

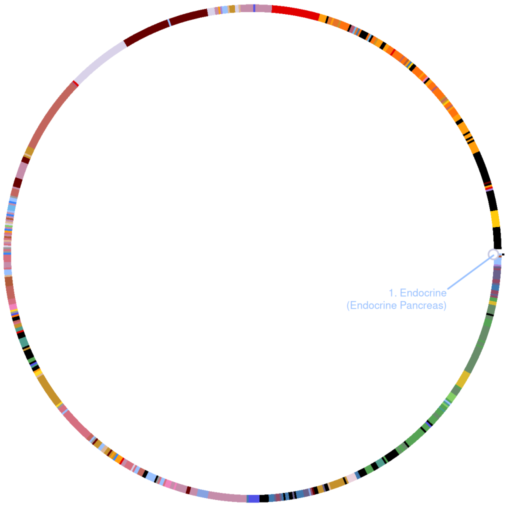

10,701 European ancestry individuals

Blood pressure

21909110

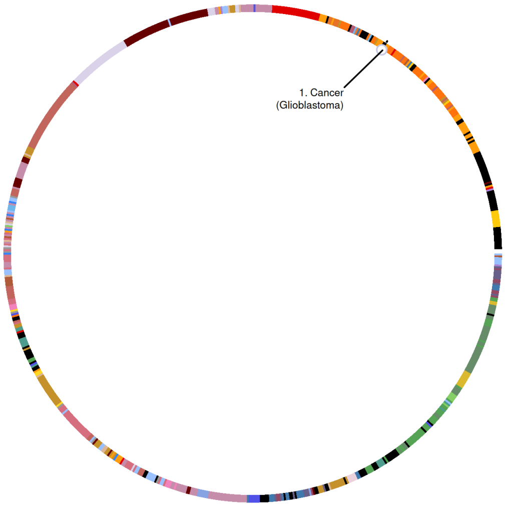

74,064 European ancestry individuals

Pulmonary function

21946350

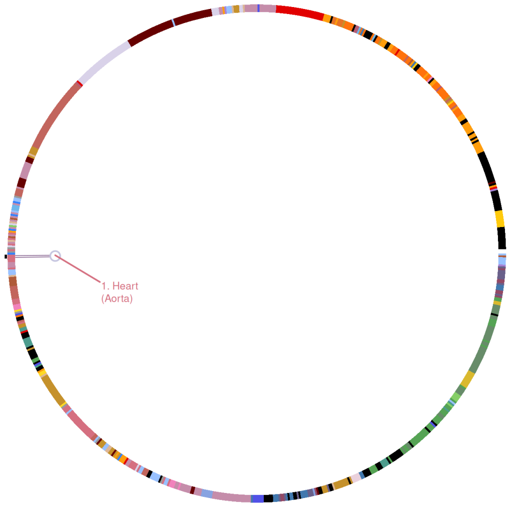

48,201 European ancestry individuals

Liver enzyme levels (gamma-glutamyl transferase)

22001757

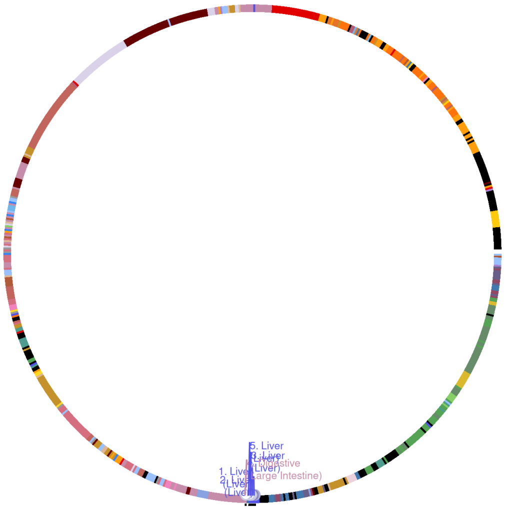

Up to 52,350 European ancestry individuals, up to 8,739 Indian Asian ancestry individuals

Celiac disease

22057235

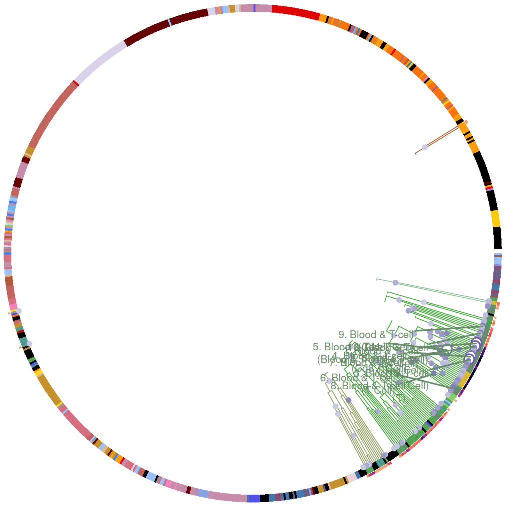

11,812 European ancestry cases, 229 Indian ancestry cases, 11,837 European ancestry controls, 391 Indian ancestry controls

Mean platelet volume

22139419

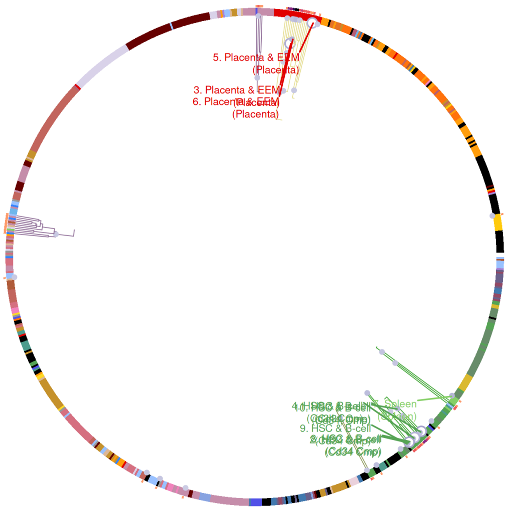

13,106 European ancestry individuals, 1,652 Val Borbera (founder/genetic isolate) individuals, 593 Carlantino (founder/genetic isolate) individuals, 1,146 Friuli Venezia Giulia (founder/genetic isolate) individuals, 1,213 South Tyrolean (founder/genetic isolate) individuals, 890 Sorbian (founder/genetic isolate) individuals

Platelet count

22139419

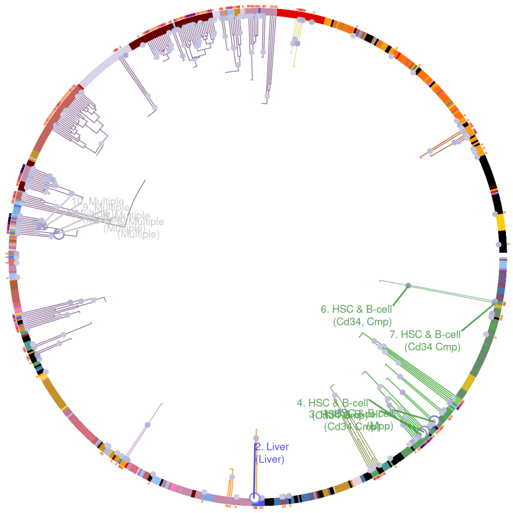

38,891 European ancestry individuals, 1,661 Val Borbera (founder/genetic isolate) individuals, 521 Carlantino (founder/genetic isolate) individuals, 1,198 Friuli Venezia Giulia (founder/genetic isolate) individuals, 1,213 South Tyrolean (founder/genetic isolate) individuals, 890 Sorbian (founder/genetic isolate) individuals, 4,292 Sardinian (founder/genetic isolate) individuals

Fasting blood glucose

22581228

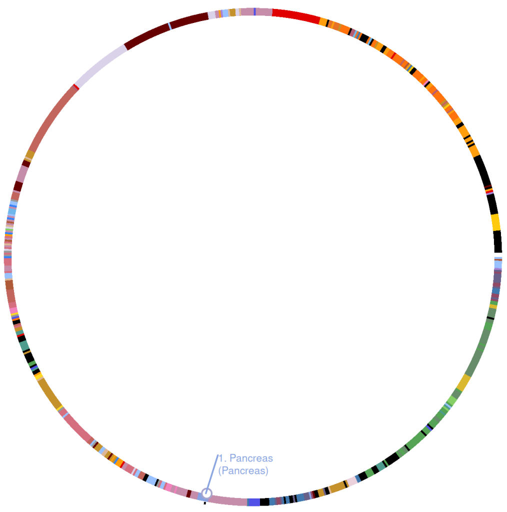

Up to 58,074 European ancestry individuals

Fasting blood glucose (BMI interaction)

22581228

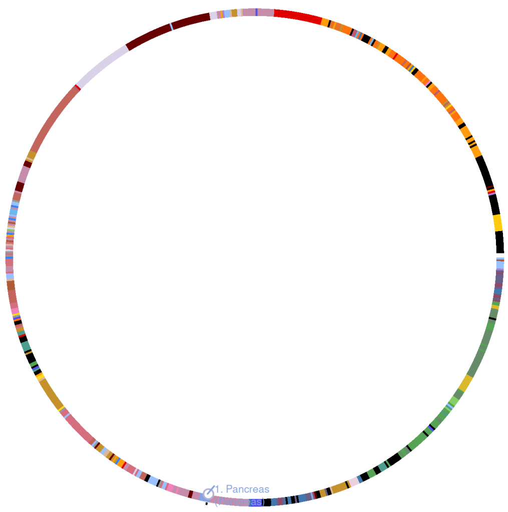

Up to 58,074 European ancestry individuals

Inflammatory bowel disease

23128233

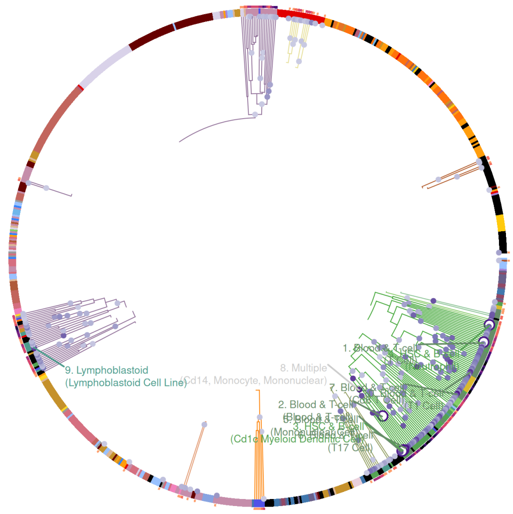

12,924 European ancestry cases, 21,442 European ancestry controls

Psoriasis

23143594

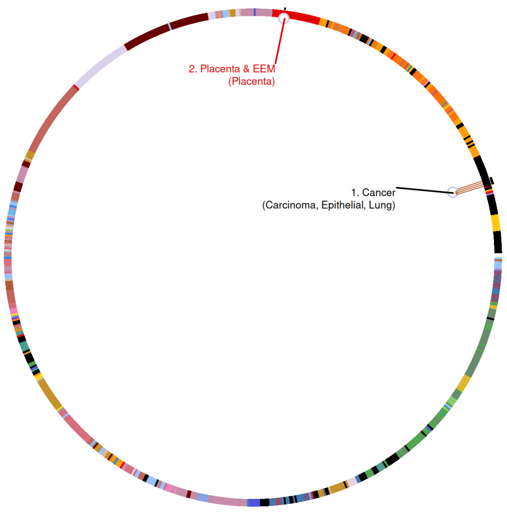

10,588 European ancestry cases, 22,806 European ancestry controls

Rheumatoid arthritis

23143596

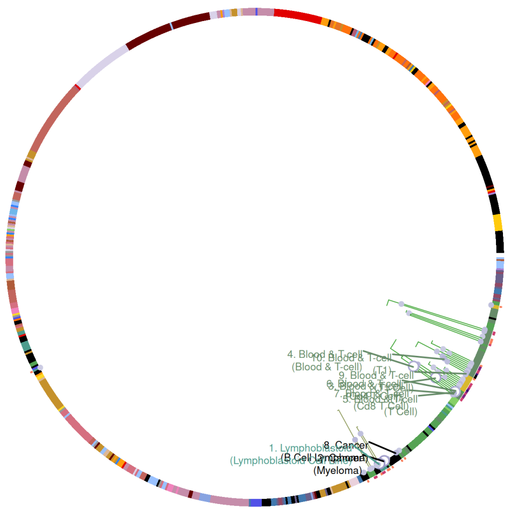

up to 13,838 European ancestry cases, up to 33,742 European ancestry controls

Red blood cell traits

23222517

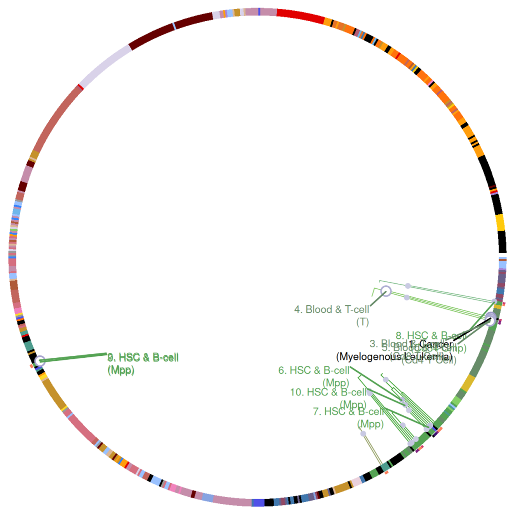

62,553 European ancestry individuals, 9,308 South Asian ancestry individuals

Urate levels

23263486

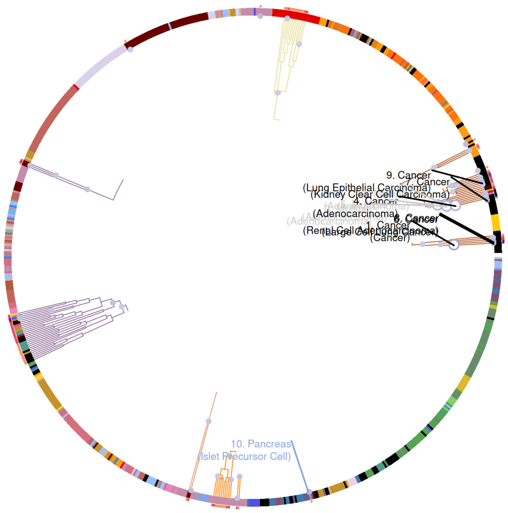

49,825 European ancestry males, 60,522 European ancestry females

Pulmonary function (smoking interaction)

23284291

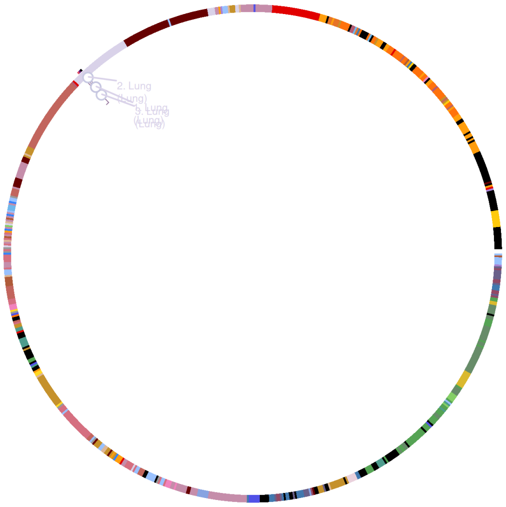

50,047 European ancestry individuals

Thyroid hormone levels

23408906

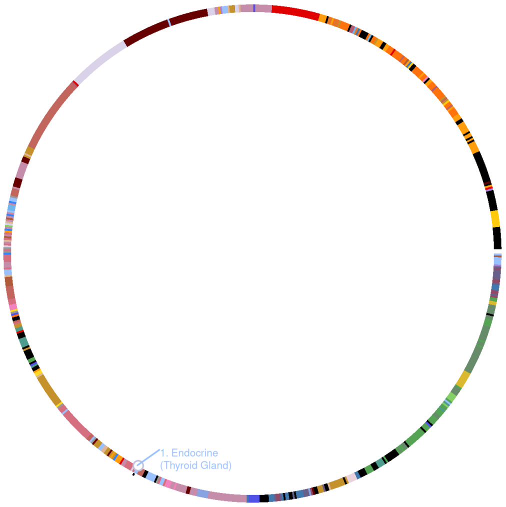

up to 14,459 European ancestry females, up to 10,936 European ancestry males, up to 433 Old Order Amish females, up to 592 Old Order Amish males

Breast cancer

23535729

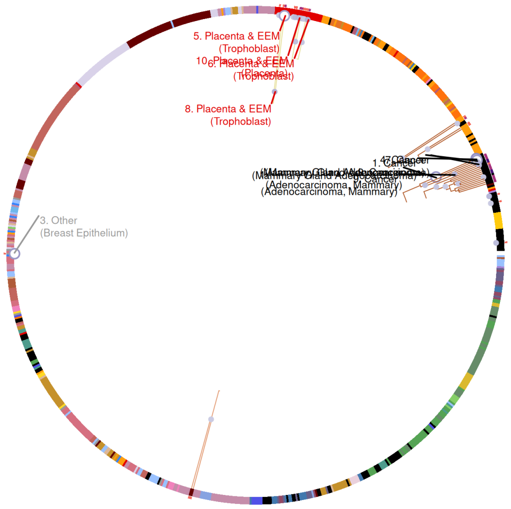

10,052 European ancestry cases, 12,575 European ancestry controls

Ankylosing spondylitis

23749187

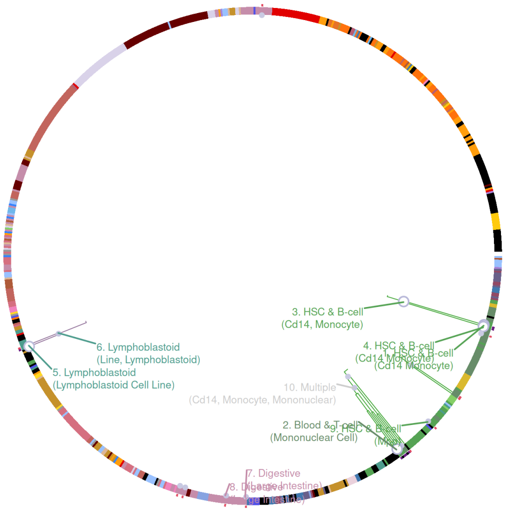

9,069 European ancestry cases, 1,550 East Asian ancestry cases, 13,578 European ancestry controls, 1,567 East Asian ancestry controls

Self-reported allergy

23817569

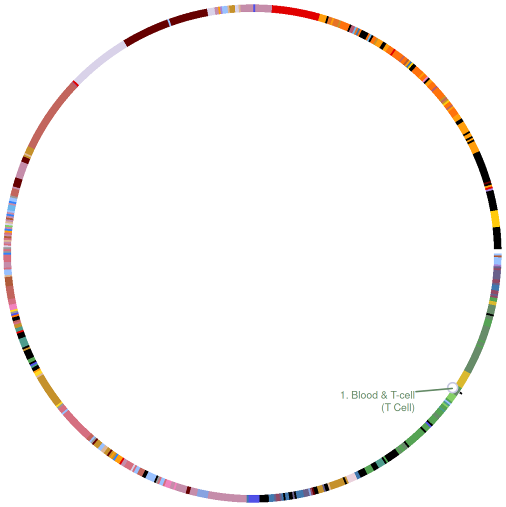

53,862 European ancestry individuals

Multiple sclerosis

24076602

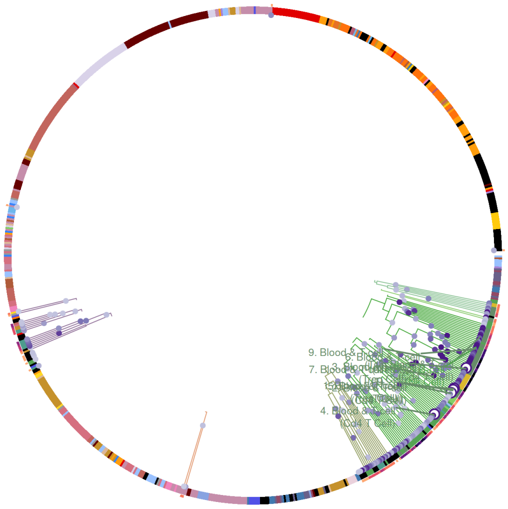

14,498 European ancestry cases, 24,091 European ancestry controls

Cholesterol, total

24097068

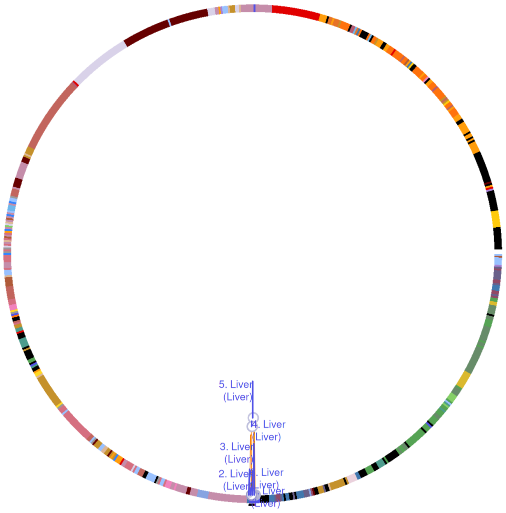

94,595 European ancestry individuals

HDL cholesterol

24097068

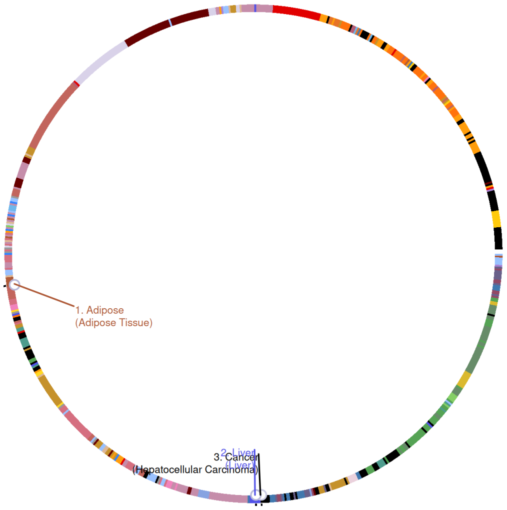

94,595 European ancestry individuals

LDL cholesterol

24097068

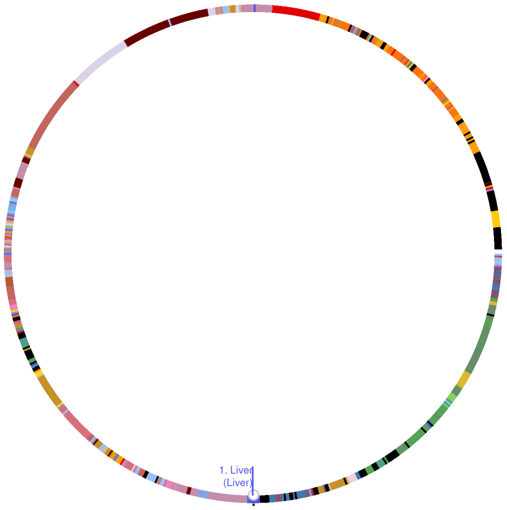

94,595 European ancestry individuals

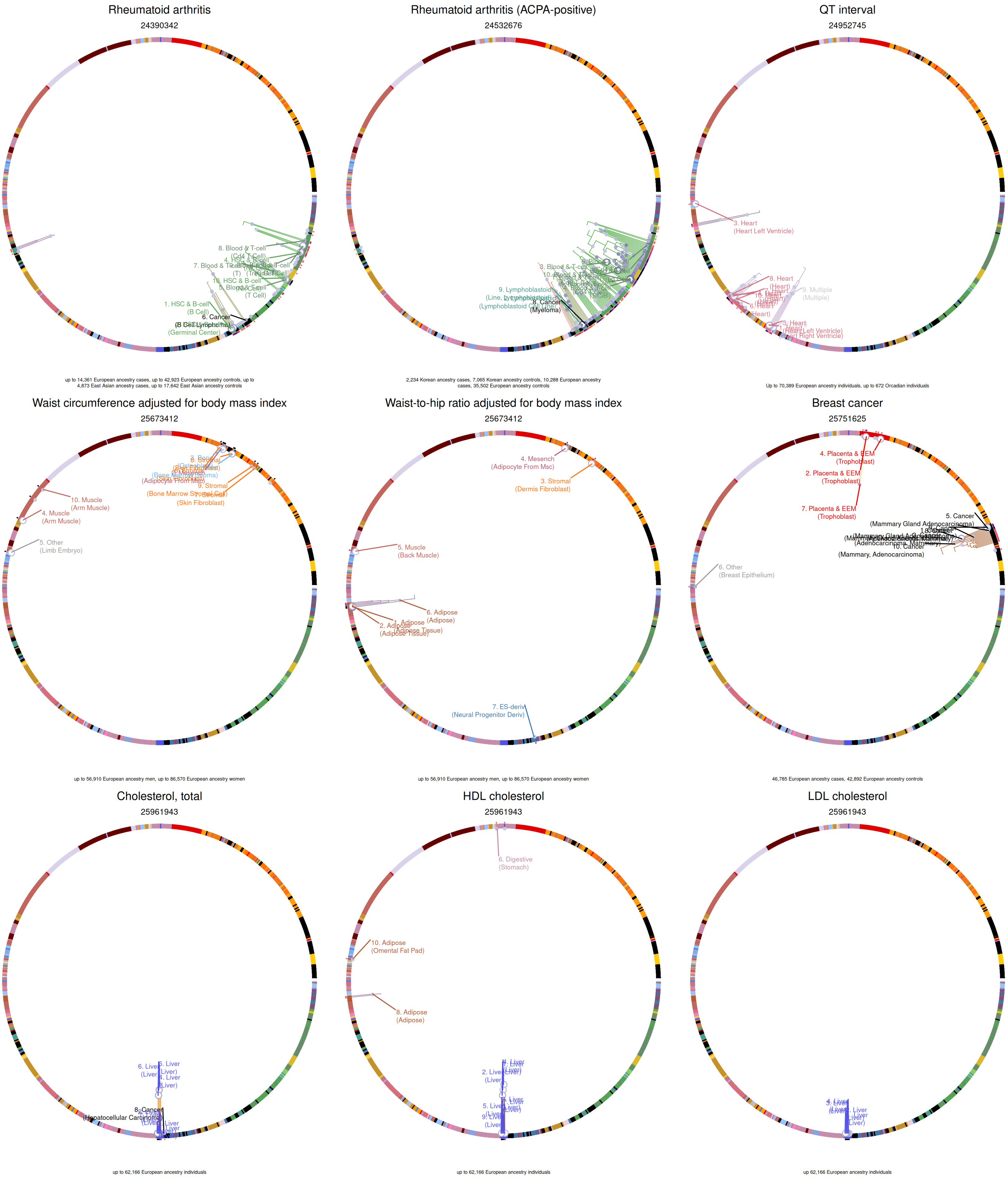
Rheumatoid arthritis
24390342
up to 14,361 European ancestry cases, up to 42,923 European ancestry controls, up to 4,873 East Asian ancestry cases, up to 17,642 East Asian ancestry controls
Rheumatoid arthritis (ACPA-positive)
24532676
2,234 Korean ancestry cases, 7,065 Korean ancestry controls, 10,288 European ancestry cases, 35,502 European ancestry controls
QT interval
24952745
Up to 70,389 European ancestry individuals, up to 672 Orcadian individuals
Waist circumference adjusted for body mass index
25673412
up to 56,910 European ancestry men, up to 86,570 European ancestry women
Waist-to-hip ratio adjusted for body mass index
25673412
up to 56,910 European ancestry men, up to 86,570 European ancestry women
Breast cancer
25751625
46,785 European ancestry cases, 42,892 European ancestry controls
Cholesterol, total
25961943
up to 62,166 European ancestry individuals
HDL cholesterol
25961943
up to 62,166 European ancestry individuals
LDL cholesterol
25961943
up 62,166 European ancestry individuals

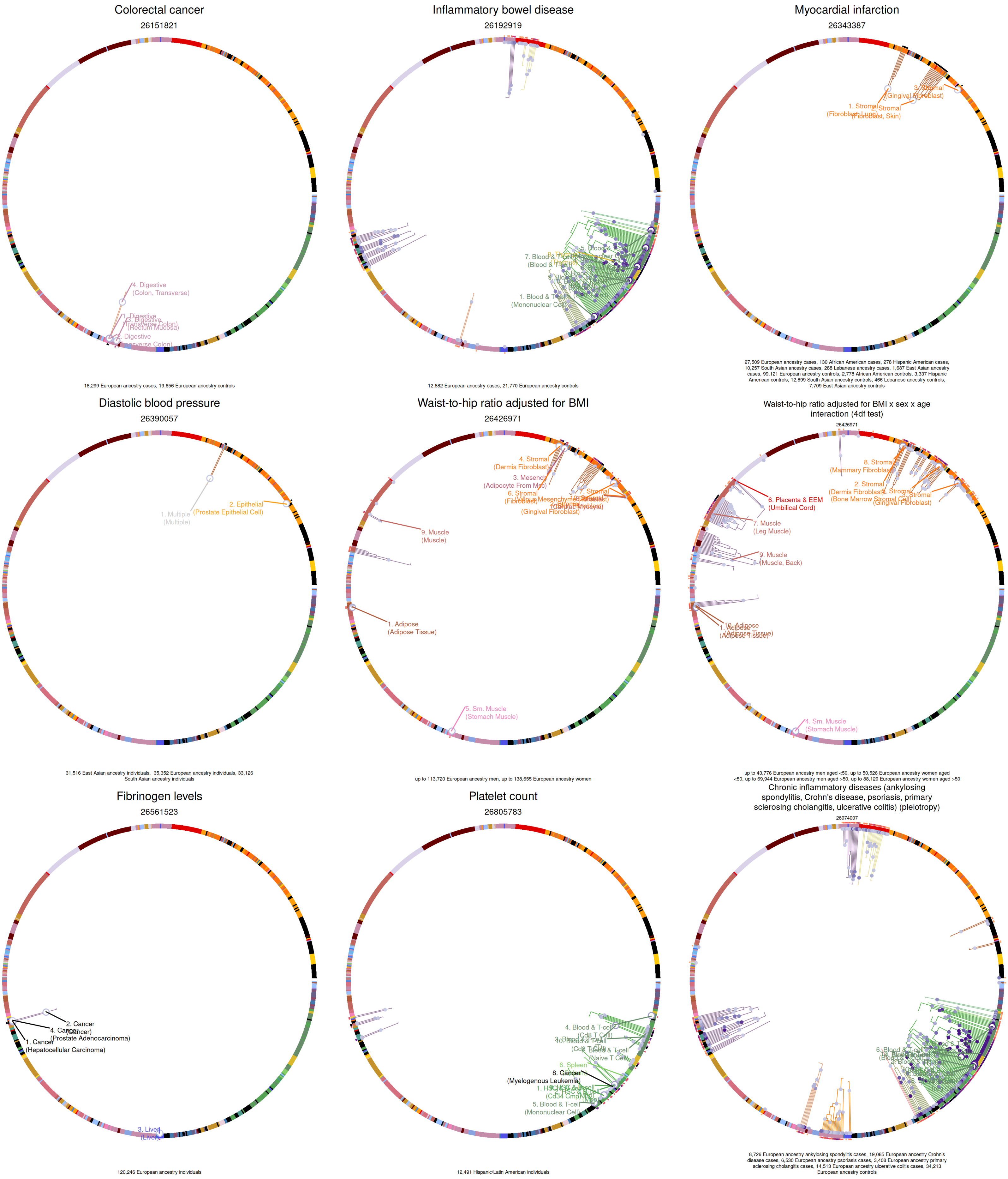

Colorectal cancer
26151821
4. Digestive
(Colon, Transverse)
18,299 European ancestry cases, 19,656 European ancestry controls
Inflammatory bowel disease
26192919
1. Blood & T-cell
(Mononuclear Cell)
12,882 European ancestry cases, 21,770 European ancestry controls
Myocardial infarction
26343387
1. Stromal
3. Stromal
27,509 European ancestry cases, 130 African American cases, 278 Hispanic American cases, 10,257 South Asian ancestry cases, 288 Lebanese ancestry cases, 1,687 East Asian ancestry cases, 99,121 European ancestry controls, 2,778 African American controls, 3,337 Hispanic American controls, 12,899 South Asian ancestry controls, 466 Lebanese ancestry controls, 7,709 East Asian ancestry controls
Diastolic blood pressure
26390057
2. Epithelial
(Prostate Epithelial Cell)
1. Multiple
(Multiple)
31,516 East Asian ancestry individuals, 35,352 European ancestry individuals, 33,126 South Asian ancestry individuals
Waist-to-hip ratio adjusted for BMI
26426971
4. Stromal
(Dermis Fibroblast)
3. Mesench
(Adipocyte From Msc)
9. Muscle
(Muscle)
1. Adipose
(Adipose Tissue)
5. Sm. Muscle
(Stomach Muscle)
up to 113,720 European ancestry men, up to 138,655 European ancestry women
Waist-to-hip ratio adjusted for BMI x sex x age interaction (4df test)
26426971
8. Stromal
2. Stromal
(Dermis Fibroblast)
6. Placenta & EEM
(Umbilical Cord)
7. Muscle
(Leg Muscle)
9. Muscle
(Muscle, Back)
4. Sm. Muscle
(Stomach Muscle)
up to 43,776 European ancestry men aged <50, up to 50,526 European ancestry women aged <50, up to 69,944 European ancestry men aged >50, up to 88,129 European ancestry women aged >50
Fibrinogen levels
26561523
2. Cancer
(Prostate Adenocarcinoma)
1. Cancer
(Hepatocellular Carcinoma)
3. Liver
(Liver)
120,246 European ancestry individuals
Platelet count
26805783
4. Blood & T-cell
6. Spleen
8. Cancer
(Myelogenous Leukemia)
5. Blood & T-cell
(Mononuclear Cell)
12,491 Hispanic/Latin American individuals
Chronic inflammatory diseases (ankylosing spondylitis, Crohn's disease, psoriasis, primary sclerosing cholangitis, ulcerative colitis) (pleiotropy)
26974007
8,726 European ancestry ankylosing spondylitis cases, 19,085 European ancestry Crohn's disease cases, 6,530 European ancestry psoriasis cases, 3,408 European ancestry primary sclerosing cholangitis cases, 14,513 European ancestry ulcerative colitis cases, 34,213 European ancestry controls

Metabolite levels (lipoprotein measures)

27005778

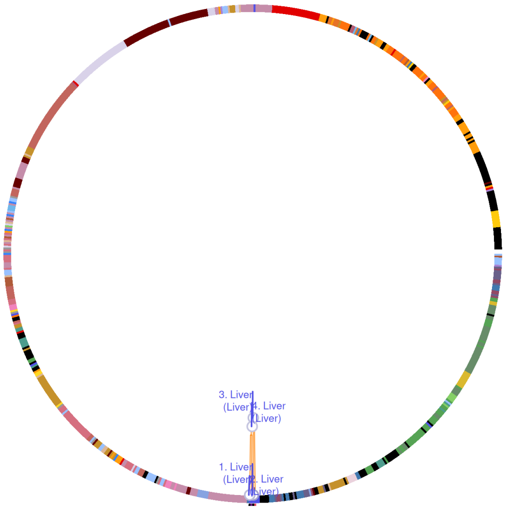

2,118 Erasmus Rucphen (founder/genetic isolate), 22,807 European ancestry individuals

Metabolite levels (small molecules and protein measures)

27005778

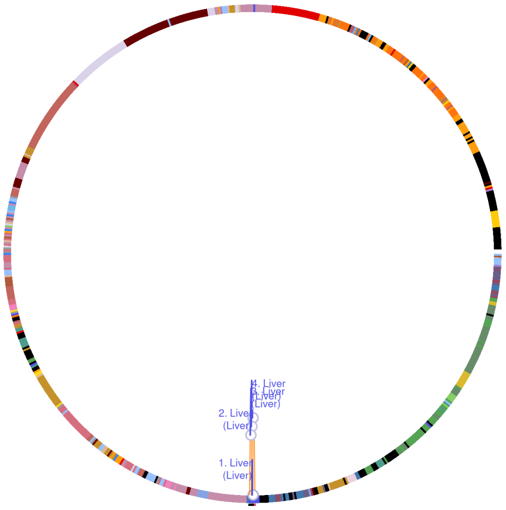

2,118 Erasmus Rucphen (founder/genetic isolate), 22,807 European ancestry individuals

Migraine

27182965

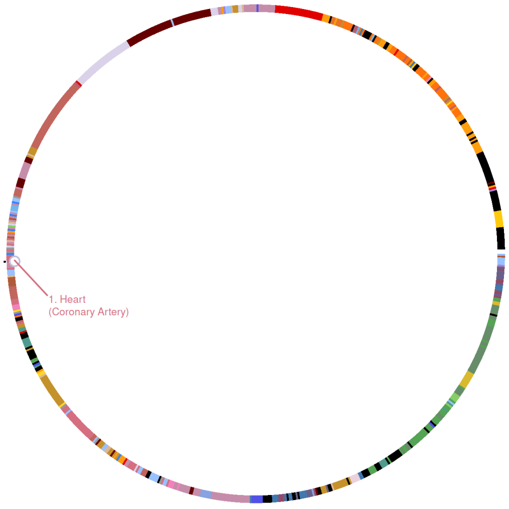

53,109 European ancestry cases, 230,876 European ancestry controls

C-reactive protein levels or HDL-cholesterol levels (pleiotropy)

27286809

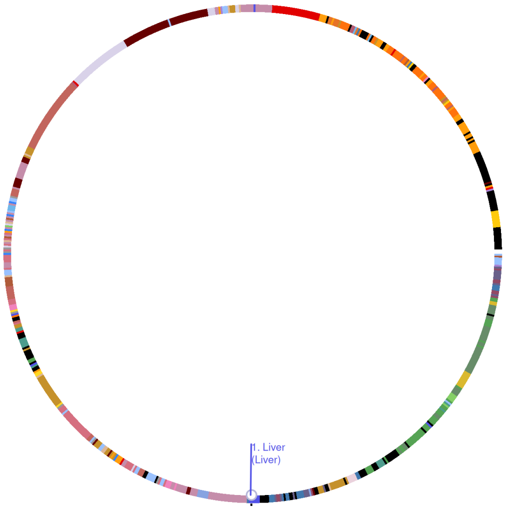

65,000 individuals (C-reactive protein), 99,900 individuals (HDL-cholesterol)

C-reactive protein levels or LDL-cholesterol levels (pleiotropy)

27286809

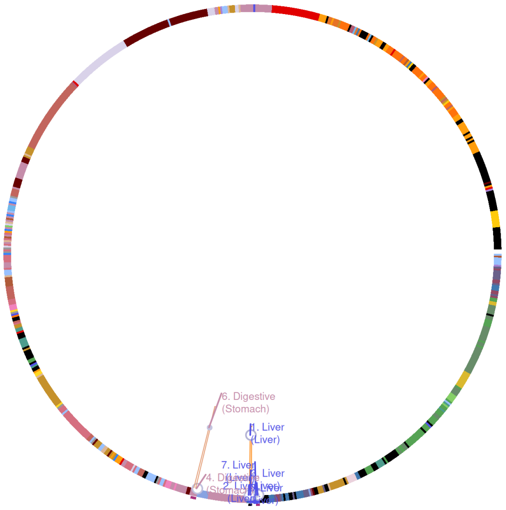

65,000 individuals (C-reactive protein), 95,454 individuals (LDL-cholesterol)

Migraine

27322543

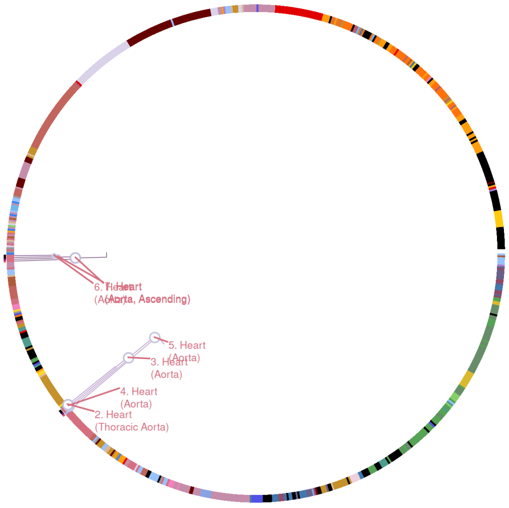

59,674 European ancestry cases, 316,078 European ancestry controls

QRS duration

27659466

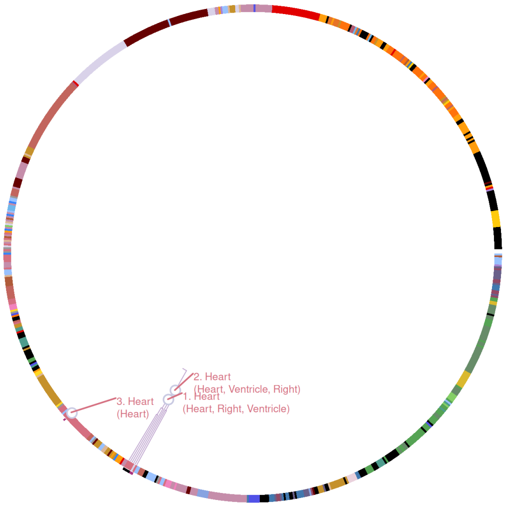

59,565 European ancestry individuals, 690 Orcadian (founder/genetic isolate) individuals

Resting heart rate

27798624

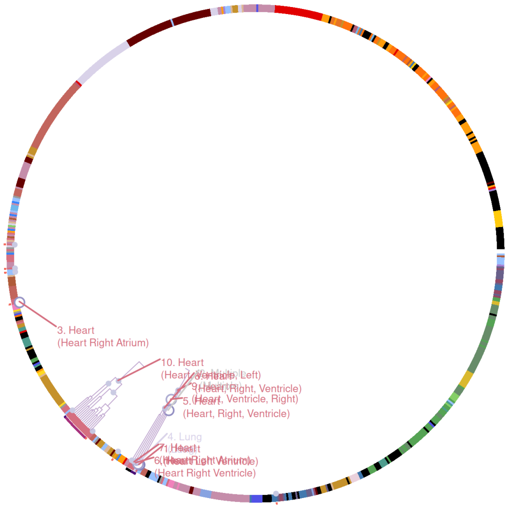

127,919 European ancestry individuals, 2,478 Asian ancestry individuals, 1,734 Black individuals, 684 Mixed ancestry individuals, 1,436 individuals

Diastolic blood pressure

27841878

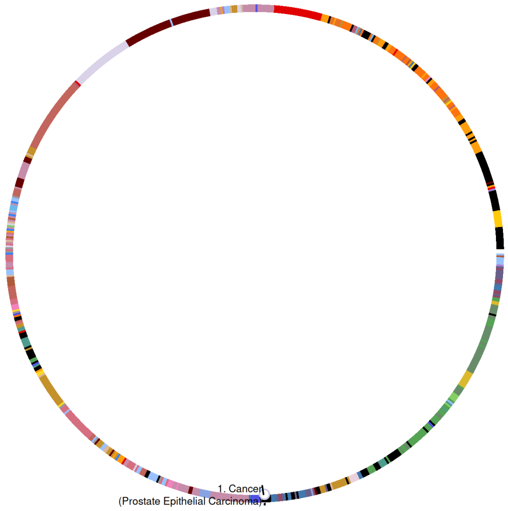

295,529 European ancestry individuals, 8,231 Latino individuals, 3,058 African American individuals, 2,029 African British individuals, 7,701 East Asian ancestry individuals, 2,735 South Asian ancestry individuals, 1,979 mixed and unknown ancestry individuals

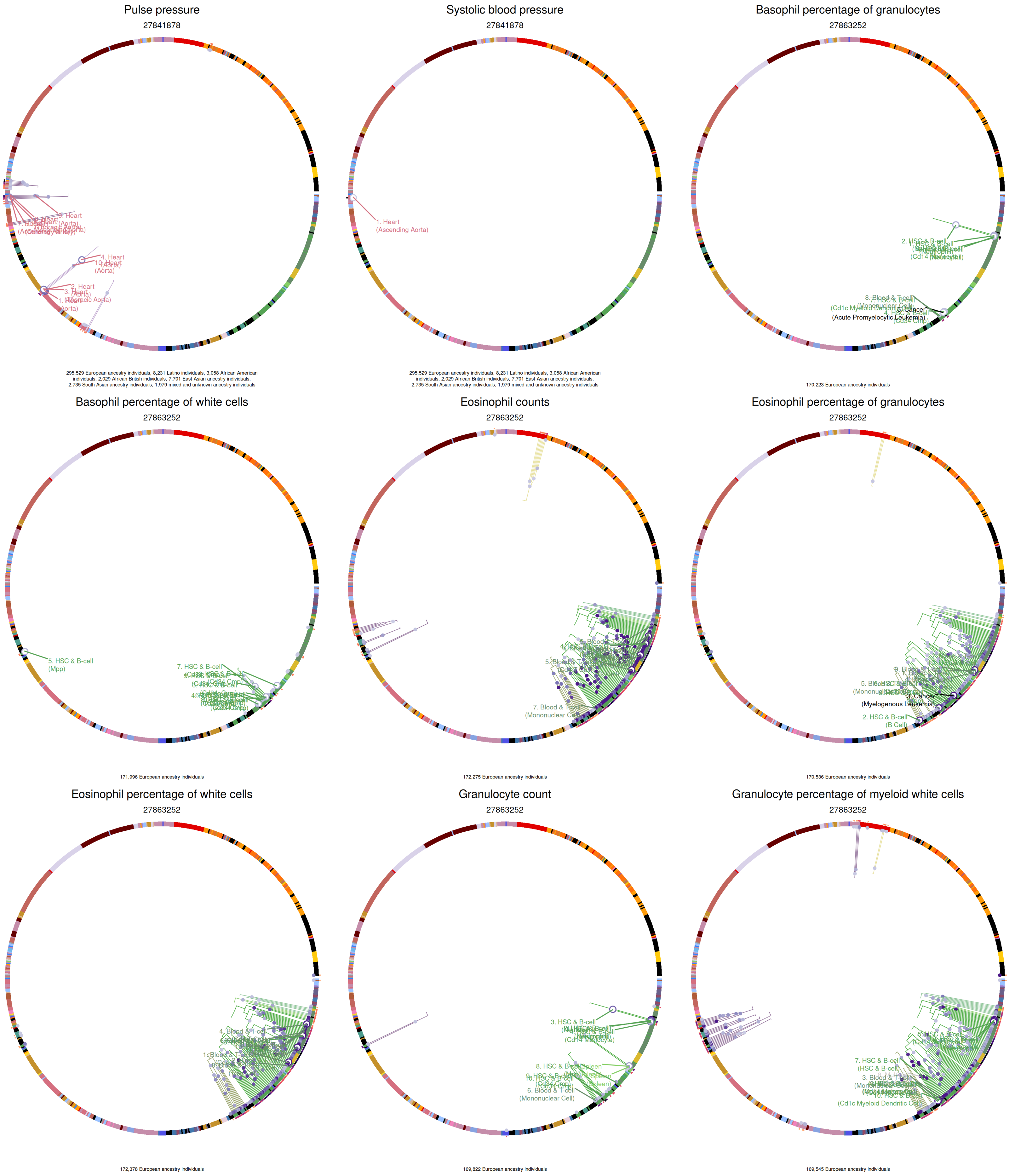

Pulse pressure
27841878
295,529 European ancestry individuals, 8,231 Latino individuals, 3,058 African American individuals, 2,029 African British individuals, 7,701 East Asian ancestry individuals, 2,735 South Asian ancestry individuals, 1,979 mixed and unknown ancestry individuals
Systolic blood pressure
27841878
1. Heart
(Ascending Aorta)
295,529 European ancestry individuals, 8,231 Latino individuals, 3,058 African American individuals, 2,029 African British individuals, 7,701 East Asian ancestry individuals, 2,735 South Asian ancestry individuals, 1,979 mixed and unknown ancestry individuals
Basophil percentage of granulocytes
27863252
5. Cancer
(Acute Promyelocytic Leukemia)
170,223 European ancestry individuals
Basophil percentage of white cells
27863252
5. HSC & B-cell
(Mpp)
7. HSC & B-cell
171,996 European ancestry individuals
Eosinophil counts
27863252
7. Blood & T-cell
(Mononuclear Cell)
172,275 European ancestry individuals
Eosinophil percentage of granulocytes
27863252
3. Cancer
(Myelogenous Leukemia)
2. HSC & B-cell
(B Cell)
170,536 European ancestry individuals
Eosinophil percentage of white cells
27863252
4. Blood & T-cell
172,378 European ancestry individuals
Granulocyte count
27863252
3. HSC & B-cell
(Cd14 Monocyte)
6. Blood & T-cell
(Mononuclear Cell)
169,822 European ancestry individuals
Granulocyte percentage of myeloid white cells
27863252
7. HSC & B-cell
(HSC & B-cell)
(Cd1c Myeloid Dendritic Cell)
169,545 European ancestry individuals

Hematocrit

27863252

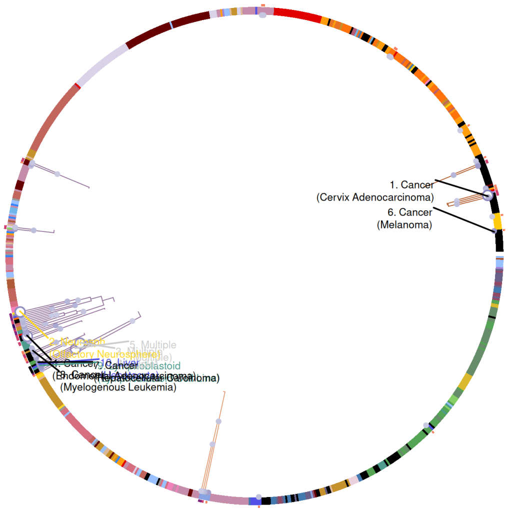

173,039 European ancestry individuals

Hemoglobin concentration

27863252

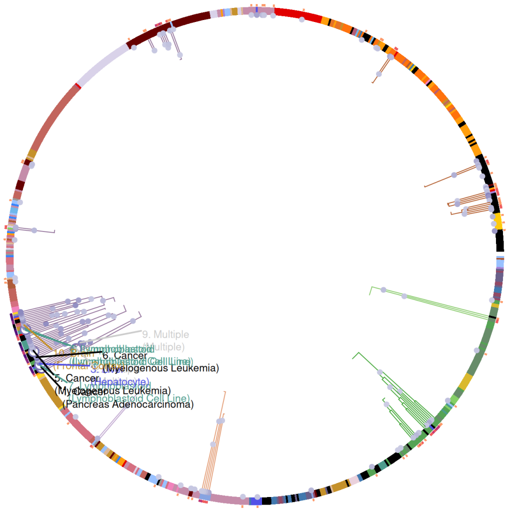

172,925 European ancestry individuals

High light scatter reticulocyte count

27863252

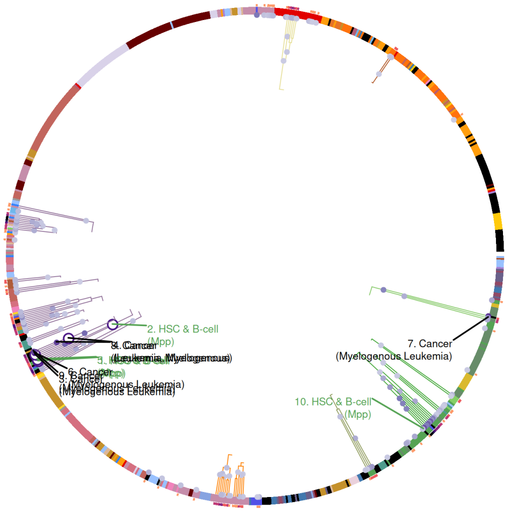

170,761 European ancestry individuals

High light scatter reticulocyte percentage of red cells

27863252

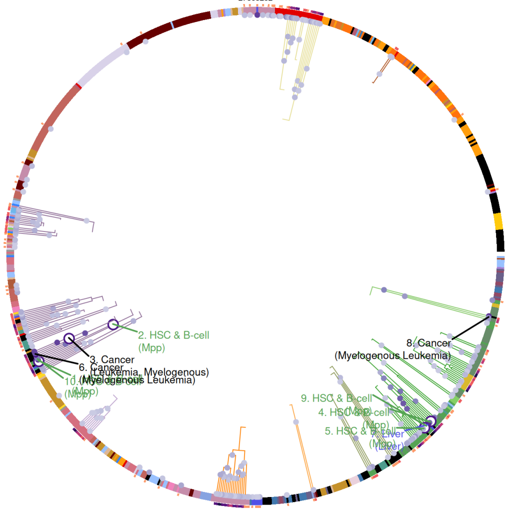

170,763 European ancestry individuals

Immature fraction of reticulocytes

27863252

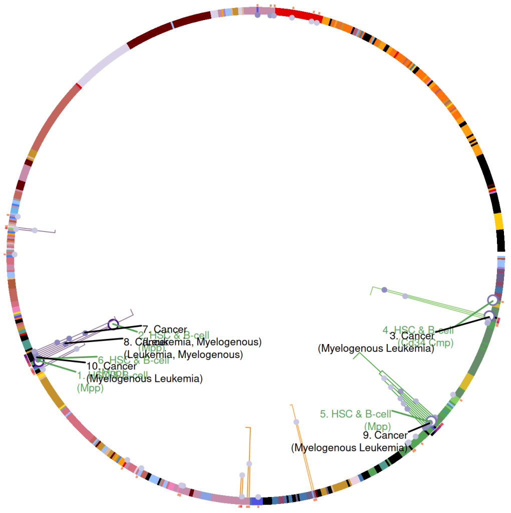

170,548 European ancestry individuals

Lymphocyte counts

27863252

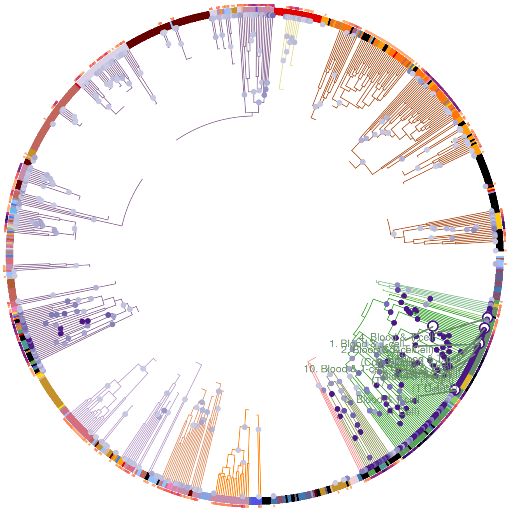

171,643 European ancestry individuals

Lymphocyte percentage of white cells

27863252

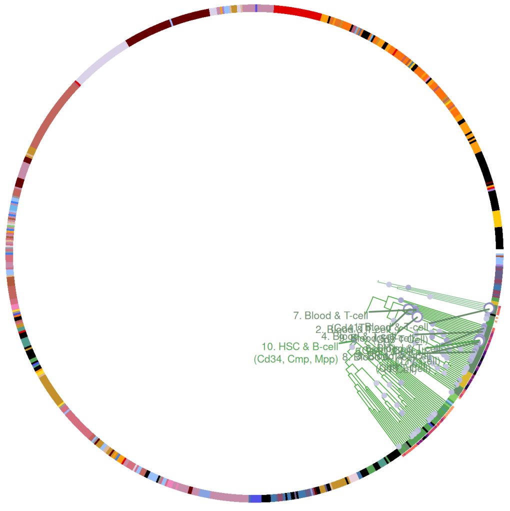

171,748 European ancestry individuals

Mean corpuscular hemoglobin

27863252

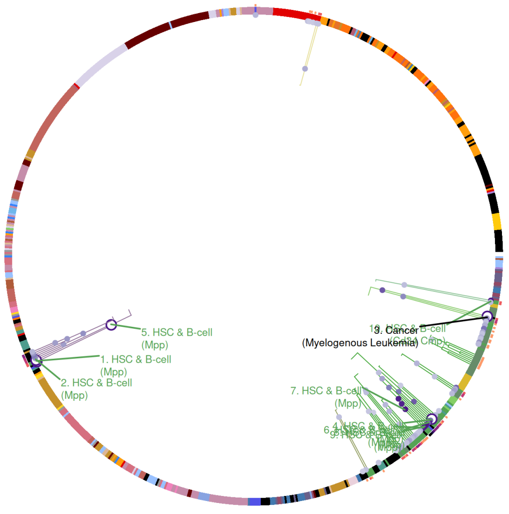

172,332 European ancestry individuals

Mean corpuscular hemoglobin concentration

27863252

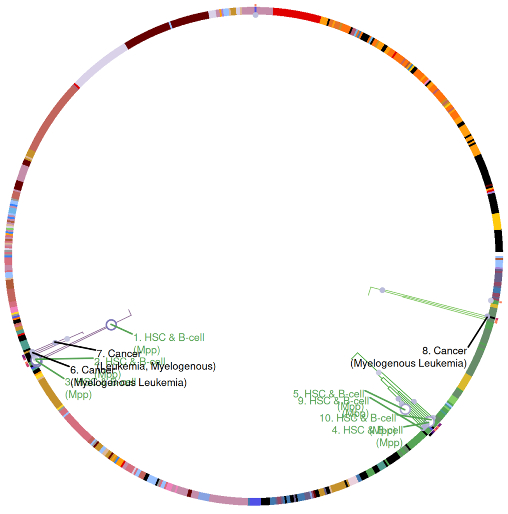

172,851 European ancestry individuals

Mean corpuscular volume

27863252

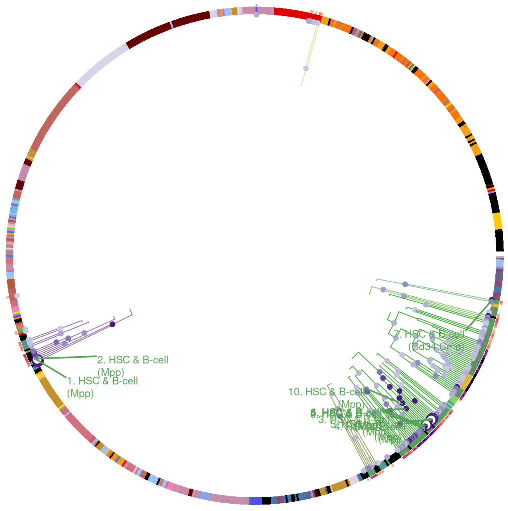

172,433 European ancestry individuals

Mean platelet volume

27863252

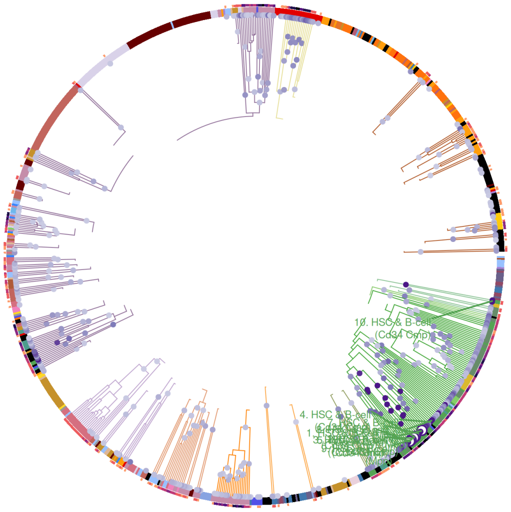

164,454 European ancestry individuals

Monocyte count

27863252

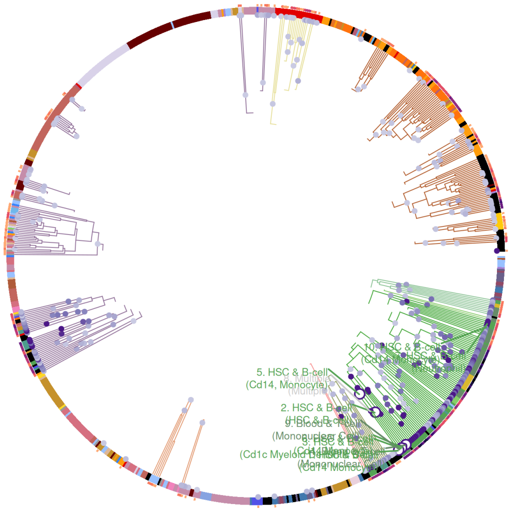

170,721 European ancestry individuals

Monocyte percentage of white cells

27863252

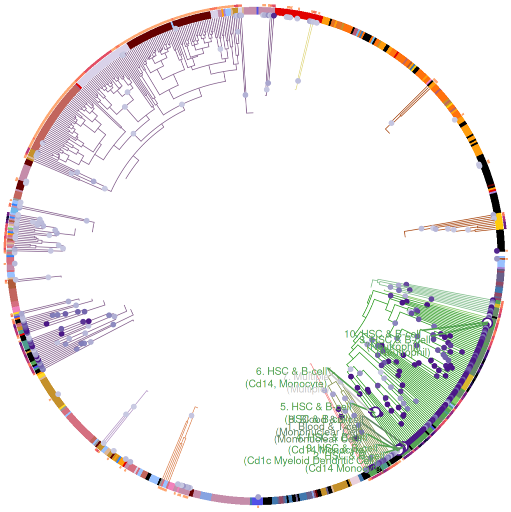

170,494 European ancestry individuals

Myeloid white cell count

27863252

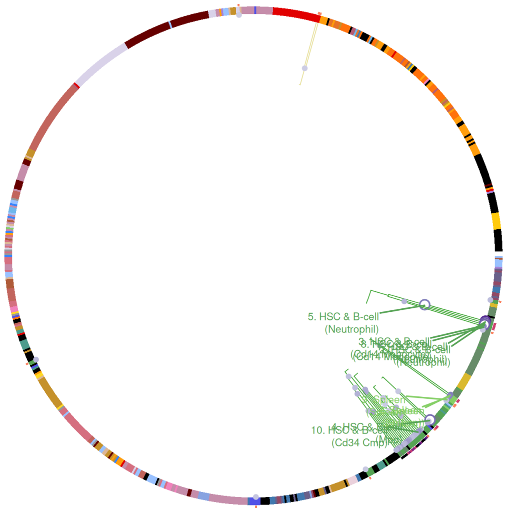

169,219 European ancestry individuals

Neutrophil count

27863252

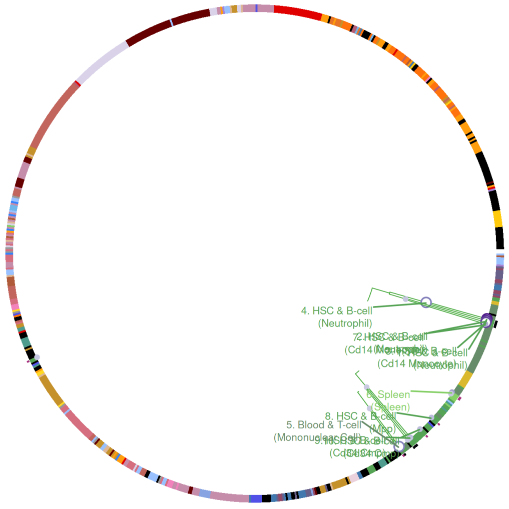

170,702 European ancestry individuals

Neutrophil percentage of granulocytes

27863252

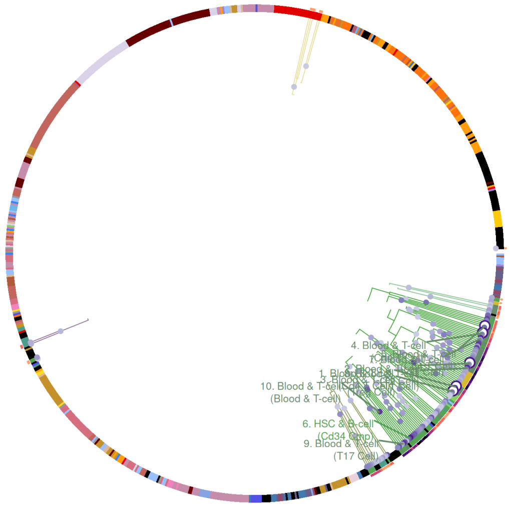

170,672 European ancestry individuals

Neutrophil percentage of white cells

27863252

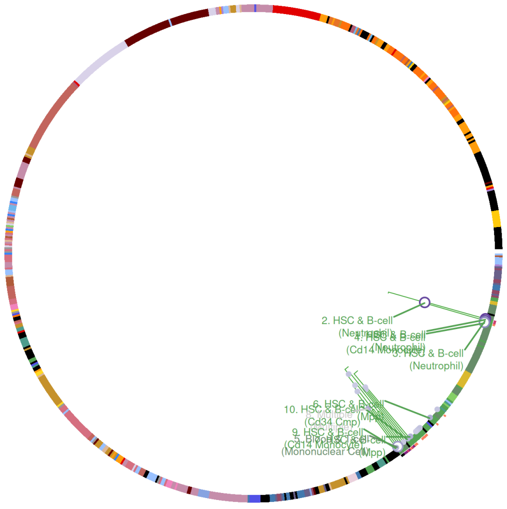

171,542 European ancestry individuals

Platelet count

27863252

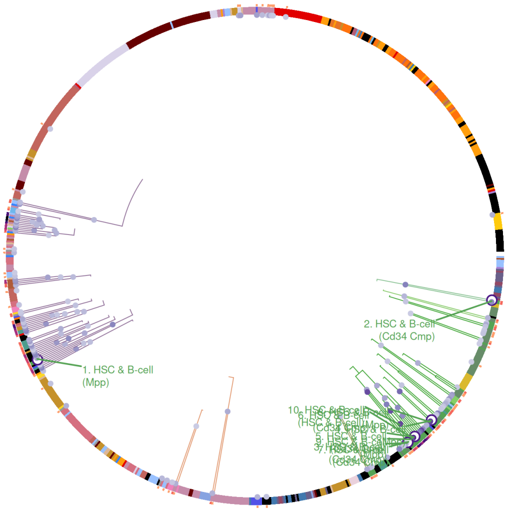

166,066 European ancestry individuals

Platelet distribution width

27863252

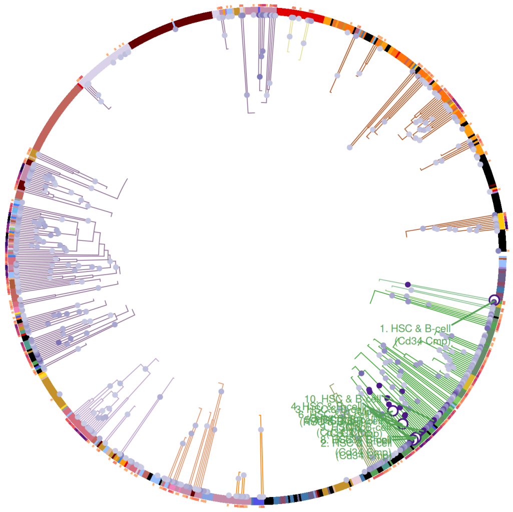

164,433 European ancestry individuals

Plateletcrit

27863252

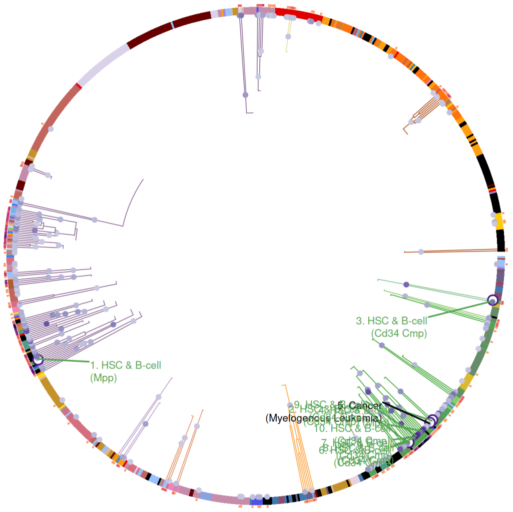

164,339 European ancestry individuals

Red blood cell count

27863252

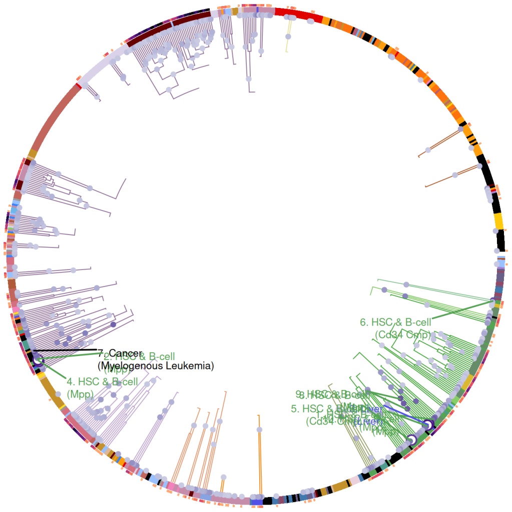

172,952 European ancestry individuals

Red cell distribution width

27863252

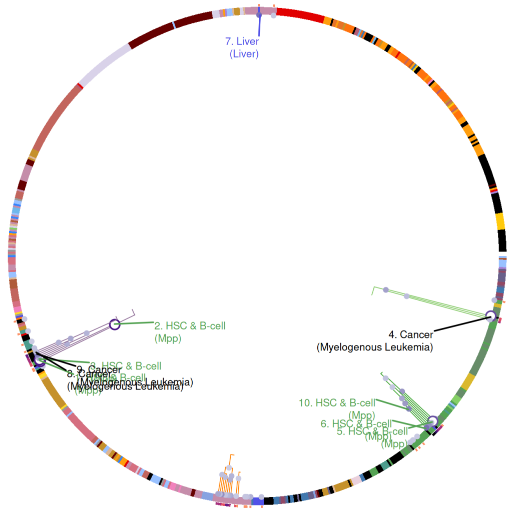

171,529 European ancestry individuals

Reticulocyte count

27863252

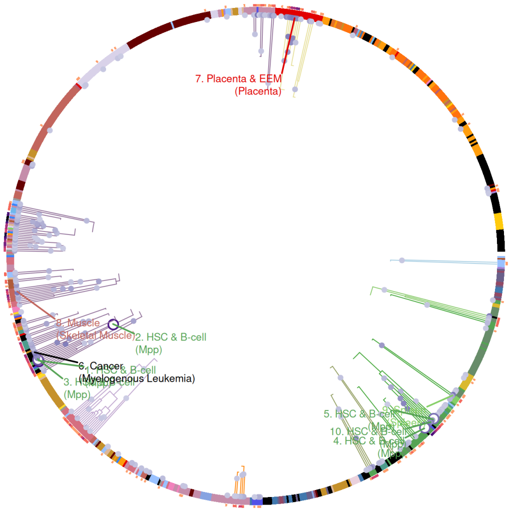

170,641 European ancestry individuals

Reticulocyte fraction of red cells

27863252

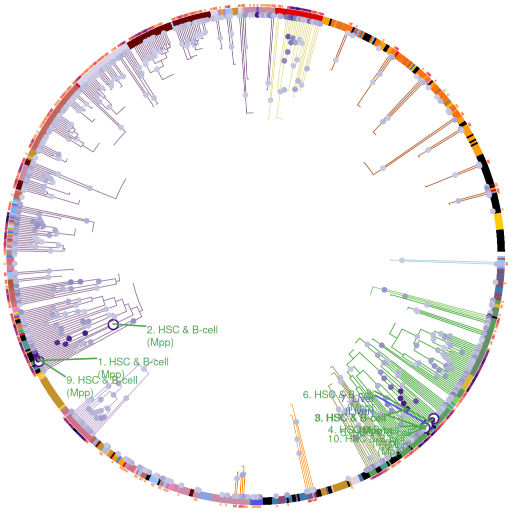

170,690 European ancestry individuals

Sum basophil neutrophil counts

27863252

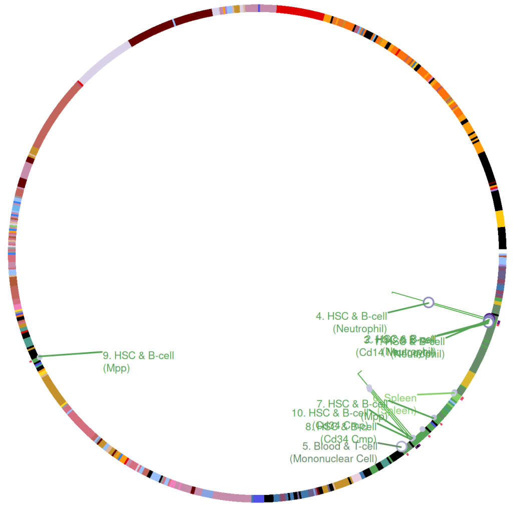

170,143 European ancestry individuals

Sum eosinophil basophil counts

27863252

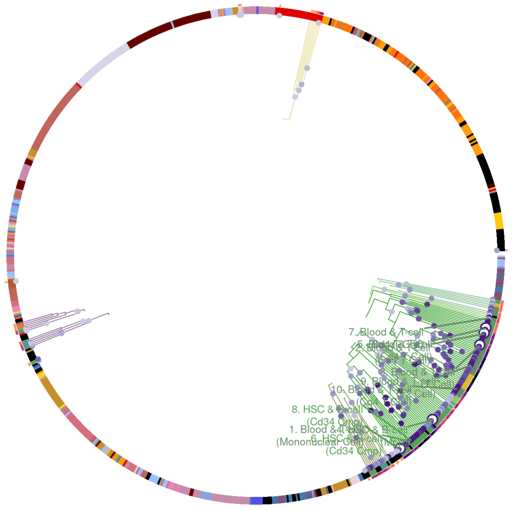

171,771 European ancestry individuals

Sum neutrophil eosinophil counts

27863252

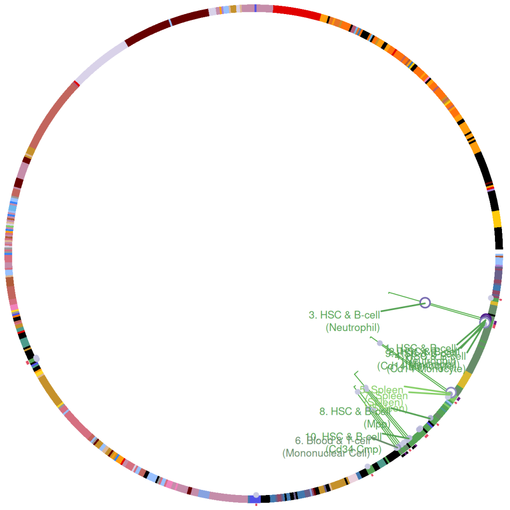

170,384 European ancestry individuals

White blood cell count
27863252

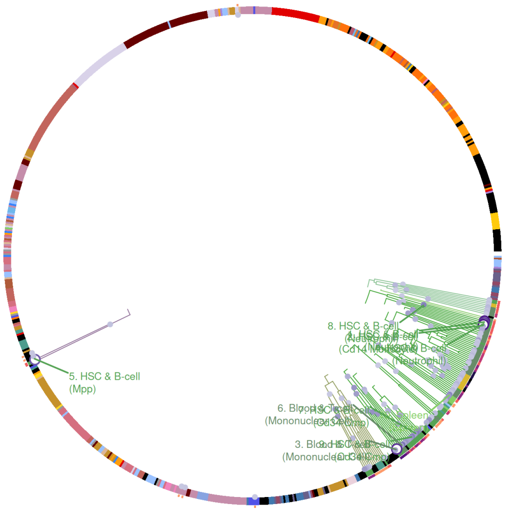

172,435 European ancestry individuals

White blood cell count (basophil)
27863252

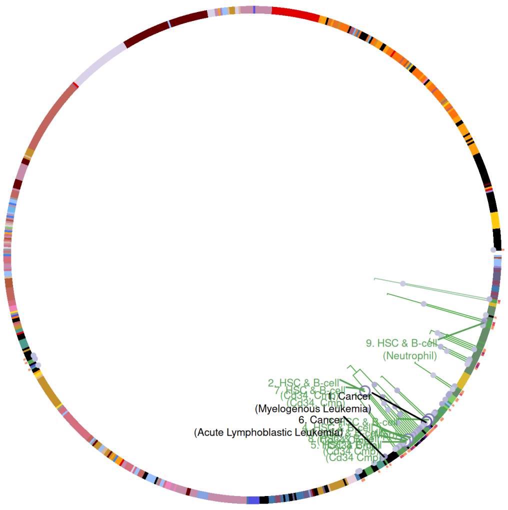

171,846 European ancestry individuals

Mean corpuscular hemoglobin
28017375

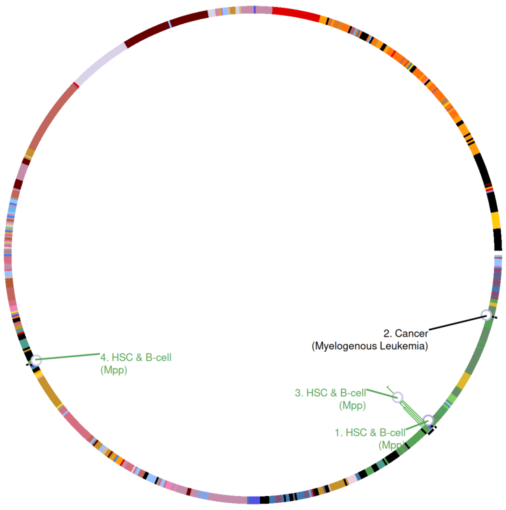

up to 21,020 European ancestry individuals, up to 3,621 African American individuals, up to 15,062 East Asian individuals.

Mean corpuscular hemoglobin concentration
28017375

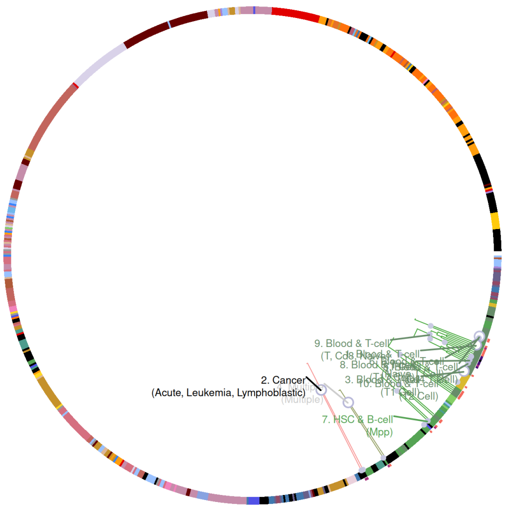

up to 21,020 European ancestry individuals, up to 3,621 African American individuals, up to 15,062 East Asian individuals.

Mean corpuscular volume
28017375

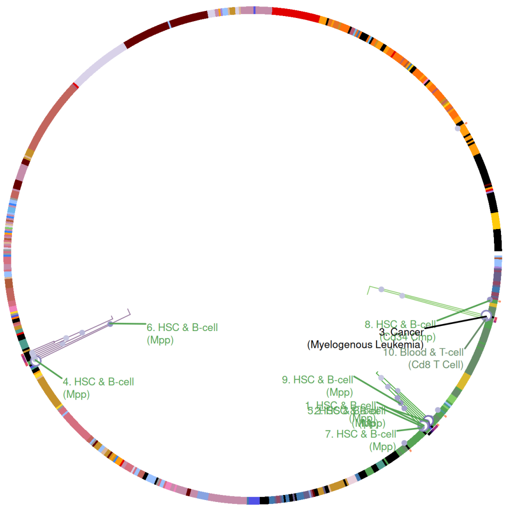

up to 29,946 European ancestry individuals, up to 6150 African American individuals, 15,074 East Asian individuals.

Crohn's disease
28067908

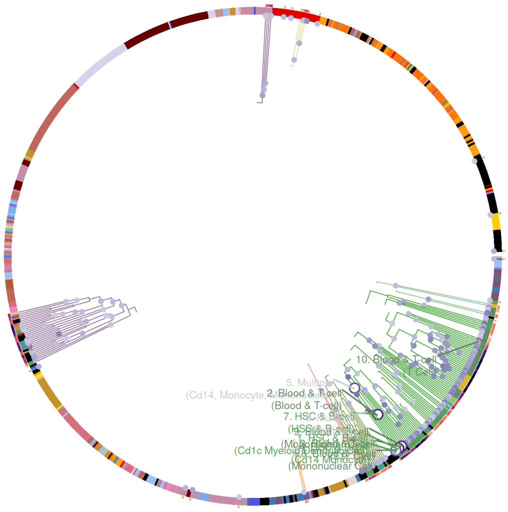

12,194 European and unknown ancestry cases, 28,072 European and unknown ancestry controls

Inflammatory bowel disease
28067908

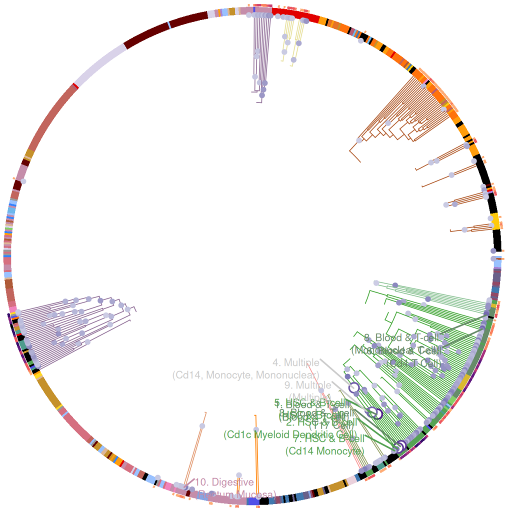

25,042 European and unknown ancestry cases, 34,915 European and unknown ancestry controls

Ulcerative colitis
28067908

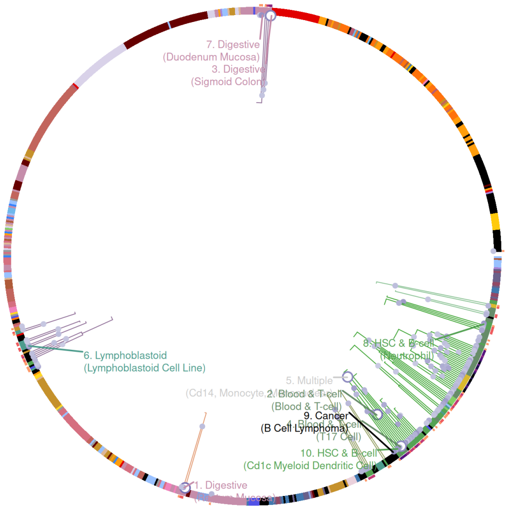

12,366 European and unknown ancestry cases, 33,609 European and unknown ancestry controls

Fibrinogen levels
28107422

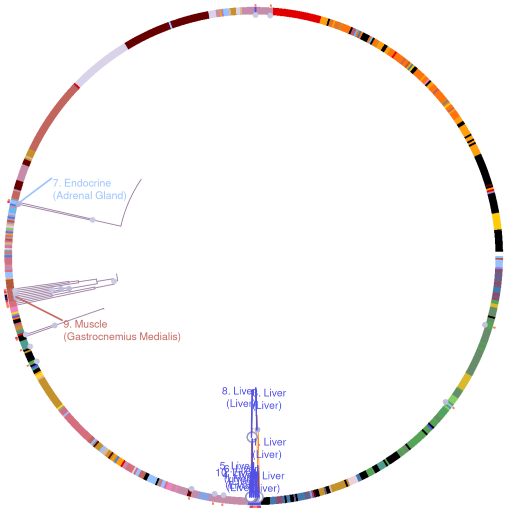

91,953 European ancestry individuals (imputed to 1000 Genomes)

Pulse pressure

28135244

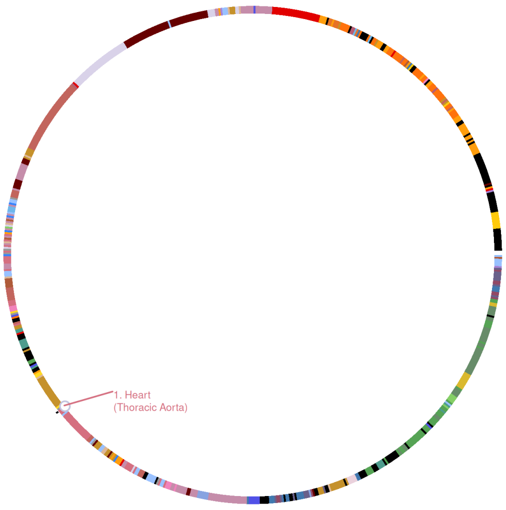

140,886 European ancestry individuals

Prostate-specific antigen levels (conditioned on lead SNPs)

28139693

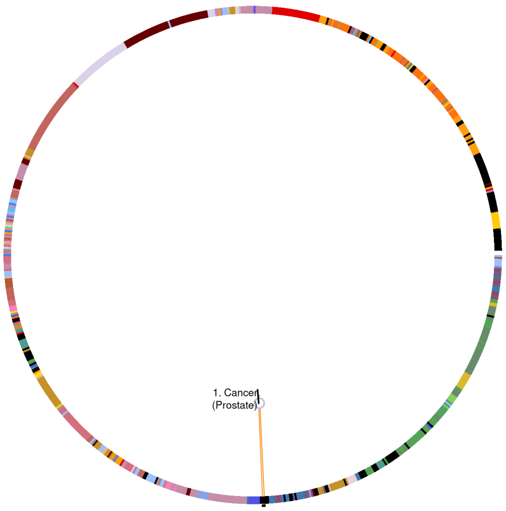

28,503 European ancestry individuals

Itch intensity from mosquito bite

28199695

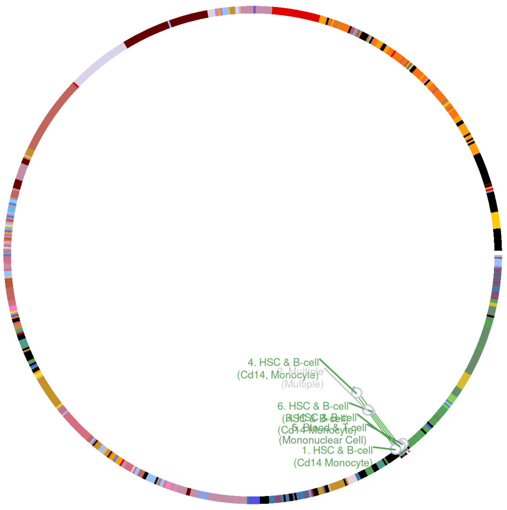

84,724 European ancestry individuals

Mosquito bite size

28199695

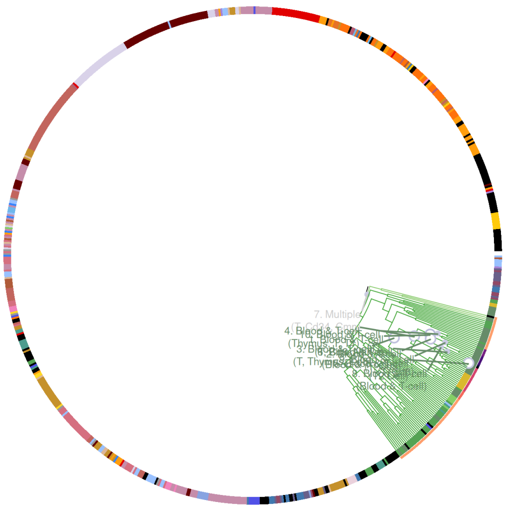

84,724 European ancestry individuals

HDL cholesterol levels

28334899

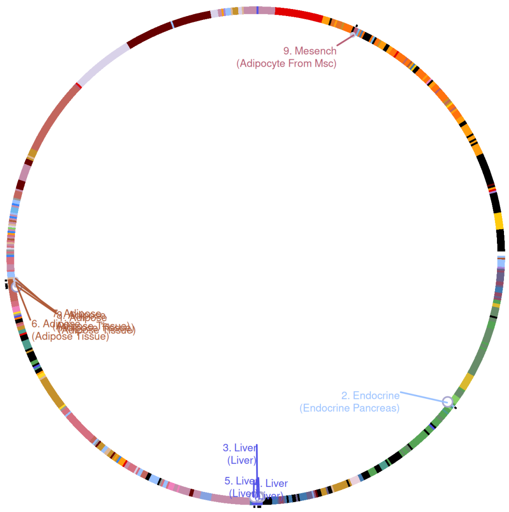

34,930 East Asian ancestry individuals, 187,167 European ancestry individuals

LDL cholesterol levels

28334899

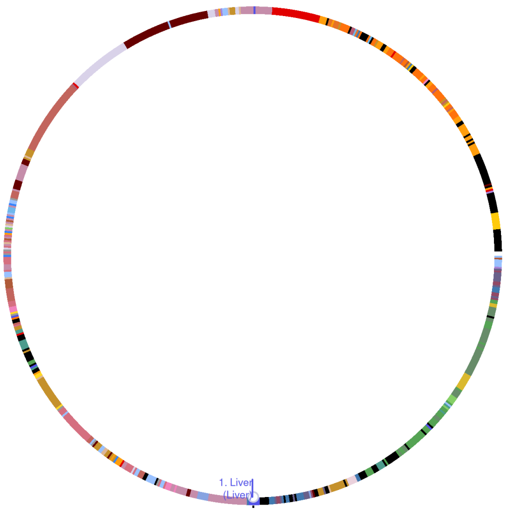

32,285 East Asian ancestry individuals, 173,082 European ancestry individuals

Total cholesterol levels

28334899

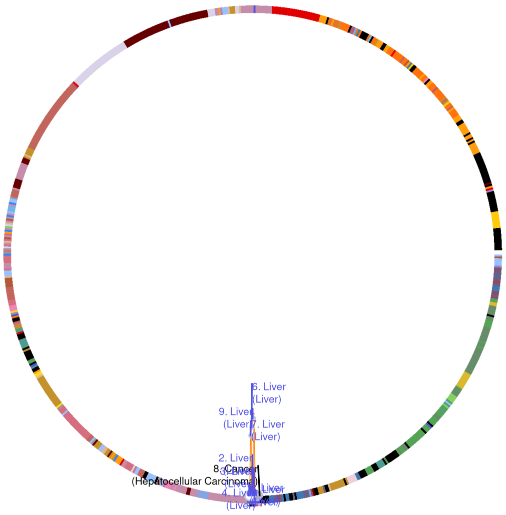

32,576 East Asian ancestry individuals, 187,365 European ancestry individuals

Waist circumference adjusted for BMI (adjusted for smoking behaviour)

28443625

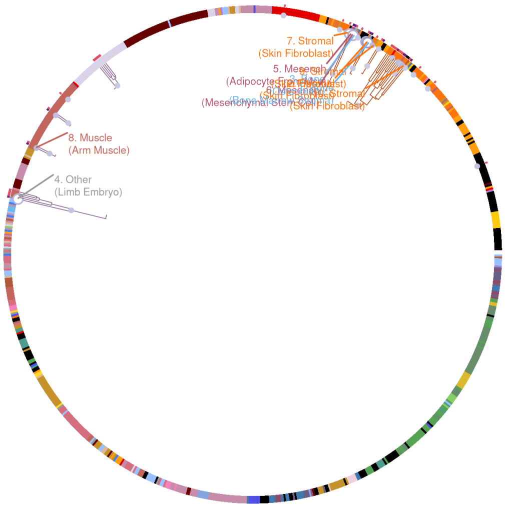

97,400 European ancestry women, 63,892 European ancestry men, 5,829 European ancestry individuals, 10,500 African American/Afro-Caribbean ancestry men, 2,706 African American/Afro-Caribbean ancestry women, 1,030 Indian Asian ancestry women, 7,648 Indian Asian ancestry men, 1,793 Filipino ancestry women, 2,944 Hispanic/Latino ancestry women, 1,764 Hispanic/Latino ancestry men

Waist circumference adjusted for BMI (joint analysis main effects and smoking interaction)

28443625

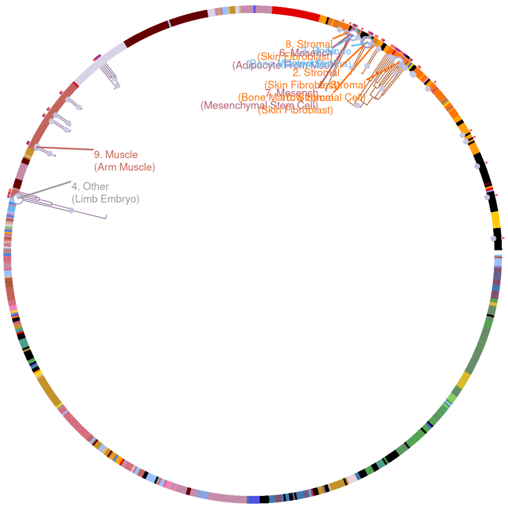

97,400 European ancestry women, 63,892 European ancestry men, 5,829 European ancestry individuals, 10,500 African American/Afro-Caribbean ancestry men, 2,706 African American/Afro-Caribbean ancestry women, 1,030 Indian Asian ancestry women, 7,648 Indian Asian ancestry men, 1,793 Filipino ancestry women, 2,944 Hispanic/Latino ancestry women, 1,764 Hispanic/Latino ancestry men

Waist circumference adjusted for BMI in non-smokers

28443625

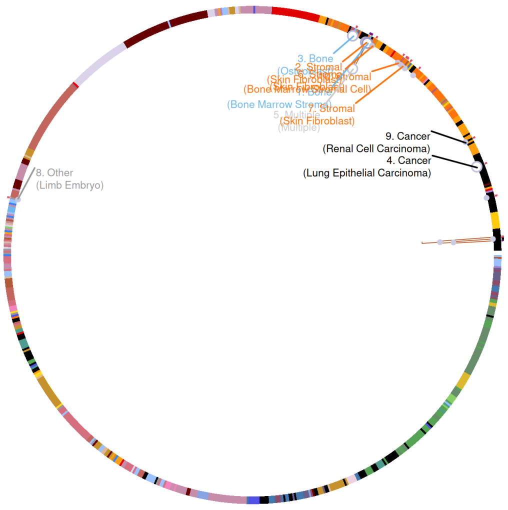

77,113 European ancestry women, 47,319 European ancestry men, 4,856 European ancestry individuals, 8,799 African American/Afro-Caribbean ancestry women, 1,803 African American/Afro-Caribbean ancestry men, 1,020 Indian Asian ancestry women, 6,691 Indian Asian ancestry men, 1,526 Filipino ancestry women, 2,469 Hispanic/Latino ancestry women, 1,318 Hispanic/Latino ancestry men

Waist circumference adjusted for BMI in smokers

28443625

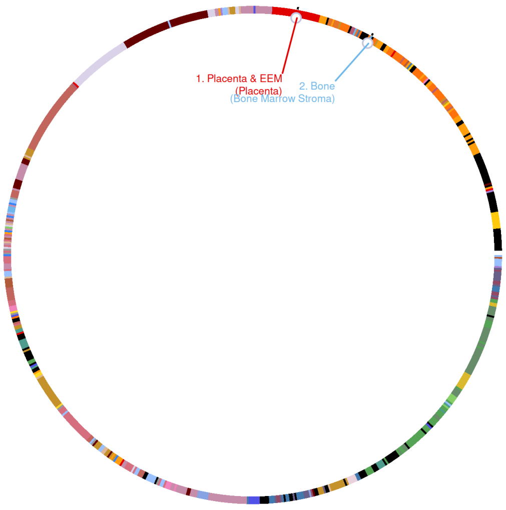

19,276 European ancestry women, 16,408 European ancestry men, 973 European ancestry individuals, 1,701 African American/Afro-Caribbean ancestry women, 903 African American/Afro-Caribbean ancestry men, 10 Indian Asian ancestry women, 957 Indian Asian ancestry men, 267 Filipino ancestry women, 475 Hispanic/Latino ancestry women, 446 Hispanic/Latino ancestry men

Waist-to-hip ratio adjusted for BMI (adjusted for smoking behaviour)

28443625

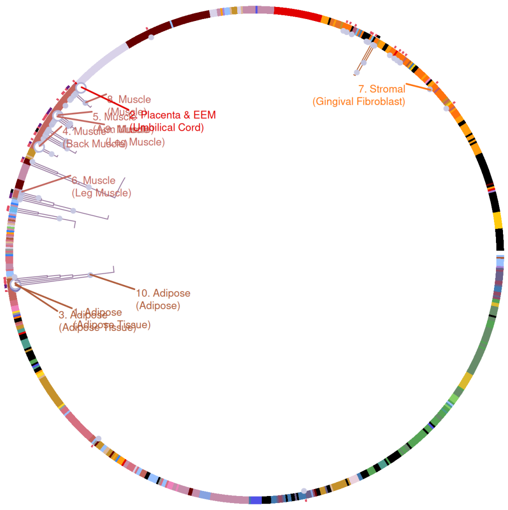

95,362 European ancestry women, 62,085 European ancestry men, 5,829 European ancestry individuals, 10,500 African American/Afro-Caribbean ancestry women, 2,706 African American/Afro-Caribbean ancestry men, 1,030 Indian Asian ancestry women, 7,648 Indian Asian ancestry men, 1,793 Filipino ancestry women, 2,944 Hispanic/Latino ancestry women, 1,764 Hispanic/Latino ancestry men

Waist-to-hip ratio adjusted for BMI (joint analysis main effects and smoking interaction)

28443625

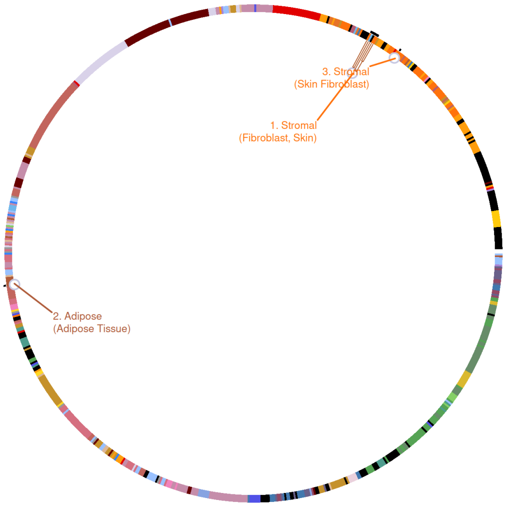

95,362 European ancestry women, 62,085 European ancestry men, 5,829 European ancestry individuals, 10,500 African American/Afro-Caribbean ancestry women, 2,706 African American/Afro-Caribbean ancestry men, 1,030 Indian Asian ancestry women, 7,648 Indian Asian ancestry men, 1,793 Filipino ancestry women, 2,944 Hispanic/Latino ancestry women, 1,764 Hispanic/Latino ancestry men

Waist-to-hip ratio adjusted for BMI in non-smokers

28443625

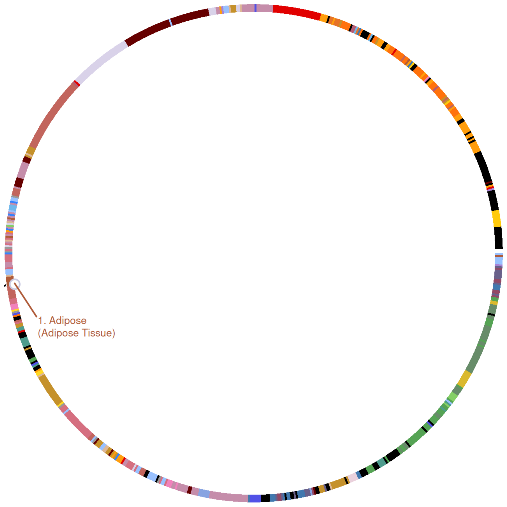

75,379 European ancestry women, 45,811 European ancestry men, 4,856 European ancestry individuals, 8,799 African American/Afro-Caribbean ancestry women, 1,803 African American/Afro-Caribbean ancestry men, 1,020 Indian Asian ancestry women, 6,691 Indian Asian ancestry men, 1,526 Filipino ancestry women, 2,469 Hispanic/Latino ancestry women, 1,318 Hispanic/Latino ancestry men

Waist circumference adjusted for BMI (joint analysis main effects and physical activity interaction)

28448500

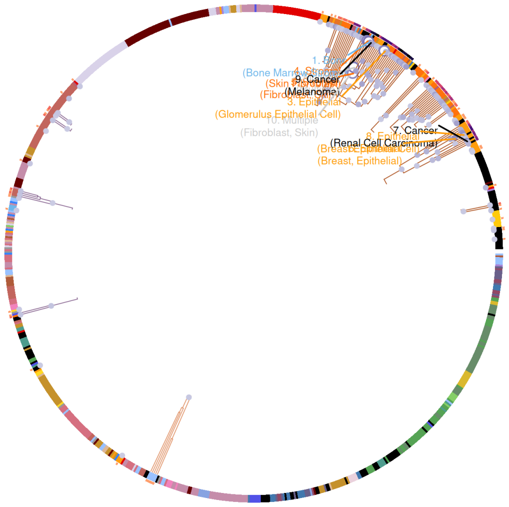

2,518 African American women, 85,730 European ancestry women, 1,773 Filipino ancestry women, 1,030 Indian ancestry women, 1,457 African American men, 54,792 European ancestry men, 7,648 Indian ancestry men, 5,527 European ancestry individuals

Waist circumference adjusted for BMI in active individuals

28448500

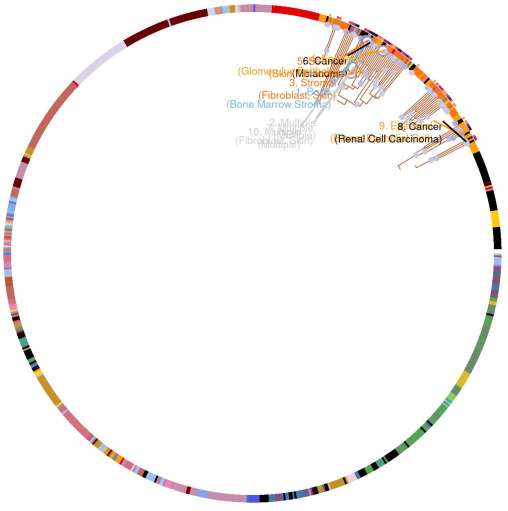

1,957 African American women, 1,773 Filipino ancestry women, 305 Indian ancestry women, 67,139 European ancestry women, 1,206 African American men, 2,029 Indian ancestry men, 43,100 European ancestry men, at least 4,819 European ancestry individuals

Waist circumference adjusted for body mass index

28448500

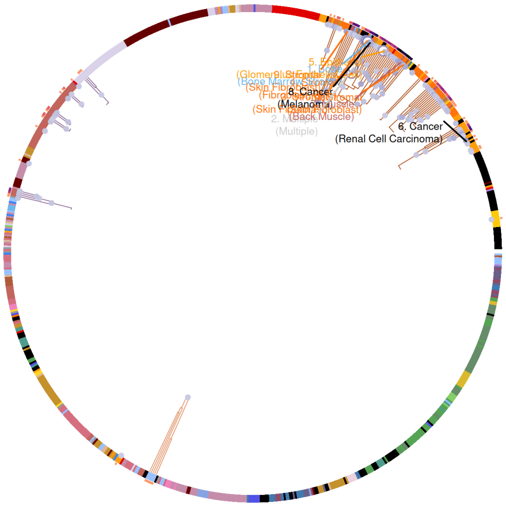

2,518 African American women, 85,730 European ancestry women, 1,773 Filipino ancestry women, 1,030 Indian ancestry women, 1,457 African American men, 54,792 European ancestry men, 7,648 Indian ancestry men, 5,527 European ancestry individuals

Waist-to-hip ratio adjusted for BMI (joint analysis for main effect and physical activity interaction)

28448500

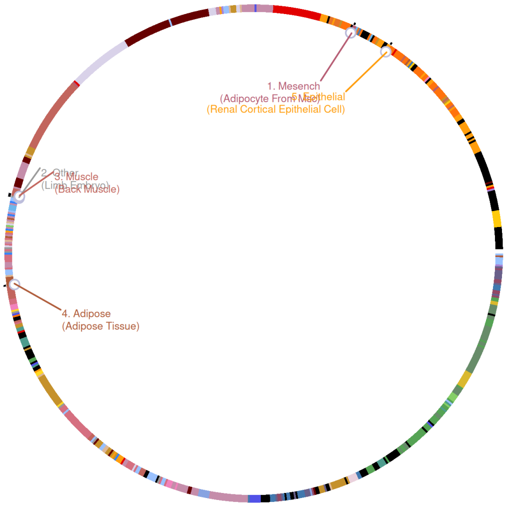

2,518 African American women, 83,707 European ancestry women, 1,773 Filipino ancestry women, 1,030 Indian ancestry women, 1,457 African American men, 52,993 European ancestry men, 7,648 Indian ancestry men, 5,527 European ancestry individuals

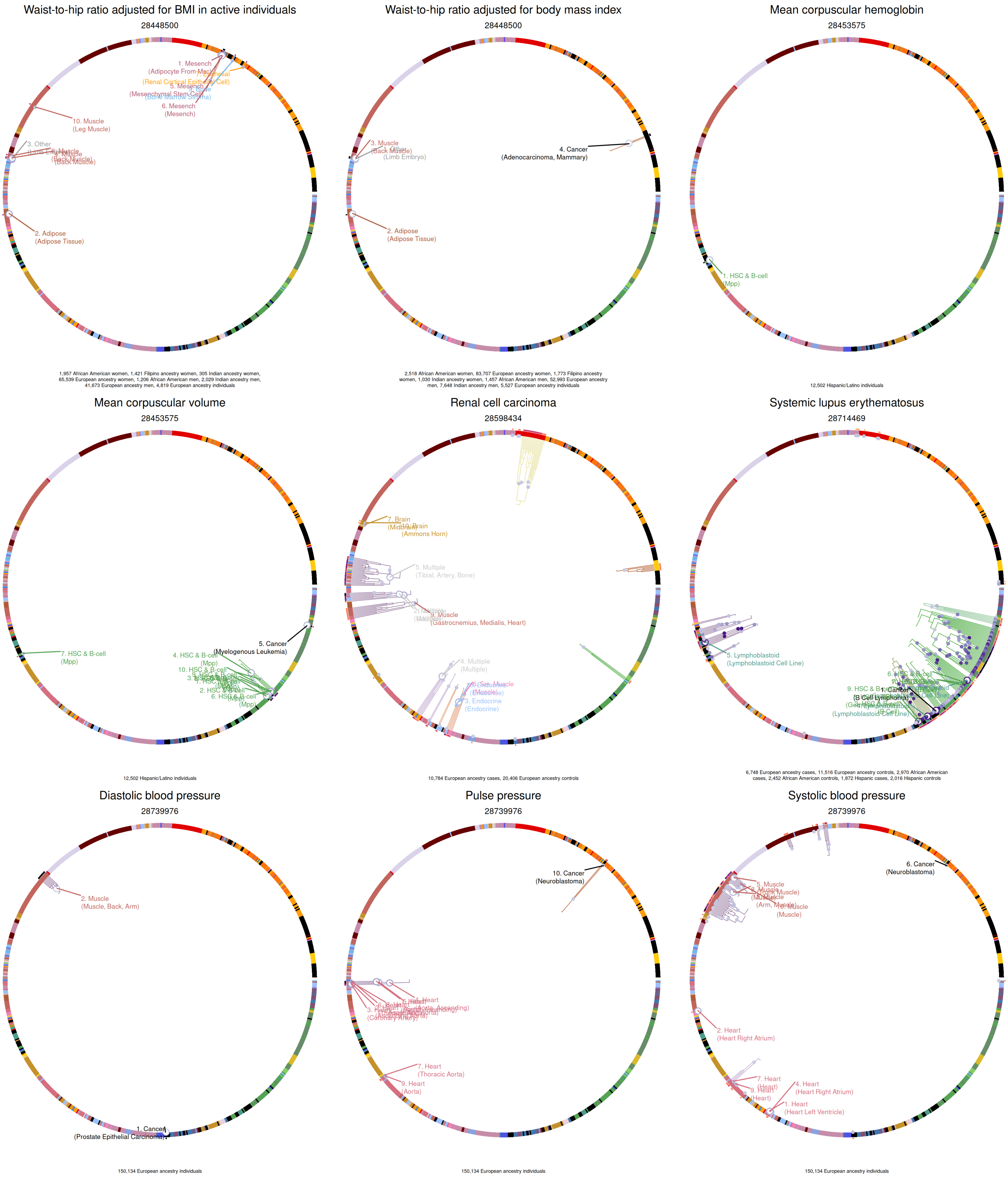
Waist-to-hip ratio adjusted for BMI in active individuals
28448500
1. Mesench
(Adipocyte From Mac)
(Renal Cortical Epithelial Cell)
(Mesenchymal Stem Cell)
6. Mesench
(Mesench)
10. Muscle
(Leg Muscle)
3. Other
2. Adipose
(Adipose Tissue)
1,957 African American women, 1,421 Filipino ancestry women, 305 Indian ancestry women, 65,539 European ancestry women, 1,206 African American men, 2,029 Indian ancestry men, 41,673 European ancestry men, 4,819 European ancestry individuals
Waist-to-hip ratio adjusted for body mass index
28448500
3. Muscle
(Limb Embryo)
4. Cancer
(Adenocarcinoma, Mammary)
2. Adipose
(Adipose Tissue)
2,518 African American women, 83,707 European ancestry women, 1,773 Filipino ancestry women, 1,030 Indian ancestry women, 1,457 African American men, 52,993 European ancestry men, 7,648 Indian ancestry men, 5,527 European ancestry individuals
Mean corpuscular hemoglobin
28453575
1. HSC & B-cell
(Mpp)
12,502 Hispanic/Latino individuals
Mean corpuscular volume
28453575
7. HSC & B-cell
(Mpp)
5. Cancer
(Myelogenous Leukemia)
4. HSC & B-cell
(Mpp)
10. HSC & B-cell
2. HSC & B-cell
6. HSC & B-cell
12,502 Hispanic/Latino individuals
Renal cell carcinoma
28598434
7. Brain
(Ammons Horn)
5. Multiple
(Tibial, Artery, Bone)
9. Muscle
(Gastrocnemius, Medialis, Heart)
4. Multiple
(Multiple)
3. Endocrine
(Endocrine)
10,784 European ancestry cases, 20,406 European ancestry controls
Systemic lupus erythematosus
28714469
5. Lymphoblastoid
(Lymphoblastoid Cell Line)
1. Cancer
(B Cell Lymphoma)
9. HSC & B-cell
(Lymphoblastoid Cell Line)
6,748 European ancestry cases, 11,516 European ancestry controls, 2,970 African American cases, 2,452 African American controls, 1,872 Hispanic cases, 2,016 Hispanic controls
Diastolic blood pressure
28739976
2. Muscle
(Muscle, Back, Arm)
1. Cancer
(Prostate Epithelial Carcinoma)
150,134 European ancestry individuals
Pulse pressure
28739976
10. Cancer
(Neuroblastoma)
4. Heart
(Coronary Artery)
7. Heart
(Thoracic Aorta)
9. Heart
(Aorta)
150,134 European ancestry individuals
Systolic blood pressure
28739976
6. Cancer
(Neuroblastoma)
5. Muscle
(Arm, Muscle)
10. Muscle
(Muscle)
2. Heart
(Heart Right Atrium)
7. Heart
(Heart)
9. Heart
(Heart)
4. Heart
(Heart Right Atrium)
1. Heart
(Heart Left Ventricle)
150,134 European ancestry individuals

P wave duration

28794112

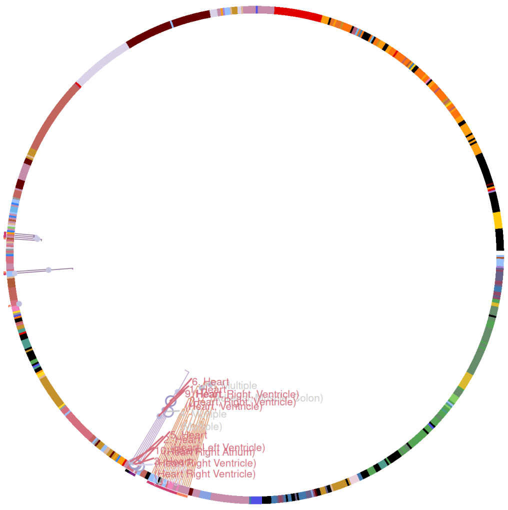

37,678 European ancestry individuals, 6,778 African ancestry individuals

Heel bone mineral density

28869591

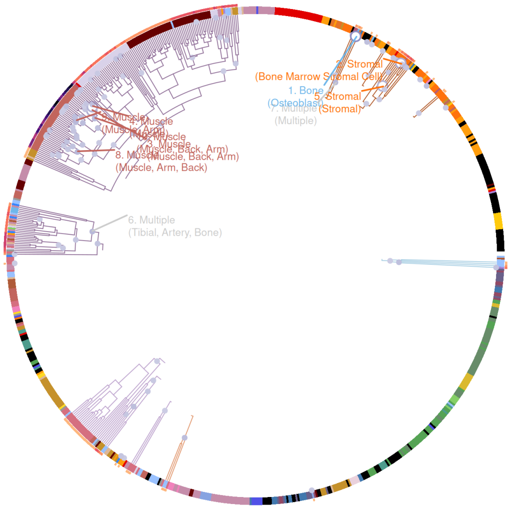

76,067 European ancestry women, 66,420 European ancestry men

Red cell distribution width

28957414

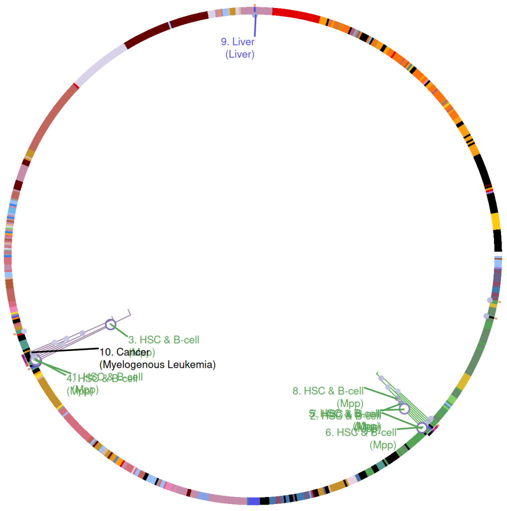

116,666 British ancestry individuals

Breast cancer (estrogen-receptor negative)

29058716

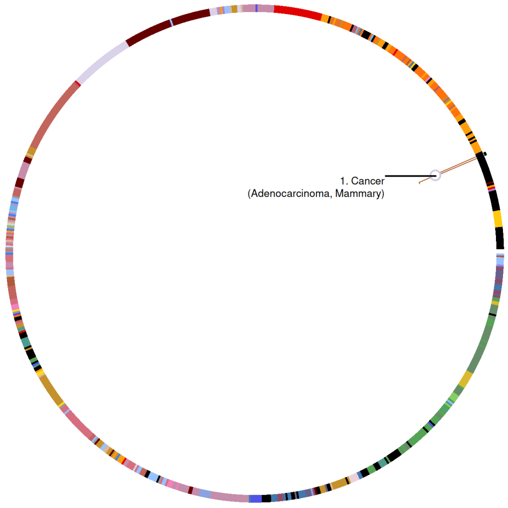

14,135 European ancestry cases, 58,126 European ancestry controls

Breast cancer

29059683

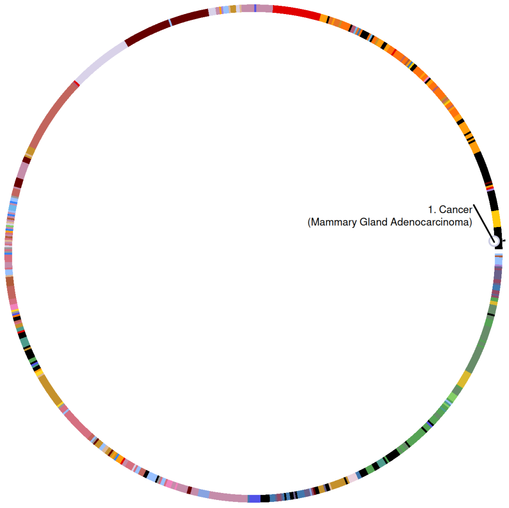

76,192 European ancestry cases, 63,082 European ancestry controls

Allergic disease (asthma, hay fever or eczema)

29083406

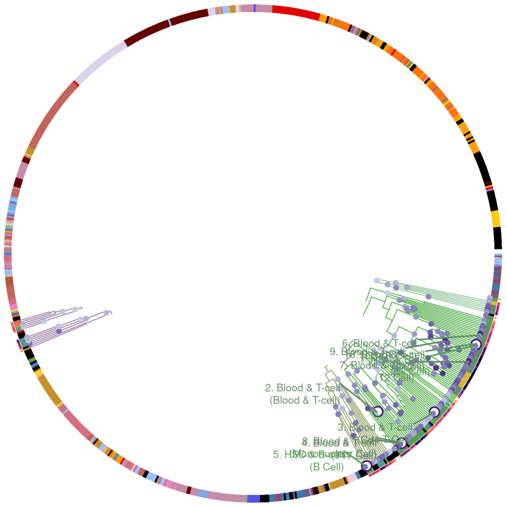

180,129 European ancestry cases, 180,709 European ancestry controls

Coronary artery disease

29212778

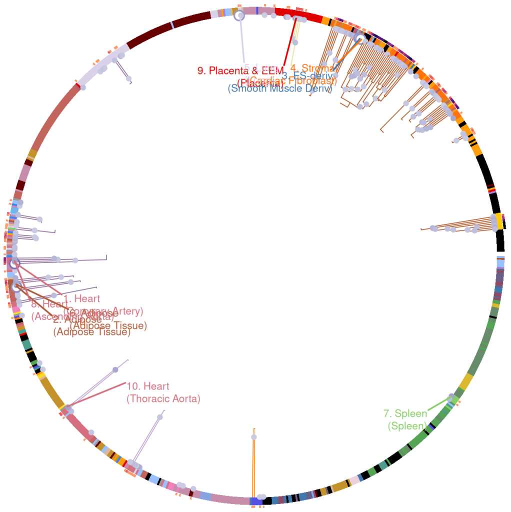

up to 122,733 cases, up to 424,528 controls

Intraocular pressure

29235454

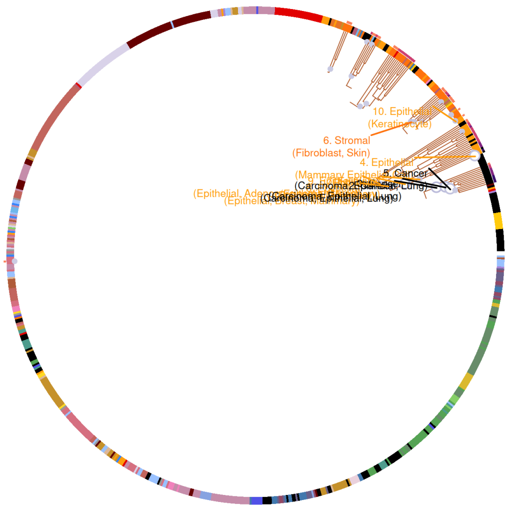

56,819 European ancestry individuals, 5,748 Hispanic/Latino individuals, 5,119 East Asian ancestry individuals, 2,070 African American individuals

Albumin-globulin ratio

29403010

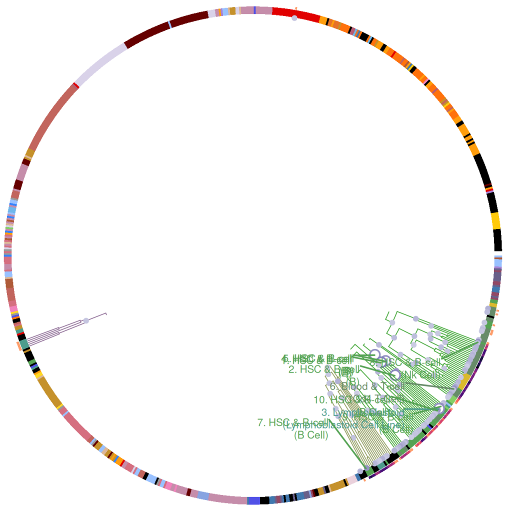

98,626 Japanese ancestry individuals

Blood urea nitrogen levels

29403010

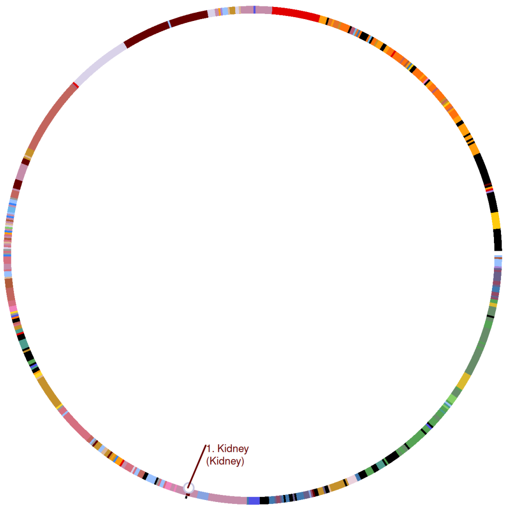

139,818 Japanese ancestry individuals

Creatine kinase levels

29403010

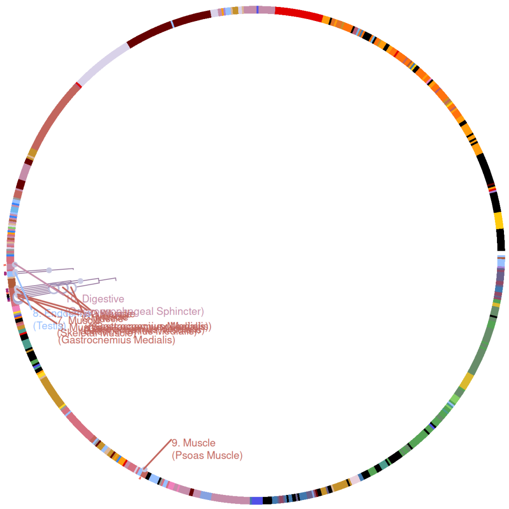

106,080 Japanese ancestry individuals

Gamma glutamyl transferase levels

29403010

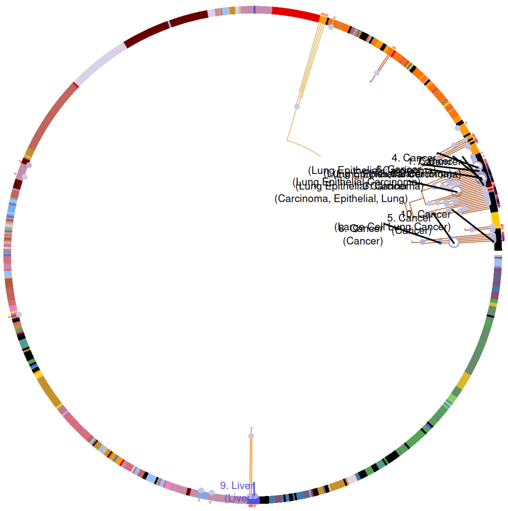

118,309 Japanese ancestry individuals

Glomerular filtration rate

29403010

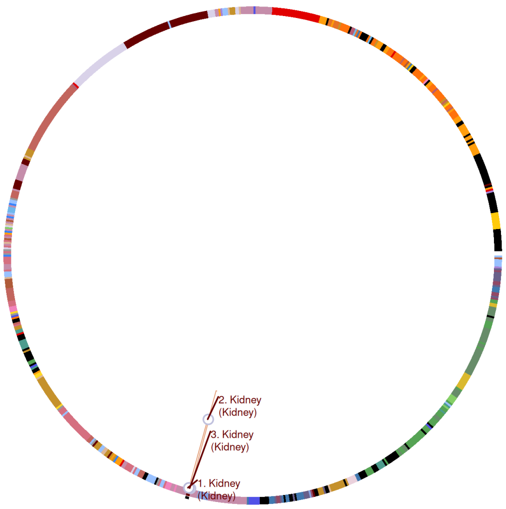

143,658 Japanese ancestry individuals

Hematocrit

29403010

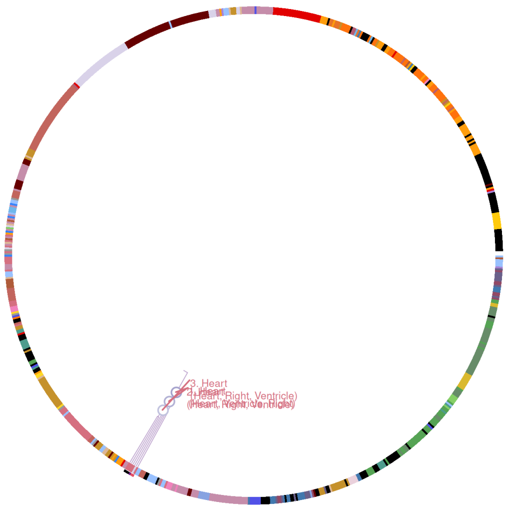

108,757 Japanese ancestry individuals

High density lipoprotein cholesterol levels

29403010

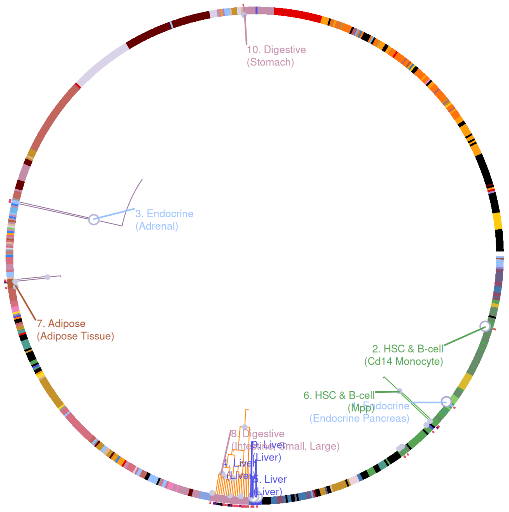

70,657 Japanese ancestry individuals

Low density lipoprotein cholesterol levels

29403010

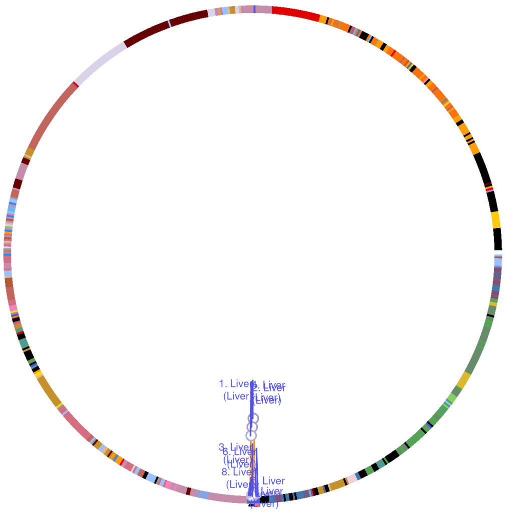

72,866 Japanese ancestry individuals

Mean corpuscular hemoglobin

29403010

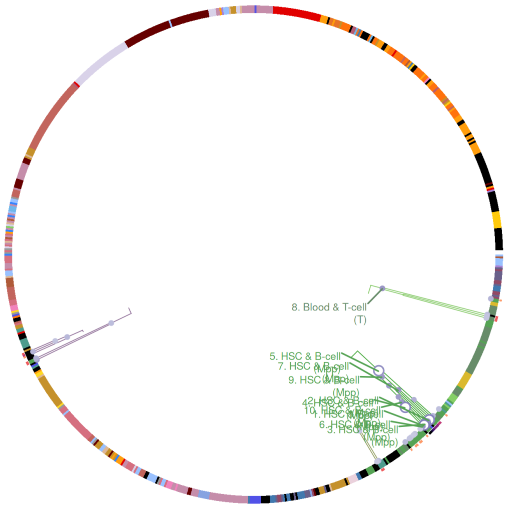

108,054 Japanese ancestry individuals

Mean corpuscular hemoglobin concentration

29403010

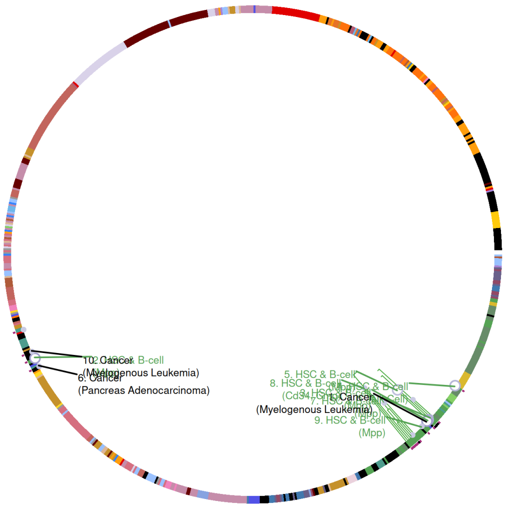

108,728 Japanese ancestry individuals

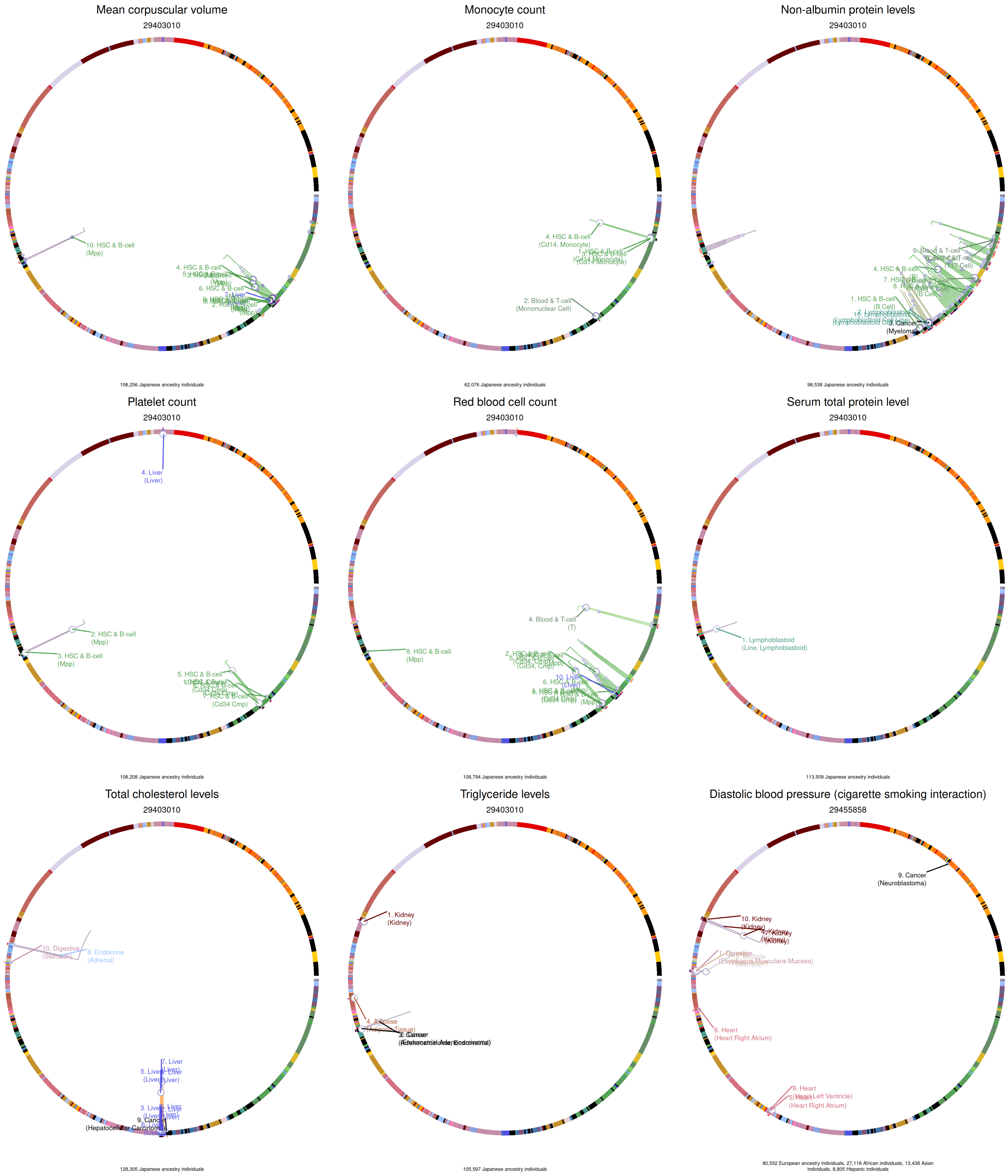

Mean corpuscular volume
29403010
10. HSC & B-cell
(Mpp)
4. HSC & B-cell
6. HSC & B-cell
7. Liver
108,256 Japanese ancestry individuals
Monocyte count
29403010
4. HSC & B-cell
(Cd14, Monocyte)
2. Blood & T-cell
(Mononuclear Cell)
62,076 Japanese ancestry individuals
Non-albumin protein levels
29403010
4. HSC & B-cell
1. HSC & B-cell
(B Cell)
3. Cancer
(Myeloma)
98,538 Japanese ancestry individuals
Platelet count
29403010
4. Liver
(Liver)
2. HSC & B-cell
(Mpp)
3. HSC & B-cell
(Mpp)
5. HSC & B-cell
7. HSC & B-cell
(Cd34 Cmp)
108,208 Japanese ancestry individuals
Red blood cell count
29403010
4. Blood & T-cell
(T)
8. HSC & B-cell
(Mpp)
10. Liver
(Liver)
108,794 Japanese ancestry individuals
Serum total protein level
29403010
1. Lymphoblastoid
(Line, Lymphoblastoid)
113,509 Japanese ancestry individuals
Total cholesterol levels
29403010
10. Digestive
(Stomach)
8. Endocrine
(Adrenal)
7. Liver
5. Liver
(Liver)
9. Cancer
(Hepatocellular Carcinoma)
128,305 Japanese ancestry individuals
Triglyceride levels
29403010
1. Kidney
(Kidney)
4. Adipose
(Adipose Tissue)
105,597 Japanese ancestry individuals
Diastolic blood pressure (cigarette smoking interaction)
29455858
9. Cancer
(Neuroblastoma)
10. Kidney
(Kidney)
6. Heart
(Heart Right Atrium)
8. Heart
(Heart Left Ventricle)
(Heart Right Atrium)
80,552 European ancestry individuals, 27,118 African individuals, 13,438 Asian individuals, 8,805 Hispanic individuals

Systolic blood pressure (cigarette smoking interaction)

29455858

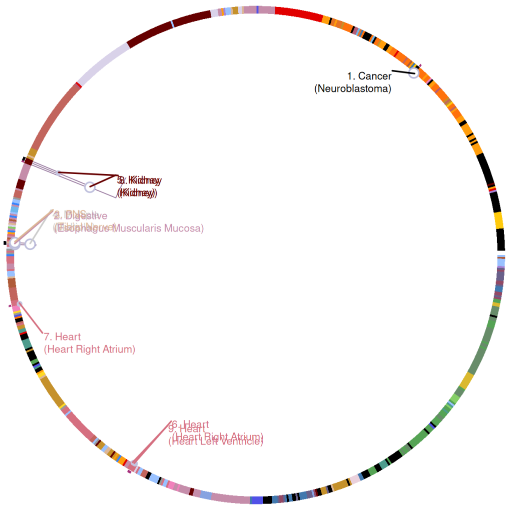

80,552 European ancestry individuals, 27,118 African individuals, 13,438 Asian individuals, 8,805 Hispanic individuals

High density lipoprotein cholesterol levels

29507422

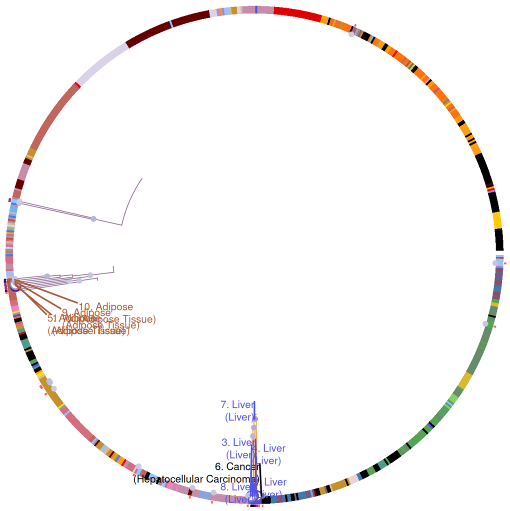

265,204 European ancestry individuals, 7,795 Hispanic individuals, 6,855 East Asian ancestry individuals, 2,958 African American individuals, 439 South Asian ancestry individuals

Low density lipoprotein cholesterol levels

29507422

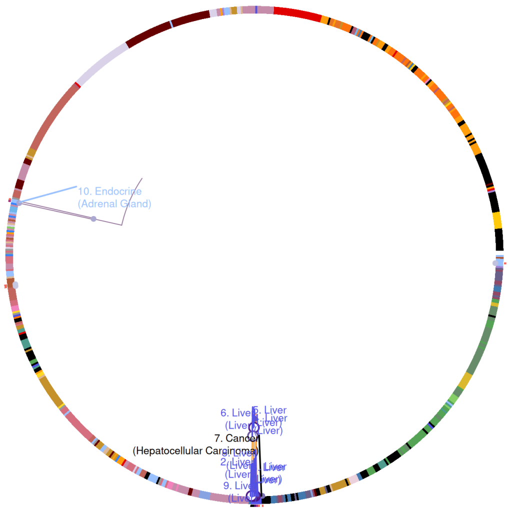

265,204 European ancestry individuals, 7,795 Hispanic individuals, 6,855 East Asian ancestry individuals, 2,958 African American individuals, 439 South Asian ancestry individuals

Total cholesterol levels

29507422

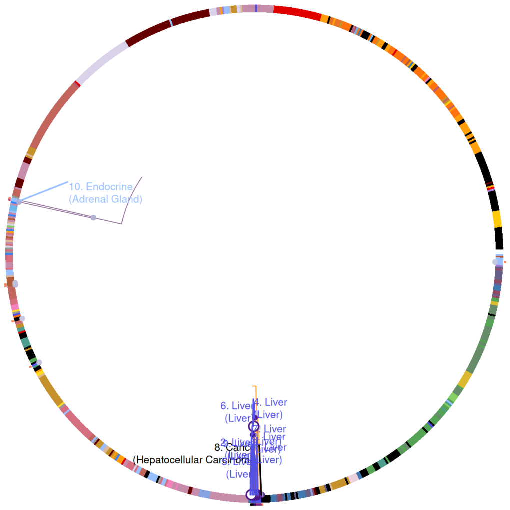

265,204 European ancestry individuals, 7,795 Hispanic individuals, 6,855 East Asian ancestry individuals, 2,958 African American individuals, 439 South Asian ancestry individuals

Triglycerides

29507422

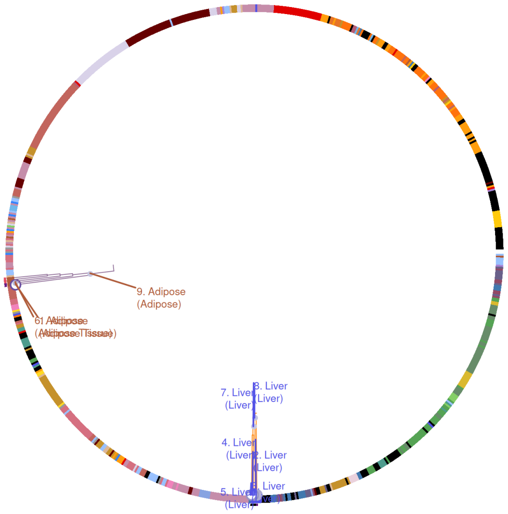

265,204 European ancestry individuals, 7,795 Hispanic individuals, 6,855 East Asian ancestry individuals, 2,958 African American individuals, 439 South Asian ancestry individuals

Ischemic stroke

29531354

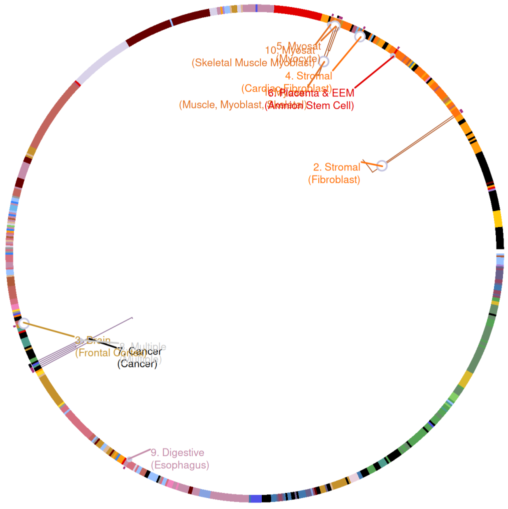

34,217 European ancestry cases, 406,111 European ancestry controls, 17,369 East Asian ancestry cases, 28,195 East Asian ancestry controls, 2,437 South Asian ancestry cases, 6,707 South Asian ancestry controls, 5,541 African American cases, 15,146 African American controls, 222 Asian ancestry cases, 333 Asian ancestry controls, 555 Latin American cases, 692 Latin American controls

Ischemic stroke (small-vessel)

29531354

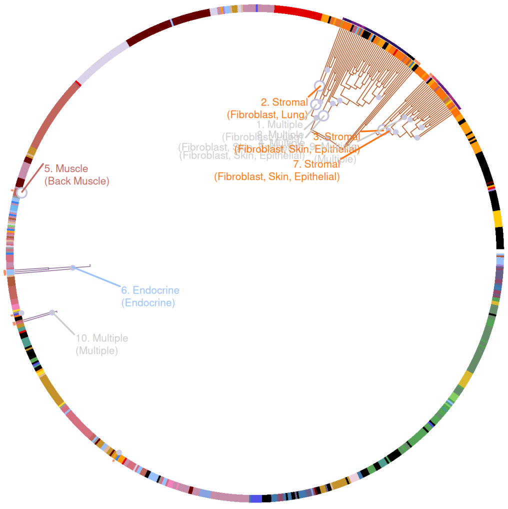

5,386 European ancestry cases, 192,662 European ancestry controls, 5,096 East Asian ancestry cases, 28,195 East Asian ancestry controls, 314 South Asian ancestry cases, 6,707 South Asian ancestry controls, 694 African American cases, 15,146 African American controls, 134 Asian ancestry cases, 333 Asian ancestry controls, 86 Latin American cases, 692 Latin American controls

Stroke

29531354

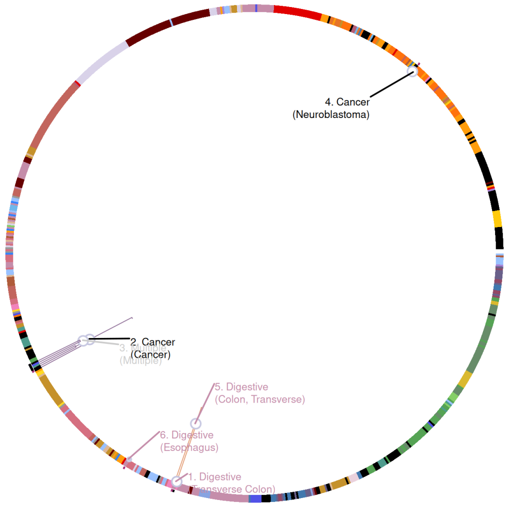

40,585 European ancestry cases, 406,111 European ancestry controls, 17,369 East Asian ancestry cases, 28,195 East Asian ancestry controls, 2,437 South Asian ancestry cases, 6,707 South Asian ancestry controls, 5,541 African American cases, 15,154 African American controls, 365 Asian ancestry cases, 333 Asian ancestry controls, 865 Latin American cases, 692 Latin American controls

Intraocular pressure

29617998

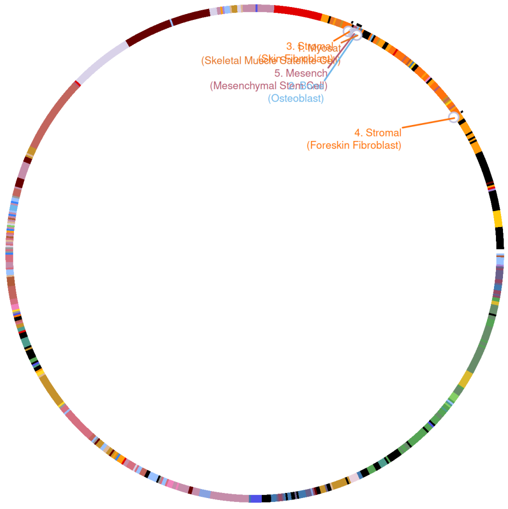

115,486 European ancestry individuals

Alzheimer's disease or family history of Alzheimer's disease

29777097

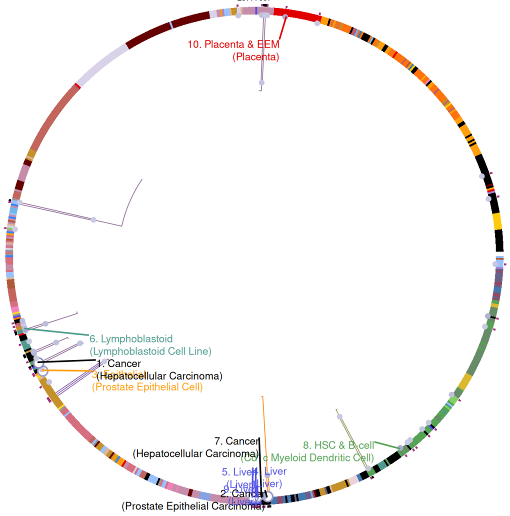

up to 42,034 British ancestry individuals with parental history of Alzheimer's disease, at least 272,244 British ancestry individuals with no parental history of Alzheimer's disease, 25,580 Alzheimer's disease cases, 48,466 controls

Family history of Alzheimer's disease

29777097

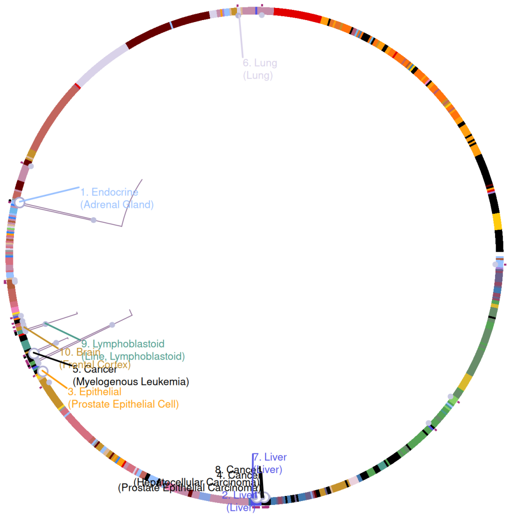

up to 42,034 British ancestry individuals with parental history of Alzheimer's disease, at least 272,244 European ancestry individuals with no parental history of Alzheimer's disease

Intraocular pressure

29785010

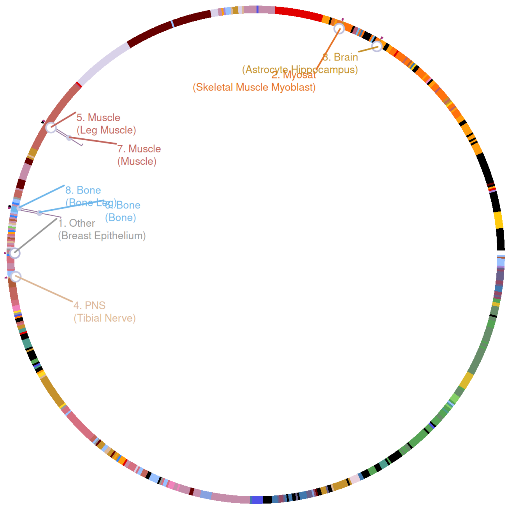

139,555 European ancestry individuals

Allergic disease (asthma, hay fever or eczema)

29785011

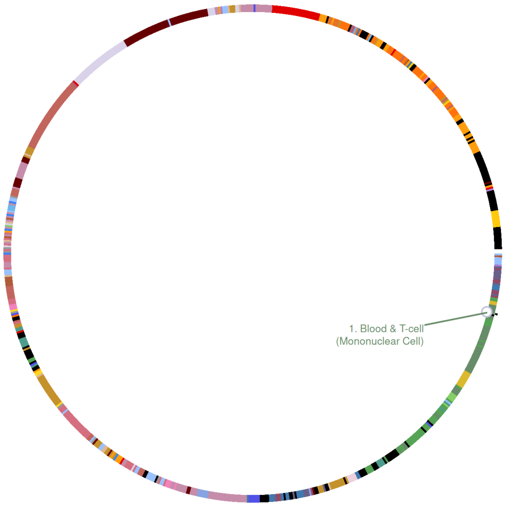

25,685 European ancestry allergic diseases cases, 76,768 European ancestry controls

Asthma or allergic disease (pleiotropy)

29785011

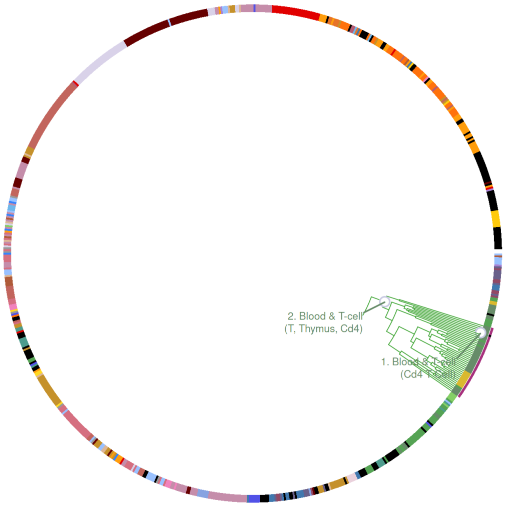

14,085 European asthma cases, 25,685 European ancestry allergic diseases cases, 76,768 European ancestry controls

Atrial fibrillation

29892015

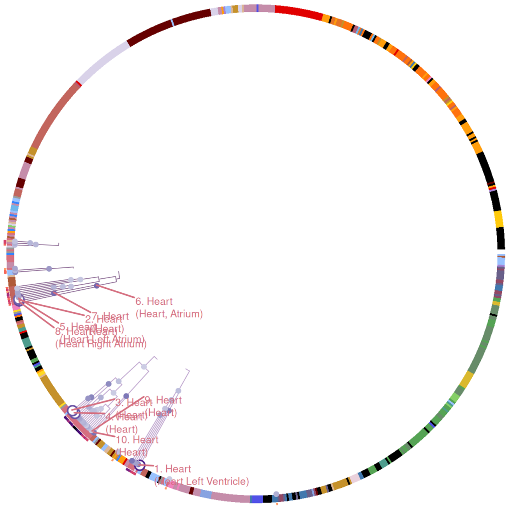

55,114 European ancestry cases, 482,295 European ancestry controls, 8,180 Japanese ancestry cases, 28,612 Japanese ancestry controls, 1,307 African American ancestry cases, 7,660 African American ancestry controls, 845 Hispanic cases, 4,177 Hispanic controls

Mean arterial pressure x alcohol consumption (light vs heavy) interaction (2df test)

29912962

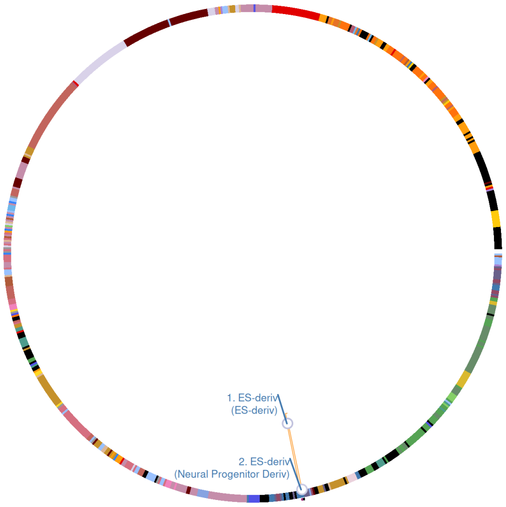

56,705 European ancestry heavy and light drinkers, 9,725 African American or Afro-Caribbean heavy and light drinkers, 1,789 Asian ancestry heavy and light drinkers, 4,812 Hispanic or Latin American heavy and light drinkers

Colorectal cancer

29917119

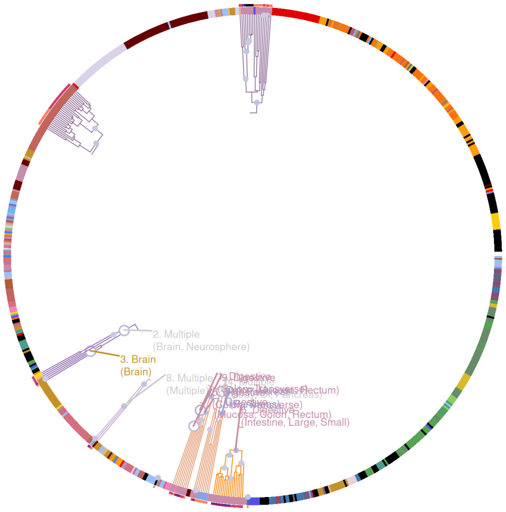

36,948 European ancestry cases, 30,864 European ancestry controls

Depressed affect

29942085

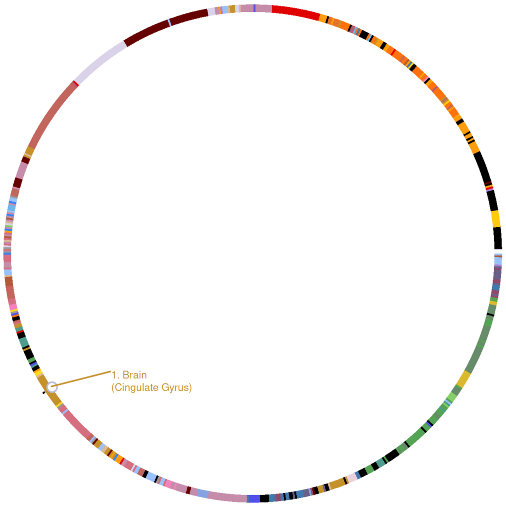

357,957 European ancestry individuals

Allergic rhinitis
30013184

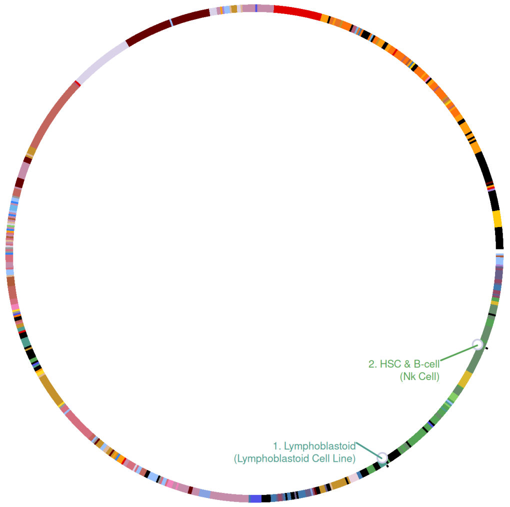

59,762 European and unknown ancestry cases, 152,358 European and unknown ancestry controls

PR interval
30046033

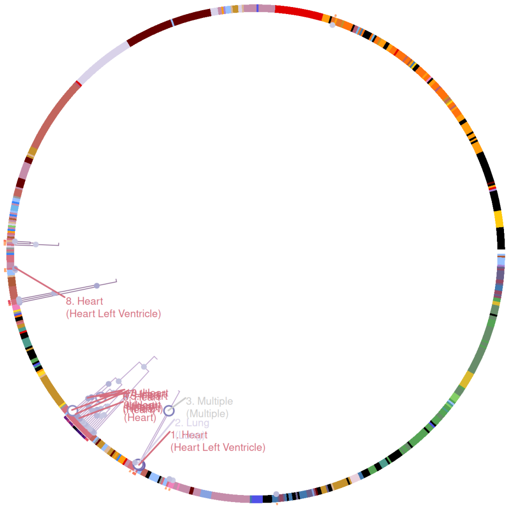

92,340 European ancestry individuals

Heel bone mineral density
30048462

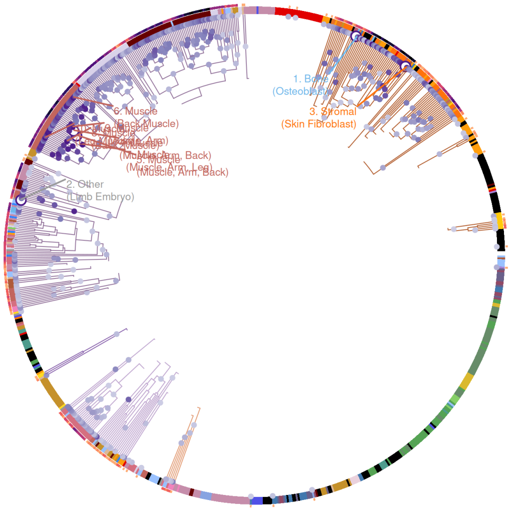

394,929 European ancestry individuals

Glaucoma
30054594

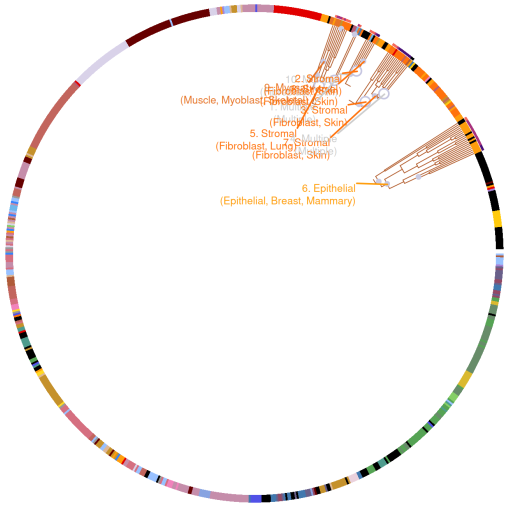

11,018 European ancestry cases, 126,068 European ancestry controls

Intraocular pressure
30054594

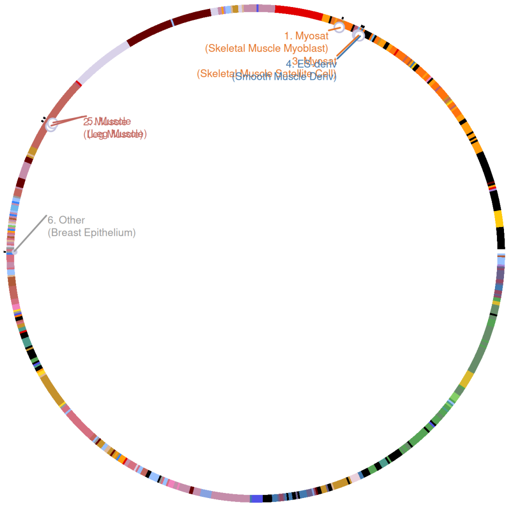

133,492 European ancestry individuals

Atrial fibrillation
30061737

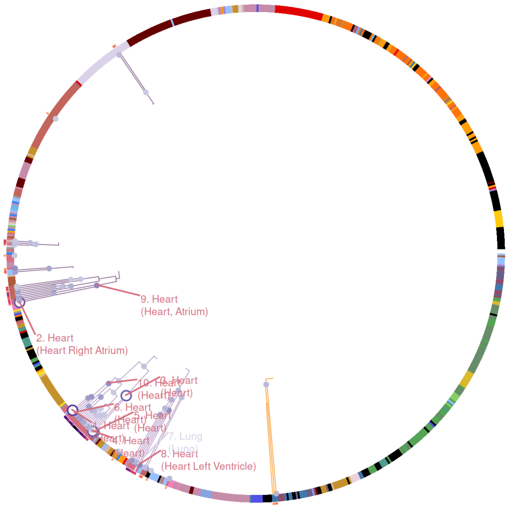

60,620 European ancestry cases, 970,216 European ancestry controls

Pulse pressure
30224653

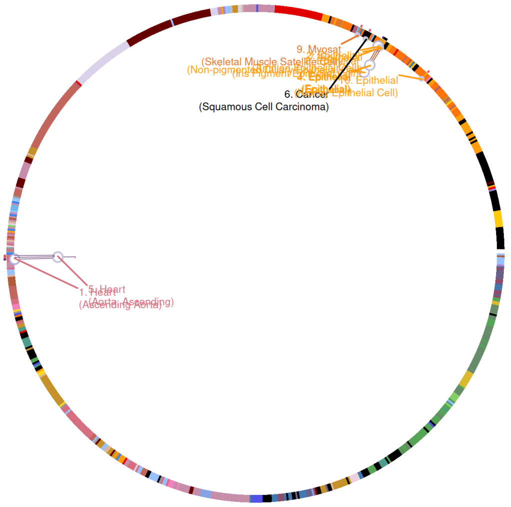

757,601 European ancestry individuals

HDL cholesterol
30275531

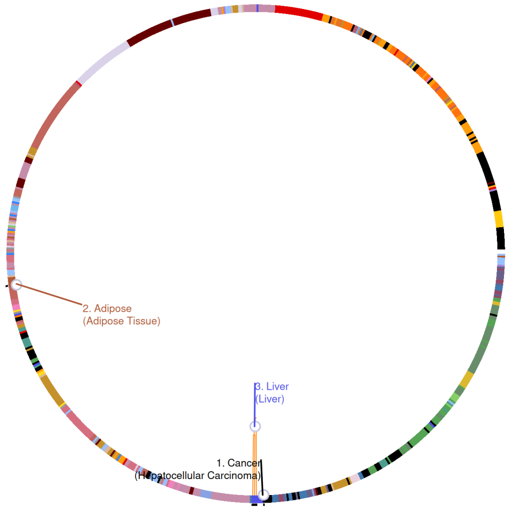

215,551 European ancestry individuals, 57,332 African American individuals, 24,743 Hispanic individuals

LDL cholesterol
30275531

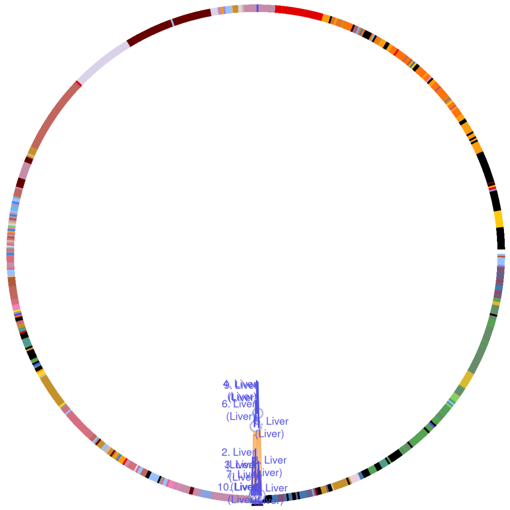

215,551 European ancestry individuals, 57,332 African American individuals, 24,743 Hispanic individuals

Total cholesterol levels

30275531

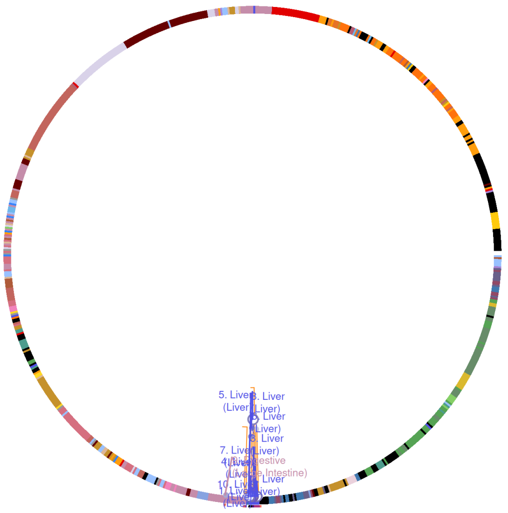

215,551 European ancestry individuals, 57,332 African American individuals, 24,743 Hispanic individuals

Triglycerides

30275531

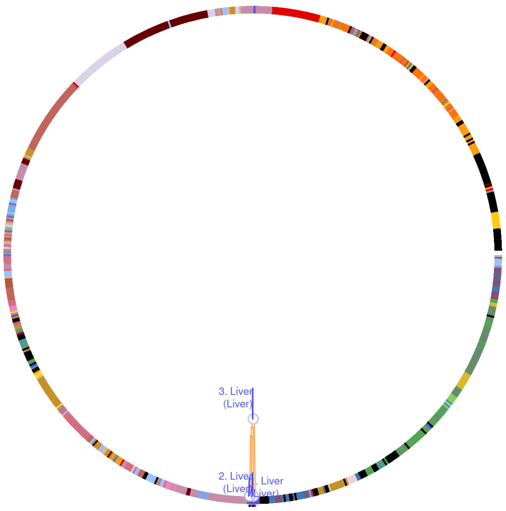

215,551 European ancestry individuals, 57,332 African American individuals, 24,743 Hispanic individuals

Lipid traits (pleiotropy) (HIPO component 1)

30289880

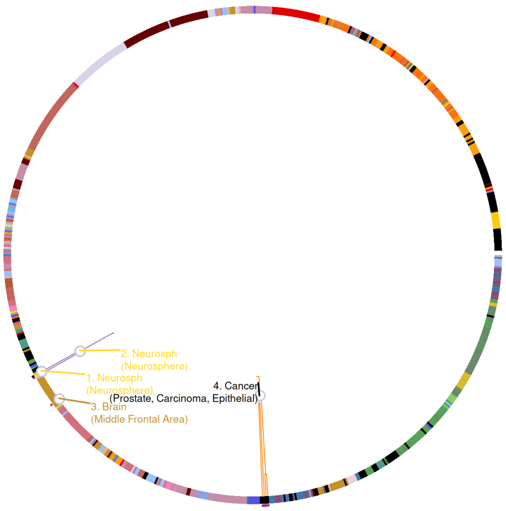

188,577 European ancestry individuals

C-reactive protein levels

30388399

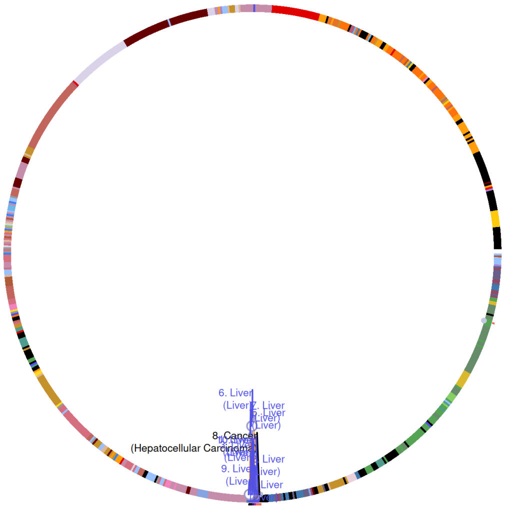

up to 92,771 European ancestry males, up to 113,387 European ancestry females

Nevus count

30429480

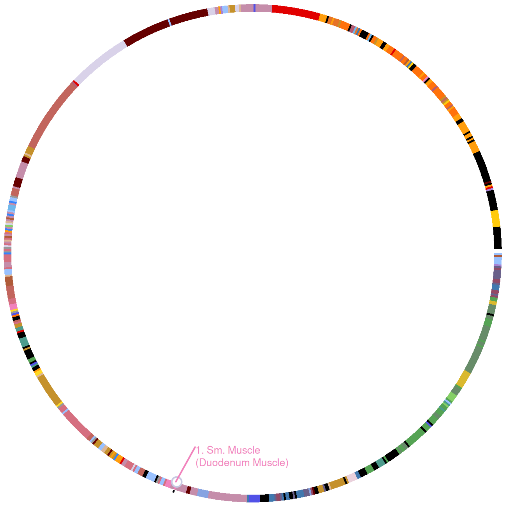

52,506 European ancestry individuals

Colorectal cancer

30529582

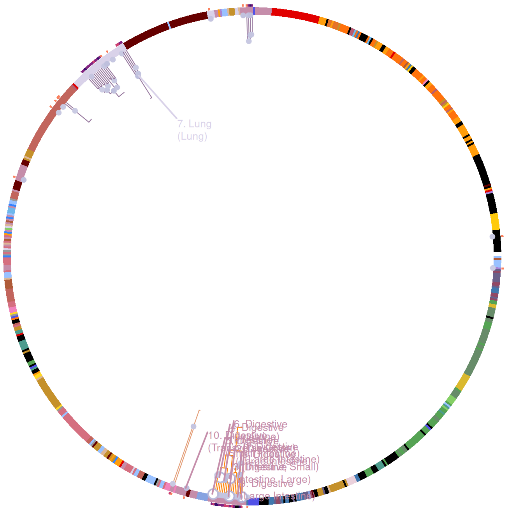

22,775 East Asian ancestry cases, 47,731 East Asian ancestry controls

Blond vs. brown/black hair color

30531825

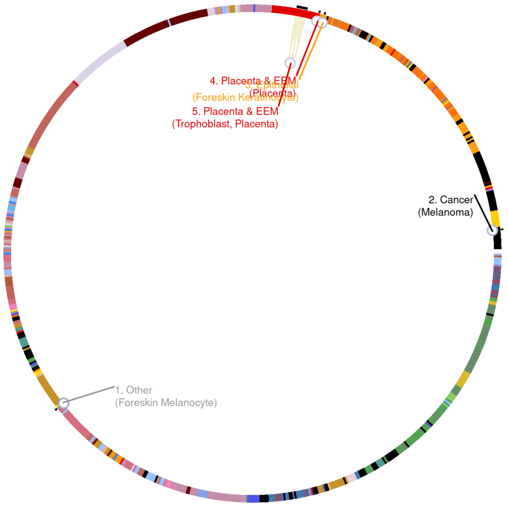

39,397 British ancestry blond hair individuals, 283,920 British ancestry brown or black hair individuals

Male-pattern baldness

30573740

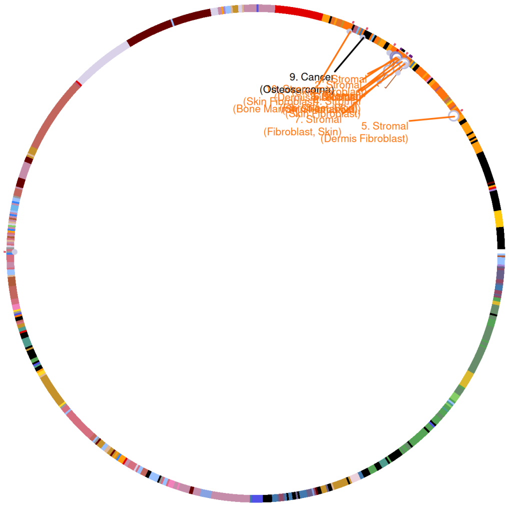

205,327 European ancestry males

Diastolic blood pressure

30578418

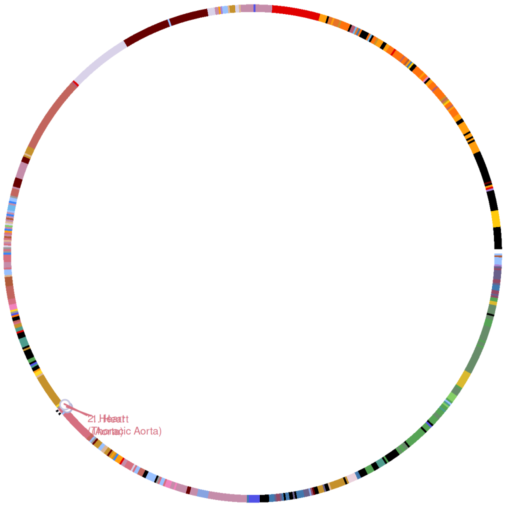

365,998 European ancestry individuals, 63,490 African ancestry individuals, 22,802 Hispanic individuals, 4,792 Asian ancestry individuals, 2,695 Native American ancestry individuals

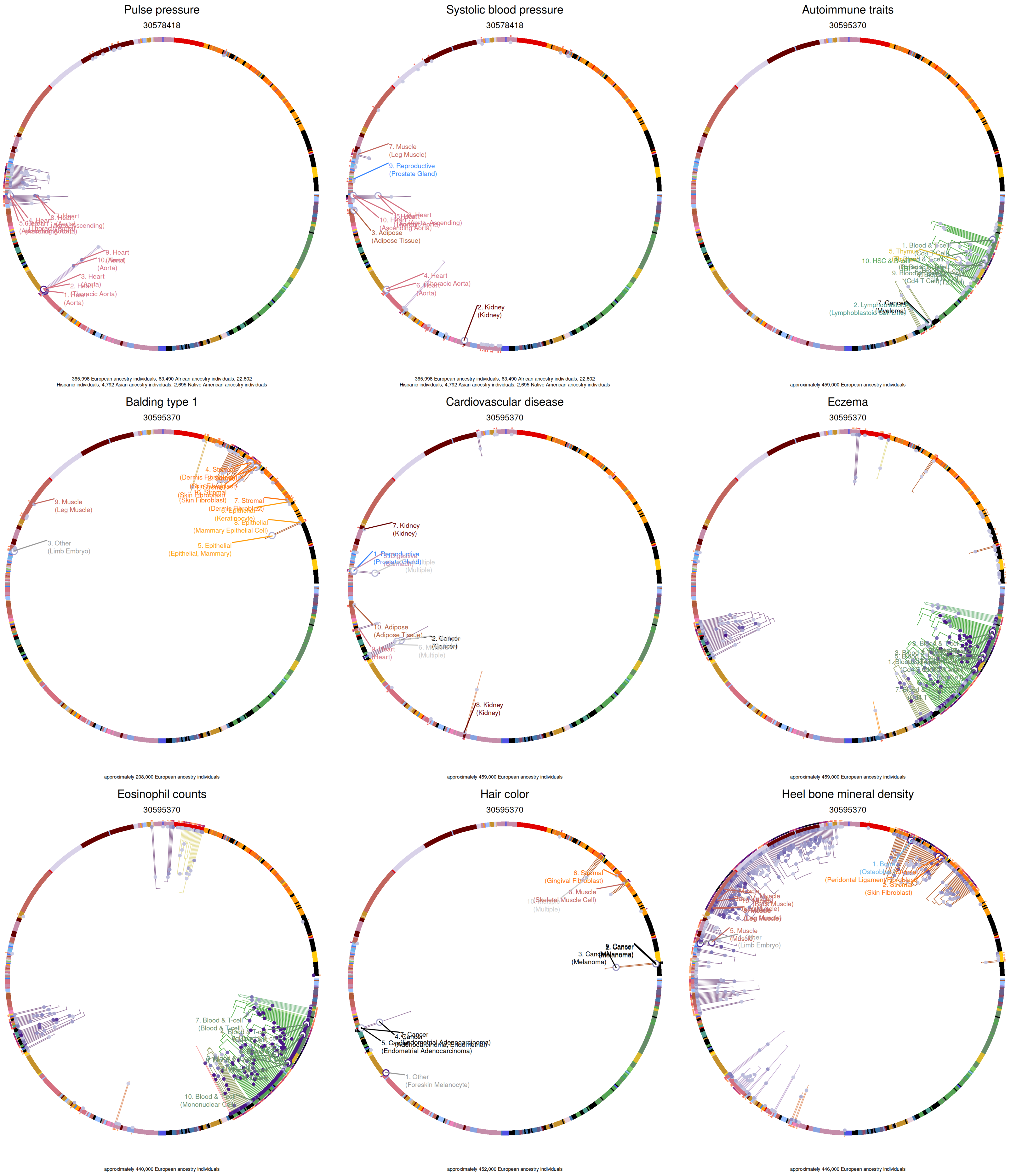

Pulse pressure
30578418
9. Heart
10. (Aorta)
(Aorta)
3. Heart
2. Heart
(Aorta)
(Thoracic Aorta)
1. Heart
(Aorta)
365,998 European ancestry individuals, 63,490 African ancestry individuals, 22,802 Hispanic individuals, 4,792 Asian ancestry individuals, 2,695 Native American ancestry individuals
Systolic blood pressure
30578418
7. Muscle
(Leg Muscle)
9. Reproductive
(Prostate Gland)
3. Adipose
(Adipose Tissue)
4. Heart
(Thoracic Aorta)
6. Heart
(Aorta)
2. Kidney
(Kidney)
365,998 European ancestry individuals, 63,490 African ancestry individuals, 22,802 Hispanic individuals, 4,792 Asian ancestry individuals, 2,695 Native American ancestry individuals
Autoimmune traits
30595370
1. Blood & T-cell
10. HSC & B-cell
7. Cancer
(Myeloma)
2. Lymphoblastoid
(Lymphoblastoid Cell Line)
approximately 459,000 European ancestry individuals
Balding type 1
30595370
9. Muscle
(Leg Muscle)
3. Other
(Limb Embryo)
4. Stromal
7. Stromal
(Dermis Fibroblast)
(Keratinocyte)
8. Epithelial
(Mammary Epithelial Cell)
5. Epithelial
(Epithelial, Mammary)
approximately 208,000 European ancestry individuals
Cardiovascular disease
30595370
7. Kidney
(Kidney)
10. Adipose
(Adipose Tissue)
2. Cancer
(Cancer)
9. Heart
(Heart)
6. Multiple
(Multiple)
8. Kidney
(Kidney)
approximately 459,000 European ancestry individuals
Eczema
30595370
8. Blood & T-cell
approximately 459,000 European ancestry individuals
Eosinophil counts
30595370
7. Blood & T-cell
(Blood & T-cell)
10. Blood & T-cell
(Mononuclear Cell)
approximately 440,000 European ancestry individuals
Hair color
30595370
6. Stromal
(Gingival Fibroblast)
8. Muscle
(Skeletal Muscle Cell)
(Multiple)
9. Cancer
(Melanoma)
3. Cancer
(Melanoma)
7. Cancer
5. Cancer
(Endometrial Adenocarcinoma)
1. Other
(Foreskin Melanocyte)
approximately 452,000 European ancestry individuals
Heel bone mineral density
30595370
1. Bone
(Peridontal Ligament Fibroblast)
2. Stromal
(Skin Fibroblast)
(Back Muscle)
(Leg Muscle)
5. Muscle
(Limb Embryo)
approximately 446,000 European ancestry individuals

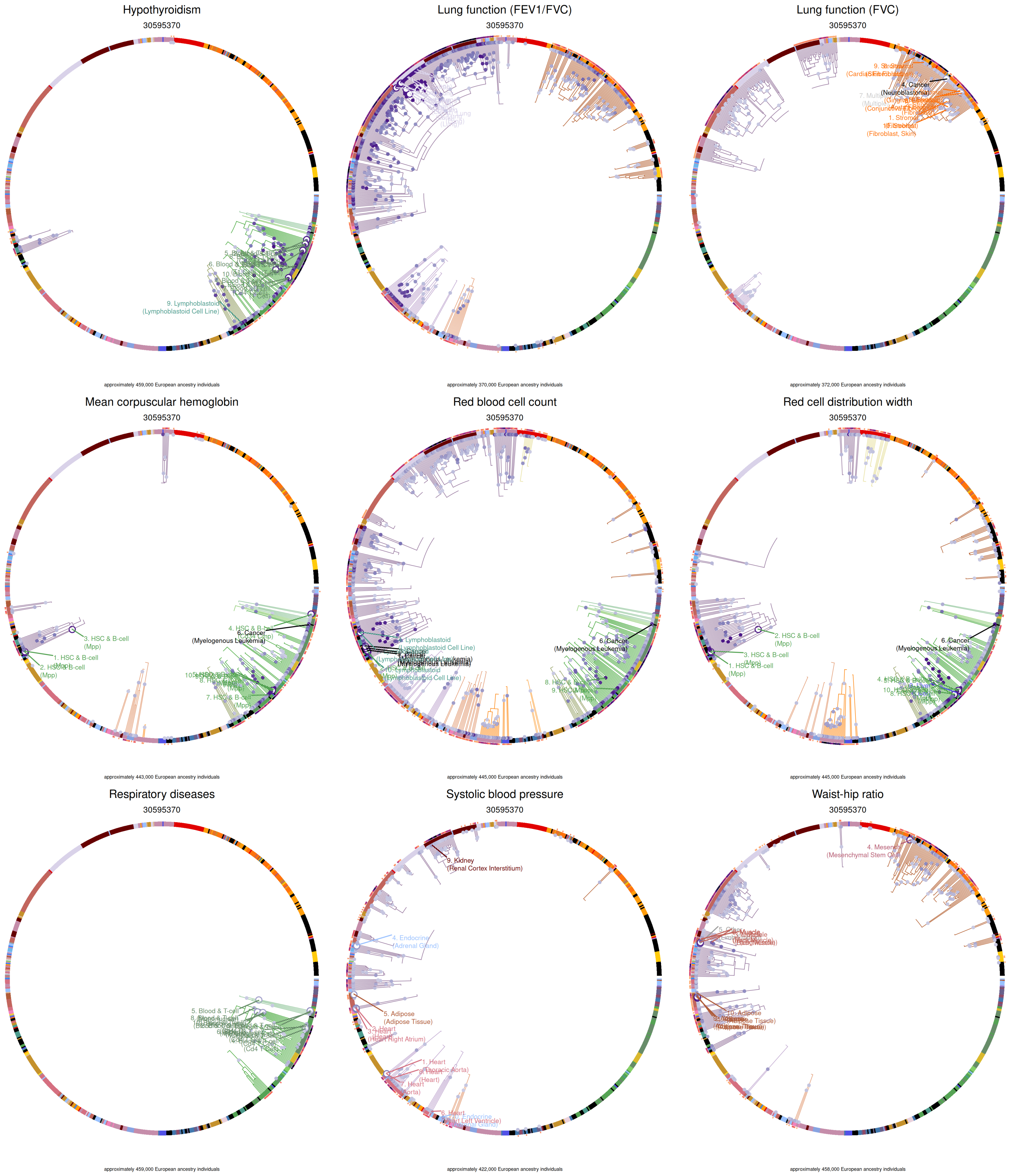
Hypothyroidism
30595370
9. Lymphoblastoid
(Lymphoblastoid Cell Line)
approximately 459,000 European ancestry individuals
Lung function (FEV1/FVC)
30595370
approximately 370,000 European ancestry individuals
Lung function (FVC)
30595370
4. Cancer
(Neuroblastoma)
(Fibroblast, Skin)
approximately 372,000 European ancestry individuals
Mean corpuscular hemoglobin
30595370
3. HSC & B-cell
(Mpp)
1. HSC & B-cell
2. HSC & B-cell
(Mpp)
6. Cancer
(Myelogenous Leukemia)
7. HSC & B-cell
(Mpp)
approximately 443,000 European ancestry individuals
Red blood cell count
30595370
6. Cancer
(Myelogenous Leukemia)
approximately 445,000 European ancestry individuals
Red cell distribution width
30595370
2. HSC & B-cell
(Mpp)
3. HSC & B-cell
1. HSC & B-cell
(Mpp)
6. Cancer
(Myelogenous Leukemia)
approximately 445,000 European ancestry individuals
Respiratory diseases
30595370
5. Blood & T-cell
approximately 459,000 European ancestry individuals
Systolic blood pressure
30595370
9. Kidney
(Renal Cortex Interstitium)
4. Endocrine
(Adrenal Gland)
5. Adipose
(Adipose Tissue)
1. Heart
(Thoracic Aorta)
6. Heart
(Heart Left Ventricle)
approximately 422,000 European ancestry individuals
Waist-hip ratio
30595370
4. Mesench
(Mesenchymal Stem Cell)
approximately 458,000 European ancestry individuals

White blood cell count

30595370

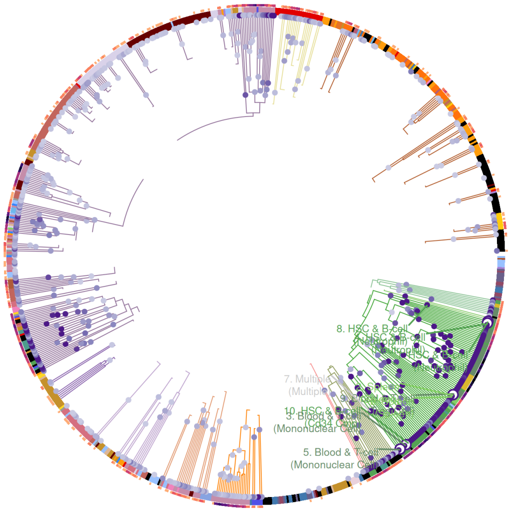

approximately 444,000 European ancestry individuals

Heel bone mineral density

30598549

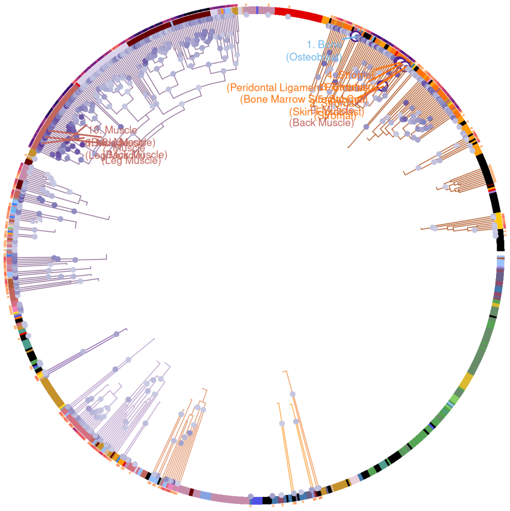

426,824 British ancestry individuals

Estimated glomerular filtration rate

30604766

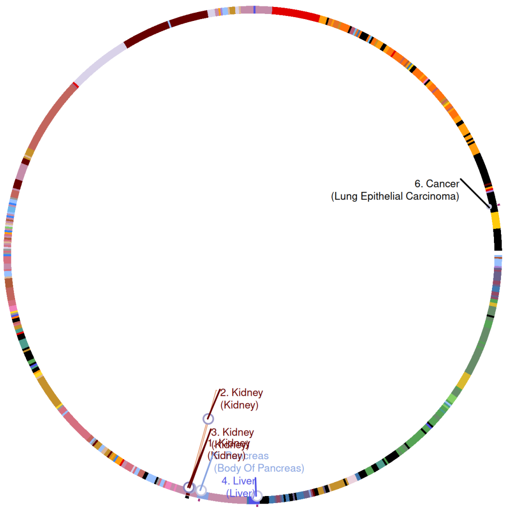

134,070 European ancestry individuals, 143,658 East Asian ancestry individuals, 26,344 Hispanic or Latino ancestry individuals, 8,224 African American individuals

Alzheimer's disease (late onset)

30617256

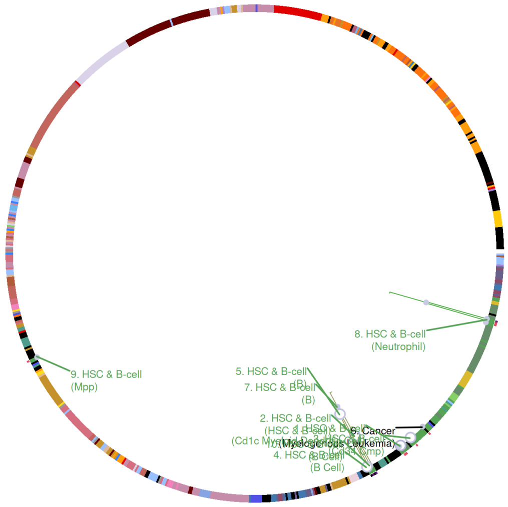

24,087 European ancestry cases, 55,058 European ancestry controls

Alzheimer's disease or family history of Alzheimer's disease

30617256

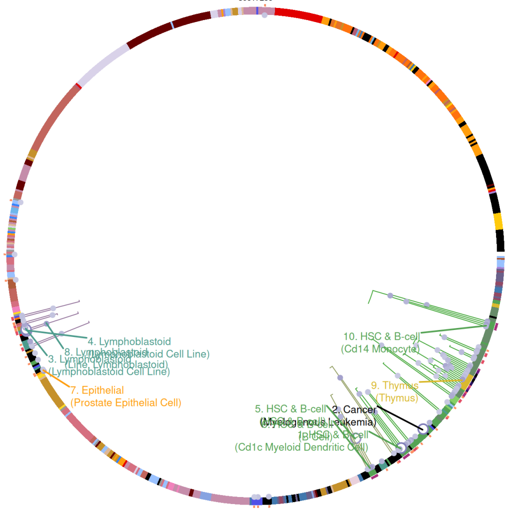

24,087 European ancestry late-onset Alzheimer's disease cases, 47,793 European ancestry individuals with family history of Alzheimer's disease, 383,378 European ancestry controls

Family history of Alzheimer's disease

30617256

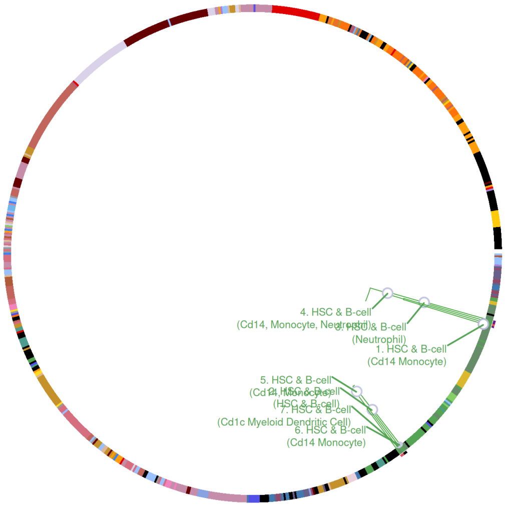

47,793 European ancestry cases, 328,320 European ancestry controls

Body fat distribution (leg fat ratio)

30664634

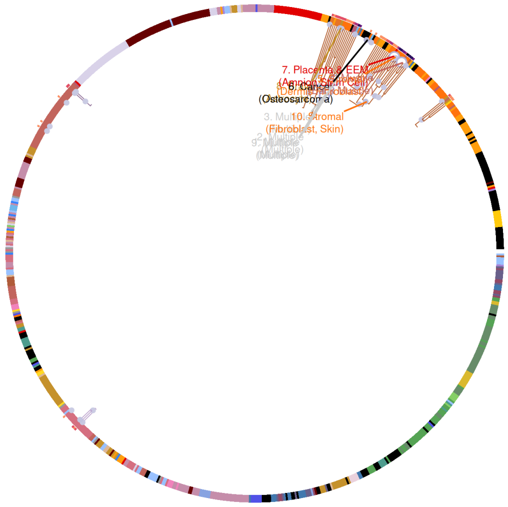

55,006 British ancestry males, 61,132 British ancestry females

Body fat distribution (trunk fat ratio)

30664634

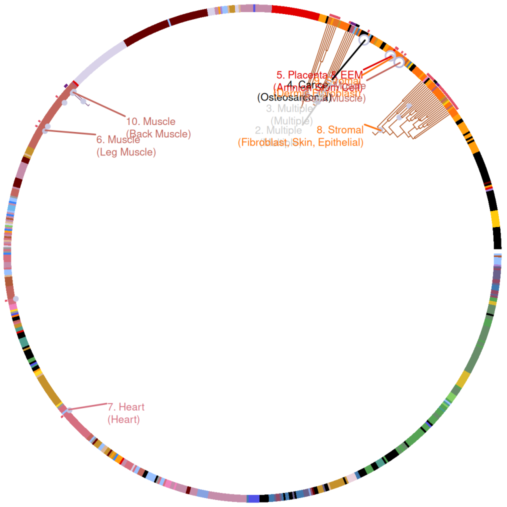

55,006 British ancestry males, 61,132 British ancestry females

HDL cholesterol x physical activity interaction (2df test)

30670697

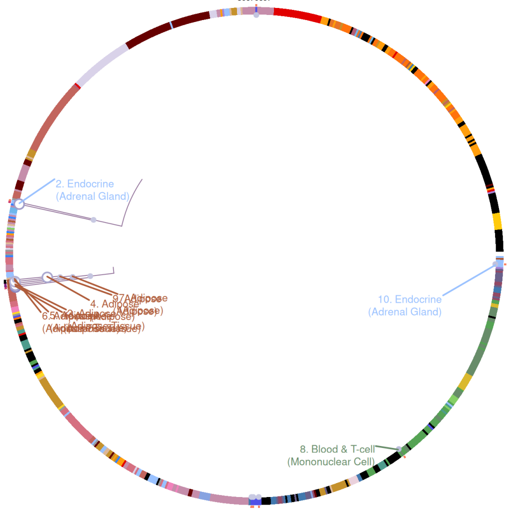

7,395 African American inactive individuals, 13,092 African American active individuals, 2,753 Asian ancestry inactive individuals, 3,650 Asian ancestry active individuals, 22,963 European ancestry inactive individuals, 61,939 European ancestry active individuals, 2,829 Hispanic or Latin American inactive individuals, 6,358 Hispanic or Latin American active individuals

Triglycerides x physical activity interaction (2df test)

30670697

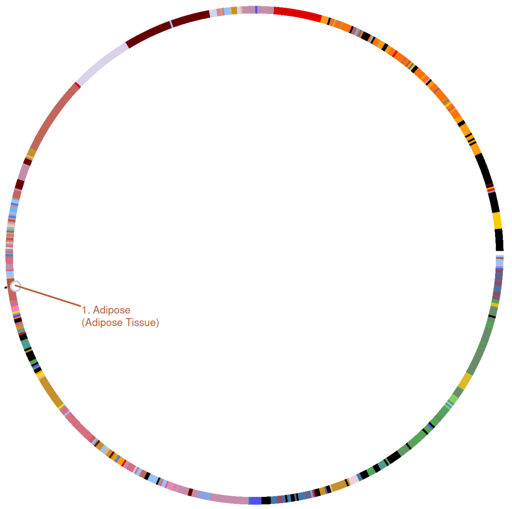

7,294 African American inactive individuals, 12,832 African American active individuals, 1,352 Asian ancestry inactive individuals, 3,049 Asian ancestry active individuals, 20,622 European ancestry inactive individuals, 51,480 European ancestry active individuals, 2,166 Hispanic or Latin American inactive individuals, 3,486 Hispanic or Latin American active individuals

PR interval

30679814

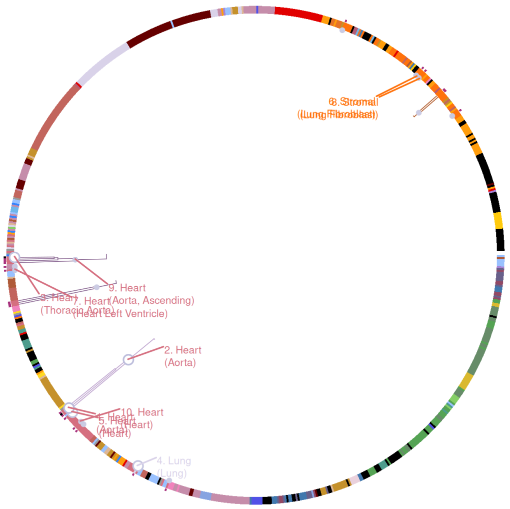

2,370 Erasmus Rucphen Family (founder/genetic isolate) individuals, 29,325 European ancestry individuals

Waist-to-hip ratio adjusted for BMI (additive genetic model)

30778226

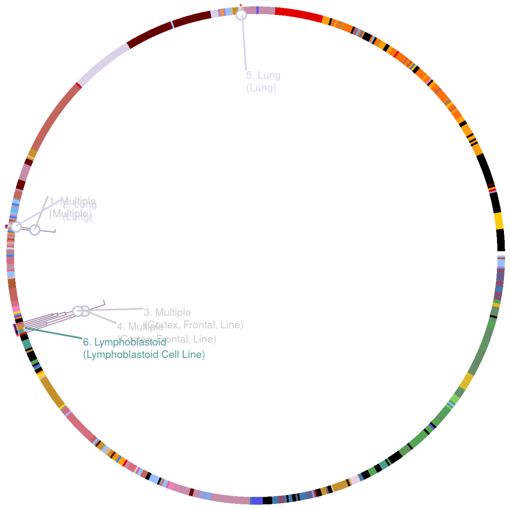

288,492 European ancestry individuals, up to 15,687 African American individuals, up to 29,315 South Asian ancestry individuals, up to 6,800 East Asian ancestry individuals, up to 4,075 Hispanic ancestry individuals

FEV1

30804560

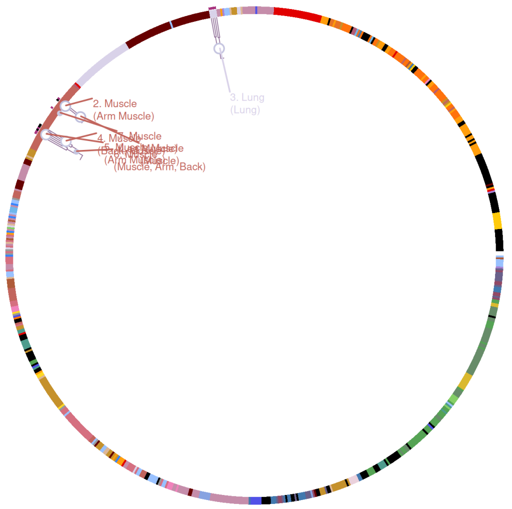

321,047 European ancestry individuals

Lung function (FEV1/FVC)

30804560

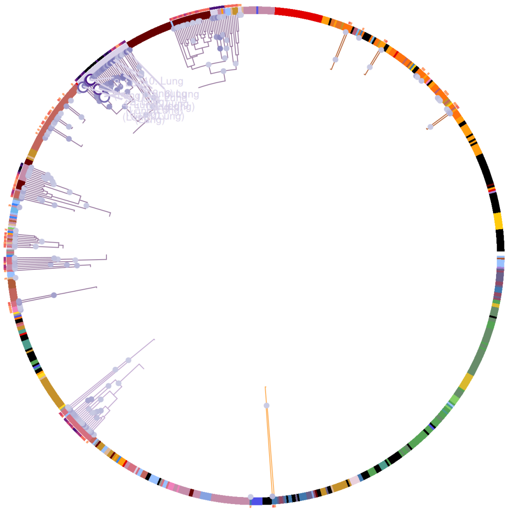

321,047 European ancestry individuals

Peak expiratory flow

30804560

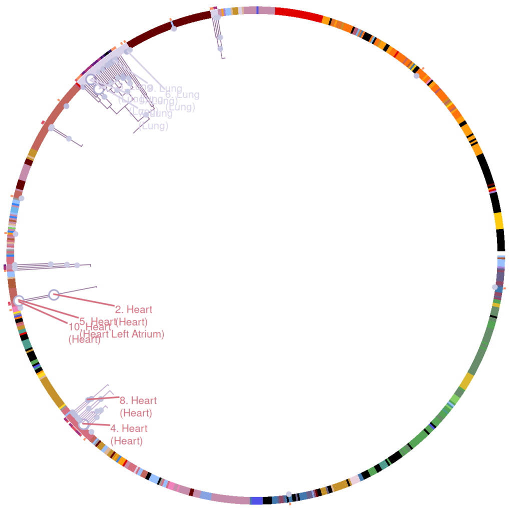

321,047 European ancestry individuals

Chronic obstructive pulmonary disease or high blood pressure (pleiotropy)

30940143

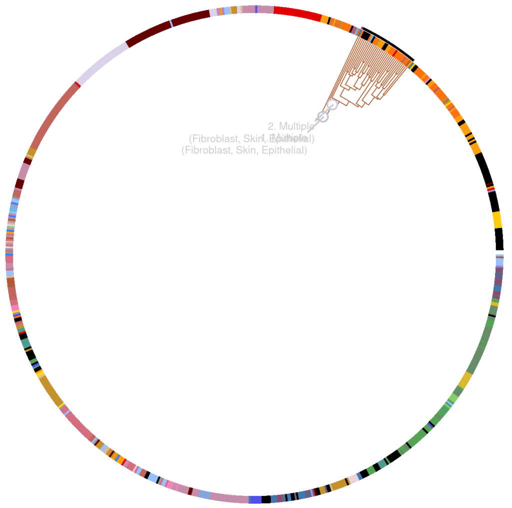

12,550 European and unknown ancestry chronic obstructive pulmonary disease cases, 46,368 European and unknown ancestry controls, 144,793 European ancestry high blood pressure cases, 313,761 European ancestry controls
