# Supplementary material for: Author Correction: Regulatory genomic circuitry of human disease loci by integrative epigenomics
Source: Nature. 2025 Jun 20;643(8071):E11. doi: 10.1038/s41586-025-09134-4 (PMC12240796; doi:10.1038/s41586-025-09134-4)
Supplement: Supplementary file 1 — Original, uncorrected article [file 41586_2025_9134_MOESM1_ESM.pdf]

Original, uncorrected article

Article

Regulatory genomic circuitry of human disease loci by integrative epigenomics

https://doi.org/10.1038/s41586-020-03145-z

Received: 30 October 2019

Accepted: 18 December 2020

Published online: 3 February 2021

Open access

Check for updates

Carles A. Boix[1,2,3], Benjamin T. James[1,2], Yongjin P. Park[1,2,4], Wouter Meuleman[5] & Manolis Kellis[1,2]✉

Annotating the molecular basis of human disease remains an unsolved challenge, as 93% of disease loci are non-coding and gene-regulatory annotations are highly incomplete[1–3]. Here we present EpiMap, a compendium comprising 10,000 epigenomic maps across 800 samples, which we used to define chromatin states, high-resolution enhancers, enhancer modules, upstream regulators and downstream target genes. We used this resource to annotate 30,000 genetic loci that were associated with 540 traits[4], predicting trait-relevant tissues, putative causal nucleotide variants in enriched tissue enhancers and candidate tissue-specific target genes for each. We partitioned multifactorial traits into tissue-specific contributing factors with distinct functional enrichments and disease comorbidity patterns, and revealed both single-factor monotropic and multifactor pleiotropic loci. Top-scoring loci frequently had multiple predicted driver variants, converging through multiple enhancers with a common target gene, multiple genes in common tissues, or multiple genes and multiple tissues, indicating extensive pleiotropy. Our results demonstrate the importance of dense, rich, high-resolution epigenomic annotations for the investigation of complex traits.

Genome-wide association studies (GWAS) have been successful in discovering more than 100,000 genomic loci that contain common single-nucleotide polymorphisms (SNPs) associated with complex traits and disease-related phenotypes, providing a very important starting point for the systematic investigation of the molecular mechanism of human disease[1,4]. However, the vast majority of these genetic associations remain devoid of any mechanistic hypothesis underlying their molecular and cellular functions, as more than 90% lie outside protein-coding exons and probably have non-coding roles in gene-regulatory regions with circuitry that remains unresolved[2,3].

Large-scale experimental mapping[5–8] and integration of histone modification marks and DNA accessibility have helped to annotate diverse classes of gene-regulatory annotations, including distal-acting and tissue-specific enhancers and proximal-acting and mostly constitutive promoters[9,10]. These maps help to elucidate the molecular basis of complex traits by revealing preferential localization (enrichment) of trait-associated genetic variants in tissue-specific gene-regulatory elements[3,6,11–16] and by fine-mapping possible causal genetic variants in enriched annotations[14,17–19]. However, these maps also have limitations: they miss many disease-relevant tissues, have variable quality, and are prone to experimental noise and methodological variation between protocols, laboratories, antibody lots, reagents, batches, computational processing pipelines, software versions and integration pipelines. Moreover, consortia that require common marks across samples often exclude samples that miss some marks or marks that are missing in some samples, thus reducing biological space coverage, and often only profile few marks in many samples, or many marks in few samples owing to cost limitations.

Here we overcome many of these limitations and present a new human epigenome reference, EpiMap (for epigenome integration across multiple annotation projects) (Fig. 1a). We inferred chromatin-state annotations that combine multiple marks[9], and a high-resolution enhancer annotation that combines DNA accessibility and multiple chromatin enhancer states. We grouped enhancers into modules that show common activity patterns, and inferred candidate upstream regulators and enriched functions of downstream genes for each module on the basis of regulatory motif and gene ontology enrichments. We also inferred enhancer target genes using a machine learning approach. We integrated this high-resolution gene-regulatory circuitry with genetic association results, revealing traits with epigenomic enrichments, and predicting causal variants and tissue-specific target genes. We distinguished unifactorial, multifactorial and polyfactorial traits on the basis of the diversity of their enriched tissues, and partitioned the loci of polyfactorial traits according to their overlap in distinct enriched tissues, thus revealing their distinct biological processes and disease comorbidity patterns. We also distinguished monotropic versus pleiotropic loci, and found that top-scoring loci frequently have multiple predicted driver variants, converging through diverse pleiotropy patterns involving multiple enhancers with a common target gene, multiple genes in a common tissue, or multiple genes in multiple tissues. Our results demonstrate the utility of dense, rich, multidimensional, high-resolution epigenomic and regulatory circuitry annotations for

[1]Computer Science and Artificial Intelligence Laboratory, Massachusetts Institute of Technology, Cambridge, MA, USA. [2]Broad Institute of MIT and Harvard, Cambridge, MA, USA. [3]Computational and Systems Biology Program, Massachusetts Institute of Technology, Cambridge, MA, USA. [4]Department of Pathology and Laboratory Medicine, University of British Columbia, Vancouver, British Columbia, Canada. [5]Altius Institute for Biomedical Sciences, Seattle, WA, USA. ✉e-mail: manoli@mit.edu

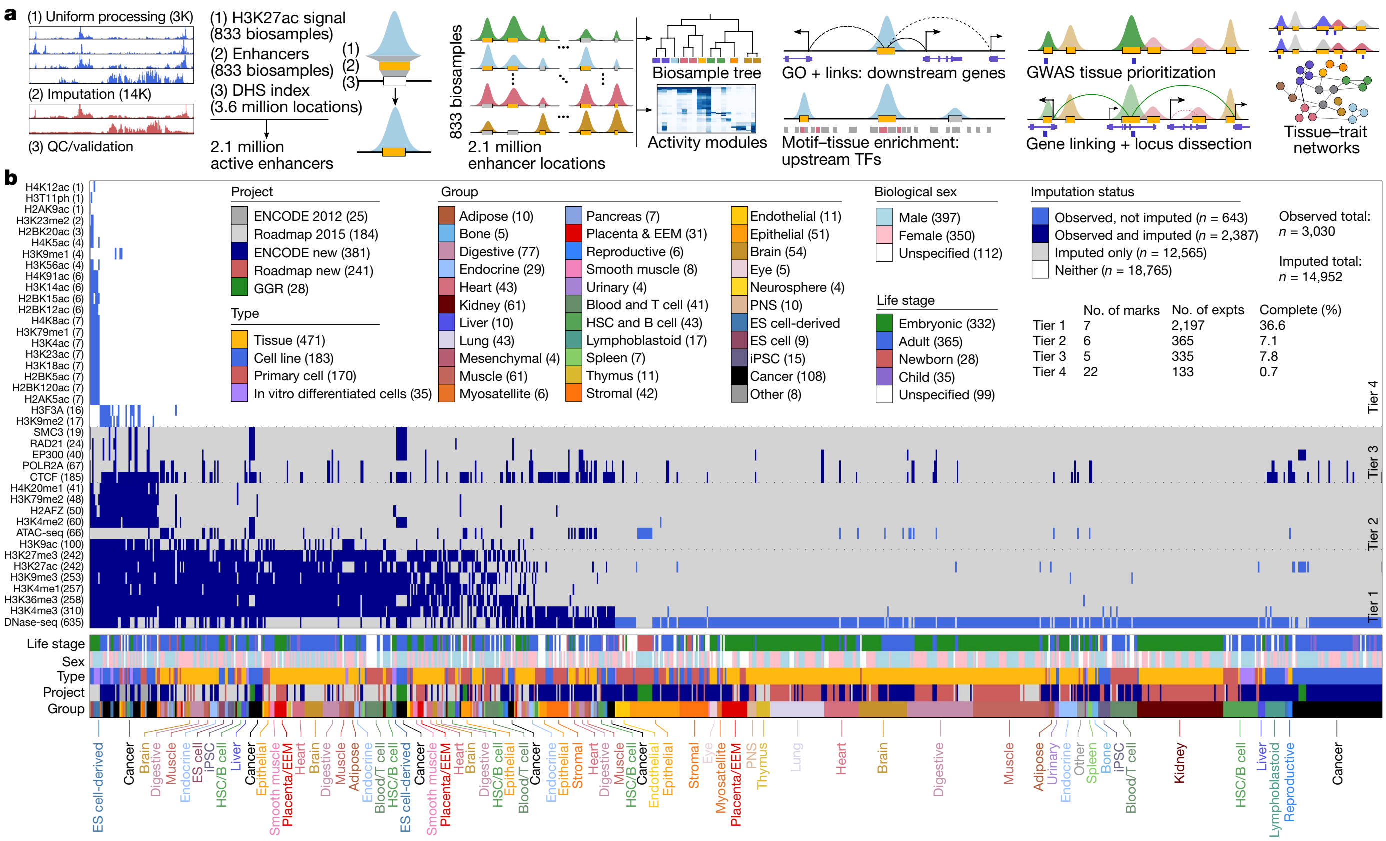

Fig. 1 | EpiMap resource overview. a, We created a compendium of over 17,000 epigenomic tracks across 18 marks by uniform processing and imputation and used these to call chromatin states for 833 biosamples and active-enhancer states over 2.1 million DNase I hypersensitive sites (DHSs). We used unsupervised clusters of the enhancer activities to call enhancer downstream target genes, upstream regulators, and to prioritize, investigate and compare hundreds of GWAS traits and thousands of loci. GO, gene ontology; QC, quality control; TF, transcription factor. **b**, Data matrix across 859 samples (columns) and 40 assays (rows), ordered by the number of experiments (parentheses) and coloured by metadata. EEM, extra-embryonic membranes; ES, embryonic stem; expts, experiments; HSC, haematopoietic stem cell; iPSC, induced pluripotent stem cell; H3T11ph, histone H3 phosphorylated at T11; PNS, peripheral nervous system. ENCODE new, ENCODE post-2012 data freeze + publication; Roadmap new, Roadmap post-2015 data freeze + publication.

gene regulatory studies, complex trait investigation and studies of disease locus mechanism, resulting in unprecedented scale, scope and coverage of biological space and disease complexity.

EpiMap generation and validation

We uniformly processed 3,030 observed[5–8] genomic tracks across 859 biosamples (406 ENCODE[5], 425 Roadmap Epigenomics[6] and 28 Genomics of Gene Regulation (GGR)[8] samples) that span 18 epigenomic assays, and computationally imputed[20] 14,952 tracks (Fig. 1b, Supplementary Fig. 1, Supplementary Table 1), which are available for download and interactive visualization[21] at http://compbio.mit.edu/epimap.

Our imputed tracks matched held-out observed tracks, both visually across randomly selected regions (Extended Data Fig. 1a, b) and quantitatively with more than 85% peak recovery and more than 75% average genome-wide correlation for punctate marks (59% of tracks) genome-wide (Supplementary Fig. 2). Imputation was robust even with few supporting datasets, and performed best when target datasets showed more than 50% average correlation to their ten nearest datasets, which held for 98% of single-assay samples (Supplementary Fig. 3).

Imputed data also matched independent post-data freeze experiments, outperforming 'average signal' and 'nearest track' benchmarks (in practice knowable only after generating the target track) for 96% of punctate marks and 77% of broad marks both genome-wide and specifically focusing on rare events (Extended Data Fig. 1c–h).

Disagreement between imputed and observed tracks helped to flag 138 potentially problematic datasets, which independently also showed markedly lower quality control scores (Supplementary Fig. 2a–c) and revealed potential sample or antibody swaps (Supplementary Figs. 4, 5), some of which were independently flagged by the data producers. Subtraction of the imputed track signal from the observed track signal revealed 13 experiments with potential antibody cross-reactivity or secondary specificity (Supplementary Figs. 6–8). From subsequent analyses, we removed the 138 flagged datasets and 442 tracks based solely on assay for transposase-accessible chromatin with high-throughput sequencing (ATAC-seq) or low-quality DNase-seq data, resulting in 2,850 observed and 14,510 imputed marks across 833 biosamples used in the remainder of this work.

Epigenomic landscape

The resulting compendium of 833 high-quality reference epigenomes, grouped into 33 tissue categories, represents a major increase in biological space coverage, with 75% (624 of 833) of biosamples corresponding to new biological specimens. Observed and imputed data co-clustered, but imputed datasets better captured the continuity between and within different sample categories, and more clearly revealed sample-type groups, probably driven by both their cleaner signal tracks and their sheer number. Moreover, the distances between imputed datasets were less affected by technical covariates (Supplementary Fig. 9) and were more consistent with sample groupings (Supplementary Fig. 10).

Hierarchical and two-dimensional embedding clusterings of multiple marks using both genome-wide and relevant-region-specific correlation patterns (Extended Data Fig. 2a–c) grouped biosamples first by

life stage (adult versus embryonic) and sample type (complex tissues versus primary cells versus cell lines), and second by distinct groups of brain, blood, immune, stem cell, epithelial, stromal and endothelial biosamples within them. Active marks (histone H3 lysine 27 acetylation (H3K27ac), histone H3 lysine 4 monomethylation (H3K4me1) and H3K9ac) primarily grouped biosamples by differentiation lineage (blood, immune, spleen, thymus, epithelial, stromal and endothelial) and tissue type (lung, kidney, heart, muscle and brain), while repressive marks (H3K27me3 and H3K9me3) captured life stage (pluripotent, induced pluripotent stem cell derived, embryonic and adult) (Extended Data Fig. 2b, Supplementary Fig. 11), consistent with previous studies[22,23]. Donor sex was not a primary factor in sample grouping.

We annotated genome-wide locations of 18 chromatin states[5,6,9] in all 833 biosamples using combinations of histone modifications, including multiple types of enhancer, promoter, transcribed, bivalent and repressed regions (Extended Data Fig. 3a), using a mixture of observed and scaled imputed data and excluding the 138 flagged observed datasets (Methods). Genomic coverage and mark frequencies remained stable across biosamples for most states (Extended Data Fig. 3b–d), but biosamples with fewer observed datasets showed more heterochromatin and Polycomb-repressed states, consistent with our previously noted lower imputation accuracy for broad marks.

We annotated 2.1 million high-resolution active-enhancer regions by intersecting five active-enhancer states with 3.6 million accessible DNA regions from 733 DNase-seq experiments[24]. These covered 13% of the genome cumulatively and 0.8% on average for biosamples individually (Fig. 2a, Supplementary Fig. 13a–g), and represent a more than twofold increase relative to the ENCODE 2020 release[25] (Extended Data Fig. 4). Clustering biosamples by sharing of active enhancers captured biologically meaningful groupings (Extended Data Fig. 5, Supplementary Fig. 12).

Enhancer modules, targets and regulators

For each high-resolution active-enhancer region, we defined H3K27ac-based local activity levels across 833 biosamples and used them to group enhancers into 300 enhancer modules (Fig. 2a, b, Extended Data Fig. 6a–c, Supplementary Figs. 14, 15), including 290 tissue-specific modules (1.8 million enhancers, 88% of enhancers cumulatively, active in 2% of biosamples on average) and 10 broadly active modules (251,079 enhancers, 12% of enhancers, active across 77% of sample categories on average).

Enhancer modules showed substantial high-resolution, tissue-specific gene ontology enrichments for neighbouring genes (Extended Data Figs. 6, 7, Supplementary Fig. 16), including ion channels (for brain modules); camera-type eye development (eye); neural precursor cell proliferation (neurosphere); endothelial proliferation, hemidesmosomes and digit morphogenesis (endothelial, stromal and epithelial); and organ development and morphogenesis (embryonic).

We predicted 3.3 million tissue-specific enhancer–gene links by combining epigenomic–transcriptional correlation and genomic proximity, each gene linked to 13 enhancers and each enhancer to 1.5 genes on average, at a median distance of 42,359 bp. Links were approximately sixfold more specific than enhancers, and sample-specific links spanned larger distances than constitutive links (Extended Data Fig. 8a–f). Our links outperformed previous linking approaches, using both gene-set enrichment metrics and curated gold-standard datasets (Methods, Extended Data Fig. 8g, h), and greatly expanded the biosamples with predicted links (from 127 to 833).

We predicted upstream regulators for 273 modules (91%), implicating 1,175 motifs grouped into 160 motif archetypes[26] (Extended Data Figs. 6, 7, Supplementary Fig. 17), including 152 tissue-specific motif archetypes (enriched in 6 modules on average) and 8 broadly enriched (enriched in 53 modules on average). Specific motifs include: *GATA* and *SPI1* in the blood and immune samples[27]; *NEUROD2* and *RFX4* in the brain and peripheral nervous system[28,29]; *KLF4* for digestive tissues[30]; and *TEAD3* for the placenta, myosatellite and epithelial cells[31].

Broadly enriched motifs revealed highly connected, combinatorially acting master regulators, including *HNF1A* in the liver, kidney and pancreas (with *NR5A2*)[32]; *AP-1* (also known as *JUN*) or *JDP2* in immune, bone and cancer samples[33]; and *TEAD3*, paired alternately with *MYF6* (myosatellite), *TFAP2A* (placenta) and *AP-1* (stromal) (Extended Data Fig. 7d, e).

Motif enrichments often partitioned tissue categories into subgroups specific for developmental stage and tissue type (Fig. 2c, Extended Data Fig. 7a–c), including heart into embryonic heart (*NFIX* and *E2F1*), aorta and arteries (*SRF* and *PAX5*), and heart chambers (*MEF2D* and *ESRRG*); brain into embryonic (*NFIX* and *NEUROD2*), adult brain (*RFX2* and *SOX10*), and astrocytes (*NFE2L2* and *JDP2*); and haematopoietic cells into natural killer cells (*ETV2*), B cells (*NFKB2* and *SPIB*), and multipotent progenitors (*GATA1* and *NFE2L2*).

Interpreting GWAS loci

We next used our 2.1 million enhancer annotations and their tissue specificity to interpret genetic variants associated with complex traits[3,6,11]. We compiled a compendium of 803 well-powered GWAS[34] with 10 or more significant loci and over 10,000 cases (15% of 5,454 GWAS publications) that capture over 70,000 GWAS loci (63% of the NHGRI-EBI catalogue[4]).

We found 17,658 significant trait–tissue enrichments, enabling fine-mapping of candidate driver SNPs in over 27,000 loci (39%) from 245 traits in tissue-enriched enhancers (false discovery rate of <1%) (Extended Data Fig. 9a, Supplementary Figs. 18, 19). New biosamples captured the strongest GWAS enrichment in 79% of cases (193 of 245) and the only significant enrichment in 24% of cases ($n = 58$), and our annotations captured 2.5-fold more GWAS studies than DNase alone (245 versus 97) (Extended Data Figs. 9b, 10a–d).

To capture common enrichments of similar biosamples, we also calculated trait enrichments for enhancer modules, resulting in approximately threefold more enriched traits (717 versus 245) but 38% fewer SNPs in enriched annotations (Supplementary Fig. 20). Instead, using the hierarchical enhancer-sharing tree (Extended Data Fig. 2c) to reveal the appropriate tissue resolution of GWAS enrichment in comparisons of subtree-versus-parent enhancers showed 2.2-fold more enriched traits (540 versus 245) and 20% more SNPs in enriched annotations (32,532 SNPs) (Extended Data Fig. 10e, Supplementary Fig. 18), representing an approximately tenfold increase from the 54 traits enriched in H3K27ac and the 58 traits enriched in H3K4me1 reported by the Roadmap Epigenomics project[6].

Our epigenomic enrichments and enhancer–gene links yielded new biological insights on disease loci, with many compelling examples. For breast cancer GWAS[35], enriched in epithelial and cancer biosamples (Extended Data Fig. 9c), the highly localized rs17356907 genetic signal ($P = 10^{-39}$, rank no. 16) localized precisely in a narrow epithelial and cancer enhancer nearest to *USP44* but linked instead to *NTN4*, which is implicated in tumorigenesis and angiogenesis (Extended Data Fig. 9d). For schizophrenia GWAS[36], maximally enriched in the mid-frontal cortex (Extended Data Fig. 9e), the diffuse rs2007044 genetic signal ($P = 10^{-17}$, rank no. 3) overlapped a broad set of enhancers nearest to the *DCP1B* promoter, all of which linked to *CACNA1C*, which encodes a calcium channel implicated in neuropsychiatric disorders, suggesting that multiple causal variants may contribute jointly to its dysregulation[37] (Extended Data Fig. 9f). We have provided an interactive website for exploring more than 30,000 additional loci across more than 500 traits at http://compbio.mit.edu/epimap.

GWAS and tissue co-enrichments

We then studied trait–tissue, trait–trait and tissue–tissue epigenome GWAS co-enrichment patterns to gain insights into their complex

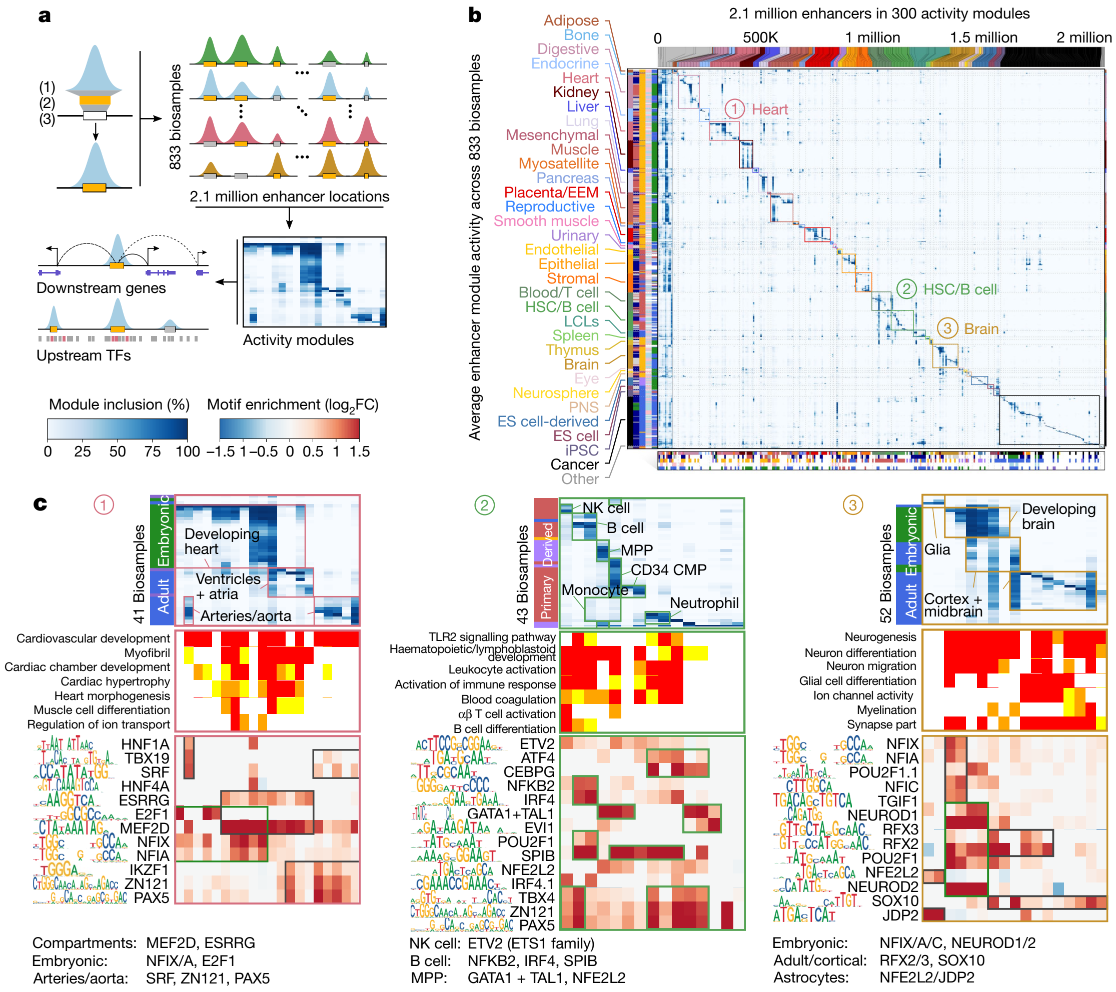

Fig. 2 | Enhancer module circuitry. a, Overview of gene-regulatory module clustering. The full module breakdown is shown in Extended Data Figs. 6, 7 and online at http://compbio.mit.edu/epimap. Activity modules are shown in Fig. 2b, Extended Data Fig. 6a. FC, fold change. **b**, Clustering of 2.1 million enhancer elements (top) into 300 modules (columns) using the activity levels of enhancers (heat map) across 833 samples (rows), quantified by the levels of H3K27ac within accessible enhancer chromatin states. Bottom, the enrichment of each module for each metadata annotation, highlighting 34 groups of modules (separated by dotted lines): 33 specific to sample type (coloured boxes) and 1 multiply enriched (left-most). LCL, lymphoblastoid cell line. **c**, Subsets of enhancer module centres (top panels) and motifs (bottom panels) for heart, brain and haematopoietic cell samples (top, rows), selected GO terms (middle, rows) and selected motifs (bottom, rows) in modules (columns) with maximal enrichment in each of the three sample categories. The GO heat map is coloured by enrichment $-\log_{10}P$ (0–2: white; 2–3: yellow; 3–4: orange; and 4+: red). The full subsets are shown in Extended Data Fig. 7. CMP, common myeloid progenitor; MPP, multipotent progenitor; NK, natural killer.

interactions. First, we used the number of distinct tissue categories enriched in each trait (Extended Data Fig. 9a (left), Supplementary Data 1) to distinguish: 56 'unifactorial' traits (22%) with most enriched nodes in only one tissue group (for example, QT interval in the heart, educational attainment in the brain and hypothyroidism in immune cells) versus 192 'multifactorial' traits (79%) enriched in five tissue categories on average (for example, Alzheimer disease in immune cells and the brain[38]; waist-to-hip ratio[39] in adipose, muscle, kidney and digestive tissues), of which 26 'polyfactorial' traits (11%) enriched in 14 tissue categories on average (including coronary artery disease (CAD)[40] in 19 tissue groups, including liver, heart, adipose, muscle and endocrine samples).

Second, we used trait co-enrichment patterns in the same tissues to cluster GWAS traits with similar properties. The resulting network (Fig. 3) showed a small number of densely connected communities of primarily unifactorial traits (for example, cognitive and psychiatric traits in the brain and neurons, heartbeat intervals in the heart, cholesterol in the liver, filtration in the kidney, immune traits in T cells and blood cell counts in haematopoietic cells) with multifactorial connectors between them (for example, CAD between heart, endocrine and liver; HDL and triglycerides between liver and adipose; lung function between lung, heart and digestive tissue; blood pressure between heart and endocrine, endothelial and liver; and cell count between liver and digestive tissue) (Supplementary Figs. 21, 22). Many biologically meaningful similarities in this epigenomic co-enrichment-based network are missed by a network based on genetic overlap (934 edges, traits sharing 5% or more loci at a 10-kb resolution), which only captures 5% of epigenomic co-enrichment edges (283 of 5,547) (Supplementary Figs. 23–25).

Third, we used co-enrichment properties of pairs of tissues in the same traits to distinguish 'principal' tissues (for example, immune cells, liver, heart, brain and adipose tissues) that showed consistently higher enrichments versus 'partner' tissues (for example, digestive, lung, muscle and epithelial tissues) for the same GWAS traits, suggesting that they have driver rather than auxiliary roles (Extended Data Fig. 11a). Specific principal–partner tissue pairs co-occurred more frequently than expected (Extended Data Fig. 11b), and revealed biologically meaningful traits where they probably co-act (Extended Data Fig. 11c), including: liver

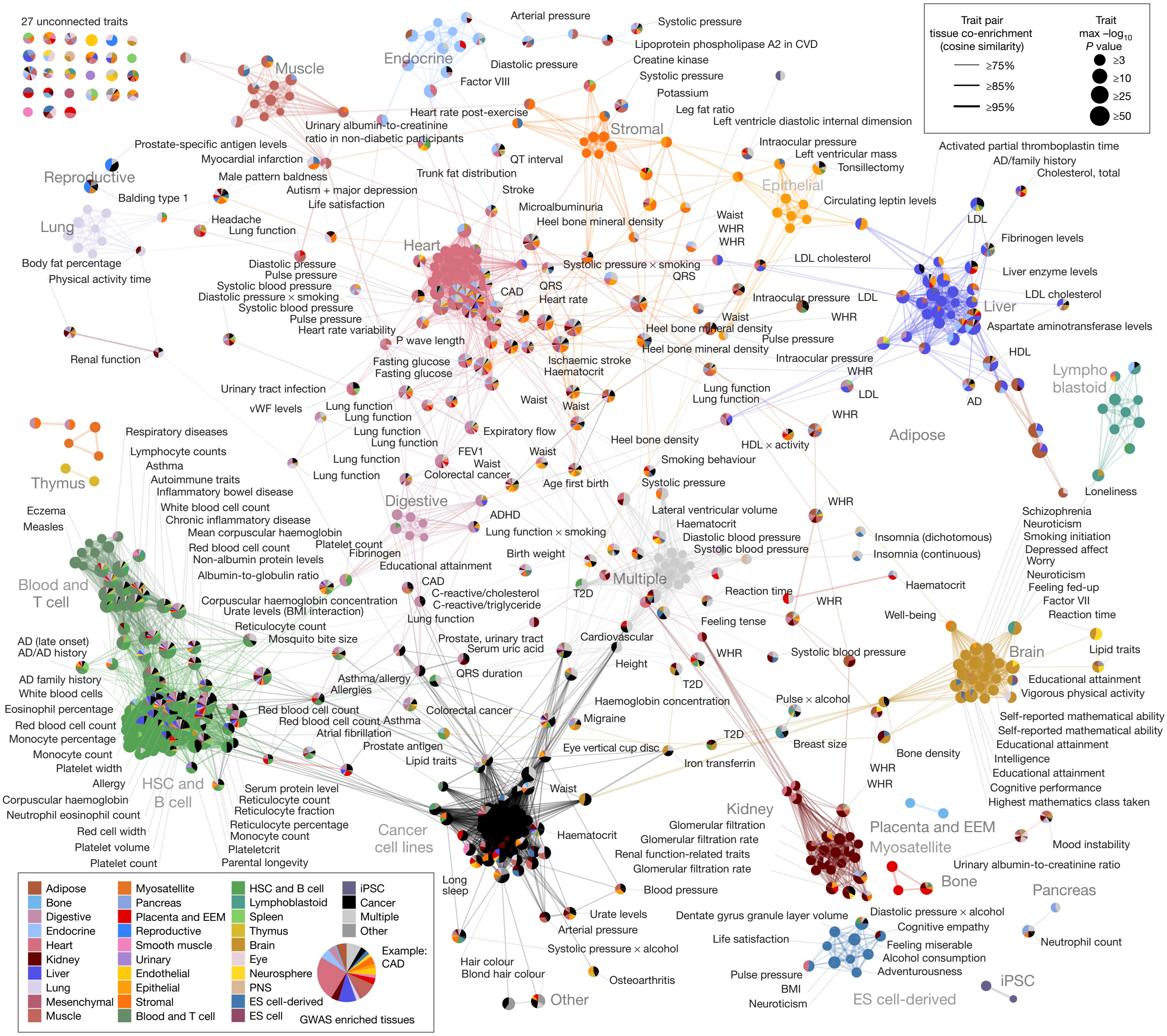

Fig. 3 | Trait–trait network. The network across 538 traits (by per-node false discovery rate correction) by similarity of epigenetic enrichments (cosine similarity ≥ 0.75), laid out using the Fruchterman–Reingold algorithm. Traits (nodes) are coloured by the contributing groups (pie chart by the fraction of $-\log_{10}P$, and size by maximal $-\log_{10}P$) and interactions (edges) by the group with the maximal dot product of enrichments between two traits. The redundant node names indicate different GWAS (the full names for non-singleton nodes are available in Supplementary Fig. 22). AD, Alzheimer disease; ADHD, attention-deficit/hyperactivity disorder; BMI, body mass index; CVD, cardiovascular disease; FEV1, forced expiratory volume in 1 s; T2D, type 2 diabetes; vWF, von Willebrand factor; WHR, waist-to-hip ratio.

with adipose tissue (for cholesterol traits), with digestive tissue (for gallstone) and with blood cells (for serum protein levels); and adipose tissue with endothelial cells (for waist-to-hip ratio), with heart tissue (for atrial fibrillation) and with muscle tissue (for blood pressure).

Partitioning multifactorial traits

We next used our epigenomic annotations to partition multifactorial trait SNPs into tissue-specific components, by studying functional and disease enrichments for distinct subsets of enhancer-overlapping SNPs in each enriched tissue (Fig. 4a–d, Extended Data Fig. 12, Supplementary Fig. 26).

For example, the 339 CAD-associated SNPs lying in enriched tissue enhancers partitioned into: 195 heart-enhancer SNPs enriched in artery, cardiac and vessel morphogenesis; 171 endocrine-enhancer SNPs in lipid homeostasis; 169 liver-enhancer SNPs in cholesterol and lipid metabolism and transport; 122 adipose-enhancer SNPs in axon guidance and focal adhesion, consistent with adipose tissue innervation processes; and 112 embryonic stem cell-derived–muscle enhancer SNPs, enriched in septum morphogenesis, chamber and aorta development.

These partitions also showed distinct co-associations (Fig. 4e). For example: heart, muscle and endothelial enhancer CAD SNPs co-associated with high blood pressure and atrial fibrillation; liver and endocrine enhancer CAD SNPs with systolic blood pressure; adipose enhancer CAD SNPs with waist-to-hip ratio; and liver, adipose and endocrine CAD SNPs with HDL cholesterol.

Individual multifactorial trait loci included both single-tissue and multiple-tissue loci (Fig. 4f). Some CAD loci overlapped only heart

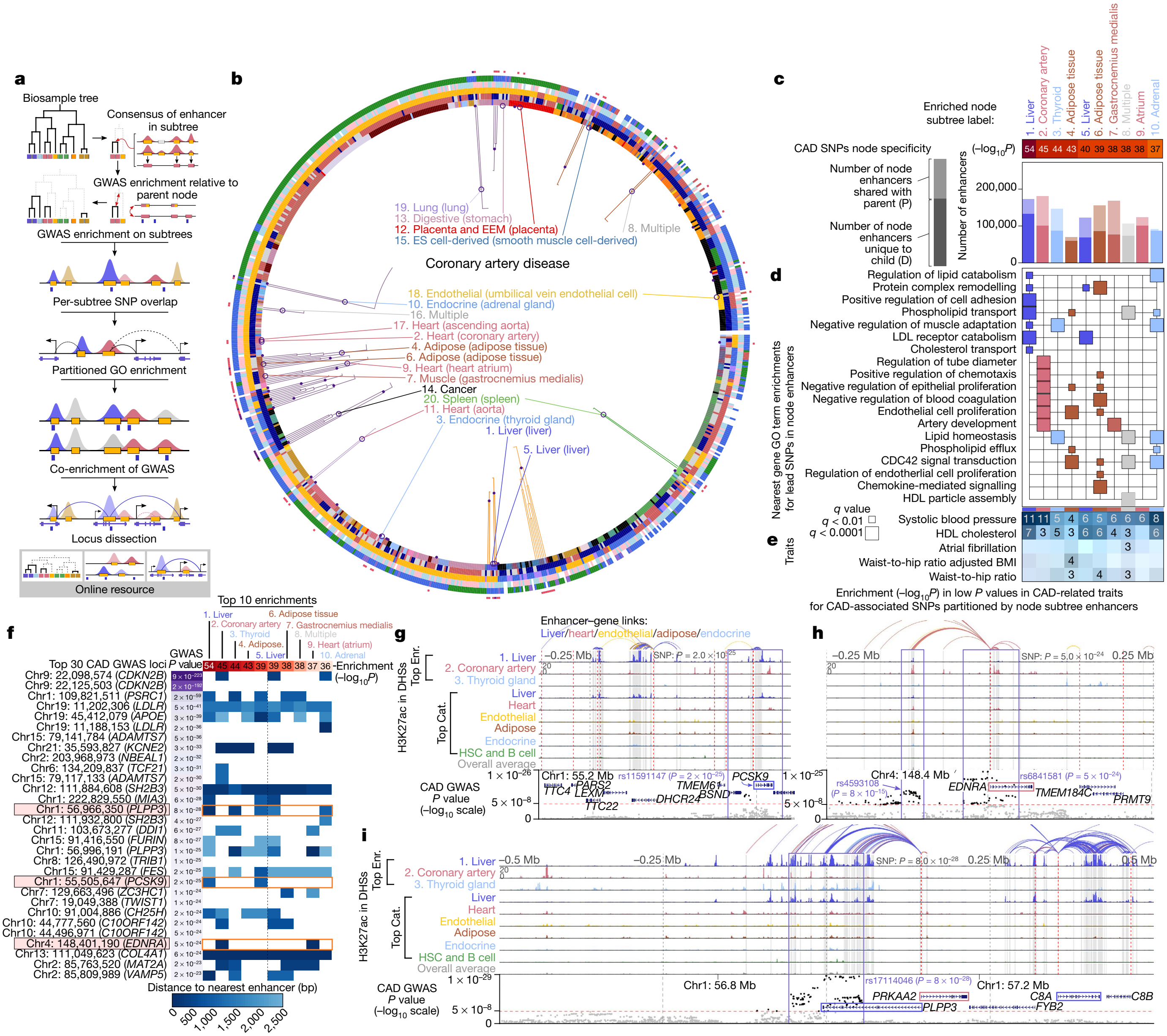

Fig. 4 | Partitioning of polyfactorial traits. a, Workflow for the investigation of GWAS epigenomic enrichment using the biosample tree (Extended Data Fig. 2c). Additional trait enrichments, SNP assignments, links and their corresponding loci are available at http://compbio.mit.edu/epimap. **b**, Epigenomic enrichments for CAD[40] on the enhancer-sharing tree. Nodes that passed false discovery rate < 0.1% are labelled by rank, category and components, and subtrees are shown (the large circles are the top 20 nodes by $-\log_{10}P$). The leaves are annotated by metadata and the number of enriched parent nodes (outer, red = 1, black = 2). **c**, The top 10 enriched nodes for CAD with nominal *P* values (heat map) and shared enhancer set sizes (bar plot) with the number at the subtree (full bar) and the number of differential enhancers between the node and its parent (tested set, dark bar). **d**, GO enrichments of node enhancers with lead SNPs (nearest expressed genes), coloured by the tissue group of each node and diagonalized (over-representation test). **e**, Enrichment for significant loci in overlap of CAD loci with loci from five related traits, within enriched enhancers in each node (heat map, $-\log_{10}P$ of one-tailed Mann–Whitney test against the loci of each trait in the enhancer annotations). **f**, Enhancer overlaps with the top 30 lead SNPs from CAD GWAS for the top 10 enrichments on the enhancer tree. **g**–**i**, Loci centred on CAD lead SNPs with links (top), the H3K27ac signal (middle) and GWAS summary statistics, for lead SNPs rs11591147 (chr1: 55,505,647; $P = 2\times10^{-25}$) (**g**), rs6841581 (chr4: 148,401,190; $P = 5\times10^{-24}$) (**h**) and rs17114046 (chr1: 56,966,350; $P = 8\times10^{-28}$) (**i**). Loci show enhancer–gene links for SNP proximal enhancers for the top enrichments (Enr.) and across the locus for labelled categories (Cat.; linked enhancers in grey) (top); the H3K27ac signal in enhancers for the top three enriched subtrees, the six selected tissue categories and the average (middle); and genes (transcription start site (red lines)) and CAD GWAS summary statistics, with SNPs below $P = 5\times10^{-8}$ in grey (bottom).

enhancers (for example, *EDNRA*, *TCF21* and *ADAMTS7*), some only liver enhancers (for example, *PCSK9*), some lacked any enhancer overlaps (possibly acting at non-enhancer levels of regulation, or in uncaptured tissues or conditions), and many overlapped enhancers that were active in multiple tissues (for example, *LDLR*, *APOE*, *SH2B3* and *COL4A1*), suggesting multiple mechanisms of action even at the single-locus level.

For example, the liver-only CAD-associated locus near the LDL cholesterol regulator *PCSK9* (ref. [41]) (rs11591147, $P = 2\times10^{-25}$, rank no. 21) showed a strong liver-specific signal and liver-specific enhancer–gene links to *PCSK9* (Fig. 4g). The heart-only CAD-associated 250-kb locus near *EDNRA* contains two separate associations, the transcription start site-centred rs6841581 ($P = 5\times10^{-24}$, rank 27) and the enhancer-centred

rs4583018 ($P = 8 \times 10^{-15}$, rank 66), both in strong coronary artery enhancers and both linked to *EDNRA* through strong artery links, putatively reflecting multiple functional variants[37] that converge on the same target gene in the same tissue (Fig. 4h).

Even seemingly single-tissue loci sometimes showed second-tissue signals: the 1-Mb rs17114046 locus ($P = 8 \times 10^{-28}$, rank no. 14; Fig. 4i) showed primarily liver activity with multiple SNP-overlapping enhancers linked to liver-expressed *PLPP3*, the liver-specific deletion of which increases atherosclerosis[42]; however, our liver-specific links also implicated liver-produced complement factor *C8A*[43], and our heart-specific and muscle-specific links implicated *PRKAA2*, which encodes an AMP kinase subunit that is involved in cardiac metabolism[44]. These are both biologically relevant, highlighting that even individual loci may be pleiotropic, a property repeatedly found for many top-scoring loci.

Discussion

In this work, we presented a comprehensive map of the human epigenome, EpiMap, encompassing approximately 15,000 epigenomic tracks across 833 distinct biological samples that greatly expand the coverage of both embryonic and adult tissues and cells. We combined observed and imputed datasets across 18 epigenomic marks to jointly annotate and distinguish diverse classes of chromatin states, including enhancer, promoter, transcribed, repressed and quiescent regions. We extensively validated the high quality of our annotations and found that they outperformed stringent benchmarks, using both held-out and external experimental datasets for validation.

We used this resource to assemble a comprehensive view of human genome circuitry across primary tissues, cells and cell lines, annotating 2.1 million high-resolution gene-regulatory regions; their activity patterns across 833 biosamples; their enriched regulatory motifs, motif combinations and putative upstream regulators that are responsible for their co-regulation; their enriched gene functions and biological pathways that they probably control; and their tissue-specific target genes. Our high-resolution enhancer annotations provide a highly concentrated view of the non-coding landscape, yielding many gene-regulatory insights but covering only 0.8% of the genome in each sample and only 13% total across all samples. Our linking revealed the high number of enhancers that control each gene and the high tissue specificity of long-range enhancer–gene links. Our upstream regulator analysis revealed a highly combinatorial and hierarchical view of gene regulation, with a small number of master regulators (for example, *RFX2–RFX4*, *GRHL1*, *HNF1A* and *AP-1*) interacting with diverse partners in different tissues to define tissue-specific gene-regulatory programs.

Our work has also provided high-resolution molecular investigations of complex traits and human disease circuitry. We found statistically significant epigenomic enrichments for 540 GWAS traits implicating 30,000 SNPs in tissue-enriched enhancers, used trait and tissue co-enrichment patterns to annotate tissue partnerships and trait pleiotropy, and to partition disease SNPs into tissue-specific functional components. For individual GWAS loci, our work provides mechanistic insights at unprecedented scale. We have highlighted specific examples of GWAS investigations at varying levels of complexity, from the typically sought single-enhancer to single-gene, to multiple enhancers converging on a single target[37], to multiple genes and multiple tissues acting in pleiotropy in a single locus.

Beyond the specific examples highlighted in our figures, we have also provided a rich interactive supplementary website (Supplementary Fig. 27) for our study (at http://compbio.mit.edu/epimap), enabling detailed interactive exploration of functional and motif enrichments of 300 enhancer modules; motif–tissue networks and enrichments; GWAS enrichments for 540 traits against our biosample tree; GWAS-enriched tissue enhancer SNP overlaps and target gene predictions; and 30,000 disease locus visualizations with putative driver SNPs, enhancers, tissues and tissue-specific target genes. These can enable the generation of detailed hypotheses for future experimental follow-up in countless studies of gene regulation and disease.

Our collection also has several limitations: tissue samples are not at single-cell resolution; we do not consider donor genotype or phenotype; imputation may result in increased homogeneity and miss rare sample-specific events; and we still miss many tissues, environmental and stimulation conditions, and developmental stages.

Our work enables many future studies: hierarchical and multi-resolution tree-based analyses of gene regulation and GWAS; machine learning-based gene circuitry and combinatorial regulatory motif analyses[45,46]; more sophisticated network analyses of our tissue–trait, trait–trait and tissue–tissue relationships; and guiding the experimental prioritization, methodological development and validation experiments, which can continue to further our understanding of gene regulation and human disease circuitry.

Online content

Any methods, additional references, Nature Research reporting summaries, source data, extended data, supplementary information, acknowledgements, peer review information; details of author contributions and competing interests; and statements of data and code availability are available at https://doi.org/10.1038/s41586-020-03145-z.

1. Visscher, P. M. et al. 10 Years of GWAS discovery: biology, function, and translation. *Am. J. Hum. Genet*. **101**, 5–22 (2017).
2. Gallagher, M. D. & Chen-Plotkin, A. S. The post-GWAS era: from association to function. *Am. J. Hum. Genet*. **102**, 717–730 (2018).
3. Ward, L. D. & Kellis, M. Interpreting noncoding genetic variation in complex traits and human disease. *Nat. Biotechnol*. **30**, 1095–1106 (2012).
4. Buniello, A. et al. The NHGRI-EBI GWAS catalog of published genome-wide association studies, targeted arrays and summary statistics 2019. *Nucleic Acids Res*. **47**, D1005–D1012 (2019).
5. ENCODE Project Consortium. An integrated encyclopedia of DNA elements in the human genome. *Nature* **489**, 57–74 (2012).
6. Roadmap Epigenomics Consortium et al. Integrative analysis of 111 reference human epigenomes. *Nature* **518**, 317–330 (2015).
7. Stunnenberg, H. G. & Hirst, M. The International Human Epigenome Consortium. a blueprint for scientific collaboration and discovery. *Cell* **167**, 1145–1149 (2016).
8. Genomics of Gene Regulation. *Genome.gov* https://www.genome.gov/Funded-Programs-Projects/Genomics-of-Gene-Regulation (accessed 28 September 2020).
9. Ernst, J. & Kellis, M. Discovery and characterization of chromatin states for systematic annotation of the human genome. *Nat. Biotechnol*. **28**, 817–825 (2010).
10. Hoffman, M. M. et al. Unsupervised pattern discovery in human chromatin structure through genomic segmentation. *Nat. Methods* **9**, 473–476 (2012).
11. Ernst, J. et al. Mapping and analysis of chromatin state dynamics in nine human cell types. *Nature* **473**, 43–49 (2011).
12. Maurano, M. T. et al. Systematic localization of common disease-associated variation in regulatory DNA. *Science* **337**, 1190–1195 (2012).
13. Cantor, R. M., Lange, K. & Sinsheimer, J. S. Prioritizing GWAS results: a review of statistical methods and recommendations for their application. *Am. J. Hum. Genet*. **86**, 6–22 (2010).
14. Farh, K. K.-H. et al. Genetic and epigenetic fine mapping of causal autoimmune disease variants. *Nature* **518**, 337–343 (2015).
15. Dimas, A. S., Deutsch, S. & Stranger, B. E. Common regulatory variation impacts gene expression in a cell type-dependent manner. *Science* **325**, 1246–1250 (2009).
16. Ward, L. D. & Kellis, M. HaploReg: a resource for exploring chromatin states, conservation, and regulatory motif alterations within sets of genetically linked variants. *Nucleic Acids Res*. **40**, D930–D934 (2012).
17. Pickrell, J. K. Joint analysis of functional genomic data and genome-wide association studies of 18 human traits. *Am. J. Hum. Genet*. **94**, 559–573 (2014).
18. Gusev, A. et al. Partitioning heritability of regulatory and cell-type-specific variants across 11 common diseases. *Am. J. Hum. Genet*. **95**, 535–552 (2014).
19. Finucane, H. K. et al. Partitioning heritability by functional annotation using genome-wide association summary statistics. *Nat. Genet*. **47**, 1228–1235 (2015).
20. Ernst, J. & Kellis, M. Large-scale imputation of epigenomic datasets for systematic annotation of diverse human tissues. *Nat. Biotechnol*. **33**, 364–376 (2015).
21. Li, D., Hsu, S., Purushotham, D., Sears, R. L. & Wang, T. Epigenome browser update 2019. *Nucleic Acids Res*. **47**, W158–W165 (2019).
22. Calo, E. & Wysocka, J. Modification of enhancer chromatin: what, how, and why? *Mol. Cell* **49**, 825–837 (2013).
23. Becker, J. S., Nicetto, D. & Zaret, K. S. H3K9me3-dependent heterochromatin: barrier to cell fate changes. *Trends Genet*. **32**, 29–41 (2016).
24. Meuleman, W. et al. Index and biological spectrum of human DNase I hypersensitive sites. *Nature* **584**, 244–251 (2020).
25. ENCODE Project Consortium et al. Expanded encyclopaedias of DNA elements in the human and mouse genomes. *Nature* **583**, 699–710 (2020).

26. Vierstra, J. et al. Global reference mapping of human transcription factor footprints. *Nature* **583**, 729–736 (2020).
27. Laslo, P. et al. Multilineage transcriptional priming and determination of alternate hematopoietic cell fates. *Cell* **126**, 755–766 (2006).
28. Blackshear, P. J. et al. Graded phenotypic response to partial and complete deficiency of a brain-specific transcript variant of the winged helix transcription factor RFX4. *Development* **130**, 4539–4552 (2003).
29. Olson, J. M. et al. NeuroD2 is necessary for development and survival of central nervous system neurons. *Dev. Biol*. **234**, 174–187 (2001).
30. Katz, J. P. et al. The zinc-finger transcription factor Klf4 is required for terminal differentiation of goblet cells in the colon. *Development* **129**, 2619–2628 (2002).
31. Jacquemin, P., Martial, J. A. & Davidson, I. Human TEF-5 is preferentially expressed in placenta and binds to multiple functional elements of the human chorionic somatomammotropin-B gene enhancer. *J. Biol. Chem*. **272**, 12928–12937 (1997).
32. Tanaka, T. et al. Dysregulated expression of P1 and P2 promoter-driven hepatocyte nuclear factor-4α in the pathogenesis of human cancer. *J. Pathol*. **208**, 662–672 (2006).
33. Wagner, E. F. & Eferl, R. Fos/AP-1 proteins in bone and the immune system. *Immunol. Rev*. **208**, 126–140 (2005).
34. MacArthur, J. et al. The new NHGRI-EBI catalog of published genome-wide association studies (GWAS catalog). *Nucleic Acids Res*. **45**, D896–D901 (2017).
35. Michailidou, K. et al. Association analysis identifies 65 new breast cancer risk loci. *Nature* **551**, 92–94 (2017).
36. Goes, F. S. et al. Genome-wide association study of schizophrenia in Ashkenazi Jews. *Am. J. Med. Genet. B. Neuropsychiatr. Genet*. **168**, 649–659 (2015).
37. Lupien, M., Markowitz, S. & Scacheri, P. C. Combinatorial effects of multiple enhancer variants in linkage disequilibrium dictate levels of gene expression to confer susceptibility to common traits. *Genome Res*. **24**, 1–13 (2014).
38. Henstridge, C. M., Hyman, B. T. & Spires-Jones, T. L. Beyond the neuron–cellular interactions early in Alzheimer disease pathogenesis. *Nat. Rev. Neurosci*. **20**, 94–108 (2019).
39. Winkler, T. W. et al. The influence of age and sex on genetic associations with adult body size and shape: a large-scale genome-wide interaction study. *PLoS Genet*. **11**, e1005378 (2015).
40. van der Harst, P. & Verweij, N. Identification of 64 novel genetic loci provides an expanded view on the genetic architecture of coronary artery disease. *Circ. Res*. **122**, 433–443 (2018).
41. Abifadel, M. et al. Mutations in *PCSK9* cause autosomal dominant hypercholesterolemia. *Nat. Genet*. **34**, 154–156 (2003).
42. Busnelli, M. et al. Liver-specific deletion of the *Plpp3* gene alters plasma lipid composition and worsens atherosclerosis in $apoE^{-/-}$ mice. *Sci. Rep*. **7**, 44503 (2017).
43. Lappegård, K. T. et al. A vital role for complement in heart disease. *Mol. Immunol*. **61**, 126–134 (2014).
44. Arad, M., Seidman, C. E. & Seidman, J. G. AMP-activated protein kinase in the heart: role during health and disease. *Circ. Res*. **100**, 474–488 (2007).
45. Lee, D. et al. A method to predict the impact of regulatory variants from DNA sequence. *Nat. Genet*. **47**, 955–961 (2015).
46. Moyerbrailean, G. A. et al. Which genetics variants in DNase-seq footprints are more likely to alter binding? *PLoS Genet*. **12**, e1005875 (2016).

Publisher's note Springer Nature remains neutral with regard to jurisdictional claims in published maps and institutional affiliations.

Open Access This article is licensed under a Creative Commons Attribution 4.0 International License, which permits use, sharing, adaptation, distribution and reproduction in any medium or format, as long as you give appropriate credit to the original author(s) and the source, provide a link to the Creative Commons license, and indicate if changes were made. The images or other third party material in this article are included in the article's Creative Commons license, unless indicated otherwise in a credit line to the material. If material is not included in the article's Creative Commons license and your intended use is not permitted by statutory regulation or exceeds the permitted use, you will need to obtain permission directly from the copyright holder. To view a copy of this license, visit http://creativecommons.org/licenses/by/4.0/.

© The Author(s) 2021

Methods

Epigenomic datasets and processing

Primary data sources and metadata information. We analysed 3,030 datasets, including 2,329 epigenomic chromatin immunoprecipitation followed by sequencing (ChIP–seq) datasets, 635 DNase-seq datasets and 66 ATAC-seq datasets from ENCODE at https://www.encodeproject.org/, released as of 24 September 2018. These marks include tier 1 assays: DNase-seq, H3K4me1, H3K4me3, H3K27ac, H3K36me3, H3K9me3 and H3K27me3; tier 2 assays: ATAC-seq, H3K9ac, H3K4me2, H2AFZ, H3K79me2 and H4K20me1; tier 3 assays: POLR2A, p300, CTCF, SMC3 and RAD21; and tier 4 histone marks: 16 non-imputed histone acetylation marks, 4 methylation marks (H3K9me2, H3K79me1, H3K9me1 and H3K23me2), H3.3 and H3T11ph. We assigned unique sample IDs to each unique combination of: extended biosample summary, donor, sex, age and life stage, wherever each attribute was available. We removed samples with genetic perturbations and kept only samples with appropriately matched ChIP–seq controls. We provide a metadata matrix including the mapping between ENCODE accessions and our unique sample IDs (Supplementary Table 1; also at http://compbio.mit.edu/epimap). We mapped the 111 Roadmap biosamples and the 16 ENCODE 2012 biosamples to any of our biosamples with overlapping dataset accessions if the accessions were used in the flagship Roadmap epigenomics analysis. This mapping assigned 25 samples to ENCODE 2012 and 184 samples to Roadmap 2015, some of which were merged multi-donor samples in Roadmap, out of the final 833 samples that passed quality control. These were merged into 16 and 111 tissue types, respectively, in the Roadmap 2015 publication[6].

Uniform data processing. We downloaded one alignment file per replicate, prioritizing filtered alignments aligned with BWA in hg19 whenever possible. We uniformly processed the ChIP–seq and DNase-seq datasets according to the processing pipelines established by the Roadmap Epigenomics Consortium[6]. In brief, we filtered out improperly paired and non-uniquely mapped reads, truncated reads to 36 bp, filtered out a blacklist of low complexity and artefact regions (ENCODE accession ENCSR636HFF), and filtered reads against a mappability track of uniquely mappable regions for 36-bp reads[47]. Truncating read lengths inevitably missed some repetitive regions that the longer reads could have helped resolve, but helped to avoid potential biases from alignment differences, as over two-thirds of the datasets had read lengths of 36 bp or lower (Supplementary Fig. 9). We converted .bam files to tagAlign, used liftOver[48] to map GRCh38 alignments to hg19, and pooled all experiments within each ID and assay combination. We subsampled the pooled ChIP–seq datasets to a maximum of 30 million reads and the DNase-seq and ATAC-seq datasets to a maximum of 50 million reads. We used the SPP peak caller[49] to estimate fragment length. In cases with extremely low fragment length in the ATAC-seq and DNase-seq datasets we used the average fragment length (73 bp) from the average of the rest of the tracks. We generated $-\log_{10}$ *P* value signal tracks against matched whole cell extracts for both the ChIP–seq and the accessibility datasets using the MACS2[50] and the SPP[49] peak caller and cross-correlation analysis to identify the proper fragment length as in the Roadmap analysis.

Epigenomic imputation

Imputation. We carried out epigenomic imputation on 859 unique biosamples using ChromImpute[20] for a total of 10,778 imputed datasets over 13 tier 1 and tier 2 assays using predictors trained on all 35 epigenomic assays across 859 samples. We also imputed 4,345 datasets for the five DNA-associated factors, using only the 35 epigenomic assays as features to train predictors with ChromImpute. We provide all imputed and processed observed tracks along with track sets for the 833 quality controlled samples at https://epigenome.wustl.edu/epimap[21].

Quality control. For imputation quality control and validation, we compared observed tracks to imputed tracks when both were available (that is, when at least two original observed datasets were available for that biosample). We calculated all imputation quality control metrics from the original ChromImpute publication[20], including genome-wide correlation, imputed and observed peak recovery (%), and the area under the receiver-operator characteristic curve (AUC) for all pairs of imputed and observed tracks. In addition to the quantitative metrics, we visually inspected the epigenomic predictions as part of our quality control. We showed (Extended Data Fig. 1b) three dense and varied regions of different resolutions (25 kb, 200 kb and 1.5 Mb) for each of two randomly chosen samples containing both observed and imputed tracks for each assay. We calculated the epigenomic profile quality metrics normalized strand cross-correlation coefficient (NSC), relative strand cross-correlation coefficient (RSC) and read depth for all datasets and compared these to the imputation quality control metrics (see the tables in Supplementary Table 1). We flagged low-quality tracks by detecting the elbow in the ranked correlation metrics, which we calculated as the point where the change in correlation exceeded 5% of the correlation. Validation on external datasets was carried out on 51 experimental tracks across eight marks and assays from ENCODE after our data freeze, similarly subsampled to 30 million (marks) and 50 million reads (accessibility), which we remapped from GRCh38 to hg19 and evaluated on fully remapped 200-bp bins (90.1%) in chromosome 1 (Extended Data Fig. 1c, d). For the data homogeneity analysis, we restricted the data to only biosamples in each mark with both observed and imputed data (Supplementary Fig. 10).

Sample and antibody swap detection. To systematically identify both potential sample or antibody swaps and poor-quality experiments, we computed the correlation of each observed experiment against all 10,734 imputed tracks for histone marks and assays (all imputed tracks before removing samples by quality control). We then calculated the average correlation among the top 10 most similar tracks to each observed track. We flagged potential antibody swaps by comparing the average correlation against samples of the putative mark against those computed for other marks. We fitted a multivariate linear model to each mark comparison, flagged datasets with residuals greater than 3 standard deviations of the average correlation and visually confirmed seven antibody swaps (six low-quality tracks). Similarly, we flagged potential sample swaps by comparing the correlation between imputed and observed tracks against the average correlation in the top 10 tracks in the same mark. We fitted a multivariate linear model and flagged datasets with residuals greater than 3 standard deviations of the residuals distribution. We report 19 potentially swapped samples, of which 5 were also flagged as low-quality tracks (Supplementary Fig. 8).

Secondary reactivities. In addition to genome-wide quality control of imputed tracks, we also focused on the specific differences between observed and imputed tracks. For each observed mark, we generated a genome-wide 'delta' track, computed as the difference in signal intensity between the observed and the imputed data, rescaling imputed tracks to match the signal intensity properties of the observed tracks, as the observed tracks showed a general bias for higher intensity. Some of these 'delta' tracks showed surprisingly high correlations with 'primary' tracks of non-putative marks, indicating potential secondary antibody reactivities. To flag these reactivities, we compared the average correlation of each of the delta tracks to the top 10 closest imputed tracks for each mark. As with antibody swaps, we fitted a multivariate linear model in each mark combination to flag outliers. We flagged 19 tracks and reported 13 after visual inspection as potential secondary reactivities or single replicate swaps (for example, in the case of DNase-seq) (Supplementary Figs. 7, 8). We noted that some cases showed clear

difference tracks that do not match available antibodies, suggesting that the secondary reactivity is not a common mark in our compendium.

Biological space coverage. To evaluate the similarity of imputed and observed tracks across samples, we calculated the pairwise genomic correlations between all pairs of imputed and observed signal tracks. We hierarchically clustered the imputed or observed correlation matrix of each individual mark using Ward's method. We averaged all imputed matrices for the six main marks (H3K27ac, H3K4me1, H3K4me3, H3K36me3, H3K27me3 and H3K9me3) to create a fused correlation matrix, which we similarly clustered. We plotted the hierarchically clustered tree for the fused matrix alongside the metadata information for each biosample using the circlize R package[51].

In addition, we calculated mark-specific Spearman correlations that were restricted to relevant features within all observed and imputed tracks per mark. We mapped each of the 13 marks to its top state by emission probability in the ChromHMM 25-state model and any other states with emission probability over 80%. For ATAC-seq, we used the same region list as DNase-seq. For each mark, we averaged and reduced each 25-bp signal track to any 200-bp regions that were labelled as one of the states associated with the mark in any of the 127 imputed Roadmap biosamples under the 25-state model[6,20]. We calculated the Spearman correlation between sets of these region-restricted mark signal tracks and generated similarity matrices across all datasets for a mark. Using these Spearman correlation matrices on all observed and imputed signal tracks, we computed UMAP dimensionality reductions for each mark and assay using with the uwot R package[52] with the default parameters, except for n_neighbours = 250, min_dist = 0.25 and repulsion_strength = 0.25.

Epigenomic annotations

Chromatin-state annotations. We computed epigenomic annotations on 3,533 imputed and 1,465 observed datasets for 6 marks on 833 samples using ChromHMM with the fixed 18-state model from Roadmap[6] with the same mnemonics and colours. We used observed data wherever possible, except in cases with no observed data or where observed data were removed in quality control. The table of the signal tracks used to calculate the annotations is available as Supplementary Table 2. The observed data were binarized from signal tracks with a $-\log_{10} P$ value signal cut-off of 2. To binarize the imputed data and facilitate comparison with the observed data, we established mark-specific binarization cut-offs. We first separately calculated the overall probability distributions of all imputed and observed tracks for each mark. Then, for each mark, we set the imputed binarization cut-off value to the value of the quantile that matched the quantile in the observed data for the $-\log_{10} P$ value > 2 cut-off. We used liftOver[48] to map all 833 (after quality control) ChromHMM annotations to GRCh38, using a stringent reciprocal mapping strategy, ensuring that all resulting GRCh38 regions were also 200 bp and non-overlapping, and we have provided these alongside hg19 annotations and as track sets at https://epigenome.wustl.edu/epimap/.

Defining active enhancers. We define active enhancers as the intersection of DHS regions with enhancer annotations and high H3K27ac signal (average signal of >2 in the region containing the DHS ± 100 bp). We defined DHS regions from an index list of 3,591,898 DHS element consensus locations in GRCh38, determined from 733 DNase-seq experiments, that we mapped using liftOver[48] to 3,568,912 hg19 locations[24]. We intersected the hg19 regions with the 833 imputed enhancer annotations (states 7, 8, 9, 10, 11 and 15 in the 18-state model). This resulted in 2,842,995 regions with at least one enhancer annotation in any biosample. Finally, we intersected this matrix with the H3K27ac signal in the ±100-bp region that encompassed each DHS from the same tissue-specific imputed and observed datasets used to calculate the ChromHMM annotations. This procedure resulted in 2,356,914 active-enhancer regions. We created an equivalent promoter element region using the promoter annotations (states 1, 2, 3, 4 and 14 in the 18-state model). We noticed that several regions shared both enhancer and promoter annotations. As a conservative cut-off, we assigned all regions to either enhancers or promoters if over 75% of its active occurrences were labelled as that type of element (Supplementary Fig. 13). This final thresholding procedure yielded 2,069,090 enhancers, 204,104 promoters and 122,358 dyadic elements (neither specifically promoter nor enhancer). The matrices and enhancer locations are available at http://compbio.mit.edu/epimap.

For all images using tissue group order, including ChromHMM tracks and module heat maps, groups were ordered alphabetically within six major groups: tissue or organs (adipose, bone, digestive, endocrine, heart, kidney, liver, lung, mesenchymal, muscle, myosatellite, pancreas, placenta and EEM, reproductive, smooth muscle and urinary), other primary cells (endothelial, epithelial and stromal), blood and immune (blood and T cell, HSC and B cell, lymphoblastoid, spleen and thymus), nervous system (brain, eye, neurosphere and PNS), stem (embryonic stem cell-derived, embryonic stem cell and induced pluripotent stem cell), and other (cancer and other).

Defining enhancer modules. To define enhancer modules, we clustered the binary enhancer matrix defined by intersecting enhancer annotations with DHS regions and with the average centred and flanking (±100 bp) H3K27ac signal above a $-\log_{10} P$ value of 2 using the *k*-centroids algorithm with the Jaccard distance and the number of clusters set to $k = 300$. The average module contained 6,897 enhancers, and the largest module (enumerating constitutive elements) contained 93,554 enhancer regions. In all heat map plots of module centres (and associated enrichment figures), we diagonalized the matrix by ordering each column in the heat map (module centres) by the biosample that contributed the maximal signal. All columns that had a signal over 25% in more than 50% of rows were shown first. We used this diagonalization procedure for all diagonalized heat maps. We coloured each module by the tissue group that contained its maximal signal. Modules highlight sample groupings and organize according to cell type and tissue. Major groups were ordered alphabetically within six major groups and samples were ordered within groups according to Ward method's clustering of the Jaccard distance of the module centres matrix. We performed enrichment on the module centres against the metadata of included samples (signal over 25%) by the hypergeometric test, and show enrichments with $-\log_{10} P > 2$ (Fig. 2b).

Gene ontology enrichment. We performed gene ontology enrichments on each enhancer module using GREAT v3.0.0 for the biological process, cellular component and molecular function ontologies[53]. We analysed and visualized the results in the same manner as in the Roadmap core paper[6]. We only considered enrichments of 2 or greater with a multiple testing-corrected $P < 0.01$. For Fig. 4c, we reduced the gene ontology enrichment by modules matrix to terms with a maximal $-\log_{10} P > 4$ that were enriched in less than 10% of modules. The full enrichment matrix is shown in Supplementary Fig. 16. As in the case of the diagonalized module centres, we labelled each term according to the module containing its maximal signal. We used a bag of words approach (as described in Roadmap[6]) to pick 36 representative terms out of 865 total terms for Extended Data Fig. 6b, such that each tissue group has at least one term and the rest are representatively allocated across groups.

Motif enrichment. We performed motif enrichment analysis across enhancer modules as described in the Roadmap paper[6,54]. In brief, we measured the enrichment of 1,690 motifs consisting of the JASPAR (2018)[55] core non-redundant vertebrate motifs, the HOCOMOCO v11[56] human motif set and the SELEX motifs by Jolma et al.[57]. We computed the enrichments for each of the 1,690 motifs relative to a joint DHS and intergenic background, additionally controlled by 100 shuffled motifs

for each motif. We reported the motif with the highest enrichment in any module for each of the 286 previously identified motif archetypes[26]. We only reported motifs with a maximum log_2-transformed fold change of at least 1, resulting in 160 motif archetypes (corresponding to 1,175 total motifs), which we show with their position weight matrix (PWM) logos against all 300 modules in Extended Data Fig. 6c.

Enhancer–gene linking. We predicted enhancer–gene links for each biosample using the Pearson correlation between gene expression and the histone mark activity of nearby enhancers (within 1 Mb) for six marks (H3K27ac, H3K4me1, H3K4me2, H3K4me3 and H3K9ac). We precomputed correlations between all genes and nearby enhancers across the 304 biosamples with paired expression data. A negative set of correlations for each enhancer was computed using random genes in a different chromosome. We predicted links for each biosample and ChromHMM enhancer state separately (states E7, E8, E9, E10, E11 and E15). Predictions were made by training an XGBoost classifier on the positive set of all valid links against their paired negative links, using precomputed correlations and distance to the transcription start site as features, and keeping all links with a probability above 5/7 (ref. [58]).

We validated enhancer–gene links using curated gold-standard data[59] in CD34, GM12878, HeLa and K562 cells (Extended Data Fig. 8). We compared four sets of correlation-based predictions (alone or with H3K27ac and H3K4me1 activity, and with and without distance-based rescaling) against distance alone, enhancer–gene links from Roadmap, and H3K27ac correlation and/or activity times distance (calculated using EpiMap tracks and enhancers in compared epigenomes)[60]. For methods without a threshold value, such as distance alone, only the nearest or highest score gene was used for each as a cut-off value for F1. In addition, we created a gene ontology-based gold-standard set of links from gene ontology terms that were enriched within enhancer clusters by GREAT[53]. For each gene ontology term per cluster, we added enhancer–gene links for enhancers within 1 Mb of at least two genes in the gene ontology term. Negative link sets were constructed by taking physical and expression quantitative trait locus (eQTL) negative link sets that were also not enriched by gene ontology.

GWAS enrichment analysis

We pruned the NHGRI-EBI GWAS catalogue[34] (downloaded from https://www.ebi.ac.uk/gwas/docs/file-downloads on 3 May 2019) using a greedy approach: within each trait + PMID combination, we ranked associations by their significance (*P* value) and added SNPs iteratively if they were not within 5 kb of previously added SNPs. We also removed all associations in the HLA locus (for hg19: chr6: 29,691,116–33,054,976). This reduced the catalogue from 121,000 to 113,000 associations. Finally, we reduced the catalogue to 926 unique GWAS (from 5,454 GWAS) with an initial sample size of at least 20,000 cases or individuals (wherever cases and controls were not annotated). This resulted in 66,801 lead SNPs, which landed in 33,417 unique genome intervals when we split the genome into 10,000-bp intervals.

Flat GWAS–epigenome enrichments and module-based GWAS–epigenome enrichments. We performed the hypergeometric test to evaluate GWAS enrichments on flat epigenomes and on modules. For these flat enrichments, we compared each number of SNP–enhancer intersections for each enhancer set (flat epigenome or module) to the full set of intersections in all M enhancers. As above, we corrected for multiple testing for each GWAS and enhancer set combination by computing and correcting with null association *P* values for flat epigenomes and modules using the null catalogues generated for the tree enrichment. Rarefaction curves were calculated on the flat epigenome enrichments by iteratively adding the sample that was either significantly enriched or the maximal enrichment for the most remaining GWAS until all GWAS were accounted for (Extended Data Fig. 10c, d).

Tree-based GWAS–epigenome enrichments. We constructed a tree by hierarchically clustering the Jaccard similarity of the binary enhancer-by-epigenomes matrix using complete-linkage clustering. Then, for each node in the tree, we calculated its consensus epigenomic set, defined as the set of all enhancers present in all leaves of the subtree, such that each node's set was a superset of that of its parent. For each GWAS, we asked whether the novel consensus enhancers at a node were significantly enriched for lead SNPs by comparing the enrichment between each node and its parent as measured by the likelihood ratio test between two logistic regressions.

In brief, for each GWAS catalogue unique trait and PubMed ID, we found all intersections of its pruned SNPs with $M = 2{,}069{,}090$ enhancers. Then $\mathbf{Y}$ is an indicator vector of size M, which shows the intersected enhancers. We found all consensus enhancers (the intersection of epigenomes in the subtree) in the node of interest (vector $\mathbf{X_N}$) and in its parent ($\mathbf{X_P}$). All vectors are 1×M. We calculated $\mathbf{X_D} = \mathbf{X_N} - \mathbf{X_P}$ (specific enhancers), which was also in $\{0,1\}^{(1\times M)}$ as each node contained a superset of its parent's enhancers. We then calculated the following two logistic regressions: M1: $\mathbf{Y} \sim \mathbf{X_P} + \mathbf{1}$; M2: $\mathbf{Y} \sim \mathbf{X_P} + \mathbf{X_D} + \mathbf{1}$. We calculated the log-likelihood difference and applied the likelihood ratio test to test whether adding the specific enhancers (M2) was significantly different from the parent model (M1). To correct for multiple testing on a per GWAS and node basis, we generated 1,000 null GWAS for each lead SNP set size by shuffling the trait associations across GWAS locations, giving 243,000 null GWAS in total. We used these catalogues to compute the null association *P* values for each permuted GWAS and used the 0.1% and 1% top quantiles as false discovery rate cut-offs.

For the CAD example, gene ontology terms[61] were calculated using the nearest gene of each enhancer hit by a lead SNP. We pruned genes to expressed genes by calculating the average RNA-seq profiles for each tissue group and excluded genes that had log_2 FPKM < 2 in the average RNA-seq of each sample's group. Of 833 samples, 341 samples have matched RNA-seq, which we list in addition to releasing the processed data at http://compbio.mit.edu/epimap. We kept only the gene ontology terms that were significant in 25% or less of nodes, and report the top two gene ontology terms per node in Fig. 4d and all gene ontology terms in Supplementary Fig. 26.

For locus investigations (in *NTN4*, *CACNA1C*, *EDNRA* and *PLPP3*), we found the nearest active enhancer to each lead SNP in each node (within 2.5 kb), plotted the H3K27ac signal in the 2.1 million enhancers only, and (1) directly mapped links that originated at one of the enhancers near a lead SNP in the top three enriched epigenomes or (2) any links in the locus present in at least half of the samples in one of the selected tissue groups.

Tissue similarity. We assigned each internal node in the tree to a unique tissue if over 50% of the leaves of the subtree came from that tissue and as 'multiple' if the subtree was not the majority of one tissue. We assigned tissue labels to 641 of 832 (77%) internal nodes where the majority of leaves corresponded to a single group. Using these assignments, we created a tissue by GWAS matrix by adding the $-\log_{10} P$ values for each tissue node set from all of the GWAS enrichments on the tree. We binarized this matrix and computed the Jaccard similarity across tissues to calculate a tissue similarity matrix. To assess the significance of tissue overlap, we compared each overlap value against the overlaps from 10,000 permuted enrichments. We collapsed each permuted matrix into a tissue by the GWAS matrix to compute the overlaps under the null. We performed the permutations for each tissue against other tissues by shuffling the enrichment *P* values on the node by the GWAS matrix. Specifically, we (1) binarized the enrichment matrix, (2) fixed the column of the group of interest, (3) permuted the remainder of the matrix, keeping its row and column marginals the same, and then (4) calculated the cosine distance between the permuted and the original matrix of enrichments.

Cross-GWAS network. To evaluate the cross-GWAS similarity, we normalized the tissue by the GWAS matrix for each GWAS to obtain the proportion of significance attributed to each tissue for each GWAS (Supplementary Fig. 21). We reduced the matrix to 538 significant GWAS with at least 20,000 cases (or individuals when no cases were specified) passing a false discovery rate correction at 0.1% at the per-node and per-GWAS size level. We created a GWAS–GWAS network using the cosine distance matrix as an adjacency matrix, keeping 5,547 links with a cosine distance of 0.25 or less. We used the Fruchterman–Reingold algorithm to lay out the graph[62]. We used the tissue by the GWAS matrix to colour links according to the maximum tissue in the product between each pair of nodes and to colour nodes according to the maximal tissue for each node (Supplementary Fig. 22).

To compare the epigenetic network to trait genetic similarity, we binned SNPs in the GWAS catalogue into 10-kb windows starting from the beginning of each chromosome. We counted the number of intersecting bins between two traits and kept any trait pairs with Jaccard similarity of at least 1%. To compare this to the epigenetic network, we plotted only links in the epigenetic network that coincided with any SNP-sharing GWAS pairs. In addition, we plotted the heat maps of the tree enrichments distance matrix and the genetic similarity matrix side by side, first organized by hierarchically clustering the enrichments matrix and then by clustering the genetic similarity matrix (Supplementary Figs. 23–25).

Reporting summary

Further information on research design is available in the Nature Research Reporting Summary linked to this paper.

Data availability

We provide all imputed and processed observed tracks along with ChromHMM annotations and track sets for the 859 imputed and the final 833 quality controlled samples at https://epigenome.wustl.edu/epimap[21]. All other processed and intermediate datasets, including metadata (Supplementary Tables 1, 2), flagged samples, annotations, DHS locations, enhancer and promoter definitions, enhancer and promoter matrices, modules and matched RNA-seq data can be found at http://compbio.mit.edu/epimap. We also provide an interactive data and analysis browser through the website, including biosample and track exploration, the creation of custom track hubs, modules and motifs enrichments, and per-GWAS investigations for each of the GWAS and their lead SNPs[63] (Supplementary Fig. 27).

Code availability

ChromImpute can be found at http://www.biolchem.ucla.edu/labs/ernst/ChromImpute. The analysis was performed with R (3.5 and 3.6) and Python 3.7. The analysis code is available at http://compbio.mit.edu/epimap.

47. Karimzadeh, M., Ernst, C., Kundaje, A. & Hoffman, M. M. Umap and Bismap: quantifying genome and methylome mappability. *Nucleic Acids Res*. **46**, e120 (2018).
48. Rosenbloom, K. R. et al. The UCSC Genome Browser database: 2015 update. *Nucleic Acids Res*. **43**, D670–D681 (2015).
49. Kharchenko, P. V., Tolstorukov, M. Y. & Park, P. J. Design and analysis of ChIP-seq experiments for DNA-binding proteins. *Nat. Biotechnol*. **26**, 1351–1359 (2008).
50. Feng, J., Liu, T., Qin, B., Zhang, Y. & Liu, X. S. Identifying ChIP-seq enrichment using MACS. *Nat. Protocols* **7**, 1728–1740 (2012).
51. Gu, Z., Gu, L., Eils, R., Schlesner, M. & Brors, B. circlize implements and enhances circular visualization in R. *Bioinformatics* **30**, 2811–2812 (2014).
52. Leland, M., Healy, J. & Melville, J. Umap: uniform manifold approximation and projection for dimension reduction. Preprint at https://arxiv.org/abs/1802.03426 (2018).
53. McLean, C. Y. et al. GREAT improves functional interpretation of cis-regulatory regions. *Nat. Biotechnol*. **28**, 495–501 (2010).
54. Kheradpour, P. & Kellis, M. Systematic discovery and characterization of regulatory motifs in ENCODE TF binding experiments. *Nucleic Acids Res*. **42**, 2976–2987 (2014).
55. Khan, A. et al. JASPAR 2018: update of the open-access database of transcription factor binding profiles and its web framework. *Nucleic Acids Res*. **46**, D1284 (2018).
56. Kulakovskiy, I. V. et al. HOCOMOCO: towards a complete collection of transcription factor binding models for human and mouse via large-scale ChIP-seq analysis. *Nucleic Acids Res*. **46**, D252–D259 (2018).
57. Jolma, A. et al. DNA-binding specificities of human transcription factors. *Cell* **152**, 327–339 (2013).
58. Liu, Y., Sarkar, A., Kheradpour, P., Ernst, J. & Kellis, M. Evidence of reduced recombination rate in human regulatory domains. *Genome Biol*. **18**, 193 (2017).
59. Moore, J. E., Pratt, H. E., Purcaro, M. J. & Weng, Z. A curated benchmark of enhancer–gene interactions for evaluating enhancer–target gene prediction methods. *Genome Biol*. **21**, 17 (2020).
60. Fulco, C. P. et al. Activity-by-contact model of enhancer-promoter regulation from thousands of CRISPR perturbations. *Nat. Genet*. **51**, 1664–1669 (2019).
61. Yu, G., Wang, L.-G., Han, Y. & He, Q.-Y. clusterProfiler: an R package for comparing biological themes among gene clusters. *OMICS* **16**, 284–287 (2012).
62. Csardi, G. & Nepusz, T. The igraph software package for complex network research. *InterJournal Complex Syst*. **1695**, 1–9 (2006).
63. Chang, W. et al. shiny: web application framework for R. R package version 1 (2017).
64. The Gene Ontology Consortium. Gene ontology: tool for the unification of biology. *Nat. Genet*. **25**, 25–29 (2000).

Acknowledgements We thank the ENCODE, Roadmap and GGR consortia for generating high-quality public datasets and rapidly disseminating their results to the broader community; D. Li and T. Wang, and I. Gabdank and J. S. Strattan for making our observed and imputed genome-wide tracks and chromatin-state annotations available through the WashU Epigenome Browser and the ENCODE portal, respectively; J. Ernst for advice, guidance and for developing the ChromImpute methodology and code base; P. Kheradpour for help with the motif enrichment analysis software; L. D. Ward for discussions on interactive visualizations of our predictions and HaploReg; C. Epstein, J. Schreiber, W. Noble, Z. Weng, M. Gerstein, ENCODE, Roadmap, GENCODE and GTEx consortia for feedback on early versions of this work; and I. Jungreis, X. Wang, L. Hou, L. Agudelo, S. Mohammadi, M. Wolf, A. Shi, K. Nguyen, M. Kousi, S. Kuosmanen, E. Schmauch and A. Amirabad for feedback on the work and the resource. This work was supported by the US National Institutes of Health grants HG008155, HG009446, HG009088, HG007234, HG007610, GM113708, MH109978, MH119509 and AG058002 (to M.K.) and the National Institutes of Health training grant GM087237 (to C.A.B.).

Author contributions C.A.B. and M.K. designed the study, analysed the data and wrote the manuscript, with input from all other authors. C.A.B. developed and applied computational methods with input from M.K. C.A.B. and B.T.J. carried out the enhancer–gene linking analysis. Y.P.P. contributed to the genetics analysis. W.M. contributed to the DNase and chromatin-state analyses. M.K. supervised the work.

Competing interests The authors declare no competing interests.

Additional information
Supplementary information The online version contains supplementary material available at https://doi.org/10.1038/s41586-020-03145-z.
Correspondence and requests for materials should be addressed to M.K.
Peer review information *Nature* thanks Ting Wang and the other, anonymous, reviewer(s) for their contribution to the peer review of this work.
Reprints and permissions information is available at http://www.nature.com/reprints.

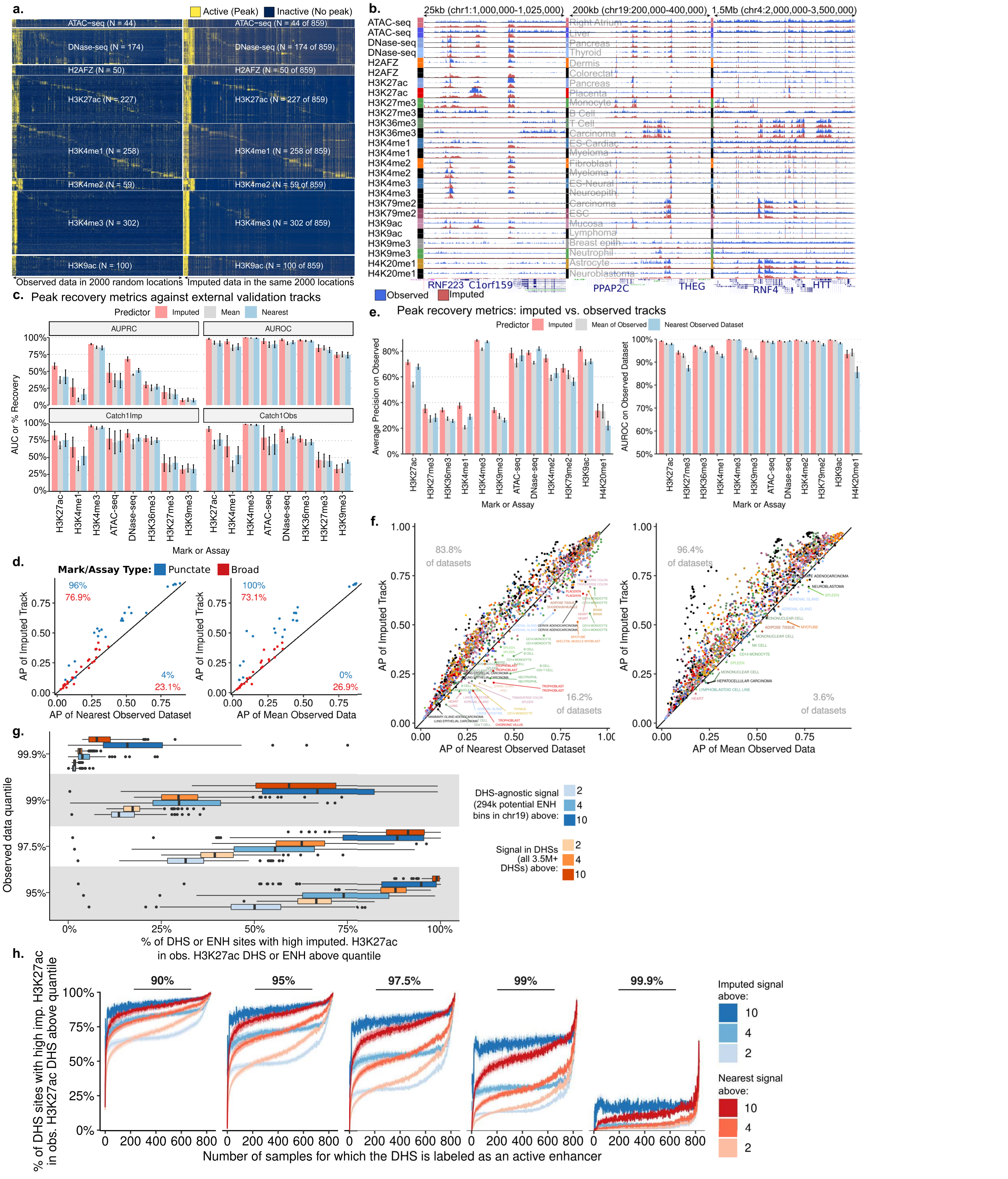

Extended Data Fig. 1 | See next page for caption.

Extended Data Fig. 1 | Imputation validation. a, Heat map of paired observed and imputed signal intensity across all punctate Tier 1 and Tier 2 assays across 2000 highest-max-signal bins among 5000 randomly-selected 25bp bins. Samples (rows) and bins (columns) are clustered and diagonalized using maximum imputed signal intensity, with broadly-active regions shown first. **b**, Paired observed (blue) and imputed (red) tracks for all Tier 1 and Tier 2 assays in three regions at different resolutions for randomly-selected samples. Each row shows a single track across three different resolutions. Full tracks at https://epigenome.wustl.edu/epimap. **c**, Genome-wide imputation performance metrics for predicting 51 external validation tracks across 8 assays in 14 biosamples (average precision, AUROC predicting top 1% of observed data and peak recovery of top 1% Imputed or Observed with top 5% Observed or Imputed, respectively) in chr1, shown for either the appropriate imputed track, the best-matching of the other observed tracks, or the observed signal average. **d**, Scatter comparison of average precision (AP) of imputed data with either nearest observed track or signal average in punctate (blue) and broad (red) marks. **e**, Genome-wide imputation performance metrics (AP, AUROC) for predicting observed tracks (evaluated on all observed tracks with an imputed prediction) in chr19, shown for either the appropriate imputed track, the best-matching of the other observed tracks, or the observed signal average. **f**, Scatter comparison of average precision (AP) of imputed data with either nearest observed track or signal average across all datasets, coloured by sample group. Cases where the nearest sample or the mean heavily outperformed the imputation are labelled (points with over 25%, for nearest, or 10%, for mean, greater average precision than the imputed track). **g**, Sample-specific percentage of the 2M DHSs with imputed H3K27ac above a certain cut-off that are also in the top 10%, 5%, 2.5%, 1%, and 0.1% of 3.6M DHSs by matched observed datasets. **h**, Sample-specific percentage of the 2M DHSs with imputed (blue) or nearest observed (red) H3K27ac above a certain cut-off that are also in the top 10%, 5%, 2.5%, 1%, and 0.1% of 3.6M DHSs by matched observed datasets, partitioning the DHSs by the number of samples in which each DHS is called as an active enhancer.

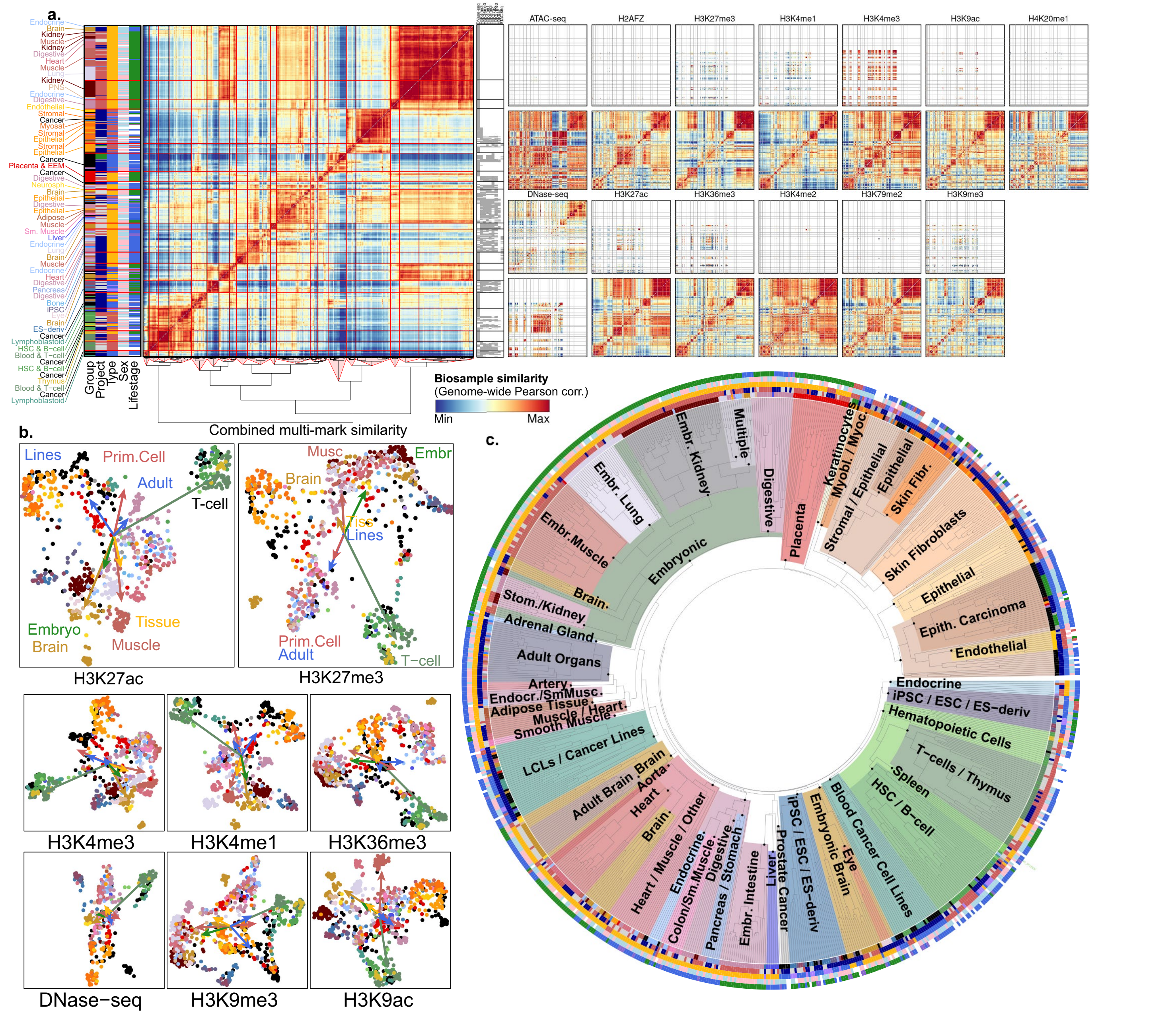

Extended Data Fig. 2 | Cross-sample relationships. a, Hierarchically clustered genome-wide correlation across samples in all 13 imputed Tier 1 and 2 assays. Observed (top) vs. imputed (bottom) matrices shown. Clustering conducted on the fused matrix (left panel, constructed as in main figure). Observed data availability matrix (grey is available, white is unavailable) is shown for the top nine marks and accessibility assays by number of observed datasets. **b**, Two-dimensional embeddings of Tier 1 and 2 marks coloured by tissue group, using Spearman correlation within matched chromatin states. Arrows point from the centre of mass of all biosamples to that of the specified group. **c**, Hierarchical clustering of 833 biosamples based on enhancer activity distances (Supplementary Fig. 12). Subtrees enriched for specific sample types are highlighted and labelled (colours). Samples are labelled by metadata in the outer ring (Supplementary Table 1).

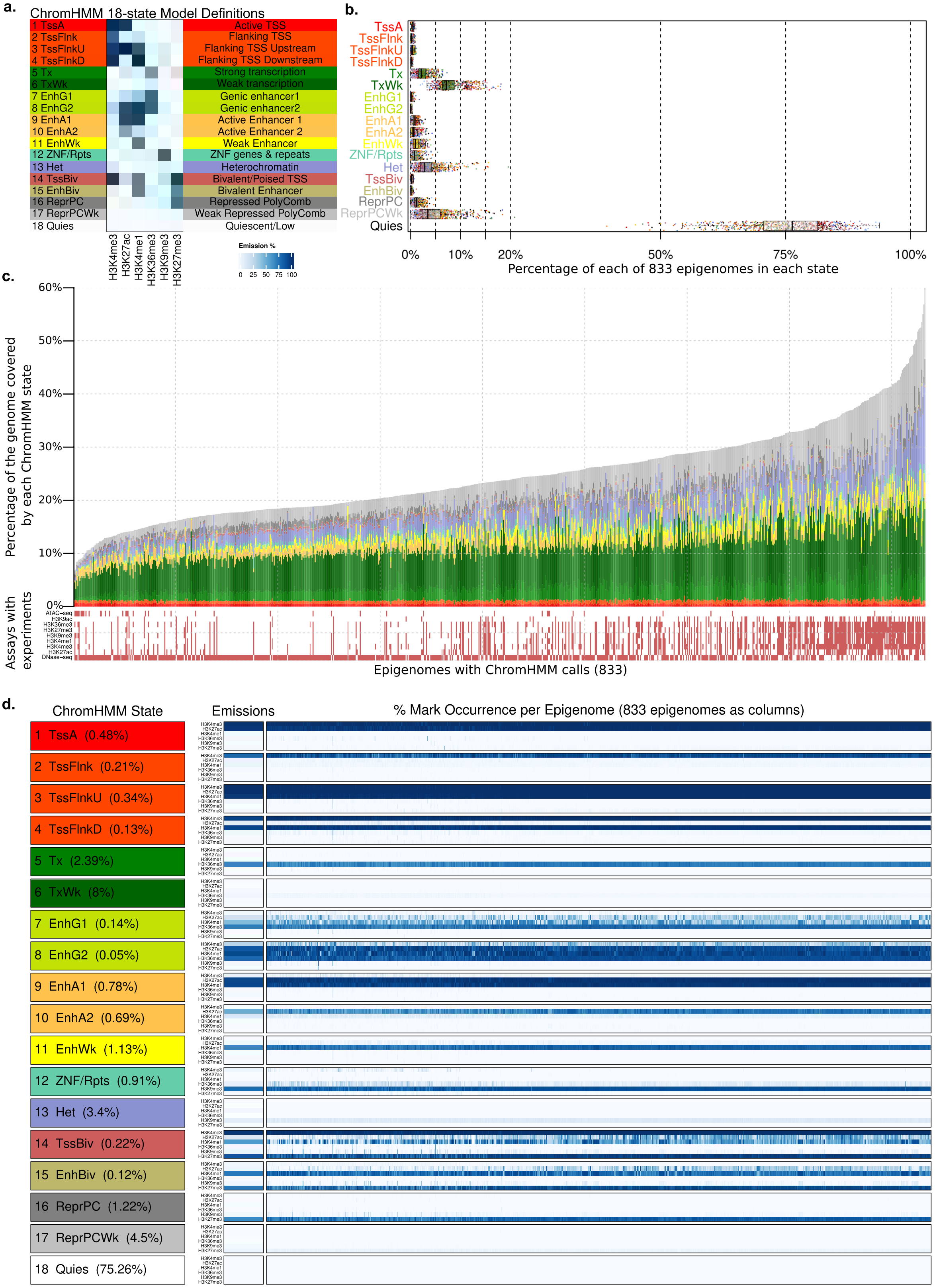

Extended Data Fig. 3 | See next page for caption.

Extended Data Fig. 3 | Chromatin states. **a**, Epigenomic state mnemonics for ChromHMM 18-state model (left) with emissions matrix (centre) and state definitions (right). The 18-state model was trained on Roadmap data for the Roadmap 2015 paper. **b**, Distributions of per-state genome coverage (box-plots) across 833 biosamples (points, coloured by tissue group) according to the ChromHMM 18-state model annotations. **c**, Genome coverage for each 18-state-model ChromHMM state across 833 biosamples after QC. Lower panel shows availability of 9 top marks, ordered by the number of observed datasets. Biosamples are ordered by per cent of the genome not annotated as quiescent. **d**, Comparison of per state (left panel) model emissions (middle panel) against mark occurrence in state calls (right panel) across 833 biosamples (columns in right panel). Observed occurrence matched the emissions closely, with three exceptions, corresponding to bivalent chromatin states and transcribed enhancers (cumulatively covering 0.48% of the genome on average), which showed discrepancies for 12.1% of biosamples on average, likely stemming from their low frequency in the genome, and the frequent co-occurrence of H3K27ac and H3K4me1.

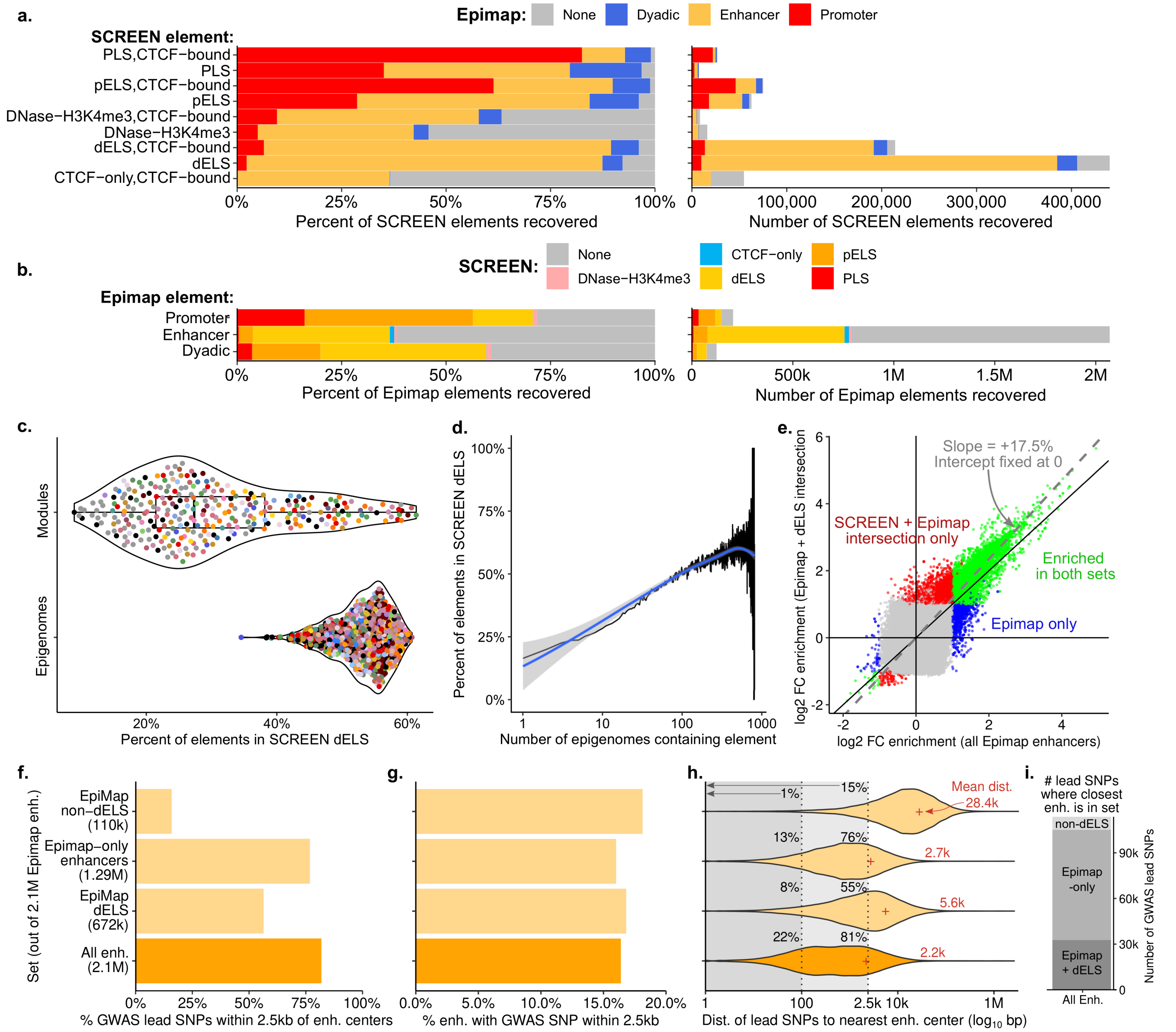

Extended Data Fig. 4 | Comparison with SCREEN. a, Recovery of each category of SCREEN elements by each category of EpiMap elements, percentage (left) and number (right). **b**, Recovery of each category of EpiMap elements by each category of SCREEN elements, percentage (left) and number (right). **c**, Percentage of EpiMap enhancers in dELS in each of 300 modules and 833 epigenomes. **d**, Percentage of EpiMap enhancers in dELS by number of epigenomes containing element (blue line represents loess smooth). **e**, Comparison of motif-module log2 fold change enrichments for all enhancers and for the intersection of enhancers and dELS. **f**–**i**, Comparison of enhancer sets within EpiMap enhancers (intersections with dELS, non-dELS, unique, and all enhancers), showing percentage of 113k pruned GWAS catalogue lead SNPs within 2.5kb of enhancer centres (**f**), per cent of enhancers with a GWAS SNP within 2.5kb of their centre (**g**), the distribution of distances of GWAS SNPs to their nearest enhancer within each set (**h**), and the number of SNPs for which the nearest enhancer fell into each of the constituent sets (**i**).

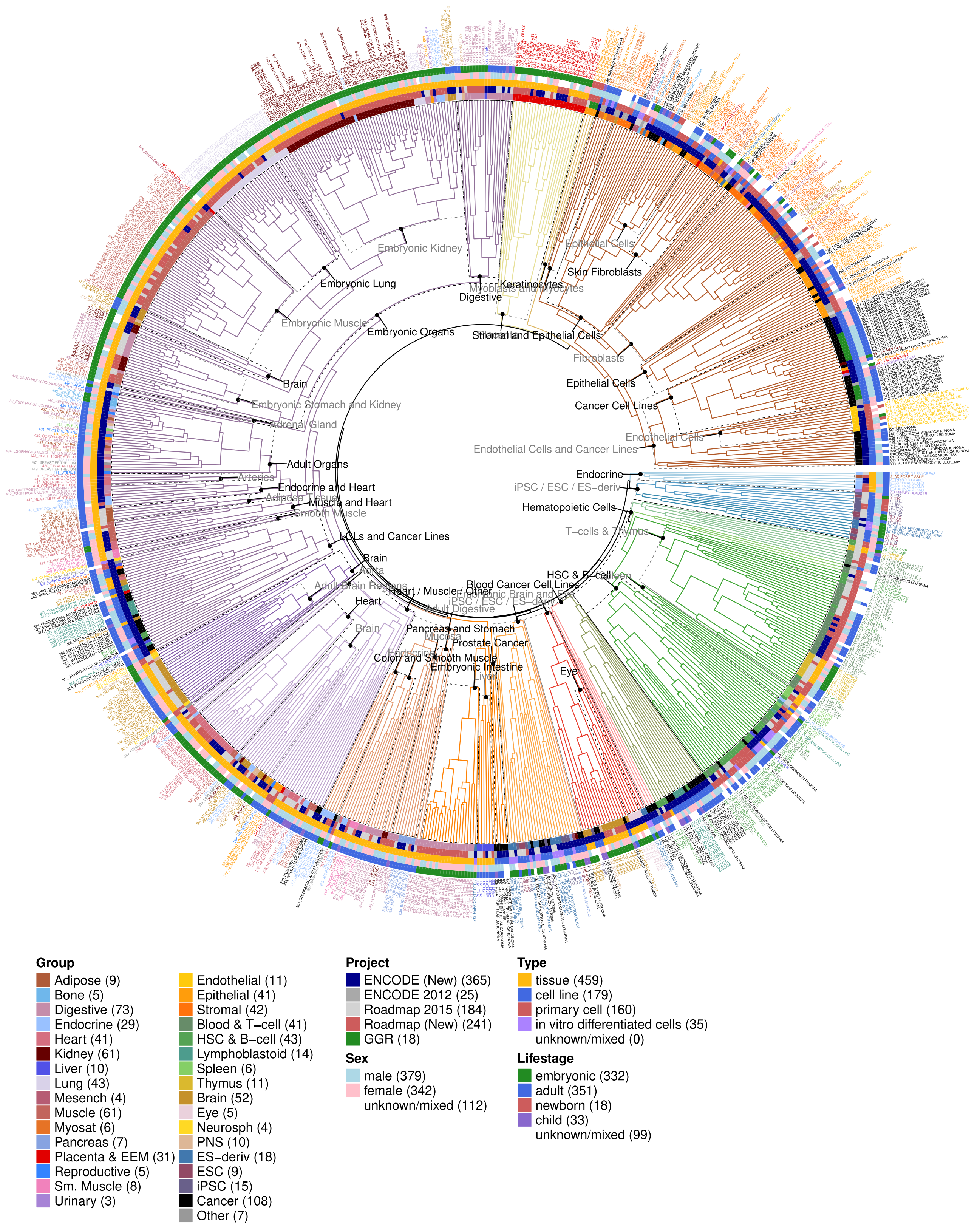

Extended Data Fig. 5 | Enhancer-sharing sample tree. Tree constructed from complete-linkage hierarchical clustering of Jaccard similarity matrix of enhancer activity across biosamples. Fifty subtrees covering the full tree are annotated with their major subgroups. Tree is cut and coloured to create 20 clusters for purposes of visualization. Leaves are labelled with metadata and reduced sample names and coloured according to their tissue group. Metadata (heat map track) from inside-out: tissue group, project, sample type, and donor sex and life stage.

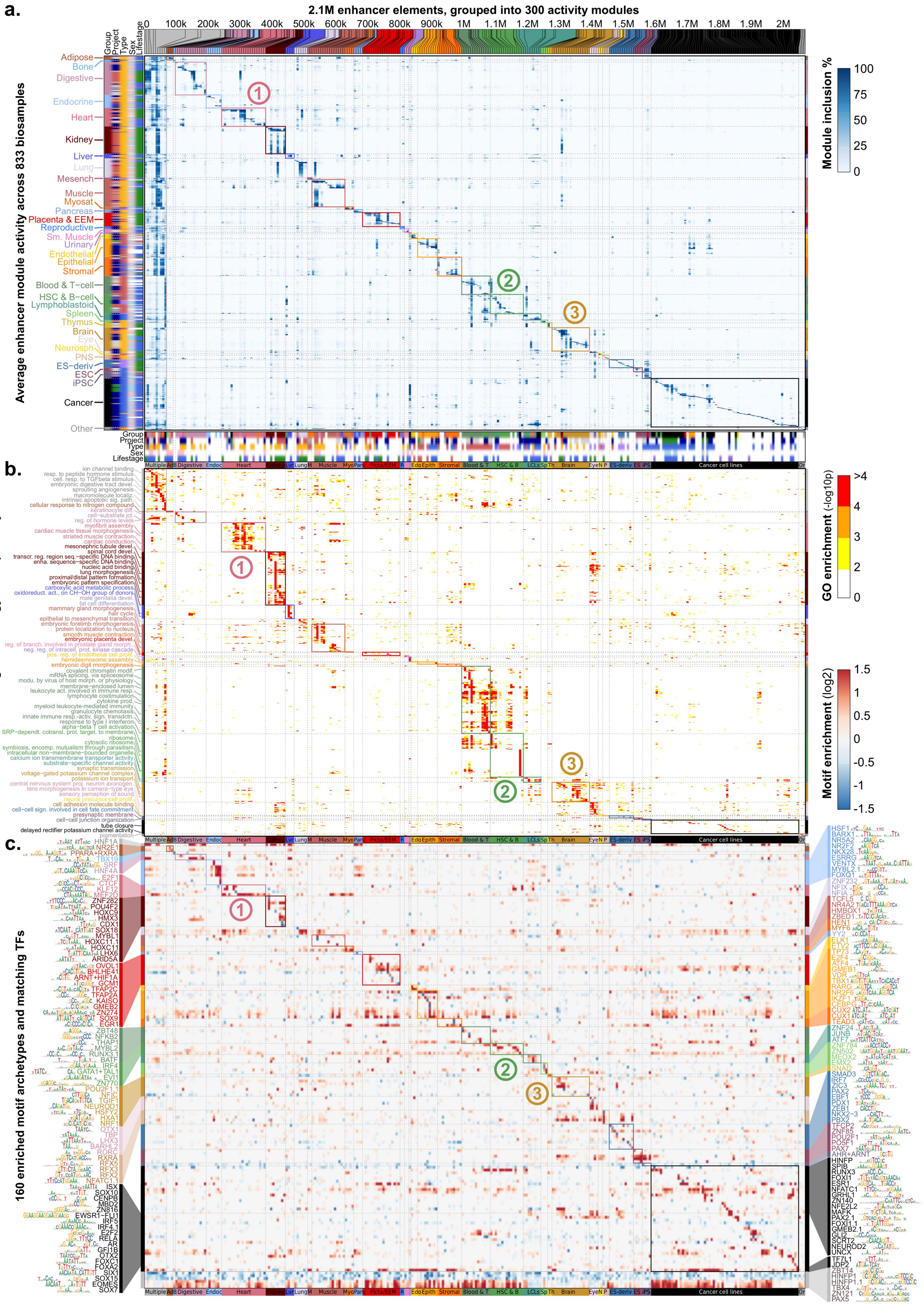

Extended Data Fig. 6 | Expanded enhancer module circuitry. a, Clustering of 2.1M enhancer elements (top) into 300 modules (columns) using enhancer activity levels (heat map) across 833 samples (rows), quantified by H3K27ac levels within accessible enhancer chromatin states. Bottom panel shows enrichment of each module for each metadata annotation, highlighting 34 groups of modules (separated by dotted lines): 33 sample-type-specific (coloured boxes) and 1 multiply-enriched (left-most). **b**, Gene ontology[53,64] (GO) enrichments (heat map) for each module (columns) across 865 terms (rows) with $P < e\text{-}4$. GO terms coloured by maximal enrichment group. Only 36 representative terms are shown, chosen by a bag-of-words approach within each tissue group. **c**, Motif enrichment (heat map) for each module (columns) across 160 motif clusters (rows) with enrichment $\log_2FC > 1$. Motifs coloured by module of maximal enrichment.

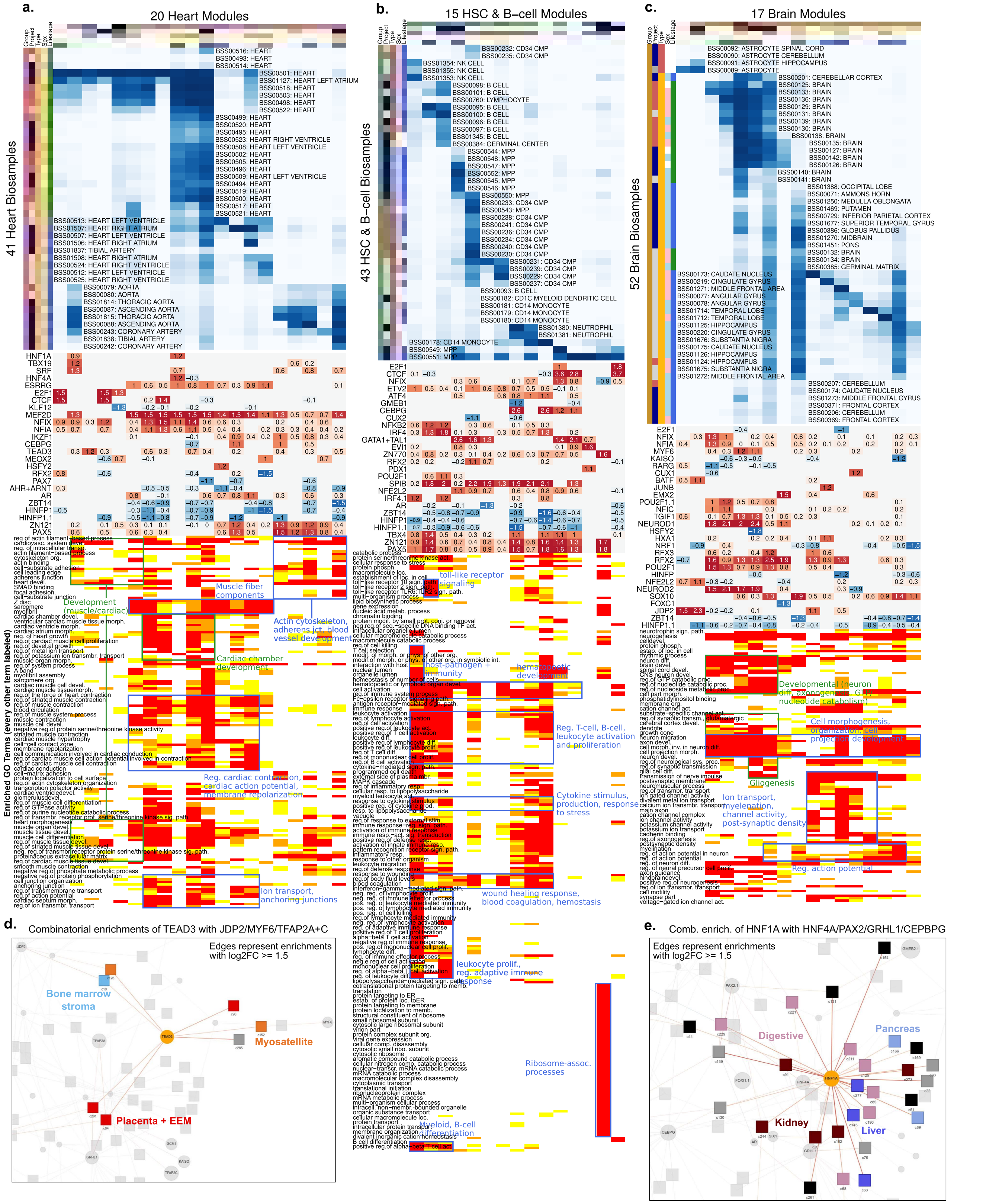

Extended Data Fig. 7 | Expanded module enrichments and motif networks. **a**–**c**, Enhancer module circuitry for heart (**a**), brain (**b**), and haematopoietic cells (**c**) expanding module subsets (Fig. 2d). From top to bottom, we show module centres for all samples in each group against all modules whose maximal inclusion lies in the group, motifs with over twofold enrichment (text: $\log_2$FC), and GO enrichments for each module with tissue-category-specific enrichments. **d**, **e**, Snapshots of the motif-module network highlighting TEAD3 and HNF1A. Edges represent enrichments with $\log_2$FC > = 1.5.

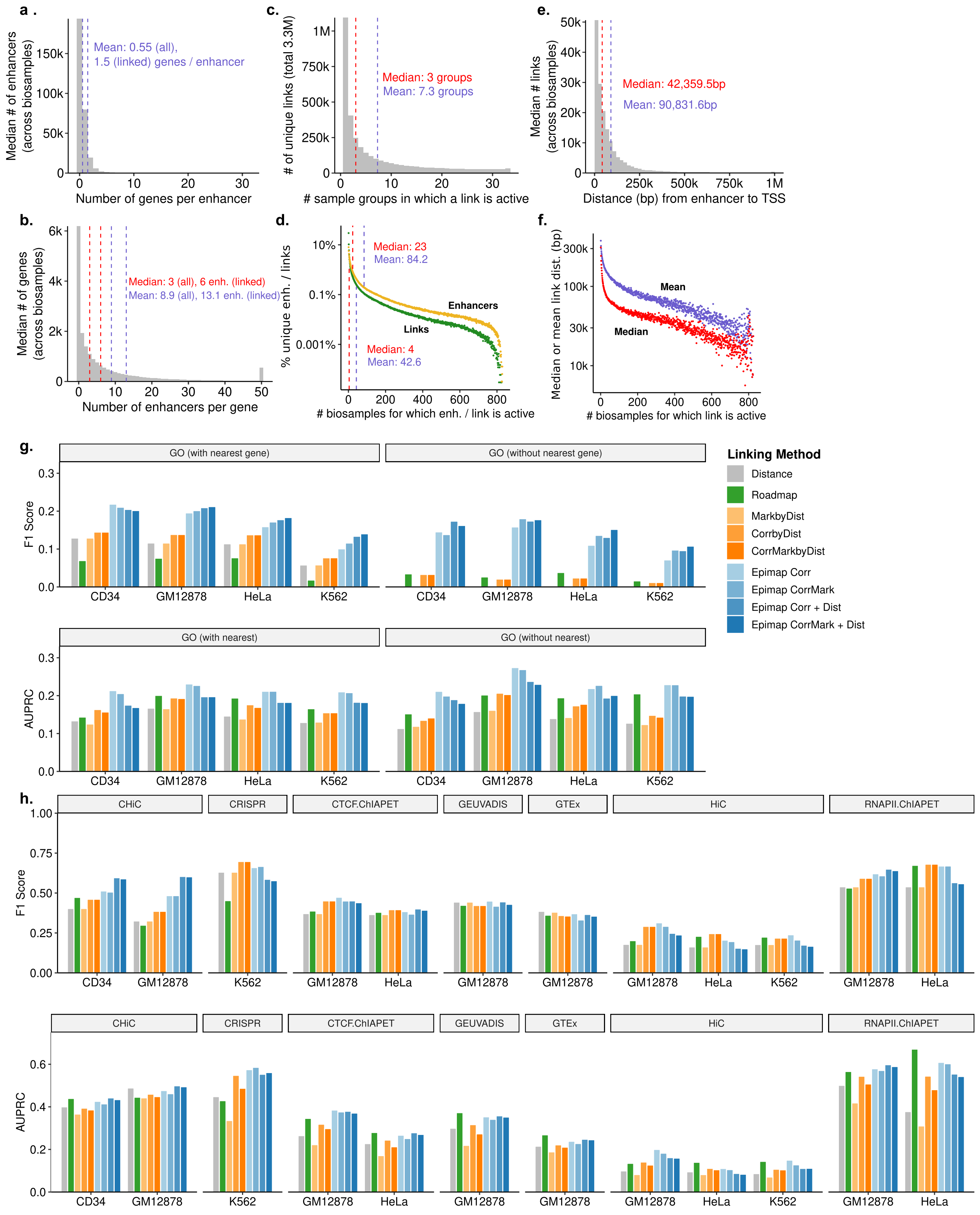

Extended Data Fig. 8 | Linking statistics and validation. **a**–**f**, Enhancer-gene linking statistics. **a**, Bar chart of median number of genes per enhancer per bin across biosamples. **b**, Bar chart of median number of enhancers per gene per bin across biosamples. **c**, Total number of sample groups in which a unique link is active, out of 3.3M unique links. **d**, Per cent of unique elements (enhancers or links) for which the element is active in a given number of biosamples. **e**, Median distance between the enhancer and TSS of a link per distance bin across biosamples. **f**, Mean or median link distance for all enhancers active in a given number of biosamples. All means represented by blue dashed lines and text and medians by red dashed lines and text. **g**, **h**, Comparison (prediction F1 score and AUPRC) of gene-enhancer link predictions (blue) with distance, activity by distance, correlation by distance, correlation and activity by distance, and Roadmap Epigenomic links on functional gene set-based links (**g**) and physical, genetic, and perturbation-based links across four cell lines (**h**).

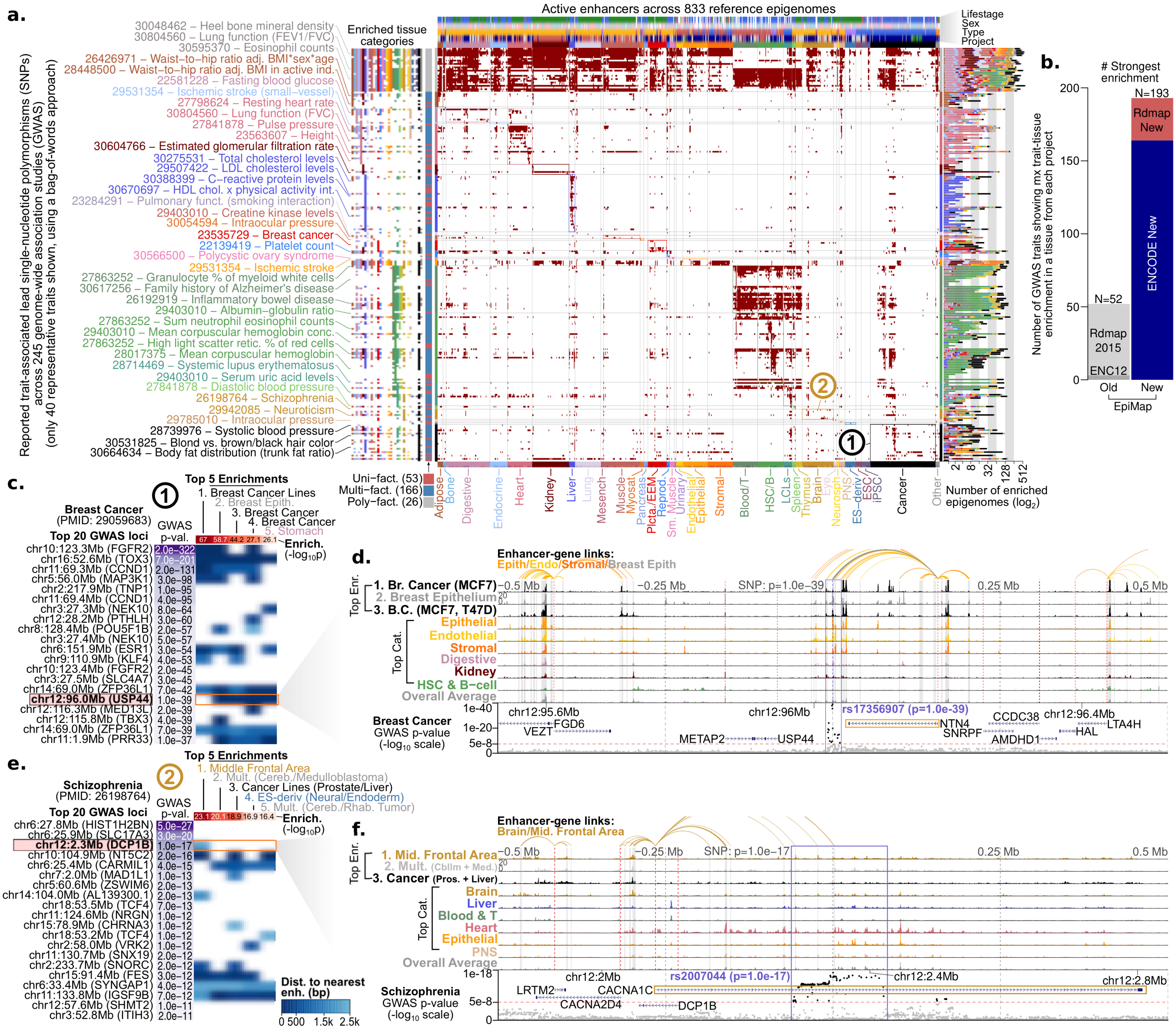

Extended Data Fig. 9 | GWAS tissue-prioritization. a, Trait-tissue enrichment (centre, heat map) between reported lead single-nucleotide polymorphisms (SNPs) from 245 genome-wide association studies (rows) and accessible active enhancers across 833 biosamples (columns) (FDR <1%). Enriched tissue groups (left) and number of enriched biosamples (right) shown for each trait. Only 40 representative traits labelled, using a bag-of-words approach (full list of traits in Supplementary Fig. 19). Traits coloured by sample with maximal trait-tissue enrichment. **b**, Contribution of each project to the maximum GWAS trait-tissue enrichment for the 245 traits with significant enrichments. **c**, Enhancer overlaps with top 20 lead SNPs for breast cancer for top 5 enrichments on the enhancer tree. **d**, 1-Mb locus centred on breast cancer lead SNP rs17356907 (chr12:96027759, P = 1.0e-39) showing H3K27ac signal (middle panel) in enhancer DHSs for the top three enriched subtrees in the enhancer tree, six selected tissue categories, and overall average signal. TSSs indicated by red dashed lines. Top panel shows enhancer gene-links for SNP-proximal enhancers for the top enrichments and across the locus for epithelial, endothelial, and stromal cell biosamples. Linked enhancers are highlighted in grey. Bottom panel shows gene models and breast cancer GWAS summary statistics, with SNPs below P = 5e-8 in grey. **e**, Enhancer overlaps with top 20 lead SNPs for schizophrenia for top 5 enrichments on the enhancer tree. **f**, 1-Mb locus centred on schizophrenia lead SNP rs2007044 (chr12:2344960, P = 1.0e-17), with links (top), H3K27ac signal (middle), and summary statistics as in **d**, with links for brain biosamples.

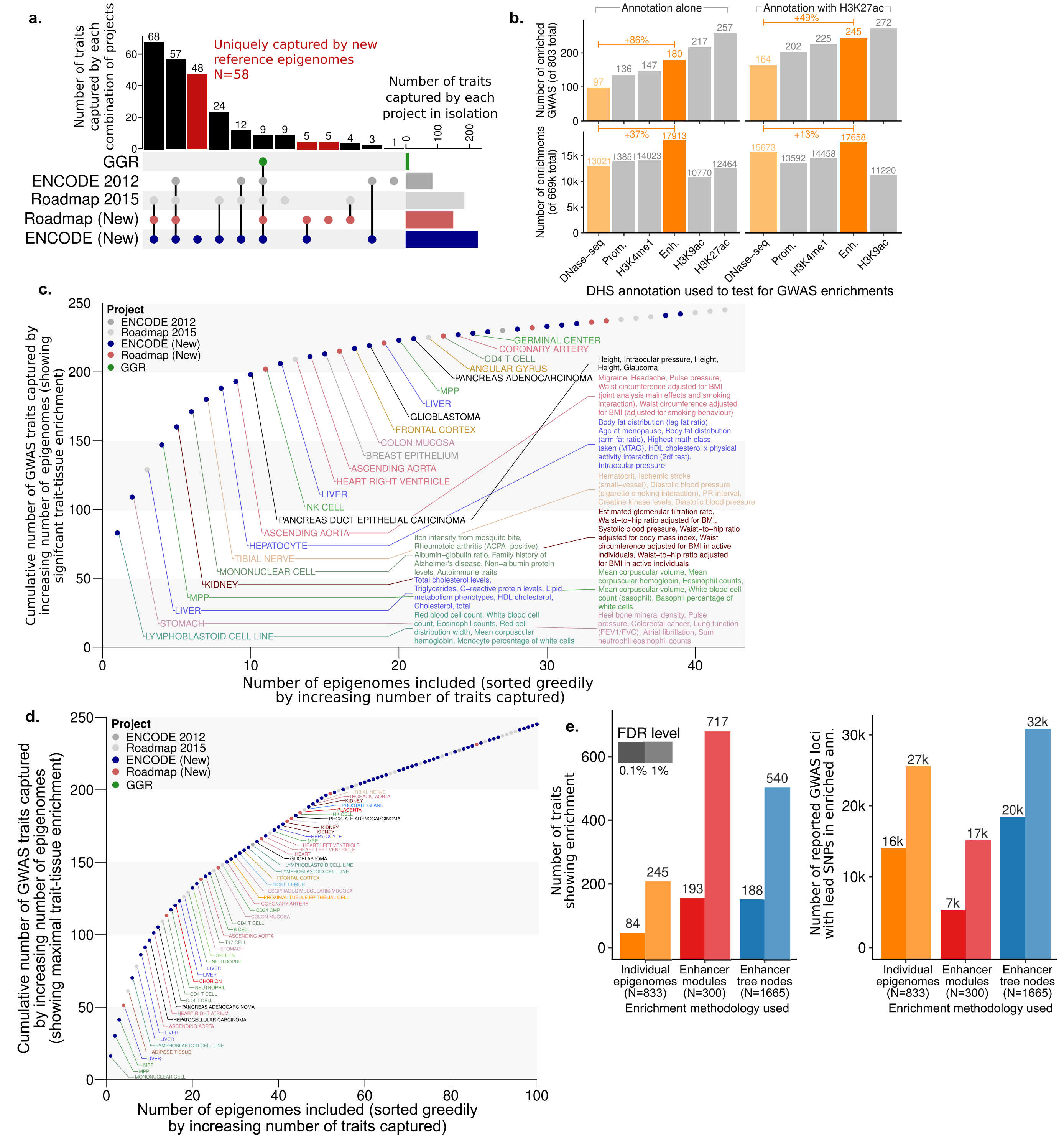

Extended Data Fig. 10 | GWAS prioritization statistics. a, Number of traits (y-axis) with significant GWAS trait-tissue enrichments for each combination (column) of projects (rows). **b**, Comparison of GWAS enrichments found (top) and number of significant trait-tissue pairs SNPs in significantly-enriched annotations (bottom) using different annotations within DHSs either without (left) or with H3K27ac signal (right). DNase-seq signal alone enriches for far fewer GWAS than enhancer states alone or with H3K27ac. **c**, Increase in the cumulative number of GWAS traits (y-axis) with significant trait-tissue enrichments with increasing numbers of biosamples (x-axis), ordered to maximize the number of novel trait annotations captured with each new biosample. Top 25 samples labelled and coloured by tissue group, with top 6 GWAS traits shown for the first 8 samples. Points coloured by project. All 245 traits are captured after inclusion of 42 samples. **d**, Increase in the cumulative number of GWAS traits (y-axis) with maximal trait-tissue enrichments with increasing numbers of biosamples (x-axis). All 245 traits are captured after inclusion of 100 samples. **e**, Comparison of GWAS enrichments found (y-axis, left) and number of lead SNPs in significantly-enriched annotations (y-axis, right) using different methodologies (x-axis) for two FDR cut-offs (shades).

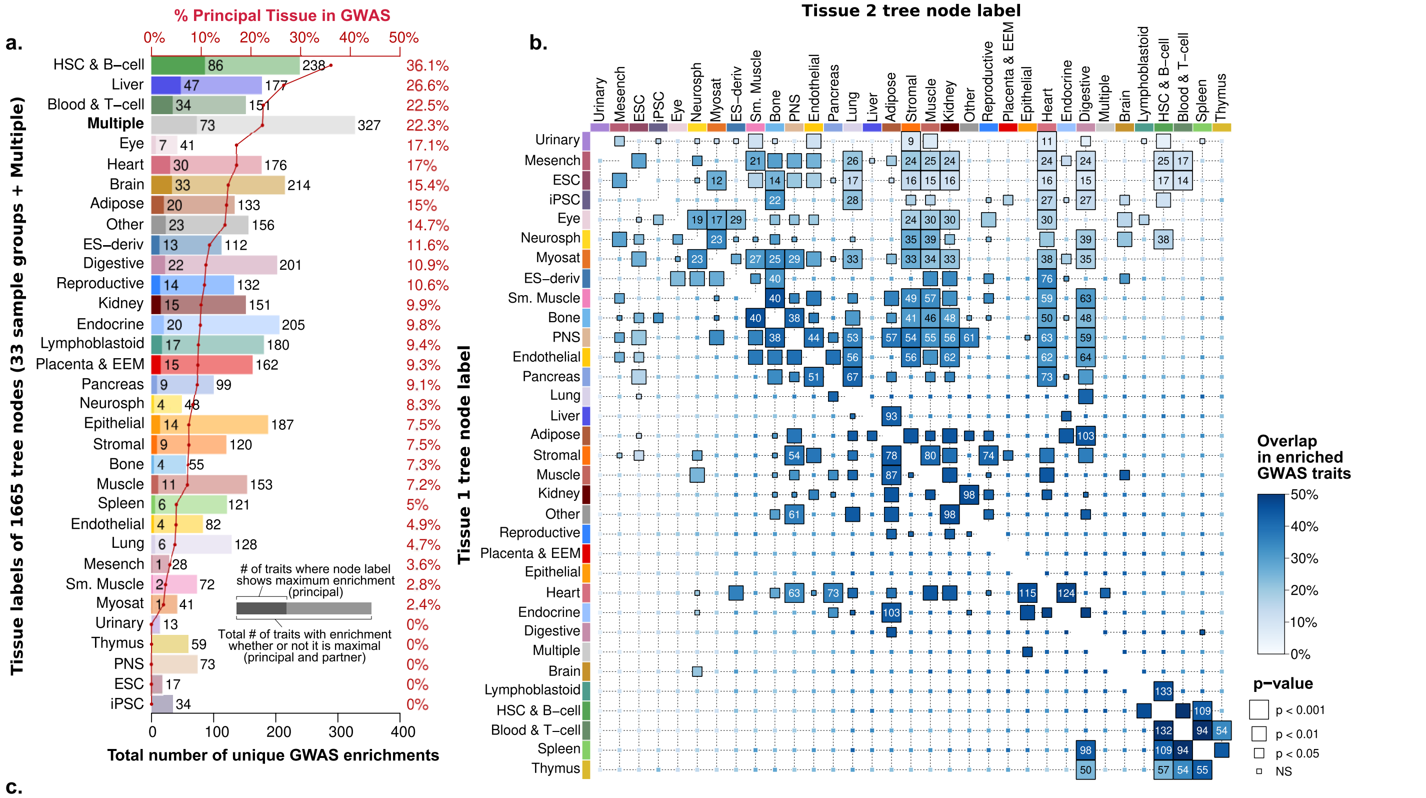

c.

T1	T2	Traits enriched in both tissues (tree node labels)
Liver	Adipose	Cholesterol, total (80%), HDL cholesterol (75%), HDL cholesterol (66.7%), LDL cholesterol (55.6%), Total cholesterol levels (55.6%)
	Digestive	HDL cholesterol (75%), Attention deficit hyperactivity disorder or cannabis use (60%), Coronary artery disease (50%), Total cholesterol levels (50%), Gallstone disease (50%)
	HSC & B−cell	C−reactive protein levels (85.7%), Basophil percentage of granulocytes (84.6%), Mean corpuscular volume (80%), Mean corpuscular volume (76.5%), Serum total protein level (75%)
	Blood & T−cell	Mean corpuscular hemoglobin concentration (53.3%), Coronary artery disease (50%), Eosinophil percentage of granulocytes (46.2%), Lipid traits (pleiotropy) (HIPO component 2) (42.9%), Fibrinogen (37.5%)
	Pancreas	Lipid metabolism phenotypes (50%), Fibrinogen (25%), Fibrinogen levels (18.6%), High density lipoprotein cholesterol levels (15.9%)
Heart	Bone	Atrial fibrillation (100%), Atrial fibrillation (37%), Atrial fibrillation (28.2%), Lung function (FVC) (21.7%), Pulse pressure (19%)
	Adipose	Resting heart rate (40%), Atrial fibrillation (37%), Atrial fibrillation (29.5%), Pulse pressure (25%), Diastolic blood pressure (25%)
	Muscle	Atrial fibrillation (43.8%), Atrial fibrillation (41%), Resting heart rate (40%), Systolic blood pressure(cigarette smoking interaction) (33.3%), Diastolic blood pressure (cigarette smoking interaction) (31.2%)
	Endocrine	QRS duration (66.7%), Electrocardiographic traits (63.6%), QRS duration (62.5%), Factor VIII levels (60%), QT interval (56.2%)
Adipose	Endothelial	Diastolic blood pressure (66.7%), Waist−to−hip ratio adjusted for BMI (adjusted for smoking behaviour) (25%)
	Stromal	Glaucoma (primary open−angle) (30.4%), Intraocular pressure (25.9%), Male−pattern baldness (13%)
	Heart	Resting heart rate (40%), Atrial fibrillation (37%), Atrial fibrillation (29.5%), Pulse pressure (25%), Diastolic blood pressure (25%)
	Muscle	Diastolic blood pressure (66.7%), Coronary artery disease (50%), Waist−to−hip ratio adjusted for BMI (adjusted for smoking behaviour) (31.2%), Triglycerides (26.1%), Intraocular pressure (21.1%)

Extended Data Fig. 11 | Tissue-tissue GWAS relationships. Principal and partner tissue enrichments. **a**, For each tree node label (rows), the number of GWAS traits (black *x*-axis, bottom) showing maximum enrichment in that tree node (dark bars, principal tissue) or any enrichment in that tree node (light bars, partner tissue), and the percentage of tissue-enriched traits for which the tissue shows the maximal enrichment (red *x*-axis, top) across 540 traits. **b**, Overlap in enriched GWAS traits between pairs of tissues with maximal enrichment in the trait (principal tissue, rows) and lower enrichment in the same trait (partner tissue, columns), using tree node labels. **c**, Top traits in significant interactions for selected tissue pairs (liver, endocrine, muscle, heart, adipose, PNS). For each pair of co-enriched tissue groups we reported the top 5 GWAS by their per cent of significant enrichments coming from either group.

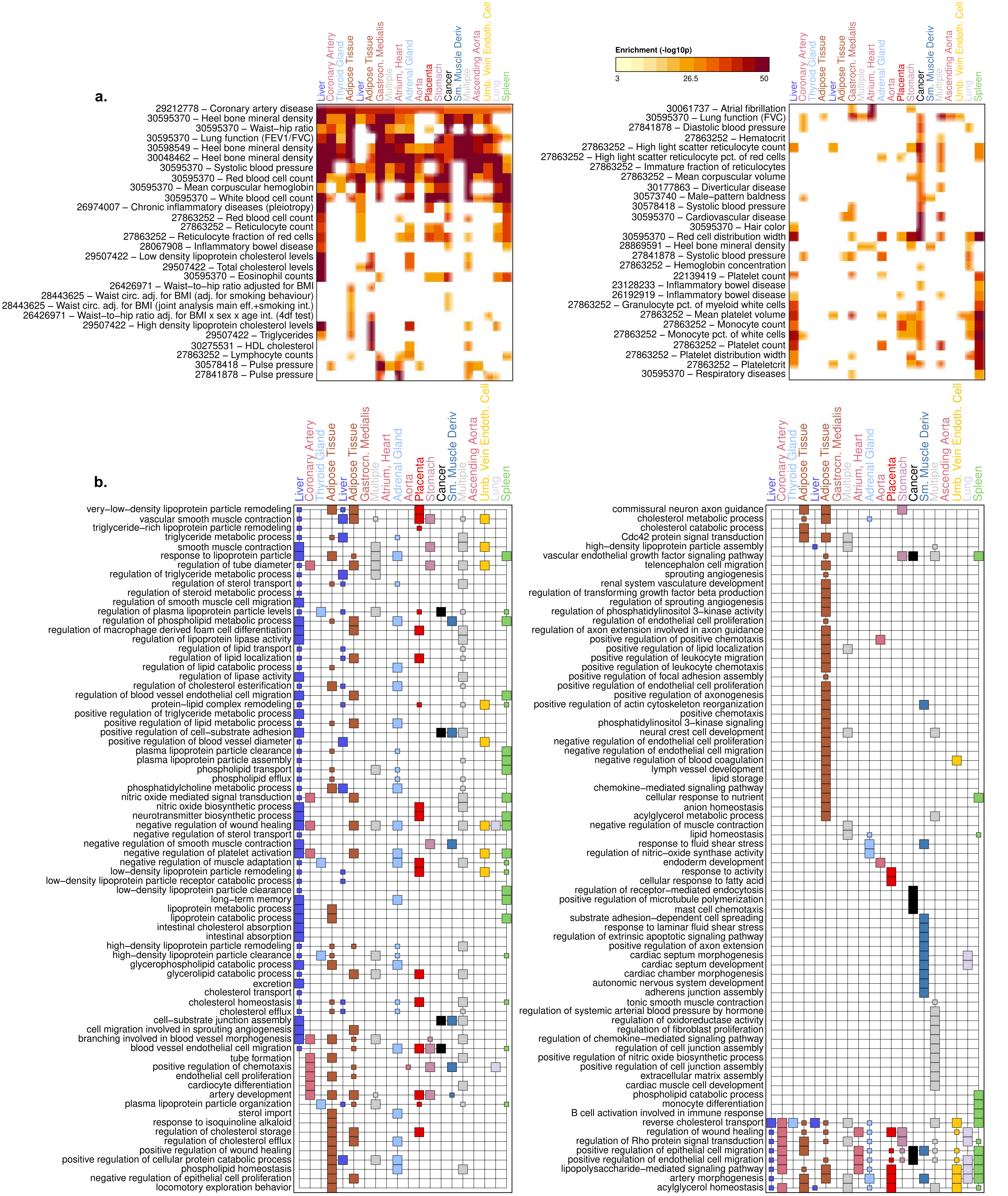

Extended Data Fig. 12 | Extended CAD investigation. a, Shared enrichments with CAD for 56 of 803 traits sharing at least two enrichments with the top 20 enriched nodes of CAD. Matrix is diagonalized according to maximal enrichment (nominal p-value, only enrichments passing FDR <0.1% are shown). **b**, Extended GO terms for CAD lead SNPs in enriched nodes. All GO terms with at least -$\log_{10}$q > 2enriched in less than 25% of nodes.

nature research

Corresponding author(s): Manolis Kellis

Last updated by author(s): Dec 7, 2020

Reporting Summary

Nature Research wishes to improve the reproducibility of the work that we publish. This form provides structure for consistency and transparency in reporting. For further information on Nature Research policies, see our Editorial Policies and the Editorial Policy Checklist.

Statistics

For all statistical analyses, confirm that the following items are present in the figure legend, table legend, main text, or Methods section.

n/a	Confirmed	
☒	☐	The exact sample size (*n*) for each experimental group/condition, given as a discrete number and unit of measurement
☒	☐	A statement on whether measurements were taken from distinct samples or whether the same sample was measured repeatedly
☐	☒	The statistical test(s) used AND whether they are one- or two-sided<br>*Only common tests should be described solely by name; describe more complex techniques in the Methods section.*
☐	☒	A description of all covariates tested
☐	☒	A description of any assumptions or corrections, such as tests of normality and adjustment for multiple comparisons
☐	☒	A full description of the statistical parameters including central tendency (e.g. means) or other basic estimates (e.g. regression coefficient) AND variation (e.g. standard deviation) or associated estimates of uncertainty (e.g. confidence intervals)
☐	☒	For null hypothesis testing, the test statistic (e.g. *F*, *t*, *r*) with confidence intervals, effect sizes, degrees of freedom and *P* value noted<br>*Give P values as exact values whenever suitable.*
☒	☐	For Bayesian analysis, information on the choice of priors and Markov chain Monte Carlo settings
☒	☐	For hierarchical and complex designs, identification of the appropriate level for tests and full reporting of outcomes
☐	☒	Estimates of effect sizes (e.g. Cohen's *d*, Pearson's *r*), indicating how they were calculated

Our web collection on statistics for biologists contains articles on many of the points above.

Software and code

Policy information about availability of computer code

Data collection	N/A
Data analysis	ChromImpute can be found at http://www.biolchem.ucla.edu/labs/ernst/ChromImpute/. Analysis was performed with R (3.5 and 3.6) and Python 3.7. Analysis code is available from http://compbio.mit.edu/epimap/ .

For manuscripts utilizing custom algorithms or software that are central to the research but not yet described in published literature, software must be made available to editors and reviewers. We strongly encourage code deposition in a community repository (e.g. GitHub). See the Nature Research guidelines for submitting code & software for further information.

Data

Policy information about availability of data

All manuscripts must include a data availability statement. This statement should provide the following information, where applicable:

- Accession codes, unique identifiers, or web links for publicly available datasets
- A list of figures that have associated raw data
- A description of any restrictions on data availability

We provide all imputed and processed observed tracks along with ChromHMM annotations and tracksets for the 859 imputed and final 833 QCed samples at https://epigenome.wustl.edu/epimap . All other processed and intermediate datasets, including metadata (Supplementary Tables S1 and S2), flagged samples, annotations, DHS locations, enhancer and promoter definitions, enhancer and promoter matrix, modules, and matched RNA-seq data can be found at http://compbio.mit.edu/epimap . We also provide an interactive data and analysis browser at https://cboix.shinyapps.io/epimap_vis/ including biosample and track exploration,

creation of custom track hubs, modules and motifs enrichments, and per-GWAS dissections for each of the GWAS and their lead SNPs (Supplementary Fig. 26)

Field-specific reporting

Please select the one below that is the best fit for your research. If you are not sure, read the appropriate sections before making your selection.

☒ Life sciences ☐ Behavioural & social sciences ☐ Ecological, evolutionary & environmental sciences

For a reference copy of the document with all sections, see nature.com/documents/nr-reporting-summary-flat.pdf

Life sciences study design

All studies must disclose on these points even when the disclosure is negative.

Sample size	N/A
Data exclusions	N/A
Replication	N/A
Randomization	N/A
Blinding	N/A

Reporting for specific materials, systems and methods

We require information from authors about some types of materials, experimental systems and methods used in many studies. Here, indicate whether each material, system or method listed is relevant to your study. If you are not sure if a list item applies to your research, read the appropriate section before selecting a response.

Materials & experimental systems

n/a	Involved in the study	
☒	☐	Antibodies
☒	☐	Eukaryotic cell lines
☒	☐	Palaeontology and archaeology
☒	☐	Animals and other organisms
☒	☐	Human research participants
☒	☐	Clinical data
☒	☐	Dual use research of concern

Methods

n/a	Involved in the study	
☒	☐	ChIP-seq
☒	☐	Flow cytometry
☒	☐	MRI-based neuroimaging
