# Supplementary material for: Author Correction: Regulatory genomic circuitry of human disease loci by integrative epigenomics
Source: Nature. 2025 Jun 20;643(8071):E11. doi: 10.1038/s41586-025-09134-4 (PMC12240796; doi:10.1038/s41586-025-09134-4)
Supplement: Supplementary file 2 — Original, uncorrected Supplementary Figures and Supplementary Data [file 41586_2025_9134_MOESM2_ESM.pdf]

Original, uncorrected Supplementary Figures

	Group	BSSID	Extended Info
1	Adipose	BSS00038	ADIPOCYTE
2	Adipose	BSS00043	ADIPOSE TISSUE
3	Adipose	BSS01671	ADIPOSE TISSUE
4	Adipose	BSS01665	ADIPOSE TISSUE
5	Adipose	BSS01666	ADIPOSE TISSUE
6	Adipose	BSS01668	ADIPOSE TISSUE
7	Adipose	BSS01669	ADIPOSE TISSUE
8	Adipose	BSS01667	ADIPOSE TISSUE
9	Adipose	BSS01394	OMENTAL FAT PAD
10	Adipose	BSS01393	OMENTAL FAT PAD
11	Blood & T-cell	BSS00188	CD4 T CELL
12	Blood & T-cell	BSS00185	CD4 T CELL
13	Blood & T-cell	BSS00189	CD4 T CELL
14	Blood & T-cell	BSS00190	CD4 T CELL
15	Blood & T-cell	BSS00183	CD4 T CELL
16	Blood & T-cell	BSS00186	CD4 T CELL
17	Blood & T-cell	BSS00191	CD4 T CELL
18	Blood & T-cell	BSS00192	CD4 T CELL
19	Blood & T-cell	BSS00274	CD4 T CELL
20	Blood & T-cell	BSS00195	CD8 T CELL
21	Blood & T-cell	BSS00196	CD8 T CELL
22	Blood & T-cell	BSS00198	CD8 T CELL
23	Blood & T-cell	BSS00200	CD8 T CELL
24	Blood & T-cell	BSS00193	CD8 T CELL
25	Blood & T-cell	BSS00194	CD8 T CELL
26	Blood & T-cell	BSS00197	CD8 T CELL
27	Blood & T-cell	BSS01420	MONONUCLEAR CELL
28	Blood & T-cell	BSS01421	MONONUCLEAR CELL
29	Blood & T-cell	BSS01279	MONONUCLEAR CELL
30	Blood & T-cell	BSS01419	MONONUCLEAR CELL
31	Blood & T-cell	BSS01423	MONONUCLEAR CELL
32	Blood & T-cell	BSS01424	MONONUCLEAR CELL
33	Blood & T-cell	BSS01347	NAIVE T CELL
34	Blood & T-cell	BSS01348	NAIVE T CELL
35	Blood & T-cell	BSS01346	NAIVE T CELL
36	Blood & T-cell	BSS01688	T CELL
37	Blood & T-cell	BSS01687	T CELL
38	Blood & T-cell	BSS01689	T CELL
39	Blood & T-cell	BSS01684	T CELL
40	Blood & T-cell	BSS01691	T1 CELL
41	Blood & T-cell	BSS01692	T1 CELL
42	Blood & T-cell	BSS01690	T1 CELL
43	Blood & T-cell	BSS01693	T17 CELL
44	Blood & T-cell	BSS01694	T17 CELL
45	Blood & T-cell	BSS01695	T17 CELL
46	Blood & T-cell	BSS01697	T2 CELL
47	Blood & T-cell	BSS01698	T2 CELL
48	Blood & T-cell	BSS01696	T2 CELL
49	Blood & T-cell	BSS01478	TREG CELL
50	Blood & T-cell	BSS01479	TREG CELL
51	Blood & T-cell	BSS01480	TREG CELL
52	Bone	BSS00084	BONE ARM
53	Bone	BSS00330	BONE FEMUR
54	Bone	BSS01154	BONE LEG
55	Bone	BSS00705	BONE MARROW STROMA
56	Bone	BSS01397	OSTEOBLAST
57	Brain	BSS00071	AMMONS HORN
58	Brain	BSS00077	ANGULAR GYRUS
59	Brain	BSS00078	ANGULAR GYRUS
60	Brain	BSS00089	ASTROCYTE
61	Brain	BSS00090	ASTROCYTE CEREBELLUM
62	Brain	BSS00091	ASTROCYTE HIPPOCAMPUS
63	Brain	BSS00092	ASTROCYTE SPINAL CORD
64	Brain	BSS00135	BRAIN
65	Brain	BSS00136	BRAIN
66	Brain	BSS00129	BRAIN
67	Brain	BSS00130	BRAIN
68	Brain	BSS00131	BRAIN
69	Brain	BSS00133	BRAIN
70	Brain	BSS00138	BRAIN
71	Brain	BSS00139	BRAIN
72	Brain	BSS00140	BRAIN
73	Brain	BSS00142	BRAIN
74	Brain	BSS00126	BRAIN
75	Brain	BSS00127	BRAIN
76	Brain	BSS00125	BRAIN
77	Brain	BSS00132	BRAIN
78	Brain	BSS00134	BRAIN
79	Brain	BSS00141	BRAIN
80	Brain	BSS00174	CAUDATE NUCLEUS
81	Brain	BSS00175	CAUDATE NUCLEUS
82	Brain	BSS00173	CAUDATE NUCLEUS
83	Brain	BSS00201	CEREBELLAR CORTEX
84	Brain	BSS00205	CEREBELLUM
85	Brain	BSS00207	CEREBELLUM

	Group	BSSID	Extended Info
288	Digestive	BSS01654	STOMACH
289	Digestive	BSS01651	STOMACH
290	Digestive	BSS01639	STOMACH
291	Digestive	BSS01284	STOMACH MUCOSA
292	Digestive	BSS01848	TRANSVERSE COLON
293	Digestive	BSS01851	TRANSVERSE COLON
294	Digestive	BSS01849	TRANSVERSE COLON
295	Digestive	BSS01850	TRANSVERSE COLON
296	Endocrine	BSS00282	ENDOCRINE PANCREAS
297	Endocrine	BSS00283	ENDOCRINE PANCREAS
298	Endocrine	BSS00284	ENDOCRINE PANCREAS
299	Endocrine	BSS00281	ENDOCRINE PANCREAS
300	Endocrine	BSS01403	OVARY
301	Endocrine	BSS01401	OVARY
302	Endocrine	BSS01402	OVARY
303	Endocrine	BSS01399	OVARY
304	Endocrine	BSS01719	TESTIS
305	Endocrine	BSS01718	TESTIS
306	Endocrine	BSS01715	TESTIS
307	Endocrine	BSS00052	ADRENAL GLAND
308	Endocrine	BSS00050	ADRENAL GLAND
309	Endocrine	BSS00051	ADRENAL GLAND
310	Endocrine	BSS00059	ADRENAL GLAND
311	Endocrine	BSS00057	ADRENAL GLAND
312	Endocrine	BSS00058	ADRENAL GLAND
313	Endocrine	BSS00045	ADRENAL GLAND
314	Endocrine	BSS00046	ADRENAL GLAND
315	Endocrine	BSS00047	ADRENAL GLAND
316	Endocrine	BSS00048	ADRENAL GLAND
317	Endocrine	BSS00054	ADRENAL GLAND
318	Endocrine	BSS00060	ADRENAL GLAND
319	Endocrine	BSS00055	ADRENAL GLAND
320	Endocrine	BSS00056	ADRENAL GLAND
321	Endocrine	BSS01831	THYROID GLAND
322	Endocrine	BSS01834	THYROID GLAND
323	Endocrine	BSS01832	THYROID GLAND
324	Endocrine	BSS01835	THYROID GLAND
325	Endothelial	BSS00143	BRAIN MICROVASCULAR ENDOTHELIAL CELL
326	Endothelial	BSS00387	GLOMERULUS ENDOTHELIAL CELL
327	Endothelial	BSS01077	KIDNEY CAPILLARY ENDOTHELIAL CELL
328	Endothelial	BSS01206	LUNG MICROVASCULAR ENDOTHELIAL CELL
329	Endothelial	BSS01465	PULMONARY ARTERY ENDOTHELIAL CELL
330	Endothelial	BSS00298	UMBILICAL VEIN ENDOTHELIAL CELL
331	Endothelial	BSS00296	UMBILICAL VEIN ENDOTHELIAL CELL
332	Endothelial	BSS00258	DERMIS BLOOD VESSEL ENDOTHELIAL CELL
333	Endothelial	BSS00260	DERMIS BLOOD VESSEL ENDOTHELIAL CELL
334	Endothelial	BSS00262	DERMIS LYMPHATIC VESSEL ENDOTHELIAL CELL
335	Endothelial	BSS00264	DERMIS LYMPHATIC VESSEL ENDOTHELIAL CELL
336	Epithelial	BSS00704	BONE MARROW EPITHELIAL CELL
337	Epithelial	BSS00218	CHOROID PLEXUS EPITHELIAL CELL
338	Epithelial	BSS00223	COLON EPITHELIAL CELL
339	Epithelial	BSS00307	ESOPHAGUS EPITHELIAL CELL
340	Epithelial	BSS00743	IRIS PIGMENT EPITHELIAL CELL
341	Epithelial	BSS01385	NON-PIGMENTED CILIARY EPITHELIAL CELL
342	Epithelial	BSS01092	GLOMERULUS EPITHELIAL CELL
343	Epithelial	BSS00389	GLOMERULUS VISCERAL EPITHELIAL CELL
344	Epithelial	BSS01080	KIDNEY EPITHELIAL CELL
345	Epithelial	BSS00310	PROXIMAL TUBULE EPITHELIAL CELL
346	Epithelial	BSS00701	PROXIMAL TUBULE EPITHELIAL CELL
347	Epithelial	BSS01491	RENAL CORTICAL EPITHELIAL CELL
348	Epithelial	BSS01505	RETINAL EPITHELIAL CELL
349	Epithelial	BSS01103	TUBULE CELL
350	Epithelial	BSS01102	TUBULE CELL
351	Epithelial	BSS00153	BRONCHIAL EPITHELIAL CELL
352	Epithelial	BSS00150	BRONCHIAL EPITHELIAL CELL
353	Epithelial	BSS00703	PANCREATIC DUCT EPITHELIAL CELL
354	Epithelial	BSS00075	AMNION EPITHELIAL CELL
355	Epithelial	BSS00308	PROSTATE EPITHELIAL CELL
356	Epithelial	BSS00309	PROSTATE EPITHELIAL CELL
357	Epithelial	BSS01539	PROSTATE EPITHELIAL CELL
358	Epithelial	BSS01538	PROSTATE EPITHELIAL CELL
359	Epithelial	BSS01217	BREAST EPITHELIAL CELL
360	Epithelial	BSS01224	BREAST EPITHELIAL CELL
361	Epithelial	BSS01225	BREAST EPITHELIAL CELL
362	Epithelial	BSS00356	FORESKIN KERATINOCYTE
363	Epithelial	BSS00357	FORESKIN KERATINOCYTE
364	Epithelial	BSS00358	FORESKIN KERATINOCYTE
365	Epithelial	BSS00359	FORESKIN KERATINOCYTE
366	Epithelial	BSS00360	FORESKIN KERATINOCYTE
367	Epithelial	BSS00362	FORESKIN KERATINOCYTE
368	Epithelial	BSS00363	FORESKIN KERATINOCYTE
369	Epithelial	BSS00364	FORESKIN KERATINOCYTE
370	Epithelial	BSS00365	FORESKIN KERATINOCYTE
371	Epithelial	BSS00366	FORESKIN KERATINOCYTE
372	Epithelial	BSS00355	FORESKIN KERATINOCYTE

	Group	BSSID	Extended Info
575	Kidney	BSS01533	RENAL PELVIS
576	Kidney	BSS01497	RENAL PELVIS
577	Kidney	BSS01498	RENAL PELVIS
578	Kidney	BSS01153	RENAL PELVIS
579	Kidney	BSS01534	RENAL PELVIS
580	Kidney	BSS01499	RENAL PELVIS
581	Liver	BSS00553	HEPATIC STELLATE CELL
582	Liver	BSS00554	HEPATOCYTE
583	Liver	BSS00511	LIVER
584	Liver	BSS01164	LIVER
585	Liver	BSS01170	LIVER
586	Liver	BSS01169	LIVER
587	Liver	BSS01159	LIVER
588	Liver	BSS01168	LIVER
589	Liver	BSS01519	LIVER
590	Liver	BSS01158	LIVER
591	Lung	BSS01195	LUNG
592	Lung	BSS01142	LUNG
593	Lung	BSS01525	LUNG
594	Lung	BSS01143	LUNG
595	Lung	BSS01526	LUNG
596	Lung	BSS01137	LUNG
597	Lung	BSS01520	LUNG
598	Lung	BSS01138	LUNG
599	Lung	BSS01521	LUNG
600	Lung	BSS01139	LUNG
601	Lung	BSS01192	LUNG
602	Lung	BSS01522	LUNG
603	Lung	BSS01140	LUNG
604	Lung	BSS01523	LUNG
605	Lung	BSS01141	LUNG
606	Lung	BSS01524	LUNG
607	Lung	BSS01205	LUNG
608	Lung	BSS01147	LUNG
609	Lung	BSS01529	LUNG
610	Lung	BSS01148	LUNG
611	Lung	BSS01149	LUNG
612	Lung	BSS01530	LUNG
613	Lung	BSS01202	LUNG
614	Lung	BSS01144	LUNG
615	Lung	BSS01527	LUNG
616	Lung	BSS01203	LUNG
617	Lung	BSS01145	LUNG
618	Lung	BSS01146	LUNG
619	Lung	BSS01528	LUNG
620	Lung	BSS01204	LUNG
621	Lung	BSS01189	LUNG
622	Lung	BSS01188	LUNG
623	Lung	BSS01187	LUNG
624	Lung	BSS01198	LUNG
625	Lung	BSS01186	LUNG
626	Lung	BSS01869	LUNG
627	Lung	BSS01196	LUNG
628	Lung	BSS01197	LUNG
629	Lung	BSS01871	LUNG
630	Lung	BSS01201	LUNG
631	Lung	BSS01190	LUNG
632	Lung	BSS01870	LUNG
633	Lung	BSS01193	LUNG
634	Lymphoblastoid	BSS00403	LYMPHOBLASTOID CELL LINE
635	Lymphoblastoid	BSS00462	LYMPHOBLASTOID CELL LINE
636	Lymphoblastoid	BSS00456	LYMPHOBLASTOID CELL LINE
637	Lymphoblastoid	BSS00457	LYMPHOBLASTOID CELL LINE
638	Lymphoblastoid	BSS00438	LYMPHOBLASTOID CELL LINE
639	Lymphoblastoid	BSS00473	LYMPHOBLASTOID CELL LINE
640	Lymphoblastoid	BSS00471	LYMPHOBLASTOID CELL LINE
641	Lymphoblastoid	BSS00474	LYMPHOBLASTOID CELL LINE
642	Lymphoblastoid	BSS00404	LYMPHOBLASTOID CELL LINE
643	Lymphoblastoid	BSS00405	LYMPHOBLASTOID CELL LINE
644	Lymphoblastoid	BSS00472	LYMPHOBLASTOID CELL LINE
645	Lymphoblastoid	BSS00428	LYMPHOBLASTOID CELL LINE
646	Lymphoblastoid	BSS00454	LYMPHOBLASTOID CELL LINE
647	Lymphoblastoid	BSS00427	LYMPHOBLASTOID CELL LINE
648	Lymphoblastoid	BSS00452	LYMPHOBLASTOID CELL LINE
649	Lymphoblastoid	BSS00395	LYMPHOBLASTOID CELL LINE
650	Lymphoblastoid	BSS00439	LYMPHOBLASTOID CELL LINE
651	Mesench	BSS00039	ADIPOCYTE FROM MSC
652	Mesench	BSS00250	AMNIOTIC FLUID FROM MSC
653	Mesench	BSS00279	EMBRYONIC FACIAL PROMINENCE
654	Mesench	BSS01260	MESENCHYMAL STEM CELL
655	Muscle	BSS01293	ARM MUSCLE
656	Muscle	BSS01294	ARM MUSCLE
657	Muscle	BSS01290	ARM MUSCLE
658	Muscle	BSS00352	ARM MUSCLE
659	Muscle	BSS01291	ARM MUSCLE

Figure S1: Sample list with tissue group, unique identifier, and short name for 859 observed/imputed samples (full metadata in Table S1, page 1 of 4).

	Group	BSSID	Extended Info
86	Brain	BSS00206	CEREBELLUM
87	Brain	BSS00219	CINGULATE GYRUS
88	Brain	BSS00220	CINGULATE GYRUS
89	Brain	BSS00369	FRONTAL CORTEX
90	Brain	BSS00371	FRONTAL CORTEX
91	Brain	BSS00385	GERMINAL MATRIX
92	Brain	BSS00386	GLOBUS PALLIDUS
93	Brain	BSS01125	HIPPOCAMPUS
94	Brain	BSS01126	HIPPOCAMPUS
95	Brain	BSS01124	HIPPOCAMPUS
96	Brain	BSS00729	INFERIOR PARIETAL CORTEX
97	Brain	BSS01250	MEDULLA OBLONGATA
98	Brain	BSS01270	MIDBRAIN
99	Brain	BSS01271	MIDDLE FRONTAL AREA
100	Brain	BSS01272	MIDDLE FRONTAL AREA
101	Brain	BSS01273	MIDDLE FRONTAL GYRUS
102	Brain	BSS01388	OCCIPITAL LOBE
103	Brain	BSS01451	PONS
104	Brain	BSS01452	POSTERIOR CINGULATE CORTEX
105	Brain	BSS01469	PUTAMEN
106	Brain	BSS01675	SUBSTANTIA NIGRA
107	Brain	BSS01676	SUBSTANTIA NIGRA
108	Brain	BSS01677	SUPERIOR TEMPORAL GYRUS
109	Brain	BSS01714	TEMPORAL LOBE
110	Brain	BSS01712	TEMPORAL LOBE
111	Cancer	BSS01105	ACUTE LYMPHOBLASTIC LEUKEMIA
112	Cancer	BSS00267	ACUTE LYMPHOBLASTIC LEUKEMIA
113	Cancer	BSS01178	ACUTE LYMPHOBLASTIC LEUKEMIA
114	Cancer	BSS01267	OSTEOSARCOMA
115	Cancer	BSS01550	OSTEOSARCOMA
116	Cancer	BSS00246	DESMOPLASTIC MEDULLOBLASTOMA
117	Cancer	BSS01208	GLIOBLASTOMA
118	Cancer	BSS00004	GLIOBLASTOMA
119	Cancer	BSS00482	GLIOBLASTOMA
120	Cancer	BSS01251	MEDULLOBLASTOMA
121	Cancer	BSS01554	NEUROBLASTOMA
122	Cancer	BSS01558	NEUROBLASTOMA
123	Cancer	BSS00102	NEUROBLASTOMA
124	Cancer	BSS01571	NEUROBLASTOMA
125	Cancer	BSS01562	NEUROBLASTOMA
126	Cancer	BSS01559	NEUROEPITHELIOMA
127	Cancer	BSS00481	NEUROGLIOMA
128	Cancer	BSS01535	COLON CARCINOMA
129	Cancer	BSS01682	COLORECTAL ADENOCARCINOMA
130	Cancer	BSS00708	COLORECTAL ADENOCARCINOMA
131	Cancer	BSS01179	COLORECTAL ADENOCARCINOMA
132	Cancer	BSS00159	COLORECTAL ADENOCARCINOMA
133	Cancer	BSS00492	COLORECTAL ADENOCARCINOMA
134	Cancer	BSS01412	PARATHYROID ADENOMA
135	Cancer	BSS01411	PARATHYROID ADENOMA
136	Cancer	BSS01386	TESTICULAR EMBRYONAL CARCINOMA
137	Cancer	BSS01536	MELANOMA
138	Cancer	BSS01551	MELANOMA
139	Cancer	BSS00222	MELANOMA
140	Cancer	BSS01365	MYELOMA
141	Cancer	BSS01890	EYE RETINOBLASTOMA
142	Cancer	BSS00702	ACUTE PROMYELOCYTIC LEUKEMIA
143	Cancer	BSS01356	ACUTE PROMYELOCYTIC LEUKEMIA
144	Cancer	BSS01391	B CELL LYMPHOMA
145	Cancer	BSS01390	B CELL LYMPHOMA
146	Cancer	BSS01065	B CELL LYMPHOMA
147	Cancer	BSS00268	B CELL LYMPHOMA
148	Cancer	BSS01664	B CELL LYMPHOMA
149	Cancer	BSS01389	B CELL LYMPHOMA
150	Cancer	BSS01350	BURKITT LYMPHOMA
151	Cancer	BSS01351	BURKITT LYMPHOMA
152	Cancer	BSS00491	HAPLOID MYELOGENOUS LEUKEMIA
153	Cancer	BSS01038	MYELOGENOUS LEUKEMIA
154	Cancer	BSS01039	MYELOGENOUS LEUKEMIA
155	Cancer	BSS01056	MYELOGENOUS LEUKEMIA
156	Cancer	BSS01057	MYELOGENOUS LEUKEMIA
157	Cancer	BSS01059	MYELOGENOUS LEUKEMIA
158	Cancer	BSS00221	MYELOGENOUS LEUKEMIA
159	Cancer	BSS01066	MYELOGENOUS LEUKEMIA
160	Cancer	BSS00762	MYELOGENOUS LEUKEMIA
161	Cancer	BSS01104	MYELOMA
162	Cancer	BSS01274	MYELOMA
163	Cancer	BSS01537	PLASMA CELL MYELOMA
164	Cancer	BSS00160	KIDNEY CLEAR CELL CARCINOMA
165	Cancer	BSS00372	KIDNEY RHABOID TUMOR
166	Cancer	BSS00037	RENAL CELL ADENOCARCINOMA
167	Cancer	BSS01474	RENAL CELL ADENOCARCINOMA
168	Cancer	BSS01481	RENAL CELL CARCINOMA
169	Cancer	BSS00718	HEPATOCELLULAR CARCINOMA

	Group	BSSID	Extended Info
373	Epithelial	BSS00361	FORESKIN KERATINOCYTE
374	Epithelial	BSS00367	FORESKIN KERATINOCYTE
375	Epithelial	BSS00354	FORESKIN KERATINOCYTE
376	Epithelial	BSS01071	KERATINOCYTE
377	Epithelial	BSS01068	KERATINOCYTE
378	Epithelial	BSS01209	MAMMARY EPITHELIAL CELL
379	Epithelial	BSS01211	MAMMARY EPITHELIAL CELL
380	Epithelial	BSS01213	MAMMARY EPITHELIAL CELL
381	Epithelial	BSS01185	MAMMARY LUMINAL EPITHELIAL CELL
382	Epithelial	BSS01340	MAMMARY MYOEPITHELIAL CELL
383	Epithelial	BSS01341	MAMMARY MYOEPITHELIAL CELL
384	Epithelial	BSS01181	SKIN LEG
385	Epithelial	BSS01182	SKIN LEG
386	Epithelial	BSS01587	SKIN OF BODY
387	ES-deriv	BSS00112	BIPOLAR NEURON DERIV
388	ES-deriv	BSS01366	NEURAL DERIV
389	ES-deriv	BSS00272	NEURAL PROGENITOR DERIV
390	ES-deriv	BSS01372	NEURAL PROGENITOR DERIV
391	ES-deriv	BSS01370	NEURAL PROGENITOR DERIV
392	ES-deriv	BSS01371	NEURAL PROGENITOR DERIV
393	ES-deriv	BSS01375	NEURON DERIV
394	ES-deriv	BSS00169	CARDIAC MESODERM DERIV
395	ES-deriv	BSS00171	CARDIAC MUSCLE DERIV
396	ES-deriv	BSS00556	HEPATOCYTE DERIV
397	ES-deriv	BSS01261	MESENCHYMAL STEM DERIV
398	ES-deriv	BSS01857	TROPHOBLAST DERIV
399	ES-deriv	BSS01612	SMOOTH MUSCLE DERIV
400	ES-deriv	BSS00273	ECTODERMAL DERIV
401	ES-deriv	BSS00285	ENDODERMAL CELL
402	ES-deriv	BSS00287	ENDODERMAL DERIV
403	ES-deriv	BSS01263	MESENDODERM DERIV
404	ES-deriv	BSS01264	MESODERMAL DERIV
405	ESC	BSS00277	ESC
406	ESC	BSS00315	ESC
407	ESC	BSS01866	ESC
408	ESC	BSS00483	ESC
409	ESC	BSS00715	ESC
410	ESC	BSS00716	ESC
411	ESC	BSS00717	ESC
412	ESC	BSS00484	ESC
413	ESC	BSS00478	ESC
414	Eye	BSS00329	EYE
415	Eye	BSS00328	EYE
416	Eye	BSS01504	EYE RETINA
417	Eye	BSS01503	EYE RETINA
418	Eye	BSS01502	EYE RETINA
419	Heart	BSS00079	AORTA
420	Heart	BSS00080	AORTA
421	Heart	BSS00088	ASCENDING AORTA
422	Heart	BSS00087	ASCENDING AORTA
423	Heart	BSS00242	CORONARY ARTERY
424	Heart	BSS00243	CORONARY ARTERY
425	Heart	BSS00505	HEART
426	Heart	BSS00498	HEART
427	Heart	BSS00499	HEART
428	Heart	BSS00500	HEART
429	Heart	BSS00502	HEART
430	Heart	BSS00503	HEART
431	Heart	BSS00501	HEART
432	Heart	BSS00516	HEART
433	Heart	BSS00522	HEART
434	Heart	BSS00518	HEART
435	Heart	BSS00519	HEART
436	Heart	BSS00514	HEART
437	Heart	BSS00520	HEART
438	Heart	BSS00495	HEART
439	Heart	BSS00496	HEART
440	Heart	BSS00494	HEART
441	Heart	BSS00521	HEART
442	Heart	BSS00517	HEART
443	Heart	BSS00493	HEART
444	Heart	BSS01127	HEART LEFT ATRIUM
445	Heart	BSS00509	HEART LEFT VENTRICLE
446	Heart	BSS00508	HEART LEFT VENTRICLE
447	Heart	BSS00506	HEART LEFT VENTRICLE
448	Heart	BSS00513	HEART LEFT VENTRICLE
449	Heart	BSS00512	HEART LEFT VENTRICLE
450	Heart	BSS00507	HEART LEFT VENTRICLE
451	Heart	BSS01506	HEART RIGHT ATRIUM
452	Heart	BSS01508	HEART RIGHT ATRIUM
453	Heart	BSS01507	HEART RIGHT ATRIUM
454	Heart	BSS00523	HEART RIGHT VENTRICLE
455	Heart	BSS00524	HEART RIGHT VENTRICLE
456	Heart	BSS00525	HEART RIGHT VENTRICLE

	Group	BSSID	Extended Info
660	Muscle	BSS01292	ARM MUSCLE
661	Muscle	BSS01303	ARM MUSCLE
662	Muscle	BSS01304	ARM MUSCLE
663	Muscle	BSS01295	ARM MUSCLE
664	Muscle	BSS01296	ARM MUSCLE
665	Muscle	BSS01297	ARM MUSCLE
666	Muscle	BSS01298	ARM MUSCLE
667	Muscle	BSS01299	ARM MUSCLE
668	Muscle	BSS01300	ARM MUSCLE
669	Muscle	BSS01301	ARM MUSCLE
670	Muscle	BSS01289	ARM MUSCLE
671	Muscle	BSS01308	BACK MUSCLE
672	Muscle	BSS01309	BACK MUSCLE
673	Muscle	BSS01305	BACK MUSCLE
674	Muscle	BSS01306	BACK MUSCLE
675	Muscle	BSS01307	BACK MUSCLE
676	Muscle	BSS01315	BACK MUSCLE
677	Muscle	BSS01316	BACK MUSCLE
678	Muscle	BSS01317	BACK MUSCLE
679	Muscle	BSS01310	BACK MUSCLE
680	Muscle	BSS01311	BACK MUSCLE
681	Muscle	BSS01312	BACK MUSCLE
682	Muscle	BSS01313	BACK MUSCLE
683	Muscle	BSS01314	BACK MUSCLE
684	Muscle	BSS00170	CARDIAC MYOCYTE
685	Muscle	BSS00376	GASTROCNEMIUS MEDIALIS
686	Muscle	BSS00378	GASTROCNEMIUS MEDIALIS
687	Muscle	BSS00377	GASTROCNEMIUS MEDIALIS
688	Muscle	BSS00379	GASTROCNEMIUS MEDIALIS
689	Muscle	BSS01322	LEG MUSCLE
690	Muscle	BSS01318	LEG MUSCLE
691	Muscle	BSS01320	LEG MUSCLE
692	Muscle	BSS01321	LEG MUSCLE
693	Muscle	BSS01329	LEG MUSCLE
694	Muscle	BSS01330	LEG MUSCLE
695	Muscle	BSS01323	LEG MUSCLE
696	Muscle	BSS01324	LEG MUSCLE
697	Muscle	BSS01325	LEG MUSCLE
698	Muscle	BSS01327	LEG MUSCLE
699	Muscle	BSS00700	LEG MUSCLE
700	Muscle	BSS01328	LEG MUSCLE
701	Muscle	BSS01319	LEG MUSCLE
702	Muscle	BSS01460	PSOAS MUSCLE
703	Muscle	BSS01461	PSOAS MUSCLE
704	Muscle	BSS01462	PSOAS MUSCLE
705	Muscle	BSS01463	PSOAS MUSCLE
706	Muscle	BSS01581	SKELETAL MUSCLE
707	Muscle	BSS01577	SKELETAL MUSCLE
708	Muscle	BSS01578	SKELETAL MUSCLE
709	Muscle	BSS01572	SKELETAL MUSCLE CELL
710	Muscle	BSS01845	TONGUE
711	Muscle	BSS01846	TONGUE
712	Muscle	BSS01331	TRUNK MUSCLE
713	Muscle	BSS01333	TRUNK MUSCLE
714	Muscle	BSS01334	TRUNK MUSCLE
715	Muscle	BSS01332	TRUNK MUSCLE
716	Myosat	BSS01338	MYOCYTE
717	Myosat	BSS01344	MYOTUBE
718	Myosat	BSS01155	SKELETAL MUSCLE MYOBLAST
719	Myosat	BSS01573	SKELETAL MUSCLE MYOBLAST
720	Myosat	BSS01574	SKELETAL MUSCLE MYOBLAST
721	Myosat	BSS01576	SKELETAL MUSCLE SATELLITE CELL
722	Neurosph	BSS01378	NEUROSPHERE
723	Neurosph	BSS01379	NEUROSPHERE
724	Neurosph	BSS01377	NEUROSPHERE
725	Neurosph	BSS01392	OLFACTORY NEUROSPHERE
726	Other	BSS00148	BREAST EPITHELIUM
727	Other	BSS00145	BREAST EPITHELIUM
728	Other	BSS00146	BREAST EPITHELIUM
729	Other	BSS00304	EPIDERMAL MELANOCYTE
730	Other	BSS00368	FORESKIN MELANOCYTE
731	Other	BSS01156	LIMB EMBRYO
732	Other	BSS01157	LIMB EMBRYO
733	Other	BSS01216	MAMMARY STEM CELL
734	Pancreas	BSS00121	BODY OF PANCREAS
735	Pancreas	BSS00122	BODY OF PANCREAS
736	Pancreas	BSS00123	BODY OF PANCREAS
737	Pancreas	BSS00124	BODY OF PANCREAS
738	Pancreas	BSS00758	ISLET PRECURSOR CELL
739	Pancreas	BSS01406	PANCREAS
740	Pancreas	BSS01407	PANCREAS
741	Placenta & EEM	BSS00074	AMNION
742	Placenta & EEM	BSS00076	AMNION STEM CELL
743	Placenta & EEM	BSS00209	CHORION

Figure S1: (continued, 2 of 4)

	Group	BSSID	Extended Info
170	Cancer	BSS00719	HEPATOCELLULAR CARCINOMA
171	Cancer	BSS00558	HEPATOCELLULAR CARCINOMA
172	Cancer	BSS01360	LARGE CELL LUNG CANCER
173	Cancer	BSS01415	LUNG ADENOCARCINOMA
174	Cancer	BSS00017	LUNG EPITHELIAL CARCINOMA
175	Cancer	BSS00019	LUNG EPITHELIAL CARCINOMA
176	Cancer	BSS00021	LUNG EPITHELIAL CARCINOMA
177	Cancer	BSS00022	LUNG EPITHELIAL CARCINOMA
178	Cancer	BSS00027	LUNG EPITHELIAL CARCINOMA
179	Cancer	BSS00016	LUNG EPITHELIAL CARCINOMA
180	Cancer	BSS00020	LUNG EPITHELIAL CARCINOMA
181	Cancer	BSS00023	LUNG EPITHELIAL CARCINOMA
182	Cancer	BSS00024	LUNG EPITHELIAL CARCINOMA
183	Cancer	BSS00026	LUNG EPITHELIAL CARCINOMA
184	Cancer	BSS00028	LUNG EPITHELIAL CARCINOMA
185	Cancer	BSS00029	LUNG EPITHELIAL CARCINOMA
186	Cancer	BSS00018	LUNG EPITHELIAL CARCINOMA
187	Cancer	BSS00025	LUNG EPITHELIAL CARCINOMA
188	Cancer	BSS00030	LUNG EPITHELIAL CARCINOMA
189	Cancer	BSS00007	LUNG EPITHELIAL CARCINOMA
190	Cancer	BSS00013	LUNG EPITHELIAL CARCINOMA
191	Cancer	BSS00015	LUNG EPITHELIAL CARCINOMA
192	Cancer	BSS01359	SQUAMOUS CELL CARCINOMA
193	Cancer	BSS00035	MUSCLE EWING SARCOMA
194	Cancer	BSS01549	RHABDOMYOSARCOMA
195	Cancer	BSS00036	ADENOID CYSTIC CARCINOMA
196	Cancer	BSS01240	MAMMARY GLAND ADENOCARCINOMA
197	Cancer	BSS01243	MAMMARY GLAND ADENOCARCINOMA
198	Cancer	BSS01244	MAMMARY GLAND ADENOCARCINOMA
199	Cancer	BSS01235	MAMMARY GLAND ADENOCARCINOMA
200	Cancer	BSS01226	MAMMARY GLAND ADENOCARCINOMA
201	Cancer	BSS01699	MAMMARY GLAND DUCTAL CARCINOMA
202	Cancer	BSS01705	MAMMARY GLAND DUCTAL CARCINOMA
203	Cancer	BSS00003	PANCREAS ADENOCARCINOMA
204	Cancer	BSS01405	PANCREAS DUCT EPITHELIAL CARCINOMA
205	Cancer	BSS00541	CERVIX ADENOCARCINOMA
206	Cancer	BSS00531	CERVIX ADENOCARCINOMA
207	Cancer	BSS00529	CERVIX ADENOCARCINOMA
208	Cancer	BSS00748	ENDOMETRIAL ADENOCARCINOMA
209	Cancer	BSS00756	ENDOMETRIAL ADENOCARCINOMA
210	Cancer	BSS00745	ENDOMETRIAL ADENOCARCINOMA
211	Cancer	BSS01174	PROSTATE ADENOCARCINOMA
212	Cancer	BSS01173	PROSTATE ADENOCARCINOMA
213	Cancer	BSS01414	PROSTATE ADENOCARCINOMA
214	Cancer	BSS00157	PROSTATE CANCER
215	Cancer	BSS01888	PROSTATE EPITHELIAL CARCINOMA
216	Cancer	BSS00001	PROSTATE EPITHELIAL CARCINOMA
217	Cancer	BSS00002	PROSTATE EPITHELIAL CARCINOMA
218	Cancer	BSS00709	FIBROSARCOMA
219	Digestive	BSS00227	COLON MUCOSA
220	Digestive	BSS00228	COLON MUCOSA
221	Digestive	BSS00271	DUODENUM MUCOSA
222	Digestive	BSS00270	DUODENUM MUCOSA
223	Digestive	BSS00316	ESOPHAGUS
224	Digestive	BSS00318	ESOPHAGUS
225	Digestive	BSS00323	ESOPHAGUS MUSCULARIS MUCOSA
226	Digestive	BSS00322	ESOPHAGUS MUSCULARIS MUCOSA
227	Digestive	BSS00321	ESOPHAGUS MUSCULARIS MUCOSA
228	Digestive	BSS00324	ESOPHAGUS SQUAMOUS EPITHELIUM
229	Digestive	BSS00326	ESOPHAGUS SQUAMOUS EPITHELIUM
230	Digestive	BSS00325	ESOPHAGUS SQUAMOUS EPITHELIUM
231	Digestive	BSS00380	GASTROESOPHAGEAL SPHINCTER
232	Digestive	BSS00381	GASTROESOPHAGEAL SPHINCTER
233	Digestive	BSS01116	LARGE INTESTINE
234	Digestive	BSS01117	LARGE INTESTINE
235	Digestive	BSS01109	LARGE INTESTINE
236	Digestive	BSS01110	LARGE INTESTINE
237	Digestive	BSS01111	LARGE INTESTINE
238	Digestive	BSS01112	LARGE INTESTINE
239	Digestive	BSS01113	LARGE INTESTINE
240	Digestive	BSS01114	LARGE INTESTINE
241	Digestive	BSS01122	LARGE INTESTINE
242	Digestive	BSS01118	LARGE INTESTINE
243	Digestive	BSS01120	LARGE INTESTINE
244	Digestive	BSS01121	LARGE INTESTINE
245	Digestive	BSS01119	LARGE INTESTINE
246	Digestive	BSS01427	PEYERS PATCH
247	Digestive	BSS01428	PEYERS PATCH
248	Digestive	BSS01426	PEYERS PATCH
249	Digestive	BSS01282	RECTUM MUCOSA
250	Digestive	BSS01283	RECTUM MUCOSA
251	Digestive	BSS01542	SIGMOID COLON
252	Digestive	BSS01546	SIGMOID COLON
253	Digestive	BSS01547	SIGMOID COLON
457	Heart	BSS01815	THORACIC AORTA
458	Heart	BSS01814	THORACIC AORTA
459	Heart	BSS01839	TIBIAL ARTERY
460	Heart	BSS01838	TIBIAL ARTERY
461	Heart	BSS01837	TIBIAL ARTERY
462	HSC & B-cell	BSS00097	B CELL
463	HSC & B-cell	BSS01345	B CELL
464	HSC & B-cell	BSS00098	B CELL
465	HSC & B-cell	BSS00093	B CELL
466	HSC & B-cell	BSS00096	B CELL
467	HSC & B-cell	BSS00100	B CELL
468	HSC & B-cell	BSS00101	B CELL
469	HSC & B-cell	BSS00095	B CELL
470	HSC & B-cell	BSS00179	CD14 MONOCYTE
471	HSC & B-cell	BSS00181	CD14 MONOCYTE
472	HSC & B-cell	BSS00180	CD14 MONOCYTE
473	HSC & B-cell	BSS00178	CD14 MONOCYTE
474	HSC & B-cell	BSS00182	CD1C MYELOID DENDRITIC CELL
475	HSC & B-cell	BSS00233	CD34 CMP
476	HSC & B-cell	BSS00230	CD34 CMP
477	HSC & B-cell	BSS00236	CD34 CMP
478	HSC & B-cell	BSS00238	CD34 CMP
479	HSC & B-cell	BSS00240	CD34 CMP
480	HSC & B-cell	BSS00241	CD34 CMP
481	HSC & B-cell	BSS00234	CD34 CMP
482	HSC & B-cell	BSS00235	CD34 CMP
483	HSC & B-cell	BSS00229	CD34 CMP
484	HSC & B-cell	BSS00237	CD34 CMP
485	HSC & B-cell	BSS00239	CD34 CMP
486	HSC & B-cell	BSS00231	CD34 CMP
487	HSC & B-cell	BSS00232	CD34 CMP
488	HSC & B-cell	BSS00384	GERMINAL CENTER
489	HSC & B-cell	BSS00760	LYMPHOCYTE
490	HSC & B-cell	BSS00544	MPP
491	HSC & B-cell	BSS00545	MPP
492	HSC & B-cell	BSS00546	MPP
493	HSC & B-cell	BSS00547	MPP
494	HSC & B-cell	BSS00548	MPP
495	HSC & B-cell	BSS00549	MPP
496	HSC & B-cell	BSS00550	MPP
497	HSC & B-cell	BSS00551	MPP
498	HSC & B-cell	BSS00552	MPP
499	HSC & B-cell	BSS00543	MPP
500	HSC & B-cell	BSS01381	NEUTROPHIL
501	HSC & B-cell	BSS01380	NEUTROPHIL
502	HSC & B-cell	BSS01353	NK CELL
503	HSC & B-cell	BSS01355	NK CELL
504	HSC & B-cell	BSS01354	NK CELL
505	iPSC	BSS00742	iPSC
506	iPSC	BSS00735	iPSC
507	iPSC	BSS00741	iPSC
508	iPSC	BSS00732	iPSC
509	iPSC	BSS00733	iPSC
510	iPSC	BSS00244	iPSC
511	iPSC	BSS01107	iPSC
512	iPSC	BSS01108	iPSC
513	iPSC	BSS00738	iPSC
514	iPSC	BSS00736	iPSC
515	iPSC	BSS00737	iPSC
516	iPSC	BSS00739	iPSC
517	iPSC	BSS00731	iPSC
518	iPSC	BSS00734	iPSC
519	iPSC	BSS00477	iPSC
520	Kidney	BSS01091	KIDNEY
521	Kidney	BSS01132	KIDNEY
522	Kidney	BSS01512	KIDNEY
523	Kidney	BSS01133	KIDNEY
524	Kidney	BSS01513	KIDNEY
525	Kidney	BSS01084	KIDNEY
526	Kidney	BSS01128	KIDNEY
527	Kidney	BSS01509	KIDNEY
528	Kidney	BSS01085	KIDNEY
529	Kidney	BSS01129	KIDNEY
530	Kidney	BSS01086	KIDNEY
531	Kidney	BSS01510	KIDNEY
532	Kidney	BSS01089	KIDNEY
533	Kidney	BSS01130	KIDNEY
534	Kidney	BSS01511	KIDNEY
535	Kidney	BSS01090	KIDNEY
536	Kidney	BSS01078	KIDNEY
537	Kidney	BSS01100	KIDNEY
538	Kidney	BSS01101	KIDNEY
539	Kidney	BSS01135	KIDNEY
540	Kidney	BSS01516	KIDNEY
744	Placenta & EEM	BSS00211	CHORION
745	Placenta & EEM	BSS00212	CHORION
746	Placenta & EEM	BSS00215	CHORIONIC VILLUS
747	Placenta & EEM	BSS00216	CHORIONIC VILLUS
748	Placenta & EEM	BSS00217	CHORIONIC VILLUS
749	Placenta & EEM	BSS00214	CHORIONIC VILLUS
750	Placenta & EEM	BSS01440	PLACENTA
751	Placenta & EEM	BSS01436	PLACENTA
752	Placenta & EEM	BSS01437	PLACENTA
753	Placenta & EEM	BSS01435	PLACENTA
754	Placenta & EEM	BSS01443	PLACENTA
755	Placenta & EEM	BSS01444	PLACENTA
756	Placenta & EEM	BSS01433	PLACENTA
757	Placenta & EEM	BSS01432	PLACENTA
758	Placenta & EEM	BSS01430	PLACENTA
759	Placenta & EEM	BSS01448	PLACENTA
760	Placenta & EEM	BSS01446	PLACENTA
761	Placenta & EEM	BSS01441	PLACENTA
762	Placenta & EEM	BSS01431	PLACENTA
763	Placenta & EEM	BSS01438	PLACENTA
764	Placenta & EEM	BSS00714	TROPHOBLAST
765	Placenta & EEM	BSS01856	TROPHOBLAST
766	Placenta & EEM	BSS01853	TROPHOBLAST
767	Placenta & EEM	BSS01855	TROPHOBLAST
768	Placenta & EEM	BSS01852	TROPHOBLAST
769	Placenta & EEM	BSS01859	TROPHOBLAST
770	Placenta & EEM	BSS01860	TROPHOBLAST
771	Placenta & EEM	BSS01867	UMBILICAL CORD
772	PNS	BSS01618	SPINAL CORD
773	PNS	BSS01619	SPINAL CORD
774	PNS	BSS01617	SPINAL CORD
775	PNS	BSS01621	SPINAL CORD
776	PNS	BSS01620	SPINAL CORD
777	PNS	BSS01614	SPINAL CORD
778	PNS	BSS01613	SPINAL CORD
779	PNS	BSS01842	TIBIAL NERVE
780	PNS	BSS01840	TIBIAL NERVE
781	PNS	BSS01841	TIBIAL NERVE
782	Reproductive	BSS01456	PROSTATE GLAND
783	Reproductive	BSS01457	PROSTATE GLAND
784	Reproductive	BSS01459	PROSTATE GLAND
785	Reproductive	BSS01884	UTERUS
786	Reproductive	BSS01886	VAGINA
787	Reproductive	BSS01887	VAGINA
788	Sm. Muscle	BSS01606	BRAIN VASCULATURE SMOOTH MUSCLE CELL
789	Sm. Muscle	BSS01285	COLON MUSCLE
790	Sm. Muscle	BSS01286	COLON MUSCLE
791	Sm. Muscle	BSS01288	DUODENUM MUSCLE
792	Sm. Muscle	BSS01287	DUODENUM MUSCLE
793	Sm. Muscle	BSS01475	RECTUM MUSCLE
794	Sm. Muscle	BSS01660	STOMACH MUSCLE
795	Sm. Muscle	BSS01659	STOMACH MUSCLE
796	Spleen	BSS01625	SPLEEN
797	Spleen	BSS01628	SPLEEN
798	Spleen	BSS01629	SPLEEN
799	Spleen	BSS01633	SPLEEN
800	Spleen	BSS01634	SPLEEN
801	Spleen	BSS01631	SPLEEN
802	Spleen	BSS01630	SPLEEN
803	Stromal	BSS01661	BONE MARROW STROMAL CELL
804	Stromal	BSS00144	PERICYTE
805	Stromal	BSS00349	CONJUNCTIVA FIBROBLAST
806	Stromal	BSS00347	AORTA FIBROBLAST
807	Stromal	BSS00168	CARDIAC FIBROBLAST
808	Stromal	BSS00166	CARDIAC FIBROBLAST
809	Stromal	BSS00167	CARDIAC FIBROBLAST
810	Stromal	BSS00064	LUNG FIBROBLAST
811	Stromal	BSS01891	LUNG FIBROBLAST
812	Stromal	BSS00342	LUNG FIBROBLAST
813	Stromal	BSS00339	LUNG FIBROBLAST
814	Stromal	BSS00062	LUNG FIBROBLAST
815	Stromal	BSS00341	LUNG FIBROBLAST
816	Stromal	BSS00720	LUNG FIBROBLAST
817	Stromal	BSS00345	PULMONARY ARTERY FIBROBLAST
818	Stromal	BSS00338	GINGIVAL FIBROBLAST
819	Stromal	BSS00067	GINGIVAL FIBROBLAST
820	Stromal	BSS00344	PERIDONTAL LIGAMENT FIBROBLAST
821	Stromal	BSS00350	VILLOUS MESENCHYME FIBROBLAST
822	Stromal	BSS00332	BREAST FIBROBLAST
823	Stromal	BSS00333	BREAST FIBROBLAST
824	Stromal	BSS00335	DERMIS FIBROBLAST
825	Stromal	BSS00334	DERMIS FIBROBLAST
826	Stromal	BSS00337	DERMIS FIBROBLAST
827	Stromal	BSS00697	FORESKIN FIBROBLAST

Figure S1: (continued, 3 of 4)

	Group	BSSID	Extended Info
254	Digestive	BSS01548	SIGMOID COLON
255	Digestive	BSS01545	SIGMOID COLON
256	Digestive	BSS01543	SIGMOID COLON
257	Digestive	BSS01595	SMALL INTESTINE
258	Digestive	BSS01596	SMALL INTESTINE
259	Digestive	BSS01590	SMALL INTESTINE
260	Digestive	BSS01591	SMALL INTESTINE
261	Digestive	BSS01592	SMALL INTESTINE
262	Digestive	BSS01593	SMALL INTESTINE
263	Digestive	BSS01594	SMALL INTESTINE
264	Digestive	BSS01603	SMALL INTESTINE
265	Digestive	BSS01604	SMALL INTESTINE
266	Digestive	BSS01600	SMALL INTESTINE
267	Digestive	BSS01602	SMALL INTESTINE
268	Digestive	BSS01597	SMALL INTESTINE
269	Digestive	BSS01599	SMALL INTESTINE
270	Digestive	BSS01588	SMALL INTESTINE
271	Digestive	BSS01601	SMALL INTESTINE
272	Digestive	BSS01637	STOMACH
273	Digestive	BSS01642	STOMACH
274	Digestive	BSS01643	STOMACH
275	Digestive	BSS01644	STOMACH
276	Digestive	BSS01646	STOMACH
277	Digestive	BSS01647	STOMACH
278	Digestive	BSS01641	STOMACH
279	Digestive	BSS01658	STOMACH
280	Digestive	BSS01655	STOMACH
281	Digestive	BSS01656	STOMACH
282	Digestive	BSS01657	STOMACH
283	Digestive	BSS01636	STOMACH
284	Digestive	BSS01638	STOMACH
285	Digestive	BSS01650	STOMACH
286	Digestive	BSS01653	STOMACH
287	Digestive	BSS01649	STOMACH

	Group	BSSID	Extended Info
541	Kidney	BSS01517	KIDNEY
542	Kidney	BSS01136	KIDNEY
543	Kidney	BSS01518	KIDNEY
544	Kidney	BSS01099	KIDNEY
545	Kidney	BSS01514	KIDNEY
546	Kidney	BSS01134	KIDNEY
547	Kidney	BSS01515	KIDNEY
548	Kidney	BSS01131	KIDNEY
549	Kidney	BSS01079	KIDNEY
550	Kidney	BSS01096	KIDNEY
551	Kidney	BSS01097	KIDNEY
552	Kidney	BSS01088	KIDNEY
553	Kidney	BSS00528	KIDNEY CELL
554	Kidney	BSS00526	KIDNEY CELL
555	Kidney	BSS01484	RENAL CORTEX INTERSTITIUM
556	Kidney	BSS01485	RENAL CORTEX INTERSTITIUM
557	Kidney	BSS01482	RENAL CORTEX INTERSTITIUM
558	Kidney	BSS01483	RENAL CORTEX INTERSTITIUM
559	Kidney	BSS01489	RENAL CORTEX INTERSTITIUM
560	Kidney	BSS01490	RENAL CORTEX INTERSTITIUM
561	Kidney	BSS01150	RENAL CORTEX INTERSTITIUM
562	Kidney	BSS01531	RENAL CORTEX INTERSTITIUM
563	Kidney	BSS01486	RENAL CORTEX INTERSTITIUM
564	Kidney	BSS01487	RENAL CORTEX INTERSTITIUM
565	Kidney	BSS01151	RENAL CORTEX INTERSTITIUM
566	Kidney	BSS01532	RENAL CORTEX INTERSTITIUM
567	Kidney	BSS01488	RENAL CORTEX INTERSTITIUM
568	Kidney	BSS01495	RENAL PELVIS
569	Kidney	BSS01496	RENAL PELVIS
570	Kidney	BSS01493	RENAL PELVIS
571	Kidney	BSS01494	RENAL PELVIS
572	Kidney	BSS01500	RENAL PELVIS
573	Kidney	BSS01501	RENAL PELVIS
574	Kidney	BSS01152	RENAL PELVIS

	Group	BSSID	Extended Info
828	Stromal	BSS00353	FORESKIN FIBROBLAST
829	Stromal	BSS00343	MAMMARY FIBROBLAST
830	Stromal	BSS00275	SKIN FIBROBLAST
831	Stromal	BSS00276	SKIN FIBROBLAST
832	Stromal	BSS00393	SKIN FIBROBLAST
833	Stromal	BSS00394	SKIN FIBROBLAST
834	Stromal	BSS00063	SKIN FIBROBLAST
835	Stromal	BSS00069	SKIN FIBROBLAST
836	Stromal	BSS00346	SKIN FIBROBLAST
837	Stromal	BSS01583	SKIN FIBROBLAST
838	Stromal	BSS00278	SKIN FIBROBLAST
839	Stromal	BSS00390	SKIN FIBROBLAST
840	Stromal	BSS00066	SKIN FIBROBLAST
841	Stromal	BSS00061	SKIN FIBROBLAST
842	Stromal	BSS00068	SKIN FIBROBLAST
843	Stromal	BSS00476	SKIN FIBROBLAST
844	Stromal	BSS00113	SKIN FIBROBLAST
845	Thymus	BSS01824	THYMUS
846	Thymus	BSS01819	THYMUS
847	Thymus	BSS01821	THYMUS
848	Thymus	BSS01823	THYMUS
849	Thymus	BSS01818	THYMUS
850	Thymus	BSS01826	THYMUS
851	Thymus	BSS01827	THYMUS
852	Thymus	BSS01828	THYMUS
853	Thymus	BSS01829	THYMUS
854	Thymus	BSS01820	THYMUS
855	Thymus	BSS01825	THYMUS
856	Urinary	BSS01878	URINARY BLADDER
857	Urinary	BSS01876	URINARY BLADDER
858	Urinary	BSS01879	UROTHELIUM CELL
859	Urinary	BSS01880	UROTHELIUM CELL

Figure S1: (continued, 4 of 4)

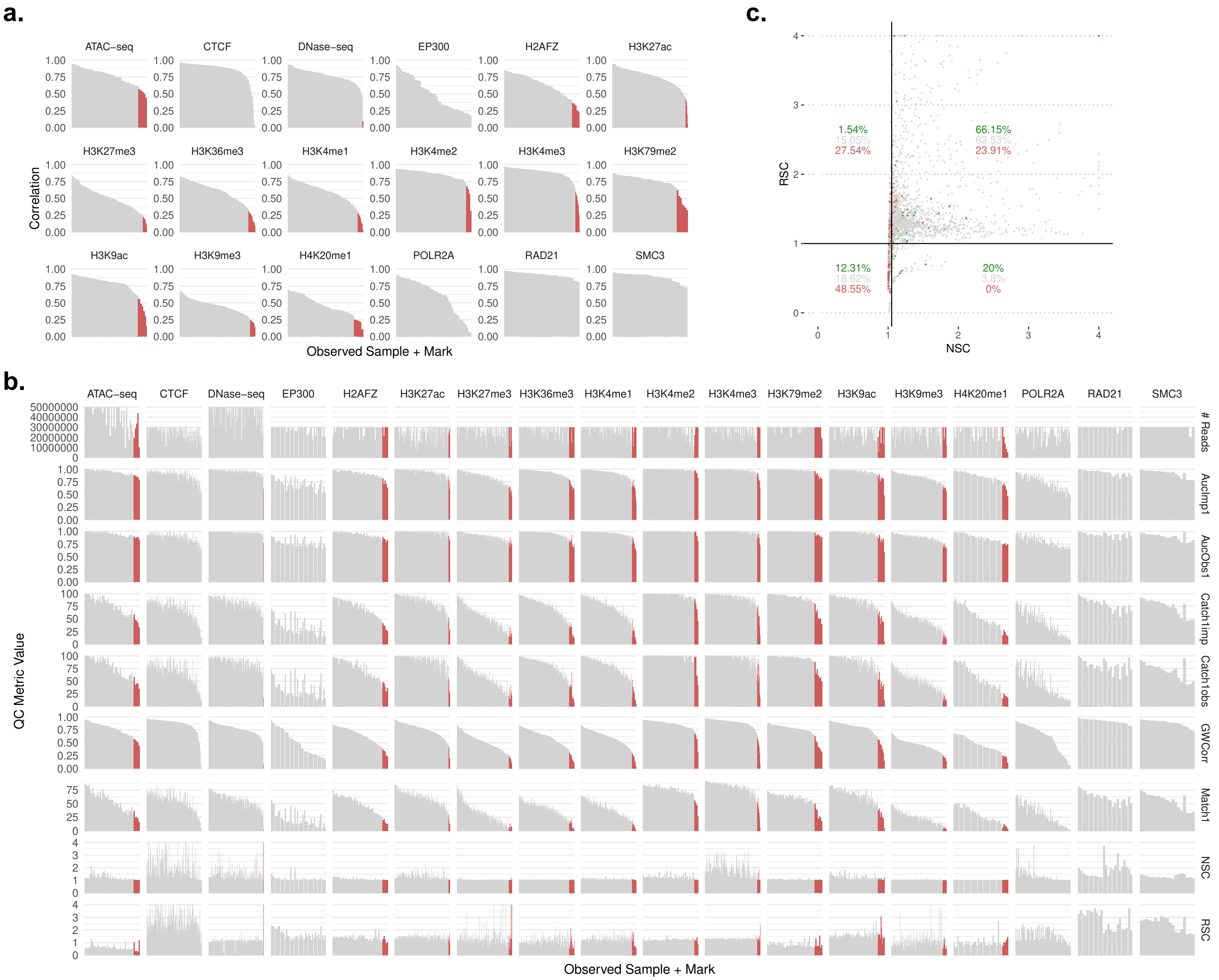

Figure S2: a. Track agreement between observed and imputed in the Tier 1 marks, where both are available, by genome wide correlation. Top (green) and bottom (red) tracks are labeled according to automated elbow discovery in the ranked list. **b.** All imputation metrics (from original ChromImpute paper: correlation, AUC, and peak recovery) and QC metrics (reads, NSC, RSC) across all 18 imputed Tier 1,2, and 3 assays, ordered according to correlation between imputed and observed within each assay. Bottom tracks for Tier 1 and 2 assays are labeled in red, as above. **c.** Imputation QC metrics reflect external ChIP-seq quality metrics (NSC and RSC). Normalized Strand Cross-correlation coefficient (NSC) against Relative Strand Cross-correlation coefficient (RSC) for observed tracks. Top (green) and bottom (red) tracks are labeled according to automated elbow discovery in imputed vs. observed correlation ranked list. Poor agreement tracks are strongly clustered in lower left quadrant of tracks failing QC. Critical values subdivide the plot for NSC (1.05) and RSC (1).

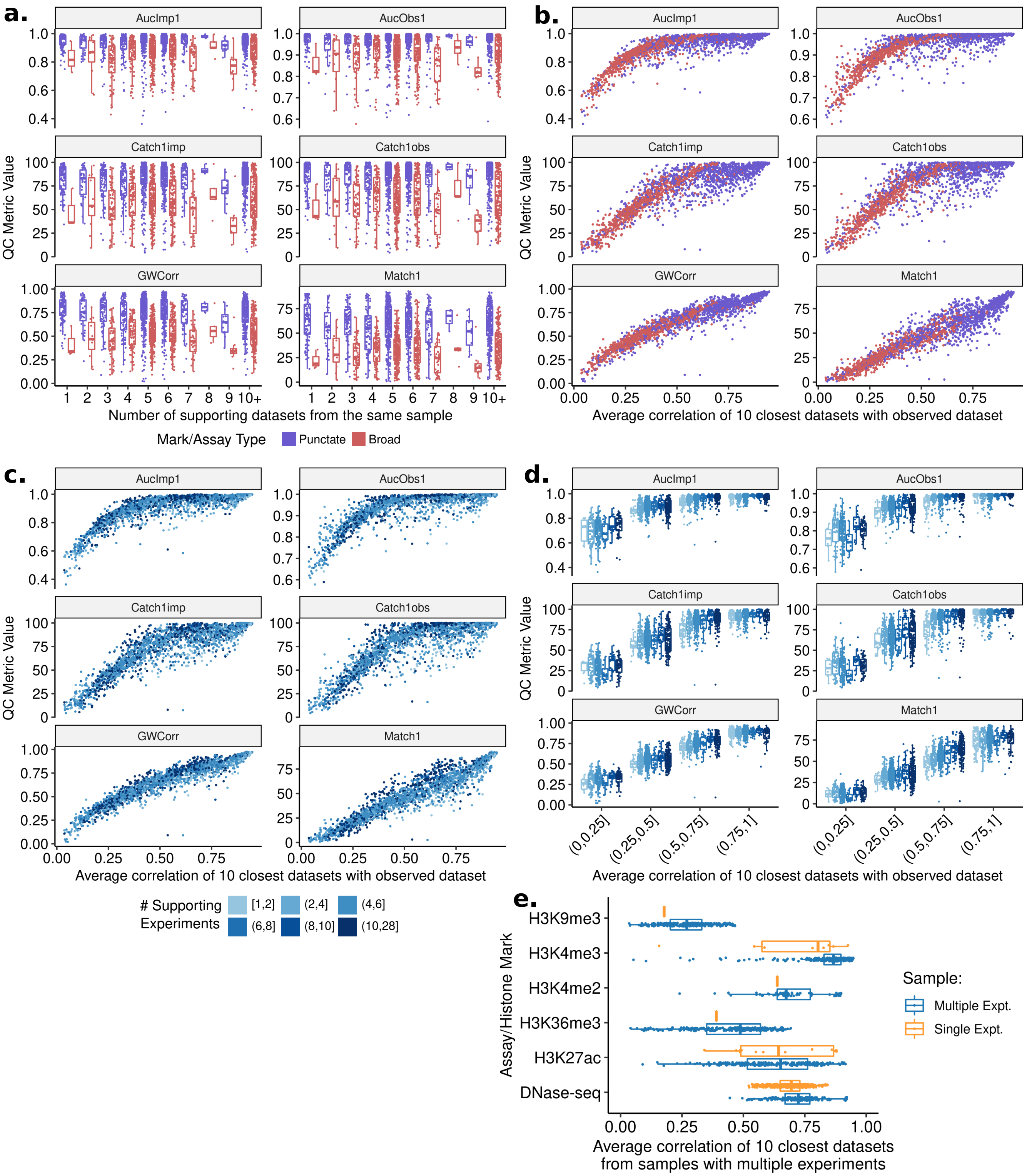

Figure S3: Imputation quality by level of sample-internal and sample-external support. a. Imputation QC metrics in punctate (blue) and broad (red) marks by number of other supporting datasets from the same sample (sample-internal support). **b.** Imputation QC metrics for punctate and broad marks against the average correlation of the 10 closest datasets with the observed dataset. (sample-external support). **c-d.** Metrics against continuous (**c**) and quartiles of (**d**) sample-external support, colored by level of sample-internal support, showing that imputation metrics are dominated by the level of sample-external support. **e.** Level of sample-external support from samples with multiple experiments for samples with only one experiment (orange) or samples with multiple experiments (blue), by available mark or assay. Almost all samples with only DNase-seq have strong cross-sample support from other samples with multiple experiments (average correlation of nearest samples above 50

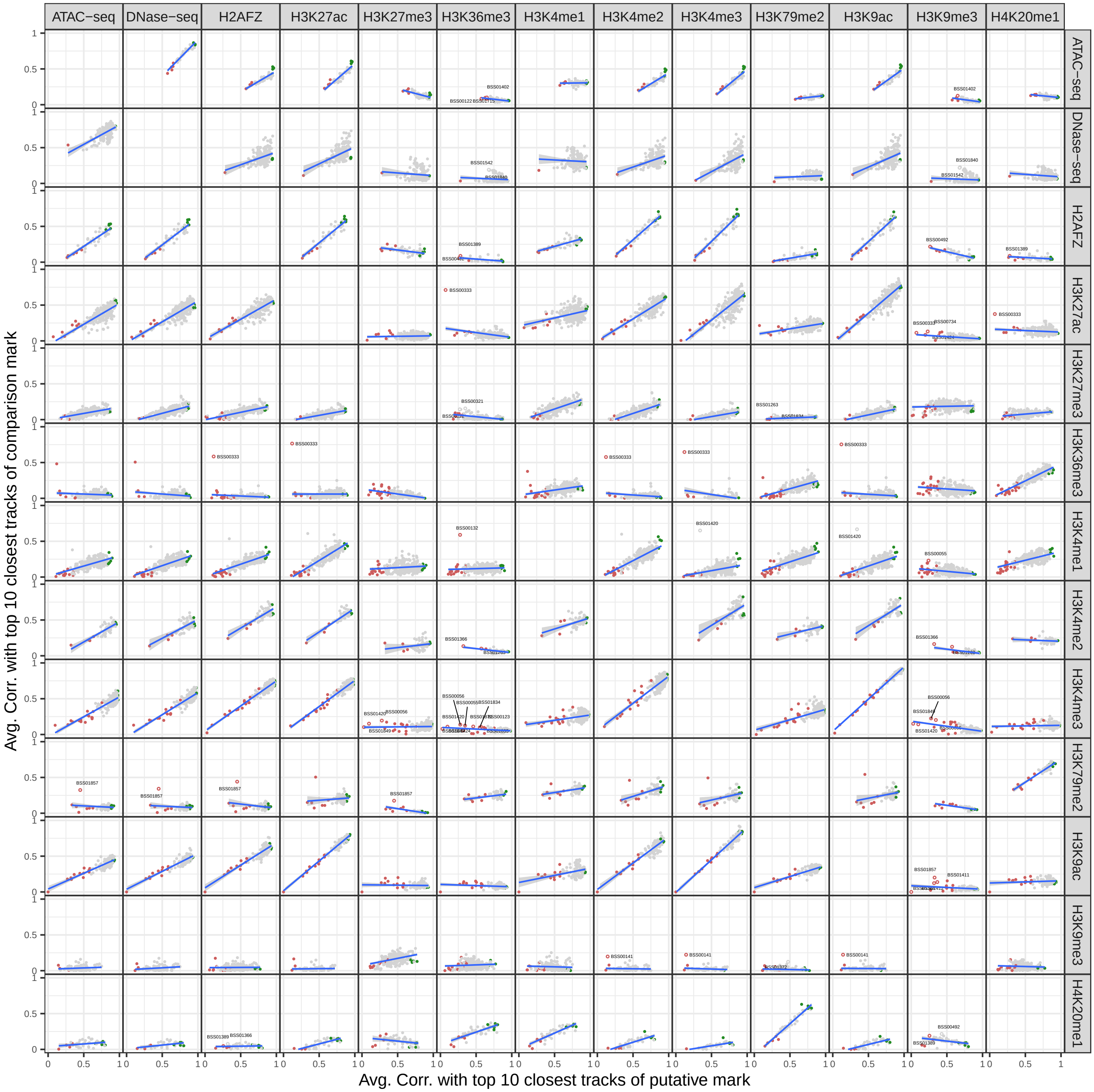

Figure S4: Imputed-observed agreement can be used to systematically flag antibody swaps. Each panel shows the average correlation with closest 10 tracks within the putative mark (each row) for each observed dataset against all other Tier 1 and 2 marks and assays. We used the overall mark-mark trend to flag outliers for visual inspection.

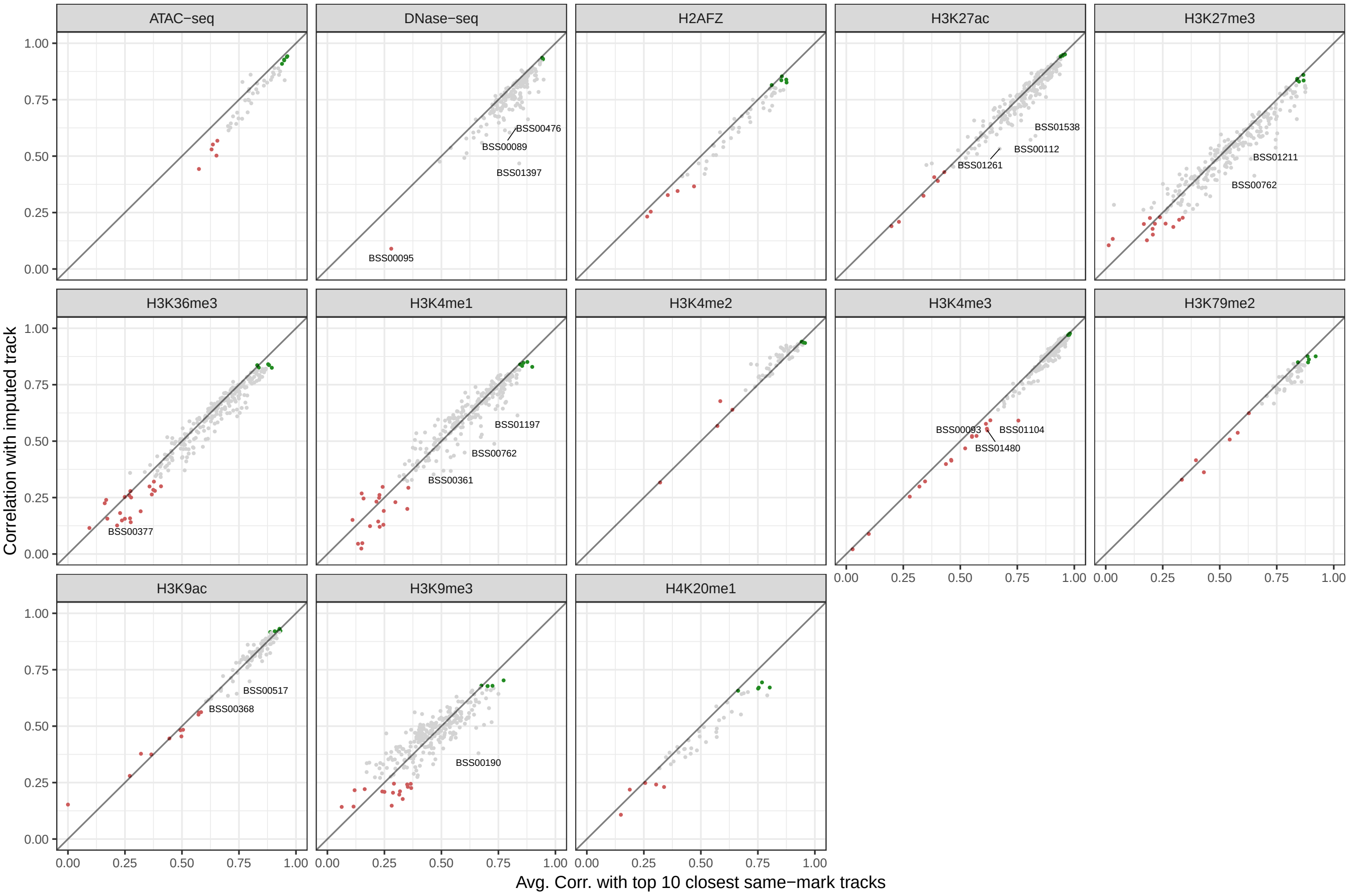

Figure S5: Imputed-observed agreement can be used to flag sample swaps. We compare the imputed-observed correlation to the average imputed-observed correlation within the top 10 closest samples for the putative mark and flagged outliers for visual inspection (after removing antibody swaps).

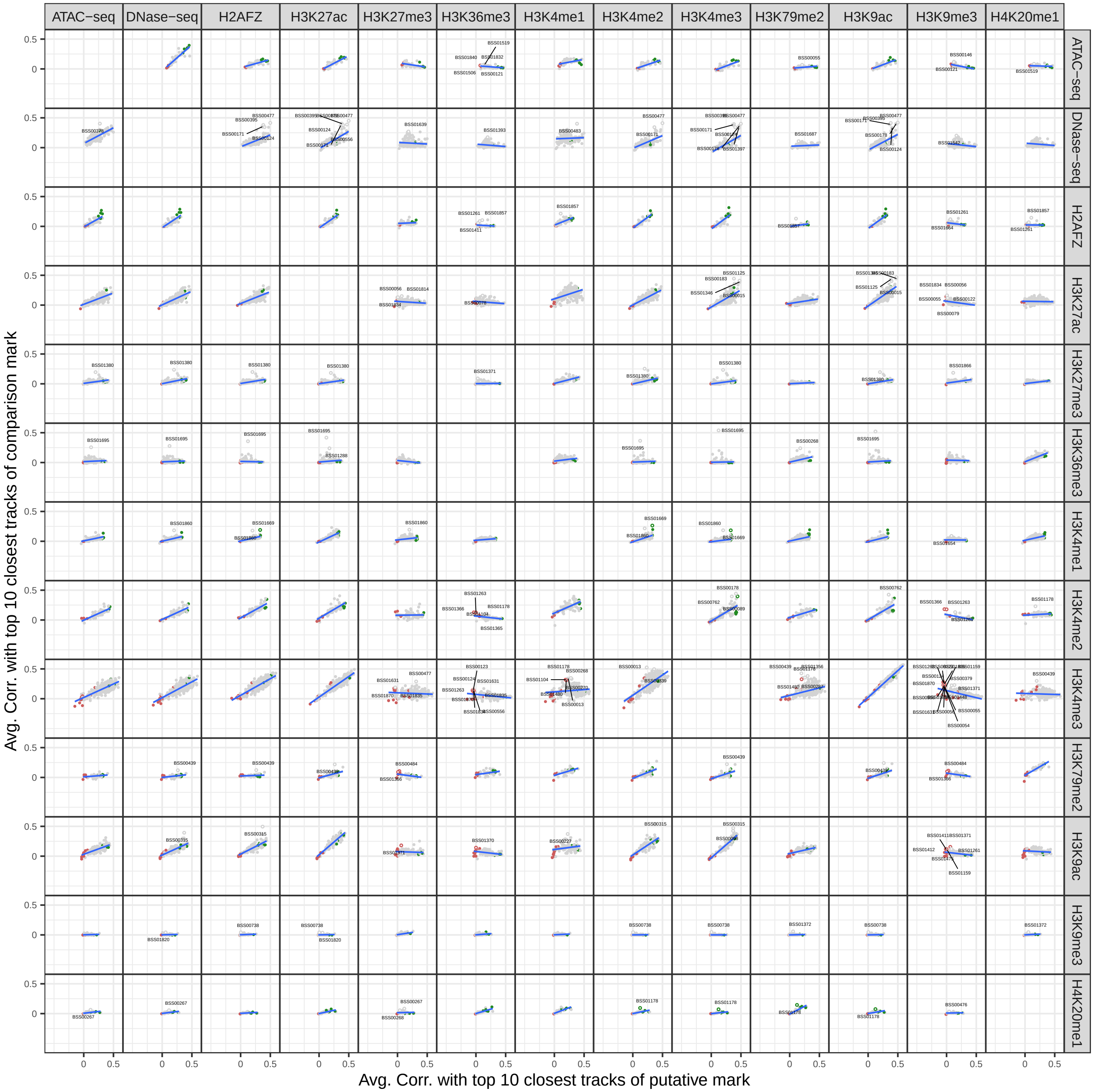

Figure S6: Imputed-observed agreement can be used to systematically flag secondary reactivities of antibodies. Each panel shows the average correlation of the difference between a pair of observed and imputed datasets with closest 10 tracks within the putative mark (each row) for each dataset against all other Tier 1 and 2 marks and assays. As with antibody swaps, we used the overall mark-mark trend to flag outliers for visual inspection.

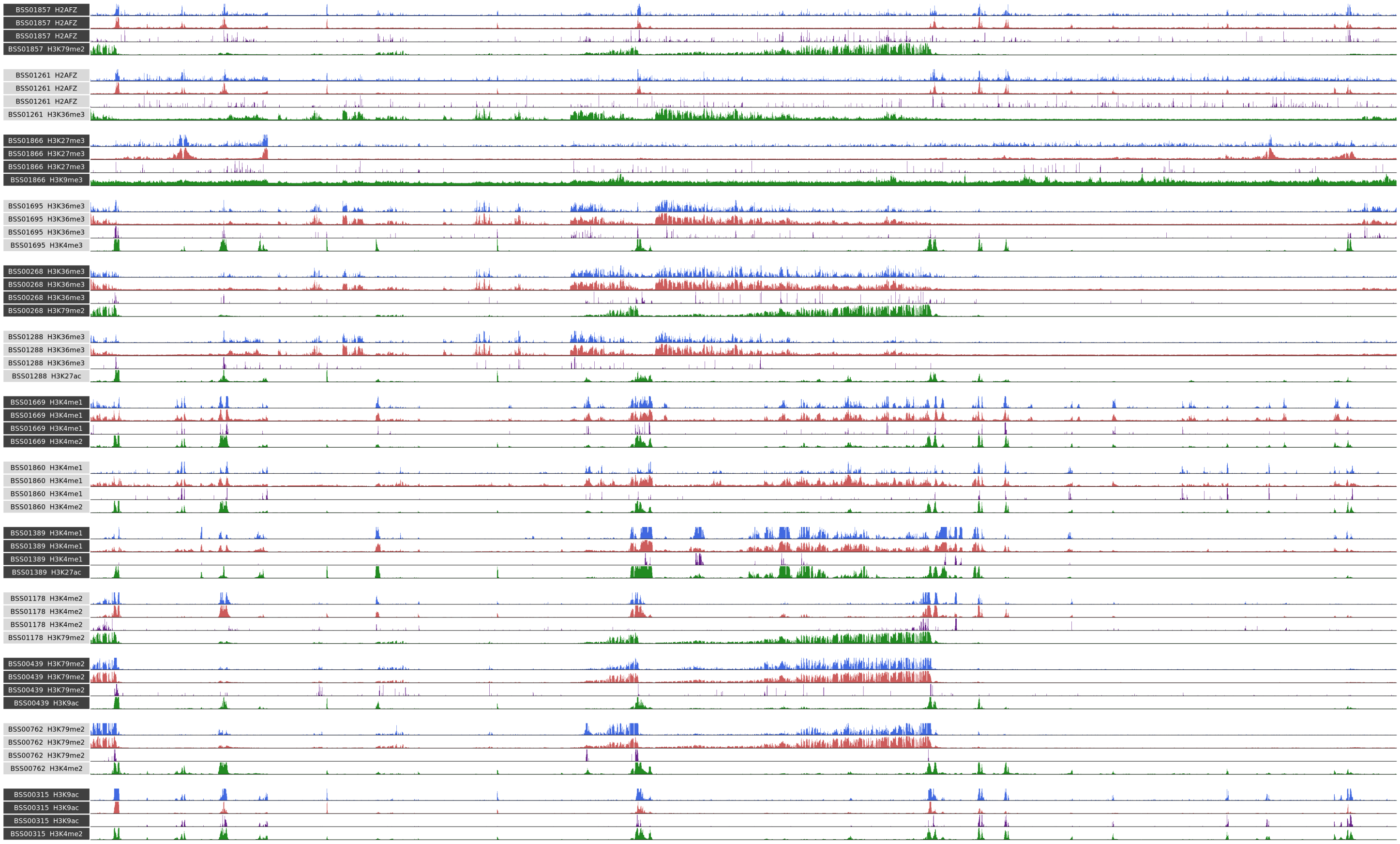

Figure S7: We can use imputation to identify antibodies with secondary reactivities. Track sets for 13 flagged samples highlight a disagreement from secondary reactivity (or sample swapping of one experiment out of multiple replicates), where the difference between observed and imputed best correlate with an external histone mark or assay. Each track set shows the Difference (purple) from Observed (blue) to Imputed (red) and the best Match (green) to the difference (a mark in the same sample) in chromosome 1 from 1.5Mb to 2Mb (chosen to show a range of diverse elements and marks).

	id	mark	(low quality tracks)					
1	BSS01365	H3K4me2	47	BSS01857	H3K9ac	93	BSS00734	H3K27ac
2	BSS01104	H3K4me2	48	BSS01832	H3K36me3	94	BSS01715	H3K9me3
3	BSS01365	H3K79me2	49	BSS01841	H3K36me3	95	BSS00325	H3K9me3
4	BSS00556	H3K4me3	50	BSS01424	H3K4me3	96	BSS01850	H3K27me3
5	BSS01104	H3K4me3	51	BSS00055	H3K4me1	97	BSS01407	H3K27me3
6	BSS01263	H3K4me3	52	BSS00087	H3K4me1	98	BSS00381	H3K4me1
7	BSS01815	ATAC-seq	53	BSS00080	H3K4me1	99	BSS00395	H3K27me3
8	BSS01263	H3K4me2	54	BSS00325	H3K36me3	100	BSS00381	H3K9me3
9	BSS01475	H3K9ac	55	BSS00556	H3K36me3	101	BSS00321	H3K4me1
10	BSS01196	H3K9ac	56	BSS01340	H3K9ac	102	BSS01424	H3K27ac
11	BSS00093	H3K4me3	57	BSS00284	H3K36me3	103	BSS00124	H3K36me3
12	BSS01506	ATAC-seq	58	BSS00055	H3K36me3	104	BSS01507	H3K27me3
13	BSS01667	H3K9ac	59	BSS00281	H3K4me1	105	BSS01715	H3K36me3
14	BSS01480	H3K4me3	60	BSS01543	H3K36me3	106	BSS01209	H3K27me3
15	BSS00484	H3K79me2	61	BSS01835	H3K4me1	107	BSS01832	H3K9me3
16	BSS01715	ATAC-seq	62	BSS01080	H3K36me3	108	BSS01850	H3K36me3
17	BSS00123	H3K4me3	63	BSS00056	H3K4me3	109	BSS01411	H3K36me3
18	BSS01834	H3K4me3	64	BSS01389	H2AFZ	110	BSS01370	H3K36me3
19	BSS01835	H3K4me3	65	BSS00493	H3K36me3	111	BSS01366	H3K9ac
20	BSS01366	H3K79me2	66	BSS01835	H3K36me3	112	BSS01870	H3K27me3
21	BSS01402	ATAC-seq	67	BSS00284	H3K4me1	113	BSS01834	H3K4me1
22	BSS01371	H3K9ac	68	BSS01389	H4K20me1	114	BSS01887	H3K36me3
23	BSS01370	H3K9ac	69	BSS00054	H3K4me1	115	BSS01831	H3K9me3
24	BSS00284	H3K4me3	70	BSS00122	H3K9me3	116	BSS01630	H3K4me1
25	BSS00197	H3K9ac	71	BSS00123	H3K9me3	117	BSS01459	H3K9me3
26	BSS01341	H3K9ac	72	BSS01850	H3K9me3	118	BSS01424	H3K9me3
27	BSS00122	ATAC-seq	73	BSS01213	H4K20me1	119	BSS00377	H3K36me3
28	BSS00055	H3K27ac	74	BSS00093	H3K36me3	120	BSS00159	H3K27me3
29	BSS01870	H3K4me3	75	BSS00132	H3K4me1	121	BSS01507	H3K4me1
30	BSS01412	H3K79me2	76	BSS00492	H2AFZ	122	BSS01837	H3K27me3
31	BSS00141	H3K4me3	77	BSS01876	H3K4me1	123	BSS01426	H3K36me3
32	BSS00074	H3K27ac	78	BSS01370	H3K9me3	124	BSS01837	H3K4me1
33	BSS00521	H3K4me3	79	BSS00439	H4K20me1	125	BSS01426	H3K4me1
34	BSS01857	H3K79me2	80	BSS00325	H3K4me1	126	BSS01459	H3K36me3
35	BSS01866	H3K27ac	81	BSS01178	H3K27me3	127	BSS00484	H4K20me1
36	BSS01412	H3K9ac	82	BSS00141	H3K9me3	128	BSS00531	H3K27me3
37	BSS01411	H3K9ac	83	BSS01849	H3K27me3	129	BSS00333	H3K36me3
38	BSS00478	H2AFZ	84	BSS01543	H3K9me3	130	BSS00093	H3K27me3
39	BSS00556	H3K79me2	85	BSS00284	H3K27me3	131	BSS01562	DNase-seq
40	BSS00558	H2AFZ	86	BSS00054	H3K36me3	132	BSS00095	DNase-seq
41	BSS01411	H3K79me2	87	BSS00493	H3K9me3	133	BSS01420	H3K4me3
42	BSS00477	H2AFZ	88	BSS00341	H4K20me1	134	BSS00333	H3K27ac
43	BSS00281	H3K27ac	89	BSS00325	H3K27me3	135	BSS01849	H3K4me1
44	BSS00055	H3K4me3	90	BSS00074	H3K9me3	136	BSS01519	H3K4me1
45	BSS00378	H3K36me3	91	BSS01426	H3K9me3	137	BSS01543	H3K4me1
46	BSS01366	H3K4me2	92	BSS00483	H3K9me3	138	BSS01849	H3K4me3

	id	mark	potential.abswap
1	BSS00333	H3K36me3	H3K27ac
2	BSS00333	H3K27ac	H3K36me3
3	BSS01857	H3K9ac	H3K79me2
4	BSS01857	H3K79me2	H3K9ac
5	BSS00141	H3K9me3	H3K9ac
6	BSS00132	H3K4me1	H3K36me3
7	BSS01420	H3K4me1	H3K4me3

	id	mark	potential.sampswap
1	BSS00089	DNase-seq	BSS00339
2	BSS00095	DNase-seq	BSS01452
3	BSS00476	DNase-seq	BSS01397
4	BSS01397	DNase-seq	BSS00334
5	BSS00112	H3K27ac	BSS01366
6	BSS01261	H3K27ac	BSS00387
7	BSS01538	H3K27ac	BSS00703
8	BSS00762	H3K27me3	BSS00547
9	BSS01211	H3K27me3	BSS01224
10	BSS00377	H3K36me3	BSS01715
11	BSS00361	H3K4me1	BSS00365
12	BSS00762	H3K4me1	BSS01038
13	BSS01197	H3K4me1	BSS01144
14	BSS00093	H3K4me3	BSS00702
15	BSS01104	H3K4me3	BSS01365
16	BSS01480	H3K4me3	BSS00702
17	BSS00368	H3K9ac	BSS00207
18	BSS00517	H3K9ac	BSS00502
19	BSS00190	H3K9me3	BSS00196

	id	mark	potential.secondary
1	BSS01857	H2AFZ	H3K79me2
2	BSS01261	H2AFZ	H3K36me3
3	BSS01866	H3K27me3	H3K9me3
4	BSS01695	H3K36me3	H3K4me3
5	BSS00268	H3K36me3	H3K79me2
6	BSS01288	H3K36me3	H3K27ac
7	BSS01669	H3K4me1	H3K4me2
8	BSS01860	H3K4me1	H3K4me2
9	BSS01389	H3K4me1	H3K27ac
10	BSS01178	H3K4me2	H3K79me2
11	BSS00439	H3K79me2	H3K9ac
12	BSS00762	H3K79me2	H3K4me2
13	BSS00315	H3K9ac	H3K4me2

Figure S8: Table of flagged samples. **(left)** low agreement tracks, **(right, top)** potential antibody swaps, **(right, middle)** potential sample swaps, **(right, bottom)** potential secondary antibody reactivities or single replicate or experiment swaps.

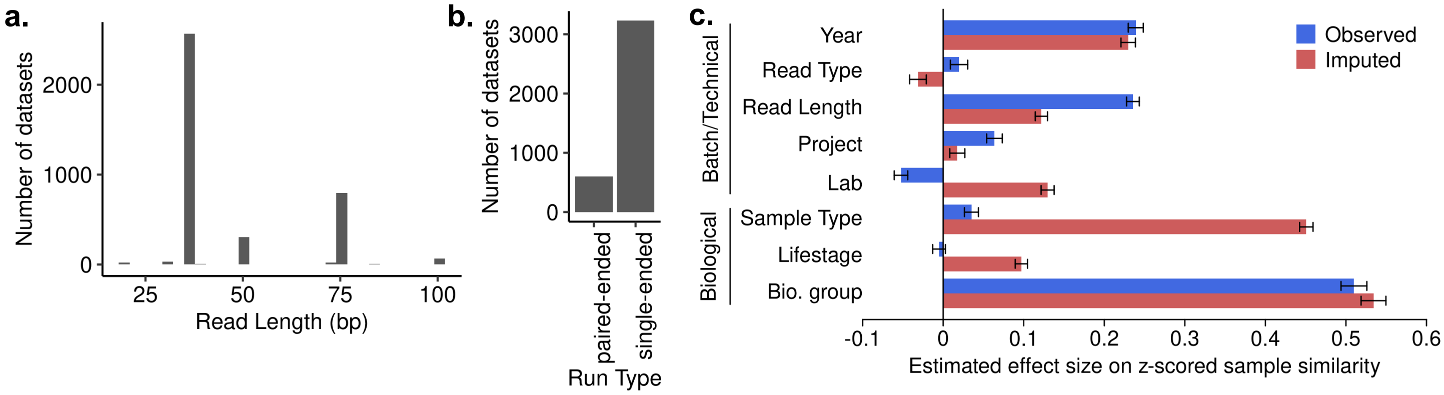

Figure S9: a. Read length distribution in observed datasets before processing. Over two-thirds of observed datasets have read-length of 36bp or lower. **b.** Distribution of sequencing run type in observed datasets. **c.** Estimated effect sizes of technical and biological covariates on the genome-wide correlation between two imputed or two observed datasets in the same mark. We regressed indicator variables for each covariate against per-mark z-scored correlations of all pair-wise combinations of either observed or imputed datasets in each mark. Imputed datasets show lower effect sizes from all technical covariates except for lab or origin and higher effect sizes for all biological covariates than observed datasets. Error bars represent two standard errors.

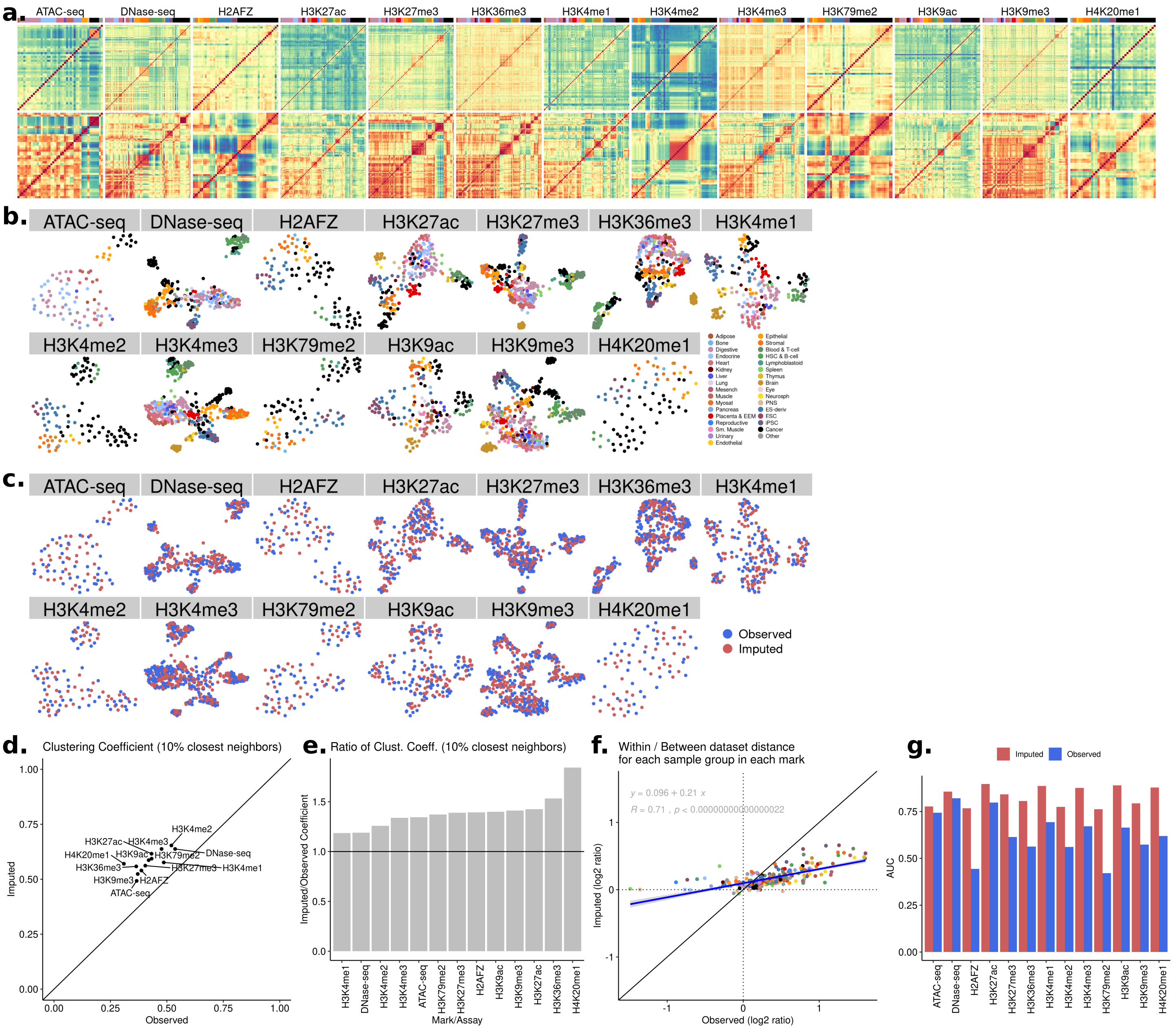

Figure S10: Data clustering and homogeneity in samples with both observed and imputed data. a. Per-mark heatmap of Spearman correlation for observed (top) and imputed (bottom) data in samples with both observed and imputed data in the mark or assay. **b,c.** Joint imputed and observed per-mark dimensionality reduction (UMAP) from Spearman correlation between all imputed and observed tracks in the subset of samples with both datatypes, colored by **b.** sample group and **c.** datatype. **d.** Per-mark observed versus imputed clustering coefficients. Clustering coefficients were computed on per-mark networks for either observed or imputed datasets constructed by connecting each dataset with its top decile of closest neighbors. **e.** Ratio of imputed to observed clustering coefficients. **f.** Ratio of within-group to between-group distance in observed versus imputed data for each sample group (colors) and each mark. Imputed and observed data show concordant ratios (Pearson $R = 0.72$, Spearman $\rho = 0.71$) **g.** AUC for predicting whether a pair of marks is in the same sample group from their pairwise distance. Imputed data outperforms observed data across all marks and assays and achieves $AUC > 0.75$ for most marks.

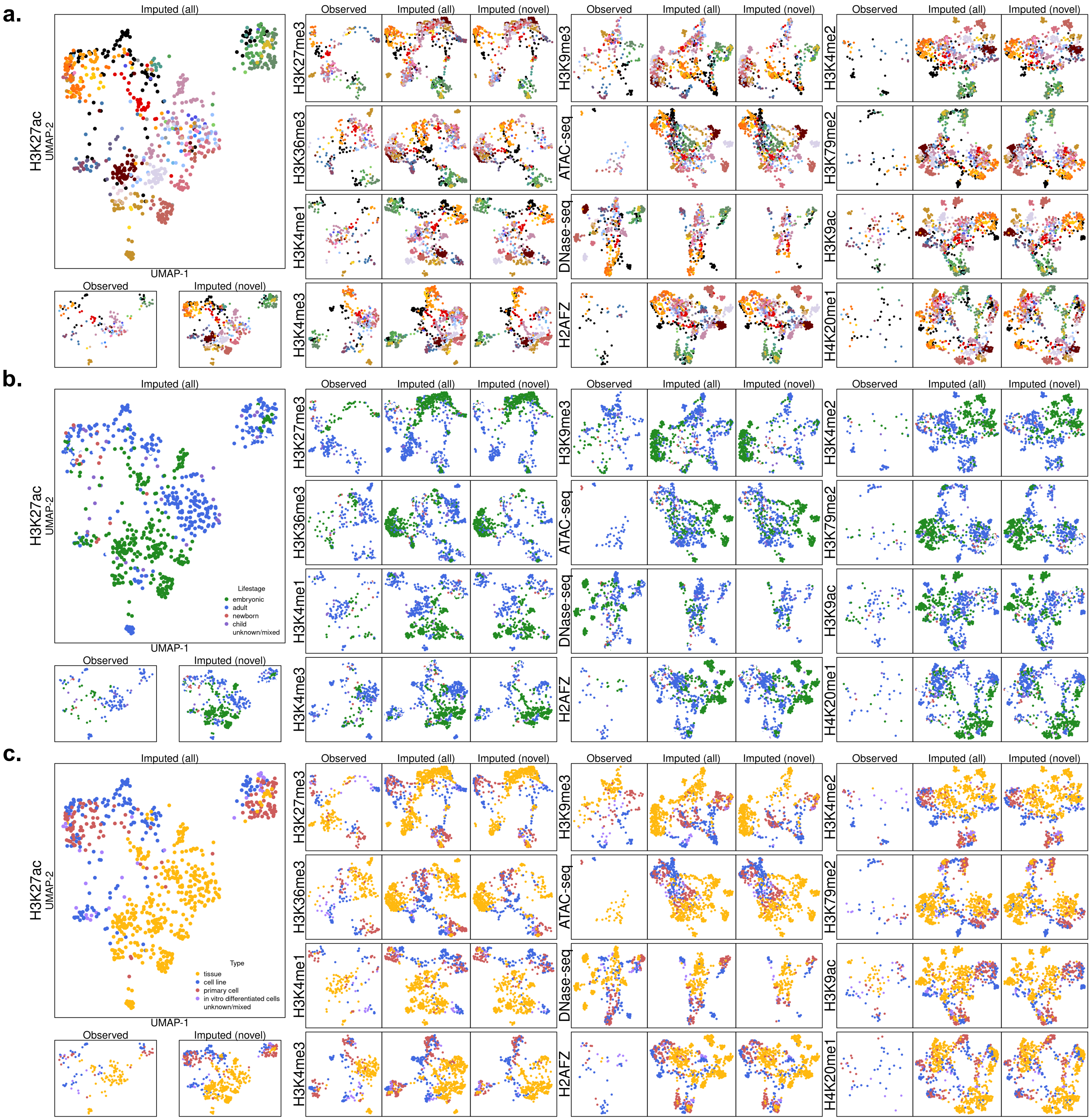

Figure S11: a-c. Joint UMAP embeddings of observed and imputed data within each Tier 1 and 2 mark/assay. Separately, observed, imputed samples, and all novel imputed tracks are plotted for each mark/assay and colored according to **a.** tissue group, **b.** biological lifestage, or **d.** sample type. Imputed UMAP highlights differences in cell types: H3K27ac clusters hematopoietic cells and tissues (green in panel a) closely, reflecting lineage, whereas H3K27me3 clusters iPSC, ESCs, and derived cells (purple/blue in panel a), reflecting differentiation stage. UMAP embedding was calculated from spearman correlation of tracks within regions marked by mark or assay relevant states within the Roadmap compendium.

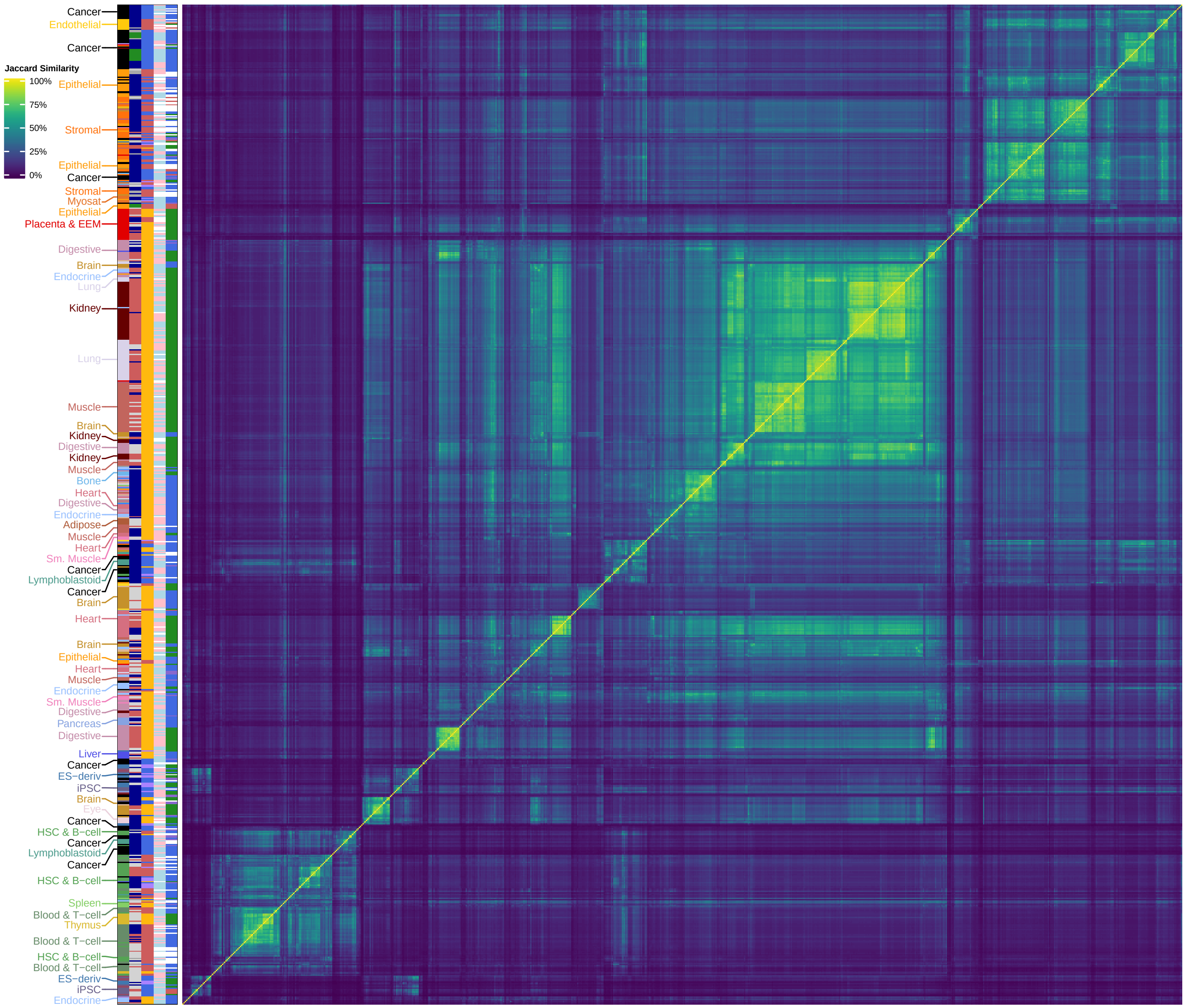

Figure S12: Jaccard similarity matrix (heatmap) across 833 epigenomes (metadata on left) from binarized enhancer activity matrix (2.1M enhancers by 833 epigenomes). Similarity matrix clustered by complete-linkage clustering. Consecutive blocks of at least six samples from the same group are labeled on the left.

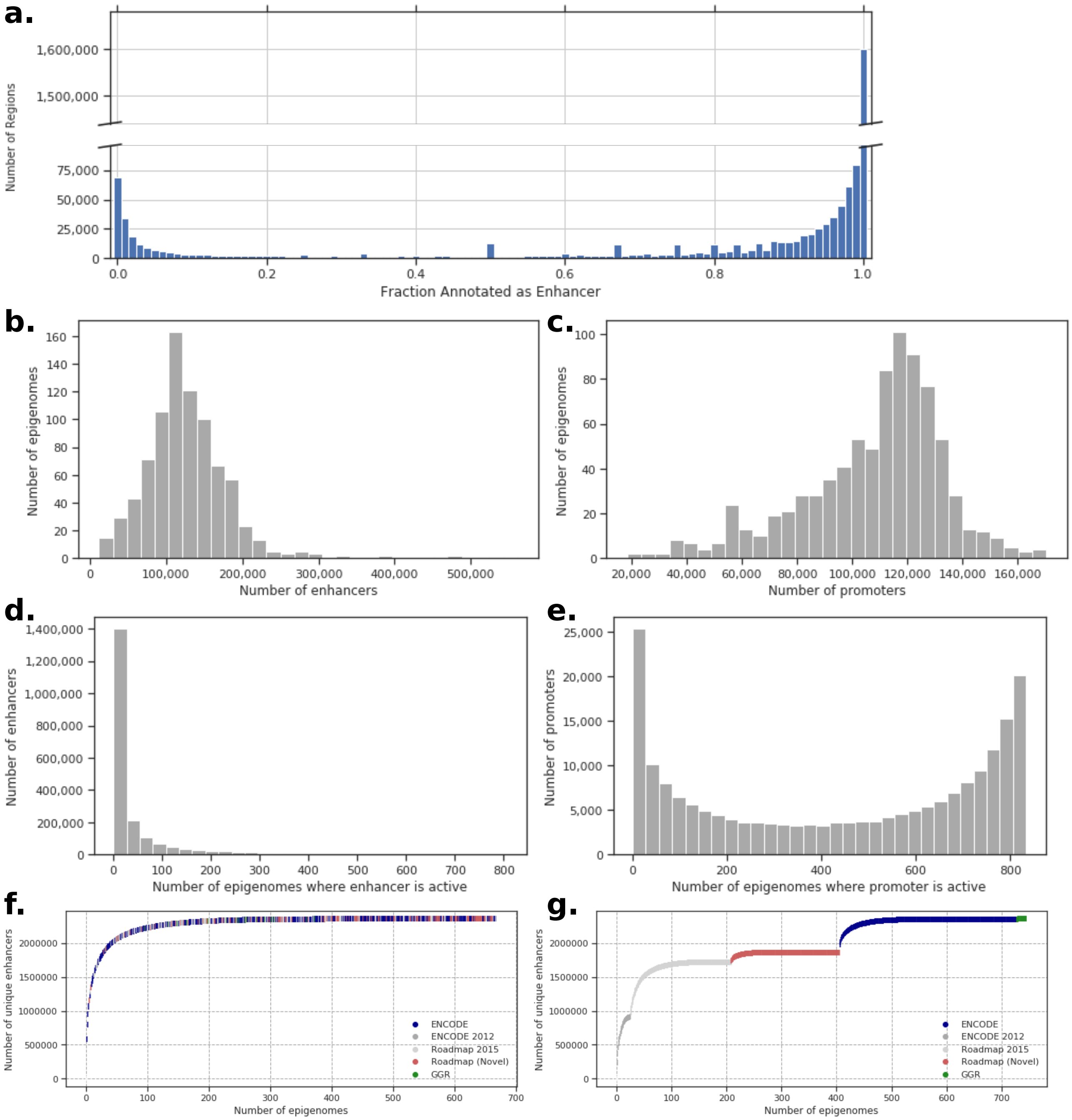

Figure S13: a. Number of DHS sites annotated as enhancers instead of promoters across 833 epigenomes. Most regions are either labeled enhancer (at least 75% of occurrences are enhancers) or promoter (at least 75% of occurrences are promoters) across all of their active occurrences. Using these cutoffs, we labeled 2,069,090 enhancers, 204,104 promoters, and 122,358 dyadic elements (neither specifically promoter or enhancer). **b,c.** Histogram of number of active enhancers (**b**) and promoters (**c**) per sample (row-margins of binary activity matrix). **d,e.** Histogram of the number of samples for which each enhancer (**d**) or promoter (**e**) is an active enhancer (column-margins of binary activity matrix) **f.** Rarefaction curve for enhancer recovery for all enhancer and dyadic elements (2.3M total) across 833 samples (points, colored by project of origin). Curve was created by iteratively adding the sample contributing the most novel active elements until all 2.3M elements were accounted for. **g.** Rarefaction curve for enhancer recovery, shown in order of project completion, for all enhancer and dyadic elements (2.3M total) across 833 samples (points, colored by project of origin). Curve was created by iteratively adding the sample contributing the most novel active elements until all 2.3M elements were accounted for. Samples were considered in order of project publication/completion, only taking samples from the next project when the current project did not contribute any more enhancers.

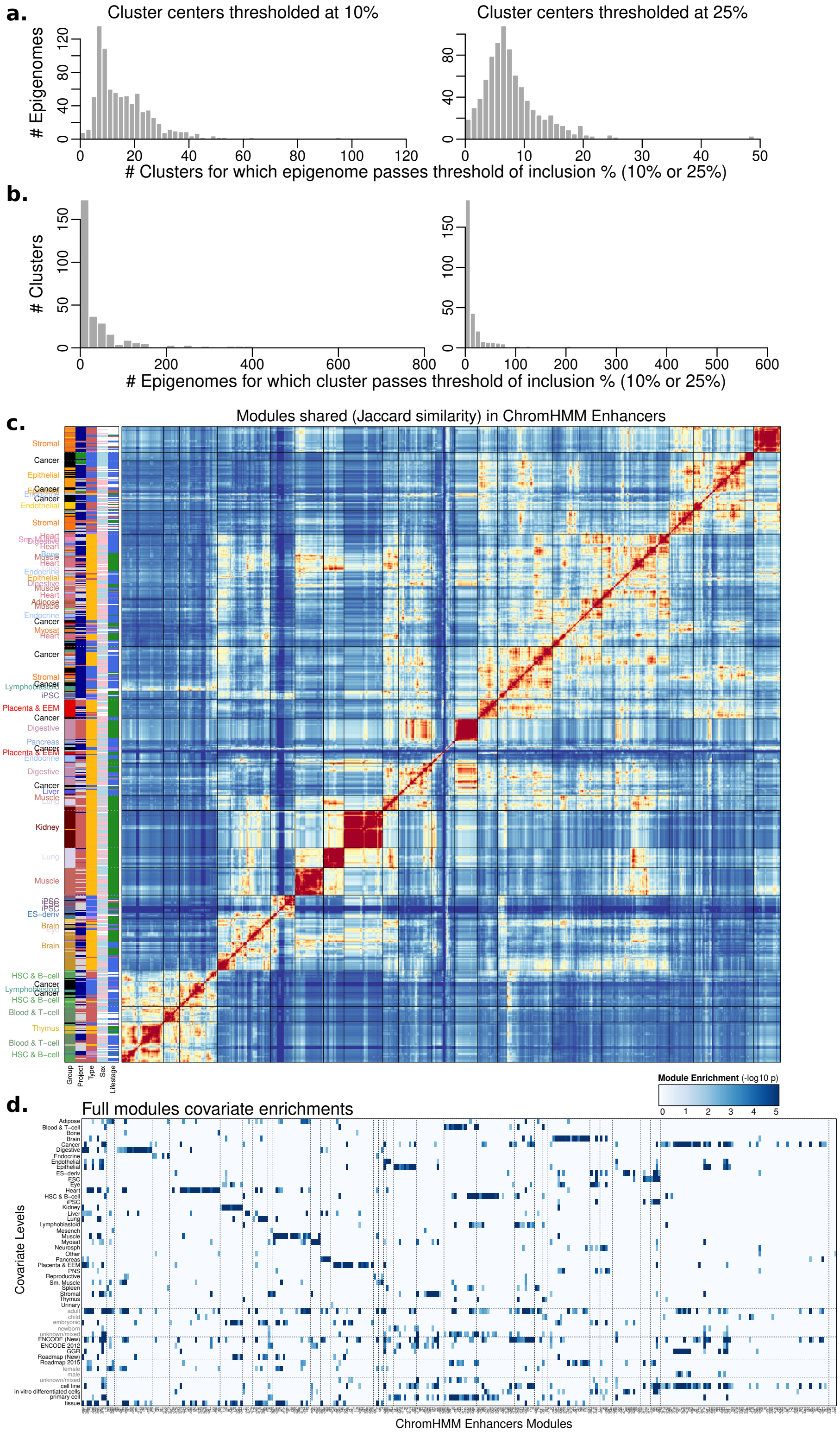

Figure S14: a,b. Module inclusion distributions according to two different module inclusion cutoffs of 10% and 25% (columns). **a.** Number of modules (N=300) for each of 833 epigenomes with module inclusion of at least 10% or 25% (top). **b.** Number of epigenomes (N=833) for each of 300 modules at the same inclusion cutoffs. **c.** Sample similarity (heatmap) across 833 epigenomes by their number of shared modules.(jaccard similarity, intersection over union of modules of each pair of epigenomes). Similarity matrix clustered by Ward's method. Consecutive blocks of at least six samples from the same group are labeled on the left. **d.** Full module enrichments across main metadata facets of group, project, type, sex, and lifestage, for which the reduced version is shown as a panel in Figure 2a. Significance assessed according to hypergeometric test on the module centers matrix with module inclusion of at least 25%.

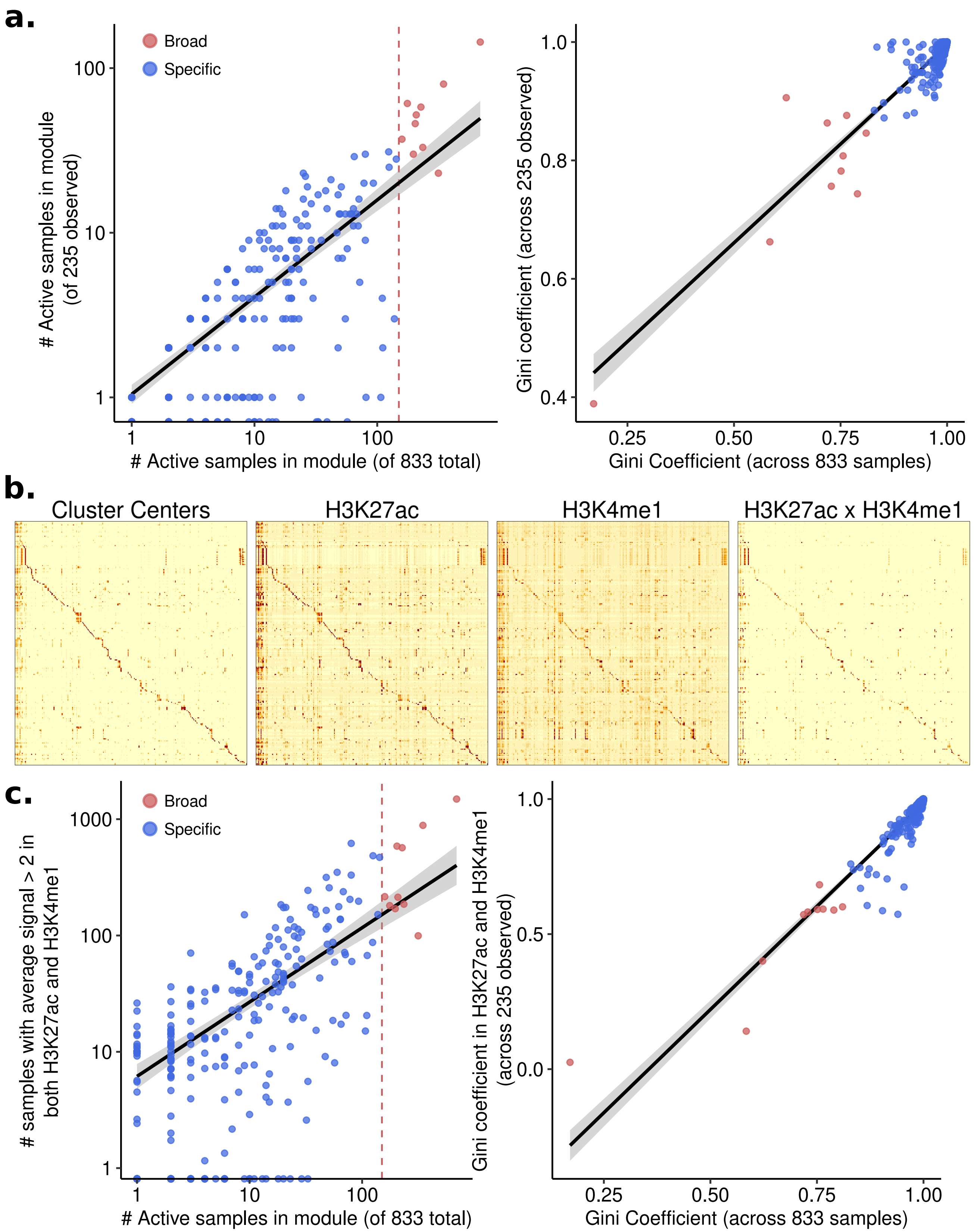

Figure S15: Observed data validation of broad and specific modules. a. Validation with 235 observed H3K27ac datasets. Number of samples (left) and Gini coefficient (right) per module in the full data versus the observed-only H3K27ac data. Broad modules are defined as modules with over 150 actie samples in the full data (dashed line) and labeled in red. Despite the sample composition differences in observed vs. full data, the fraction of active samples is quite consistent between the datasets (left, R^2=0.802, right R^2=0.82). For the 10 broad modules (red) from the full data, all were active in at least 9.7

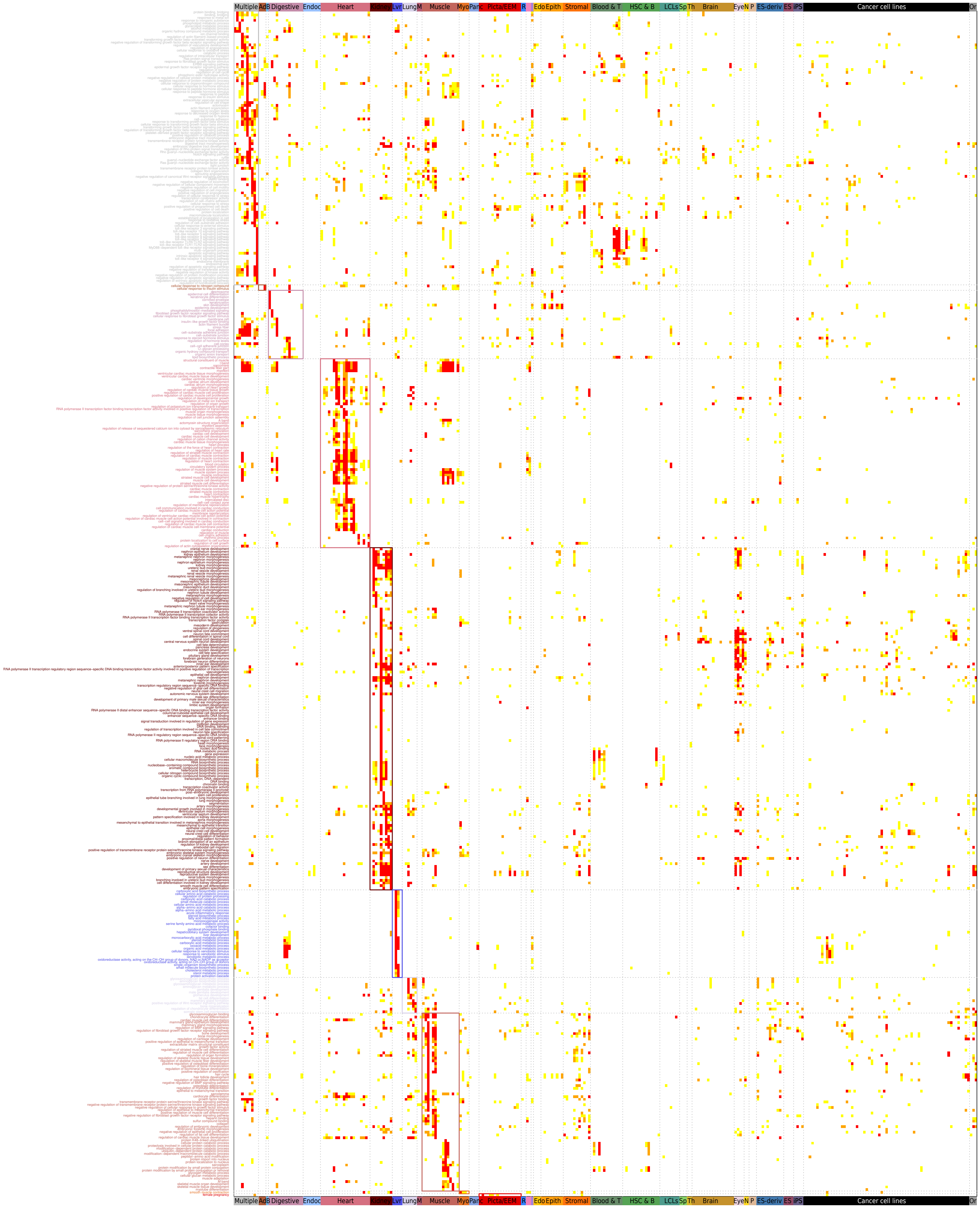

Figure S16: All 865 module-specific GO terms (BP, MF, CC), including all terms enriched in less than 10% of modules and with a maximum enrichment of at least $-\log_{10} p > 4$. Full version of Figure 2b.

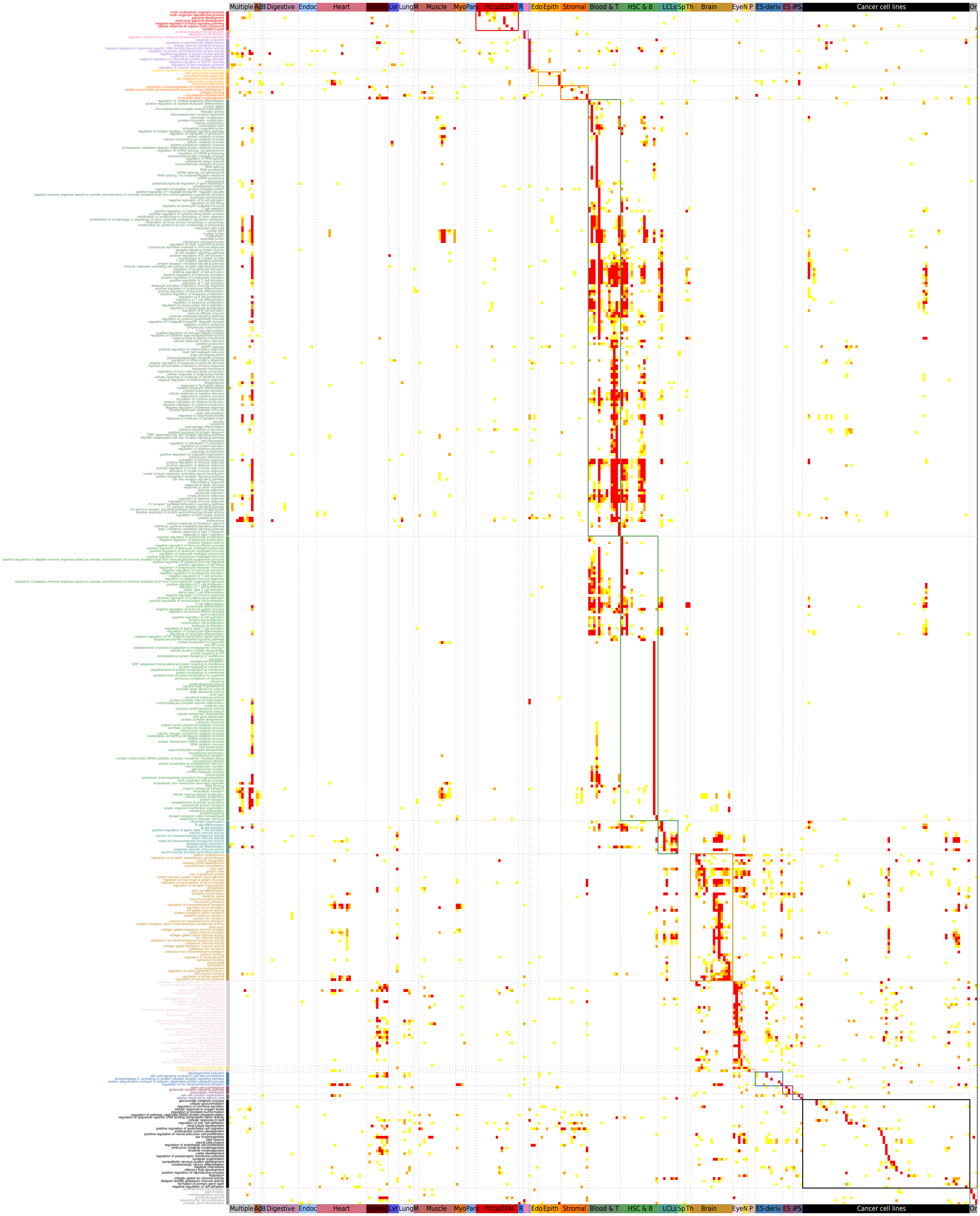

Figure S16: (continued)

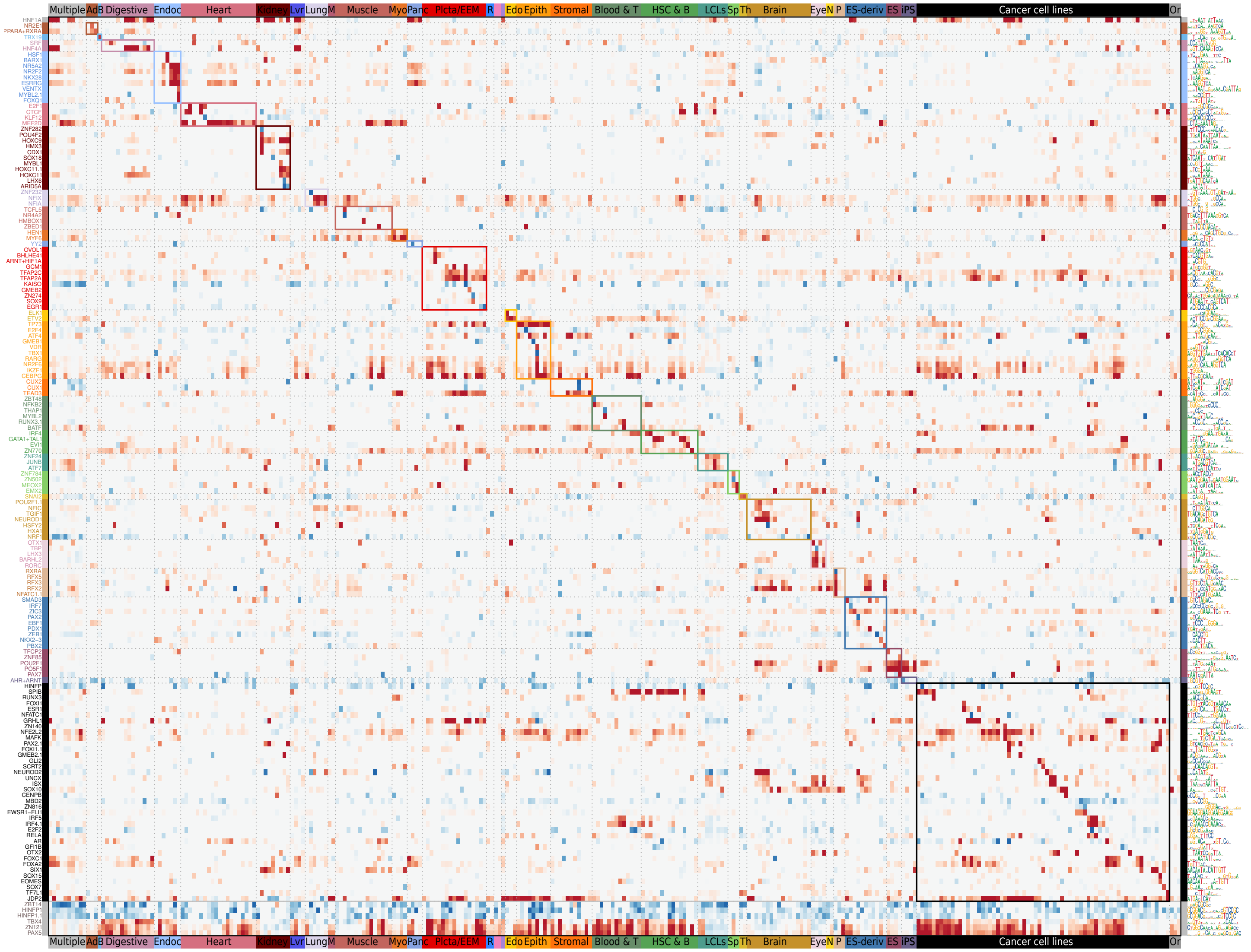

Figure S17: Full motif enrichments for 160 motif families with enrichments with log2FC >= 1 on modules (Figure 2c).

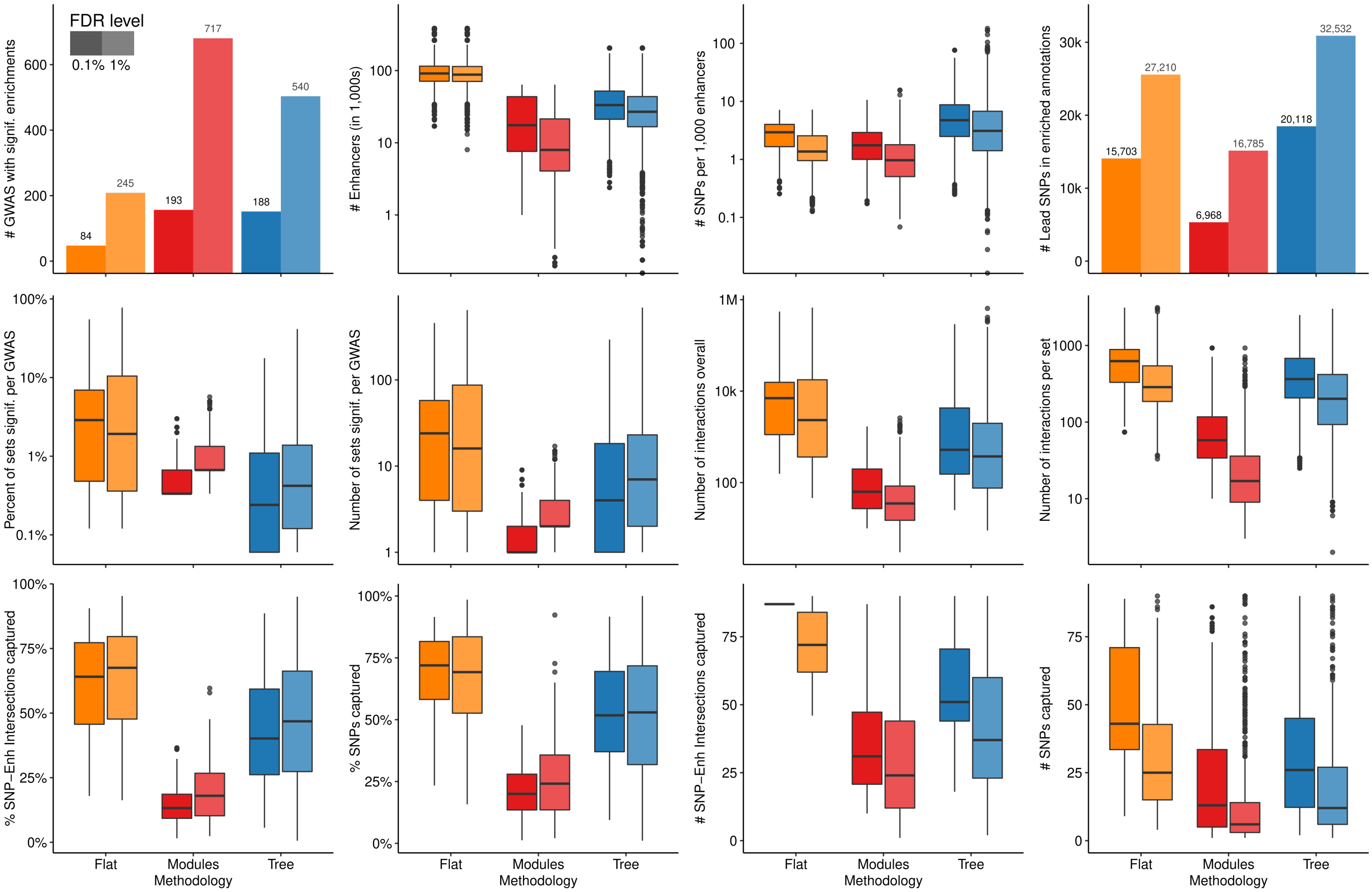

Figure S18: Extended method validation figures comparing epigenomic GWAS enrichments using different methodologies (x-axis) for three FDR cutoffs (shades). First and last figures from top row are shown as Extended Data Fig. 7e.

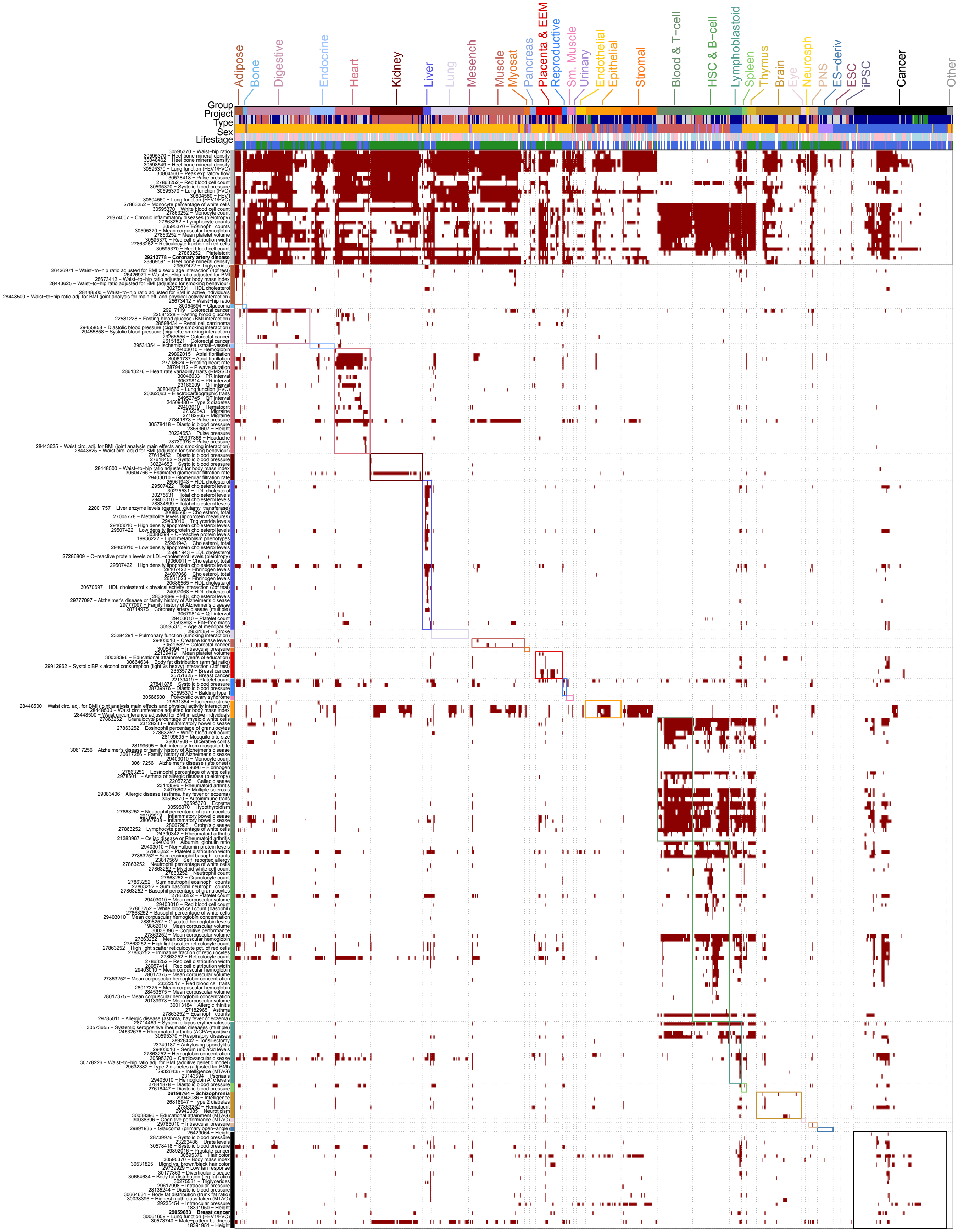

Figure S19: All epigenome trait enrichments at FDR < 1%, for 245 traits (rows) and 833 epigenomes (columns)

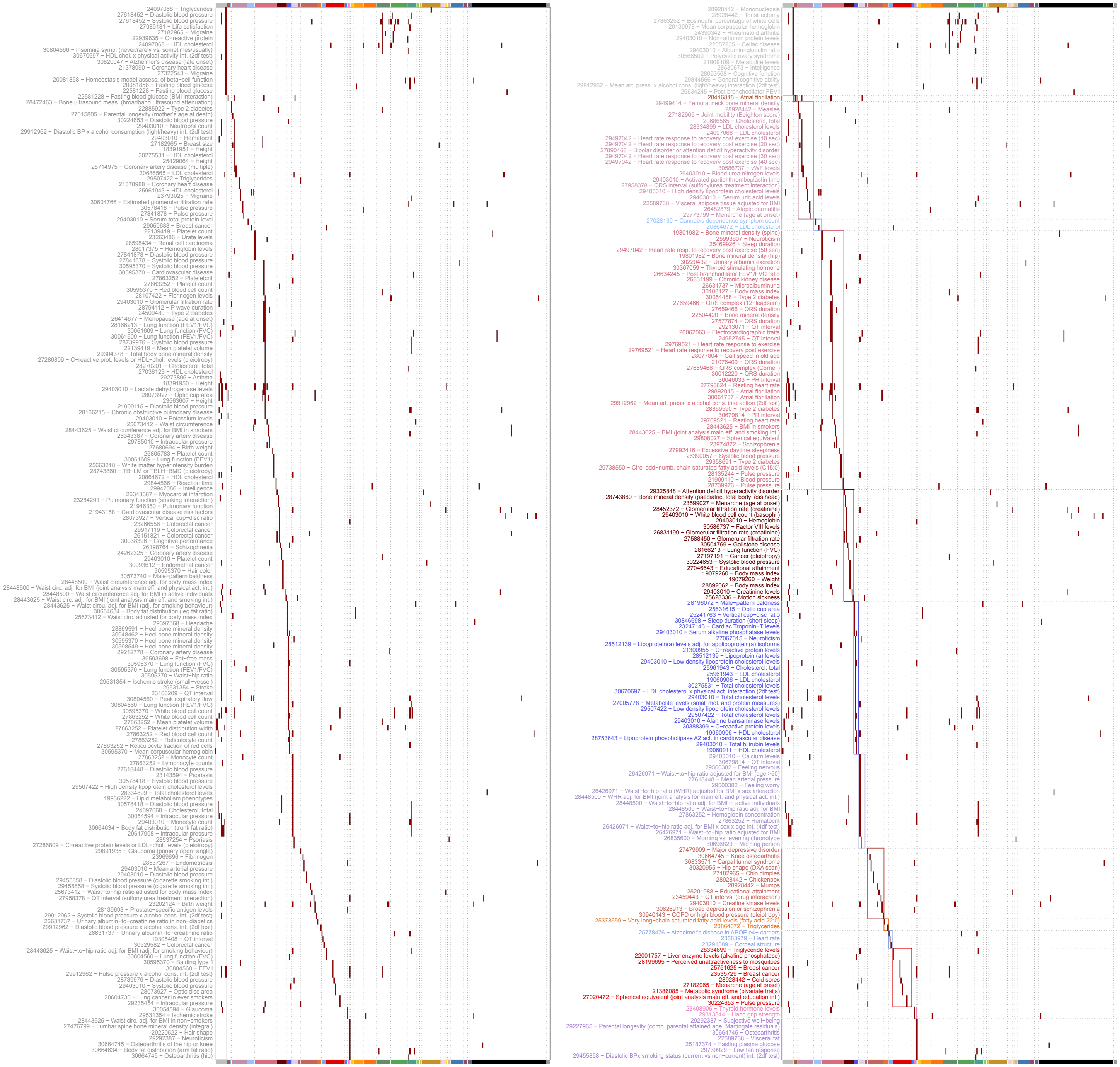

Figure S20: All module trait enrichments at FDR < 1%, for 717 traits (rows) and 300 modules (columns). Modules are ordered as in Figure 2a.

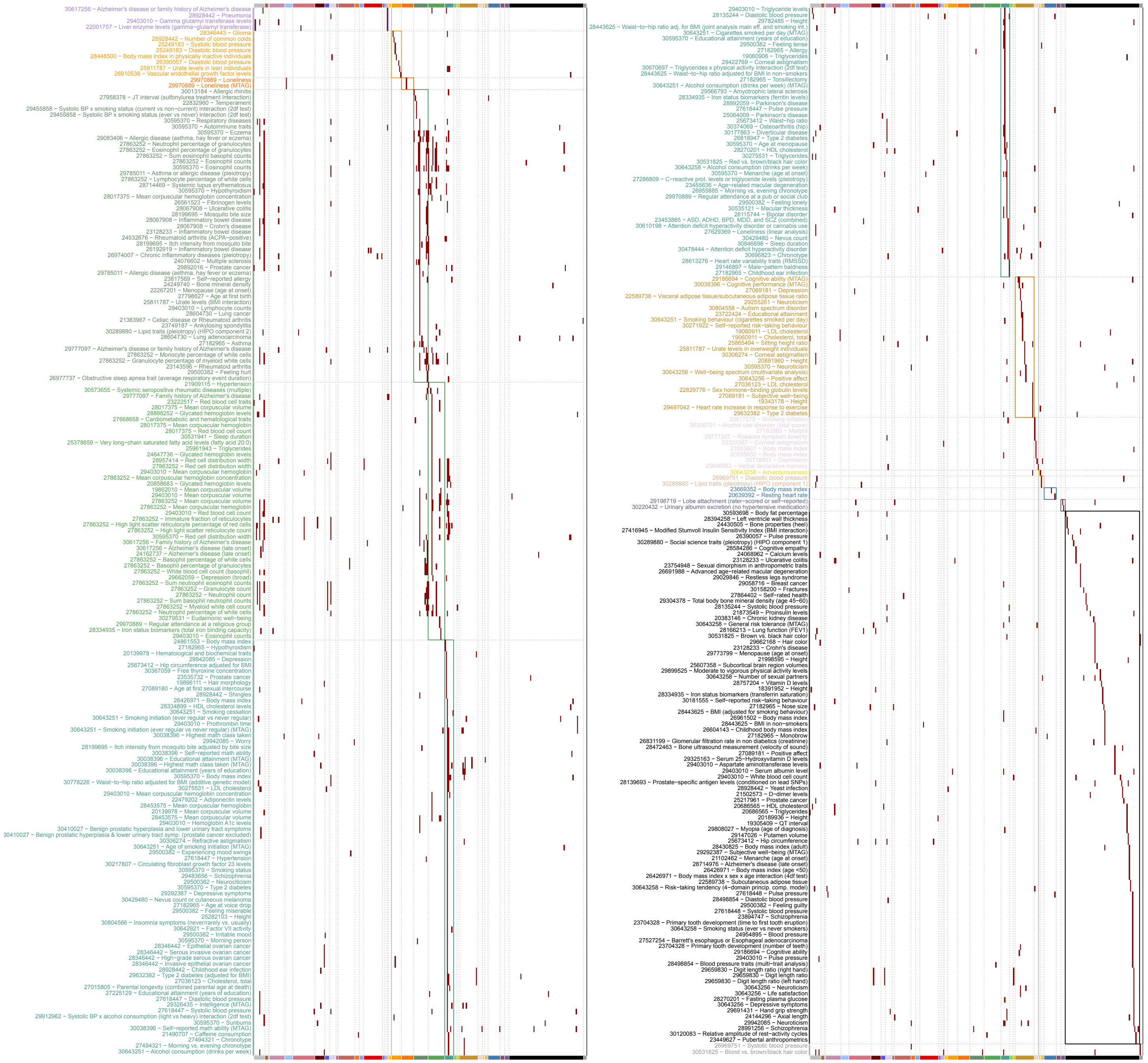

Figure S20: (continued)

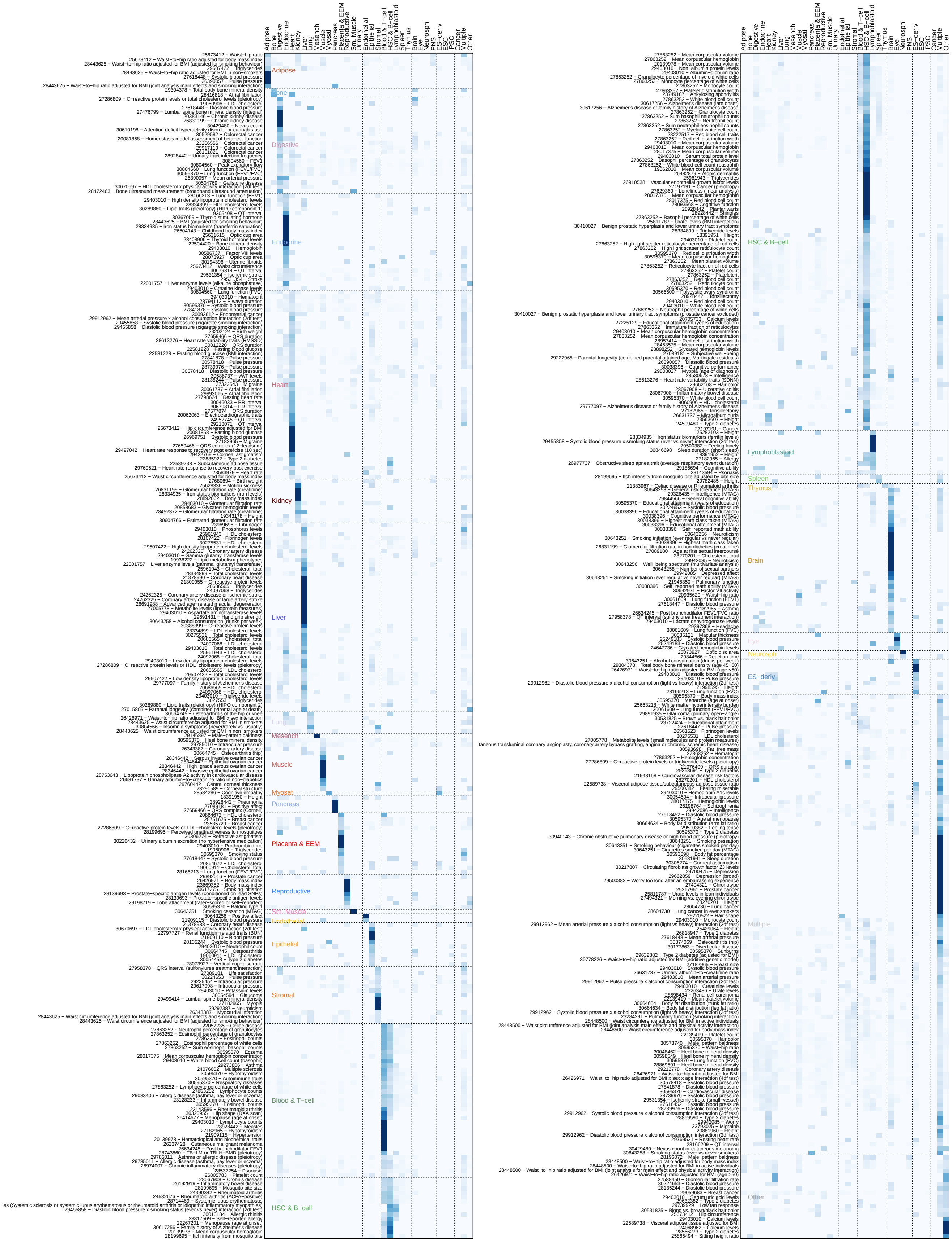

Figure S21: Overall tissue-level prioritization of 540 traits at FDR $< 1\%$. Heatmap represents trait (rows) vs. tissue matrix and is split for visibility. Traits are diagonalized according to their top tissue enrichment, and values are the trait-normalized and tissue aggregated $-\log_{10}$p-values.

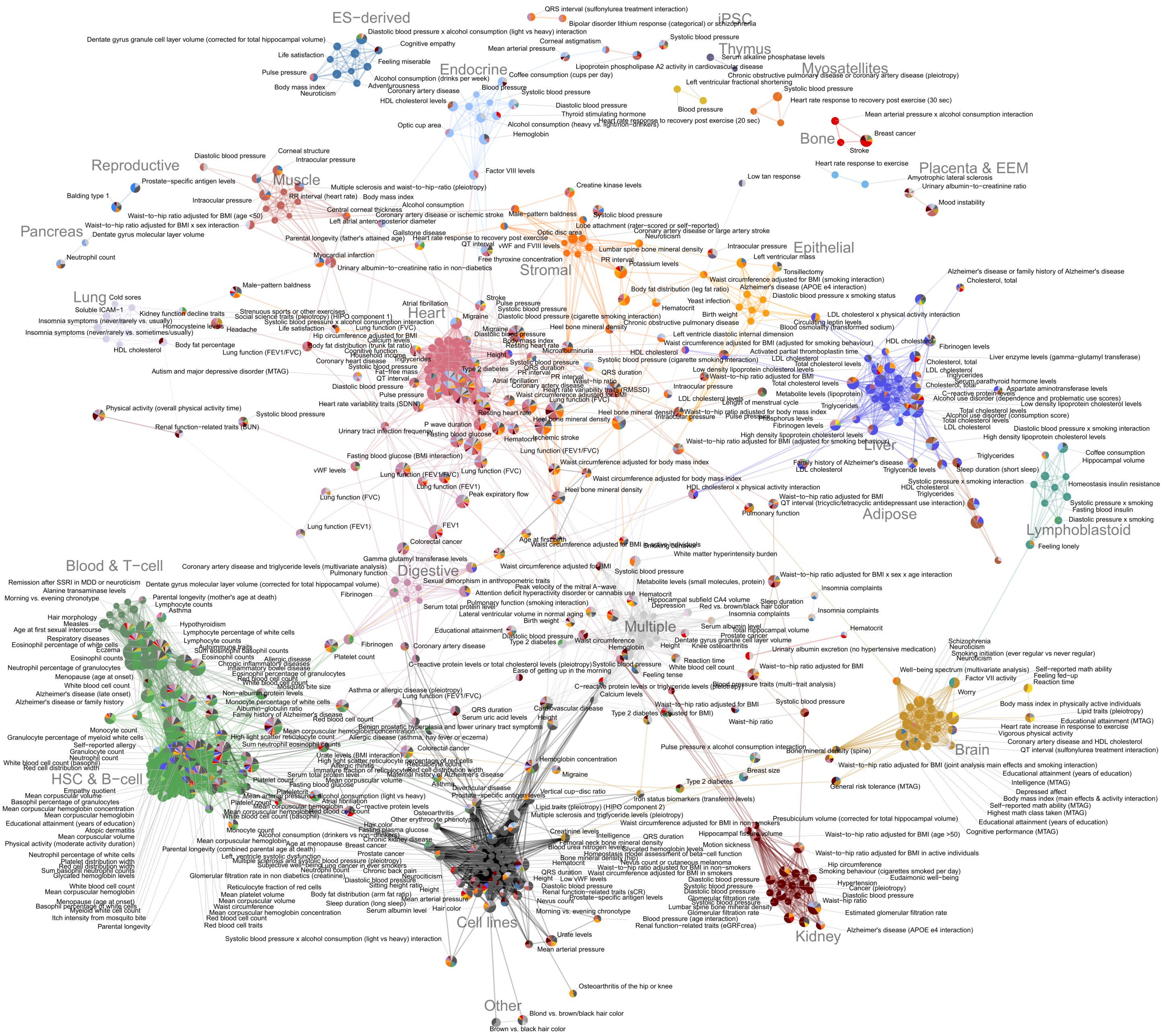

Figure S22: Trait-trait network (as Figure 5) across 511 traits by similarity of epigenetic enrichments (cosine sim. ≥ 0.75), laid out using the Fruchterman-Reingold algorithm. Traits (nodes) are colored by contributing groups (pie chart by fraction of -log10p, size by maximal -log10p) and interactions (edges) by the group with maximal dot product of enrichments between two traits. All 511 traits labeled.

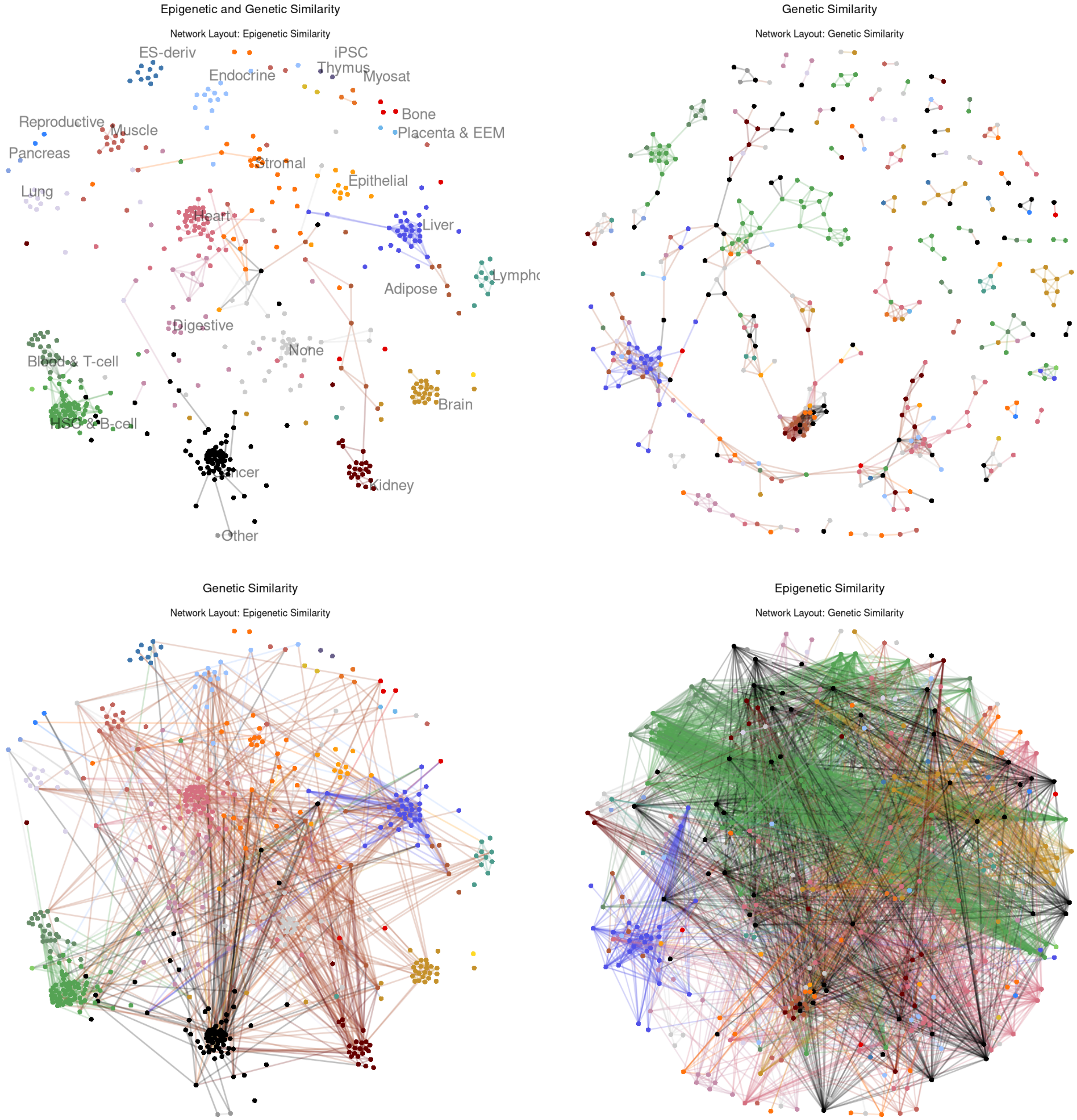

Figure S23: Comparison networks with genetics and epigenetics. (top left) Edges with both high epigenetic similarity and any genetic overlap on epigenetic similarity layout. (bottom left) All trait pairs with genetic overlap (> 5% jaccard similarity of lead SNPs overlapping when binned into 10k bp bins starting from the start of each chromosome, network using epigenetic layout. (top right) Network of trait pairs with any genetic overlap laid out by genetics. (bottom right) All trait pairs with high epigenetic similarity on the genetic layout network

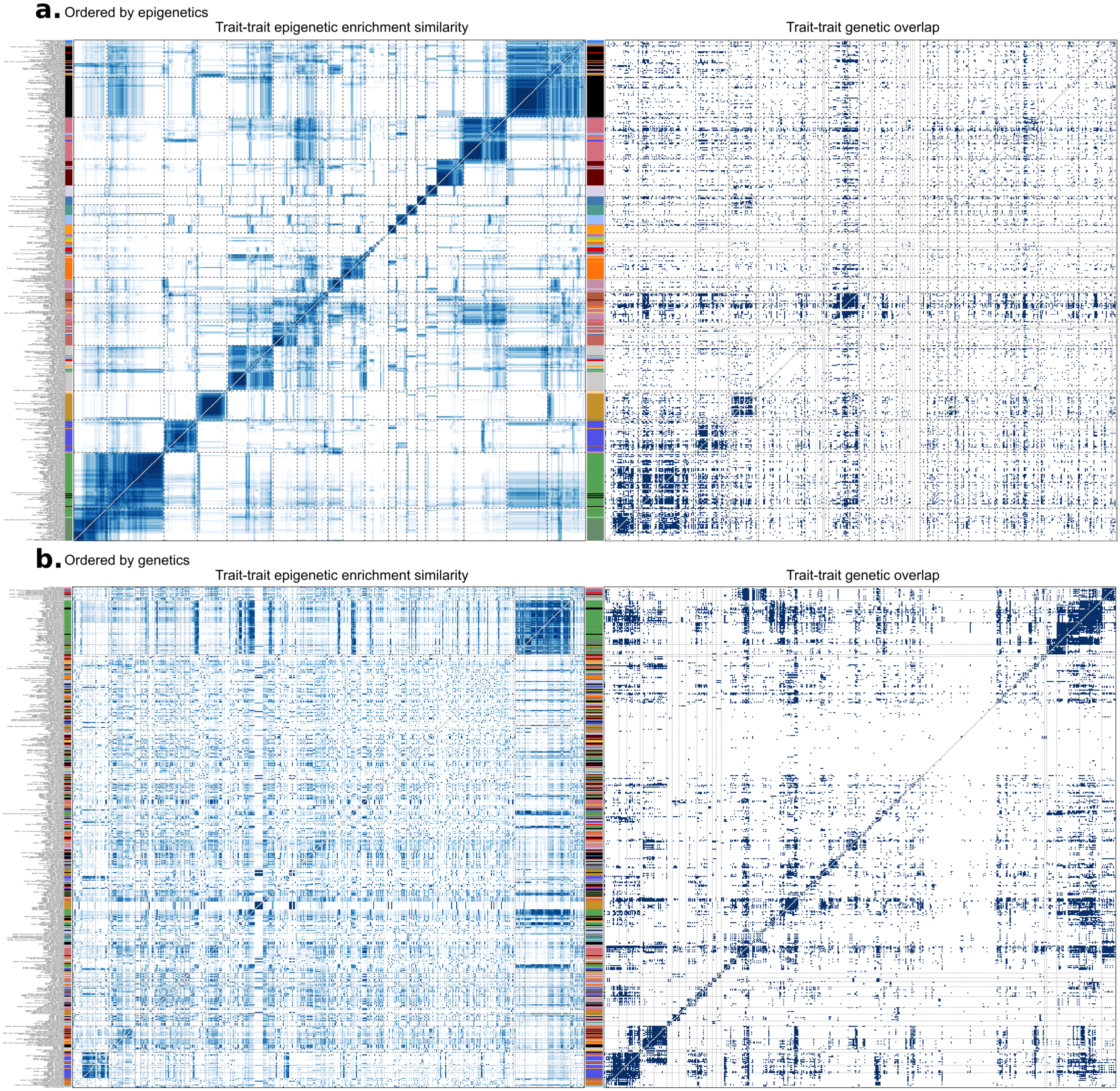

Figure S24: a. Trait-trait similarity matrix by cosine similarity of epigenetic enrichments (left) and by 0/1 genetic similarity (defined as $> 5\%$ jaccard similarity of lead SNPs overlapping when binned into 10k bp bins starting from the start of each chromosome). Matrices are ordered by hierarchical clustering according to Ward's method on the epigenetic matrix. **b.** Epigenetic similarity (left) and genetic similarity (right) matrices, as above. Matrices ordered by hierarchical clustering according to Ward's method on the genetic similarity matrix.

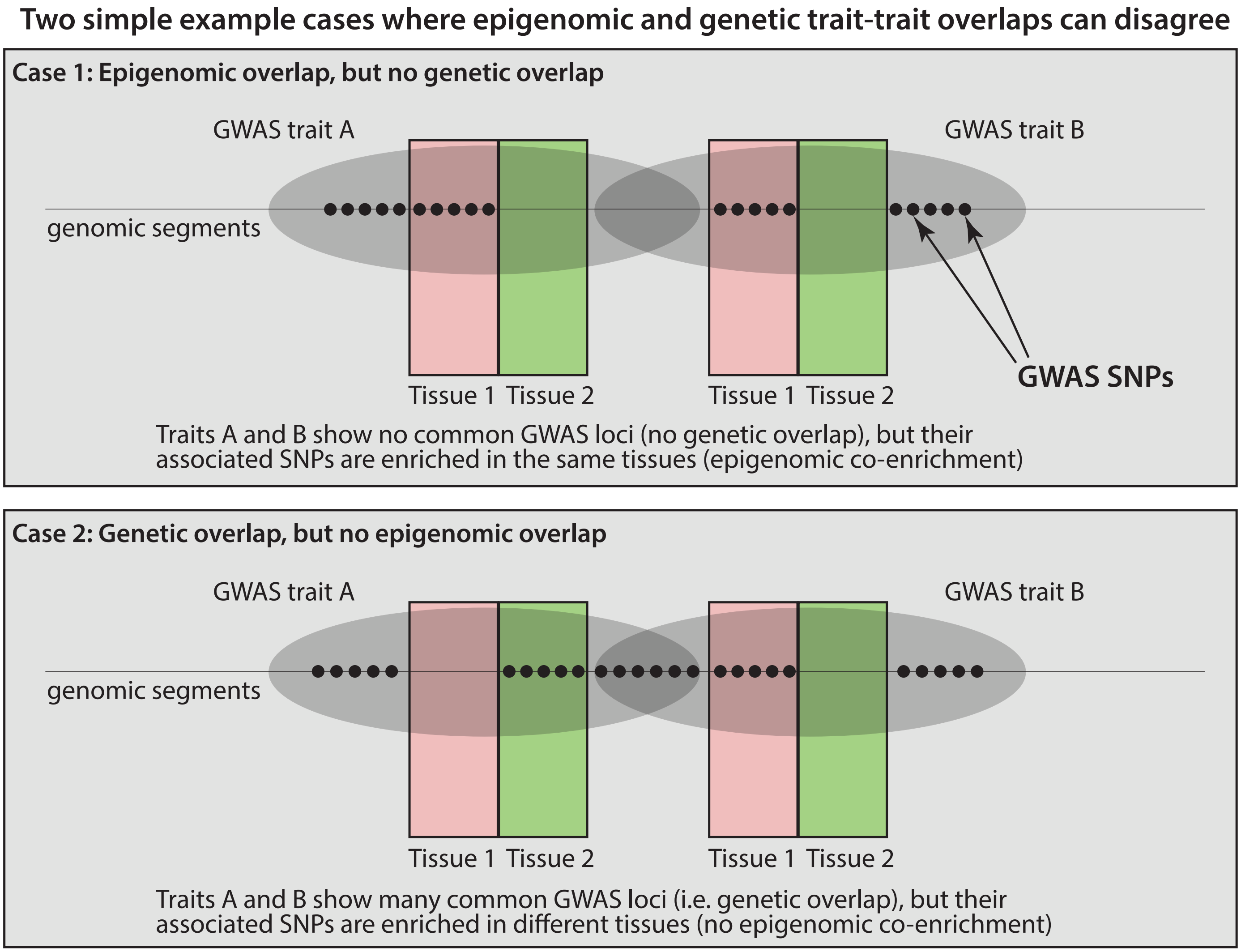

Figure S25: Two example cases where the epigenetic and the genetic trait-trait links may not agree.

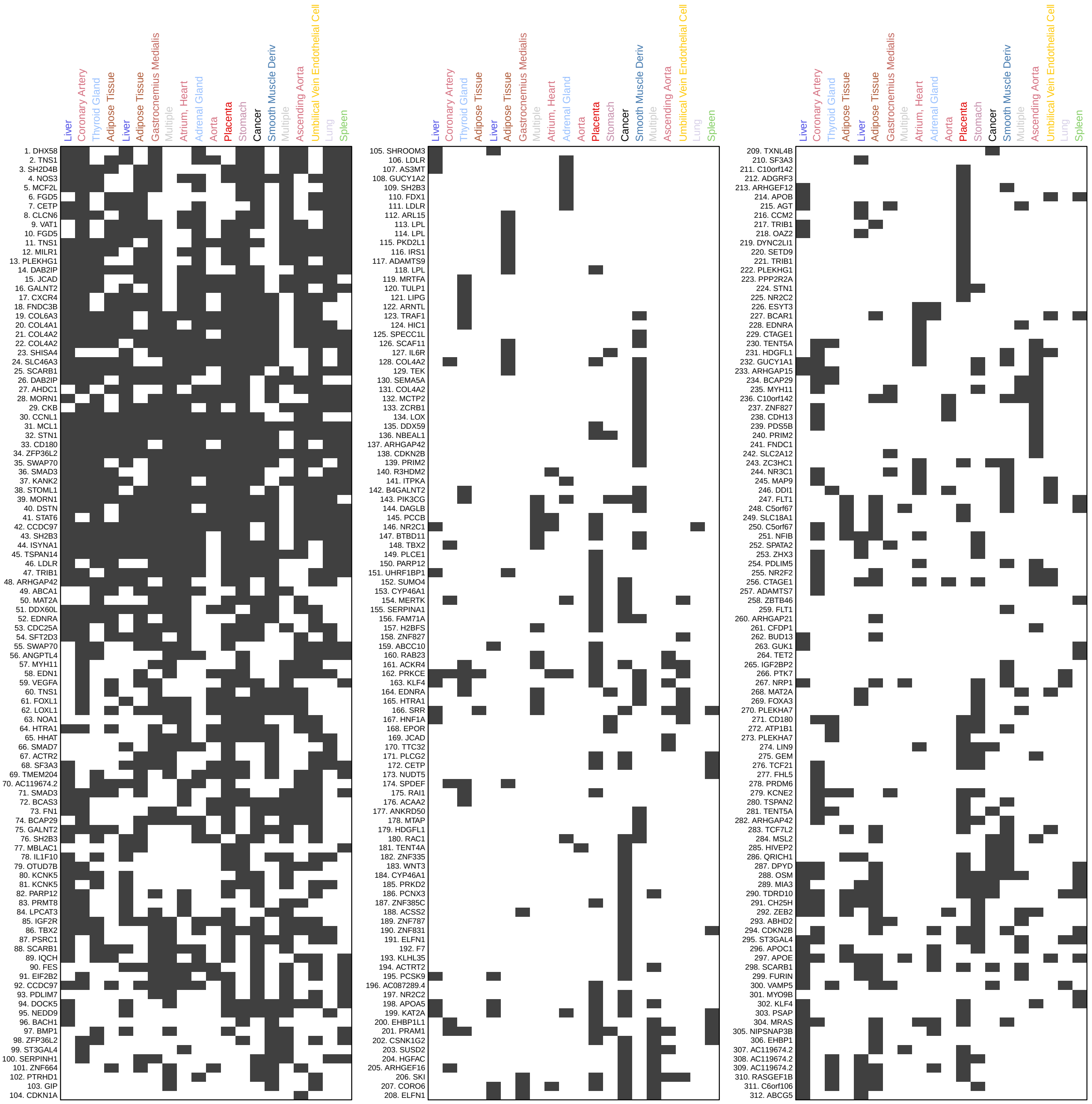

Figure S26: Loci of lead SNPs for CAD in top 20 significantly enriched nodes. Matrix is split in three and shows 312 SNPs against 20 nodes, by presence (black) or absence (white) in the node's enhancers. Loci are ordered by clustering their jaccard similarity across nodes using the Ward method. Each SNP is annotated with its nearest protein-coding gene.

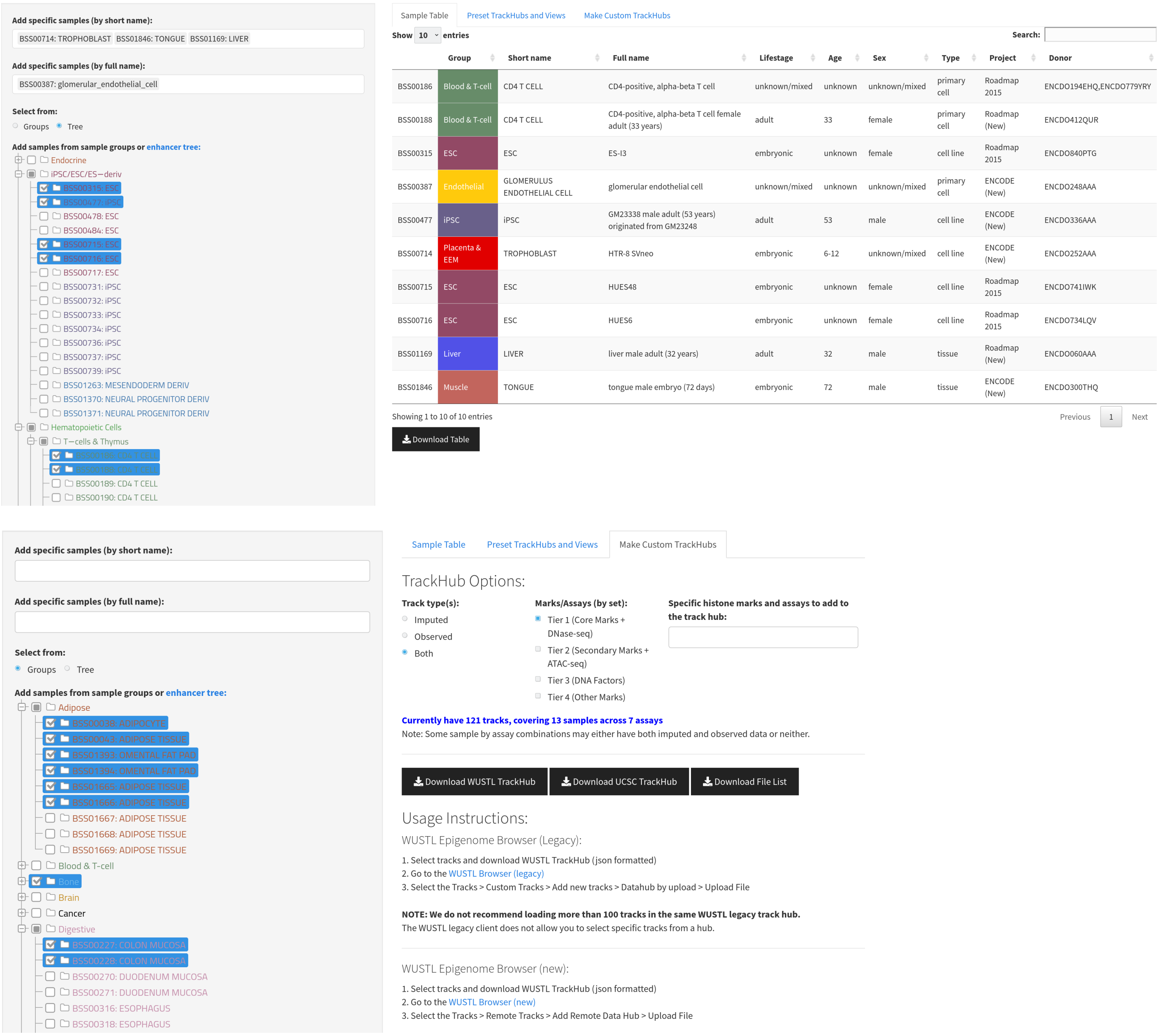

Figure S27: a. Example screenshot from interactive browser showing dataset selection by groups and by enhancer tree, metadata, and custom trackhub creation.

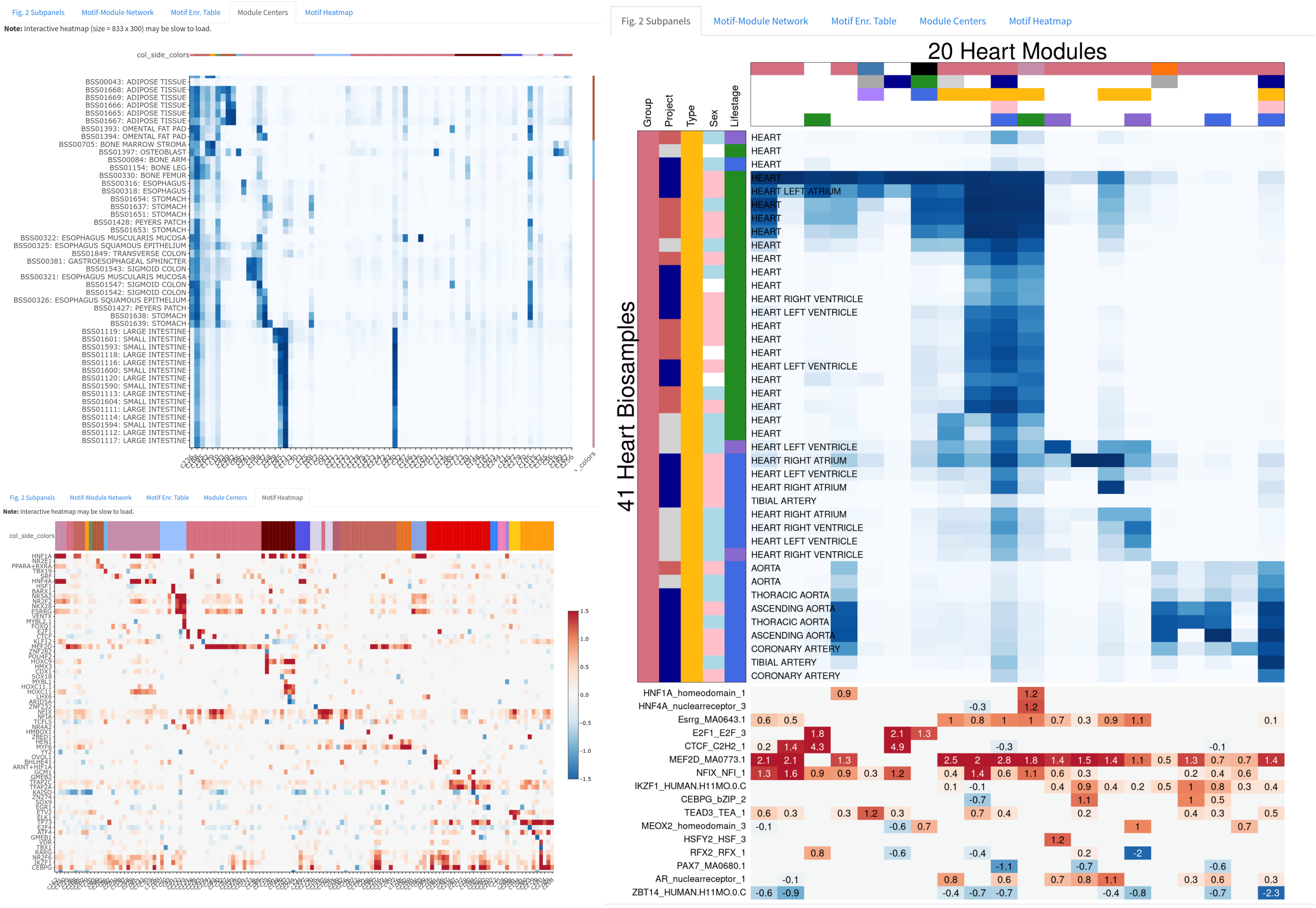

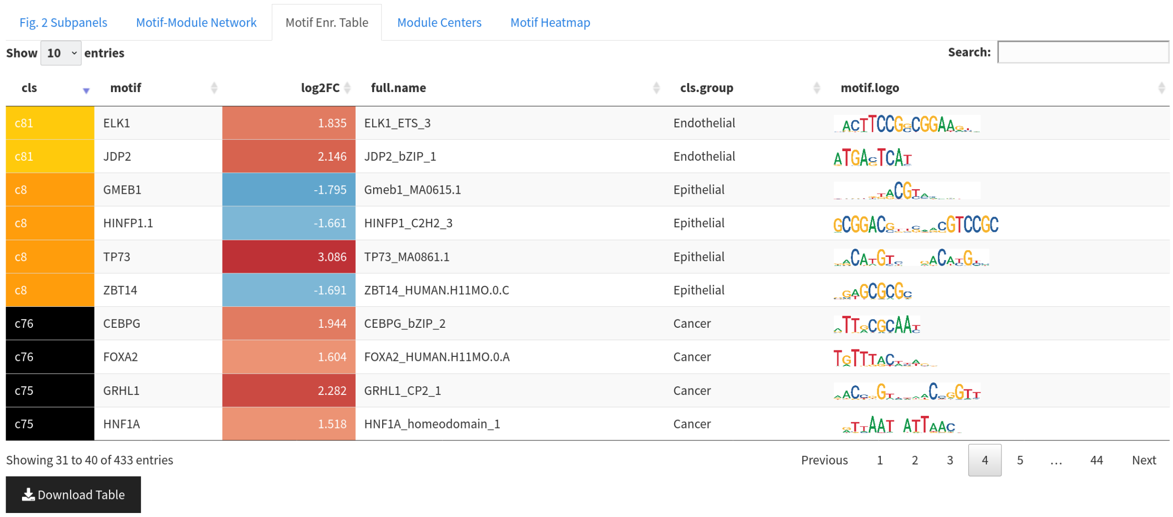

cls	motif	log2FC	full.name	cls.group	motif.logo
c81	ELK1	1.835	ELK1_ETS_3	Endothelial	
c81	JDP2	2.146	JDP2_bZIP_1	Endothelial	
c8	GMEB1	-1.795	Gmeb1_MA0615.1	Epithelial	
c8	HINFP1.1	-1.661	HINFP1_C2H2_3	Epithelial	
c8	TP73	3.086	TP73_MA0861.1	Epithelial	
c8	ZBT14	-1.691	ZBT14_HUMAN.H11MO.0.C	Epithelial	
c76	CEBPG	1.944	CEBPG_bZIP_2	Cancer	
c76	FOXA2	1.604	FOXA2_HUMAN.H11MO.0.A	Cancer	
c75	GRHL1	2.282	GRHL1_CP2_1	Cancer	
c75	HNF1A	1.518	HNF1A_homeodomain_1	Cancer	

Showing 31 to 40 of 433 entries

Previous 1 2 3 4 5 … 44 Next

Download Table

Figure S27: b. Example screenshots from interactive browser showing exploration of modules and motifs, subpanels of Figure 2 for heart, and motif enrichments as a table.

Fig. 2 Subpanels | Motif-Module Network | Motif Enr. Table | Module Centers | Motif Heatmap

Note: Select nodes to see edge and node descriptions below the network.

Select by id

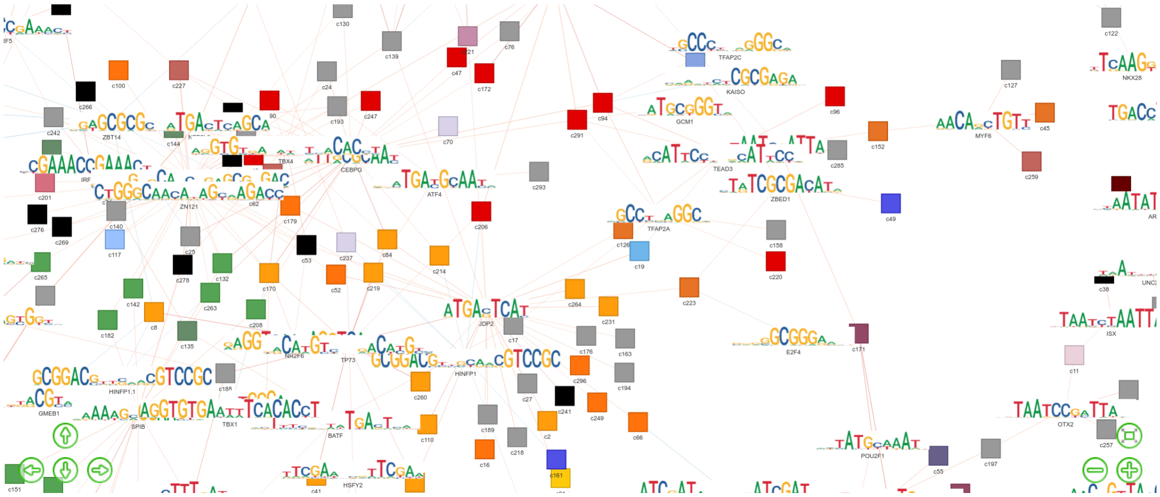

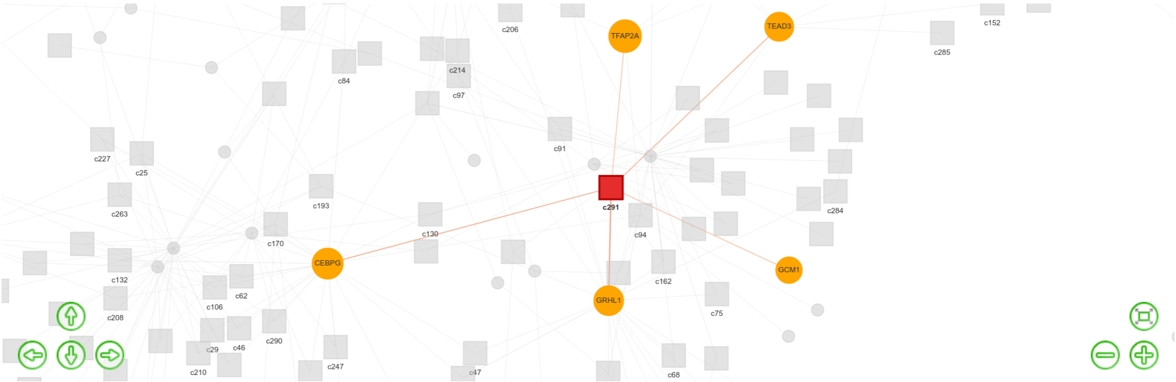

Network edges (for selected node):

Show 10 entries   Search:

cls	motif	log2FC	full.name	cls.group	motif.logo
c291	GRHL1	2.414	GRHL1_CP2_1	Placenta/EEM	
c291	CEBPG	1.913	CEBPG_bZIP_2	Placenta/EEM	
c291	TEAD3	1.814	TEAD3_TEA_1	Placenta/EEM	
c291	TFAP2A	1.552	TFAP2A_AP2_4	Placenta/EEM	
c291	GCM1	1.547	GCM1_GCM_1	Placenta/EEM	

Showing 1 to 5 of 5 entries   Previous 1 Next

Node summary (samples in cluster/motif logo):

Show 10 entries   Search:

cls	id	inclusion	name	group	type	lifestage
c291	BSS01852	0.91	TROPHOBLAST	Placenta/EEM	primary cell	embryonic
c291	BSS01853	0.8	TROPHOBLAST	Placenta/EEM	primary cell	embryonic
c291	BSS01435	0.79	PLACENTA	Placenta/EEM	tissue	embryonic
c291	BSS01859	0.76	TROPHOBLAST	Placenta/EEM	tissue	embryonic
c291	BSS01436	0.55	PLACENTA	Placenta/EEM	tissue	embryonic
c291	BSS01860	0.4	TROPHOBLAST	Placenta/EEM	tissue	embryonic
c291	BSS01440	0.37	PLACENTA	Placenta/EEM	tissue	embryonic

Figure S27: c. Example screenshots from interactive browser showing motif-module network and selection of specific module nodes to show enriched motifs and all included samples in the module.

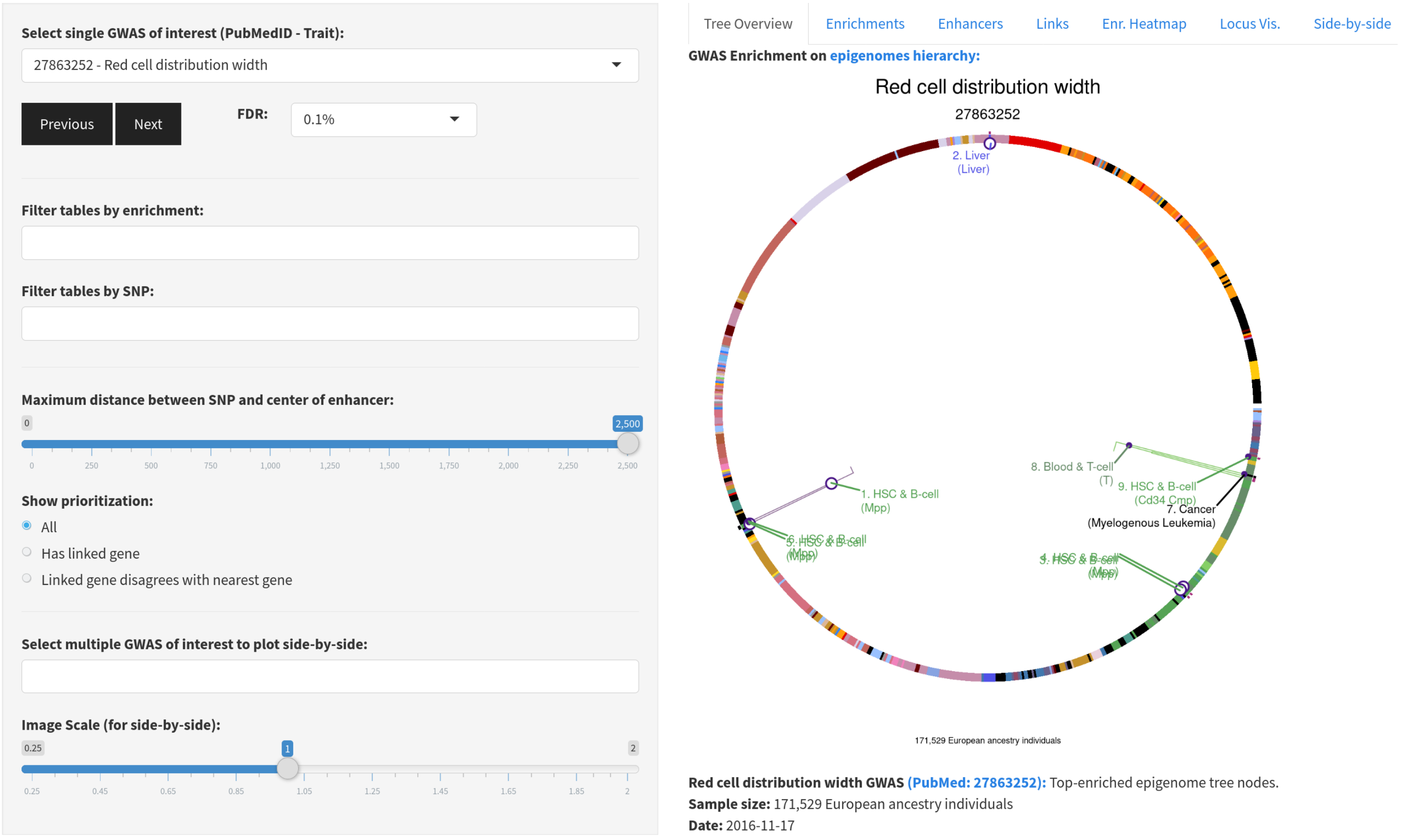

Red cell distribution width GWAS (PubMed: 27863252): Top-enriched epigenome tree nodes and nominal enrichment p-values.
Sample size: 171,529 European ancestry individuals
Date: 2016-11-17

Show 10 **entries** **Search:**

	enrRank	enrName	enr.pValue	pubMedID	trait	enrGroup
1	1	Mpp	5e-73	27863252	Red cell distribution width	HSC & B-cell
2	2	Liver	1.9e-66	27863252	Red cell distribution width	Liver
3	3	Mpp	7.6e-46	27863252	Red cell distribution width	HSC & B-cell
4	4	Mpp	1.2e-41	27863252	Red cell distribution width	HSC & B-cell
5	5	Mpp	6.2e-39	27863252	Red cell distribution width	HSC & B-cell
6	6	Mpp	1.4e-36	27863252	Red cell distribution width	HSC & B-cell
7	7	Myelogenous Leukemia	1.5e-31	27863252	Red cell distribution width	Cancer
8	8	T	1e-29	27863252	Red cell distribution width	Blood & T-cell
9	9	Cd34 Cmp	4.3e-28	27863252	Red cell distribution width	HSC & B-cell

Showing 1 to 9 of 9 entries — Previous 1 Next

Figure S27: d. Example screenshots from interactive browser showing GWAS browsing and enrichments in figure and table view.

Tissue-specific enhancers near GWAS lead SNPs: All enhancers within 2.5kb of a GWAS lead SNP that are also active in one of the top-enriched tree nodes in the GWAS.

Show 10 entries Search:

	chr	snpPos	snp.pValue	enhStart	enhEnd	distToCenter	nearestGene	enrRank	enrName	enr.pValue	enrGroup
1	chr1	43428757	1e-16	43429771	43430151	1204.5	SLC2A1	9	Cd34 Cmp	4.3e-28	HSC & B-cell
2	chr2	60725451	8e-21	60727115	60727295	1754.5	BCL11A	9	Cd34 Cmp	4.3e-28	HSC & B-cell
3	chr8	26243136	9e-19	26243356	26243576	330.5	BNIP3L	9	Cd34 Cmp	4.3e-28	HSC & B-cell
4	chr1	43428757	1e-16	43429051	43429407	472.5	SLC2A1	9	Cd34 Cmp	4.3e-28	HSC & B-cell
5	chr11	205198	4e-24	202620	202880	2447.5	BET1L	9	Cd34 Cmp	4.3e-28	HSC & B-cell
6	chr1	3691998	1e-17	3691784	3692024	93.5	SMIM1	9	Cd34 Cmp	4.3e-28	HSC & B-cell
7	chr11	44594611	1e-21	44595064	44595290	566.5	CD82	9	Cd34 Cmp	4.3e-28	HSC & B-cell
8	chr8	26243136	9e-19	26244376	26244596	1350.5	BNIP3L	9	Cd34 Cmp	4.3e-28	HSC & B-cell
9	chr19	1014538	1e-12	1016279	1016819	2011.5	TMEM259	9	Cd34 Cmp	4.3e-28	HSC & B-cell
10	chr1	203281175	6e-14	203279688	203279808	1426.5	BTG2	9	Cd34 Cmp	4.3e-28	HSC & B-cell

Showing 1 to 10 of 9,039 entries Previous 1 2 3 4 5 … 904 Next

Download Enhancers

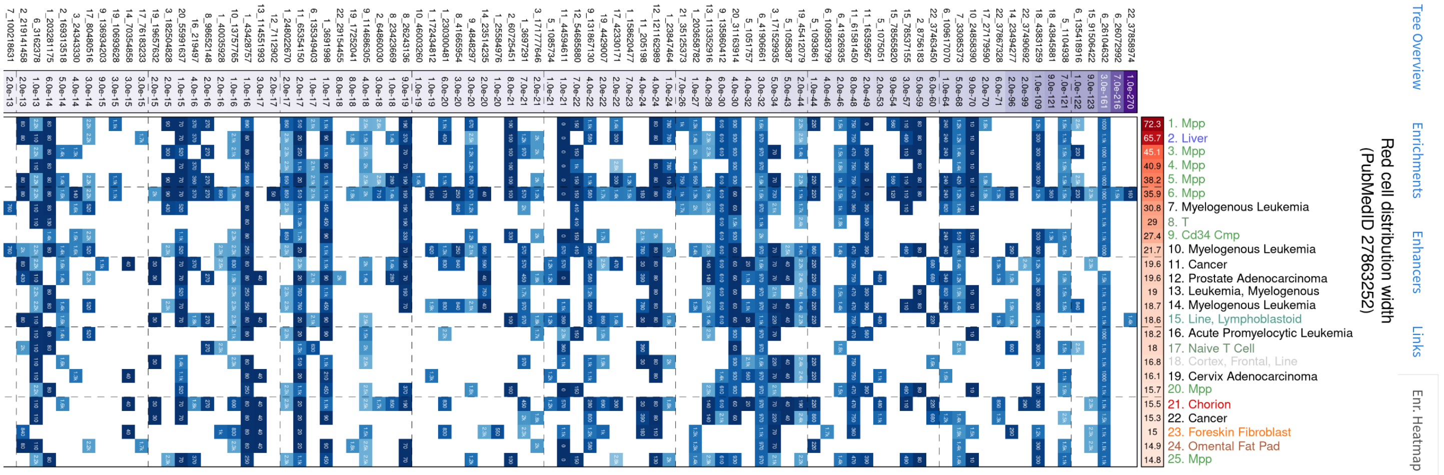

Figure S27: e. Example screenshots from interactive browser showing enhancer predictions nearby GWAS SNPs and enhancer versus subtree intersection, showing the closest enhancer to the SNP in each tissue.

Tree Overview | Enrichments | Enhancers | Links | Enr. Heatmap | Locus Vis. | Side-by-side

Table of gene-enhancer links: Gene-enhancer links in the GWAS loci (SNPs +/- 1Mb), reported for the top-enriched sample groups in the GWAS.

Show 10 entries Search:

	chr	snpPos	snp.pValue	distToCenter	nearestGene	linkedGene	linkScore	linkDist	enrRank	enrName	enr.pValue	enrGroup
6774	chr6	26104632	3e-161	1464	HIST1H4C	HFE	0.89	15658.5	1	Mpp	5e-73	HSC & B-cell
6769	chr6	26104632	3e-161	1621	HIST1H4C	HFE	0.87	15501.5	1	Mpp	5e-73	HSC & B-cell
6764	chr6	26104632	3e-161	996.5	HIST1H4C	HIST1H1T	0.36	-2736	1	Mpp	5e-73	HSC & B-cell
2563	chr6	26104632	3e-161	1199	HIST1H4C	HFE	0.84	15923.5	2	Liver	1.9e-66	Liver
2575	chr6	26104632	3e-161	1060.5	HIST1H4C	HFE	0.84	16062	2	Liver	1.9e-66	Liver
2759	chr6	26104632	3e-161	1621	HIST1H4C	HFE	0.9	15501.5	3	Mpp	7.6e-46	HSC & B-cell
2954	chr6	26104632	3e-161	1199	HIST1H4C	HFE	0.9	15923.5	3	Mpp	7.6e-46	HSC & B-cell
3149	chr6	26104632	3e-161	1464	HIST1H4C	HFE	0.89	15658.5	3	Mpp	7.6e-46	HSC & B-cell
2804	chr6	26104632	3e-161	1106.5	HIST1H4C	HIST1H1T	0.81	-2626	3	Mpp	7.6e-46	HSC & B-cell
3144	chr6	26104632	3e-161	996.5	HIST1H4C	SLC17A2	0.8	174674	3	Mpp	7.6e-46	HSC & B-cell

Showing 1 to 10 of 1,459 entries Previous 1 2 3 4 5 … 146 Next

Download Links

Select locus:

chr6:135,418,916 (p=1.0e-122, HBS1L)

Previous Next

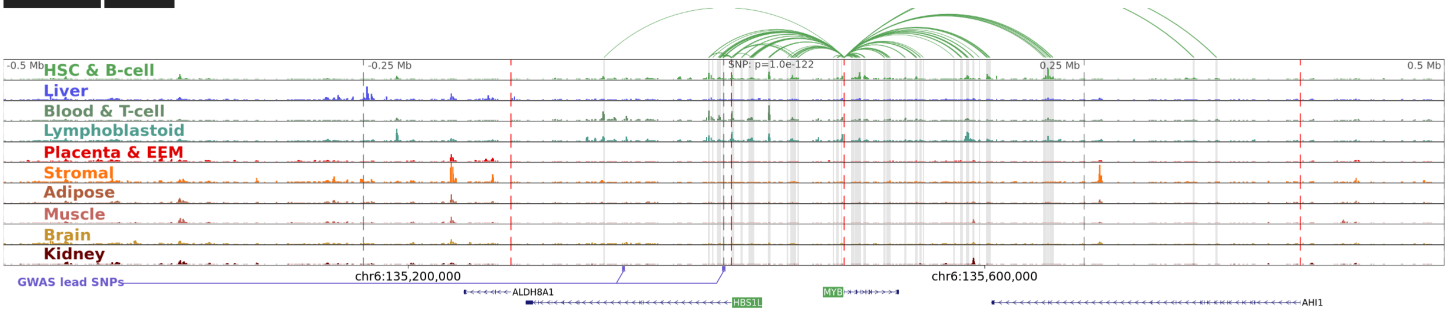

Locus overview for 1Mb around selected lead SNP: Tracks show average H3K27ac signal of enhancers in locus, and red dashed lines indicate the TSSes of nearby genes. Two types of correlation-based links are plotted: **(1)** Links from one of the enhancers near a lead SNP in the enriched epigenomes. **(2)** Any links in the locus present in at least half of the samples in one of the top sample groups (**HSC & B-cell**). Genes linked to an enhancer within 2.5kb of a GWAS lead SNP are highlighted and colored according to the sample group with the highest link score. Link data and images for this GWAS are also available from our **data repository** .
Click to enable/disable zoom on locus, scroll to change zoom size.

Figure S27: f. Example screenshots from interactive browser showing linking predictions on GWAS SNPs and locus visualization with links, enhancers, and prioritized genes.

Original, uncorrected Supplementary Data

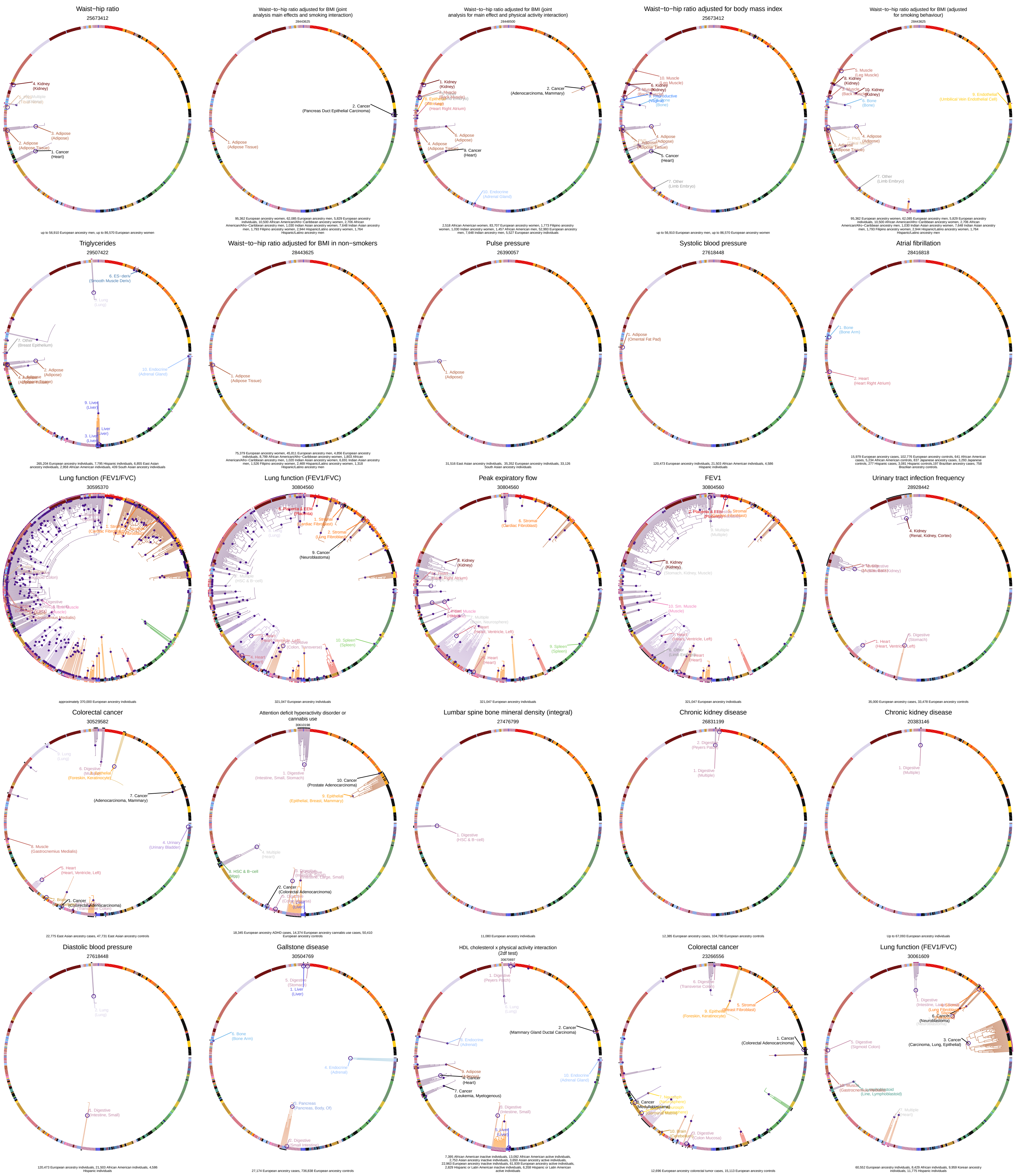

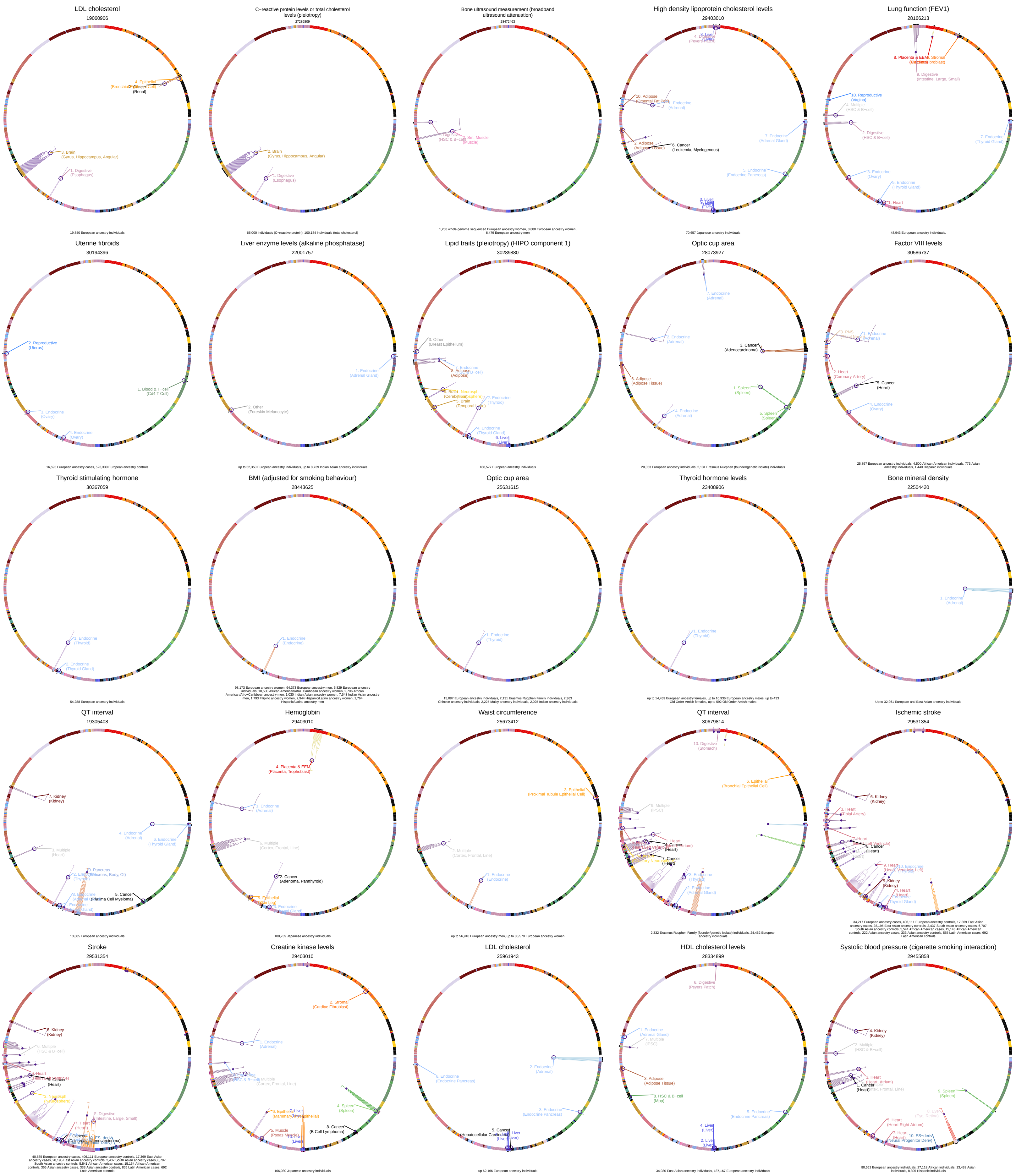

LDL cholesterol
19060906
19,840 European ancestry individuals
C−reactive protein levels or total cholesterol levels (pleiotropy)
27286809
65,000 individuals (C−reactive protein), 100,184 individuals (total cholesterol)
Bone ultrasound measurement (broadband ultrasound attenuation)
28472463
1,268 whole genome sequenced European ancestry women, 8,880 European ancestry women, 6,479 European ancestry men
High density lipoprotein cholesterol levels
29403010
70,657 Japanese ancestry individuals
Lung function (FEV1)
28166213
48,943 European ancestry individuals
Uterine fibroids
30194396
16,595 European ancestry cases, 523,330 European ancestry controls
Liver enzyme levels (alkaline phosphatase)
22001757
Up to 52,350 European ancestry individuals, up to 8,739 Indian Asian ancestry individuals
Lipid traits (pleiotropy) (HIPO component 1)
30289880
188,577 European ancestry individuals
Optic cup area
28073927
20,353 European ancestry individuals, 2,131 Erasmus Rucphen (founder/genetic isolate) individuals
Factor VIII levels
30586737
25,897 European ancestry individuals, 4,500 African American individuals, 773 Asian ancestry individuals, 1,440 Hispanic individuals
Thyroid stimulating hormone
30367059
54,288 European ancestry individuals
BMI (adjusted for smoking behaviour)
28443625
98,173 European ancestry women, 64,373 European ancestry men, 5,829 European ancestry individuals, 10,500 African American/Afro−Caribbean ancestry women, 2,706 African American/Afro−Caribbean ancestry men, 1,030 Indian Asian ancestry women, 7,648 Indian Asian ancestry men, 1,793 Filipino ancestry women, 2,944 Hispanic/Latino ancestry women, 1,764 Hispanic/Latino ancestry men
Optic cup area
25631615
15,087 European ancestry individuals, 2,131 Erasmus Rucphen Family individuals, 2,363 Chinese ancestry individuals, 2,225 Malay ancestry individuals, 2,025 Indian ancestry individuals
Thyroid hormone levels
23408906
up to 14,459 European ancestry females, up to 10,936 European ancestry males, up to 433 Old Order Amish females, up to 592 Old Order Amish males
Bone mineral density
22504420
Up to 32,961 European and East Asian ancestry individuals
QT interval
19305408
13,685 European ancestry individuals
Hemoglobin
29403010
108,769 Japanese ancestry individuals
Waist circumference
25673412
up to 56,910 European ancestry men, up to 86,570 European ancestry women
QT interval
30679814
2,332 Erasmus Rucphen Family (founder/genetic isolate) individuals, 24,462 European ancestry individuals
Ischemic stroke
29531354
34,217 European ancestry cases, 406,111 European ancestry controls, 17,369 East Asian ancestry cases, 28,195 East Asian ancestry controls, 2,437 South Asian ancestry cases, 6,707 South Asian ancestry controls, 5,541 African American cases, 15,146 African American controls, 222 Asian ancestry cases, 333 Asian ancestry controls, 555 Latin American cases, 692 Latin American controls
Stroke
29531354
40,585 European ancestry cases, 406,111 European ancestry controls, 17,369 East Asian ancestry cases, 28,195 East Asian ancestry controls, 2,437 South Asian ancestry cases, 6,707 South Asian ancestry controls, 5,541 African American cases, 15,154 African American controls, 365 Asian ancestry cases, 333 Asian ancestry controls, 865 Latin American cases, 692 Latin American controls
Creatine kinase levels
29403010
106,080 Japanese ancestry individuals
LDL cholesterol
25961943
up to 62,166 European ancestry individuals
HDL cholesterol levels
28334899
34,930 East Asian ancestry individuals, 187,167 European ancestry individuals
Systolic blood pressure (cigarette smoking interaction)
29455858
80,552 European ancestry individuals, 27,118 African individuals, 13,438 Asian individuals, 8,805 Hispanic individuals

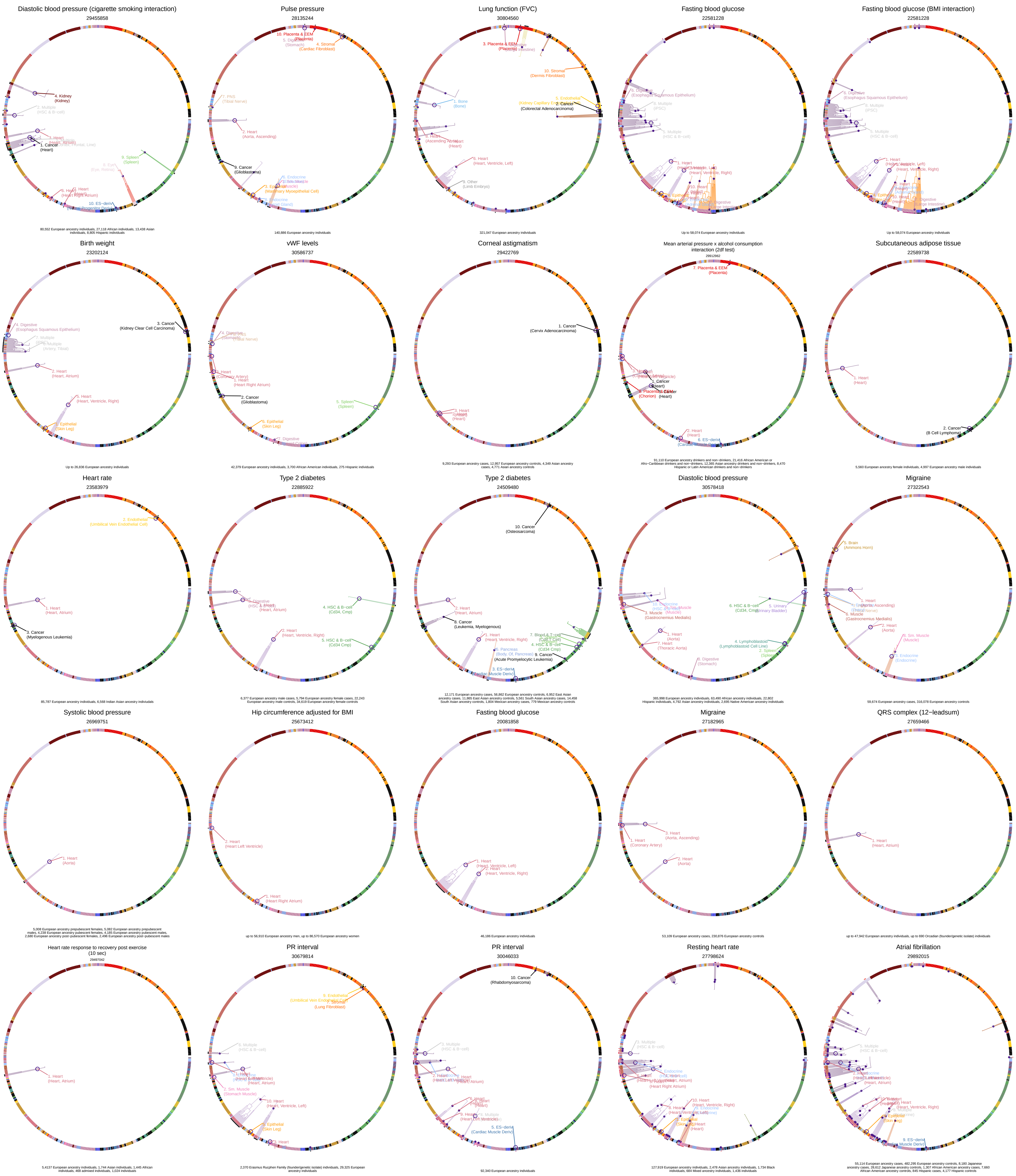
Diastolic blood pressure (cigarette smoking interaction)
29455858
80,552 European ancestry individuals, 27,118 African individuals, 13,438 Asian individuals, 8,805 Hispanic individuals
Pulse pressure
28135244
140,886 European ancestry individuals
Lung function (FVC)
30804560
321,047 European ancestry individuals
Fasting blood glucose
22581228
Up to 58,074 European ancestry individuals
Fasting blood glucose (BMI interaction)
22581228
Up to 58,074 European ancestry individuals
Birth weight
23202124
Up to 26,836 European ancestry individuals
vWF levels
30586737
42,379 European ancestry individuals, 3,700 African American individuals, 275 Hispanic individuals
Corneal astigmatism
29422769
9,293 European ancestry cases, 12,957 European ancestry controls, 4,349 Asian ancestry cases, 4,771 Asian ancestry controls
Mean arterial pressure x alcohol consumption interaction (2df test)
29912962
91,110 European ancestry drinkers and non−drinkers, 21,416 African American or Afro−Caribbean drinkers and non−drinkers, 12,365 Asian ancestry drinkers and non−drinkers, 8,470 Hispanic or Latin American drinkers and non−drinkers
Subcutaneous adipose tissue
22589738
5,560 European ancestry female individuals, 4,997 European ancestry male individuals
Heart rate
23583979
85,787 European ancestry individuals, 6,568 Indian Asian ancestry individuals
Type 2 diabetes
22885922
6,377 European ancestry male cases, 5,794 European ancestry female cases, 22,243 European ancestry male controls, 34,619 European ancestry female controls
Type 2 diabetes
24509480
12,171 European ancestry cases, 56,862 European ancestry controls, 6,952 East Asian ancestry cases, 11,865 East Asian ancestry controls, 5,561 South Asian ancestry cases, 14,458 South Asian ancestry controls, 1,804 Mexican ancestry cases, 779 Mexican ancestry controls
Diastolic blood pressure
30578418
365,988 European ancestry individuals, 63,490 African ancestry individuals, 22,802 Hispanic individuals, 4,792 Asian ancestry individuals, 2,695 Native American ancestry individuals
Migraine
27322543
59,674 European ancestry cases, 316,078 European ancestry controls
Systolic blood pressure
26969751
5,008 European ancestry prepubescent females, 5,082 European ancestry prepubescent males, 4,238 European ancestry pubescent females, 4,185 European ancestry pubescent males, 2,680 European ancestry post−pubescent females, 2,496 European ancestry post−pubescent males
Hip circumference adjusted for BMI
25673412
up to 56,910 European ancestry men, up to 86,570 European ancestry women
Fasting blood glucose
20081858
46,186 European ancestry individuals
Migraine
27182965
53,109 European ancestry cases, 230,876 European ancestry controls
QRS complex (12−leadsum)
27659466
up to 47,942 European ancestry individuals, up to 690 Orcadian (founder/genetic isolate) individuals
Heart rate response to recovery post exercise (10 sec)
29497042
5,4137 European ancestry individuals, 1,744 Asian individuals, 1,445 African individuals, 468 admixed individuals, 1,024 individuals
PR interval
30679814
2,370 Erasmus Rucphen Family (founder/genetic isolate) individuals, 29,325 European ancestry individuals
PR interval
30046033
92,340 European ancestry individuals
Resting heart rate
27798624
127,919 European ancestry individuals, 2,478 Asian ancestry individuals, 1,734 Black individuals, 684 Mixed ancestry individuals, 1,436 individuals
Atrial fibrillation
29892015
55,114 European ancestry cases, 482,295 European ancestry controls, 8,180 Japanese ancestry cases, 28,612 Japanese ancestry controls, 1,307 African American ancestry cases, 7,660 African American ancestry controls, 845 Hispanic cases, 4,177 Hispanic controls

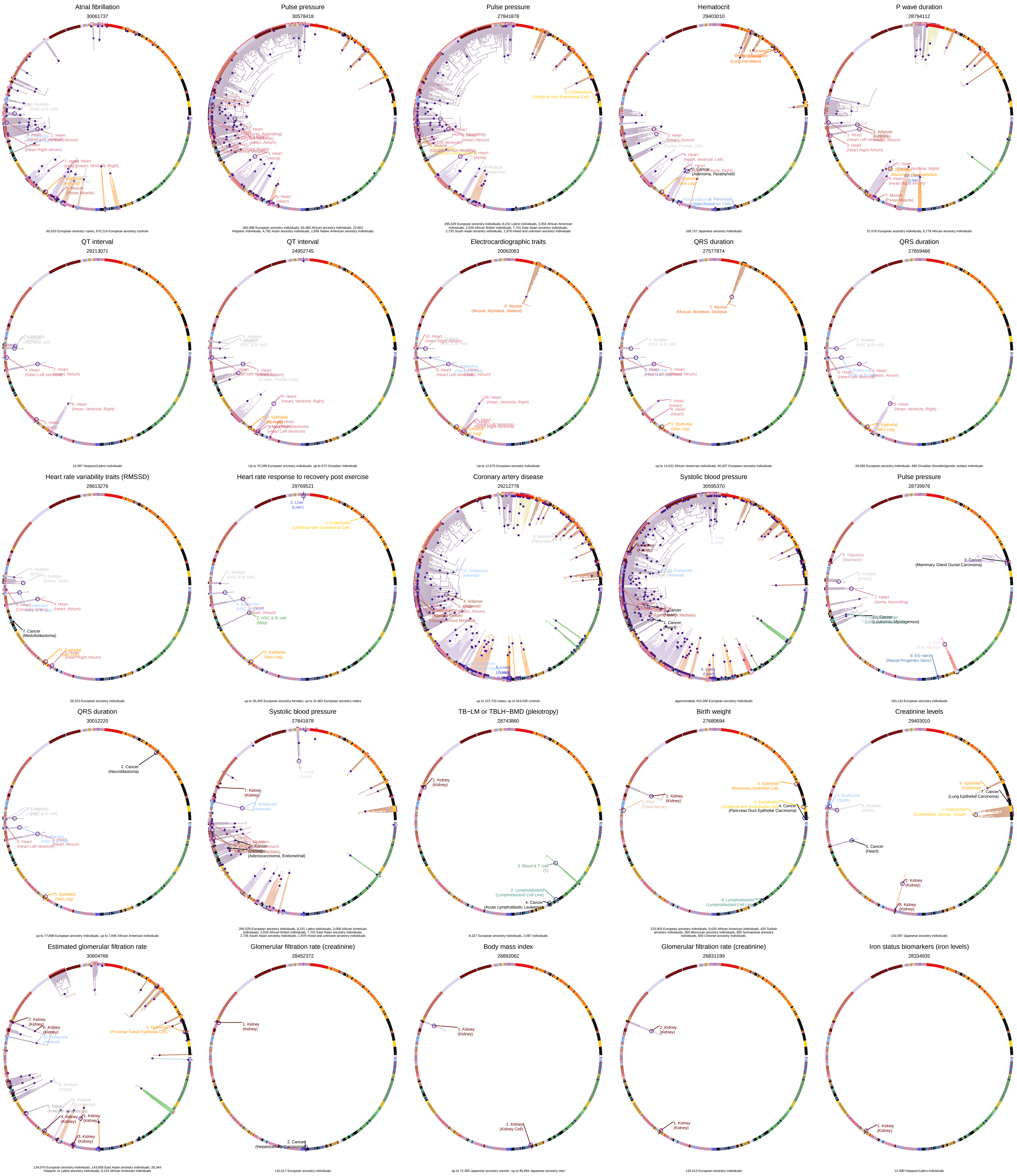

Atrial fibrillation
30061737
60,620 European ancestry cases, 970,216 European ancestry controls
Pulse pressure
30578418
365,998 European ancestry individuals, 63,490 African ancestry individuals, 22,802 Hispanic individuals, 4,792 Asian ancestry individuals, 2,695 Native American ancestry individuals
Pulse pressure
27841878
295,529 European ancestry individuals, 8,231 Latino individuals, 3,058 African American individuals, 2,029 African British individuals, 7,701 East Asian ancestry individuals, 2,735 South Asian ancestry individuals, 1,979 mixed and unknown ancestry individuals
Hematocrit
29403010
108,757 Japanese ancestry individuals
P wave duration
28794112
37,678 European ancestry individuals, 6,778 African ancestry individuals
QT interval
29213071
15,997 Hispanic/Latino individuals
QT interval
24952745
Up to 70,389 European ancestry individuals, up to 672 Orcadian individuals
Electrocardiographic traits
20062063
Up to 12,670 European ancestry individuals
QRS duration
27577874
up to 13,031 African American individuals, 40,407 European ancestry individuals
QRS duration
27659466
59,565 European ancestry individuals, 690 Orcadian (founder/genetic isolate) individuals
Heart rate variability traits (RMSSD)
28613276
26,523 European ancestry individuals
Heart rate response to recovery post exercise
29769521
up to 35,455 European ancestry females, up to 31,802 European ancestry males
Coronary artery disease
29212778
up to 122,733 cases, up to 424,528 controls
Systolic blood pressure
30595370
approximately 422,000 European ancestry individuals
Pulse pressure
28739976
150,134 European ancestry individuals
QRS duration
30012220
up to 77,898 European ancestry individuals, up to 7,695 African American individuals
Systolic blood pressure
27841878
295,529 European ancestry individuals, 8,231 Latino individuals, 3,058 African American individuals, 2,029 African British individuals, 7,701 East Asian ancestry individuals, 2,735 South Asian ancestry individuals, 1,979 mixed and unknown ancestry individuals
TB−LM or TBLH−BMD (pleiotropy)
28743860
8,327 European ancestry individuals, 2,087 individuals
Birth weight
27680694
133,903 European ancestry individuals, 6,635 African American individuals, 420 Turkish ancestry individuals, 365 Moroccan ancestry individuals, 395 Surinamese ancestry individuals, 840 Chinese ancestry individuals
Creatinine levels
29403010
142,097 Japanese ancestry individuals
Estimated glomerular filtration rate
30604766
134,070 European ancestry individuals, 143,658 East Asian ancestry individuals, 26,344 Hispanic or Latino ancestry individuals, 8,224 African American individuals
Glomerular filtration rate (creatinine)
28452372
110,517 European ancestry individuals
Body mass index
28892062
up to 72,390 Japanese ancestry women, up to 85,894 Japanese ancestry men
Glomerular filtration rate (creatinine)
26831199
133,413 European ancestry individuals
Iron status biomarkers (iron levels)
28334935
12,580 Hispanic/Latino individuals

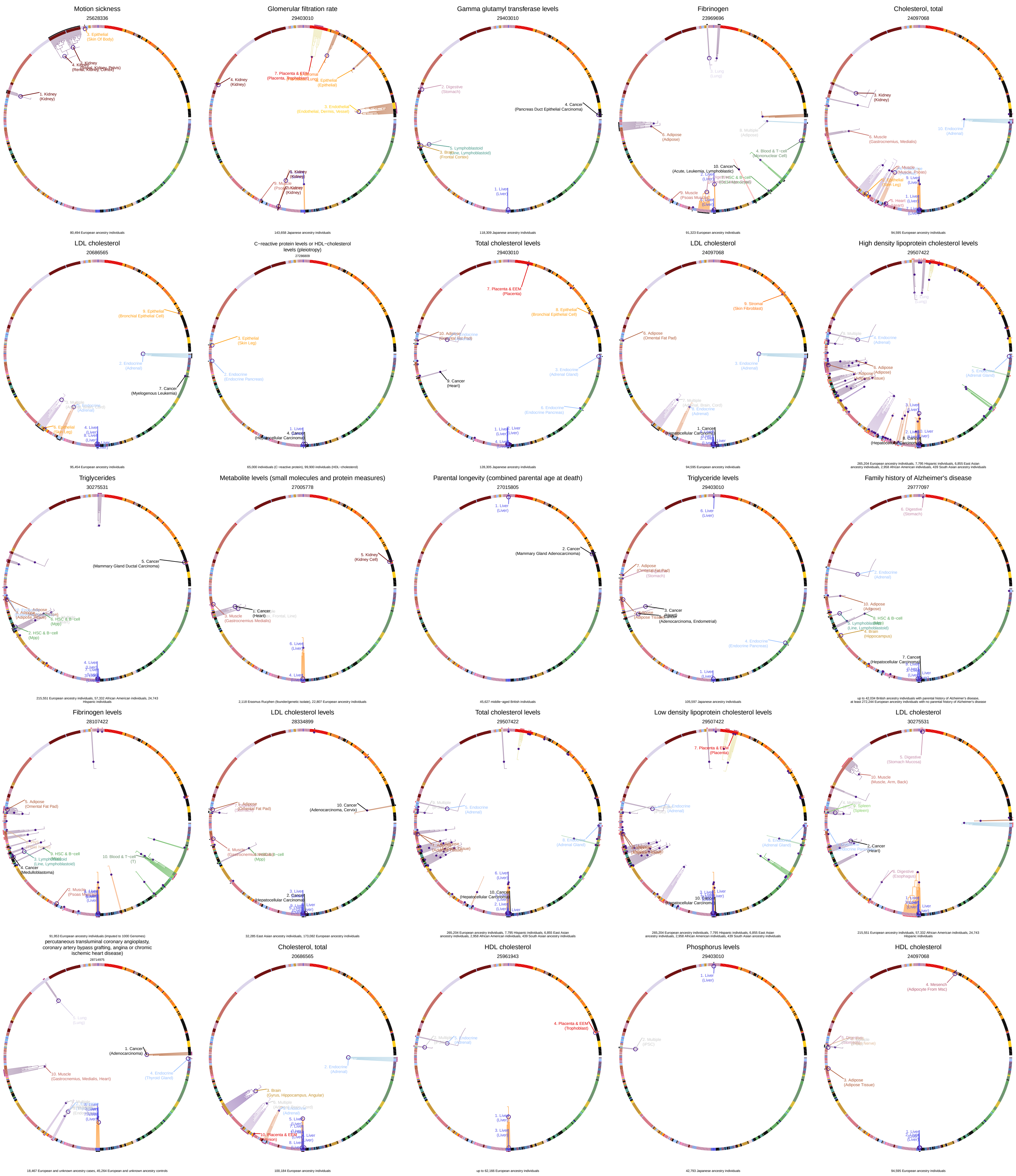

Motion sickness
25628336
80,494 European ancestry individuals
Glomerular filtration rate
29403010
143,658 Japanese ancestry individuals
Gamma glutamyl transferase levels
29403010
118,309 Japanese ancestry individuals
Fibrinogen
23969696
91,323 European ancestry individuals
Cholesterol, total
24097068
94,595 European ancestry individuals
LDL cholesterol
20686565
95,454 European ancestry individuals
C−reactive protein levels or HDL−cholesterol levels (pleiotropy)
27286809
65,000 individuals (C−reactive protein), 99,900 individuals (HDL−cholesterol)
Total cholesterol levels
29403010
128,305 Japanese ancestry individuals
LDL cholesterol
24097068
94,595 European ancestry individuals
High density lipoprotein cholesterol levels
29507422
265,204 European ancestry individuals, 7,795 Hispanic individuals, 6,855 East Asian ancestry individuals, 2,958 African American individuals, 439 South Asian ancestry individuals
Triglycerides
30275531
215,551 European ancestry individuals, 57,332 African American individuals, 24,743 Hispanic individuals
Metabolite levels (small molecules and protein measures)
27005778
2,118 Erasmus Rucphen (founder/genetic isolate), 22,807 European ancestry individuals
Parental longevity (combined parental age at death)
27015805
45,627 middle−aged British individuals
Triglyceride levels
29403010
105,597 Japanese ancestry individuals
Family history of Alzheimer's disease
29777097
up to 42,034 British ancestry individuals with parental history of Alzheimer's disease, at least 272,244 European ancestry individuals with no parental history of Alzheimer's disease
Fibrinogen levels
28107422
91,953 European ancestry individuals (imputed to 1000 Genomes)
LDL cholesterol levels
28334899
32,285 East Asian ancestry individuals, 173,082 European ancestry individuals
Total cholesterol levels
29507422
265,204 European ancestry individuals, 7,795 Hispanic individuals, 6,855 East Asian ancestry individuals, 2,958 African American individuals, 439 South Asian ancestry individuals
Low density lipoprotein cholesterol levels
29507422
265,204 European ancestry individuals, 7,795 Hispanic individuals, 6,855 East Asian ancestry individuals, 2,958 African American individuals, 439 South Asian ancestry individuals
LDL cholesterol
30275531
215,551 European ancestry individuals, 57,332 African American individuals, 24,743 Hispanic individuals
percutaneous transluminal coronary angioplasty, coronary artery bypass grafting, angina or chromic ischemic heart disease)
28714975
18,467 European and unknown ancestry cases, 45,264 European and unknown ancestry controls
Cholesterol, total
20686565
100,184 European ancestry individuals
HDL cholesterol
25961943
up to 62,166 European ancestry individuals
Phosphorus levels
29403010
42,793 Japanese ancestry individuals
HDL cholesterol
24097068
94,595 European ancestry individuals

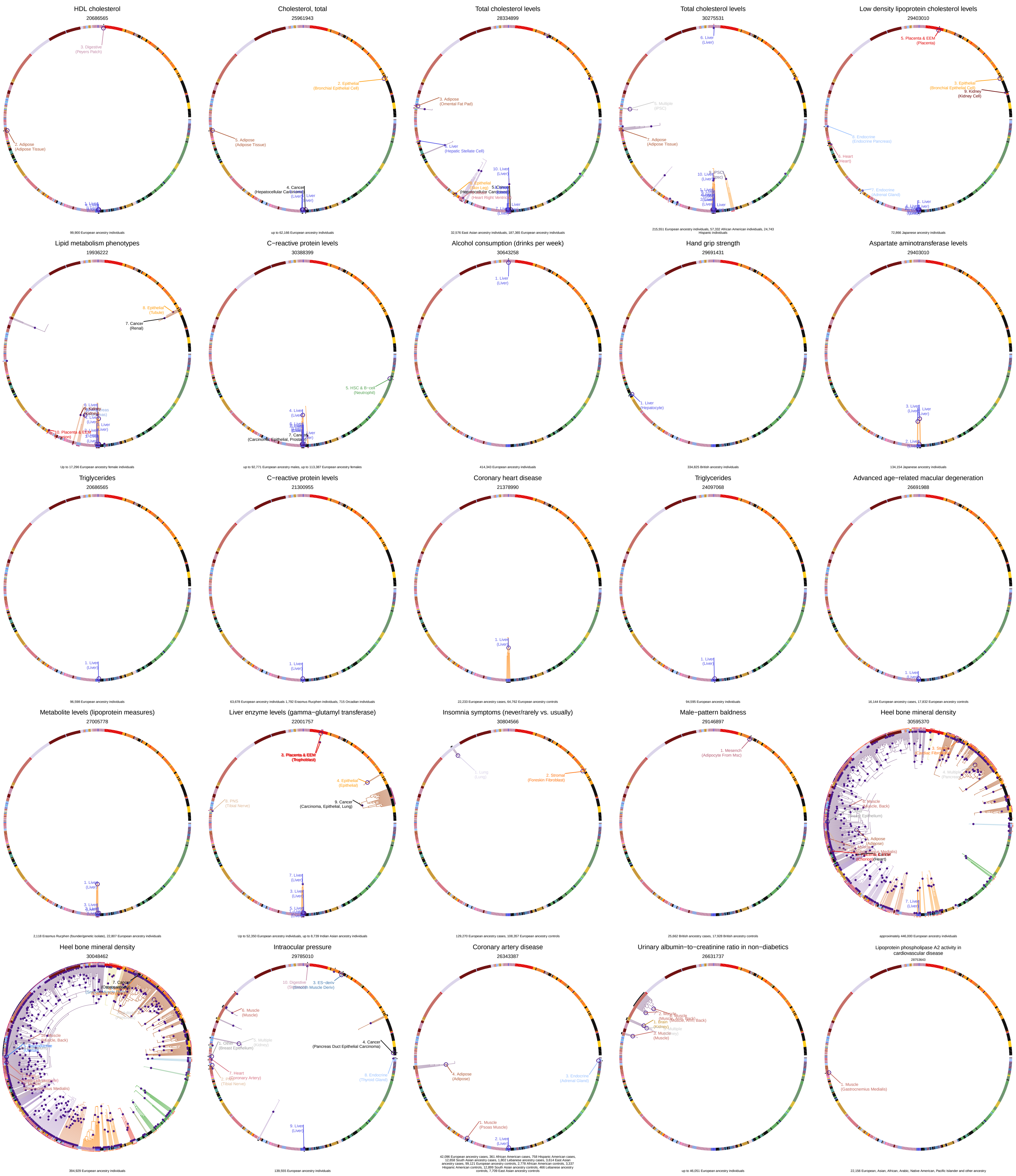

HDL cholesterol
20686565
3. Digestive (Peyers Patch)
2. Adipose (Adipose Tissue)
99,900 European ancestry individuals
Cholesterol, total
25961943
2. Epithelial (Bronchial Epithelial Cell)
5. Adipose (Adipose Tissue)
4. Cancer (Hepatocellular Carcinoma)
up to 62,166 European ancestry individuals
Total cholesterol levels
28334899
3. Adipose (Omental Fat Pad)
6. Liver (Hepatic Stellate Cell)
32,576 East Asian ancestry individuals, 187,365 European ancestry individuals
Total cholesterol levels
30275531
6. Liver (Liver)
7. Adipose (Adipose Tissue)
215,551 European ancestry individuals, 57,332 African American individuals, 24,743 Hispanic individuals
Low density lipoprotein cholesterol levels
29403010
5. Placenta & EEM (Placenta)
3. Epithelial (Bronchial Epithelial Cell)
9. Kidney (Kidney Cell)
8. Endocrine (Endocrine Pancreas)
6. Heart (Heart)
7. Endocrine (Adrenal Gland)
72,866 Japanese ancestry individuals
Lipid metabolism phenotypes
19936222
8. Epithelial (Tubule)
7. Cancer (Renal)
10. Placenta & EEM
Up to 17,296 European ancestry female individuals
C−reactive protein levels
30388399
5. HSC & B−cell (Neutrophil)
4. Liver (Liver)
up to 92,771 European ancestry males, up to 113,387 European ancestry females
Alcohol consumption (drinks per week)
30643258
1. Liver (Liver)
414,343 European ancestry individuals
Hand grip strength
29691431
1. Liver (Hepatocyte)
334,825 British ancestry individuals
Aspartate aminotransferase levels
29403010
3. Liver (Liver)
2. Liver (Liver)
134,154 Japanese ancestry individuals
Triglycerides
20686565
1. Liver (Liver)
96,598 European ancestry individuals
C−reactive protein levels
21300955
1. Liver (Liver)
63,678 European ancestry individuals 1,792 Erasmus Rucphen individuals, 715 Orcadian individuals
Coronary heart disease
21378990
1. Liver (Liver)
22,233 European ancestry cases, 64,762 European ancestry controls
Triglycerides
24097068
1. Liver (Liver)
94,595 European ancestry individuals
Advanced age−related macular degeneration
26691988
1. Liver (Liver)
16,144 European ancestry cases, 17,832 European ancestry controls
Metabolite levels (lipoprotein measures)
27005778
1. Liver (Liver)
2,118 Erasmus Rucphen (founder/genetic isolate), 22,807 European ancestry individuals
Liver enzyme levels (gamma−glutamyl transferase)
22001757
2. Placenta & EEM (Trophoblast)
4. Epithelial (Epithelial)
8. PNS (Tibial Nerve)
9. Cancer (Carcinoma, Epithelial, Lung)
7. Liver (Liver)
3. Liver (Liver)
Up to 52,350 European ancestry individuals, up to 8,739 Indian Asian ancestry individuals
Insomnia symptoms (never/rarely vs. usually)
30804566
1. Lung (Lung)
2. Stromal (Foreskin Fibroblast)
129,270 European ancestry cases, 108,357 European ancestry controls
Male−pattern baldness
29146897
1. Mesench (Adipocyte From Msc)
25,662 British ancestry cases, 17,928 British ancestry controls
Heel bone mineral density
30595370
7. Liver (Liver)
approximately 446,000 European ancestry individuals
Heel bone mineral density
30048462
394,929 European ancestry individuals
Intraocular pressure
29785010
3. ES−deriv (Smooth Muscle Deriv)
6. Muscle (Muscle)
5. Multiple (Kidney)
4. Cancer (Pancreas Duct Epithelial Carcinoma)
7. Heart (Coronary Artery)
8. Endocrine (Thyroid Gland)
9. Liver (Liver)
139,555 European ancestry individuals
Coronary artery disease
26343387
4. Adipose (Adipose)
3. Endocrine (Adrenal Gland)
1. Muscle (Psoas Muscle)
2. Liver (Liver)
42,096 European ancestry cases, 361 African American cases, 758 Hispanic American cases, 12,658 South Asian ancestry cases, 1,802 Lebanese ancestry cases, 3,614 East Asian ancestry cases, 99,121 European ancestry controls, 2,778 African American controls, 3,337 Hispanic American controls, 12,899 South Asian ancestry controls, 466 Lebanese ancestry controls, 7,709 East Asian ancestry controls
Urinary albumin−to−creatinine ratio in non−diabetics
26631737
1. Brain (Kidney)
3. Muscle (Muscle)
up to 46,051 European ancestry individuals
Lipoprotein phospholipase A2 activity in cardiovascular disease
28753643
1. Muscle (Gastrocnemius Medialis)
22,156 European, Asian, African, Arabic, Native American, Pacific Islander and other ancestry

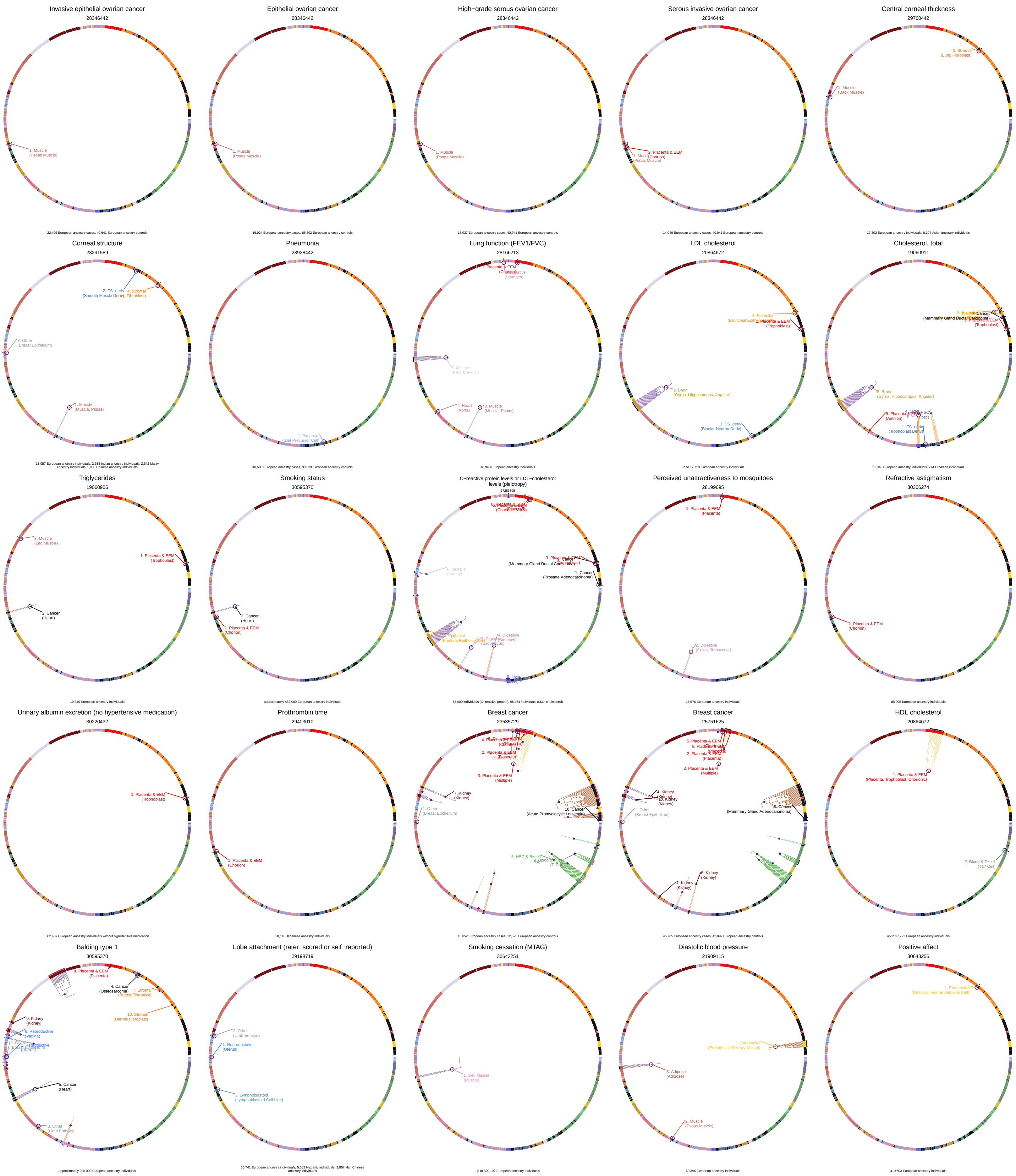

Invasive epithelial ovarian cancer
28346442
22,406 European ancestry cases, 40,941 European ancestry controls
Epithelial ovarian cancer
28346442
16,924 European ancestry cases, 68,502 European ancestry controls
High−grade serous ovarian cancer
28346442
13,037 European ancestry cases, 40,941 European ancestry controls
Serous invasive ovarian cancer
28346442
14,049 European ancestry cases, 40,941 European ancestry controls
Central corneal thickness
29760442
17,803 European ancestry individuals, 8,107 Asian ancestry individuals
Corneal structure
23291589
13,057 European ancestry individuals, 2,538 Indian ancestry individuals, 2,542 Malay ancestry individuals, 1,883 Chinese ancestry individuals
Pneumonia
28928442
40,600 European ancestry cases, 90,039 European ancestry controls
Lung function (FEV1/FVC)
28166213
48,943 European ancestry individuals
LDL cholesterol
20864672
up to 17,723 European ancestry individuals
Cholesterol, total
19060911
21,848 European ancestry individuals, 714 Orcadian individuals
Triglycerides
19060906
19,840 European ancestry individuals
Smoking status
30595370
approximately 458,000 European ancestry individuals
C−reactive protein levels or LDL−cholesterol levels (pleiotropy)
27286809
65,000 individuals (C−reactive protein), 95,454 individuals (LDL−cholesterol)
Perceived unattractiveness to mosquitoes
28199695
16,576 European ancestry individuals
Refractive astigmatism
30306274
89,005 European ancestry individuals
Urinary albumin excretion (no hypertensive medication)
30220432
302,687 European ancestry individuals without hypertensive medication
Prothrombin time
29403010
58,110 Japanese ancestry individuals
Breast cancer
23535729
10,052 European ancestry cases, 12,575 European ancestry controls
Breast cancer
25751625
46,785 European ancestry cases, 42,892 European ancestry controls
HDL cholesterol
20864672
up to 17,723 European ancestry individuals
Balding type 1
30595370
approximately 208,000 European ancestry individuals
Lobe attachment (rater−scored or self−reported)
29198719
66,741 European ancestry individuals, 5,062 Hispanic individuals, 2,857 Han Chinese ancestry individuals
Smoking cessation (MTAG)
30643251
up to 820,192 European ancestry individuals
Diastolic blood pressure
21909115
69,395 European ancestry individuals
Positive affect
30643256
410,603 European ancestry individuals

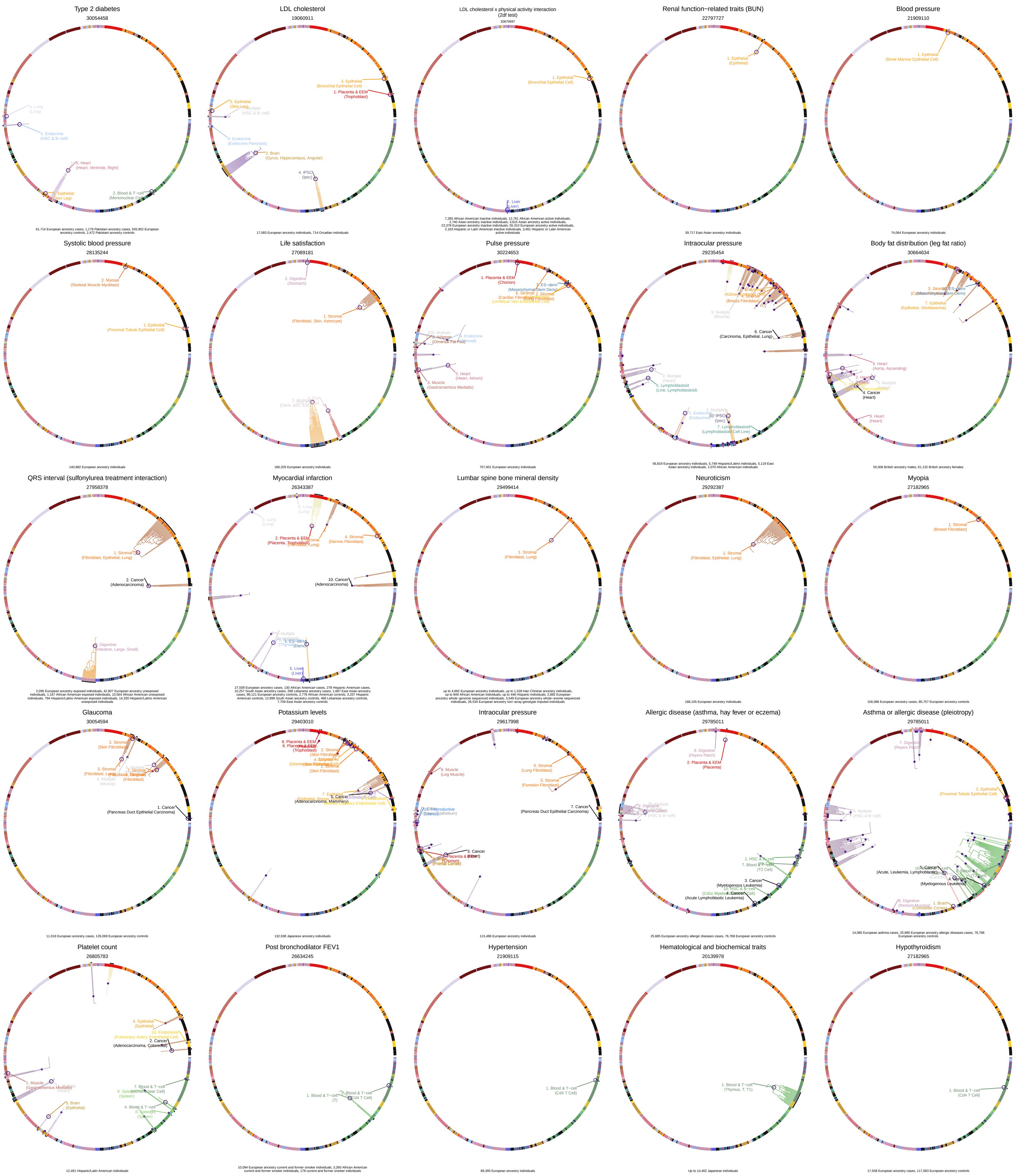

Type 2 diabetes
30054458
61,714 European ancestry cases, 1,178 Pakistani ancestry cases, 593,952 European ancestry controls, 2,472 Pakistani ancestry controls
LDL cholesterol
19060911
17,083 European ancestry individuals, 714 Orcadian individuals
LDL cholesterol x physical activity interaction (2df test)
30670697
7,260 African American inactive individuals, 12,761 African American active individuals, 2,740 Asian ancestry inactive individuals, 3,615 Asian ancestry active individuals, 22,378 European ancestry inactive individuals, 55,310 European ancestry active individuals, 2,163 Hispanic or Latin American inactive individuals, 3,461 Hispanic or Latin American active individuals
Renal function−related traits (BUN)
22797727
39,717 East Asian ancestry individuals
Blood pressure
21909110
74,064 European ancestry individuals
Systolic blood pressure
28135244
140,882 European ancestry individuals
Life satisfaction
27089181
166,205 European ancestry individuals
Pulse pressure
30224653
757,601 European ancestry individuals
Intraocular pressure
29235454
56,819 European ancestry individuals, 5,748 Hispanic/Latino individuals, 5,119 East Asian ancestry individuals, 2,070 African American individuals
Body fat distribution (leg fat ratio)
30664634
55,006 British ancestry males, 61,132 British ancestry females
QRS interval (sulfonylurea treatment interaction)
27958378
2,095 European ancestry exposed individuals, 42,907 European ancestry unexposed individuals, 1,167 African American exposed individuals, 10,564 African American unexposed individuals, 794 Hispanic/Latino American exposed individuals, 14,330 Hispanic/Latino American unexposed individuals
Myocardial infarction
26343387
27,509 European ancestry cases, 130 African American cases, 278 Hispanic American cases, 10,257 South Asian ancestry cases, 288 Lebanese ancestry cases, 1,687 East Asian ancestry cases, 99,121 European ancestry controls, 2,778 African American controls, 3,337 Hispanic American controls, 12,899 South Asian ancestry controls, 466 Lebanese ancestry controls, 7,709 East Asian ancestry controls
Lumbar spine bone mineral density
29499414
up to 4,692 European ancestry individuals, up to 1,539 Han Chinese ancestry individuals, up to 849 African American individuals, up to 448 Hispanic individuals, 2,882 European ancestry whole−genome sequenced individuals, 3,549 European ancestry whole−exome sequenced individuals, 26,534 European ancestry non−array genotype imputed individuals
Neuroticism
29292387
168,105 European ancestry individuals
Myopia
27182965
106,086 European ancestry cases, 85,757 European ancestry controls
Glaucoma
30054594
11,018 European ancestry cases, 126,068 European ancestry controls
Potassium levels
29403010
132,938 Japanese ancestry individuals
Intraocular pressure
29617998
115,486 European ancestry individuals
Allergic disease (asthma, hay fever or eczema)
29785011
25,685 European ancestry allergic diseases cases, 76,768 European ancestry controls
Asthma or allergic disease (pleiotropy)
29785011
14,085 European asthma cases, 25,685 European ancestry allergic diseases cases, 76,768 European ancestry controls
Platelet count
26805783
12,491 Hispanic/Latin American individuals
Post bronchodilator FEV1
26634245
10,094 European ancestry current and former smoker individuals, 3,260 African American current and former smoker individuals, 178 current and former smoker individuals
Hypertension
21909115
69,395 European ancestry individuals
Hematological and biochemical traits
20139978
Up to 14,402 Japanese individuals
Hypothyroidism
27182965
17,558 European ancestry cases, 117,083 European ancestry controls

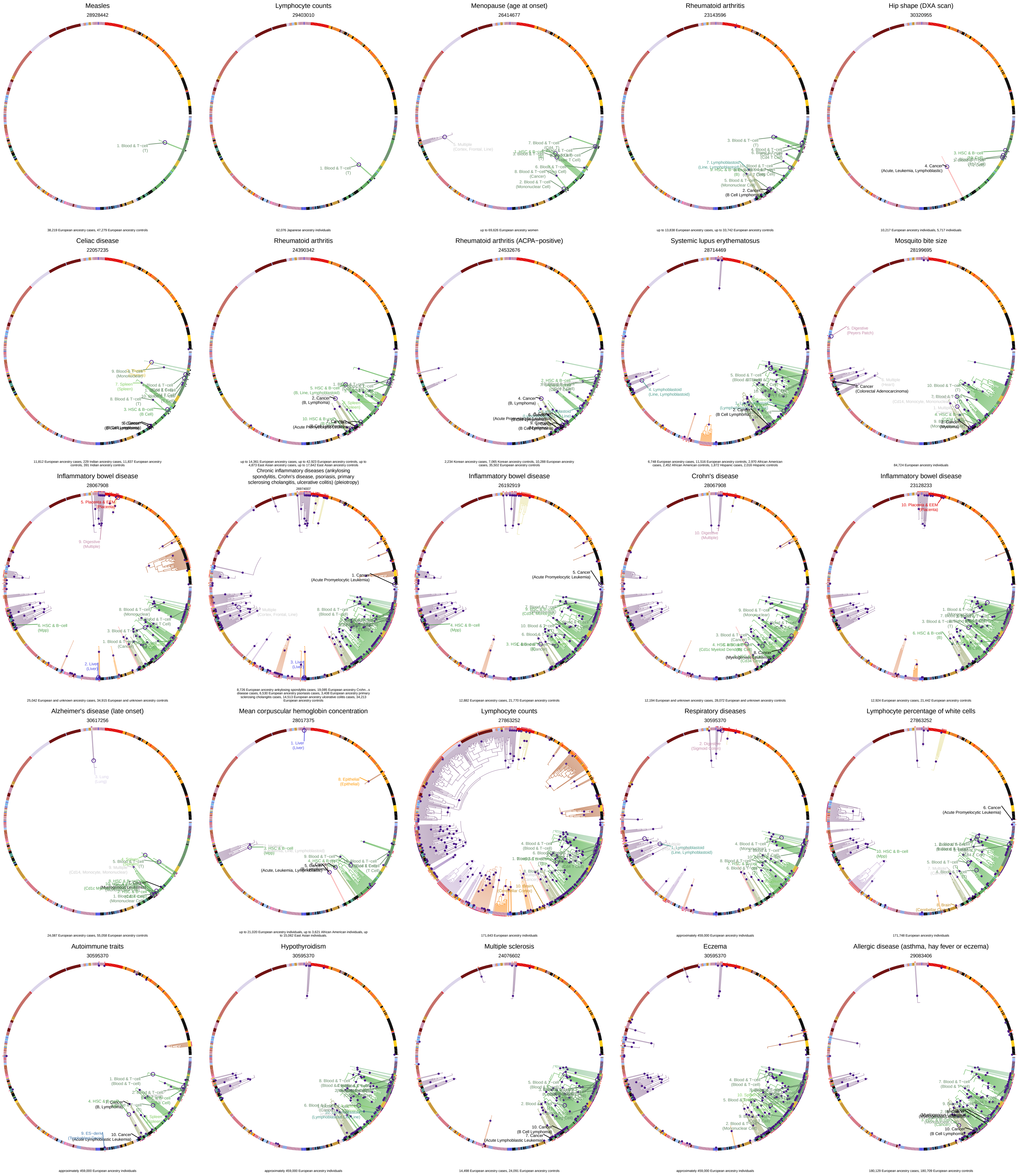

Measles
28928442
38,219 European ancestry cases, 47,279 European ancestry controls
Lymphocyte counts
29403010
62,076 Japanese ancestry individuals
Menopause (age at onset)
26414677
up to 69,626 European ancestry women
Rheumatoid arthritis
23143596
up to 13,838 European ancestry cases, up to 33,742 European ancestry controls
Hip shape (DXA scan)
30320955
10,217 European ancestry individuals, 5,717 individuals
Celiac disease
22057235
11,812 European ancestry cases, 229 Indian ancestry cases, 11,837 European ancestry controls, 391 Indian ancestry controls
Rheumatoid arthritis
24390342
up to 14,361 European ancestry cases, up to 42,923 European ancestry controls, up to 4,873 East Asian ancestry cases, up to 17,642 East Asian ancestry controls
Rheumatoid arthritis (ACPA−positive)
24532676
2,234 Korean ancestry cases, 7,065 Korean ancestry controls, 10,288 European ancestry cases, 35,502 European ancestry controls
Systemic lupus erythematosus
28714469
6,748 European ancestry cases, 11,516 European ancestry controls, 2,970 African American cases, 2,452 African American controls, 1,872 Hispanic cases, 2,016 Hispanic controls
Mosquito bite size
28199695
84,724 European ancestry individuals
Inflammatory bowel disease
28067908
25,042 European and unknown ancestry cases, 34,915 European and unknown ancestry controls
Chronic inflammatory diseases (ankylosing spondylitis, Crohn's disease, psoriasis, primary sclerosing cholangitis, ulcerative colitis) (pleiotropy)
26974007
8,726 European ancestry ankylosing spondylitis cases, 19,085 European ancestry Crohn...s disease cases, 6,530 European ancestry psoriasis cases, 3,408 European ancestry primary sclerosing cholangitis cases, 14,513 European ancestry ulcerative colitis cases, 34,213 European ancestry controls
Inflammatory bowel disease
26192919
12,882 European ancestry cases, 21,770 European ancestry controls
Crohn's disease
28067908
12,194 European and unknown ancestry cases, 28,072 European and unknown ancestry controls
Inflammatory bowel disease
23128233
12,924 European ancestry cases, 21,442 European ancestry controls
Alzheimer's disease (late onset)
30617256
24,087 European ancestry cases, 55,058 European ancestry controls
Mean corpuscular hemoglobin concentration
28017375
up to 21,020 European ancestry individuals, up to 3,621 African American individuals, up to 15,062 East Asian individuals
Lymphocyte counts
27863252
171,643 European ancestry individuals
Respiratory diseases
30595370
approximately 459,000 European ancestry individuals
Lymphocyte percentage of white cells
27863252
171,748 European ancestry individuals
Autoimmune traits
30595370
approximately 459,000 European ancestry individuals
Hypothyroidism
30595370
approximately 459,000 European ancestry individuals
Multiple sclerosis
24076602
14,498 European ancestry cases, 24,091 European ancestry controls
Eczema
30595370
approximately 459,000 European ancestry individuals
Allergic disease (asthma, hay fever or eczema)
29083406
180,129 European ancestry cases, 180,709 European ancestry controls

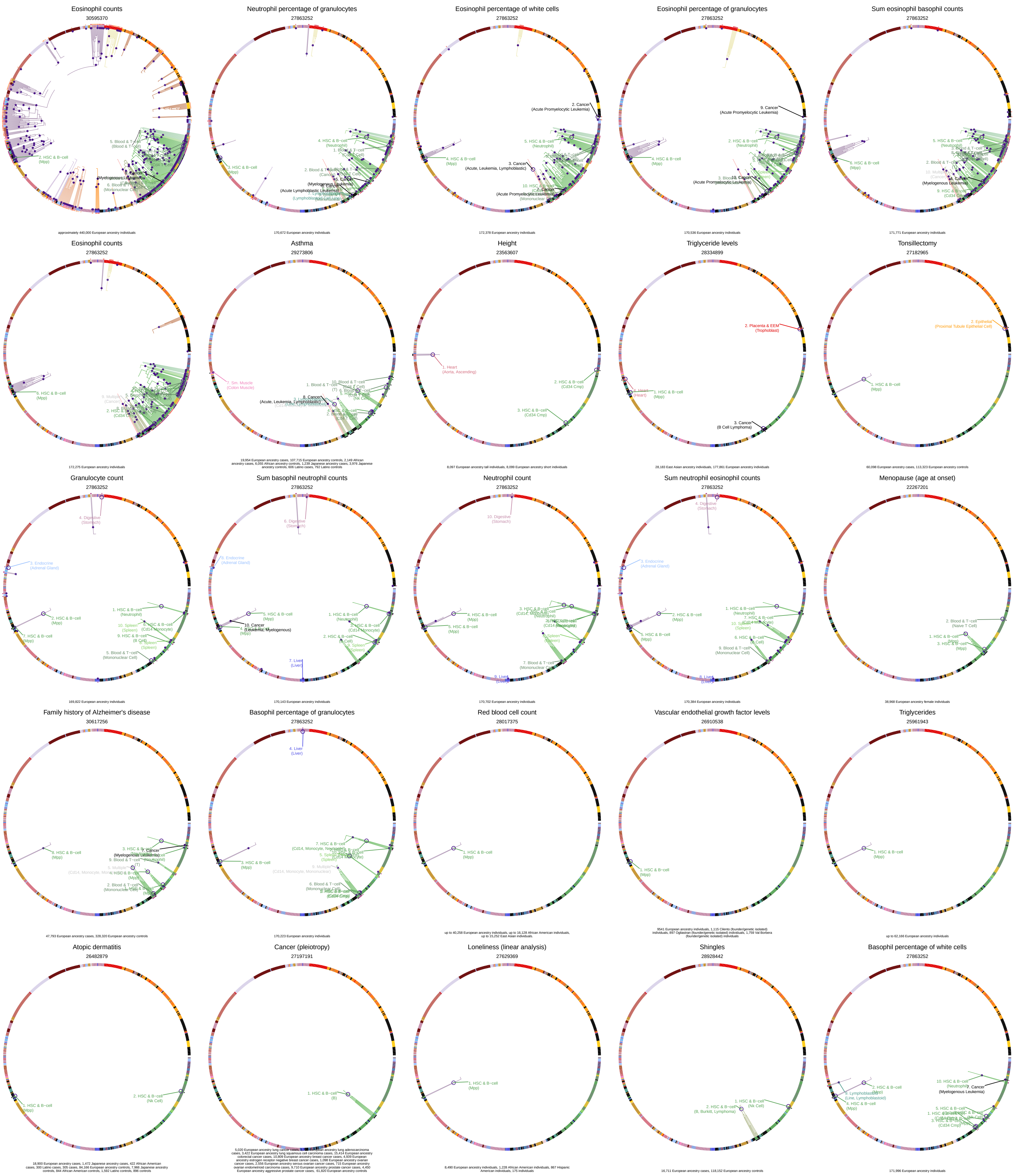

Eosinophil counts
30595370
approximately 440,000 European ancestry individuals
Neutrophil percentage of granulocytes
27863252
170,672 European ancestry individuals
Eosinophil percentage of white cells
27863252
172,378 European ancestry individuals
Eosinophil percentage of granulocytes
27863252
170,536 European ancestry individuals
Sum eosinophil basophil counts
27863252
171,771 European ancestry individuals
Eosinophil counts
27863252
172,275 European ancestry individuals
Asthma
29273806
19,954 European ancestry cases, 107,715 European ancestry controls, 2,149 African ancestry cases, 6,055 African ancestry controls, 1,239 Japanese ancestry cases, 3,976 Japanese ancestry controls, 606 Latino cases, 792 Latino controls
Height
23563607
1. Heart
(Aorta, Ascending)
2. HSC & B−cell
(Cd34 Cmp)
3. HSC & B−cell
(Cd34 Cmp)
8,097 European ancestry tall individuals, 8,099 European ancestry short individuals
Triglyceride levels
28334899
2. Placenta & EEM
(Trophoblast)
1. HSC & B−cell
(Mpp)
4. Heart
(Heart)
3. Cancer
(B Cell Lymphoma)
28,183 East Asian ancestry individuals, 177,861 European ancestry individuals
Tonsillectomy
27182965
2. Epithelial
(Proximal Tubule Epithelial Cell)
1. HSC & B−cell
(Mpp)
60,098 European ancestry cases, 113,323 European ancestry controls
Granulocyte count
27863252
169,822 European ancestry individuals
Sum basophil neutrophil counts
27863252
170,143 European ancestry individuals
Neutrophil count
27863252
170,702 European ancestry individuals
Sum neutrophil eosinophil counts
27863252
170,384 European ancestry individuals
Menopause (age at onset)
22267201
2. Blood & T−cell
(Naive T Cell)
38,968 European ancestry female individuals
Family history of Alzheimer's disease
30617256
47,793 European ancestry cases, 328,320 European ancestry controls
Basophil percentage of granulocytes
27863252
170,223 European ancestry individuals
Red blood cell count
28017375
1. HSC & B−cell
(Mpp)
up to 40,258 European ancestry individuals, up to 16,128 African American individuals, up to 15,252 East Asian individuals.
Vascular endothelial growth factor levels
26910538
1. HSC & B−cell
(Mpp)
9541 European ancestry individuals, 1,115 Cilento (founder/genetic isolated) individuals, 897 Ogliastran (founder/genetic isolated) individuals, 1,759 Val Borbera (founder/genetic isolated) individuals
Triglycerides
25961943
1. HSC & B−cell
(Mpp)
up to 62,166 European ancestry individuals
Atopic dermatitis
26482879
2. HSC & B−cell
(Nk Cell)
1. HSC & B−cell
(Mpp)
18,900 European ancestry cases, 1,472 Japanese ancestry cases, 422 African American cases, 300 Latino cases, 305 cases, 84,166 European ancestry controls, 7,966 Japanese ancestry controls, 844 African American controls, 1,592 Latino controls, 896 controls
Cancer (pleiotropy)
27197191
1. HSC & B−cell
(B)
Loneliness (linear analysis)
27629369
1. HSC & B−cell
(Mpp)
8,490 European ancestry individuals, 1,228 African American individuals, 867 Hispanic American individuals, 175 individuals
Shingles
28928442
1. HSC & B−cell
(Nk Cell)
2. HSC & B−cell
(B, Burkitt, Lymphoma)
16,711 European ancestry cases, 118,152 European ancestry controls
Basophil percentage of white cells
27863252
7. Cancer
(Myelogenous Leukemia)
171,996 European ancestry individuals

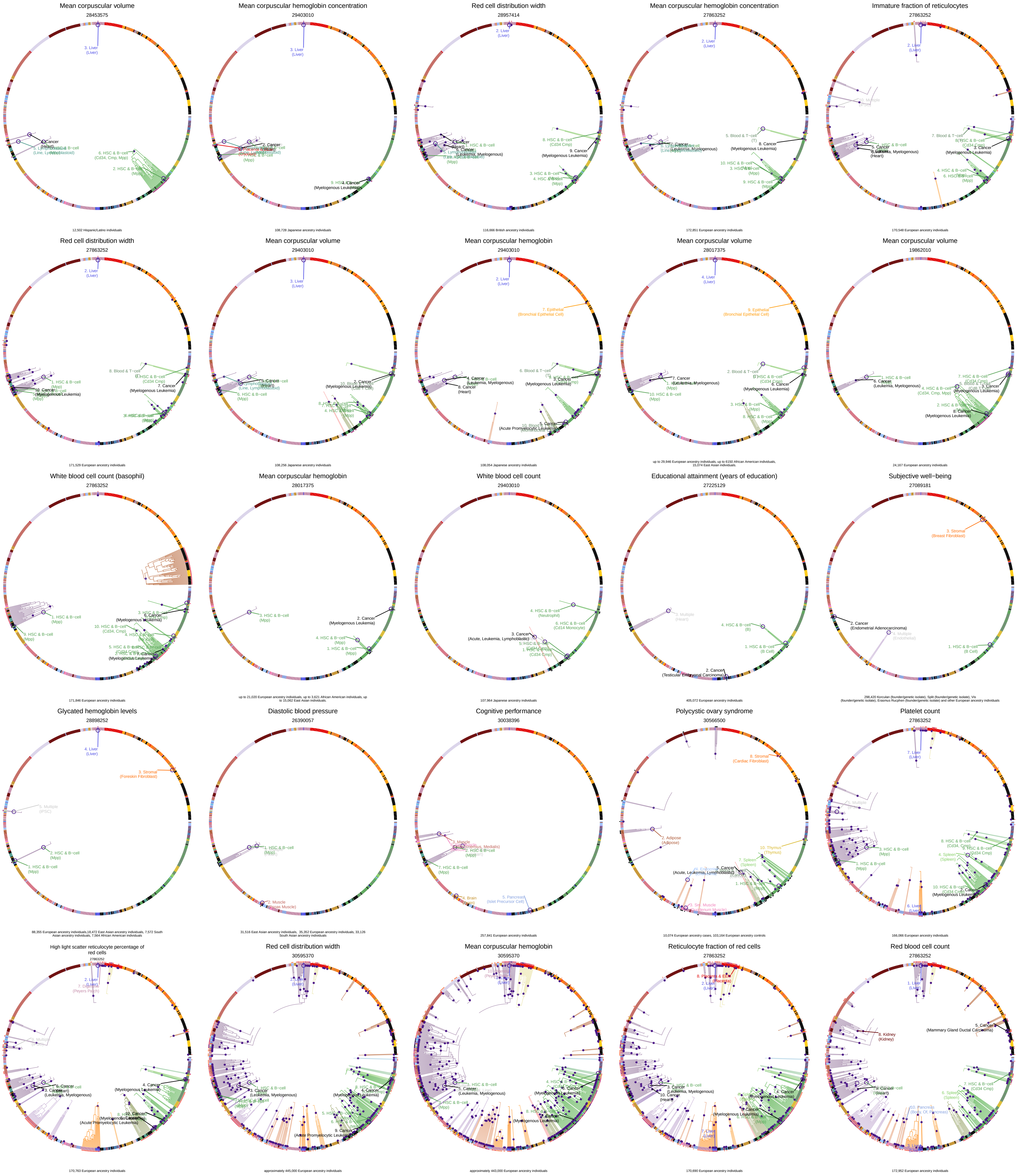

Mean corpuscular volume
28453575
12,502 Hispanic/Latino individuals
Mean corpuscular hemoglobin concentration
29403010
108,728 Japanese ancestry individuals
Red cell distribution width
28957414
116,666 British ancestry individuals
Mean corpuscular hemoglobin concentration
27863252
172,851 European ancestry individuals
Immature fraction of reticulocytes
27863252
170,548 European ancestry individuals
Red cell distribution width
27863252
171,529 European ancestry individuals
Mean corpuscular volume
29403010
108,256 Japanese ancestry individuals
Mean corpuscular hemoglobin
29403010
108,054 Japanese ancestry individuals
Mean corpuscular volume
28017375
up to 29,946 European ancestry individuals, up to 6150 African American individuals, 15,074 East Asian individuals.
Mean corpuscular volume
19862010
24,167 European ancestry individuals
White blood cell count (basophil)
27863252
171,846 European ancestry individuals
Mean corpuscular hemoglobin
28017375
up to 21,020 European ancestry individuals, up to 3,621 African American individuals, up to 15,062 East Asian individuals.
White blood cell count
29403010
107,964 Japanese ancestry individuals
Educational attainment (years of education)
27225129
405,072 European ancestry individuals
Subjective well−being
27089181
298,420 Korculan (founder/genetic isolate), Split (founder/genetic isolate), Vis (founder/genetic isolate), Erasmus Rucphen (founder/genetic isolate) and other European ancestry individuals
Glycated hemoglobin levels
28898252
88,355 European ancestry individuals,18,472 East Asian ancestry individuals, 7,572 South Asian ancestry individuals, 7,564 African American individuals
Diastolic blood pressure
26390057
31,516 East Asian ancestry individuals, 35,352 European ancestry individuals, 33,126 South Asian ancestry individuals
Cognitive performance
30038396
257,841 European ancestry individuals
Polycystic ovary syndrome
30566500
10,074 European ancestry cases, 103,164 European ancestry controls
Platelet count
27863252
166,066 European ancestry individuals
High light scatter reticulocyte percentage of red cells
27863252
170,763 European ancestry individuals
Red cell distribution width
30595370
approximately 445,000 European ancestry individuals
Mean corpuscular hemoglobin
30595370
approximately 443,000 European ancestry individuals
Reticulocyte fraction of red cells
27863252
170,690 European ancestry individuals
Red blood cell count
27863252
172,952 European ancestry individuals

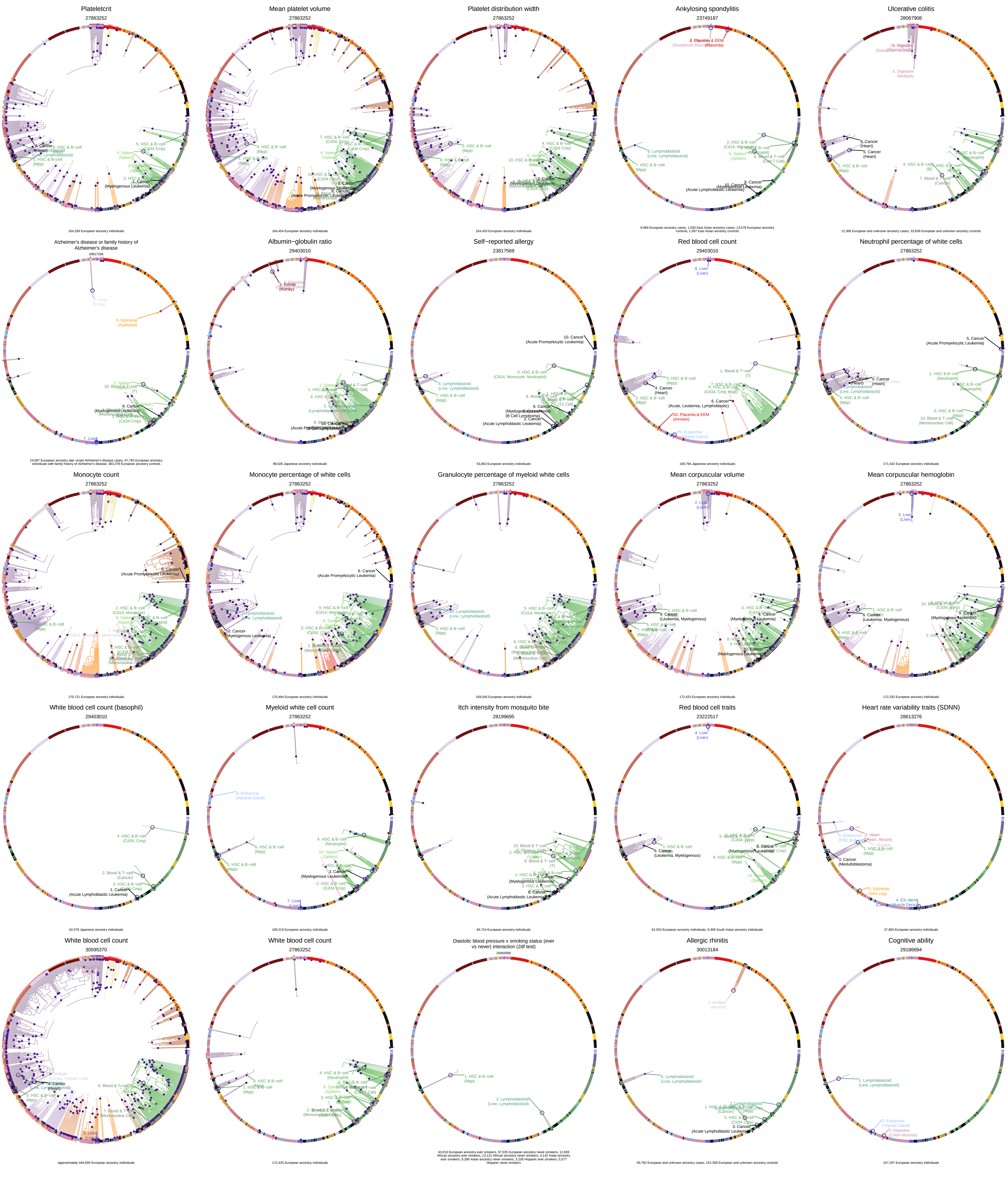

Plateletcrit
27863252
164,339 European ancestry individuals
Mean platelet volume
27863252
164,454 European ancestry individuals
Platelet distribution width
27863252
164,433 European ancestry individuals
Ankylosing spondylitis
23749187
9,069 European ancestry cases, 1,550 East Asian ancestry cases, 13,578 European ancestry controls, 1,567 East Asian ancestry controls
Ulcerative colitis
28067908
12,366 European and unknown ancestry cases, 33,609 European and unknown ancestry controls
Alzheimer's disease or family history of Alzheimer's disease
30617256
24,087 European ancestry late−onset Alzheimer's disease cases, 47,793 European ancestry individuals with family history of Alzheimer's disease, 383,378 European ancestry controls
Albumin−globulin ratio
29403010
98,626 Japanese ancestry individuals
Self−reported allergy
23817569
53,862 European ancestry individuals
Red blood cell count
29403010
108,794 Japanese ancestry individuals
Neutrophil percentage of white cells
27863252
171,542 European ancestry individuals
Monocyte count
27863252
170,721 European ancestry individuals
Monocyte percentage of white cells
27863252
170,494 European ancestry individuals
Granulocyte percentage of myeloid white cells
27863252
169,545 European ancestry individuals
Mean corpuscular volume
27863252
172,433 European ancestry individuals
Mean corpuscular hemoglobin
27863252
172,332 European ancestry individuals
White blood cell count (basophil)
29403010
62,076 Japanese ancestry individuals
Myeloid white cell count
27863252
169,219 European ancestry individuals
Itch intensity from mosquito bite
28199695
84,724 European ancestry individuals
Red blood cell traits
23222517
62,553 European ancestry individuals, 9,308 South Asian ancestry individuals
Heart rate variability traits (SDNN)
28613276
27,850 European ancestry individuals
White blood cell count
30595370
approximately 444,000 European ancestry individuals
White blood cell count
27863252
172,435 European ancestry individuals
Diastolic blood pressure x smoking status (ever vs never) interaction (2df test)
29455858
43,016 European ancestry ever smokers, 37,535 European ancestry never smokers, 12,693 African ancestry ever smokers, 13,121 African ancestry never smokers, 4,142 Asian ancestry ever smokers, 9,296 Asian ancestry never smokers, 3,228 Hispanic ever smokers, 5,577 Hispanic never smokers
Allergic rhinitis
30013184
59,762 European and unknown ancestry cases, 152,358 European and unknown ancestry controls
Cognitive ability
29186694
107,207 European ancestry individuals

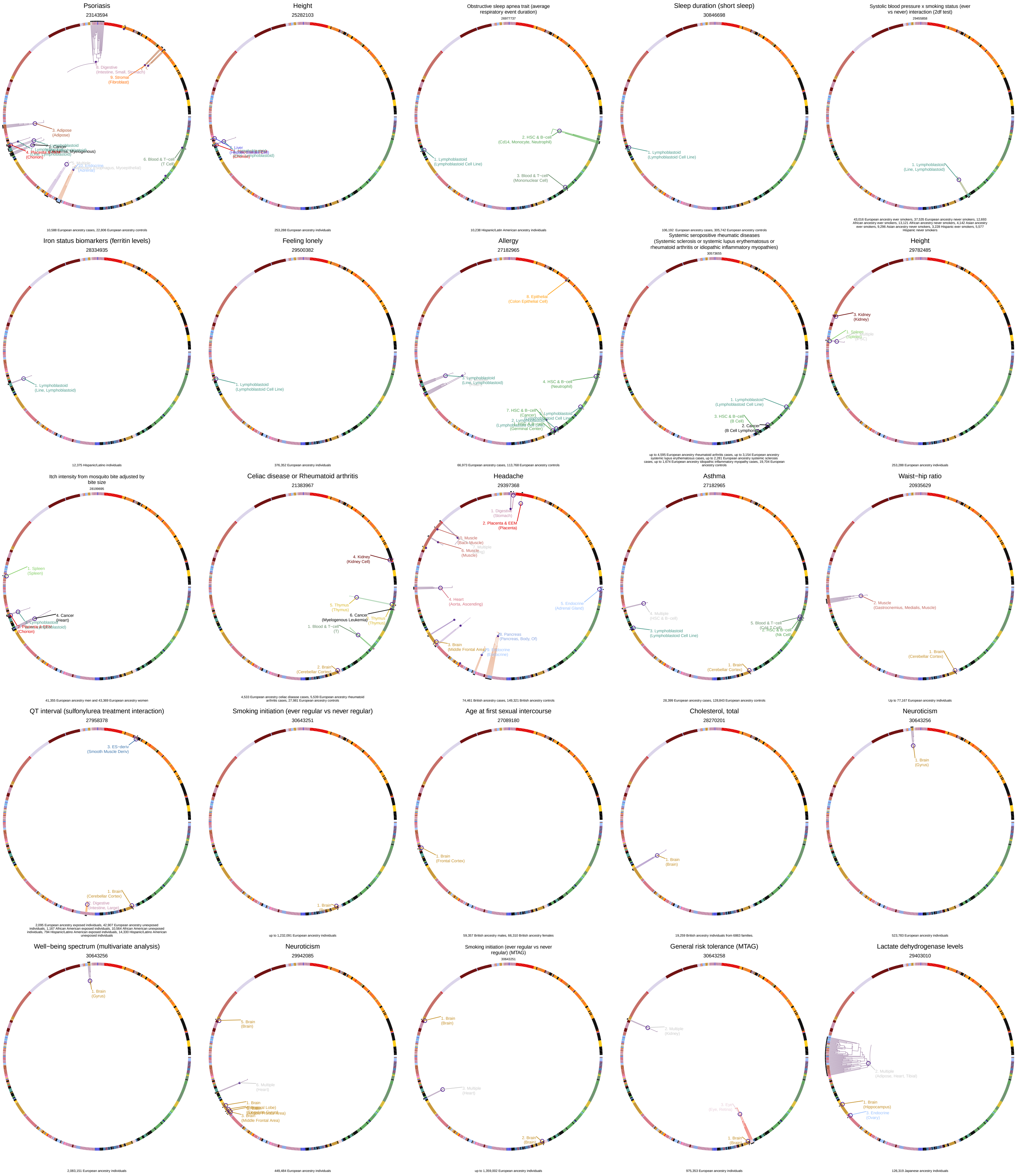
Psoriasis
23143594
8. Digestive (Intestine, Small, Stomach)
9. Stromal (Fibroblast)
3. Adipose (Adipose)
1. Cancer (Leukemia, Myelogenous)
4. Placenta & EEM (Chorion)
6. Blood & T−cell (T Cell)
10,588 European ancestry cases, 22,806 European ancestry controls
Height
25282103
253,288 European ancestry individuals
Obstructive sleep apnea trait (average respiratory event duration)
26977737
2. HSC & B−cell (Cd14, Monocyte, Neutrophil)
1. Lymphoblastoid (Lymphoblastoid Cell Line)
3. Blood & T−cell (Mononuclear Cell)
10,238 Hispanic/Latin American ancestry individuals
Sleep duration (short sleep)
30846698
1. Lymphoblastoid (Lymphoblastoid Cell Line)
106,192 European ancestry cases, 305,742 European ancestry controls
Systolic blood pressure x smoking status (ever vs never) interaction (2df test)
29455858
1. Lymphoblastoid (Line, Lymphoblastoid)
43,016 European ancestry ever smokers, 37,535 European ancestry never smokers, 12,693 African ancestry ever smokers, 13,121 African ancestry never smokers, 4,142 Asian ancestry ever smokers, 9,296 Asian ancestry never smokers, 3,228 Hispanic ever smokers, 5,577 Hispanic never smokers
Iron status biomarkers (ferritin levels)
28334935
1. Lymphoblastoid (Line, Lymphoblastoid)
12,375 Hispanic/Latino individuals
Feeling lonely
29500382
1. Lymphoblastoid (Lymphoblastoid Cell Line)
376,352 European ancestry individuals
Allergy
27182965
8. Epithelial (Colon Epithelial Cell)
4. HSC & B−cell (Neutrophil)
7. HSC & B−cell (Cancer)
66,973 European ancestry cases, 113,768 European ancestry controls
Systemic seropositive rheumatic diseases (Systemic sclerosis or systemic lupus erythematosus or rheumatoid arthritis or idiopathic inflammatory myopathies)
30573655
1. Lymphoblastoid (Lymphoblastoid Cell Line)
3. HSC & B−cell (B Cell)
2. Cancer
up to 4,595 European ancestry rheumatoid arthritis cases, up to 3,154 European ancestry systemic lupus erythematosus cases, up to 2,281 European ancestry systemic sclerosis cases, up to 1,674 European ancestry idiopathic inflammatory myopathy cases, 19,704 European ancestry controls
Height
29782485
3. Kidney (Kidney)
253,288 European ancestry individuals
Itch intensity from mosquito bite adjusted by bite size
28199695
1. Spleen (Spleen)
4. Cancer (Heart)
41,355 European ancestry men and 43,369 European ancestry women
Celiac disease or Rheumatoid arthritis
21383967
4. Kidney (Kidney Cell)
5. Thymus (Thymus)
6. Cancer (Myelogenous Leukemia)
1. Blood & T−cell (T)
2. Brain (Cerebellar Cortex)
4,533 European ancestry celiac disease cases, 5,539 European ancestry rheumatoid arthritis cases, 27,981 European ancestry controls
Headache
29397368
1. Digestive (Stomach)
2. Placenta & EEM (Placenta)
10. Muscle (Back Muscle)
6. Muscle (Muscle)
4. Heart (Aorta, Ascending)
5. Endocrine (Adrenal Gland)
8. Pancreas (Pancreas, Body, Of)
3. Brain (Middle Frontal Area)
74,461 British ancestry cases, 149,321 British ancestry controls
Asthma
27182965
4. Multiple (HSC & B−cell)
3. Lymphoblastoid (Lymphoblastoid Cell Line)
5. Blood & T−cell
1. Brain (Cerebellar Cortex)
28,399 European ancestry cases, 128,843 European ancestry controls
Waist−hip ratio
20935629
2. Muscle (Gastrocnemius, Medialis, Muscle)
1. Brain (Cerebellar Cortex)
Up to 77,167 European ancestry individuals
QT interval (sulfonylurea treatment interaction)
27958378
3. ES−deriv (Smooth Muscle Deriv)
1. Brain (Cerebellar Cortex)
2. Digestive (Intestine, Large)
2,095 European ancestry exposed individuals, 42,907 European ancestry unexposed individuals, 1,167 African American exposed individuals, 10,564 African American unexposed individuals, 794 Hispanic/Latino American exposed individuals, 14,330 Hispanic/Latino American unexposed individuals
Smoking initiation (ever regular vs never regular)
30643251
up to 1,232,091 European ancestry individuals
Age at first sexual intercourse
27089180
1. Brain (Frontal Cortex)
59,357 British ancestry males, 66,310 British ancestry females
Cholesterol, total
28270201
1. Brain (Brain)
19,259 British ancestry individuals from 6863 families.
Neuroticism
30643256
1. Brain (Gyrus)
523,783 European ancestry individuals
Well−being spectrum (multivariate analysis)
30643256
1. Brain (Gyrus)
2,083,151 European ancestry individuals
Neuroticism
29942085
5. Brain (Brain)
6. Multiple (Heart)
1. Brain
3. Brain (Middle Frontal Area)
449,484 European ancestry individuals
Smoking initiation (ever regular vs never regular) (MTAG)
30643251
1. Brain (Brain)
3. Multiple (Heart)
2. Brain (Brain)
up to 1,359,002 European ancestry individuals
General risk tolerance (MTAG)
30643258
2. Multiple (Kidney)
3. Eye (Eye, Retina)
1. Brain (Brain)
975,353 European ancestry individuals
Lactate dehydrogenase levels
29403010
2. Multiple (Adipose, Heart, Tibial)
1. Brain (Hippocampus)
3. Endocrine (Ovary)
126,319 Japanese ancestry individuals

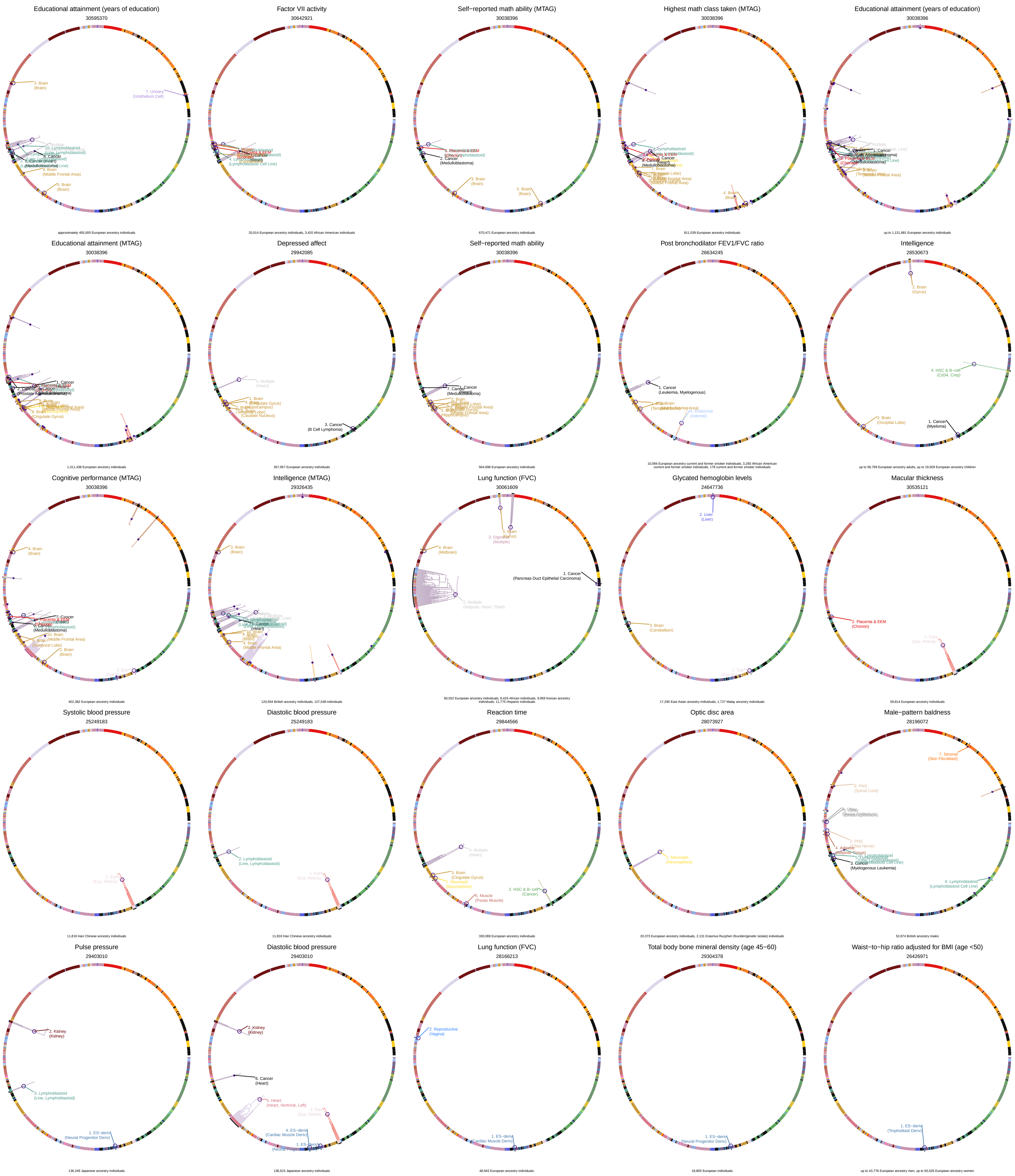

Educational attainment (years of education)
30595370
approximately 455,000 European ancestry individuals
Factor VII activity
30642921
20,014 European ancestry individuals, 3,420 African American individuals
Self−reported math ability (MTAG)
30038396
670,471 European ancestry individuals
Highest math class taken (MTAG)
30038396
811,539 European ancestry individuals
Educational attainment (years of education)
30038396
up to 1,131,881 European ancestry individuals
Educational attainment (MTAG)
30038396
1,311,438 European ancestry individuals
Depressed affect
29942085
357,957 European ancestry individuals
Self−reported math ability
30038396
564,698 European ancestry individuals
Post bronchodilator FEV1/FVC ratio
26634245
10,094 European ancestry current and former smoker individuals, 3,260 African American current and former smoker individuals, 178 current and former smoker individuals
Intelligence
28530673
up to 58,799 European ancestry adults, up to 19,509 European ancestry children
Cognitive performance (MTAG)
30038396
402,382 European ancestry individuals
Intelligence (MTAG)
29326435
120,934 British ancestry individuals, 127,548 individuals
Lung function (FVC)
30061609
60,552 European ancestry individuals, 8,429 African individuals, 9,959 Korean ancestry individuals, 11,775 Hispanic individuals
Glycated hemoglobin levels
24647736
17,290 East Asian ancestry individuals, 1,727 Malay ancestry individuals
Macular thickness
30535121
59,814 European ancestry individuals
Systolic blood pressure
25249183
11,816 Han Chinese ancestry individuals
Diastolic blood pressure
25249183
11,816 Han Chinese ancestry individuals
Reaction time
29844566
330,069 European ancestry individuals
Optic disc area
28073927
20,373 European ancestry individuals, 2,131 Erasmus Rucphen (founder/genetic isolate) individuals
Male−pattern baldness
28196072
52,874 British ancestry males
Pulse pressure
29403010
136,249 Japanese ancestry individuals
Diastolic blood pressure
29403010
136,615 Japanese ancestry individuals
Lung function (FVC)
28166213
48,943 European ancestry individuals.
Total body bone mineral density (age 45−60)
29304378
18,805 European individuals
Waist−to−hip ratio adjusted for BMI (age <50)
26426971
up to 43,776 European ancestry men, up to 50,526 European ancestry women

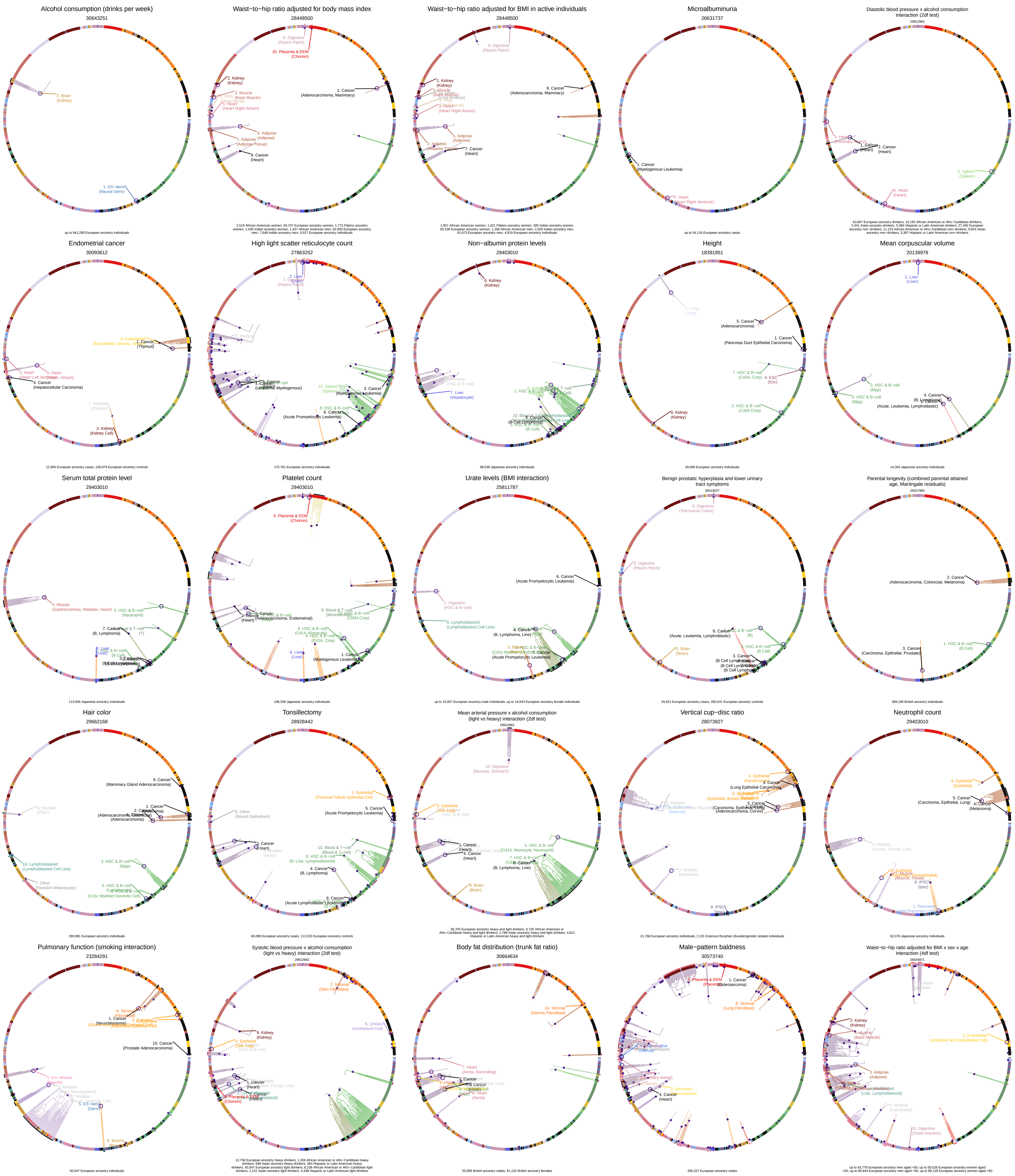
Alcohol consumption (drinks per week)
30643251
up to 941,280 European ancestry individuals
Waist−to−hip ratio adjusted for body mass index
28448500
2,518 African American women, 83,707 European ancestry women, 1,773 Filipino ancestry women, 1,030 Indian ancestry women, 1,457 African American men, 52,993 European ancestry men, 7,648 Indian ancestry men, 5,527 European ancestry individuals
Waist−to−hip ratio adjusted for BMI in active individuals
28448500
1,957 African American women, 1,421 Filipino ancestry women, 305 Indian ancestry women, 65,539 European ancestry women, 1,206 African American men, 2,029 Indian ancestry men, 41,673 European ancestry men, 4,819 European ancestry individuals
Microalbuminuria
26631737
up to 54,116 European ancestry cases
Diastolic blood pressure x alcohol consumption interaction (2df test)
29912962
63,607 European ancestry drinkers, 10,193 African American or Afro−Caribbean drinkers, 2,441 Asian ancestry drinkers, 5,084 Hispanic or Latin American drinkers, 27,492 European ancestry non−drinkers, 11,223 African American or Afro−Caribbean non−drinkers, 9,924 Asian ancestry non−drinkers, 3,387 Hispanic or Latin American non−drinkers
Endometrial cancer
30093612
12,906 European ancestry cases, 108,979 European ancestry controls
High light scatter reticulocyte count
27863252
170,761 European ancestry individuals
Non−albumin protein levels
29403010
98,538 Japanese ancestry individuals
Height
18391951
30,968 European ancestry individuals
Mean corpuscular volume
20139978
14,364 Japanese ancestry individuals
Serum total protein level
29403010
113,509 Japanese ancestry individuals
Platelet count
29403010
108,208 Japanese ancestry individuals
Urate levels (BMI interaction)
25811787
up to 15,007 European ancestry male individuals, up to 16,943 European ancestry female individuals
Benign prostatic hyperplasia and lower urinary tract symptoms
30410027
20,621 European ancestry cases, 280,541 European ancestry controls
Parental longevity (combined parental attained age, Martingale residuals)
29227965
389,166 British ancestry individuals
Hair color
29662168
290,891 European ancestry individuals
Tonsillectomy
28928442
60,098 European ancestry cases, 113,323 European ancestry controls
Mean arterial pressure x alcohol consumption (light vs heavy) interaction (2df test)
29912962
56,705 European ancestry heavy and light drinkers, 9,725 African American or Afro−Caribbean heavy and light drinkers, 1,789 Asian ancestry heavy and light drinkers, 4,812 Hispanic or Latin American heavy and light drinkers
Vertical cup−disc ratio
28073927
21,768 European ancestry individuals, 2,131 Erasmus Rucphen (founder/genetic isolate) individuals
Neutrophil count
29403010
62,076 Japanese ancestry individuals
Pulmonary function (smoking interaction)
23284291
50,047 European ancestry individuals
Systolic blood pressure x alcohol consumption (light vs heavy) interaction (2df test)
29912962
12,758 European ancestry heavy drinkers, 1,326 African American or Afro−Caribbean heavy drinkers, 648 Asian ancestry heavy drinkers, 364 Hispanic or Latin American heavy drinkers, 43,947 European ancestry light drinkers, 8,239 African American or Afro−Caribbean light drinkers, 1,141 Asian ancestry light drinkers, 4,448 Hispanic or Latin American light drinkers
Body fat distribution (trunk fat ratio)
30664634
55,006 British ancestry males, 61,132 British ancestry females
Male−pattern baldness
30573740
205,327 European ancestry males
Waist−to−hip ratio adjusted for BMI x sex x age interaction (4df test)
28426971
up to 43,776 European ancestry men aged <50, up to 50,526 European ancestry women aged <50, up to 69,944 European ancestry men aged >50, up to 88,129 European ancestry women aged >50

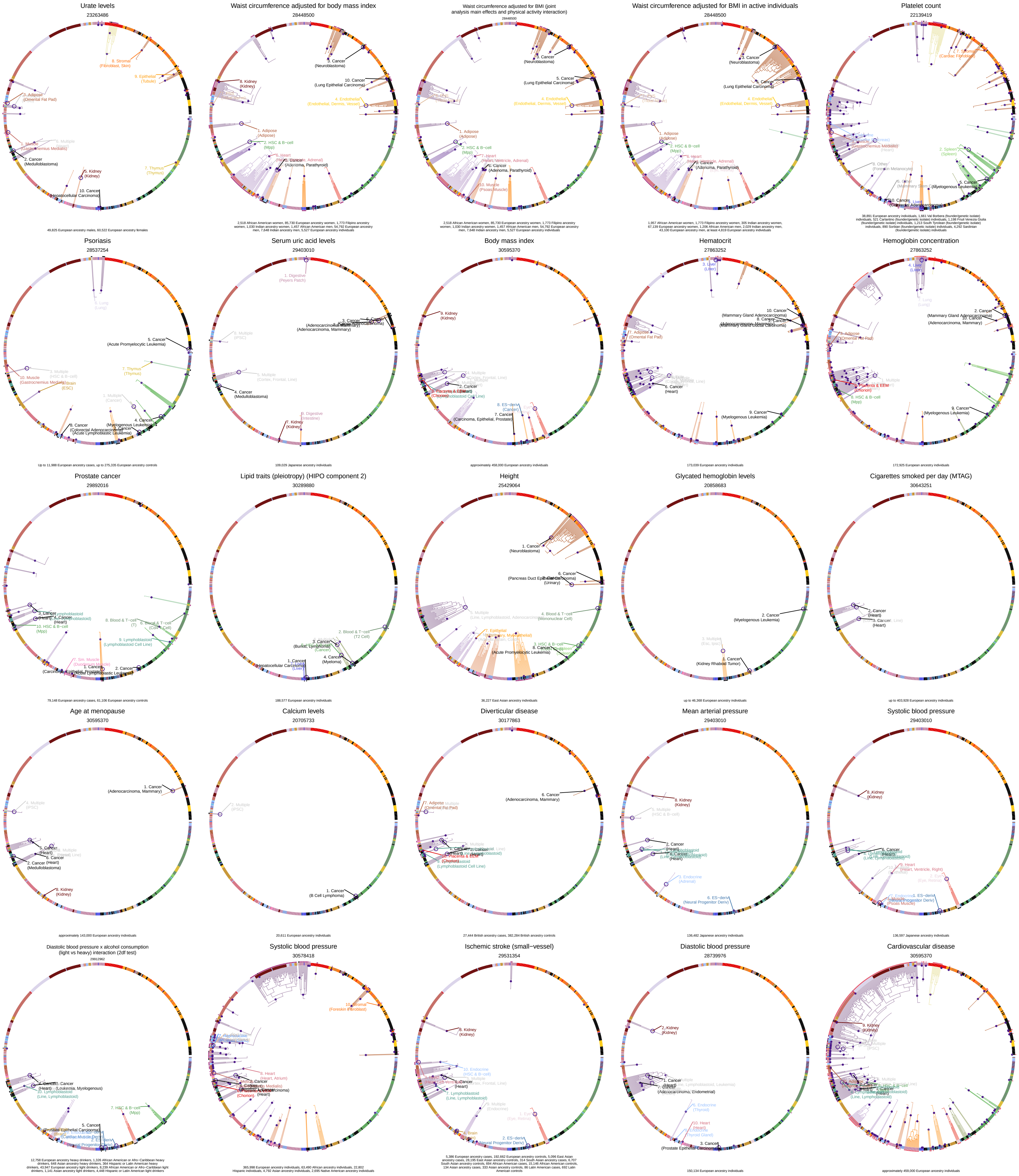
Urate levels
23263486
49,825 European ancestry males, 60,522 European ancestry females
Waist circumference adjusted for body mass index
28448500
2,518 African American women, 85,730 European ancestry women, 1,773 Filipino ancestry women, 1,030 Indian ancestry women, 1,457 African American men, 54,792 European ancestry men, 7,648 Indian ancestry men, 5,527 European ancestry individuals
Waist circumference adjusted for BMI (joint analysis main effects and physical activity interaction)
28448500
2,518 African American women, 85,730 European ancestry women, 1,773 Filipino ancestry women, 1,030 Indian ancestry women, 1,457 African American men, 54,792 European ancestry men, 7,648 Indian ancestry men, 5,527 European ancestry individuals
Waist circumference adjusted for BMI in active individuals
28448500
1,957 African American women, 1,773 Filipino ancestry women, 305 Indian ancestry women, 67,139 European ancestry women, 1,206 African American men, 2,029 Indian ancestry men, 43,100 European ancestry men, at least 4,819 European ancestry individuals
Platelet count
22139419
38,891 European ancestry individuals, 1,661 Val Borbera (founder/genetic isolate) individuals, 521 Carlantino (founder/genetic isolate) individuals, 1,138 Friuli Venezia Giulia (founder/genetic isolate) individuals, 1,213 South Tyrolean (founder/genetic isolate) individuals, 890 Sorbian (founder/genetic isolate) individuals, 4,292 Sardinian (founder/genetic isolate) individuals
Psoriasis
28537254
Up to 11,988 European ancestry cases, up to 275,335 European ancestry controls
Serum uric acid levels
29403010
109,029 Japanese ancestry individuals
Body mass index
30595370
approximately 458,000 European ancestry individuals
Hematocrit
27863252
173,039 European ancestry individuals
Hemoglobin concentration
27863252
172,925 European ancestry individuals
Prostate cancer
29892016
79,148 European ancestry cases, 61,106 European ancestry controls
Lipid traits (pleiotropy) (HIPO component 2)
30289880
188,577 European ancestry individuals
Height
25429064
36,227 East Asian ancestry individuals
Glycated hemoglobin levels
20858683
up to 46,368 European ancestry individuals
Cigarettes smoked per day (MTAG)
30643251
up to 403,928 European ancestry individuals
Age at menopause
30595370
approximately 143,000 European ancestry individuals
Calcium levels
20705733
20,611 European ancestry individuals
Diverticular disease
30177863
27,444 British ancestry cases, 382,284 British ancestry controls
Mean arterial pressure
29403010
136,482 Japanese ancestry individuals
Systolic blood pressure
29403010
136,597 Japanese ancestry individuals
Diastolic blood pressure x alcohol consumption (light vs heavy) interaction (2df test)
29912962
12,758 European ancestry heavy drinkers, 1,326 African American or Afro-Caribbean heavy drinkers, 646 Asian ancestry heavy drinkers, 364 Hispanic or Latin American heavy drinkers, 43,947 European ancestry light drinkers, 8,239 African American or Afro-Caribbean light drinkers, 1,141 Asian ancestry light drinkers, 4,448 Hispanic or Latin American light drinkers
Systolic blood pressure
30578418
365,998 European ancestry individuals, 63,490 African ancestry individuals, 22,802 Hispanic individuals, 4,792 Asian ancestry individuals, 2,695 Native American ancestry individuals
Ischemic stroke (small−vessel)
29531354
5,386 European ancestry cases, 192,662 European ancestry controls, 5,096 East Asian ancestry cases, 28,195 East Asian ancestry controls, 314 South Asian ancestry cases, 6,707 South Asian ancestry controls, 694 African American cases, 15,146 African American controls, 134 Asian ancestry cases, 333 Asian ancestry controls, 86 Latin American cases, 692 Latin American controls
Diastolic blood pressure
28739976
150,134 European ancestry individuals
Cardiovascular disease
30595370
approximately 459,000 European ancestry individuals

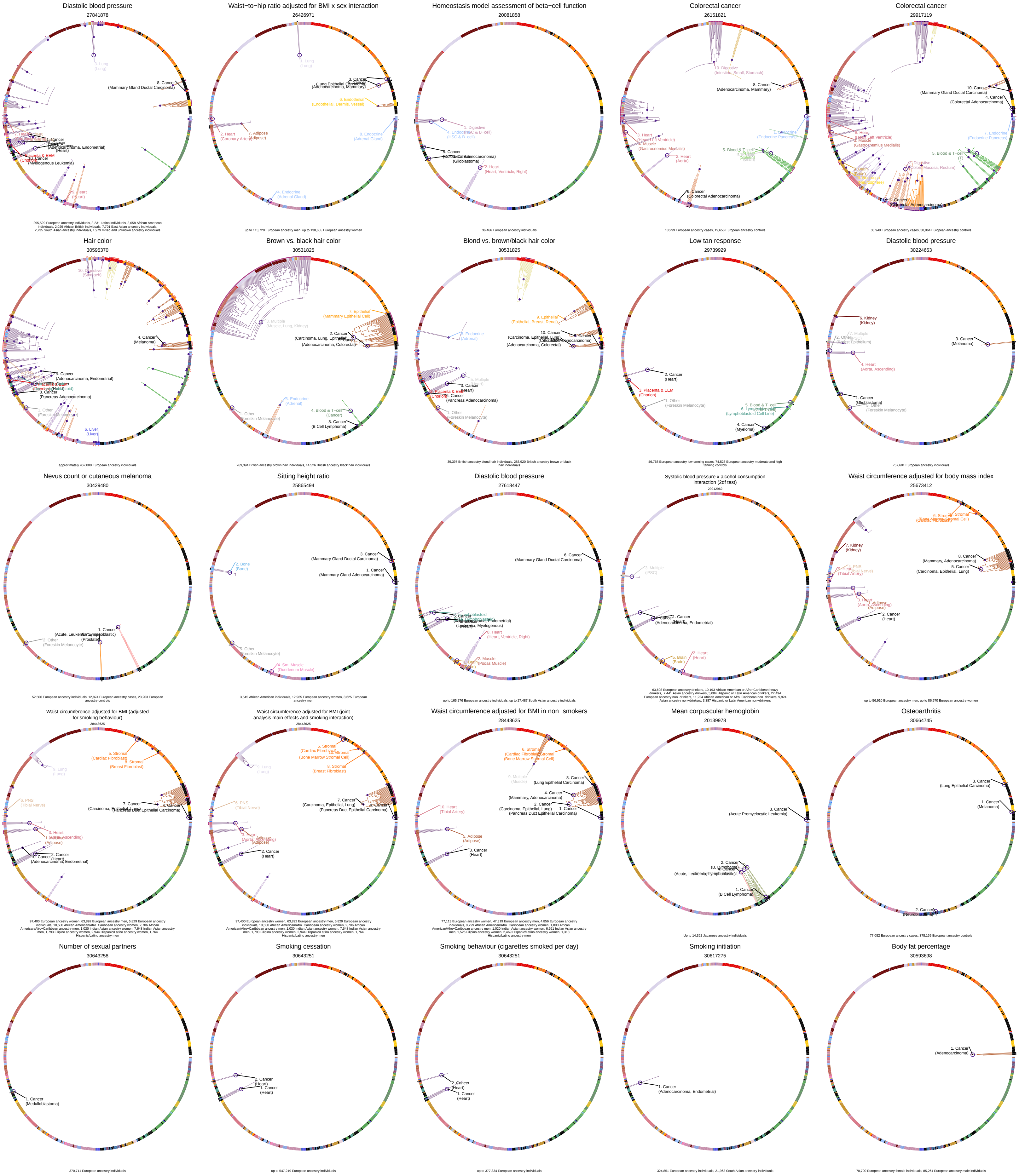
Diastolic blood pressure
27841878
295,529 European ancestry individuals, 8,231 Latino individuals, 3,058 African American individuals, 2,029 African British individuals, 7,701 East Asian ancestry individuals, 2,735 South Asian ancestry individuals, 1,979 mixed and unknown ancestry individuals
Waist−to−hip ratio adjusted for BMI x sex interaction
26426971
up to 113,720 European ancestry men, up to 138,655 European ancestry women
Homeostasis model assessment of beta−cell function
20081858
36,466 European ancestry individuals
Colorectal cancer
26151821
18,299 European ancestry cases, 19,656 European ancestry controls
Colorectal cancer
29917119
36,948 European ancestry cases, 30,864 European ancestry controls
Hair color
30595370
approximately 452,000 European ancestry individuals
Brown vs. black hair color
30531825
269,394 British ancestry brown hair individuals, 14,526 British ancestry black hair individuals
Blond vs. brown/black hair color
30531825
39,397 British ancestry blond hair individuals, 283,920 British ancestry brown or black hair individuals
Low tan response
29739929
46,768 European ancestry low tanning cases, 74,528 European ancestry moderate and high tanning controls
Diastolic blood pressure
30224653
757,601 European ancestry individuals
Nevus count or cutaneous melanoma
30429480
52,506 European ancestry individuals, 12,874 European ancestry cases, 23,203 European ancestry controls
Sitting height ratio
25865494
3,545 African American individuals, 12,965 European ancestry women, 8,625 European ancestry men
Diastolic blood pressure
27618447
up to 165,276 European ancestry individuals, up to 27,487 South Asian ancestry individuals
Systolic blood pressure x alcohol consumption interaction (2df test)
29912962
63,608 European ancestry drinkers, 10,193 African American or Afro−Caribbean heavy drinkers, 2,441 Asian ancestry drinkers, 5,084 Hispanic or Latin American drinkers, 27,494 European ancestry non−drinkers, 11,224 African American or Afro−Caribbean non−drinkers, 9,924 Asian ancestry non−drinkers, 3,387 Hispanic or Latin American non−drinkers
Waist circumference adjusted for body mass index
25673412
up to 56,910 European ancestry men, up to 86,570 European ancestry women
Waist circumference adjusted for BMI (adjusted for smoking behaviour)
28443625
97,400 European ancestry women, 63,892 European ancestry men, 5,829 European ancestry individuals, 10,500 African American/Afro−Caribbean ancestry women, 2,706 African American/Afro−Caribbean ancestry men, 1,030 Indian Asian ancestry women, 7,648 Indian Asian ancestry men, 1,793 Filipino ancestry women, 2,944 Hispanic/Latino ancestry women, 1,764 Hispanic/Latino ancestry men
Waist circumference adjusted for BMI (joint analysis main effects and smoking interaction)
28443625
97,400 European ancestry women, 63,892 European ancestry men, 5,829 European ancestry individuals, 10,500 African American/Afro−Caribbean ancestry women, 2,706 African American/Afro−Caribbean ancestry men, 1,030 Indian Asian ancestry women, 7,648 Indian Asian ancestry men, 1,793 Filipino ancestry women, 2,944 Hispanic/Latino ancestry women, 1,764 Hispanic/Latino ancestry men
Waist circumference adjusted for BMI in non−smokers
28443625
77,113 European ancestry women, 47,319 European ancestry men, 4,856 European ancestry individuals, 8,799 African American/Afro−Caribbean ancestry women, 1,803 African American/Afro−Caribbean ancestry men, 1,020 Indian Asian ancestry women, 6,691 Indian Asian ancestry men, 1,526 Filipino ancestry women, 2,489 Hispanic/Latino ancestry women, 1,318 Hispanic/Latino ancestry men
Mean corpuscular hemoglobin
20139978
Up to 14,362 Japanese ancestry individuals
Osteoarthritis
30664745
77,052 European ancestry cases, 378,169 European ancestry controls
Number of sexual partners
30643258
370,711 European ancestry individuals
Smoking cessation
30643251
up to 547,219 European ancestry individuals
Smoking behaviour (cigarettes smoked per day)
30643251
up to 377,334 European ancestry individuals
Smoking initiation
30617275
324,851 European ancestry individuals, 21,962 South Asian ancestry individuals
Body fat percentage
30593698
70,700 European ancestry female individuals, 85,261 European ancestry male individuals

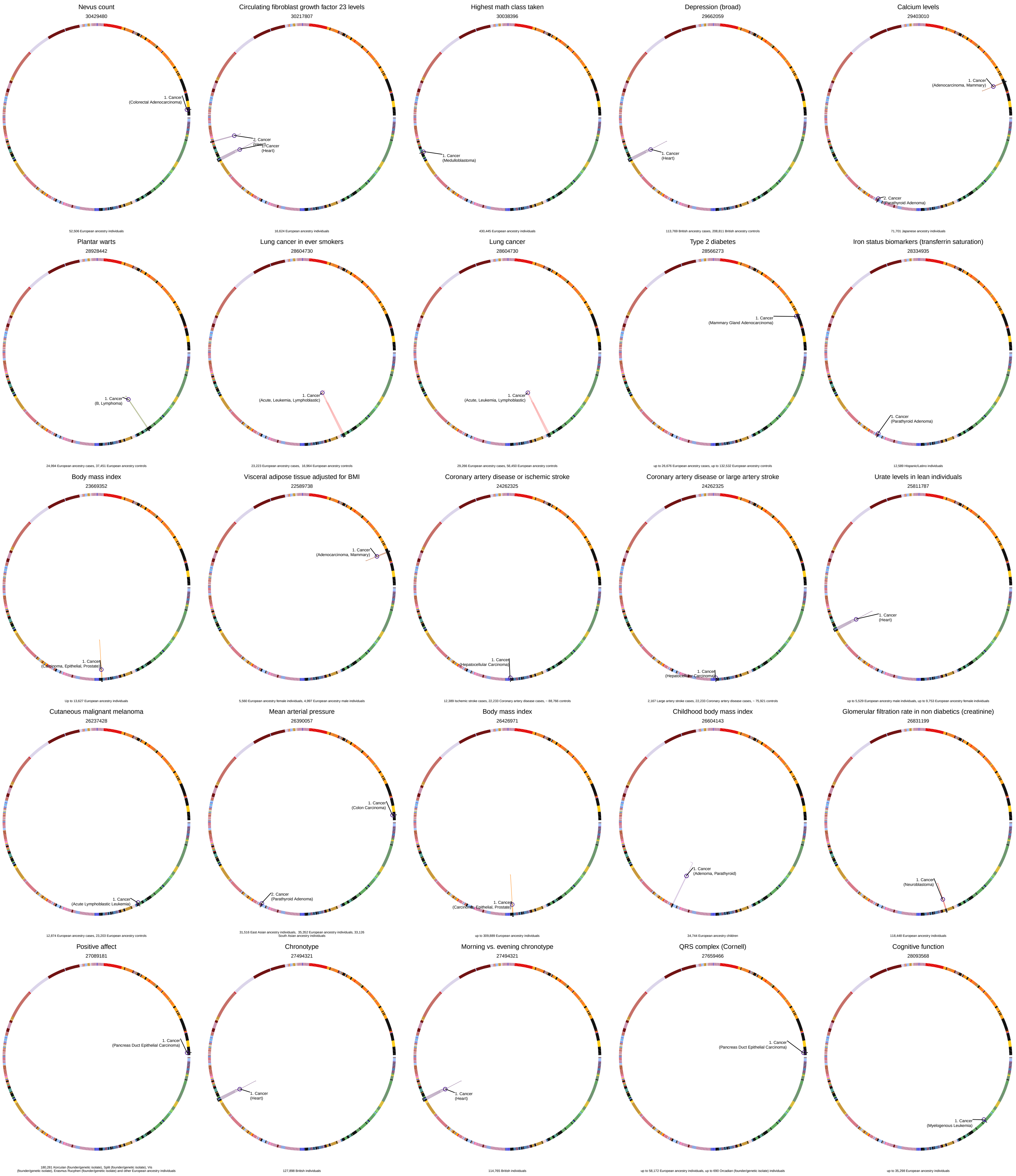
Nevus count
30429480
1. Cancer
(Colorectal Adenocarcinoma)
52,506 European ancestry individuals
Circulating fibroblast growth factor 23 levels
30217807
2. Cancer
(Heart)
1. Cancer
(Heart)
16,624 European ancestry individuals
Highest math class taken
30038396
1. Cancer
(Medulloblastoma)
430,445 European ancestry individuals
Depression (broad)
29662059
1. Cancer
(Heart)
113,769 British ancestry cases, 208,811 British ancestry controls
Calcium levels
29403010
1. Cancer
(Adenocarcinoma, Mammary)
2. Cancer
(Parathyroid Adenoma)
71,701 Japanese ancestry individuals
Plantar warts
28928442
1. Cancer
(B, Lymphoma)
24,994 European ancestry cases, 37,451 European ancestry controls
Lung cancer in ever smokers
28604730
1. Cancer
(Acute, Leukemia, Lymphoblastic)
23,223 European ancestry cases, 16,964 European ancestry controls
Lung cancer
28604730
1. Cancer
(Acute, Leukemia, Lymphoblastic)
29,266 European ancestry cases, 56,450 European ancestry controls
Type 2 diabetes
28566273
1. Cancer
(Mammary Gland Adenocarcinoma)
up to 26,676 European ancestry cases, up to 132,532 European ancestry controls
Iron status biomarkers (transferrin saturation)
28334935
1. Cancer
(Parathyroid Adenoma)
12,589 Hispanic/Latino individuals
Body mass index
23669352
1. Cancer
(Carcinoma, Epithelial, Prostate)
Up to 13,627 European ancestry individuals
Visceral adipose tissue adjusted for BMI
22589738
1. Cancer
(Adenocarcinoma, Mammary)
5,560 European ancestry female individuals, 4,997 European ancestry male individuals
Coronary artery disease or ischemic stroke
24262325
1. Cancer
(Hepatocellular Carcinoma)
12,389 Ischemic stroke cases, 22,233 Coronary artery disease cases, ~ 88,766 controls
Coronary artery disease or large artery stroke
24262325
1. Cancer
(Hepatocellular Carcinoma)
2,167 Large artery stroke cases, 22,233 Coronary artery disease cases, ~ 75,921 controls
Urate levels in lean individuals
25811787
1. Cancer
(Heart)
up to 5,529 European ancestry male individuals, up to 9,753 European ancestry female individuals
Cutaneous malignant melanoma
26237428
1. Cancer
(Acute Lymphoblastic Leukemia)
12,874 European ancestry cases, 23,203 European ancestry controls
Mean arterial pressure
26390057
1. Cancer
(Colon Carcinoma)
2. Cancer
(Parathyroid Adenoma)
31,516 East Asian ancestry individuals, 35,352 European ancestry individuals, 33,126
South Asian ancestry individuals
Body mass index
26426971
1. Cancer
(Carcinoma, Epithelial, Prostate)
up to 309,889 European ancestry individuals
Childhood body mass index
26604143
1. Cancer
(Adenoma, Parathyroid)
34,744 European ancestry children
Glomerular filtration rate in non diabetics (creatinine)
26831199
1. Cancer
(Neuroblastoma)
118,448 European ancestry individuals
Positive affect
27089181
1. Cancer
(Pancreas Duct Epithelial Carcinoma)
180,281 Korculan (founder/genetic isolate), Split (founder/genetic isolate), Vis
(founder/genetic isolate), Erasmus Rucphen (founder/genetic isolate) and other European ancestry individuals
Chronotype
27494321
1. Cancer
(Heart)
127,898 British individuals
Morning vs. evening chronotype
27494321
1. Cancer
(Heart)
114,765 British individuals
QRS complex (Cornell)
27659466
1. Cancer
(Pancreas Duct Epithelial Carcinoma)
up to 58,172 European ancestry individuals, up to 690 Orcadian (founder/genetic isolate) individuals
Cognitive function
28093568
1. Cancer
(Myelogenous Leukemia)
up to 35,298 European ancestry individuals

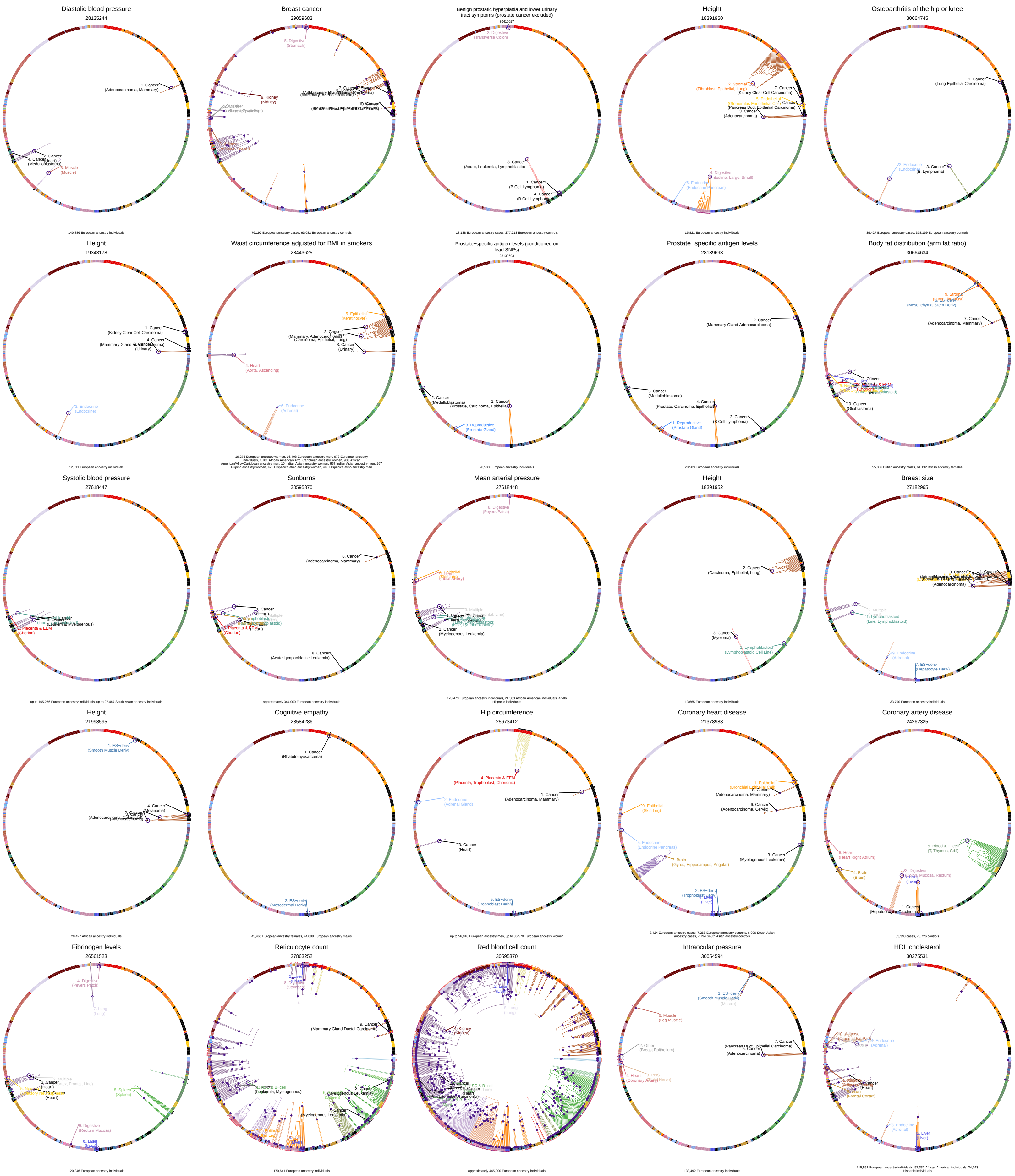

Diastolic blood pressure
28135244
140,886 European ancestry individuals
Breast cancer
29059683
76,192 European ancestry cases, 63,082 European ancestry controls
Benign prostatic hyperplasia and lower urinary tract symptoms (prostate cancer excluded)
30410027
18,138 European ancestry cases, 277,213 European ancestry controls
Height
18391950
15,821 European ancestry individuals
Osteoarthritis of the hip or knee
30664745
39,427 European ancestry cases, 378,169 European ancestry controls
Height
19343178
12,611 European ancestry individuals
Waist circumference adjusted for BMI in smokers
28443625
19,276 European ancestry women, 16,408 European ancestry men, 973 European ancestry individuals, 1,701 African American/Afro−Caribbean ancestry women, 903 African American/Afro−Caribbean ancestry men, 10 Indian Asian ancestry women, 957 Indian Asian ancestry men, 267 Filipino ancestry women, 475 Hispanic/Latino ancestry women, 446 Hispanic/Latino ancestry men
Prostate−specific antigen levels (conditioned on lead SNPs)
28139693
28,503 European ancestry individuals
Prostate−specific antigen levels
28139693
28,503 European ancestry individuals
Body fat distribution (arm fat ratio)
30664634
55,006 British ancestry males, 61,132 British ancestry females
Systolic blood pressure
27618447
up to 165,276 European ancestry individuals, up to 27,487 South Asian ancestry individuals
Sunburns
30595370
approximately 344,000 European ancestry individuals
Mean arterial pressure
27618448
120,473 European ancestry individuals, 21,503 African American individuals, 4,586 Hispanic individuals
Height
18391952
13,665 European ancestry individuals
Breast size
27182965
33,790 European ancestry individuals
Height
21998595
20,427 African ancestry individuals
Cognitive empathy
28584286
45,465 European ancestry females, 44,088 European ancestry males
Hip circumference
25673412
up to 56,910 European ancestry men, up to 86,570 European ancestry women
Coronary heart disease
21378988
8,424 European ancestry cases, 7,268 European ancestry controls, 6,996 South Asian ancestry cases, 7,794 South Asian ancestry controls
Coronary artery disease
24262325
33,398 cases, 75,726 controls
Fibrinogen levels
26561523
120,246 European ancestry individuals
Reticulocyte count
27863252
170,641 European ancestry individuals
Red blood cell count
30595370
approximately 445,000 European ancestry individuals
Intraocular pressure
30054594
133,492 European ancestry individuals
HDL cholesterol
30275531
215,551 European ancestry individuals, 57,332 African American individuals, 24,743 Hispanic individuals

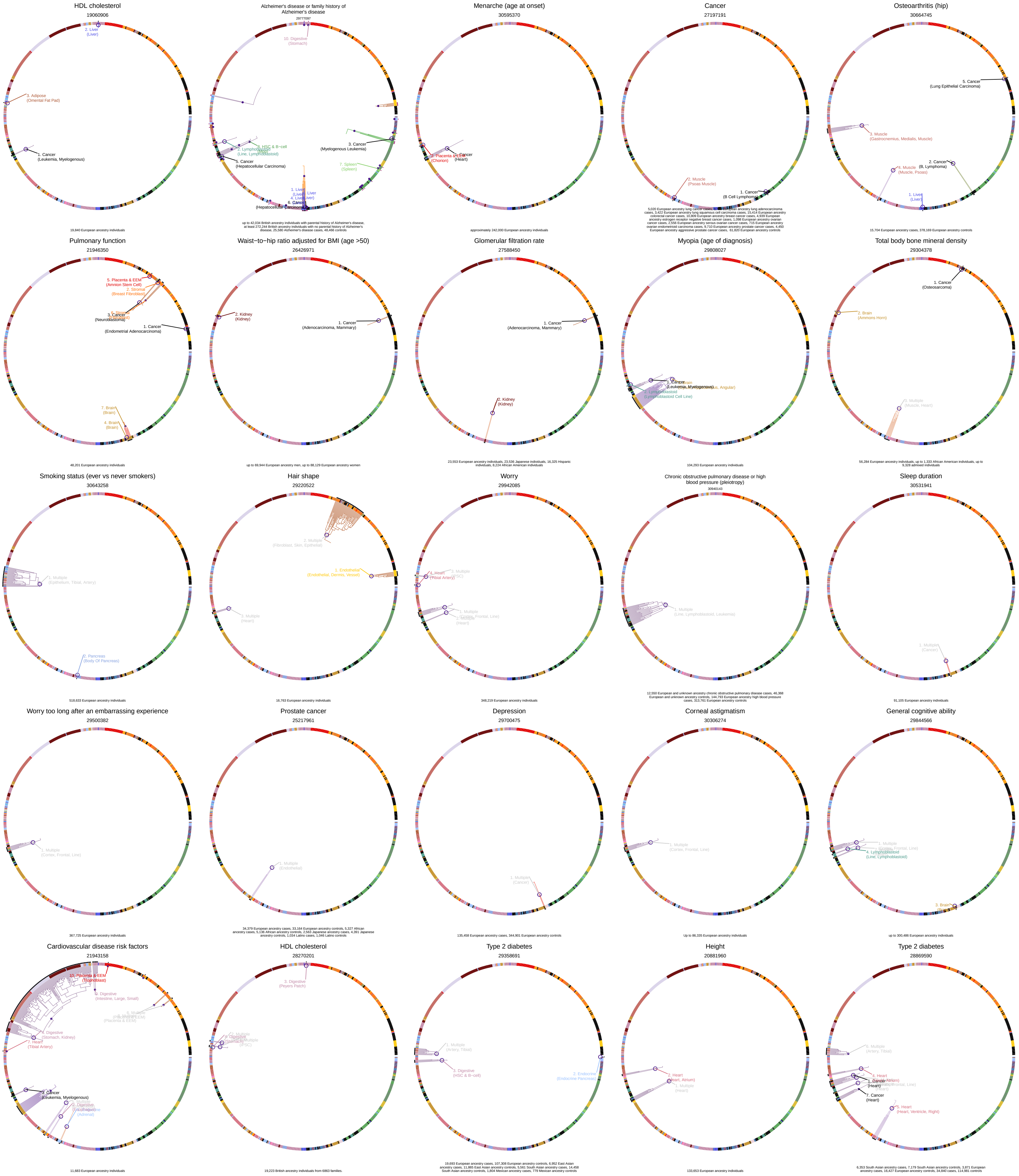

HDL cholesterol
19060906
19,840 European ancestry individuals
Alzheimer's disease or family history of Alzheimer's disease
29777097
up to 42,034 British ancestry individuals with parental history of Alzheimer's disease, at least 272,244 British ancestry individuals with no parental history of Alzheimer's disease, 25,580 Alzheimer's disease cases, 48,466 controls
Menarche (age at onset)
30595370
approximately 242,000 European ancestry individuals
Cancer
27197191
2. Muscle (Psoas Muscle)
1. Cancer (B Cell Lymphoma)
Osteoarthritis (hip)
30664745
15,704 European ancestry cases, 378,169 European ancestry controls
Pulmonary function
21946350
48,201 European ancestry individuals
Waist−to−hip ratio adjusted for BMI (age >50)
26426971
up to 69,944 European ancestry men, up to 88,129 European ancestry women
Glomerular filtration rate
27588450
23,553 European ancestry individuals, 23,536 Japanese individuals, 16,325 Hispanic individuals, 8,224 African American individuals
Myopia (age of diagnosis)
29808027
104,293 European ancestry individuals
Total body bone mineral density
29304378
56,284 European ancestry individuals, up to 1,333 African American individuals, up to 9,328 admixed individuals
Smoking status (ever vs never smokers)
30643258
518,633 European ancestry individuals
Hair shape
29220522
16,763 European ancestry individuals
Worry
29942085
348,219 European ancestry individuals
Chronic obstructive pulmonary disease or high blood pressure (pleiotropy)
30940143
12,550 European and unknown ancestry chronic obstructive pulmonary disease cases, 46,368 European and unknown ancestry controls, 144,793 European ancestry high blood pressure cases, 313,761 European ancestry controls
Sleep duration
30531941
91,105 European ancestry individuals
Worry too long after an embarrassing experience
29500382
367,725 European ancestry individuals
Prostate cancer
25217961
1. Multiple (Endothelial)
34,379 European ancestry cases, 33,164 European ancestry controls, 5,327 African ancestry cases, 5,136 African ancestry controls, 2,563 Japanese ancestry cases, 4,391 Japanese ancestry controls, 1,034 Latino cases, 1,046 Latino controls
Depression
29700475
135,458 European ancestry cases, 344,901 European ancestry controls
Corneal astigmatism
30306274
Up to 86,335 European ancestry individuals
General cognitive ability
29844566
up to 300,486 European ancestry individuals
Cardiovascular disease risk factors
21943158
11,683 European ancestry individuals
HDL cholesterol
28270201
3. Digestive (Peyers Patch)
19,223 British ancestry individuals from 6863 families.
Type 2 diabetes
29358691
19,693 European ancestry cases, 107,308 European ancestry controls, 6,952 East Asian ancestry cases, 11,865 East Asian ancestry controls, 5,561 South Asian ancestry cases, 14,458 South Asian ancestry controls, 1,804 Mexican ancestry cases, 779 Mexican ancestry controls
Height
20881960
133,653 European ancestry individuals
Type 2 diabetes
28869590
6,353 South Asian ancestry cases, 7,179 South Asian ancestry controls, 3,871 European ancestry cases, 16,427 European ancestry controls, 34,840 cases, 114,981 controls

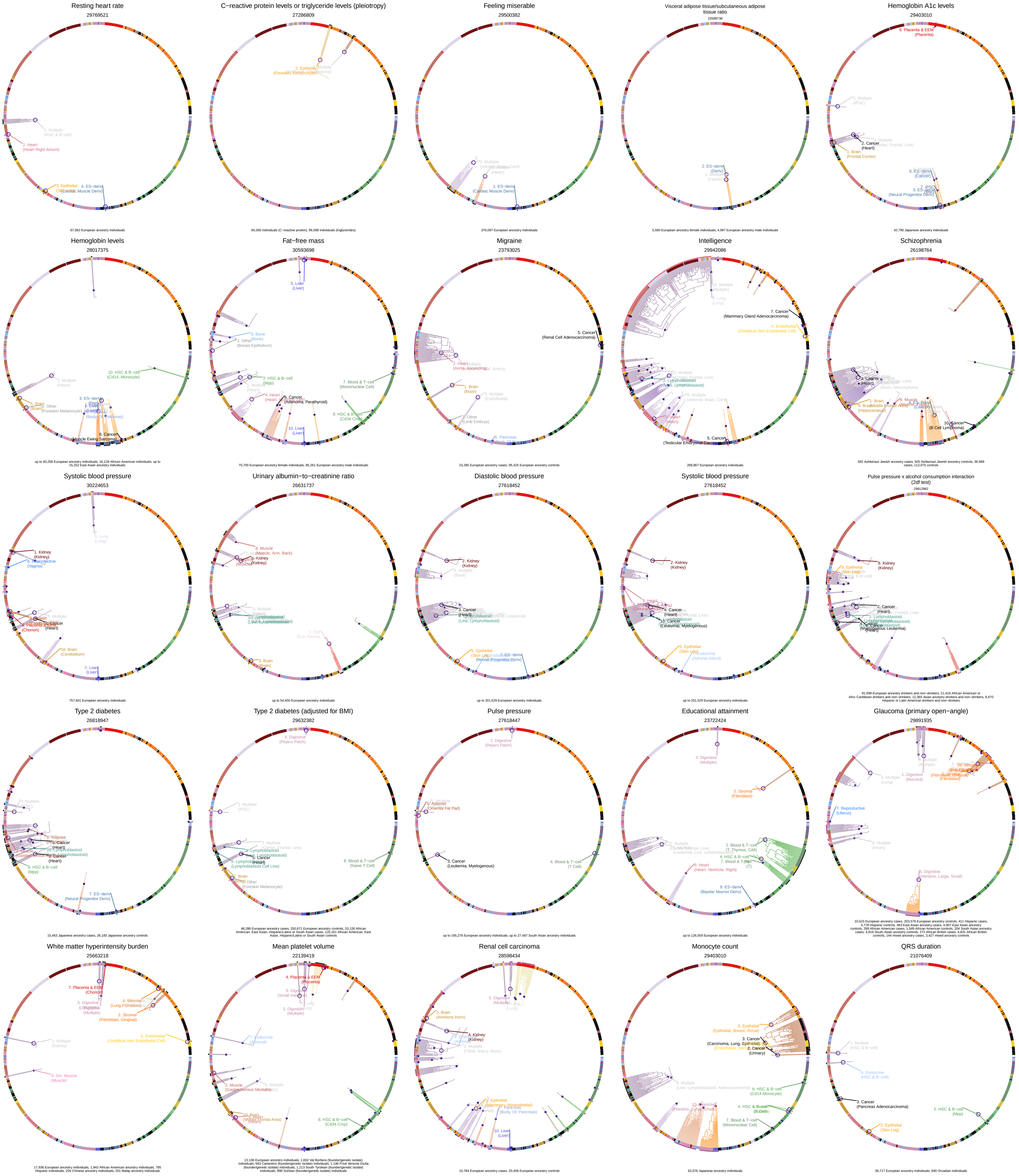
Resting heart rate
29769521
67,062 European ancestry individuals
C−reactive protein levels or triglyceride levels (pleiotropy)
27286809
65,000 individuals (C−reactive protein), 96,598 individuals (triglycerides)
Feeling miserable
29500382
376,097 European ancestry individuals
Visceral adipose tissue/subcutaneous adipose tissue ratio
22589738
5,560 European ancestry female individuals, 4,997 European ancestry male individuals
Hemoglobin A1c levels
29403010
42,790 Japanese ancestry individuals
Hemoglobin levels
28017375
up to 40,258 European ancestry individuals, 16,128 African American individuals, up to 15,252 East Asian ancestry individuals.
Fat−free mass
30593698
70,700 European ancestry female individuals, 85,261 European ancestry male individuals
Migraine
23793025
23,285 European ancestry cases, 95,425 European ancestry controls
Intelligence
29942086
269,867 European ancestry individuals
Schizophrenia
26198764
592 Ashkenazi Jewish ancestry cases, 505 Ashkenazi Jewish ancestry controls, 36,989 cases, 113,075 controls
Systolic blood pressure
30224653
757,601 European ancestry individuals
Urinary albumin−to−creatinine ratio
26631737
up to 54,450 European ancestry individuals
Diastolic blood pressure
27618452
up to 201,529 European ancestry individuals
Systolic blood pressure
27618452
up to 201,529 European ancestry individuals
Pulse pressure x alcohol consumption interaction (2df test)
29912962
91,099 European ancestry drinkers and non−drinkers, 21,416 African American or Afro−Caribbean drinkers and non−drinkers, 12,365 Asian ancestry drinkers and non−drinkers, 8,470 Hispanic or Latin American drinkers and non−drinkers
Type 2 diabetes
26818947
15,463 Japanese ancestry cases, 26,183 Japanese ancestry controls
Type 2 diabetes (adjusted for BMI)
29632382
48,286 European ancestry cases, 250,671 European ancestry controls, 33,126 African American, East Asian, Hispanic/Latino or South Asian cases, 120,161 African American, East Asian, Hispanic/Latino or South Asian controls
Pulse pressure
27618447
up to 165,276 European ancestry individuals, up to 27,487 South Asian ancestry individuals
Educational attainment
23722424
up to 126,559 European ancestry individuals
Glaucoma (primary open−angle)
29891935
10,523 European ancestry cases, 203,579 European ancestry controls, 411 Hispanic cases, 4,778 Hispanic controls, 463 East Asian ancestry cases, 4,907 East Asian ancestry controls, 298 African American cases, 1,549 African American controls, 204 South Asian ancestry cases, 4,916 South Asian ancestry controls, 272 African British cases, 4,831 African British controls, 144 mixed ancestry cases, 3,427 mixed ancestry controls
White matter hyperintensity burden
25663218
17,936 European ancestry individuals, 1,943 African American ancestry individuals, 795 Hispanic individuals, 204 Chinese ancestry individuals, 201 Malay ancestry individuals
Mean platelet volume
22139419
13,106 European ancestry individuals, 1,652 Val Borbera (founder/genetic isolate) individuals, 593 Carlantino (founder/genetic isolate) individuals, 1,146 Friuli Venezia Giulia (founder/genetic isolate) individuals, 1,213 South Tyrolean (founder/genetic isolate) individuals, 890 Sorbian (founder/genetic isolate) individuals
Renal cell carcinoma
28598434
10,784 European ancestry cases, 20,406 European ancestry controls
Monocyte count
29403010
62,076 Japanese ancestry individuals
QRS duration
21076409
39,717 European ancestry individuals, 690 Orcadian individuals

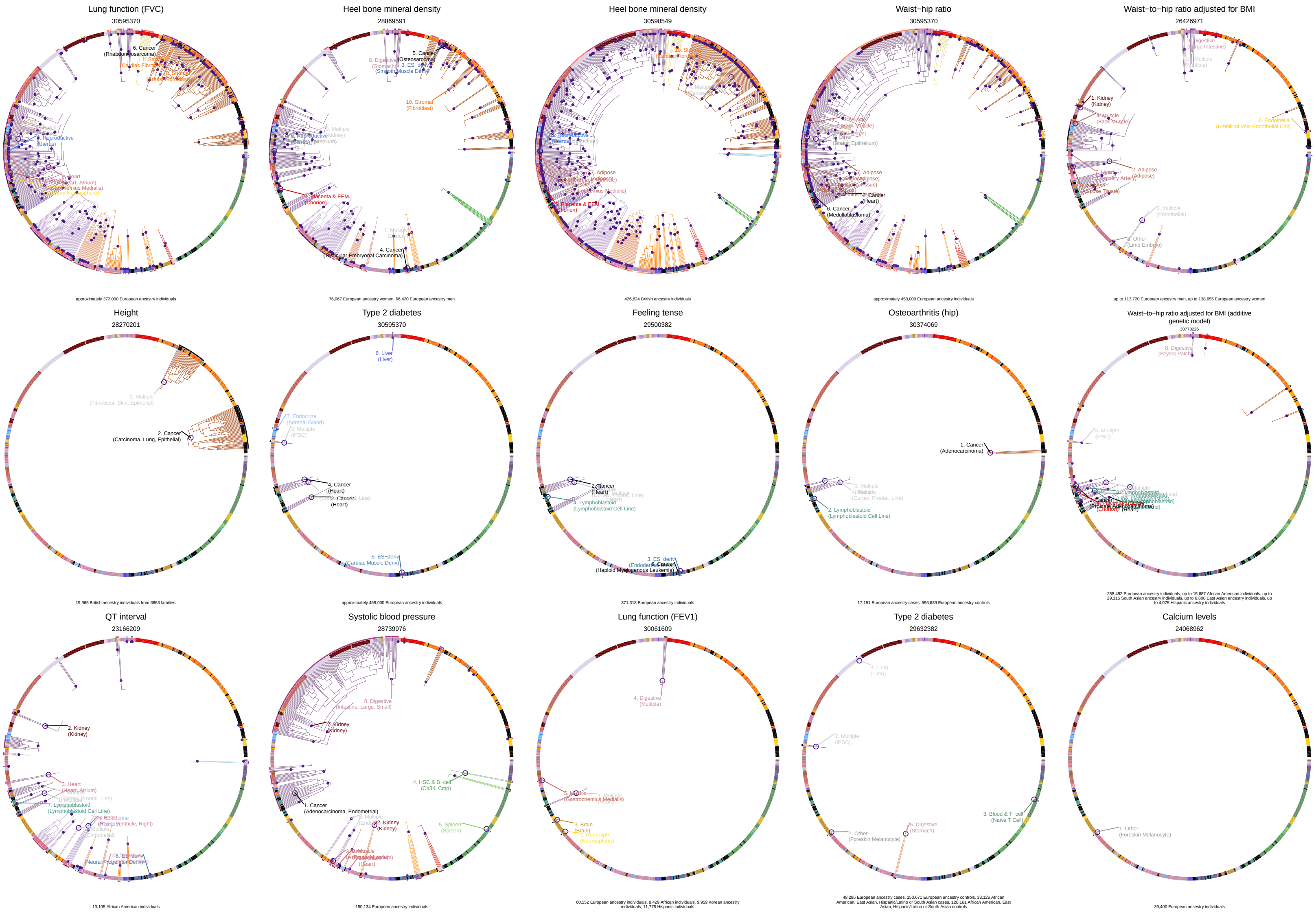
Lung function (FVC)
30595370
approximately 372,000 European ancestry individuals
Heel bone mineral density
28869591
76,067 European ancestry women, 66,420 European ancestry men
Heel bone mineral density
30598549
426,824 British ancestry individuals
Waist−hip ratio
30595370
approximately 458,000 European ancestry individuals
Waist−to−hip ratio adjusted for BMI
26426971
up to 113,720 European ancestry men, up to 138,655 European ancestry women
Height
28270201
19,965 British ancestry individuals from 6863 families.
Type 2 diabetes
30595370
approximately 459,000 European ancestry individuals
Feeling tense
29500382
371,318 European ancestry individuals
Osteoarthritis (hip)
30374069
17,151 European ancestry cases, 596,639 European ancestry controls
Waist−to−hip ratio adjusted for BMI (additive genetic model)
30778226
288,492 European ancestry individuals, up to 15,687 African American individuals, up to 29,315 South Asian ancestry individuals, up to 6,800 East Asian ancestry individuals, up to 4,075 Hispanic ancestry individuals
QT interval
23166209
13,105 African American individuals
Systolic blood pressure
28739976
150,134 European ancestry individuals
Lung function (FEV1)
30061609
60,552 European ancestry individuals, 8,429 African individuals, 9,959 Korean ancestry individuals, 11,775 Hispanic individuals
Type 2 diabetes
29632382
48,286 European ancestry cases, 250,671 European ancestry controls, 33,126 African American, East Asian, Hispanic/Latino or South Asian cases, 120,161 African American, East Asian, Hispanic/Latino or South Asian controls
Calcium levels
24068962
39,400 European ancestry individuals
